# Supplementary material for: Exploring differential gene expression and biomarker potential in systemic lupus erythematosus: a retrospective study
Source: PeerJ. 2025 Sep 8;13:e19891. doi: 10.7717/peerj.19891 (PMC12424612; doi:10.7717/peerj.19891)
Supplement: Supplemental Information 1 — The data include the Ct value for each sample, the Ct value for reference genes such as GAPDH, and the relative expression levels calculated using the ΔΔCt method. These data were used to validate the RNA sequencing results and further evaluate the potential of these genes as biomarkers for SLE. [file peerj-13-19891-s001.zip › qPCR/20241212 qPCR/1212plate1 SLE group.pdf]

cobas® 4800 User Defined Workflow (UDF)

20241212 xjplate1

Experiment

Creation Date	12/12/2024 8:31:42 PM	Last Modified Date	12/12/2024 9:36:26 PM
Operator	System Admin	Owner	System Admin
Start Time	12/12/2024 8:32:29 PM	End Time	12/12/2024 9:31:24 PM
Run State	Completed	Software Version	LCS480 1.5.1.62 SP3 - UDF 2.1.0.26
Macro		Macro Owner	
Macro Status			
Templates	gongying	Plate ID	
Test ID		Lot ID	
Color Comp ID			
Run Notes			

Programs

Program Name	Program		
Cycles	1	Analysis Mode	None

Target (°C)	Acquisition Mode	Hold (hh:mm:ss)	Ramp Rate (°C/s)	Acquisitions (per °C)	Sec Target (°C)	Step size (°C)	Step Delay (cycles)
95	None	00:00:30	4.40		0	0	0

Program Name	Program		
Cycles	40	Analysis Mode	Quantification

Target (°C)	Acquisition Mode	Hold (hh:mm:ss)	Ramp Rate (°C/s)	Acquisitions (per °C)	Sec Target (°C)	Step size (°C)	Step Delay (cycles)
95	None	00:00:15	4.40		0	0	0
60	Single	00:00:30	2.20		0	0	0

Program Name	Program		
Cycles	1	Analysis Mode	Melting Curves

Target (°C)	Acquisition Mode	Hold (hh:mm:ss)	Ramp Rate (°C/s)	Acquisitions (per °C)	Sec Target (°C)	Step size (°C)	Step Delay (cycles)
95	None	00:01:00	4.40		0	0	0
60	None	00:00:30	2.20		0	0	0
90	Continuous		0.11	5	0	0	0

Program Name	Program		
Cycles	1	Analysis Mode	None

Target (°C)	Acquisition Mode	Hold (hh:mm:ss)	Ramp Rate (°C/s)	Acquisitions (per °C)	Sec Target (°C)	Step size (°C)	Step Delay (cycles)

Target (°C)	Acquisition Mode	Hold (hh:mm:ss)	Ramp Rate (°C/s)	Acquisitions (per °C)	Sec Target (°C)	Step size (°C)	Step Delay (cycles)
40	None	00:00:30	2.20		0	0	0

Samples

Sample Count	96
Subset	All Samples

Pos	Name	ID	Repl. Of	Sample Notes
A1	GAPDH 1			
A2	GAPDH 1			
A3	GAPDH 1			
A4	GAPDH 2			
A5	GAPDH 2			
A6	GAPDH 2			
A7	GAPDH 3			
A8	GAPDH 3			
A9	GAPDH 3			
A10	GAPDH 4			
A11	GAPDH 4			
A12	GAPDH 4			
B1	FCER1A 1			
B2	FCER1A 1			
B3	FCER1A 1			
B4	FCER1A 2			
B5	FCER1A 2			
B6	FCER1A 2			
B7	FCER1A 3			
B8	FCER1A 3			
B9	FCER1A 3			
B10	FCER1A 4			
B11	FCER1A 4			
B12	FCER1A 4			
C1	RGS1 1			
C2	RGS1 1			
C3	RGS1 1			
C4	RGS1 2			
C5	RGS1 2			
C6	RGS1 2			
C7	RGS1 3			
C8	RGS1 3			
C9	RGS1 3			
C10	RGS1 4			
C11	RGS1 4			
C12	RGS1 4			

Samples

Sample Count	96
Subset	All Samples

Pos	Name	ID	Repl. Of	Sample Notes
D1	GAPDH 5			
D2	GAPDH 5			
D3	GAPDH 5			
D4	GAPDH 7			
D5	GAPDH 7			
D6	GAPDH 7			
D7	GAPDH 11			
D8	GAPDH 11			
D9	GAPDH 11			
D10	GAPDH 14			
D11	GAPDH 14			
D12	GAPDH 14			
E1	FCER1A 5			
E2	FCER1A 5			
E3	FCER1A 5			
E4	FCER1A 7			
E5	FCER1A 7			
E6	FCER1A 7			
E7	FCER1A 11			
E8	FCER1A 11			
E9	FCER1A 11			
E10	FCER1A 14			
E11	FCER1A 14			
E12	FCER1A 14			
F1	RGS1 5			
F2	RGS1 5			
F3	RGS1 5			
F4	RGS1 7			
F5	RGS1 7			
F6	RGS1 7			
F7	RGS1 11			
F8	RGS1 11			
F9	RGS1 11			
F10	RGS1 14			
F11	RGS1 14			
F12	RGS1 14			
G1				
G2				
G3				

Samples

Sample Count	96
Subset	All Samples

Pos	Name	ID	Repl. Of	Sample Notes
G4				
G5				
G6				
G7				
G8				
G9				
G10				
G11				
G12				
H1				
H2				
H3				
H4				
H5				
H6				
H7				
H8				
H9				
H10				
H11				
H12				

Instrument

Name	Z480	Serial Number	56319

Revision History

Revision	Date	User	Reason

Abs Quant/2nd Derivative Max for All Samples (Abs Quant/2nd Derivative Max)

Settings

Channel	465-510		
Color Compensation	Off		
Program	Program	Units	
Mode	High Confidence		

Subset Name	All Samples

Results

> -Inate	CPposal	l N(lasmteive cycles) has higher uncertainty	Type	CP	Concentration	Standard	Status

Results

Inc	Pos	Name	Type	CP	Concentration	Standard	Status
☑	A1	GAPDH 1	Unknown	27.49			
☑	A2	GAPDH 1	Unknown	27.18			
☑	A3	GAPDH 1	Unknown	27.32			
☑	A4	GAPDH 2	Unknown	28.26			
☑	A5	GAPDH 2	Unknown	28.78			
☑	A6	GAPDH 2	Unknown	28.44			
☑	A7	GAPDH 3	Unknown	21.25			
☑	A8	GAPDH 3	Unknown	21.23			
☑	A9	GAPDH 3	Unknown	21.12			
☑	A10	GAPDH 4	Unknown	31.94			
☑	A11	GAPDH 4	Unknown	32.34			
☑	A12	GAPDH 4	Unknown	32.15			
☑	B1	FCER1A 1	Unknown	30.09			
☑	B2	FCER1A 1	Unknown	31.17			
☑	B3	FCER1A 1	Unknown	31.67			
☑	B4	FCER1A 2	Unknown	32.38			
☑	B5	FCER1A 2	Unknown	33.16			
☑	B6	FCER1A 2	Unknown	31.99			
☑	B7	FCER1A 3	Unknown	25.45			
☑	B8	FCER1A 3	Unknown	25.54			
☑	B9	FCER1A 3	Unknown	25.32			
☑	B10	FCER1A 4	Unknown	29.86			
☑	B11	FCER1A 4	Unknown	31.96			
☑	B12	FCER1A 4	Unknown	30.85			
☑	C1	RGS1 1	Unknown	35.00			>
☑	C2	RGS1 1	Unknown	34.28			
☑	C3	RGS1 1	Unknown	35.00			>
☑	C4	RGS1 2	Unknown	35.00			>
☑	C5	RGS1 2	Unknown	35.00			>
☑	C6	RGS1 2	Unknown	35.00			>
☑	C7	RGS1 3	Unknown	26.45			
☑	C8	RGS1 3	Unknown	26.48			
☑	C9	RGS1 3	Unknown	27.27			
☑	C10	RGS1 4	Unknown	35.00			>
☑	C11	RGS1 4	Unknown				
☑	C12	RGS1 4	Unknown	35.00			>
☑	D1	GAPDH 5	Unknown	24.44			

> - Late Cp call (last five cycles) has higher uncertainty

Results

Inc	Pos	Name	Type	CP	Concentration	Standard	Status
☑	D2	GAPDH 5	Unknown	24.50			
☑	D3	GAPDH 5	Unknown	24.51			
☑	D4	GAPDH 7	Unknown	22.87			
☑	D5	GAPDH 7	Unknown	22.83			
☑	D6	GAPDH 7	Unknown	22.79			
☑	D7	GAPDH 11	Unknown	29.43			
☑	D8	GAPDH 11	Unknown	29.73			
☑	D9	GAPDH 11	Unknown	29.22			
☑	D10	GAPDH 14	Unknown	20.68			
☑	D11	GAPDH 14	Unknown	20.64			
☑	D12	GAPDH 14	Unknown	20.67			
☑	E1	FCER1A 5	Unknown	29.94			
☑	E2	FCER1A 5	Unknown	29.68			
☑	E3	FCER1A 5	Unknown	29.28			
☑	E4	FCER1A 7	Unknown	28.35			
☑	E5	FCER1A 7	Unknown	27.90			
☑	E6	FCER1A 7	Unknown	28.04			
☑	E7	FCER1A 11	Unknown	31.14			
☑	E8	FCER1A 11	Unknown	30.93			
☑	E9	FCER1A 11	Unknown	30.88			
☑	E10	FCER1A 14	Unknown	28.65			
☑	E11	FCER1A 14	Unknown	28.78			
☑	E12	FCER1A 14	Unknown	28.62			
☑	F1	RGS1 5	Unknown	30.73			
☑	F2	RGS1 5	Unknown	30.63			
☑	F3	RGS1 5	Unknown	30.44			
☑	F4	RGS1 7	Unknown	28.91			
☑	F5	RGS1 7	Unknown	28.53			
☑	F6	RGS1 7	Unknown	28.72			
☑	F7	RGS1 11	Unknown	35.00			>
☑	F8	RGS1 11	Unknown	35.00			>
☑	F9	RGS1 11	Unknown	35.00			>
☑	F10	RGS1 14	Unknown	29.01			
☑	F11	RGS1 14	Unknown	28.68			
☑	F12	RGS1 14	Unknown	28.90			
☑	G1		Unknown				
☑	G2		Unknown				

> - Late Cp call (last five cycles) has higher uncertainty

Results

Inc	Pos	Name	Type	CP	Concentration	Standard	Status
☑	G3		Unknown				
☑	G4		Unknown				
☑	G5		Unknown				
☑	G6		Unknown				
☑	G7		Unknown				
☑	G8		Unknown				
☑	G9		Unknown				
☑	G10		Unknown				
☑	G11		Unknown				
☑	G12		Unknown				
☑	H1		Unknown				
☑	H2		Unknown				
☑	H3		Unknown				
☑	H4		Unknown				
☑	H5		Unknown				
☑	H6		Unknown				
☑	H7		Unknown				
☑	H8		Unknown				
☑	H9		Unknown				
☑	H10		Unknown				
☑	H11		Unknown				
☑	H12		Unknown				

> - Late Cp call (last five cycles) has higher uncertainty

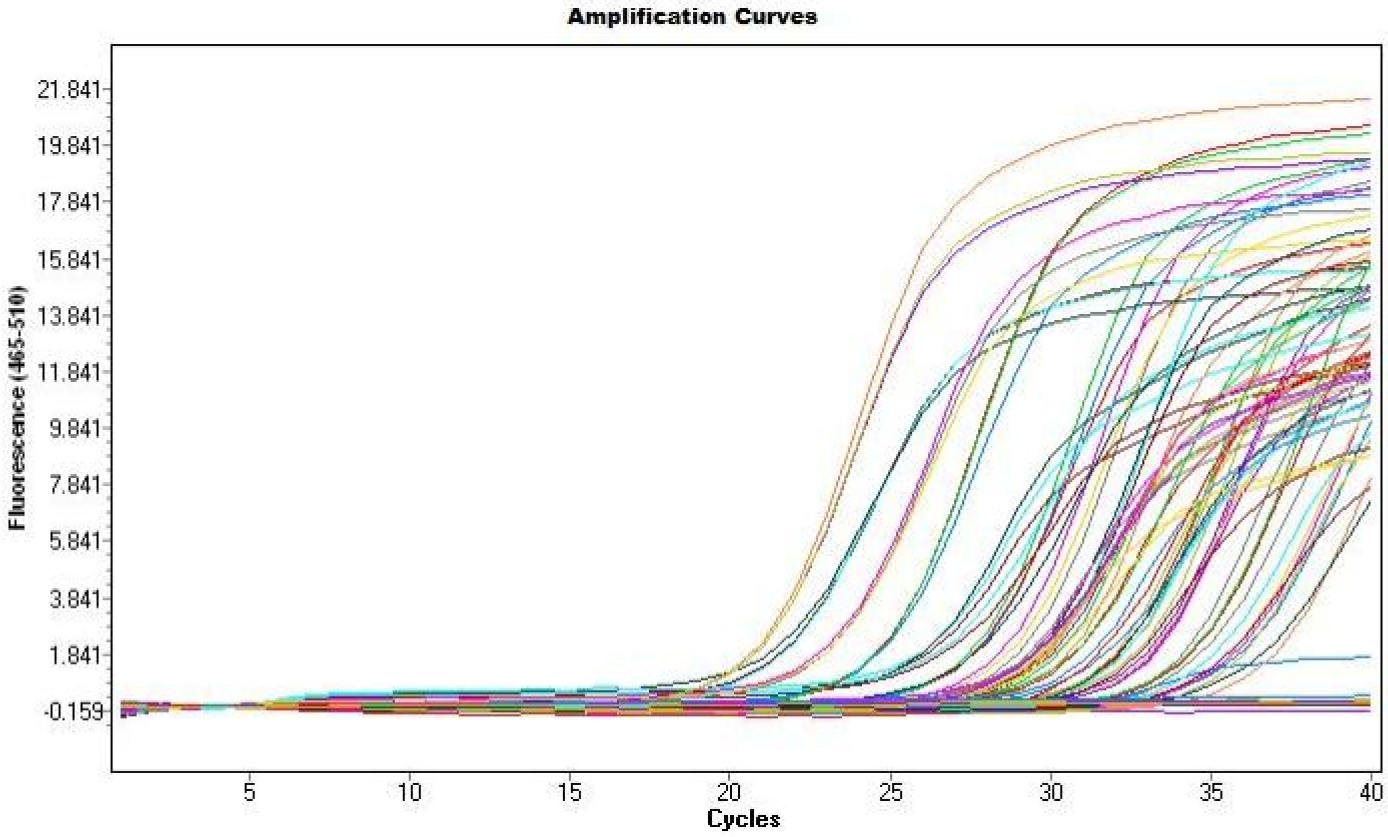

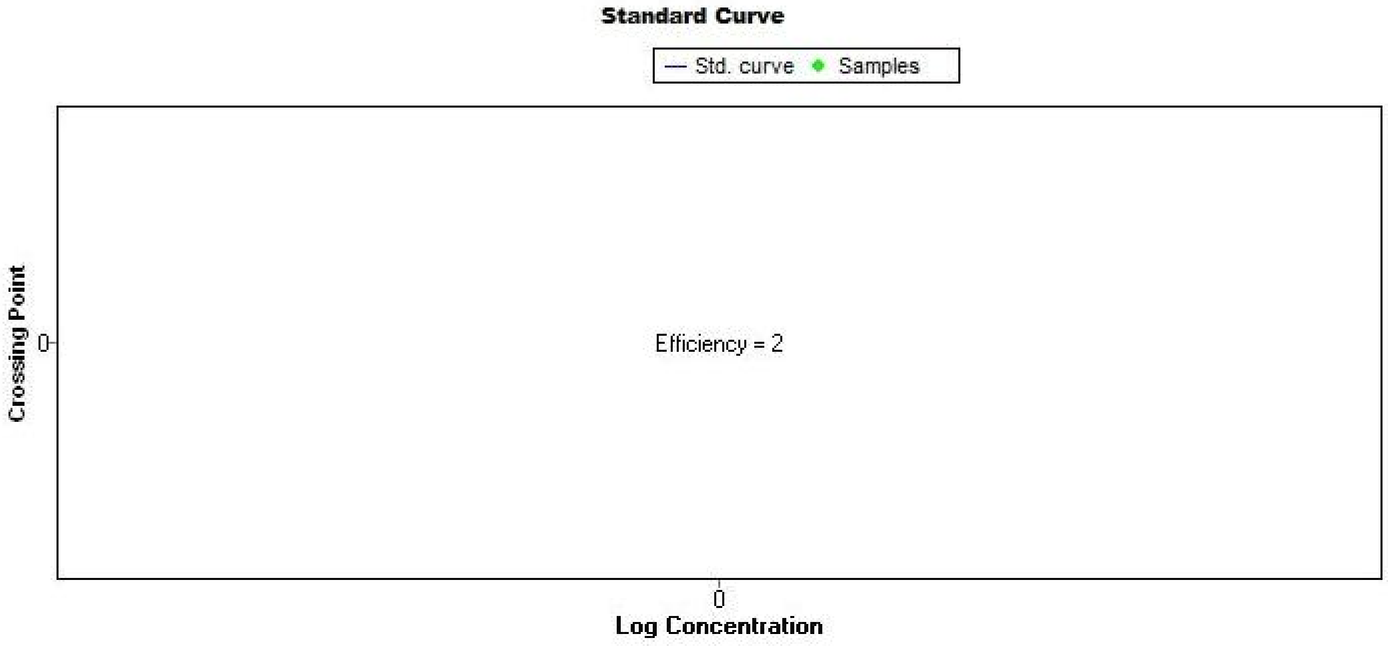

Analysis Notes	

Abs Quant/Fit Points for All Samples (Abs Quant/Fit Points)

Settings

Channel	465-510				
Color Compensation	Off				
Standard Curve	Set Efficiency (2.00)				
Program	Program			Units	
First Cycle	1	Last Cycle	40	Background	2-6
Noiseband Method	Auto	Noiseband	1.0130	STD Dev Multiplier	12.0000
# of Fit Points	2	Threshold Method	Auto	Threshold	1.0130

Subset Name	All Samples

Results

Inc	Pos	Name	Type	CP	Concentration	Standard	Status
☑	A1	GAPDH 1	Unknown	25.59			
☑	A2	GAPDH 1	Unknown	25.72			
☑	A3	GAPDH 1	Unknown	25.63			
☑	A4	GAPDH 2	Unknown	26.64			
☑	A5	GAPDH 2	Unknown	27.17			
☑	A6	GAPDH 2	Unknown	26.81			
☑	A7	GAPDH 3	Unknown	19.21			
☑	A8	GAPDH 3	Unknown	19.53			
☑	A9	GAPDH 3	Unknown				
☑	A10	GAPDH 4	Unknown	30.49			
☑	A11	GAPDH 4	Unknown	30.99			
☑	A12	GAPDH 4	Unknown	30.70			
☑	B1	FCER1A 1	Unknown	32.86			
☑	B2	FCER1A 1	Unknown	30.35			
☑	B3	FCER1A 1	Unknown	30.53			
☑	B4	FCER1A 2	Unknown	31.57			
☑	B5	FCER1A 2	Unknown	32.58			
☑	B6	FCER1A 2	Unknown	31.46			
☑	B7	FCER1A 3	Unknown	21.46			
☑	B8	FCER1A 3	Unknown	4.68			
☑	B9	FCER1A 3	Unknown	22.67			
☑	B10	FCER1A 4	Unknown	28.95			
☑	B11	FCER1A 4	Unknown	31.56			
☑	B12	FCER1A 4	Unknown	30.41			
☑	C1	RGS1 1	Unknown	33.80			
☑	C2	RGS1 1	Unknown	32.63			
☑	C3	RGS1 1	Unknown	32.49			

Results

Inc	Pos	Name	Type	CP	Concentration	Standard	Status
☑	C4	RGS1 2	Unknown	33.80			
☑	C5	RGS1 2	Unknown	32.92			
☑	C6	RGS1 2	Unknown	33.61			
☑	C7	RGS1 3	Unknown	20.58			
☑	C8	RGS1 3	Unknown	9.76			
☑	C9	RGS1 3	Unknown	25.46			
☑	C10	RGS1 4	Unknown	35.31			
☑	C11	RGS1 4	Unknown				
☑	C12	RGS1 4	Unknown	34.44			
☑	D1	GAPDH 5	Unknown	22.79			
☑	D2	GAPDH 5	Unknown	22.80			
☑	D3	GAPDH 5	Unknown	22.67			
☑	D4	GAPDH 7	Unknown	19.71			
☑	D5	GAPDH 7	Unknown	20.60			
☑	D6	GAPDH 7	Unknown	20.44			
☑	D7	GAPDH 11	Unknown	27.78			
☑	D8	GAPDH 11	Unknown	28.22			
☑	D9	GAPDH 11	Unknown	27.54			
☑	D10	GAPDH 14	Unknown	18.86			
☑	D11	GAPDH 14	Unknown	18.76			
☑	D12	GAPDH 14	Unknown	18.85			
☑	E1	FCER1A 5	Unknown	29.53			
☑	E2	FCER1A 5	Unknown	28.88			
☑	E3	FCER1A 5	Unknown	28.49			
☑	E4	FCER1A 7	Unknown	26.54			
☑	E5	FCER1A 7	Unknown	27.19			
☑	E6	FCER1A 7	Unknown	27.68			
☑	E7	FCER1A 11	Unknown	30.96			
☑	E8	FCER1A 11	Unknown	30.59			
☑	E9	FCER1A 11	Unknown	30.50			
☑	E10	FCER1A 14	Unknown	27.90			
☑	E11	FCER1A 14	Unknown	28.32			
☑	E12	FCER1A 14	Unknown	27.90			
☑	F1	RGS1 5	Unknown	29.61			
☑	F2	RGS1 5	Unknown	29.92			
☑	F3	RGS1 5	Unknown	28.61			
☑	F4	RGS1 7	Unknown	27.30			

Results

Inc	Pos	Name	Type	CP	Concentration	Standard	Status
☑	F5	RGS1 7	Unknown	27.13			
☑	F6	RGS1 7	Unknown	28.00			
☑	F7	RGS1 11	Unknown	33.60			
☑	F8	RGS1 11	Unknown	33.29			
☑	F9	RGS1 11	Unknown	34.56			
☑	F10	RGS1 14	Unknown	27.57			
☑	F11	RGS1 14	Unknown	27.48			
☑	F12	RGS1 14	Unknown	27.75			
☑	G1		Unknown				
☑	G2		Unknown				
☑	G3		Unknown				
☑	G4		Unknown				
☑	G5		Unknown				
☑	G6		Unknown				
☑	G7		Unknown				
☑	G8		Unknown				
☑	G9		Unknown				
☑	G10		Unknown				
☑	G11		Unknown				
☑	G12		Unknown				
☑	H1		Unknown				
☑	H2		Unknown				
☑	H3		Unknown				
☑	H4		Unknown				
☑	H5		Unknown				
☑	H6		Unknown				
☑	H7		Unknown				
☑	H8		Unknown				
☑	H9		Unknown				
☑	H10		Unknown				
☑	H11		Unknown				
☑	H12		Unknown				

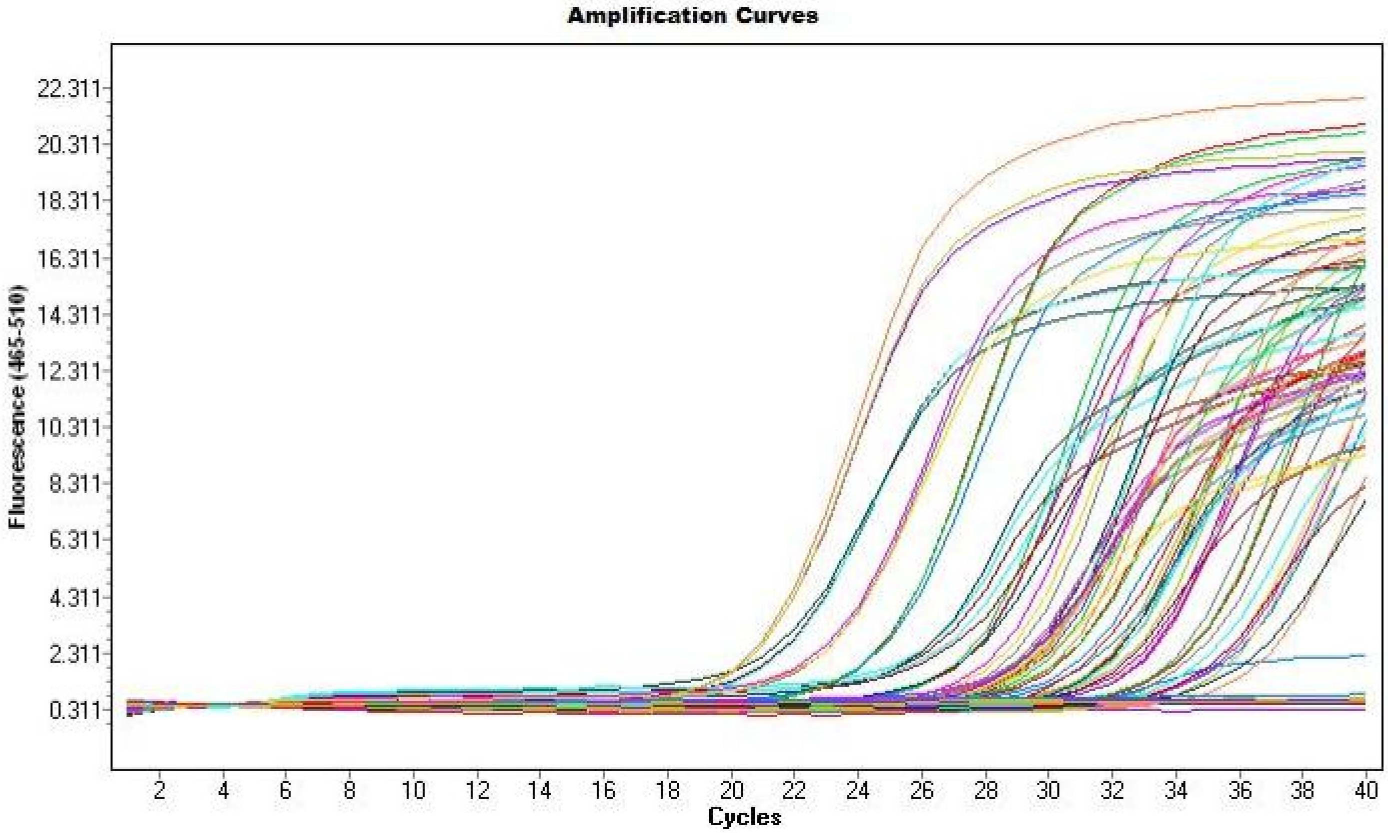
Amplification Curves
Fluorescence (465-510)
22.311
20.311
18.311
16.311
14.311
12.311
10.311
8.311
6.311
4.311
2.311
0.311
2
4
6
8
10
12
14
16
18
20
22
24
26
28
30
32
34
36
38
40
Cycles

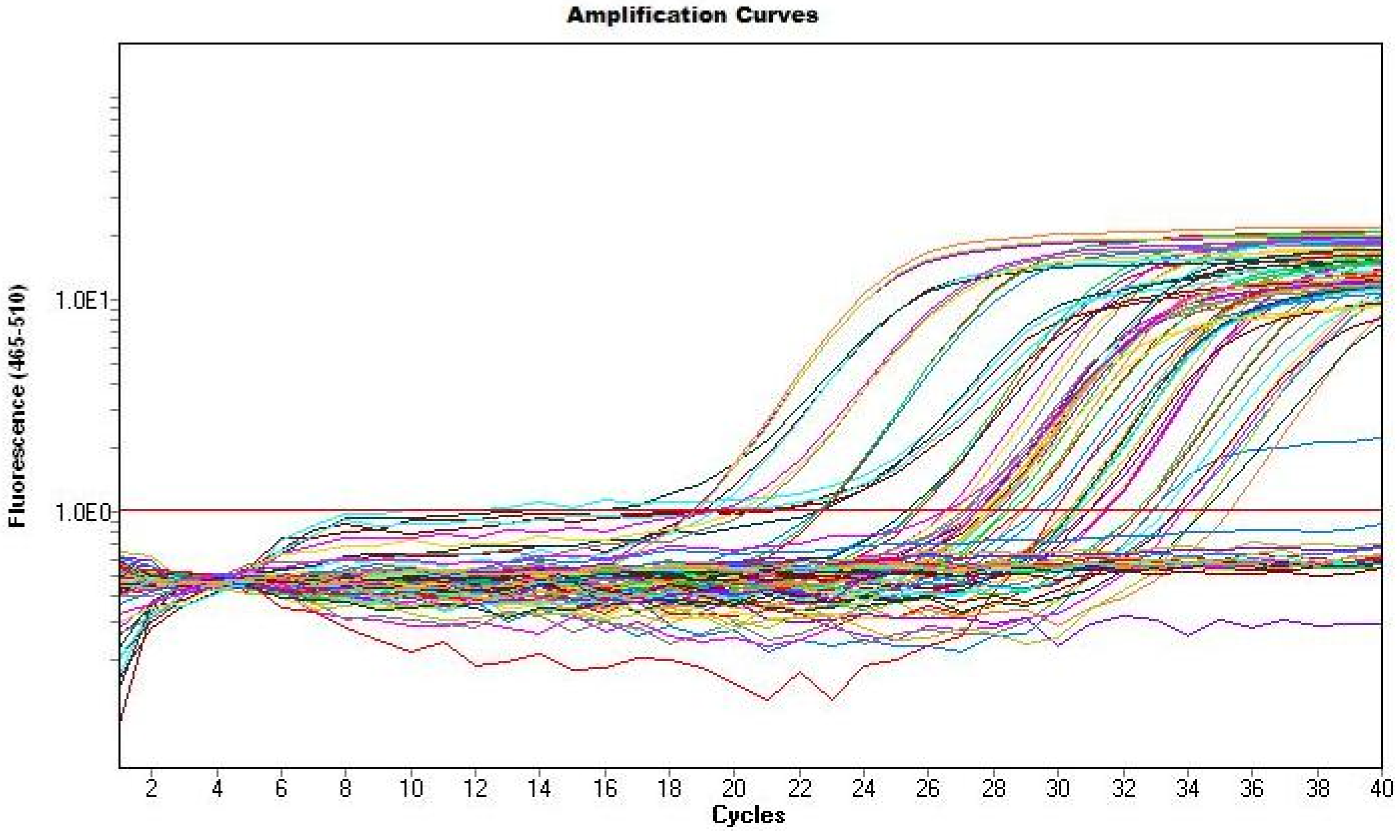
Amplification Curves
Fluorescence (465-510)
1.0E1
1.0E0
2
4
6
8
10
12
14
16
18
20
22
24
26
28
30
32
34
36
38
40
Cycles

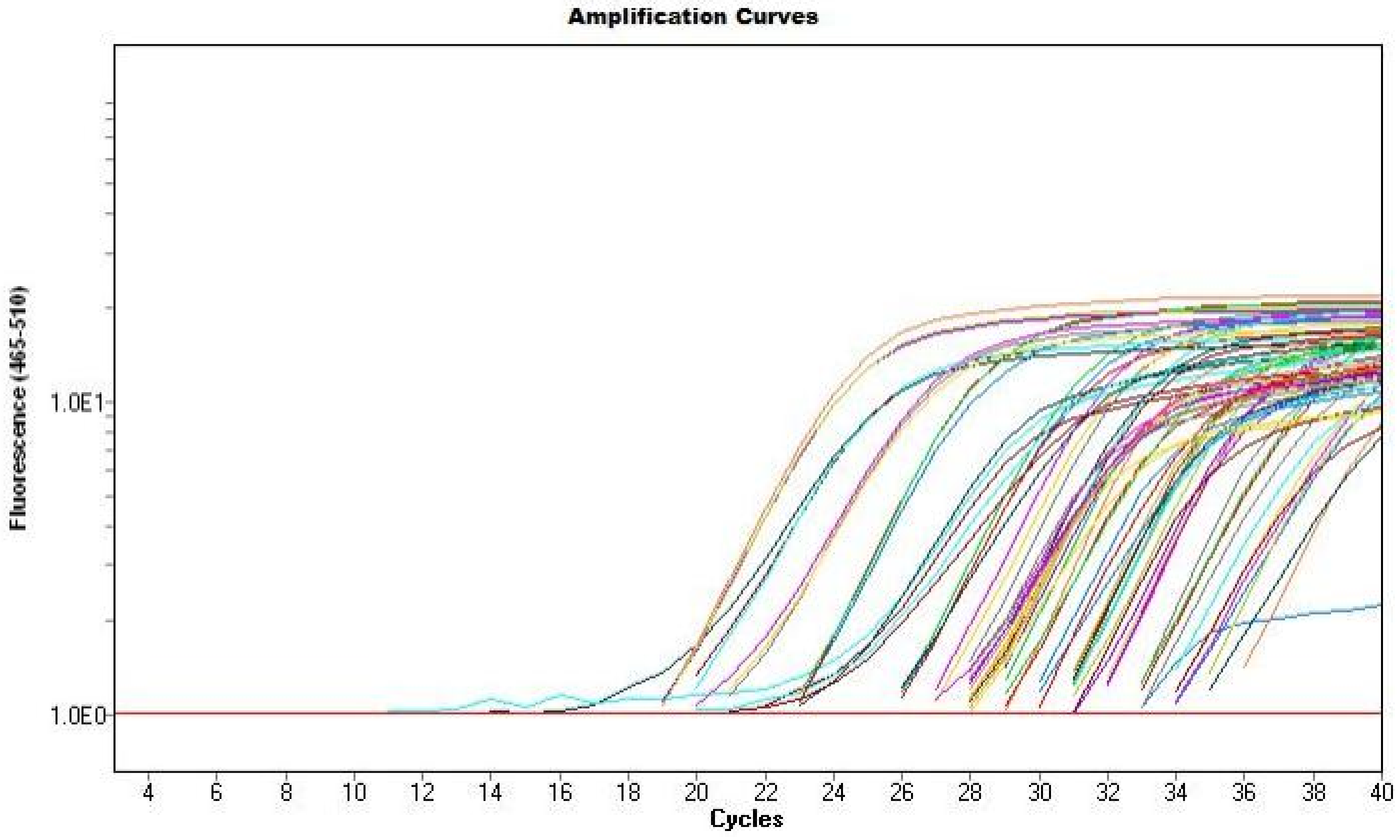

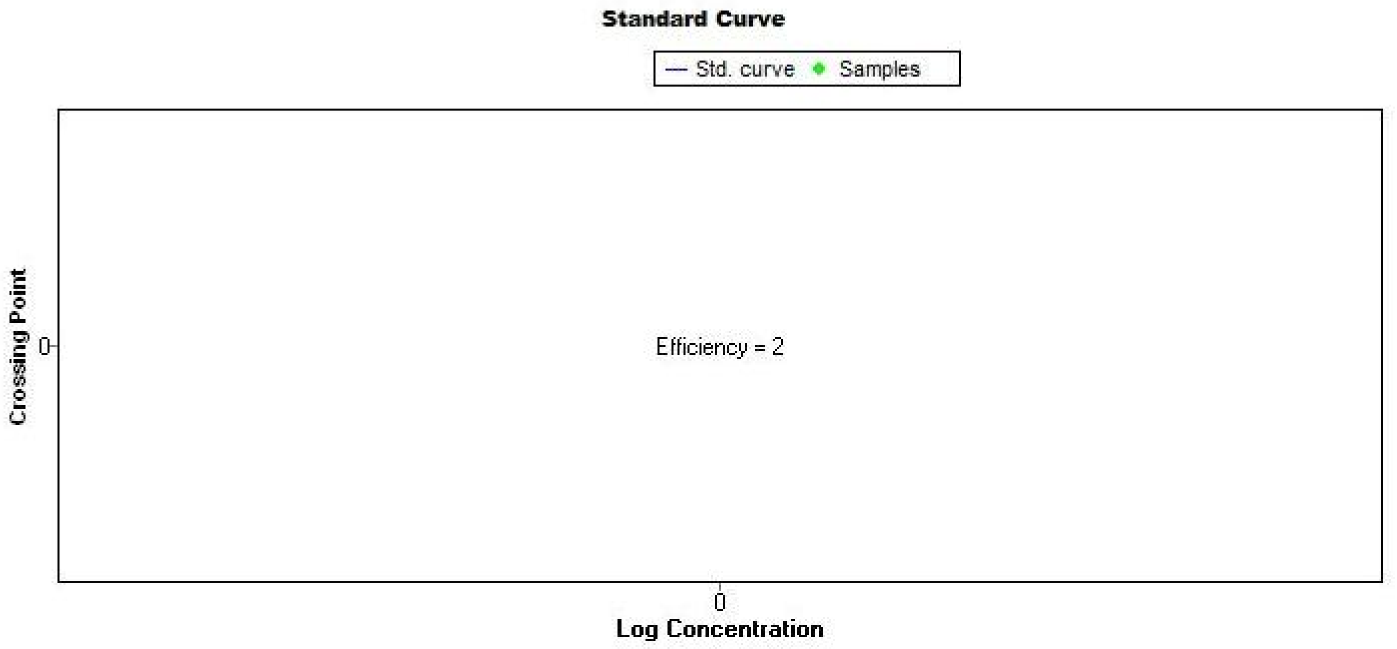

Analysis Notes	

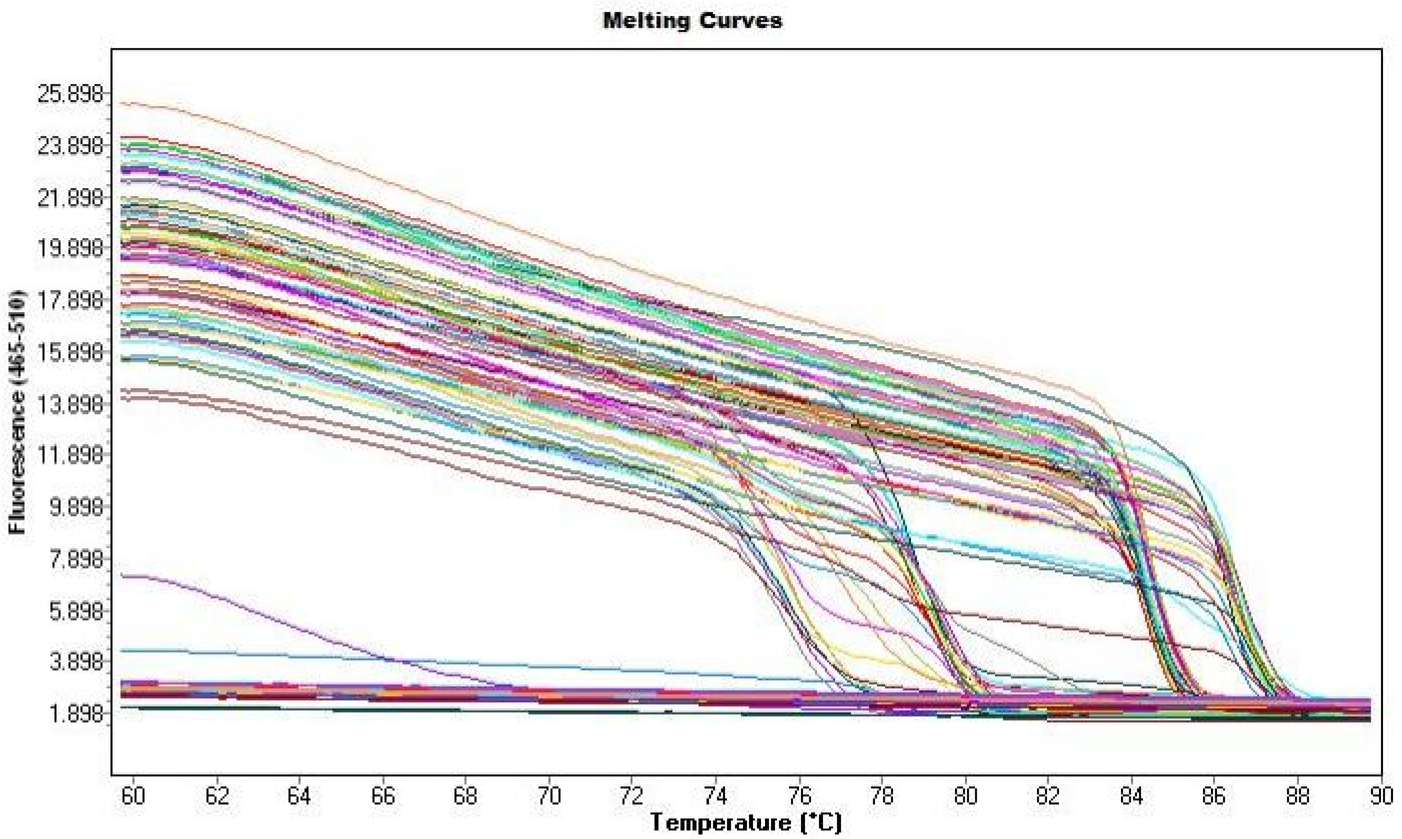
Melting Curves
Fluorescence (465-510)
25.898
23.898
21.898
19.898
17.898
15.898
13.898
11.898
9.898
7.898
5.898
3.898
1.898
60
62
64
66
68
70
72
74
76
78
80
82
84
86
88
90
Temperature (°C)

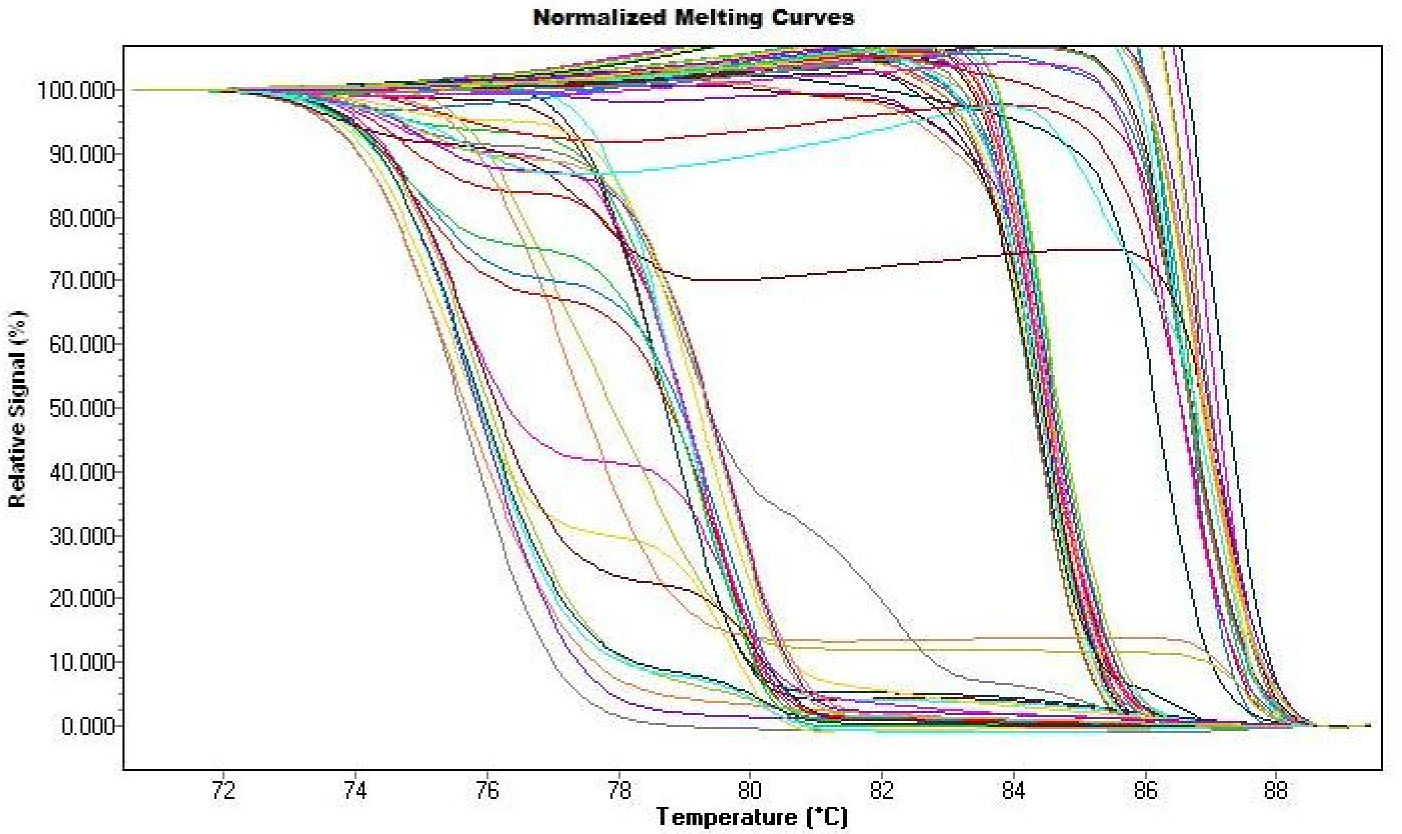
Normalized Melting Curves
Relative Signal (%)
100.000
90.000
80.000
70.000
60.000
50.000
40.000
30.000
20.000
10.000
0.000
72
74
76
78
80
82
84
86
88
Temperature (°C)

Normalized and Shifted Melting Curves

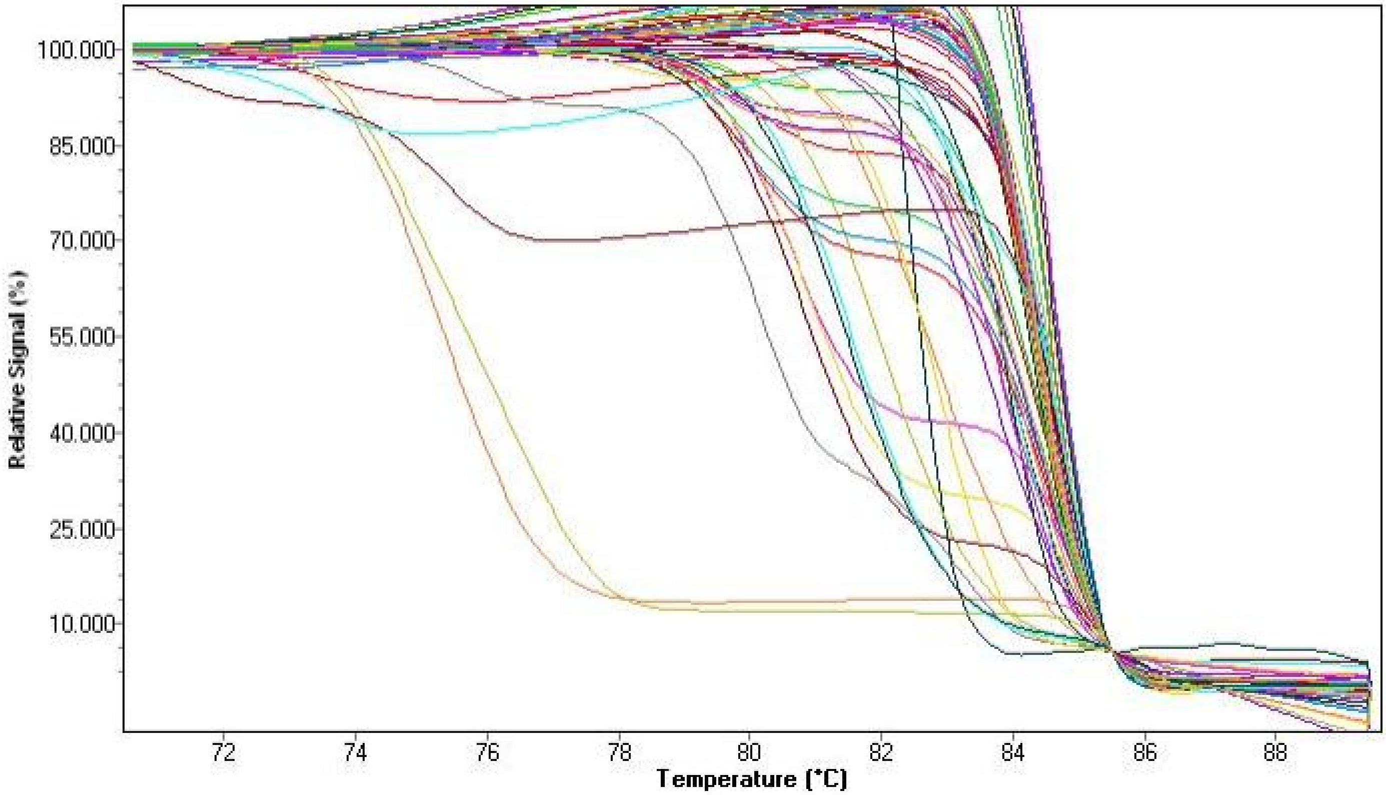

Normalized and Temp-Shifted Difference Plot

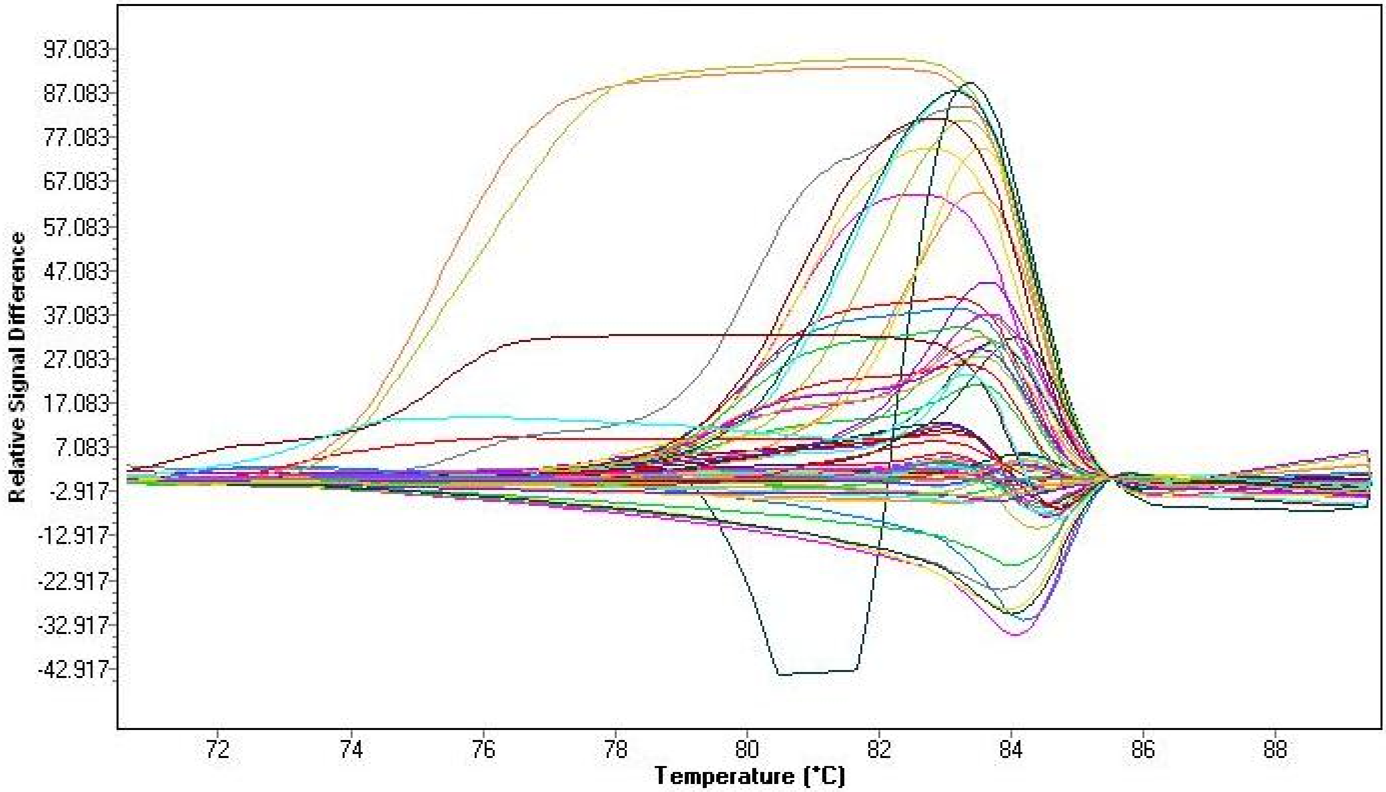

Analysis Notes	

Melt Curve Genotyping for All Samples (Melt Curve Genotyping)

Channel	465-510	Color Compensation	Off				
Progam Name	Program						
Stds Settings	Auto-Group						
Sensitivity	Normal	Temp Range	63-86	Score	0.7	Res	0.1

Subset Name	All Samples

Results

Inc	Pos.	Sample Name	Sample Type	Group	Score	Resolution	Status
☑	A1	GAPDH 1	Unknown	1	0.98	0.95	
☑	A2	GAPDH 1	Unknown	1	0.96	0.94	
☑	A3	GAPDH 1	Unknown	1	0.98	0.96	
☑	A4	GAPDH 2	Unknown	1	0.98	0.96	
☑	A5	GAPDH 2	Unknown	1	0.97	0.95	
☑	A6	GAPDH 2	Unknown	1	0.98	0.96	
☑	A7	GAPDH 3	Unknown	1	0.99	0.95	
☑	A8	GAPDH 3	Unknown	1	0.99	0.94	
☑	A9	GAPDH 3	Unknown	1	0.96	0.93	
☑	A10	GAPDH 4	Unknown	1	0.95	0.92	
☑	A11	GAPDH 4	Unknown	1	0.97	0.91	
☑	A12	GAPDH 4	Unknown	1	0.98	0.94	
☑	B1	FCER1A 1	Unknown	2	0.82	0.76	
☑	B2	FCER1A 1	Unknown	2	0.89	0.75	
☑	B3	FCER1A 1	Unknown	2	0.86	0.80	
☑	B4	FCER1A 2	Unknown	2	0.79	0.51	
☑	B5	FCER1A 2	Unknown	2	0.94	0.87	
☑	B6	FCER1A 2	Unknown	2	0.86	0.64	
☑	B7	FCER1A 3	Unknown	2	0.81	0.75	
☑	B8	FCER1A 3	Unknown	2	0.85	0.78	
☑	B9	FCER1A 3	Unknown	2	0.74	0.70	
☑	B10	FCER1A 4	Unknown	2	0.96	0.87	
☑	B11	FCER1A 4	Unknown	2	0.95	0.86	
☑	B12	FCER1A 4	Unknown	2	0.96	0.85	
☑	C1	RGS1 1	Unknown	1	0.94	0.77	
☑	C2	RGS1 1	Unknown	1	0.92	0.58	
☑	C3	RGS1 1	Unknown	1	0.98	0.83	
☑	C4	RGS1 2	Unknown	1	0.96	0.83	
☑	C5	RGS1 2	Unknown	1	0.99	0.81	
☑	C6	RGS1 2	Unknown	1	0.97	0.83	
☑	C7	RGS1 3	Unknown	1	1.00	0.80	

Results

Inc	Pos.	Sample Name	Sample Type	Group	Score	Resolution	Status
☑	C8	RGS1 3	Unknown	1	0.99	0.79	
☑	C9	RGS1 3	Unknown	1	0.93	0.86	
☑	C10	RGS1 4	Unknown	2	0.97	0.73	
☑	C11	RGS1 4	Unknown	3	1.00	0.99	
☑	C12	RGS1 4	Unknown	2	1.00	0.80	
☑	D1	GAPDH 5	Unknown	1	0.99	0.95	
☑	D2	GAPDH 5	Unknown	1	0.98	0.95	
☑	D3	GAPDH 5	Unknown	1	0.98	0.94	
☑	D4	GAPDH 7	Unknown	1	0.98	0.94	
☑	D5	GAPDH 7	Unknown	1	0.98	0.95	
☑	D6	GAPDH 7	Unknown	1	0.96	0.94	
☑	D7	GAPDH 11	Unknown	1	0.95	0.92	
☑	D8	GAPDH 11	Unknown	1	0.98	0.95	
☑	D9	GAPDH 11	Unknown	1	0.95	0.93	
☑	D10	GAPDH 14	Unknown	1	0.96	0.93	
☑	D11	GAPDH 14	Unknown	1	0.97	0.94	
☑	D12	GAPDH 14	Unknown	1	0.98	0.93	
☑	E1	FCER1A 5	Unknown	2	0.78	0.54	
☑	E2	FCER1A 5	Unknown	2	0.76	0.50	
☑	E3	FCER1A 5	Unknown	2	0.84	0.62	
☑	E4	FCER1A 7	Unknown	2	0.94	0.81	
☑	E5	FCER1A 7	Unknown	2	0.91	0.69	
☑	E6	FCER1A 7	Unknown	2	0.92	0.83	
☑	E7	FCER1A 11	Unknown	2	0.93	0.73	
☑	E8	FCER1A 11	Unknown	2	0.97	0.85	
☑	E9	FCER1A 11	Unknown	2	0.97	0.86	
☑	E10	FCER1A 14	Unknown	2	0.93	0.78	
☑	E11	FCER1A 14	Unknown	2	0.92	0.78	
☑	E12	FCER1A 14	Unknown	2	0.92	0.81	
☑	F1	RGS1 5	Unknown	1	0.98	0.75	
☑	F2	RGS1 5	Unknown	1	0.95	0.78	
☑	F3	RGS1 5	Unknown	1	0.99	0.78	
☑	F4	RGS1 7	Unknown	1	0.99	0.78	
☑	F5	RGS1 7	Unknown	1	0.99	0.78	
☑	F6	RGS1 7	Unknown	1	0.99	0.79	
☑	F7	RGS1 11	Unknown	1	0.73	0.16	
☑	F8	RGS1 11	Unknown	1	0.87	0.50	
☑	F9	RGS1 11	Unknown	1	0.95	0.83	

Results

Inc	Pos.	Sample Name	Sample Type	Group	Score	Resolution	Status
☑	F10	RGS1 14	Unknown	1	0.99	0.81	
☑	F11	RGS1 14	Unknown	1	0.99	0.82	
☑	F12	RGS1 14	Unknown	1	0.99	0.80	
☑	G1		Unknown	Negative	1.00	1.00	
☑	G2		Unknown	Negative	1.00	1.00	
☑	G3		Unknown	Negative	1.00	1.00	
☑	G4		Unknown	Negative	1.00	1.00	
☑	G5		Unknown	Negative	1.00	1.00	
☑	G6		Unknown	Negative	1.00	1.00	
☑	G7		Unknown	Negative	1.00	1.00	
☑	G8		Unknown	Negative	1.00	1.00	
☑	G9		Unknown	Negative	1.00	1.00	
☑	G10		Unknown	Negative	1.00	1.00	
☑	G11		Unknown	Negative	1.00	1.00	
☑	G12		Unknown	Negative	1.00	1.00	
☑	H1		Unknown	Negative	1.00	1.00	
☑	H2		Unknown	Negative	1.00	1.00	
☑	H3		Unknown	Negative	1.00	1.00	
☑	H4		Unknown	Negative	1.00	1.00	
☑	H5		Unknown	Negative	1.00	1.00	
☑	H6		Unknown	Negative	1.00	1.00	
☑	H7		Unknown	Negative	1.00	1.00	
☑	H8		Unknown	Negative	1.00	1.00	
☑	H9		Unknown	Negative	1.00	1.00	
☑	H10		Unknown	Negative	1.00	1.00	
☑	H11		Unknown	Negative	1.00	1.00	
☑	H12		Unknown	Negative	1.00	1.00	

Group 1

Inc	Pos	Sample Name	Sample Type	Score	Resolution	Status
☑	A1	GAPDH 1	Unknown	0.98	0.95	
☑	A2	GAPDH 1	Unknown	0.96	0.94	
☑	A3	GAPDH 1	Unknown	0.98	0.96	
☑	A4	GAPDH 2	Unknown	0.98	0.96	
☑	A5	GAPDH 2	Unknown	0.97	0.95	
☑	A6	GAPDH 2	Unknown	0.98	0.96	
☑	A7	GAPDH 3	Unknown	0.99	0.95	
☑	A8	GAPDH 3	Unknown	0.99	0.94	

Group	1

Inc	Pos	Sample Name	Sample Type	Score	Resolution	Status
☑	A9	GAPDH 3	Unknown	0.96	0.93	
☑	A10	GAPDH 4	Unknown	0.95	0.92	
☑	A11	GAPDH 4	Unknown	0.97	0.91	
☑	A12	GAPDH 4	Unknown	0.98	0.94	
☑	C1	RGS1 1	Unknown	0.94	0.77	
☑	C2	RGS1 1	Unknown	0.92	0.58	
☑	C3	RGS1 1	Unknown	0.98	0.83	
☑	C4	RGS1 2	Unknown	0.96	0.83	
☑	C5	RGS1 2	Unknown	0.99	0.81	
☑	C6	RGS1 2	Unknown	0.97	0.83	
☑	C7	RGS1 3	Unknown	1.00	0.80	
☑	C8	RGS1 3	Unknown	0.99	0.79	
☑	C9	RGS1 3	Unknown	0.93	0.86	
☑	D1	GAPDH 5	Unknown	0.99	0.95	
☑	D2	GAPDH 5	Unknown	0.98	0.95	
☑	D3	GAPDH 5	Unknown	0.98	0.94	
☑	D4	GAPDH 7	Unknown	0.98	0.94	
☑	D5	GAPDH 7	Unknown	0.98	0.95	
☑	D6	GAPDH 7	Unknown	0.96	0.94	
☑	D7	GAPDH 11	Unknown	0.95	0.92	
☑	D8	GAPDH 11	Unknown	0.98	0.95	
☑	D9	GAPDH 11	Unknown	0.95	0.93	
☑	D10	GAPDH 14	Unknown	0.96	0.93	
☑	D11	GAPDH 14	Unknown	0.97	0.94	
☑	D12	GAPDH 14	Unknown	0.98	0.93	
☑	F1	RGS1 5	Unknown	0.98	0.75	
☑	F2	RGS1 5	Unknown	0.95	0.78	
☑	F3	RGS1 5	Unknown	0.99	0.78	
☑	F4	RGS1 7	Unknown	0.99	0.78	
☑	F5	RGS1 7	Unknown	0.99	0.78	
☑	F6	RGS1 7	Unknown	0.99	0.79	
☑	F7	RGS1 11	Unknown	0.73	0.16	
☑	F8	RGS1 11	Unknown	0.87	0.50	
☑	F9	RGS1 11	Unknown	0.95	0.83	
☑	F10	RGS1 14	Unknown	0.99	0.81	
☑	F11	RGS1 14	Unknown	0.99	0.82	
☑	F12	RGS1 14	Unknown	0.99	0.80	

Group	2

Inc	Pos	Sample Name	Sample Type	Score	Resolution	Status
☑	B1	FCER1A 1	Unknown	0.82	0.76	
☑	B2	FCER1A 1	Unknown	0.89	0.75	
☑	B3	FCER1A 1	Unknown	0.86	0.80	
☑	B4	FCER1A 2	Unknown	0.79	0.51	
☑	B5	FCER1A 2	Unknown	0.94	0.87	
☑	B6	FCER1A 2	Unknown	0.86	0.64	
☑	B7	FCER1A 3	Unknown	0.81	0.75	
☑	B8	FCER1A 3	Unknown	0.85	0.78	
☑	B9	FCER1A 3	Unknown	0.74	0.70	
☑	B10	FCER1A 4	Unknown	0.96	0.87	
☑	B11	FCER1A 4	Unknown	0.95	0.86	
☑	B12	FCER1A 4	Unknown	0.96	0.85	
☑	C10	RGS1 4	Unknown	0.97	0.73	
☑	C12	RGS1 4	Unknown	1.00	0.80	
☑	E1	FCER1A 5	Unknown	0.78	0.54	
☑	E2	FCER1A 5	Unknown	0.76	0.50	
☑	E3	FCER1A 5	Unknown	0.84	0.62	
☑	E4	FCER1A 7	Unknown	0.94	0.81	
☑	E5	FCER1A 7	Unknown	0.91	0.69	
☑	E6	FCER1A 7	Unknown	0.92	0.83	
☑	E7	FCER1A 11	Unknown	0.93	0.73	
☑	E8	FCER1A 11	Unknown	0.97	0.85	
☑	E9	FCER1A 11	Unknown	0.97	0.86	
☑	E10	FCER1A 14	Unknown	0.93	0.78	
☑	E11	FCER1A 14	Unknown	0.92	0.78	
☑	E12	FCER1A 14	Unknown	0.92	0.81	

Group	3

Inc	Pos	Sample Name	Sample Type	Score	Resolution	Status
☑	C11	RGS1 4	Unknown	1.00	0.99	

Group	Negative

Inc	Pos	Sample Name	Sample Type	Score	Resolution	Status
☑	G1		Unknown	1.00	1.00	
☑	G2		Unknown	1.00	1.00	
☑	G3		Unknown	1.00	1.00	
☑	G4		Unknown	1.00	1.00	
☑	G5		Unknown	1.00	1.00	

**Group**	Negative

Inc	Pos	Sample Name	Sample Type	Score	Resolution	Status
☑	G6		Unknown	1.00	1.00	
☑	G7		Unknown	1.00	1.00	
☑	G8		Unknown	1.00	1.00	
☑	G9		Unknown	1.00	1.00	
☑	G10		Unknown	1.00	1.00	
☑	G11		Unknown	1.00	1.00	
☑	G12		Unknown	1.00	1.00	
☑	H1		Unknown	1.00	1.00	
☑	H2		Unknown	1.00	1.00	
☑	H3		Unknown	1.00	1.00	
☑	H4		Unknown	1.00	1.00	
☑	H5		Unknown	1.00	1.00	
☑	H6		Unknown	1.00	1.00	
☑	H7		Unknown	1.00	1.00	
☑	H8		Unknown	1.00	1.00	
☑	H9		Unknown	1.00	1.00	
☑	H10		Unknown	1.00	1.00	
☑	H11		Unknown	1.00	1.00	
☑	H12		Unknown	1.00	1.00	

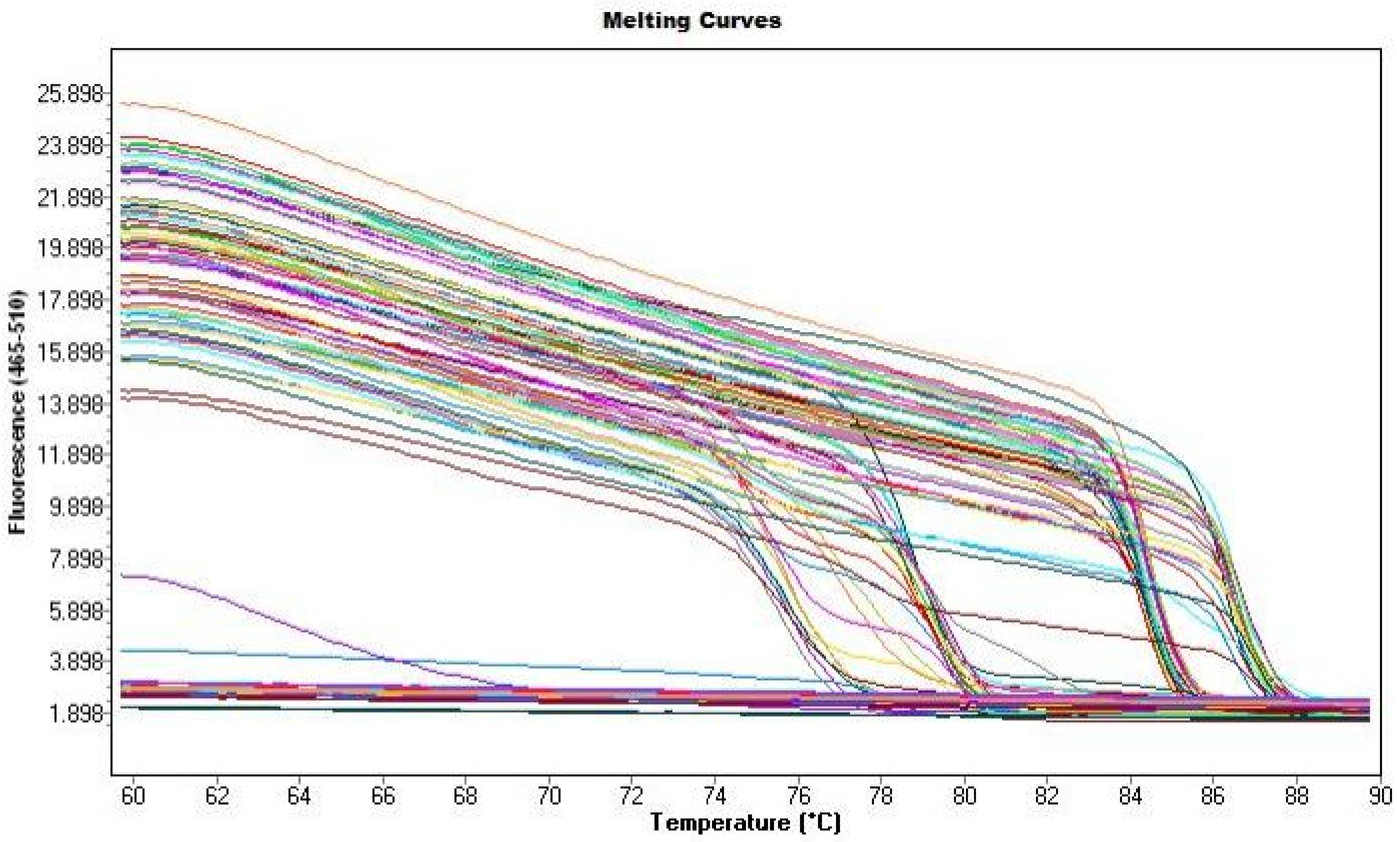

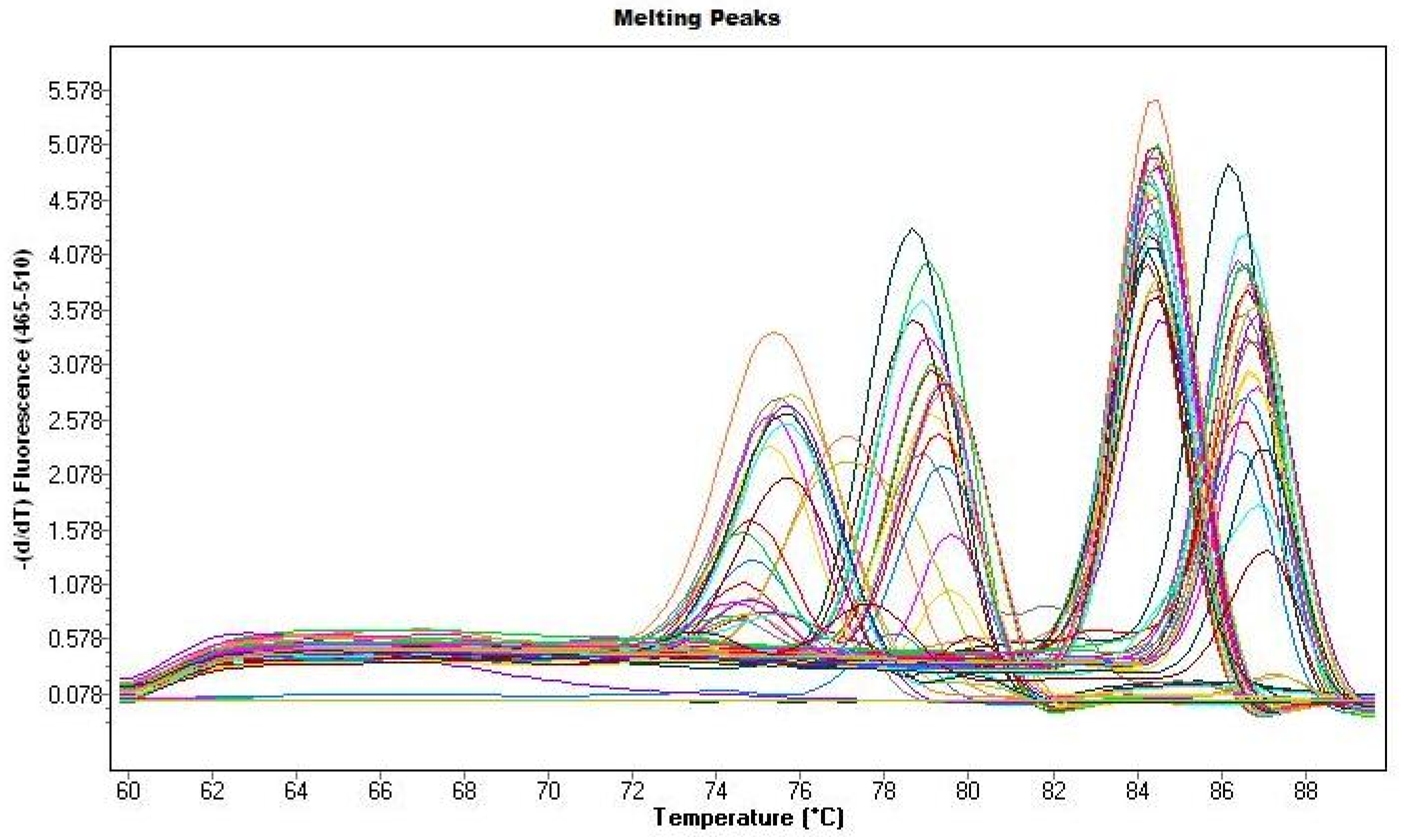

Analysis Notes	

Tm Calling for All Samples (Tm Calling)

Settings

Channel	465-510	Color Compensation	Off		
Program	Program				
Show Shoulders	False	Chemistry	HybProbe	Max Peaks	2

Subset Name	All Samples

Results

Inc	Pos	Sample Name	Peak 1 Tm	Peak 1 Area	Peak 1 Width	Peak 1 Height	Peak 2 Tm	Peak 2 Area	Peak 2 Width	Peak 2 Height	Status
☑	A1	GAPDH 1	84.48	10.91	2.32	4.71					
☑	A2	GAPDH 1	84.31	9.01	2.16	4.16					
☑	A3	GAPDH 1	84.38	11.55	2.33	4.96					
☑	A4	GAPDH 2	84.47	10.42	2.18	4.78					
☑	A5	GAPDH 2	84.27	11.30	2.34	4.84					
☑	A6	GAPDH 2	84.33	10.51	2.33	4.51					
☑	A7	GAPDH 3	84.41	9.85	2.24	4.39					
☑	A8	GAPDH 3	84.55	9.89	2.22	4.46					
☑	A9	GAPDH 3	84.48	9.49	2.24	4.23					
☑	A10	GAPDH 4	84.49	9.19	2.31	3.98					
☑	A11	GAPDH 4	84.64	8.22	2.25	3.66					
☑	A12	GAPDH 4	84.56	9.50	2.33	4.08					
☑	B1	FCER1A 1	78.23	1.37	2.10	0.65					
☑	B2	FCER1A 1	74.57	1.57	2.03	0.77	79.20	7.74	2.47	3.14	
☑	B3	FCER1A 1	79.05	10.67	2.57	4.15					
☑	B4	FCER1A 2	75.38	6.58	2.45	2.68	79.72	3.97	2.32	1.71	
☑	B5	FCER1A 2	75.59	8.80	3.10	2.84					
☑	B6	FCER1A 2	75.34	6.02	2.52	2.39	79.67	2.57	2.28	1.13	
☑	B7	FCER1A 3	78.70	8.66	2.47	3.50					
☑	B8	FCER1A 3	78.94	9.52	2.53	3.76					
☑	B9	FCER1A 3	78.67	10.47	2.42	4.32					
☑	B10	FCER1A 4	75.60	11.12	3.27	3.40					
☑	B11	FCER1A 4	75.85	8.41	3.04	2.77					
☑	B12	FCER1A 4	75.83	9.82	3.48	2.82					
☑	C1	RGS1 1	86.64	3.83	1.73	2.22					
☑	C2	RGS1 1	75.41	1.10	2.68	0.41	86.61	7.83	2.85	2.75	
☑	C3	RGS1 1	86.64	9.94	2.38	4.18					
☑	C4	RGS1 2									
☑	C5	RGS1 2									

Results

Inc	Pos	Sample Name	Peak 1 Tm	Area	Width	Height	Peak 2 Tm	Area	Width	Height	Status
☑	C6	RGS1 2									
☑	C7	RGS1 3									
☑	C8	RGS1 3	86.64	9.53	2.20	4.33					
☑	C9	RGS1 3	86.27	12.38	2.39	5.19					
☑	C10	RGS1 4	77.14	8.09	3.34	2.42					
☑	C11	RGS1 4									
☑	C12	RGS1 4	77.41	9.01	3.96	2.28					
☑	D1	GAPDH 5	84.40	10.31	2.28	4.53					
☑	D2	GAPDH 5	84.50	12.09	2.32	5.22					
☑	D3	GAPDH 5	84.60	11.85	2.21	5.36					
☑	D4	GAPDH 7	84.47	11.57	2.27	5.10					
☑	D5	GAPDH 7	84.38	10.58	2.14	4.94					
☑	D6	GAPDH 7	84.43	10.80	2.30	4.69					
☑	D7	GAPDH 11	84.49	8.93	2.30	3.89					
☑	D8	GAPDH 11	84.38	11.72	2.36	4.97					
☑	D9	GAPDH 11	84.30	9.34	2.27	4.12					
☑	D10	GAPDH 14	84.50	12.93	2.24	5.76					
☑	D11	GAPDH 14	84.56	11.60	2.27	5.11					
☑	D12	GAPDH 14	84.64	11.92	2.31	5.15					
☑	E1	FCER1A 5	74.87	2.25	2.08	1.08	79.45	5.67	2.53	2.24	
☑	E2	FCER1A 5	74.86	2.99	2.13	1.40	79.36	6.41	2.53	2.54	
☑	E3	FCER1A 5	74.62	2.46	1.99	1.23	79.21	8.07	2.54	3.18	
☑	E4	FCER1A 7	74.28	0.93	1.80	0.52	79.07	8.75	2.61	3.36	
☑	E5	FCER1A 7	78.96	5.67	2.58	2.20	82.16	2.04	2.49	0.82	
☑	E6	FCER1A 7	79.19	7.03	2.65	2.65					
☑	E7	FCER1A 11	75.72	6.86	3.55	1.93					
☑	E8	FCER1A 11	75.75	7.11	2.91	2.44					
☑	E9	FCER1A 11	75.72	7.59	3.00	2.53					
☑	E10	FCER1A 14	74.72	1.04	1.73	0.60	79.55	7.72	2.64	2.92	
☑	E11	FCER1A 14	74.75	0.97	1.65	0.58	79.53	7.68	2.56	3.00	
☑	E12	FCER1A 14	74.67	0.79	1.65	0.48	79.49	7.89	2.60	3.03	
☑	F1	RGS1 5									
☑	F2	RGS1 5									
☑	F3	RGS1 5									
☑	F4	RGS1 7	86.54	9.56	2.30	4.15					
☑	F5	RGS1 7	86.65	8.54	2.36	3.62					

Results

Inc	Pos	Sample Name	Peak 1 Tm	Peak 1 Area	Peak 1 Width	Peak 1 Height	Peak 2 Tm	Peak 2 Area	Peak 2 Width	Peak 2 Height	Status
☑	F6	RGS1 7									
☑	F7	RGS1 11	76.90	2.26	4.71	0.48					
☑	F8	RGS1 11	74.99	1.06	2.23	0.47					
☑	F9	RGS1 11									
☑	F10	RGS1 14									
☑	F11	RGS1 14									
☑	F12	RGS1 14									
☑	G1										
☑	G2										
☑	G3										
☑	G4										
☑	G5										
☑	G6										
☑	G7										
☑	G8										
☑	G9										
☑	G10										
☑	G11										
☑	G12										
☑	H1										
☑	H2										
☑	H3										
☑	H4										
☑	H5										
☑	H6										
☑	H7										
☑	H8										
☑	H9										
☑	H10										
☑	H11										
☑	H12										

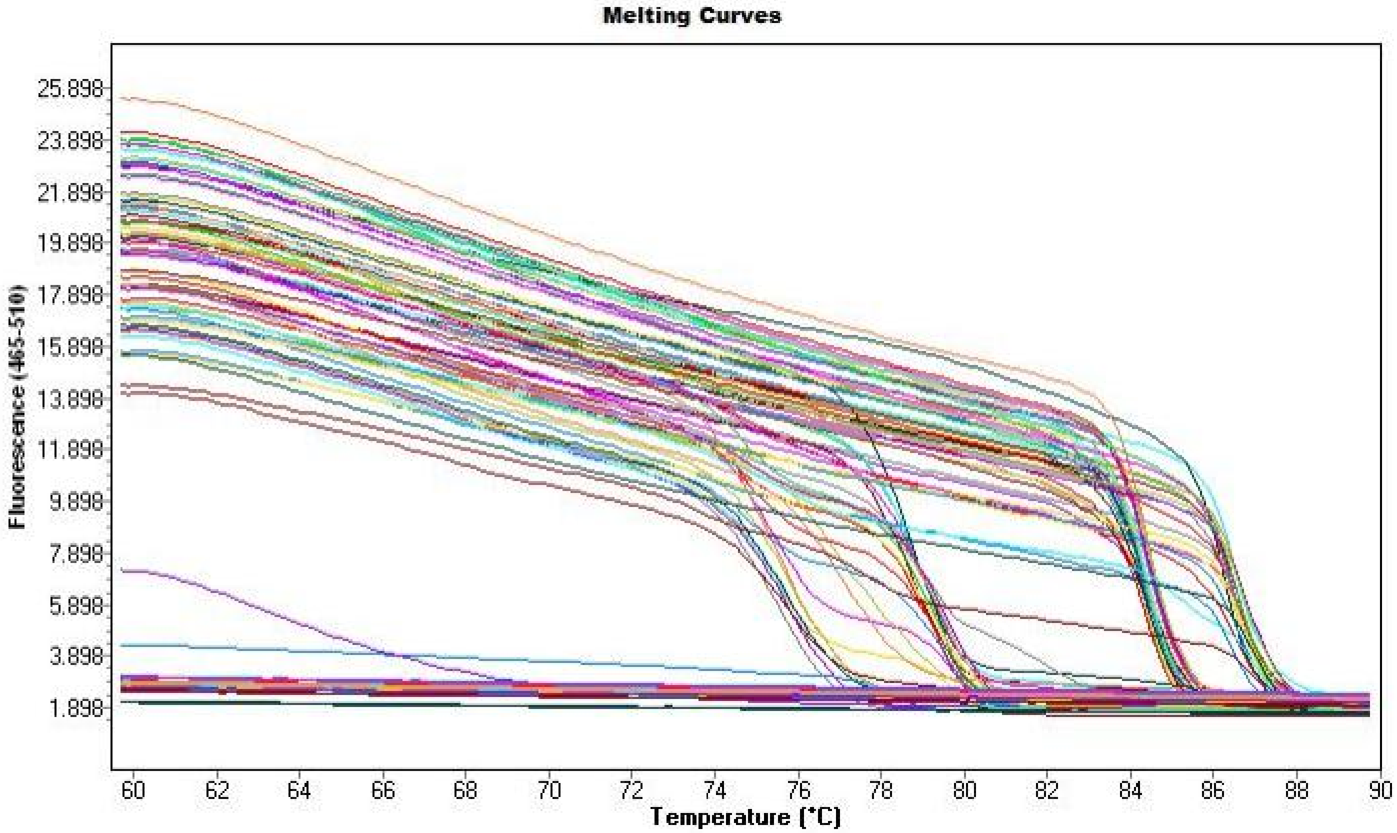

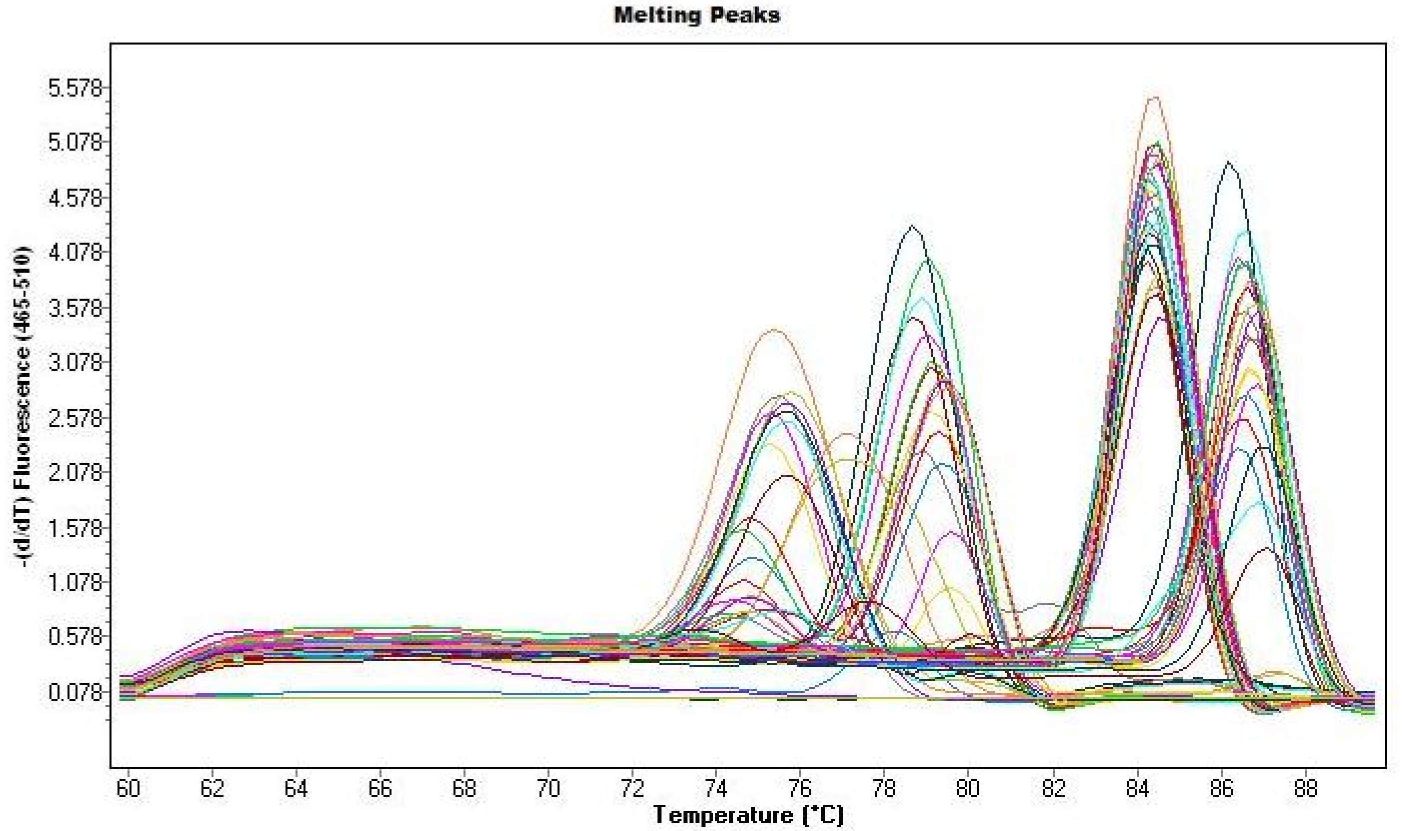

Analysis Notes	
