# Supplementary material for: Exploring differential gene expression and biomarker potential in systemic lupus erythematosus: a retrospective study
Source: PeerJ. 2025 Sep 8;13:e19891. doi: 10.7717/peerj.19891 (PMC12424612; doi:10.7717/peerj.19891)
Supplement: Supplemental Information 1 — The data include the Ct value for each sample, the Ct value for reference genes such as GAPDH, and the relative expression levels calculated using the ΔΔCt method. These data were used to validate the RNA sequencing results and further evaluate the potential of these genes as biomarkers for SLE. [file peerj-13-19891-s001.zip › qPCR/20241212 qPCR/1212plate2 SLE group.pdf]

cobas® 4800 User Defined Workflow (UDF)

20241213xj plate2

Experiment

Creation Date	12/13/2024 9:04:56 AM	Last Modified Date	12/13/2024 10:12:02 AM
Operator	System Admin	Owner	System Admin
Start Time	12/13/2024 9:10:30 AM	End Time	12/13/2024 10:09:29 AM
Run State	Completed	Software Version	LCS480 1.5.1.62 SP3 - UDF 2.1.0.26
Macro		Macro Owner	
Macro Status			
Templates	gongying	Plate ID	
Test ID		Lot ID	
Color Comp ID			
Run Notes			

Programs

Program Name	Program		
Cycles	1	Analysis Mode	None

Target (°C)	Acquisition Mode	Hold (hh:mm:ss)	Ramp Rate (°C/s)	Acquisitions (per °C)	Sec Target (°C)	Step size (°C)	Step Delay (cycles)
95	None	00:00:30	4.40		0	0	0

Program Name	Program		
Cycles	40	Analysis Mode	Quantification

Target (°C)	Acquisition Mode	Hold (hh:mm:ss)	Ramp Rate (°C/s)	Acquisitions (per °C)	Sec Target (°C)	Step size (°C)	Step Delay (cycles)
95	None	00:00:15	4.40		0	0	0
60	Single	00:00:30	2.20		0	0	0

Program Name	Program		
Cycles	1	Analysis Mode	Melting Curves

Target (°C)	Acquisition Mode	Hold (hh:mm:ss)	Ramp Rate (°C/s)	Acquisitions (per °C)	Sec Target (°C)	Step size (°C)	Step Delay (cycles)
95	None	00:01:00	4.40		0	0	0
60	None	00:00:30	2.20		0	0	0
90	Continuous		0.11	5	0	0	0

Program Name	Program		
Cycles	1	Analysis Mode	None

Target (°C)	Acquisition Mode	Hold (hh:mm:ss)	Ramp Rate (°C/s)	Acquisitions (per °C)	Sec Target (°C)	Step size (°C)	Step Delay (cycles)

Target (°C)	Acquisition Mode	Hold (hh:mm:ss)	Ramp Rate (°C/s)	Acquisitions (per °C)	Sec Target (°C)	Step size (°C)	Step Delay (cycles)
40	None	00:00:30	2.20		0	0	0

Samples

Sample Count	96
Subset	All Samples

Pos	Name	ID	Repl. Of	Sample Notes
A1	GAPDH 15			
A2	GAPDH 15			
A3	GAPDH 15			
A4	GAPDH 18			
A5	GAPDH 18			
A6	GAPDH 18			
A7	GAPDH 19			
A8	GAPDH 19			
A9	GAPDH 19			
A10	GAPDH 20			
A11	GAPDH 20			
A12	GAPDH 20			
B1	FCER1A 15			
B2	FCER1A 15			
B3	FCER1A 15			
B4	FCER1A 18			
B5	FCER1A 18			
B6	FCER1A 18			
B7	FCER1A 19			
B8	FCER1A 19			
B9	FCER1A 19			
B10	FCER1A 20			
B11	FCER1A 20			
B12	FCER1A 20			
C1	RGS1 15			
C2	RGS1 15			
C3	RGS1 15			
C4	RGS1 18			
C5	RGS1 18			
C6	RGS1 18			
C7	RGS1 19			
C8	RGS1 19			
C9	RGS1 19			
C10	RGS1 20			
C11	RGS1 20			
C12	RGS1 20			

Samples

Sample Count	96
Subset	All Samples

Pos	Name	ID	Repl. Of	Sample Notes
D1	GAPDH 24			
D2	GAPDH 24			
D3	GAPDH 24			
D4	GAPDH 26			
D5	GAPDH 26			
D6	GAPDH 26			
D7				
D8				
D9				
D10				
D11				
D12				
E1	FCER1A 24			
E2	FCER1A 24			
E3	FCER1A 24			
E4	FCER1A 26			
E5	FCER1A 26			
E6	FCER1A 26			
E7				
E8				
E9				
E10				
E11				
E12				
F1	RGS1 24			
F2	RGS1 24			
F3	RGS1 24			
F4	RGS1 26			
F5	RGS1 26			
F6	RGS1 26			
F7				
F8				
F9				
F10				
F11				
F12				
G1				
G2				
G3				

Samples

Sample Count	96
Subset	All Samples

Pos	Name	ID	Repl. Of	Sample Notes
G4				
G5				
G6				
G7				
G8				
G9				
G10				
G11				
G12				
H1				
H2				
H3				
H4				
H5				
H6				
H7				
H8				
H9				
H10				
H11				
H12				

Instrument

Name	Z480	Serial Number	56319

Revision History

Revision	Date	User	Reason

Abs Quant/2nd Derivative Max for All Samples (Abs Quant/2nd Derivative Max)

Settings

Channel	465-510		
Color Compensation	Off		
Program	Program	Units	
Mode	High Confidence		

Subset Name	All Samples

Results

> -Inate	CPposal	N(lasmteive cycles) has higher uncertainty	Type	CP	Concentration	Standard	Status

Results

Inc	Pos	Name	Type	CP	Concentration	Standard	Status
☑	A1	GAPDH 15	Unknown				
☑	A2	GAPDH 15	Unknown				
☑	A3	GAPDH 15	Unknown				
☑	A4	GAPDH 18	Unknown				
☑	A5	GAPDH 18	Unknown				
☑	A6	GAPDH 18	Unknown				
☑	A7	GAPDH 19	Unknown				
☑	A8	GAPDH 19	Unknown				
☑	A9	GAPDH 19	Unknown				
☑	A10	GAPDH 20	Unknown				
☑	A11	GAPDH 20	Unknown				
☑	A12	GAPDH 20	Unknown				
☑	B1	FCER1A 15	Unknown				
☑	B2	FCER1A 15	Unknown				
☑	B3	FCER1A 15	Unknown				
☑	B4	FCER1A 18	Unknown				
☑	B5	FCER1A 18	Unknown				
☑	B6	FCER1A 18	Unknown				
☑	B7	FCER1A 19	Unknown				
☑	B8	FCER1A 19	Unknown				
☑	B9	FCER1A 19	Unknown				
☑	B10	FCER1A 20	Unknown				
☑	B11	FCER1A 20	Unknown				
☑	B12	FCER1A 20	Unknown				
☑	C1	RGS1 15	Unknown	30.04			
☑	C2	RGS1 15	Unknown	28.89			
☑	C3	RGS1 15	Unknown	27.88			
☑	C4	RGS1 18	Unknown	29.96			
☑	C5	RGS1 18	Unknown	29.19			
☑	C6	RGS1 18	Unknown	29.97			
☑	C7	RGS1 19	Unknown	29.85			
☑	C8	RGS1 19	Unknown	35.00			>
☑	C9	RGS1 19	Unknown	29.18			
☑	C10	RGS1 20	Unknown	30.74			
☑	C11	RGS1 20	Unknown	30.46			
☑	C12	RGS1 20	Unknown	30.89			
☑	D1	GAPDH 24	Unknown	30.16			

> - Late Cp call (last five cycles) has higher uncertainty

Results

Inc	Pos	Name	Type	CP	Concentration	Standard	Status
☑	D2	GAPDH 24	Unknown	30.15			
☑	D3	GAPDH 24	Unknown	31.59			
☑	D4	GAPDH 26	Unknown	31.08			
☑	D5	GAPDH 26	Unknown	30.78			
☑	D6	GAPDH 26	Unknown	30.94			
☑	D7		Unknown				
☑	D8		Unknown	35.00			>
☑	D9		Unknown	35.00			>
☑	D10		Unknown	35.00			>
☑	D11		Unknown				
☑	D12		Unknown				
☑	E1	FCER1A 24	Unknown	22.46			
☑	E2	FCER1A 24	Unknown	22.30			
☑	E3	FCER1A 24	Unknown	21.80			
☑	E4	FCER1A 26	Unknown	21.97			
☑	E5	FCER1A 26	Unknown	22.05			
☑	E6	FCER1A 26	Unknown	22.02			
☑	E7		Unknown	35.00			>
☑	E8		Unknown				
☑	E9		Unknown	35.00			>
☑	E10		Unknown				
☑	E11		Unknown				
☑	E12		Unknown	35.00			>
☑	F1	RGS1 24	Unknown	28.30			
☑	F2	RGS1 24	Unknown	28.63			
☑	F3	RGS1 24	Unknown	28.54			
☑	F4	RGS1 26	Unknown	27.61			
☑	F5	RGS1 26	Unknown	27.44			
☑	F6	RGS1 26	Unknown	27.53			
☑	F7		Unknown	35.00			>
☑	F8		Unknown				
☑	F9		Unknown	35.00			>
☑	F10		Unknown	35.00			>
☑	F11		Unknown	35.00			>
☑	F12		Unknown	35.00			>
☑	G1		Unknown	28.17			
☑	G2		Unknown	27.16			

> - Late Cp call (last five cycles) has higher uncertainty

Results

Inc	Pos	Name	Type	CP	Concentration	Standard	Status
☑	G3		Unknown	28.20			
☑	G4		Unknown	27.05			
☑	G5		Unknown	26.71			
☑	G6		Unknown	26.94			
☑	G7		Unknown	26.70			
☑	G8		Unknown	26.74			
☑	G9		Unknown	26.89			
☑	G10		Unknown	27.15			
☑	G11		Unknown	27.65			
☑	G12		Unknown	27.23			
☑	H1		Unknown	21.29			
☑	H2		Unknown	21.25			
☑	H3		Unknown	21.45			
☑	H4		Unknown	21.80			
☑	H5		Unknown	21.64			
☑	H6		Unknown	21.88			
☑	H7		Unknown	21.57			
☑	H8		Unknown	21.51			
☑	H9		Unknown	21.51			
☑	H10		Unknown	21.88			
☑	H11		Unknown	21.81			
☑	H12		Unknown	21.81			

> - Late Cp call (last five cycles) has higher uncertainty

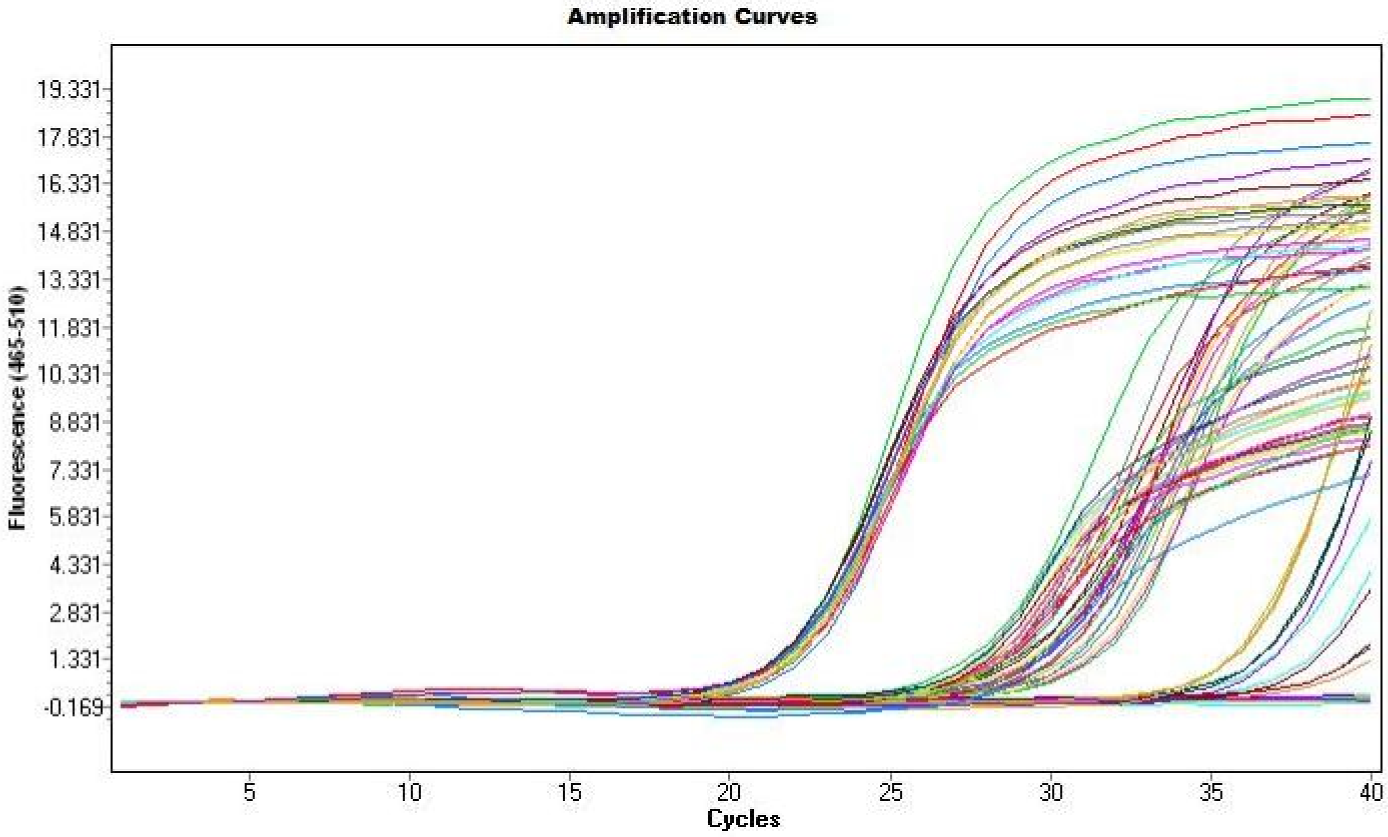

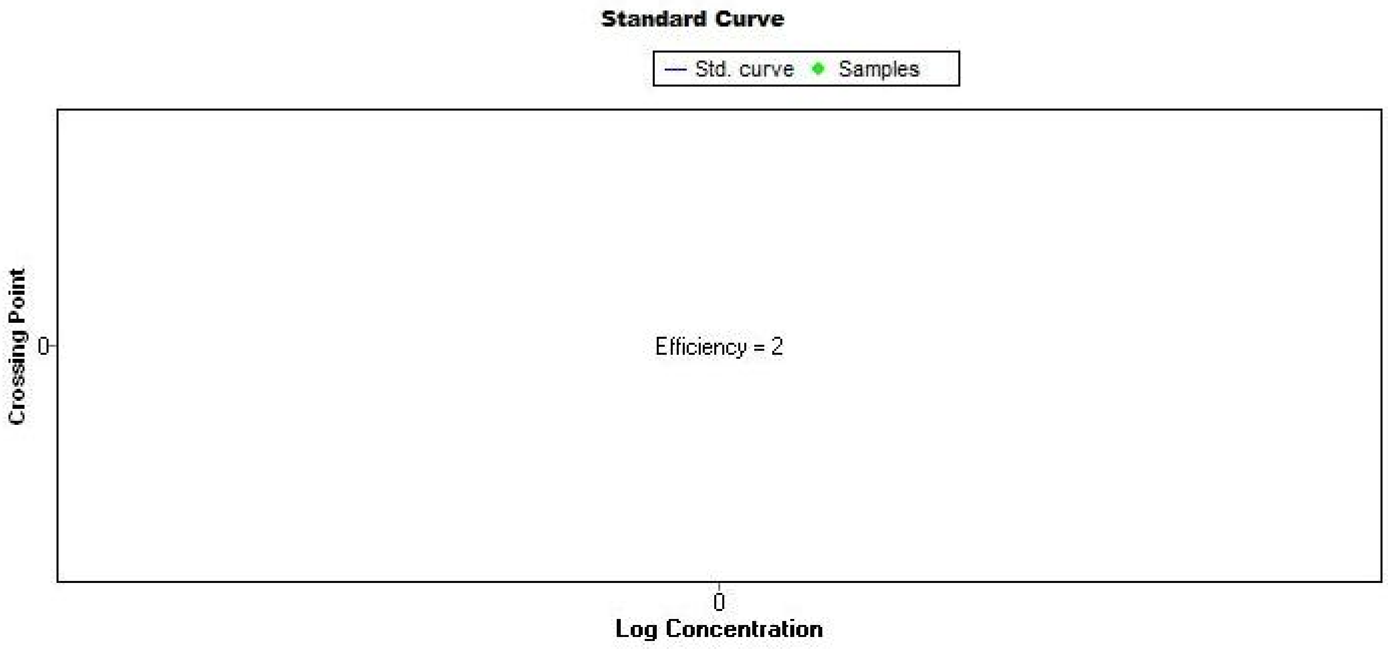

Analysis Notes	

Abs Quant/Fit Points for All Samples (Abs Quant/Fit Points)

Settings

Channel	465-510				
Color Compensation	Off				
Standard Curve	Set Efficiency (2.00)				
Program	Program			Units	
First Cycle	1	Last Cycle	40	Background	2-6
Noiseband Method	Auto	Noiseband	0.8427	STD Dev Multiplier	12.0000
# of Fit Points	2	Threshold Method	Auto	Threshold	0.8427

Subset Name	All Samples

Results

Inc	Pos	Name	Type	CP	Concentration	Standard	Status
☑	A1	GAPDH 15	Unknown				
☑	A2	GAPDH 15	Unknown				
☑	A3	GAPDH 15	Unknown				
☑	A4	GAPDH 18	Unknown				
☑	A5	GAPDH 18	Unknown				
☑	A6	GAPDH 18	Unknown				
☑	A7	GAPDH 19	Unknown				
☑	A8	GAPDH 19	Unknown				
☑	A9	GAPDH 19	Unknown				
☑	A10	GAPDH 20	Unknown				
☑	A11	GAPDH 20	Unknown				
☑	A12	GAPDH 20	Unknown				
☑	B1	FCER1A 15	Unknown				
☑	B2	FCER1A 15	Unknown				
☑	B3	FCER1A 15	Unknown				
☑	B4	FCER1A 18	Unknown				
☑	B5	FCER1A 18	Unknown				
☑	B6	FCER1A 18	Unknown				
☑	B7	FCER1A 19	Unknown				
☑	B8	FCER1A 19	Unknown				
☑	B9	FCER1A 19	Unknown				
☑	B10	FCER1A 20	Unknown				
☑	B11	FCER1A 20	Unknown				
☑	B12	FCER1A 20	Unknown				
☑	C1	RGS1 15	Unknown	26.86			
☑	C2	RGS1 15	Unknown	24.87			
☑	C3	RGS1 15	Unknown	24.67			

Results

Inc	Pos	Name	Type	CP	Concentration	Standard	Status
☑	C4	RGS1 18	Unknown	26.12			
☑	C5	RGS1 18	Unknown	25.25			
☑	C6	RGS1 18	Unknown	26.15			
☑	C7	RGS1 19	Unknown	25.61			
☑	C8	RGS1 19	Unknown	35.51			
☑	C9	RGS1 19	Unknown	24.32			
☑	C10	RGS1 20	Unknown	26.97			
☑	C11	RGS1 20	Unknown	26.79			
☑	C12	RGS1 20	Unknown	27.90			
☑	D1	GAPDH 24	Unknown	28.57			
☑	D2	GAPDH 24	Unknown	27.64			
☑	D3	GAPDH 24	Unknown	28.72			
☑	D4	GAPDH 26	Unknown	28.51			
☑	D5	GAPDH 26	Unknown	27.49			
☑	D6	GAPDH 26	Unknown	28.33			
☑	D7		Unknown				
☑	D8		Unknown	33.84			
☑	D9		Unknown	35.93			
☑	D10		Unknown	36.99			
☑	D11		Unknown				
☑	D12		Unknown				
☑	E1	FCER1A 24	Unknown	20.26			
☑	E2	FCER1A 24	Unknown	19.68			
☑	E3	FCER1A 24	Unknown	18.85			
☑	E4	FCER1A 26	Unknown	19.48			
☑	E5	FCER1A 26	Unknown	19.33			
☑	E6	FCER1A 26	Unknown	19.35			
☑	E7		Unknown	36.10			
☑	E8		Unknown				
☑	E9		Unknown	33.50			
☑	E10		Unknown				
☑	E11		Unknown				
☑	E12		Unknown	32.99			
☑	F1	RGS1 24	Unknown	27.89			
☑	F2	RGS1 24	Unknown	27.47			
☑	F3	RGS1 24	Unknown	26.29			
☑	F4	RGS1 26	Unknown	25.97			

Results

Inc	Pos	Name	Type	CP	Concentration	Standard	Status
☑	F5	RGS1 26	Unknown	25.96			
☑	F6	RGS1 26	Unknown	25.94			
☑	F7		Unknown	36.31			
☑	F8		Unknown				
☑	F9		Unknown	34.40			
☑	F10		Unknown	33.30			
☑	F11		Unknown	34.86			
☑	F12		Unknown	33.82			
☑	G1		Unknown	28.35			
☑	G2		Unknown	25.96			
☑	G3		Unknown	26.65			
☑	G4		Unknown	25.80			
☑	G5		Unknown	24.91			
☑	G6		Unknown	25.72			
☑	G7		Unknown	25.92			
☑	G8		Unknown	25.61			
☑	G9		Unknown	25.36			
☑	G10		Unknown	25.81			
☑	G11		Unknown	26.27			
☑	G12		Unknown	26.28			
☑	H1		Unknown	19.18			
☑	H2		Unknown	8.60			
☑	H3		Unknown	19.60			
☑	H4		Unknown	19.72			
☑	H5		Unknown	18.91			
☑	H6		Unknown	19.44			
☑	H7		Unknown	18.70			
☑	H8		Unknown	18.96			
☑	H9		Unknown	18.89			
☑	H10		Unknown	19.60			
☑	H11		Unknown	16.52			
☑	H12		Unknown	20.23			

Amplification Curves

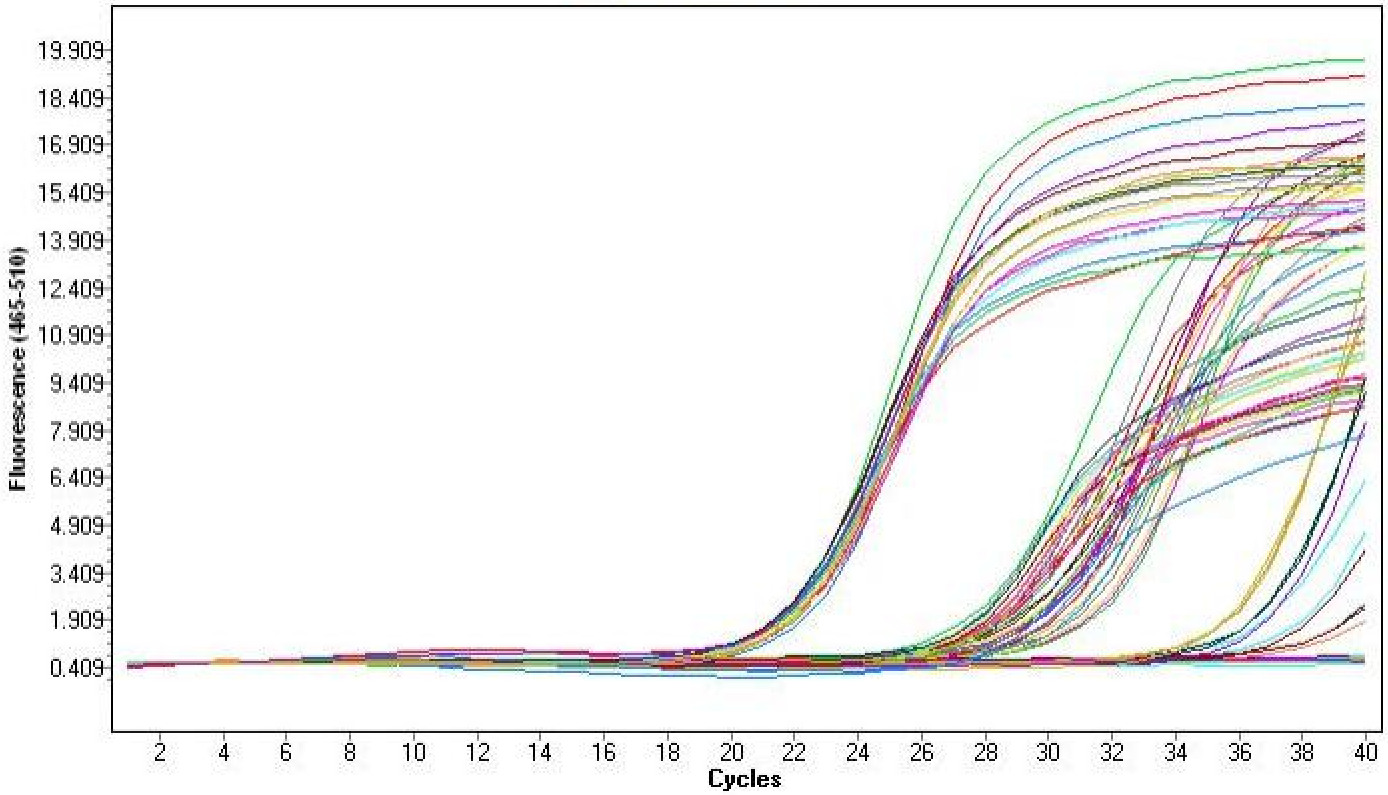

Amplification Curves

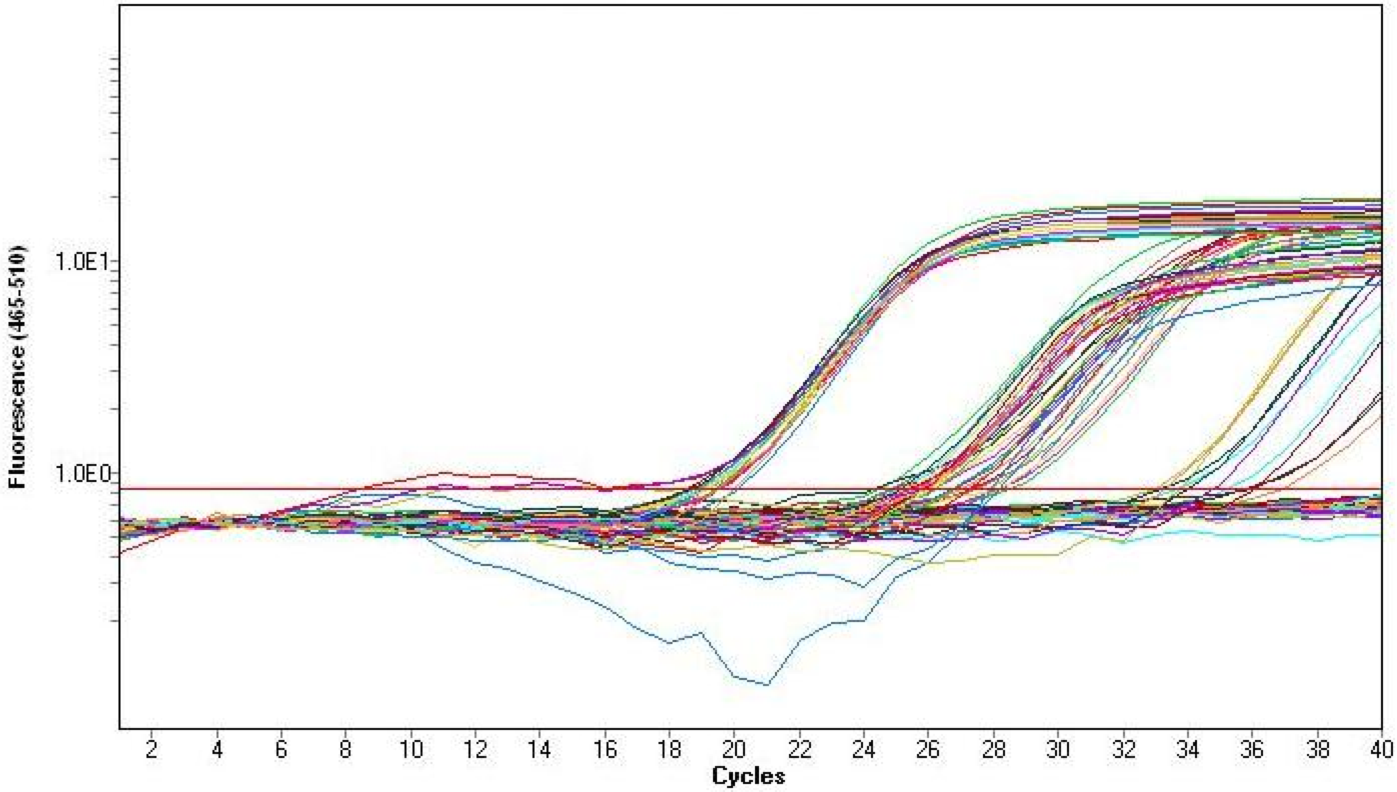

Amplification Curves

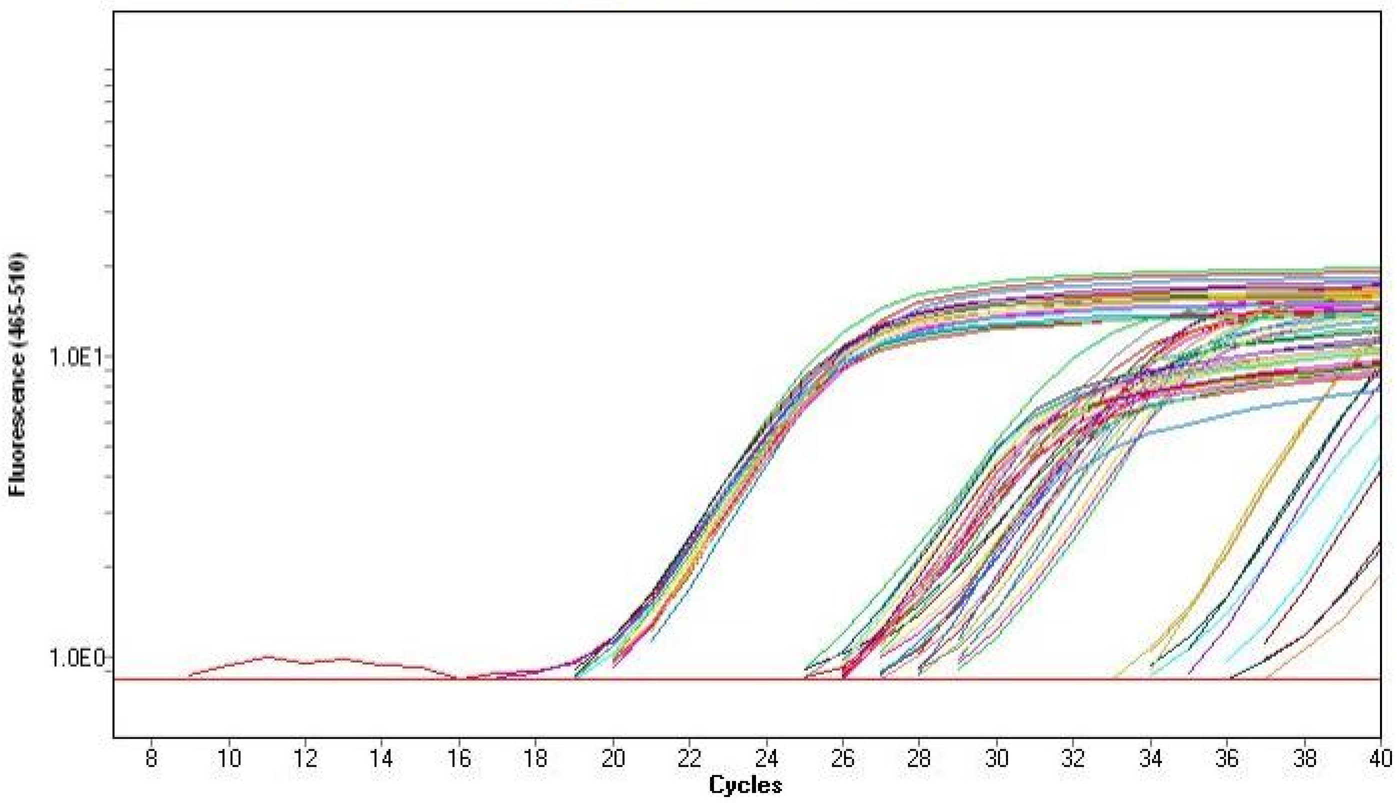

Standard Curve

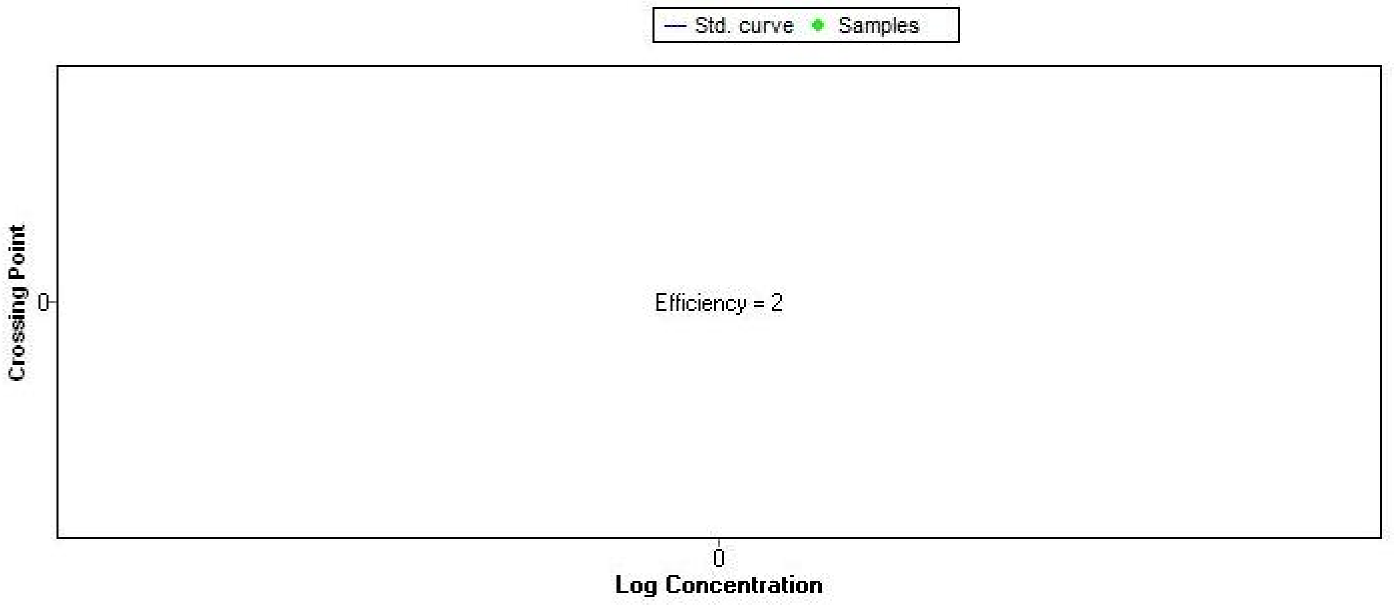

Analysis Notes	

Gene Scanning for All Samples (Gene Scanning)

Channel	465-510	Color Compensation	Off
Progam Name	Program		
Stds Settings	Auto-Group		
Sensitivity	0.30	Threshold (TmShift)	5.000
Pre-melt	69.75-70.74	Post-melt Temp	88.22-89.21

Subset Name	All Samples

Results

Inc	Pos.	Sample Name	Sample Type	Group	Status
☑	A1	GAPDH 15	Unknown	Negative	
☑	A2	GAPDH 15	Unknown	Negative	
☑	A3	GAPDH 15	Unknown	Negative	
☑	A4	GAPDH 18	Unknown	Negative	
☑	A5	GAPDH 18	Unknown	Negative	
☑	A6	GAPDH 18	Unknown	Negative	
☑	A7	GAPDH 19	Unknown	Negative	
☑	A8	GAPDH 19	Unknown	Negative	
☑	A9	GAPDH 19	Unknown	Negative	
☑	A10	GAPDH 20	Unknown	Negative	
☑	A11	GAPDH 20	Unknown	Negative	
☑	A12	GAPDH 20	Unknown	Negative	
☑	B1	FCER1A 15	Unknown	Negative	
☑	B2	FCER1A 15	Unknown	Negative	
☑	B3	FCER1A 15	Unknown	Negative	
☑	B4	FCER1A 18	Unknown	Negative	
☑	B5	FCER1A 18	Unknown	Negative	
☑	B6	FCER1A 18	Unknown	Negative	
☑	B7	FCER1A 19	Unknown	Negative	
☑	B8	FCER1A 19	Unknown	Negative	
☑	B9	FCER1A 19	Unknown	Negative	
☑	B10	FCER1A 20	Unknown	Negative	
☑	B11	FCER1A 20	Unknown	Negative	
☑	B12	FCER1A 20	Unknown	Negative	
☑	C1	RGS1 15	Unknown	1	
☑	C2	RGS1 15	Unknown	Unknown	
☑	C3	RGS1 15	Unknown	1	
☑	C4	RGS1 18	Unknown	1	
☑	C5	RGS1 18	Unknown	Unknown	
☑	C6	RGS1 18	Unknown	4	
☑	C7	RGS1 19	Unknown	1	

Results

Inc	Pos.	Sample Name	Sample Type	Group	Status
☑	C8	RGS1 19	Unknown	Unknown	
☑	C9	RGS1 19	Unknown	4	
☑	C10	RGS1 20	Unknown	4	
☑	C11	RGS1 20	Unknown	Unknown	
☑	C12	RGS1 20	Unknown	1	
☑	D1	GAPDH 24	Unknown	3	
☑	D2	GAPDH 24	Unknown	3	
☑	D3	GAPDH 24	Unknown	3	
☑	D4	GAPDH 26	Unknown	3	
☑	D5	GAPDH 26	Unknown	3	
☑	D6	GAPDH 26	Unknown	3	
☑	D7		Unknown	Negative	
☑	D8		Unknown	Unknown	
☑	D9		Unknown	Negative	
☑	D10		Unknown	Negative	
☑	D11		Unknown	Negative	
☑	D12		Unknown	Negative	
☑	E1	FCER1A 24	Unknown	1	
☑	E2	FCER1A 24	Unknown	1	
☑	E3	FCER1A 24	Unknown	1	
☑	E4	FCER1A 26	Unknown	1	
☑	E5	FCER1A 26	Unknown	1	
☑	E6	FCER1A 26	Unknown	1	
☑	E7		Unknown	Negative	
☑	E8		Unknown	Negative	
☑	E9		Unknown	Unknown	
☑	E10		Unknown	Negative	
☑	E11		Unknown	Negative	
☑	E12		Unknown	Unknown	
☑	F1	RGS1 24	Unknown	1	
☑	F2	RGS1 24	Unknown	1	
☑	F3	RGS1 24	Unknown	1	
☑	F4	RGS1 26	Unknown	1	
☑	F5	RGS1 26	Unknown	4	
☑	F6	RGS1 26	Unknown	4	
☑	F7		Unknown	2	
☑	F8		Unknown	Negative	
☑	F9		Unknown	2	

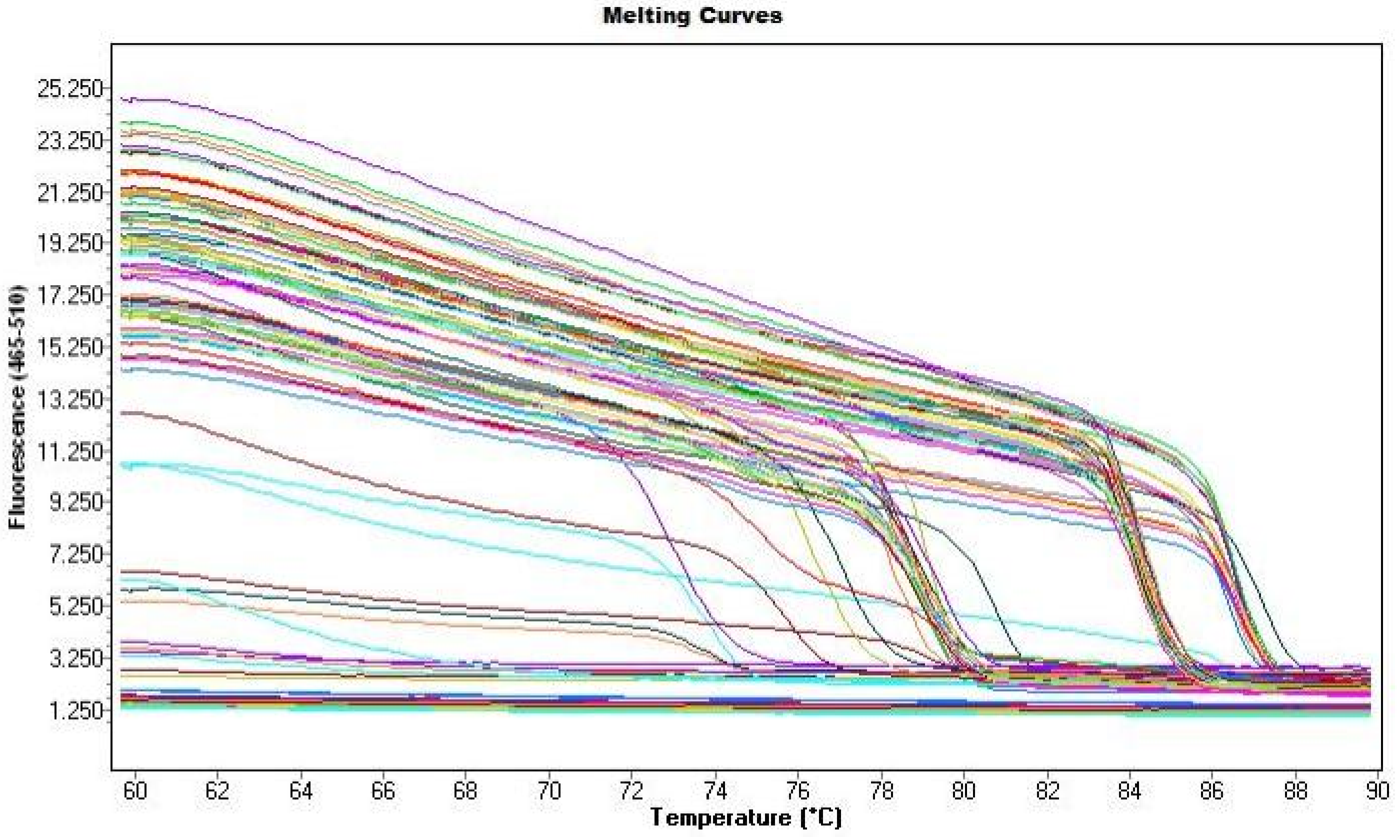
Melting Curves
Fluorescence (465-510)
25.250
23.250
21.250
19.250
17.250
15.250
13.250
11.250
9.250
7.250
5.250
3.250
1.250
60
62
64
66
68
70
72
74
76
78
80
82
84
86
88
90
Temperature (°C)

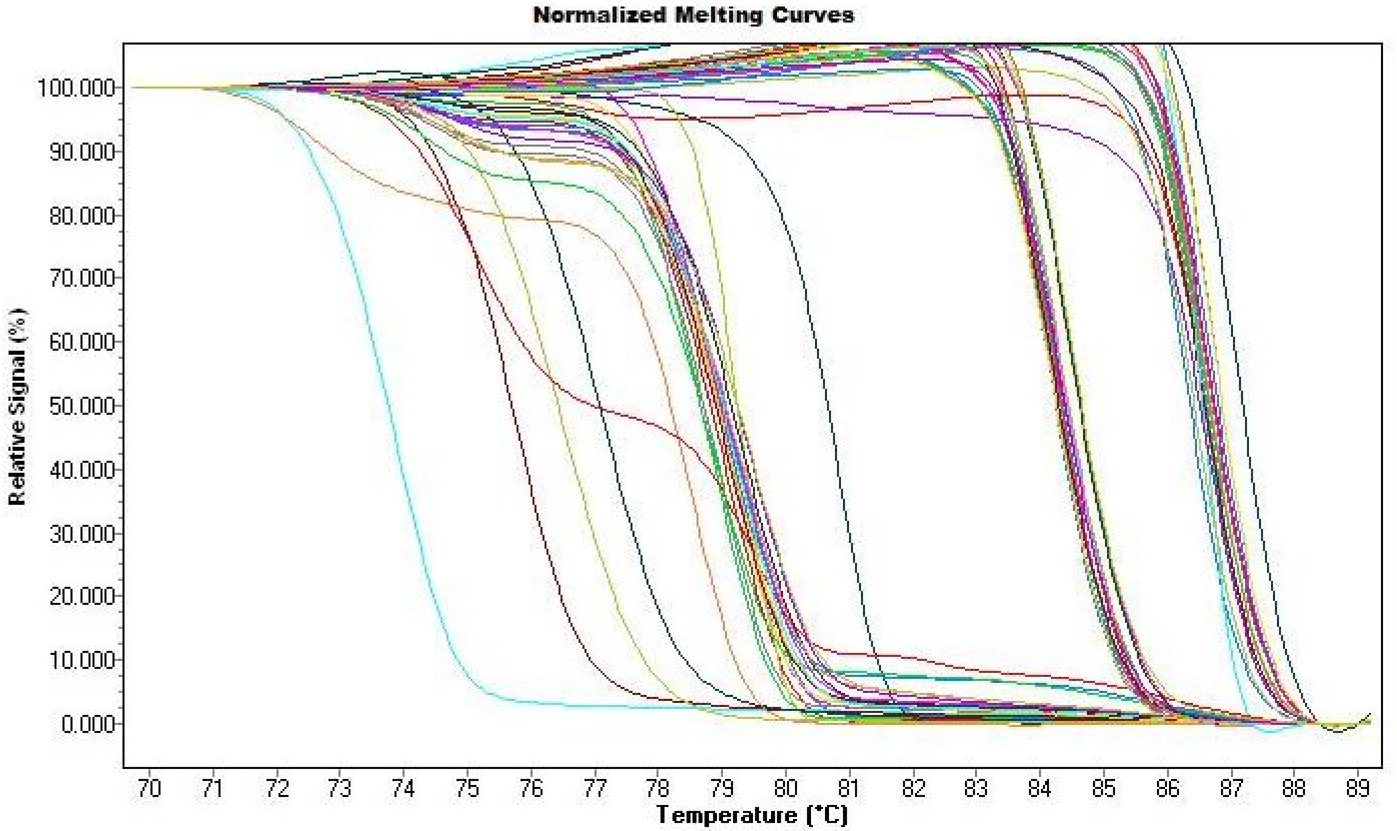
Normalized Melting Curves
Relative Signal (%)
100.000
90.000
80.000
70.000
60.000
50.000
40.000
30.000
20.000
10.000
0.000
70
71
72
73
74
75
76
77
78
79
80
81
82
83
84
85
86
87
88
89
Temperature (°C)

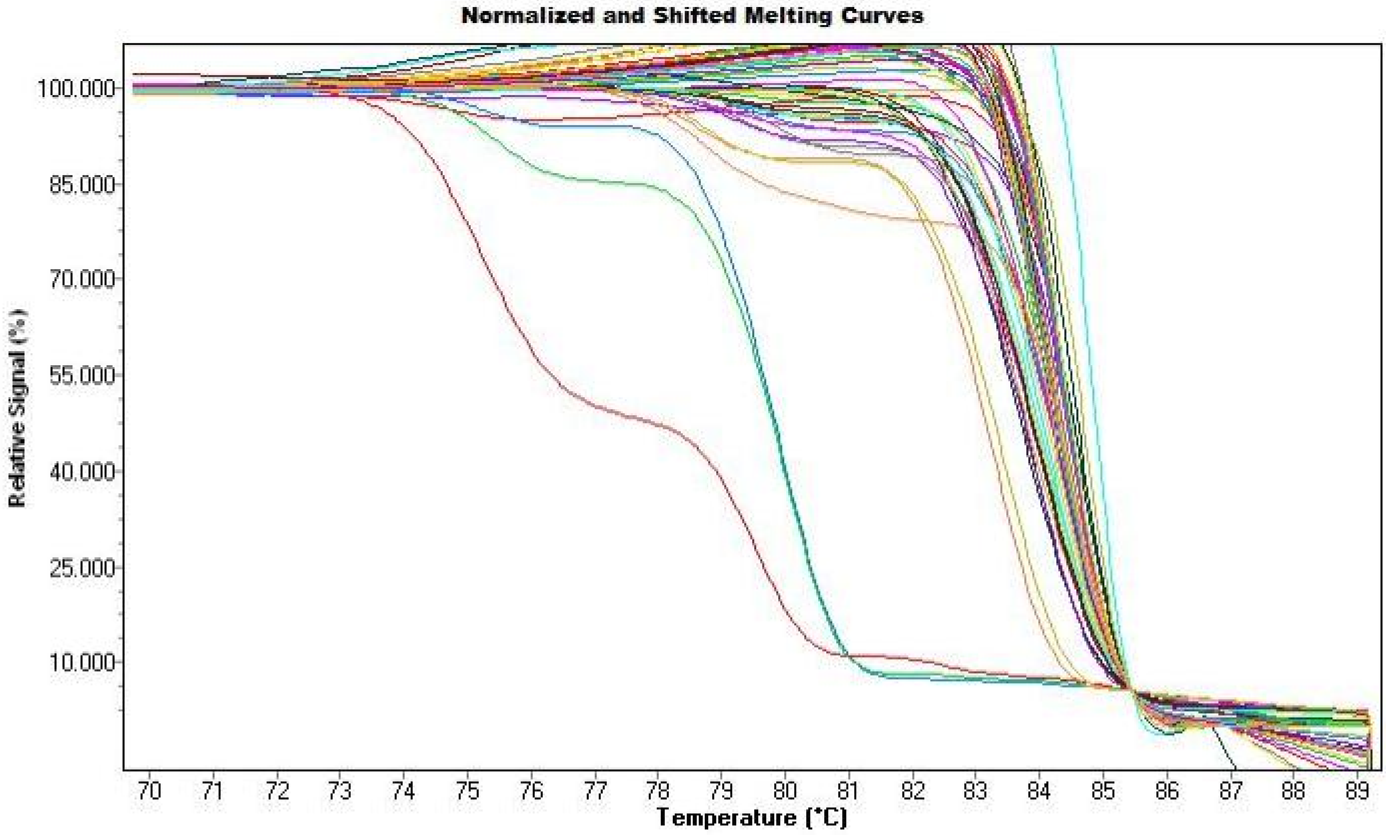

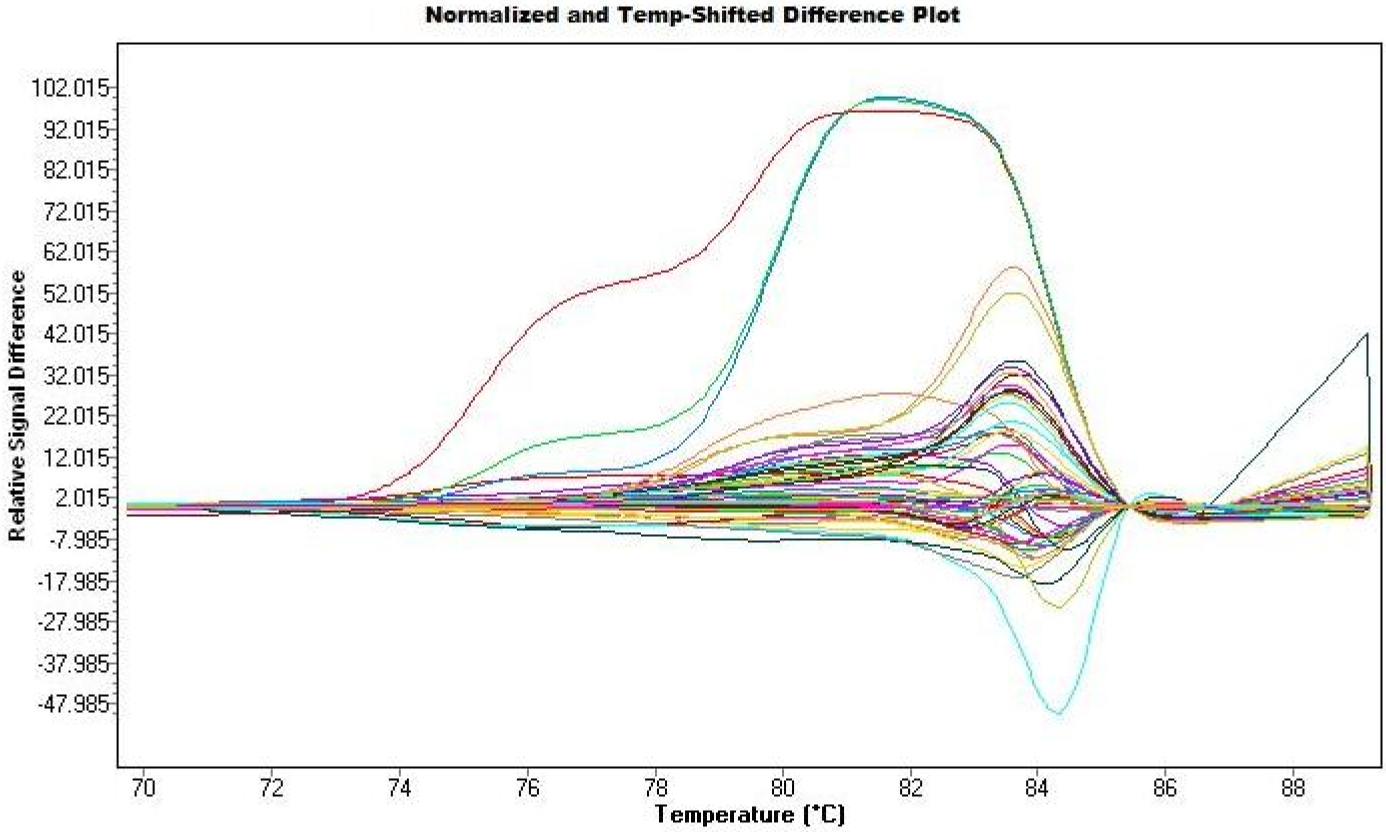

Analysis Notes	

Melt Curve Genotyping for All Samples (Melt Curve Genotyping)

Channel	465-510	Color Compensation	Off				
Progam Name	Program						
Stds Settings	Auto-Group						
Sensitivity	Normal	Temp Range	61-86	Score	0.7	Res	0.1

Subset Name	All Samples

Results

Inc	Pos.	Sample Name	Sample Type	Group	Score	Resolution	Status
☑	A1	GAPDH 15	Unknown	Negative	1.00	1.00	
☑	A2	GAPDH 15	Unknown	Negative	1.00	1.00	
☑	A3	GAPDH 15	Unknown	Negative	1.00	1.00	
☑	A4	GAPDH 18	Unknown	Negative	1.00	1.00	
☑	A5	GAPDH 18	Unknown	Negative	1.00	1.00	
☑	A6	GAPDH 18	Unknown	Negative	1.00	1.00	
☑	A7	GAPDH 19	Unknown	Negative	1.00	1.00	
☑	A8	GAPDH 19	Unknown	Negative	1.00	1.00	
☑	A9	GAPDH 19	Unknown	Negative	1.00	1.00	
☑	A10	GAPDH 20	Unknown	Negative	1.00	1.00	
☑	A11	GAPDH 20	Unknown	Negative	1.00	1.00	
☑	A12	GAPDH 20	Unknown	Negative	1.00	1.00	
☑	B1	FCER1A 15	Unknown	Negative	1.00	1.00	
☑	B2	FCER1A 15	Unknown	Negative	1.00	1.00	
☑	B3	FCER1A 15	Unknown	Negative	1.00	1.00	
☑	B4	FCER1A 18	Unknown	Negative	1.00	1.00	
☑	B5	FCER1A 18	Unknown	Negative	1.00	1.00	
☑	B6	FCER1A 18	Unknown	Negative	1.00	1.00	
☑	B7	FCER1A 19	Unknown	Negative	1.00	1.00	
☑	B8	FCER1A 19	Unknown	Negative	1.00	1.00	
☑	B9	FCER1A 19	Unknown	Negative	1.00	1.00	
☑	B10	FCER1A 20	Unknown	Negative	1.00	1.00	
☑	B11	FCER1A 20	Unknown	Negative	1.00	1.00	
☑	B12	FCER1A 20	Unknown	Negative	1.00	1.00	
☑	C1	RGS1 15	Unknown	1	1.00	0.69	
☑	C2	RGS1 15	Unknown	1	0.98	0.49	
☑	C3	RGS1 15	Unknown	1	0.99	0.63	
☑	C4	RGS1 18	Unknown	1	0.99	0.66	
☑	C5	RGS1 18	Unknown	1	0.99	0.67	
☑	C6	RGS1 18	Unknown	1	0.99	0.63	
☑	C7	RGS1 19	Unknown	1	0.98	0.77	

Results

Inc	Pos.	Sample Name	Sample Type	Group	Score	Resolution	Status
☑	C8	RGS1 19	Unknown	1	0.72	0.52	
☑	C9	RGS1 19	Unknown	1	0.99	0.54	
☑	C10	RGS1 20	Unknown	1	0.98	0.62	
☑	C11	RGS1 20	Unknown	1	0.98	0.61	
☑	C12	RGS1 20	Unknown	1	0.99	0.64	
☑	D1	GAPDH 24	Unknown	2	0.98	0.60	
☑	D2	GAPDH 24	Unknown	2	0.96	0.60	
☑	D3	GAPDH 24	Unknown	2	0.93	0.61	
☑	D4	GAPDH 26	Unknown	2	0.92	0.64	
☑	D5	GAPDH 26	Unknown	2	0.99	0.51	
☑	D6	GAPDH 26	Unknown	2	0.96	0.61	
☑	D7		Unknown	Unknown	0.24	0.10	
☑	D8		Unknown	3	0.83	0.66	
☑	D9		Unknown	3	1.00	0.79	
☑	D10		Unknown	3	0.99	0.78	
☑	D11		Unknown	6	1.00	0.42	
☑	D12		Unknown	Negative	1.00	1.00	
☑	E1	FCER1A 24	Unknown	1	0.72	0.71	
☑	E2	FCER1A 24	Unknown	1	0.76	0.76	
☑	E3	FCER1A 24	Unknown	1	0.78	0.77	
☑	E4	FCER1A 26	Unknown	1	0.83	0.82	
☑	E5	FCER1A 26	Unknown	1	0.82	0.81	
☑	E6	FCER1A 26	Unknown	1	0.83	0.82	
☑	E7		Unknown	Unknown	0.60	0.01	
☑	E8		Unknown	Unknown	0.68	0.45	
☑	E9		Unknown	2	0.86	0.60	
☑	E10		Unknown	4	0.88	0.15	
☑	E11		Unknown	4	0.98	0.45	
☑	E12		Unknown	2	0.91	0.64	
☑	F1	RGS1 24	Unknown	1	0.98	0.55	
☑	F2	RGS1 24	Unknown	1	0.99	0.61	
☑	F3	RGS1 24	Unknown	1	0.97	0.63	
☑	F4	RGS1 26	Unknown	1	0.98	0.59	
☑	F5	RGS1 26	Unknown	1	0.97	0.60	
☑	F6	RGS1 26	Unknown	1	0.98	0.58	
☑	F7		Unknown	Unknown	0.66	0.14	
☑	F8		Unknown	4	1.00	0.42	
☑	F9		Unknown	2	0.73	0.16	

Results

Inc	Pos.	Sample Name	Sample Type	Group	Score	Resolution	Status
☑	F10		Unknown	2	0.77	0.12	
☑	F11		Unknown	3	0.92	0.82	
☑	F12		Unknown	Unknown	0.51	0.10	
☑	G1		Unknown	2	0.98	0.47	
☑	G2		Unknown	5	1.00	0.52	
☑	G3		Unknown	2	0.94	0.27	
☑	G4		Unknown	2	0.98	0.59	
☑	G5		Unknown	2	1.00	0.52	
☑	G6		Unknown	2	0.98	0.58	
☑	G7		Unknown	2	0.96	0.61	
☑	G8		Unknown	2	0.97	0.60	
☑	G9		Unknown	2	0.98	0.62	
☑	G10		Unknown	2	0.98	0.43	
☑	G11		Unknown	2	0.99	0.56	
☑	G12		Unknown	2	0.97	0.42	
☑	H1		Unknown	1	0.74	0.73	
☑	H2		Unknown	1	0.72	0.71	
☑	H3		Unknown	1	0.71	0.71	
☑	H4		Unknown	1	0.71	0.71	
☑	H5		Unknown	Unknown	0.64	0.64	
☑	H6		Unknown	1	0.71	0.71	
☑	H7		Unknown	1	0.74	0.73	
☑	H8		Unknown	1	0.74	0.74	
☑	H9		Unknown	1	0.81	0.81	
☑	H10		Unknown	1	0.73	0.73	
☑	H11		Unknown	1	0.73	0.73	
☑	H12		Unknown	1	0.82	0.81	

**Group**	1

Inc	Pos	Sample Name	Sample Type	Score	Resolution	Status
☑	C1	RGS1 15	Unknown	1.00	0.69	
☑	C2	RGS1 15	Unknown	0.98	0.49	
☑	C3	RGS1 15	Unknown	0.99	0.63	
☑	C4	RGS1 18	Unknown	0.99	0.66	
☑	C5	RGS1 18	Unknown	0.99	0.67	
☑	C6	RGS1 18	Unknown	0.99	0.63	
☑	C7	RGS1 19	Unknown	0.98	0.77	
☑	C8	RGS1 19	Unknown	0.72	0.52	

**Group**	1

Inc	Pos	Sample Name	Sample Type	Score	Resolution	Status
☑	C9	RGS1 19	Unknown	0.99	0.54	
☑	C10	RGS1 20	Unknown	0.98	0.62	
☑	C11	RGS1 20	Unknown	0.98	0.61	
☑	C12	RGS1 20	Unknown	0.99	0.64	
☑	E1	FCER1A 24	Unknown	0.72	0.71	
☑	E2	FCER1A 24	Unknown	0.76	0.76	
☑	E3	FCER1A 24	Unknown	0.78	0.77	
☑	E4	FCER1A 26	Unknown	0.83	0.82	
☑	E5	FCER1A 26	Unknown	0.82	0.81	
☑	E6	FCER1A 26	Unknown	0.83	0.82	
☑	F1	RGS1 24	Unknown	0.98	0.55	
☑	F2	RGS1 24	Unknown	0.99	0.61	
☑	F3	RGS1 24	Unknown	0.97	0.63	
☑	F4	RGS1 26	Unknown	0.98	0.59	
☑	F5	RGS1 26	Unknown	0.97	0.60	
☑	F6	RGS1 26	Unknown	0.98	0.58	
☑	H1		Unknown	0.74	0.73	
☑	H2		Unknown	0.72	0.71	
☑	H3		Unknown	0.71	0.71	
☑	H4		Unknown	0.71	0.71	
☑	H6		Unknown	0.71	0.71	
☑	H7		Unknown	0.74	0.73	
☑	H8		Unknown	0.74	0.74	
☑	H9		Unknown	0.81	0.81	
☑	H10		Unknown	0.73	0.73	
☑	H11		Unknown	0.73	0.73	
☑	H12		Unknown	0.82	0.81	

**Group**	2

Inc	Pos	Sample Name	Sample Type	Score	Resolution	Status
☑	D1	GAPDH 24	Unknown	0.98	0.60	
☑	D2	GAPDH 24	Unknown	0.96	0.60	
☑	D3	GAPDH 24	Unknown	0.93	0.61	
☑	D4	GAPDH 26	Unknown	0.92	0.64	
☑	D5	GAPDH 26	Unknown	0.99	0.51	
☑	D6	GAPDH 26	Unknown	0.96	0.61	
☑	E9		Unknown	0.86	0.60	
☑	E12		Unknown	0.91	0.64	

Group	2

Inc	Pos	Sample Name	Sample Type	Score	Resolution	Status
☑	F9		Unknown	0.73	0.16	
☑	F10		Unknown	0.77	0.12	
☑	G1		Unknown	0.98	0.47	
☑	G3		Unknown	0.94	0.27	
☑	G4		Unknown	0.98	0.59	
☑	G5		Unknown	1.00	0.52	
☑	G6		Unknown	0.98	0.58	
☑	G7		Unknown	0.96	0.61	
☑	G8		Unknown	0.97	0.60	
☑	G9		Unknown	0.98	0.62	
☑	G10		Unknown	0.98	0.43	
☑	G11		Unknown	0.99	0.56	
☑	G12		Unknown	0.97	0.42	

Group	3

Inc	Pos	Sample Name	Sample Type	Score	Resolution	Status
☑	D8		Unknown	0.83	0.66	
☑	D9		Unknown	1.00	0.79	
☑	D10		Unknown	0.99	0.78	
☑	F11		Unknown	0.92	0.82	

Group	4

Inc	Pos	Sample Name	Sample Type	Score	Resolution	Status
☑	E10		Unknown	0.88	0.15	
☑	E11		Unknown	0.98	0.45	
☑	F8		Unknown	1.00	0.42	

Group	5

Inc	Pos	Sample Name	Sample Type	Score	Resolution	Status
☑	G2		Unknown	1.00	0.52	

Group	6

Inc	Pos	Sample Name	Sample Type	Score	Resolution	Status
☑	D11		Unknown	1.00	0.42	

Group	Negative

Inc	Pos	Sample Name	Sample Type	Score	Resolution	Status

Group	Negative

Inc	Pos	Sample Name	Sample Type	Score	Resolution	Status
☑	A1	GAPDH 15	Unknown	1.00	1.00	
☑	A2	GAPDH 15	Unknown	1.00	1.00	
☑	A3	GAPDH 15	Unknown	1.00	1.00	
☑	A4	GAPDH 18	Unknown	1.00	1.00	
☑	A5	GAPDH 18	Unknown	1.00	1.00	
☑	A6	GAPDH 18	Unknown	1.00	1.00	
☑	A7	GAPDH 19	Unknown	1.00	1.00	
☑	A8	GAPDH 19	Unknown	1.00	1.00	
☑	A9	GAPDH 19	Unknown	1.00	1.00	
☑	A10	GAPDH 20	Unknown	1.00	1.00	
☑	A11	GAPDH 20	Unknown	1.00	1.00	
☑	A12	GAPDH 20	Unknown	1.00	1.00	
☑	B1	FCER1A 15	Unknown	1.00	1.00	
☑	B2	FCER1A 15	Unknown	1.00	1.00	
☑	B3	FCER1A 15	Unknown	1.00	1.00	
☑	B4	FCER1A 18	Unknown	1.00	1.00	
☑	B5	FCER1A 18	Unknown	1.00	1.00	
☑	B6	FCER1A 18	Unknown	1.00	1.00	
☑	B7	FCER1A 19	Unknown	1.00	1.00	
☑	B8	FCER1A 19	Unknown	1.00	1.00	
☑	B9	FCER1A 19	Unknown	1.00	1.00	
☑	B10	FCER1A 20	Unknown	1.00	1.00	
☑	B11	FCER1A 20	Unknown	1.00	1.00	
☑	B12	FCER1A 20	Unknown	1.00	1.00	
☑	D12		Unknown	1.00	1.00	

Group	Unknown

Inc	Pos	Sample Name	Sample Type	Score	Resolution	Status
☑	D7		Unknown	0.24	0.10	
☑	E7		Unknown	0.60	0.01	
☑	E8		Unknown	0.68	0.45	
☑	F7		Unknown	0.66	0.14	
☑	F12		Unknown	0.51	0.10	
☑	H5		Unknown	0.64	0.64	

Melting Curves

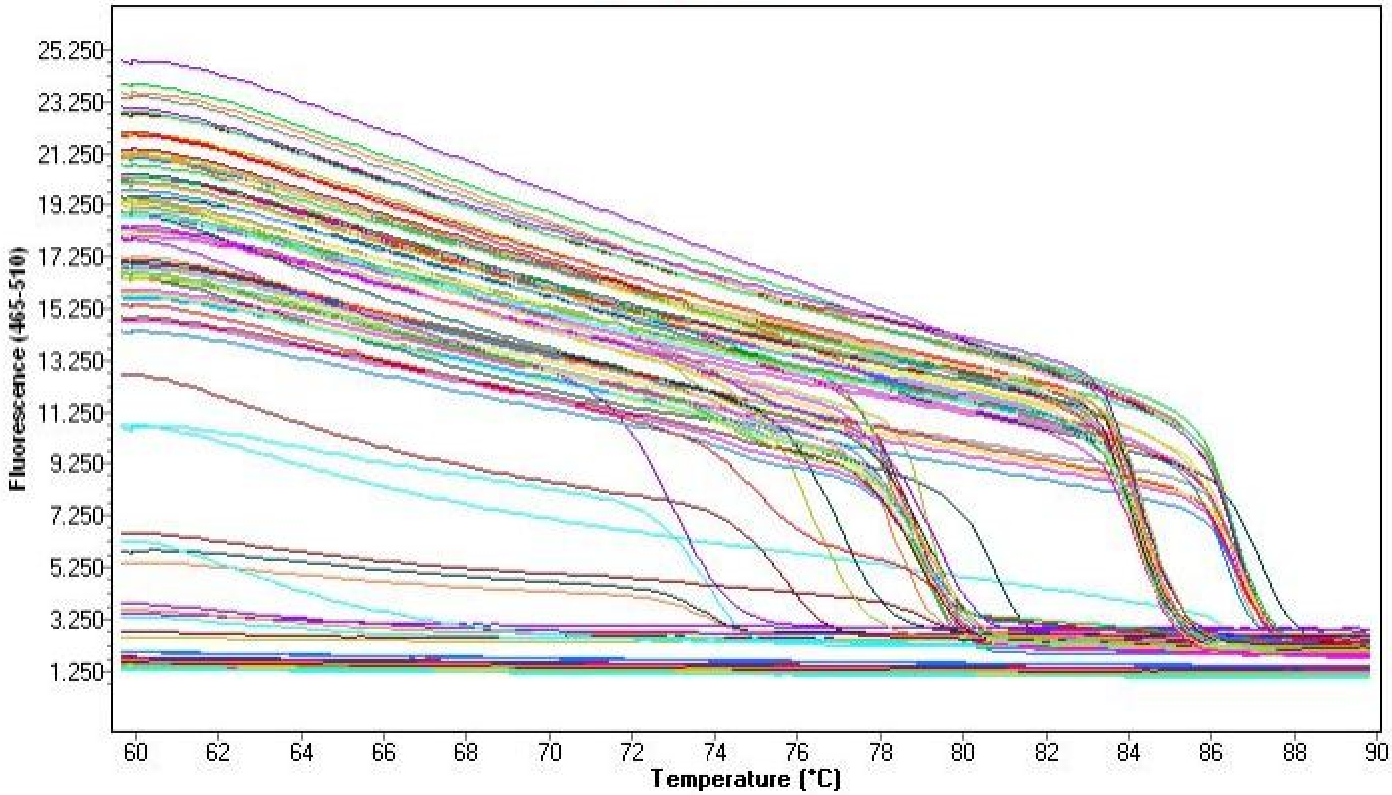

Melting Peaks

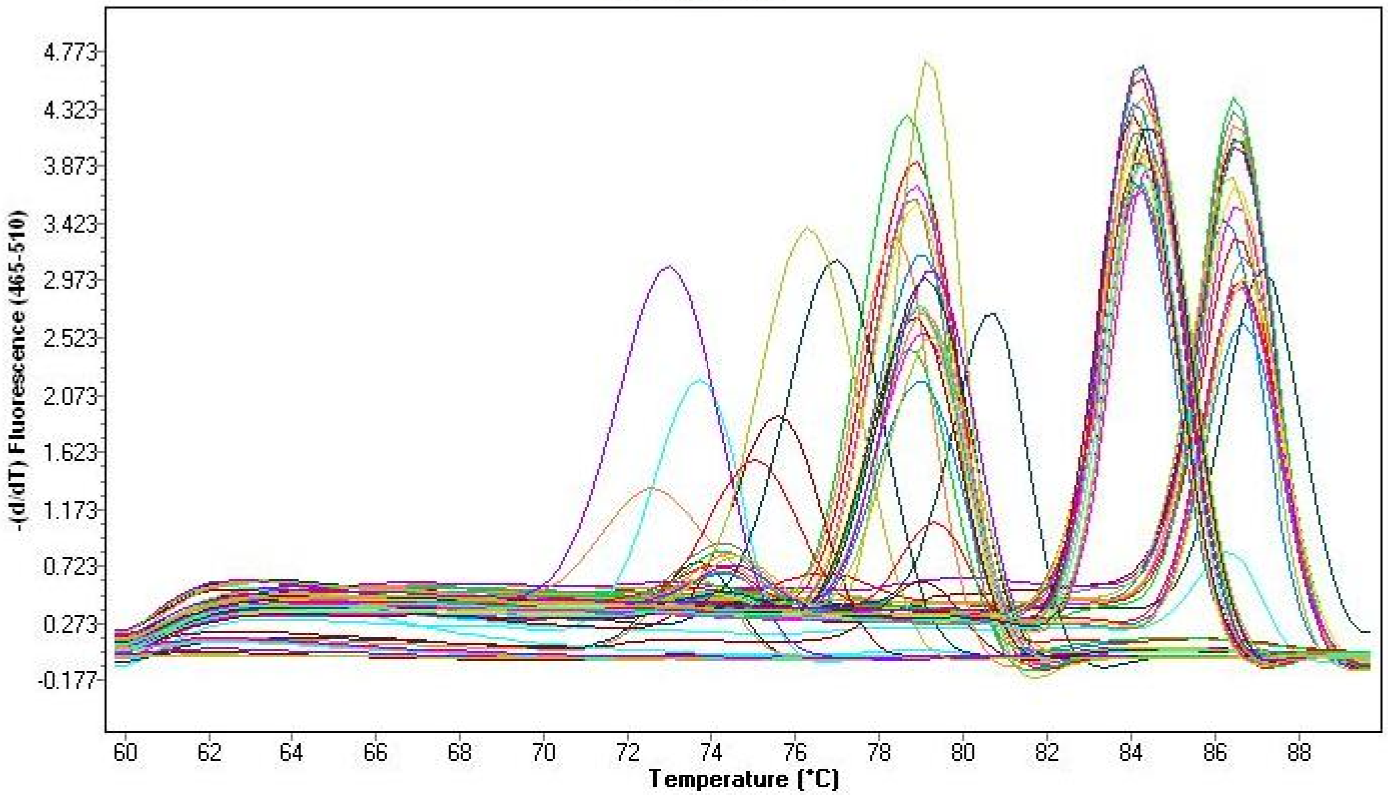

Analysis Notes	

Tm Calling for All Samples (Tm Calling)

Settings

Channel	465-510	Color Compensation	Off		
Program	Program				
Show Shoulders	False	Chemistry	HybProbe	Max Peaks	2

Subset Name	All Samples

Results

			Peak 1				Peak 2				
Inc	Pos	Sample Name	Tm	Area	Width	Height	Tm	Area	Width	Height	Status
☑	A1	GAPDH 15									
☑	A2	GAPDH 15									
☑	A3	GAPDH 15									
☑	A4	GAPDH 18									
☑	A5	GAPDH 18									
☑	A6	GAPDH 18									
☑	A7	GAPDH 19									
☑	A8	GAPDH 19									
☑	A9	GAPDH 19									
☑	A10	GAPDH 20									
☑	A11	GAPDH 20									
☑	A12	GAPDH 20									
☑	B1	FCER1A 15									
☑	B2	FCER1A 15									
☑	B3	FCER1A 15									
☑	B4	FCER1A 18									
☑	B5	FCER1A 18									
☑	B6	FCER1A 18									
☑	B7	FCER1A 19									
☑	B8	FCER1A 19									
☑	B9	FCER1A 19									
☑	B10	FCER1A 20									
☑	B11	FCER1A 20									
☑	B12	FCER1A 20									
☑	C1	RGS1 15	86.38	7.75	2.20	3.52					
☑	C2	RGS1 15	86.65	8.02	2.38	3.37					
☑	C3	RGS1 15	86.60	10.30	2.28	4.52					
☑	C4	RGS1 18	86.66	9.22	2.49	3.70					
☑	C5	RGS1 18	86.60	10.98	2.49	4.41					

Results

Inc	Pos	Sample Name	Peak 1 Tm	Peak 1 Area	Peak 1 Width	Peak 1 Height	Peak 2 Tm	Peak 2 Area	Peak 2 Width	Peak 2 Height	Status
☑	C6	RGS1 18	86.64	9.54	2.52	3.79					
☑	C7	RGS1 19	86.63	11.15	2.58	4.32					
☑	C8	RGS1 19	86.19	1.82	3.76	0.48					
☑	C9	RGS1 19									
☑	C10	RGS1 20	86.64	10.43	2.42	4.31					
☑	C11	RGS1 20	86.67	9.44	2.25	4.19					
☑	C12	RGS1 20	86.48	8.60	2.19	3.92					
☑	D1	GAPDH 24	74.03	0.58	1.73	0.33	79.04	8.37	2.53	3.31	
☑	D2	GAPDH 24	73.82	0.55	1.72	0.32	78.89	10.31	2.53	4.07	
☑	D3	GAPDH 24	78.67	11.05	2.49	4.44					
☑	D4	GAPDH 26	78.90	9.25	2.41	3.84					
☑	D5	GAPDH 26	74.10	0.95	1.77	0.53	78.82	8.59	2.33	3.69	
☑	D6	GAPDH 26	78.92	9.09	2.45	3.70					
☑	D7										
☑	D8		73.73	5.52	2.44	2.26					
☑	D9		73.64	1.87	2.44	0.77					
☑	D10		73.62	1.57	2.48	0.63					
☑	D11										
☑	D12										
☑	E1	FCER1A 24	84.24	10.36	2.31	4.49					
☑	E2	FCER1A 24	84.34	10.81	2.23	4.85					
☑	E3	FCER1A 24	84.38	11.42	2.38	4.80					
☑	E4	FCER1A 26	84.58	9.13	2.30	3.97					
☑	E5	FCER1A 26	84.52	9.49	2.27	4.18					
☑	E6	FCER1A 26	84.51	9.50	2.34	4.07					
☑	E7		79.17	1.27	2.07	0.61					
☑	E8		78.58	0.29	4.58	0.06					
☑	E9		80.70	6.54	2.32	2.81					
☑	E10		66.66	0.16	2.55	0.06					
☑	E11		65.41	0.94	7.70	0.12					
☑	E12		79.25	8.86	1.85	4.80					
☑	F1	RGS1 24									
☑	F2	RGS1 24	86.72	6.97	2.29	3.04					
☑	F3	RGS1 24	86.68	10.04	2.38	4.22					
☑	F4	RGS1 26	86.73	6.82	2.31	2.95					
☑	F5	RGS1 26									

Results

Inc	Pos	Sample Name	Peak 1 Tm	Area	Width	Height	Peak 2 Tm	Area	Width	Height	Status
☑	F6	RGS1 26									
☑	F7		75.65	5.45	2.79	1.95					
☑	F8										
☑	F9		77.03	9.22	2.88	3.20					
☑	F10		72.79	2.22	2.36	0.94	78.43	8.06	2.37	3.40	
☑	F11		73.11	9.20	2.93	3.14					
☑	F12		76.41	10.38	2.99	3.47					
☑	G1		79.00	5.28	2.46	2.15					
☑	G2		75.11	3.70	2.55	1.45	79.40	2.47	2.37	1.04	
☑	G3		74.18	0.97	1.85	0.53	78.76	5.74	2.40	2.40	
☑	G4		79.10	6.84	2.61	2.62					
☑	G5		74.11	0.57	1.52	0.37	79.03	7.33	2.58	2.84	
☑	G6		78.97	7.44	2.61	2.85					
☑	G7		78.92	7.09	2.56	2.77					
☑	G8		79.05	7.46	2.60	2.87					
☑	G9		79.16	7.94	2.61	3.04					
☑	G10		74.35	0.87	1.78	0.49	79.15	7.17	2.58	2.78	
☑	G11		74.36	0.63	1.72	0.37	79.30	8.10	2.60	3.11	
☑	G12		74.56	0.86	1.75	0.49	79.34	6.65	2.60	2.56	
☑	H1		84.19	9.14	2.40	3.80					
☑	H2		84.27	9.58	2.41	3.97					
☑	H3		84.29	9.07	2.37	3.82					
☑	H4		84.30	9.17	2.41	3.80					
☑	H5		84.24	10.41	2.45	4.25					
☑	H6		84.23	10.09	2.42	4.16					
☑	H7		84.21	10.26	2.35	4.36					
☑	H8		84.38	9.24	2.32	3.98					
☑	H9		84.54	9.98	2.30	4.33					
☑	H10		84.39	10.65	2.35	4.54					
☑	H11		84.36	11.25	2.32	4.85					
☑	H12		84.57	10.13	2.28	4.44					

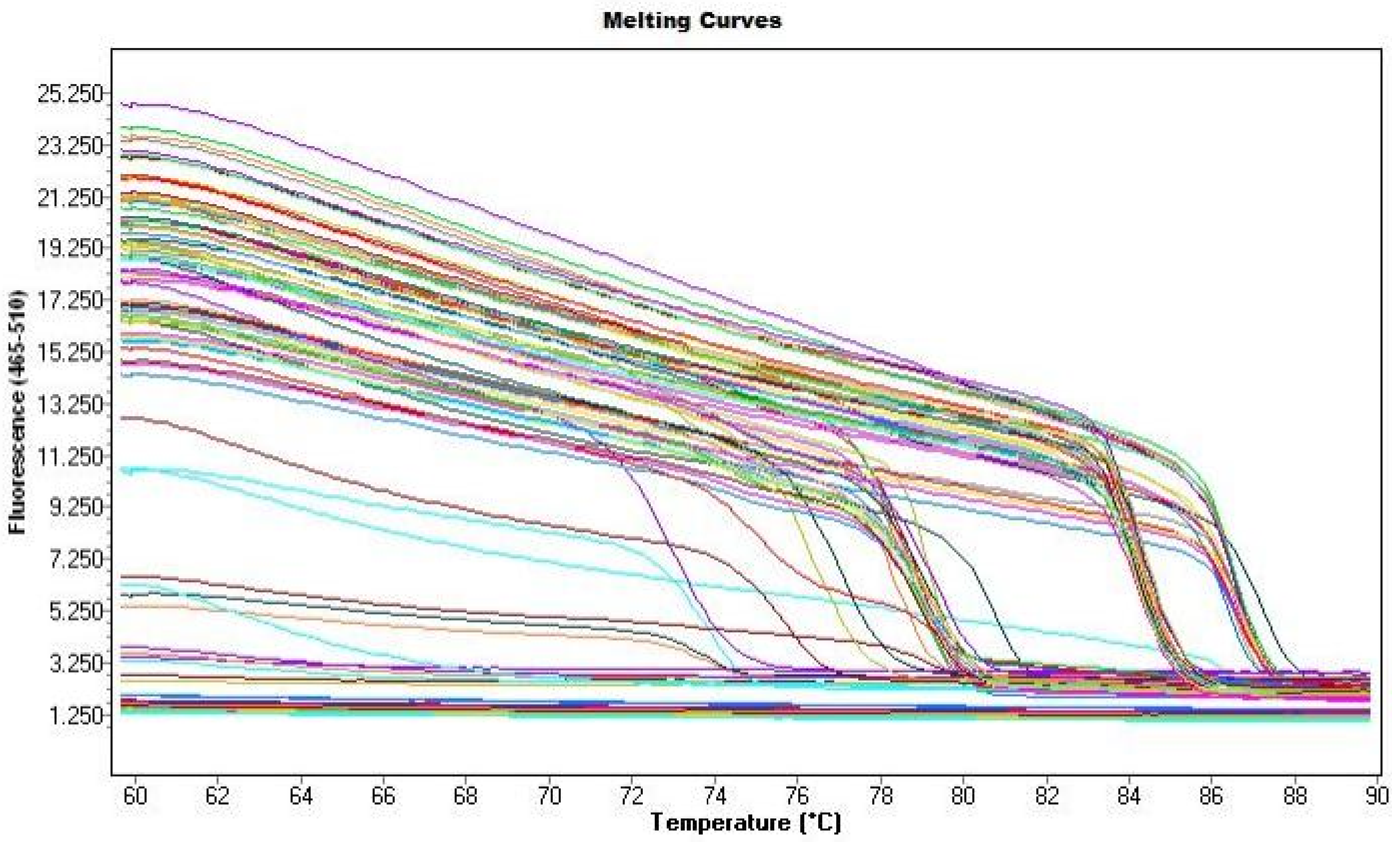

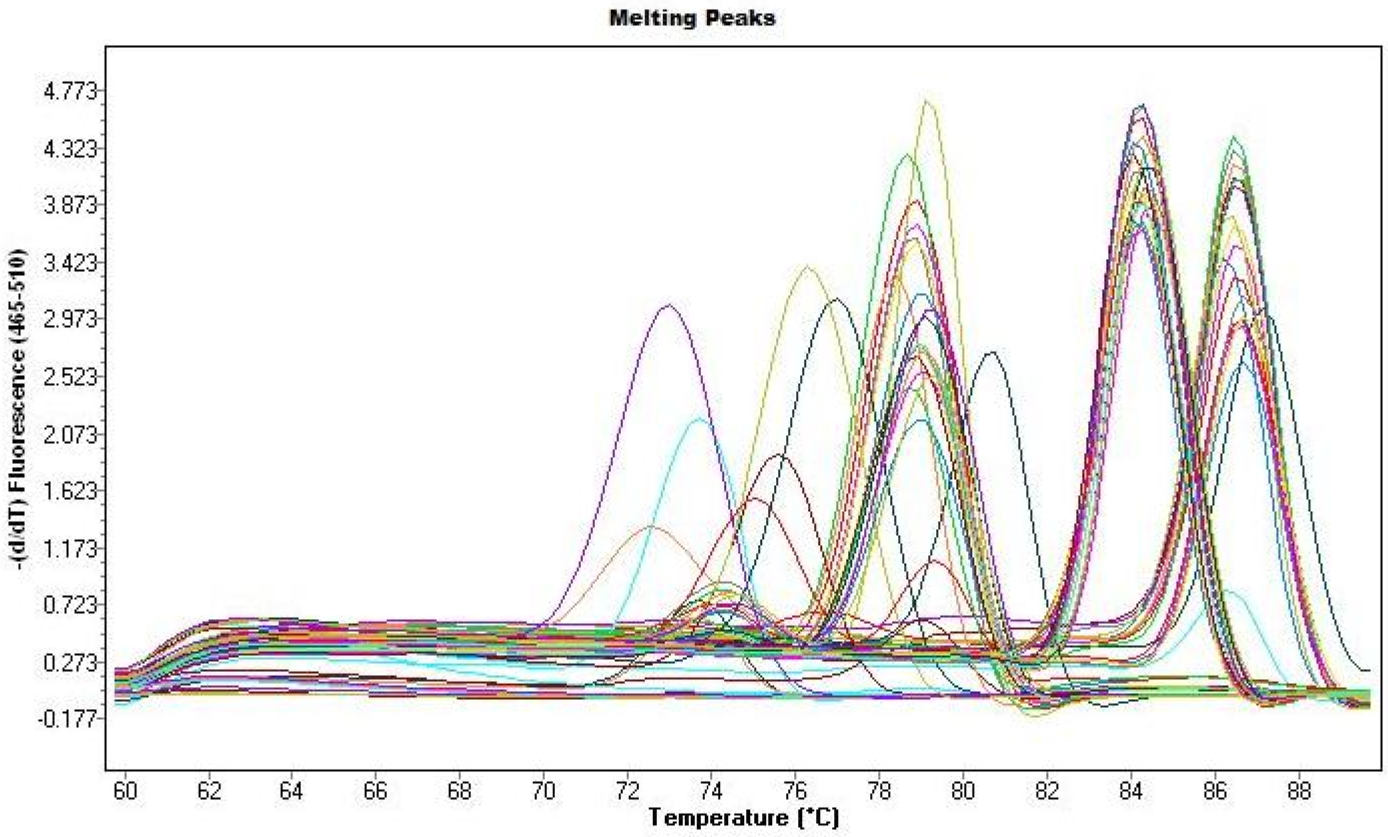

Analysis Notes	
