# Supplementary material for: Exploring differential gene expression and biomarker potential in systemic lupus erythematosus: a retrospective study
Source: PeerJ. 2025 Sep 8;13:e19891. doi: 10.7717/peerj.19891 (PMC12424612; doi:10.7717/peerj.19891)
Supplement: Supplemental Information 1 — The data include the Ct value for each sample, the Ct value for reference genes such as GAPDH, and the relative expression levels calculated using the ΔΔCt method. These data were used to validate the RNA sequencing results and further evaluate the potential of these genes as biomarkers for SLE. [file peerj-13-19891-s001.zip › qPCR/20241212 qPCR/1212plate3 HC group.pdf]

cobas® 4800 User Defined Workflow (UDF)

20241212xj plate3

Experiment

Creation Date	12/12/2024 9:42:35 PM	Last Modified Date	12/12/2024 10:45:43 PM
Operator	System Admin	Owner	System Admin
Start Time	12/12/2024 9:43:39 PM	End Time	12/12/2024 10:42:17 PM
Run State	Completed	Software Version	LCS480 1.5.1.62 SP3 - UDF 2.1.0.26
Macro		Macro Owner	
Macro Status			
Templates	gongying	Plate ID	
Test ID		Lot ID	
Color Comp ID			
Run Notes			

Programs

Program Name	Program		
Cycles	1	Analysis Mode	None

Target (°C)	Acquisition Mode	Hold (hh:mm:ss)	Ramp Rate (°C/s)	Acquisitions (per °C)	Sec Target (°C)	Step size (°C)	Step Delay (cycles)
95	None	00:00:30	4.40		0	0	0

Program Name	Program		
Cycles	40	Analysis Mode	Quantification

Target (°C)	Acquisition Mode	Hold (hh:mm:ss)	Ramp Rate (°C/s)	Acquisitions (per °C)	Sec Target (°C)	Step size (°C)	Step Delay (cycles)
95	None	00:00:15	4.40		0	0	0
60	Single	00:00:30	2.20		0	0	0

Program Name	Program		
Cycles	1	Analysis Mode	Melting Curves

Target (°C)	Acquisition Mode	Hold (hh:mm:ss)	Ramp Rate (°C/s)	Acquisitions (per °C)	Sec Target (°C)	Step size (°C)	Step Delay (cycles)
95	None	00:01:00	4.40		0	0	0
60	None	00:00:30	2.20		0	0	0
90	Continuous		0.11	5	0	0	0

Program Name	Program		
Cycles	1	Analysis Mode	None

Target (°C)	Acquisition Mode	Hold (hh:mm:ss)	Ramp Rate (°C/s)	Acquisitions (per °C)	Sec Target (°C)	Step size (°C)	Step Delay (cycles)

Target (°C)	Acquisition Mode	Hold (hh:mm:ss)	Ramp Rate (°C/s)	Acquisitions (per °C)	Sec Target (°C)	Step size (°C)	Step Delay (cycles)
40	None	00:00:30	2.20		0	0	0

Samples

Sample Count	96
Subset	All Samples

Pos	Name	ID	Repl. Of	Sample Notes
A1	GAPDH 102			
A2	GAPDH 102			
A3	GAPDH 102			
A4	GAPDH 107			
A5	GAPDH 107			
A6	GAPDH 107			
A7	GAPDH 110			
A8	GAPDH 110			
A9	GAPDH 110			
A10	GAPDH 113			
A11	GAPDH 113			
A12	GAPDH 113			
B1	FCER1A 102			
B2	FCER1A 102			
B3	FCER1A 102			
B4	FCER1A 107			
B5	FCER1A 107			
B6	FCER1A 107			
B7	FCER1A 110			
B8	FCER1A 110			
B9	FCER1A 110			
B10	FCER1A 113			
B11	FCER1A 113			
B12	FCER1A 113			
C1	RGS1 102			
C2	RGS1 102			
C3	RGS1 102			
C4	RGS1 107			
C5	RGS1 107			
C6	RGS1 107			
C7	RGS1 110			
C8	RGS1 110			
C9	RGS1 110			
C10	RGS1 113			
C11	RGS1 113			
C12	RGS1 113			

Samples

Sample Count	96
Subset	All Samples

Pos	Name	ID	Repl. Of	Sample Notes
D1	GAPDH 115			
D2	GAPDH 115			
D3	GAPDH 115			
D4	GAPDH 117			
D5	GAPDH 117			
D6	GAPDH 117			
D7	GAPDH 119			
D8	GAPDH 119			
D9	GAPDH 119			
D10	GAPDH 120			
D11	GAPDH 120			
D12	GAPDH 120			
E1	FCER1A 115			
E2	FCER1A 115			
E3	FCER1A 115			
E4	FCER1A 117			
E5	FCER1A 117			
E6	FCER1A 117			
E7	FCER1A 119			
E8	FCER1A 119			
E9	FCER1A 119			
E10	FCER1A 120			
E11	FCER1A 120			
E12	FCER1A 120			
F1	RGS1 102			
F2	RGS1 102			
F3	RGS1 102			
F4	RGS1 107			
F5	RGS1 107			
F6	RGS1 107			
F7	RGS1 110			
F8	RGS1 110			
F9	RGS1 110			
F10	RGS1 113			
F11	RGS1 113			
F12	RGS1 113			
G1				
G2				
G3				

Samples

Sample Count	96
Subset	All Samples

Pos	Name	ID	Repl. Of	Sample Notes
G4				
G5				
G6				
G7				
G8				
G9				
G10				
G11				
G12				
H1				
H2				
H3				
H4				
H5				
H6				
H7				
H8				
H9				
H10				
H11				
H12				

Instrument

Name	Z480	Serial Number	56319

Revision History

Revision	Date	User	Reason

Abs Quant/2nd Derivative Max for All Samples (Abs Quant/2nd Derivative Max)

Settings

Channel	465-510		
Color Compensation	Off		
Program	Program	Units	
Mode	High Confidence		

Subset Name	All Samples

Results

> -[illegible]te	CPpo[illegible]al	l N(lasmt[illegible]ive cycles) has higher uncertainty	Type	CP	Concentration	Standard	Status

Results

Inc	Pos	Name	Type	CP	Concentration	Standard	Status
☑	A1	GAPDH 102	Unknown	35.00			>
☑	A2	GAPDH 102	Unknown	35.00			>
☑	A3	GAPDH 102	Unknown	35.00			>
☑	A4	GAPDH 107	Unknown	22.72			
☑	A5	GAPDH 107	Unknown	22.64			
☑	A6	GAPDH 107	Unknown	22.78			
☑	A7	GAPDH 110	Unknown	21.66			
☑	A8	GAPDH 110	Unknown	21.67			
☑	A9	GAPDH 110	Unknown	21.74			
☑	A10	GAPDH 113	Unknown	26.50			
☑	A11	GAPDH 113	Unknown	26.70			
☑	A12	GAPDH 113	Unknown	26.75			
☑	B1	FCER1A 102	Unknown				
☑	B2	FCER1A 102	Unknown				
☑	B3	FCER1A 102	Unknown				
☑	B4	FCER1A 107	Unknown	29.61			
☑	B5	FCER1A 107	Unknown	31.26			
☑	B6	FCER1A 107	Unknown	35.00			>
☑	B7	FCER1A 110	Unknown	32.68			
☑	B8	FCER1A 110	Unknown	32.25			
☑	B9	FCER1A 110	Unknown	29.76			
☑	B10	FCER1A 113	Unknown	28.76			
☑	B11	FCER1A 113	Unknown	34.20			
☑	B12	FCER1A 113	Unknown	35.00			>
☑	C1	RGS1 102	Unknown				
☑	C2	RGS1 102	Unknown				
☑	C3	RGS1 102	Unknown	35.00			>
☑	C4	RGS1 107	Unknown	32.27			
☑	C5	RGS1 107	Unknown	31.94			
☑	C6	RGS1 107	Unknown	32.92			
☑	C7	RGS1 110	Unknown	31.90			
☑	C8	RGS1 110	Unknown	32.08			
☑	C9	RGS1 110	Unknown	32.79			
☑	C10	RGS1 113	Unknown	35.00			>
☑	C11	RGS1 113	Unknown	35.00			>
☑	C12	RGS1 113	Unknown	35.00			>
☑	D1	GAPDH 115	Unknown	21.75			

> - Late Cp call (last five cycles) has higher uncertainty

Results

Inc	Pos	Name	Type	CP	Concentration	Standard	Status
☑	D2	GAPDH 115	Unknown	21.90			
☑	D3	GAPDH 115	Unknown	21.87			
☑	D4	GAPDH 117	Unknown	22.14			
☑	D5	GAPDH 117	Unknown	22.10			
☑	D6	GAPDH 117	Unknown	22.14			
☑	D7	GAPDH 119	Unknown	22.29			
☑	D8	GAPDH 119	Unknown	22.34			
☑	D9	GAPDH 119	Unknown	22.30			
☑	D10	GAPDH 120	Unknown	23.22			
☑	D11	GAPDH 120	Unknown	23.18			
☑	D12	GAPDH 120	Unknown	23.27			
☑	E1	FCER1A 115	Unknown	29.54			
☑	E2	FCER1A 115	Unknown	29.79			
☑	E3	FCER1A 115	Unknown	29.17			
☑	E4	FCER1A 117	Unknown	28.82			
☑	E5	FCER1A 117	Unknown	29.01			
☑	E6	FCER1A 117	Unknown	29.05			
☑	E7	FCER1A 119	Unknown	28.88			
☑	E8	FCER1A 119	Unknown	28.85			
☑	E9	FCER1A 119	Unknown	28.74			
☑	E10	FCER1A 120	Unknown	29.98			
☑	E11	FCER1A 120	Unknown	29.58			
☑	E12	FCER1A 120	Unknown	29.60			
☑	F1	RGS1 102	Unknown	30.86			
☑	F2	RGS1 102	Unknown	31.61			
☑	F3	RGS1 102	Unknown	29.98			
☑	F4	RGS1 107	Unknown	32.01			
☑	F5	RGS1 107	Unknown	31.96			
☑	F6	RGS1 107	Unknown	31.95			
☑	F7	RGS1 110	Unknown	31.95			
☑	F8	RGS1 110	Unknown	31.39			
☑	F9	RGS1 110	Unknown	31.38			
☑	F10	RGS1 113	Unknown	31.95			
☑	F11	RGS1 113	Unknown	31.48			
☑	F12	RGS1 113	Unknown	31.47			
☑	G1		Unknown				
☑	G2		Unknown				

> - Late Cp call (last five cycles) has higher uncertainty

Results

Inc	Pos	Name	Type	CP	Concentration	Standard	Status
☑	G3		Unknown				
☑	G4		Unknown				
☑	G5		Unknown				
☑	G6		Unknown				
☑	G7		Unknown				
☑	G8		Unknown				
☑	G9		Unknown				
☑	G10		Unknown				
☑	G11		Unknown				
☑	G12		Unknown				
☑	H1		Unknown				
☑	H2		Unknown				
☑	H3		Unknown				
☑	H4		Unknown				
☑	H5		Unknown				
☑	H6		Unknown				
☑	H7		Unknown				
☑	H8		Unknown				
☑	H9		Unknown				
☑	H10		Unknown				
☑	H11		Unknown				
☑	H12		Unknown				

> - Late Cp call (last five cycles) has higher uncertainty

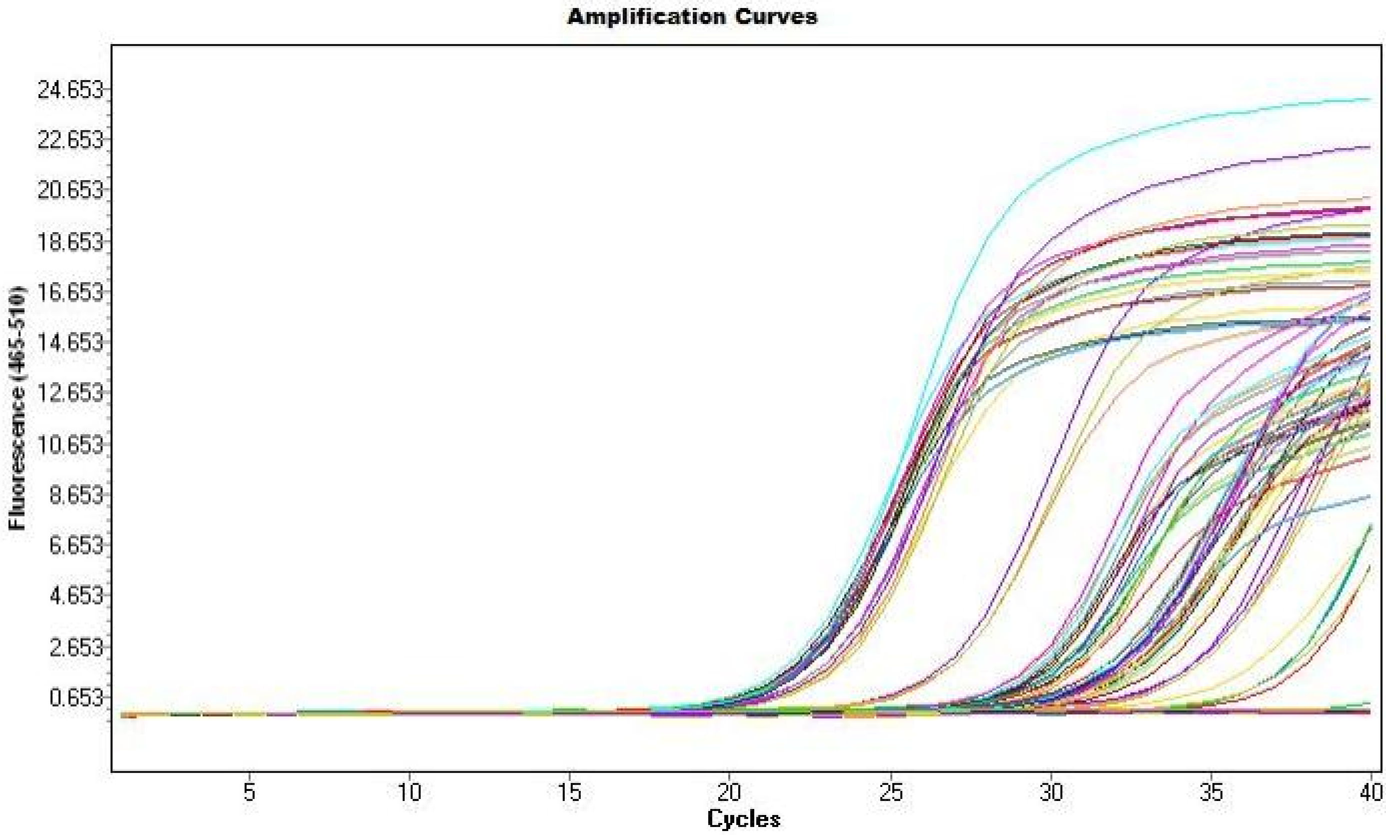

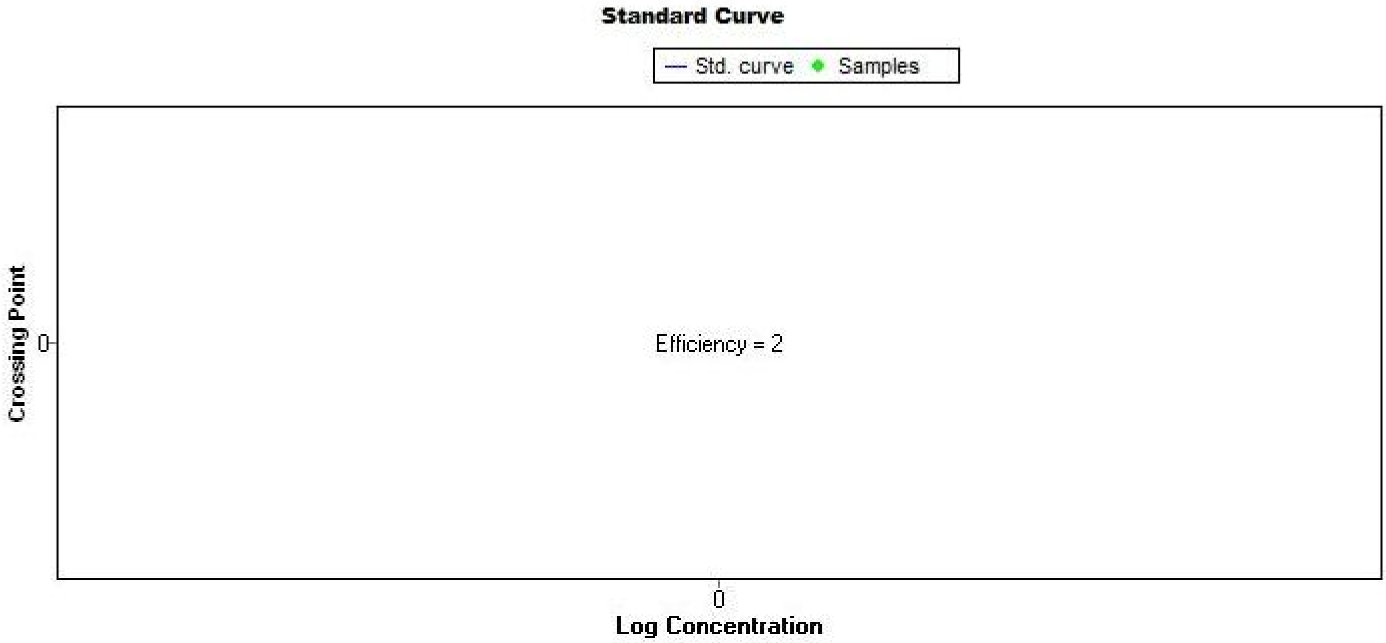

Analysis Notes	

Abs Quant/Fit Points for All Samples (Abs Quant/Fit Points)

Settings

Channel	465-510				
Color Compensation	Off				
Standard Curve	Set Efficiency (2.00)				
Program	Program			Units	
First Cycle	1	Last Cycle	40	Background	2-6
Noiseband Method	Auto	Noiseband	0.4336	STD Dev Multiplier	12.0000
# of Fit Points	2	Threshold Method	Auto	Threshold	0.4336

Subset Name	All Samples

Results

Inc	Pos	Name	Type	CP	Concentration	Standard	Status
☑	A1	GAPDH 102	Unknown	33.00			
☑	A2	GAPDH 102	Unknown	34.34			
☑	A3	GAPDH 102	Unknown	33.84			
☑	A4	GAPDH 107	Unknown	19.29			
☑	A5	GAPDH 107	Unknown	19.11			
☑	A6	GAPDH 107	Unknown	19.59			
☑	A7	GAPDH 110	Unknown	18.42			
☑	A8	GAPDH 110	Unknown	17.61			
☑	A9	GAPDH 110	Unknown	18.65			
☑	A10	GAPDH 113	Unknown	23.34			
☑	A11	GAPDH 113	Unknown	21.56			
☑	A12	GAPDH 113	Unknown	21.63			
☑	B1	FCER1A 102	Unknown				
☑	B2	FCER1A 102	Unknown				
☑	B3	FCER1A 102	Unknown				
☑	B4	FCER1A 107	Unknown	26.12			
☑	B5	FCER1A 107	Unknown	27.67			
☑	B6	FCER1A 107	Unknown	30.97			
☑	B7	FCER1A 110	Unknown	28.77			
☑	B8	FCER1A 110	Unknown	27.88			
☑	B9	FCER1A 110	Unknown	26.67			
☑	B10	FCER1A 113	Unknown	25.29			
☑	B11	FCER1A 113	Unknown	29.37			
☑	B12	FCER1A 113	Unknown	32.81			
☑	C1	RGS1 102	Unknown				
☑	C2	RGS1 102	Unknown				
☑	C3	RGS1 102	Unknown				

Results

Inc	Pos	Name	Type	CP	Concentration	Standard	Status
☑	C4	RGS1 107	Unknown	27.95			
☑	C5	RGS1 107	Unknown	27.29			
☑	C6	RGS1 107	Unknown	28.58			
☑	C7	RGS1 110	Unknown	26.91			
☑	C8	RGS1 110	Unknown	27.39			
☑	C9	RGS1 110	Unknown	27.50			
☑	C10	RGS1 113	Unknown	30.43			
☑	C11	RGS1 113	Unknown	28.65			
☑	C12	RGS1 113	Unknown	29.77			
☑	D1	GAPDH 115	Unknown	18.34			
☑	D2	GAPDH 115	Unknown	18.42			
☑	D3	GAPDH 115	Unknown	18.24			
☑	D4	GAPDH 117	Unknown	18.73			
☑	D5	GAPDH 117	Unknown	18.77			
☑	D6	GAPDH 117	Unknown	18.98			
☑	D7	GAPDH 119	Unknown	18.87			
☑	D8	GAPDH 119	Unknown	18.38			
☑	D9	GAPDH 119	Unknown	18.87			
☑	D10	GAPDH 120	Unknown	19.90			
☑	D11	GAPDH 120	Unknown	19.53			
☑	D12	GAPDH 120	Unknown	19.89			
☑	E1	FCER1A 115	Unknown	26.64			
☑	E2	FCER1A 115	Unknown	25.56			
☑	E3	FCER1A 115	Unknown	23.34			
☑	E4	FCER1A 117	Unknown	25.65			
☑	E5	FCER1A 117	Unknown	26.41			
☑	E6	FCER1A 117	Unknown	26.51			
☑	E7	FCER1A 119	Unknown	26.88			
☑	E8	FCER1A 119	Unknown	26.21			
☑	E9	FCER1A 119	Unknown	26.33			
☑	E10	FCER1A 120	Unknown	27.62			
☑	E11	FCER1A 120	Unknown	26.64			
☑	E12	FCER1A 120	Unknown	27.38			
☑	F1	RGS1 102	Unknown	27.36			
☑	F2	RGS1 102	Unknown	26.27			
☑	F3	RGS1 102	Unknown	26.67			
☑	F4	RGS1 107	Unknown	28.31			

Results

Inc	Pos	Name	Type	CP	Concentration	Standard	Status
☑	F5	RGS1 107	Unknown	28.62			
☑	F6	RGS1 107	Unknown	28.78			
☑	F7	RGS1 110	Unknown	28.90			
☑	F8	RGS1 110	Unknown	28.47			
☑	F9	RGS1 110	Unknown	28.42			
☑	F10	RGS1 113	Unknown	29.67			
☑	F11	RGS1 113	Unknown	28.97			
☑	F12	RGS1 113	Unknown	29.19			
☑	G1		Unknown				
☑	G2		Unknown				
☑	G3		Unknown				
☑	G4		Unknown				
☑	G5		Unknown				
☑	G6		Unknown				
☑	G7		Unknown				
☑	G8		Unknown				
☑	G9		Unknown				
☑	G10		Unknown				
☑	G11		Unknown				
☑	G12		Unknown				
☑	H1		Unknown	34.15			
☑	H2		Unknown				
☑	H3		Unknown				
☑	H4		Unknown				
☑	H5		Unknown				
☑	H6		Unknown				
☑	H7		Unknown				
☑	H8		Unknown				
☑	H9		Unknown				
☑	H10		Unknown				
☑	H11		Unknown				
☑	H12		Unknown				

Amplification Curves

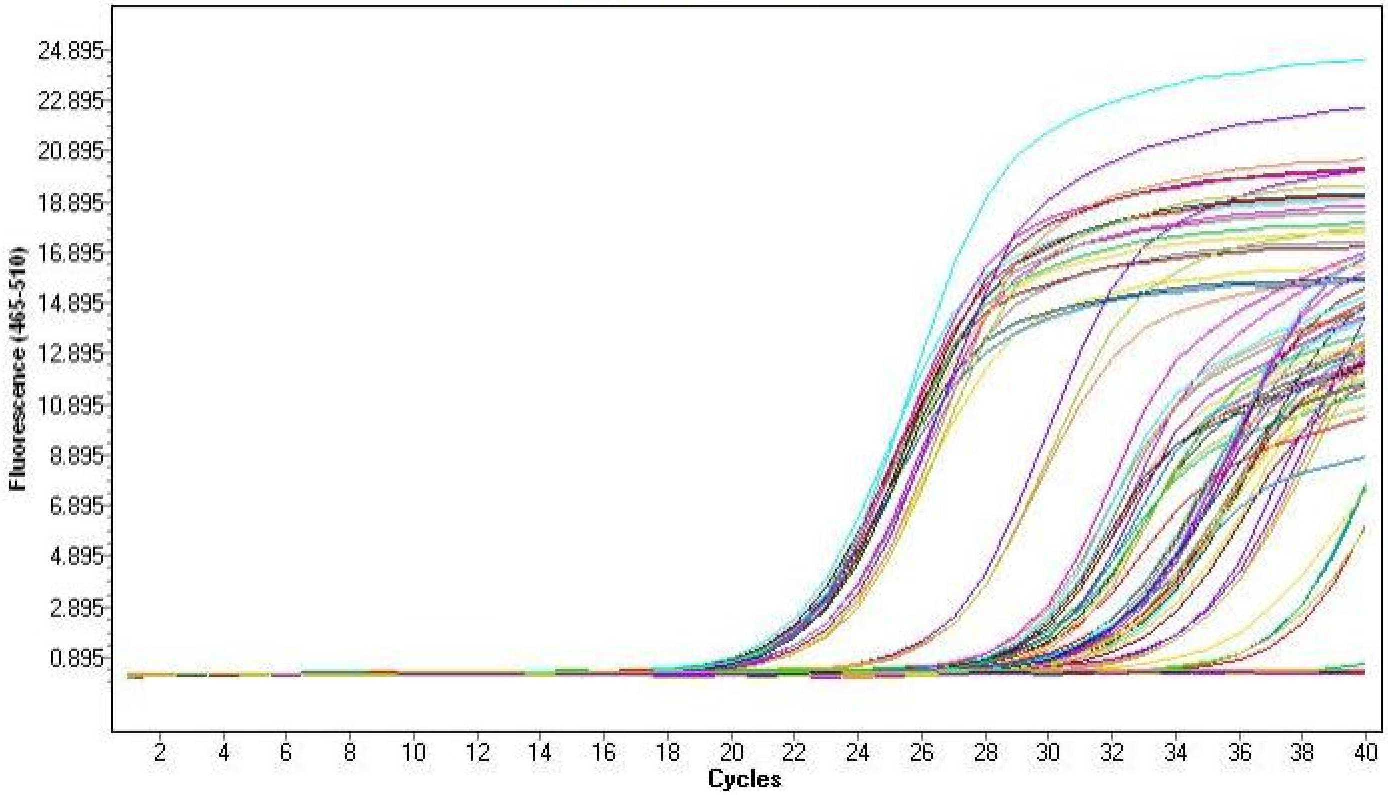

Amplification Curves

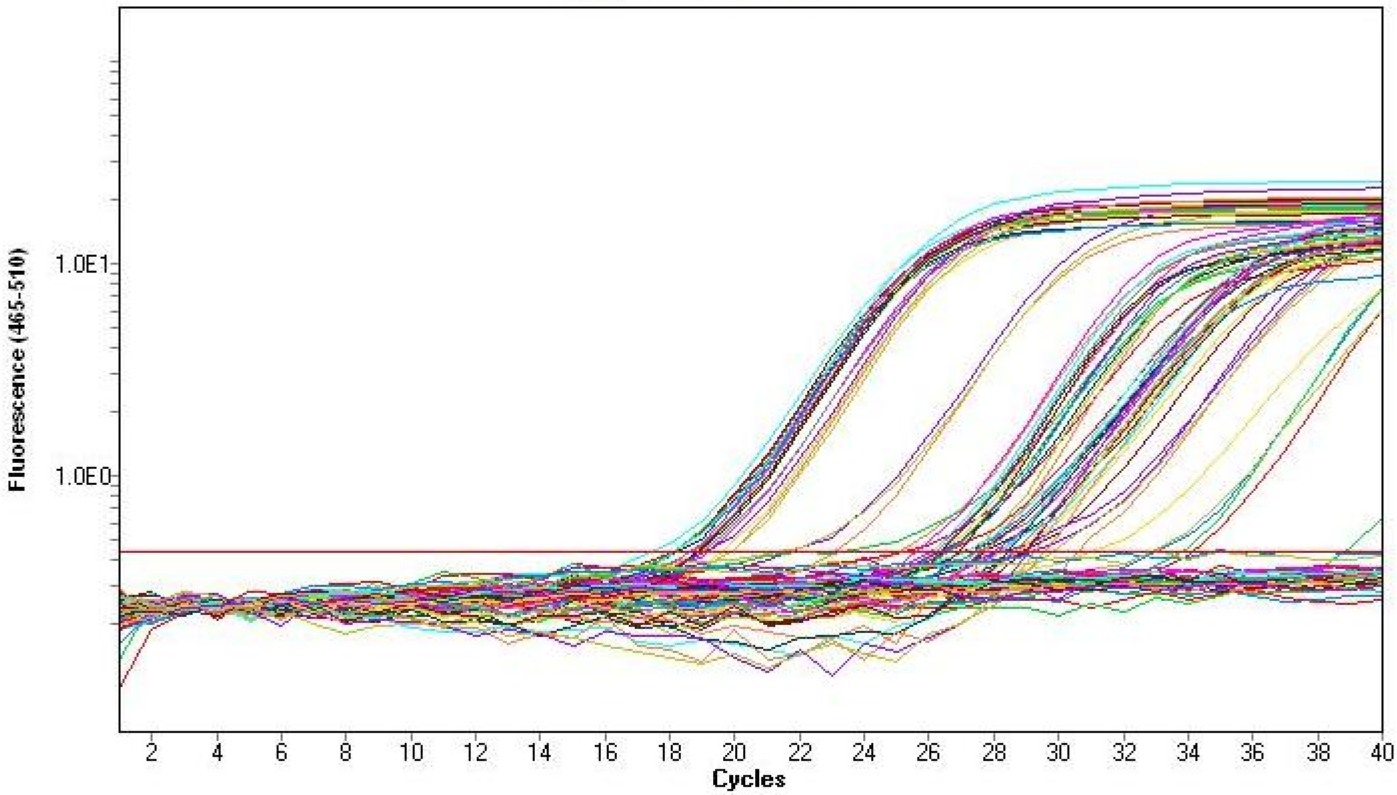

Amplification Curves

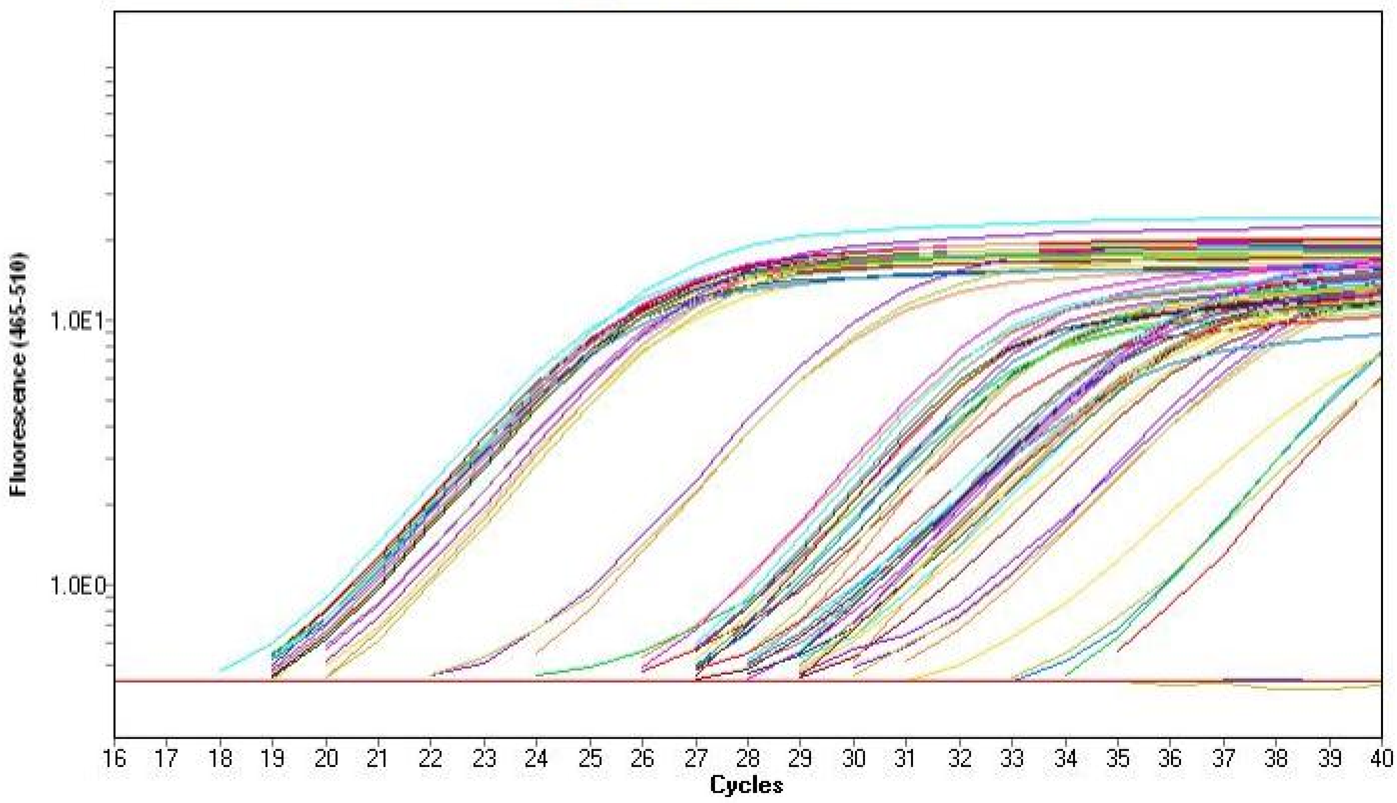

Standard Curve

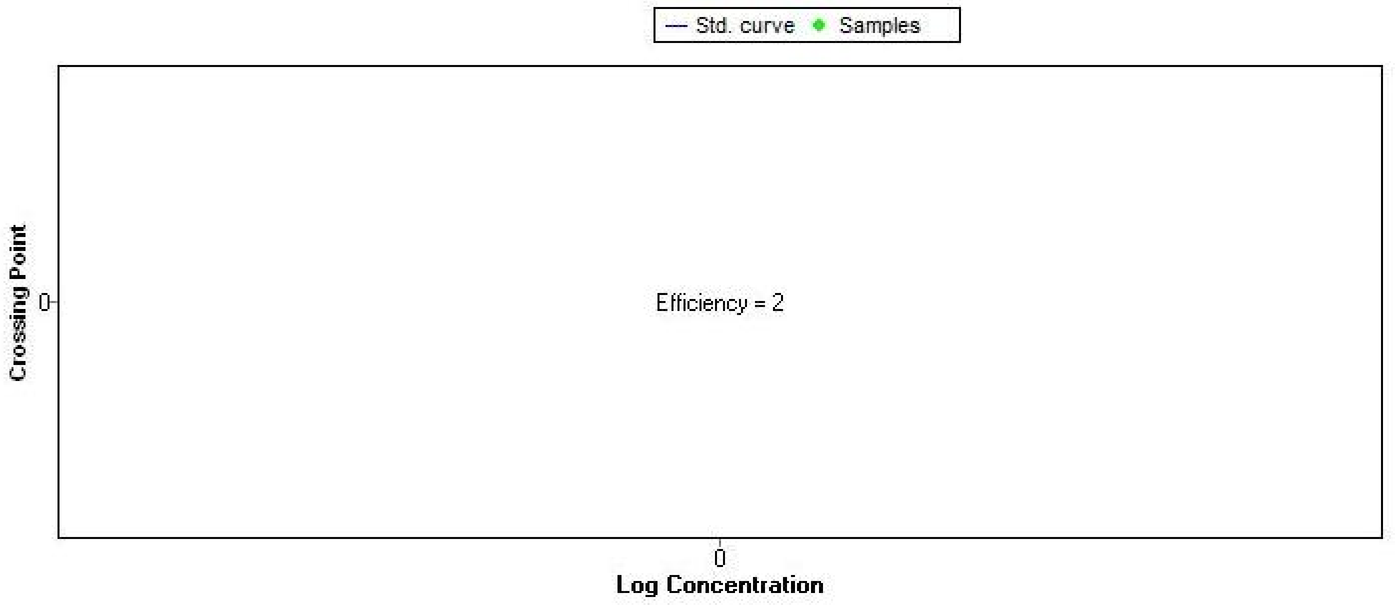

**Analysis Notes**	

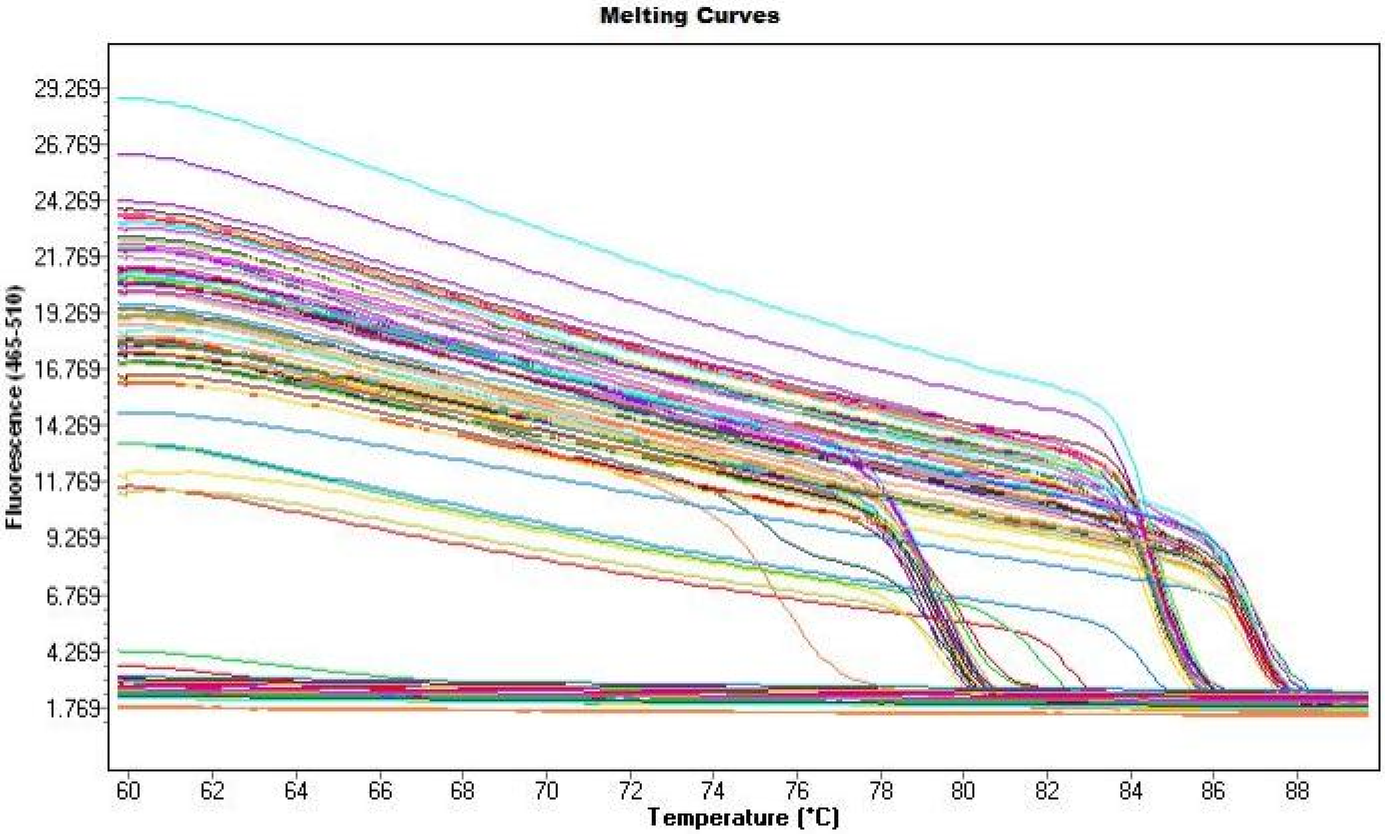
Melting Curves
Fluorescence (465-510)
29.269
26.769
24.269
21.769
19.269
16.769
14.269
11.769
9.269
6.769
4.269
1.769
60
62
64
66
68
70
72
74
76
78
80
82
84
86
88
Temperature (°C)

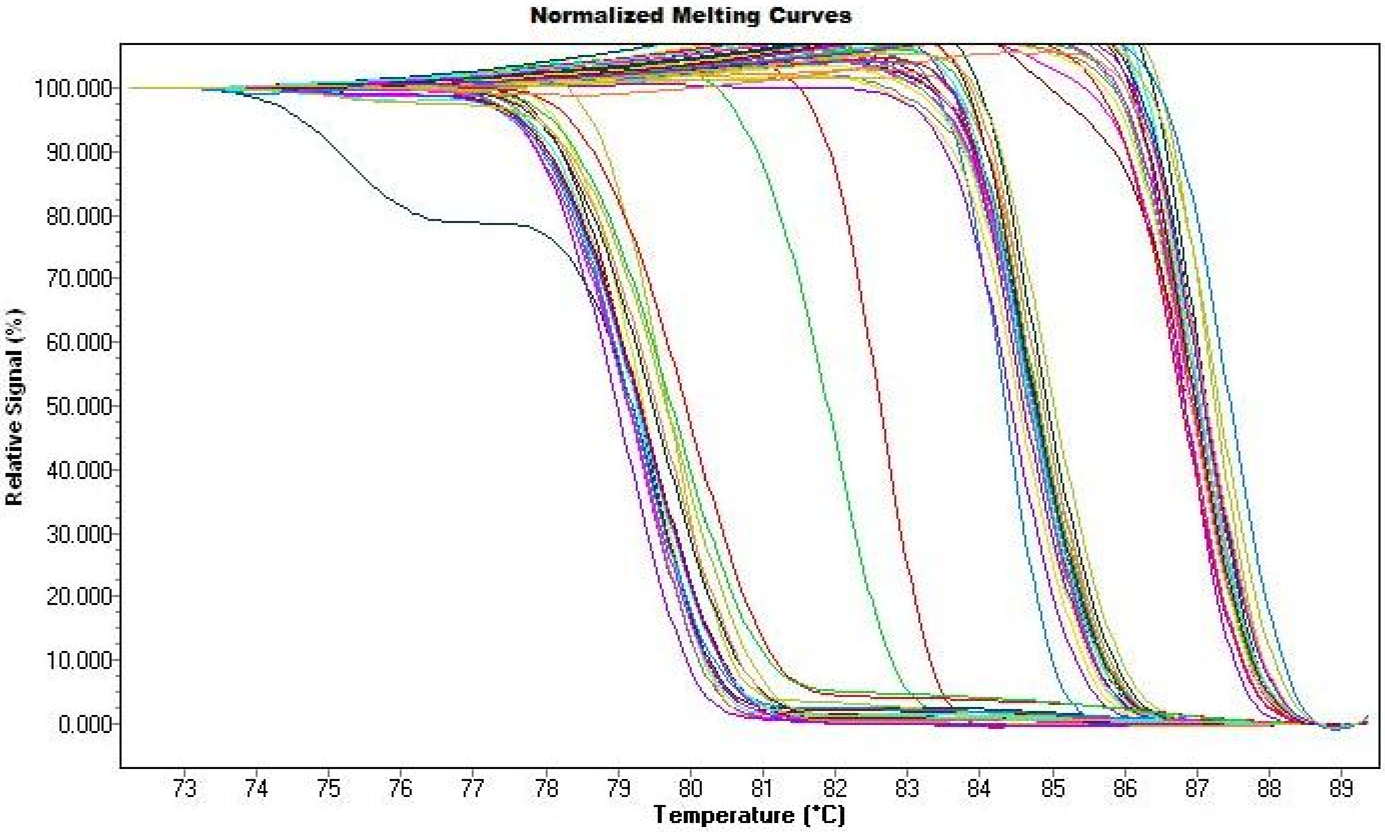
Normalized Melting Curves
Relative Signal (%)
100.000
90.000
80.000
70.000
60.000
50.000
40.000
30.000
20.000
10.000
0.000
73
74
75
76
77
78
79
80
81
82
83
84
85
86
87
88
89
Temperature (°C)

Normalized and Shifted Melting Curves

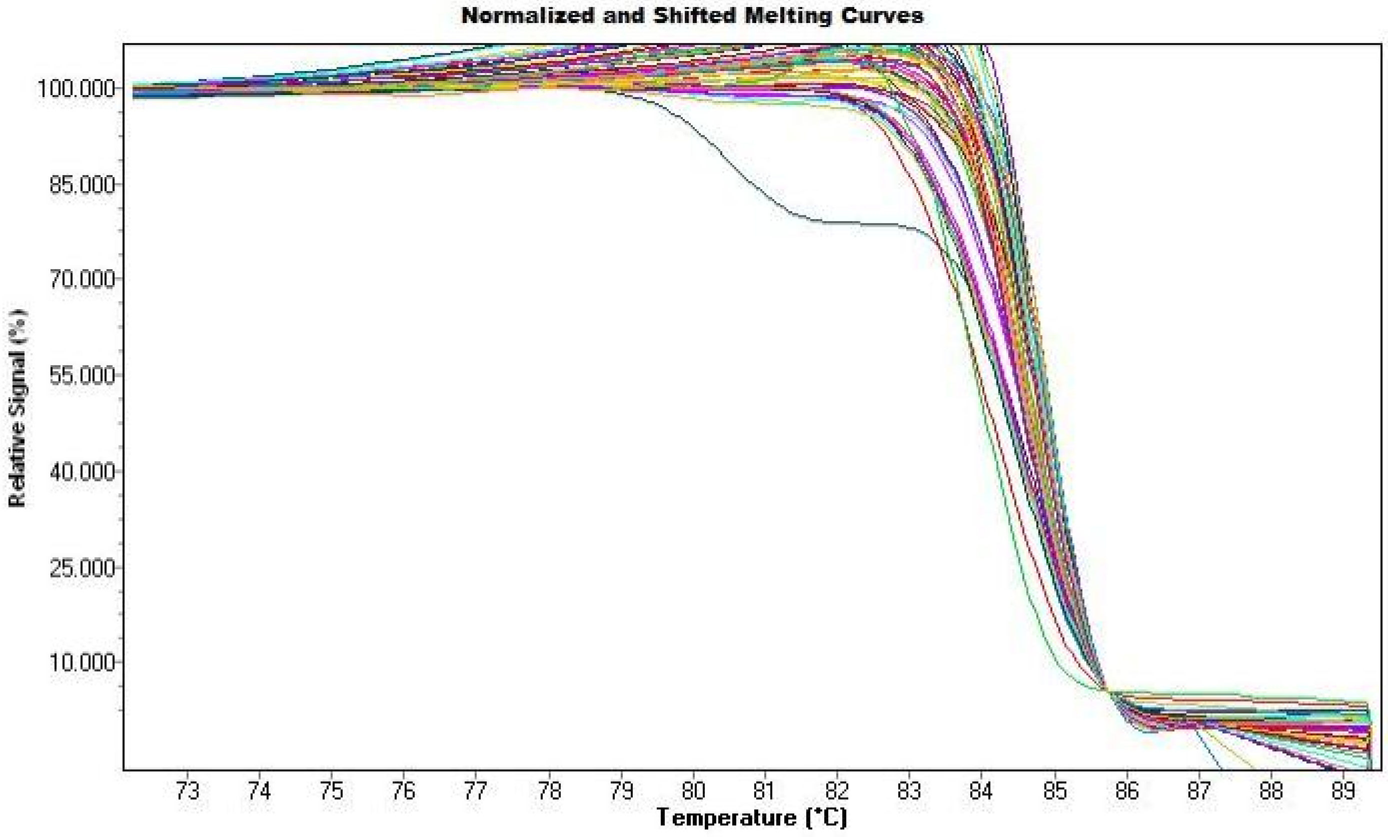

Normalized and Temp-Shifted Difference Plot

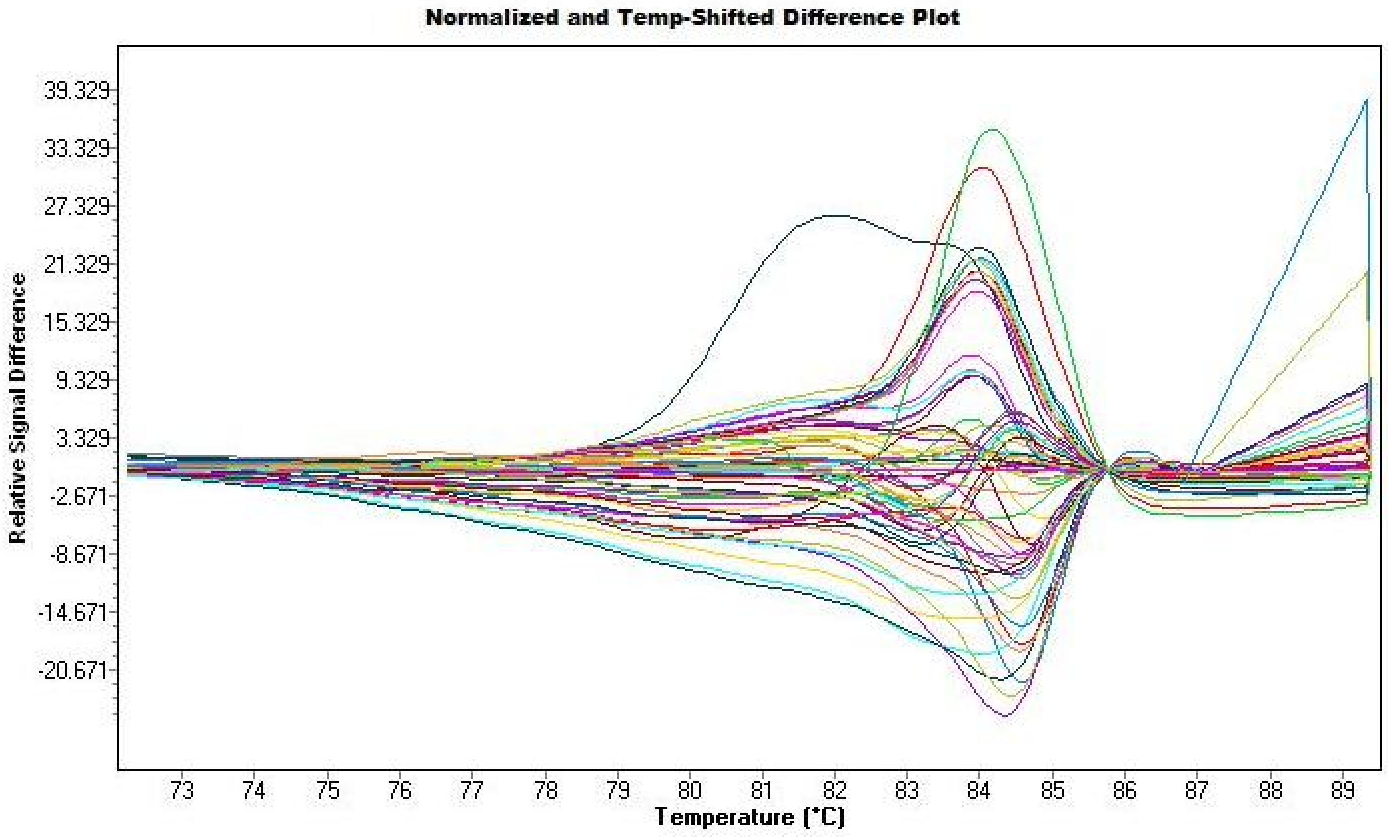

Analysis Notes	

Melt Curve Genotyping for All Samples (Melt Curve Genotyping)

Channel	465-510	Color Compensation	Off				
Progam Name	Program						
Stds Settings	Auto-Group						
Sensitivity	Normal	Temp Range	63-86	Score	0.7	Res	0.1

Subset Name	All Samples

Results

Inc	Pos.	Sample Name	Sample Type	Group	Score	Resolution	Status
☑	A1	GAPDH 102	Unknown	4	0.99	0.28	
☑	A2	GAPDH 102	Unknown	4	1.00	0.56	
☑	A3	GAPDH 102	Unknown	4	0.98	0.42	
☑	A4	GAPDH 107	Unknown	1	0.93	0.47	
☑	A5	GAPDH 107	Unknown	1	0.93	0.47	
☑	A6	GAPDH 107	Unknown	1	0.93	0.47	
☑	A7	GAPDH 110	Unknown	1	0.94	0.46	
☑	A8	GAPDH 110	Unknown	1	0.95	0.45	
☑	A9	GAPDH 110	Unknown	1	0.96	0.45	
☑	A10	GAPDH 113	Unknown	1	0.95	0.44	
☑	A11	GAPDH 113	Unknown	1	0.95	0.44	
☑	A12	GAPDH 113	Unknown	1	0.95	0.44	
☑	B1	FCER1A 102	Unknown	3	0.98	0.87	
☑	B2	FCER1A 102	Unknown	3	0.99	0.89	
☑	B3	FCER1A 102	Unknown	3	1.00	0.89	
☑	B4	FCER1A 107	Unknown	2	0.99	0.41	
☑	B5	FCER1A 107	Unknown	2	0.99	0.42	
☑	B6	FCER1A 107	Unknown	2	0.82	0.15	
☑	B7	FCER1A 110	Unknown	2	0.96	0.40	
☑	B8	FCER1A 110	Unknown	2	0.95	0.32	
☑	B9	FCER1A 110	Unknown	5	0.81	0.19	
☑	B10	FCER1A 113	Unknown	5	1.00	0.55	
☑	B11	FCER1A 113	Unknown	2	0.98	0.30	
☑	B12	FCER1A 113	Unknown	Unknown	0.80	0.07	
☑	C1	RGS1 102	Unknown	3	0.89	0.73	
☑	C2	RGS1 102	Unknown	3	0.89	0.75	
☑	C3	RGS1 102	Unknown	6	1.00	0.80	
☑	C4	RGS1 107	Unknown	1	0.99	0.55	
☑	C5	RGS1 107	Unknown	1	0.99	0.59	
☑	C6	RGS1 107	Unknown	1	0.99	0.61	
☑	C7	RGS1 110	Unknown	1	0.96	0.55	

Results

Inc	Pos.	Sample Name	Sample Type	Group	Score	Resolution	Status
☑	C8	RGS1 110	Unknown	1	0.99	0.60	
☑	C9	RGS1 110	Unknown	1	0.98	0.62	
☑	C10	RGS1 113	Unknown	1	0.98	0.52	
☑	C11	RGS1 113	Unknown	1	0.99	0.41	
☑	C12	RGS1 113	Unknown	1	0.98	0.60	
☑	D1	GAPDH 115	Unknown	1	0.94	0.47	
☑	D2	GAPDH 115	Unknown	1	0.93	0.48	
☑	D3	GAPDH 115	Unknown	1	0.93	0.48	
☑	D4	GAPDH 117	Unknown	1	0.91	0.49	
☑	D5	GAPDH 117	Unknown	1	0.93	0.46	
☑	D6	GAPDH 117	Unknown	1	0.92	0.47	
☑	D7	GAPDH 119	Unknown	1	0.91	0.49	
☑	D8	GAPDH 119	Unknown	1	0.94	0.46	
☑	D9	GAPDH 119	Unknown	1	0.93	0.47	
☑	D10	GAPDH 120	Unknown	1	0.94	0.46	
☑	D11	GAPDH 120	Unknown	1	0.92	0.47	
☑	D12	GAPDH 120	Unknown	1	0.93	0.48	
☑	E1	FCER1A 115	Unknown	2	0.99	0.48	
☑	E2	FCER1A 115	Unknown	2	0.97	0.42	
☑	E3	FCER1A 115	Unknown	2	0.97	0.46	
☑	E4	FCER1A 117	Unknown	2	0.99	0.47	
☑	E5	FCER1A 117	Unknown	2	0.99	0.53	
☑	E6	FCER1A 117	Unknown	2	1.00	0.55	
☑	E7	FCER1A 119	Unknown	2	0.99	0.52	
☑	E8	FCER1A 119	Unknown	2	0.99	0.52	
☑	E9	FCER1A 119	Unknown	2	0.99	0.57	
☑	E10	FCER1A 120	Unknown	2	0.99	0.58	
☑	E11	FCER1A 120	Unknown	2	0.99	0.53	
☑	E12	FCER1A 120	Unknown	2	0.99	0.53	
☑	F1	RGS1 115	Unknown	1	0.99	0.62	
☑	F2	RGS1 115	Unknown	1	0.99	0.52	
☑	F3	RGS1 115	Unknown	1	0.99	0.65	
☑	F4	RGS1 117	Unknown	1	0.99	0.58	
☑	F5	RGS1 117	Unknown	1	0.99	0.65	
☑	F6	RGS1 117	Unknown	1	0.99	0.62	
☑	F7	RGS1 119	Unknown	1	0.99	0.70	
☑	F8	RGS1 119	Unknown	1	0.99	0.66	
☑	F9	RGS1 119	Unknown	1	1.00	0.66	

Results

Inc	Pos.	Sample Name	Sample Type	Group	Score	Resolution	Status
☑	F10	RGS1 120	Unknown	1	0.99	0.61	
☑	F11	RGS1 120	Unknown	1	0.99	0.64	
☑	F12	RGS1 120	Unknown	1	0.99	0.63	
☑	G1		Unknown	Negative	1.00	1.00	
☑	G2		Unknown	Negative	1.00	1.00	
☑	G3		Unknown	Negative	1.00	1.00	
☑	G4		Unknown	Negative	1.00	1.00	
☑	G5		Unknown	Negative	1.00	1.00	
☑	G6		Unknown	Negative	1.00	1.00	
☑	G7		Unknown	Negative	1.00	1.00	
☑	G8		Unknown	Negative	1.00	1.00	
☑	G9		Unknown	Negative	1.00	1.00	
☑	G10		Unknown	Negative	1.00	1.00	
☑	G11		Unknown	Negative	1.00	1.00	
☑	G12		Unknown	Negative	1.00	1.00	
☑	H1		Unknown	Negative	1.00	1.00	
☑	H2		Unknown	Negative	1.00	1.00	
☑	H3		Unknown	Negative	1.00	1.00	
☑	H4		Unknown	Negative	1.00	1.00	
☑	H5		Unknown	Negative	1.00	1.00	
☑	H6		Unknown	Negative	1.00	1.00	
☑	H7		Unknown	Negative	1.00	1.00	
☑	H8		Unknown	Negative	1.00	1.00	
☑	H9		Unknown	Negative	1.00	1.00	
☑	H10		Unknown	Negative	1.00	1.00	
☑	H11		Unknown	Negative	1.00	1.00	
☑	H12		Unknown	Negative	1.00	1.00	

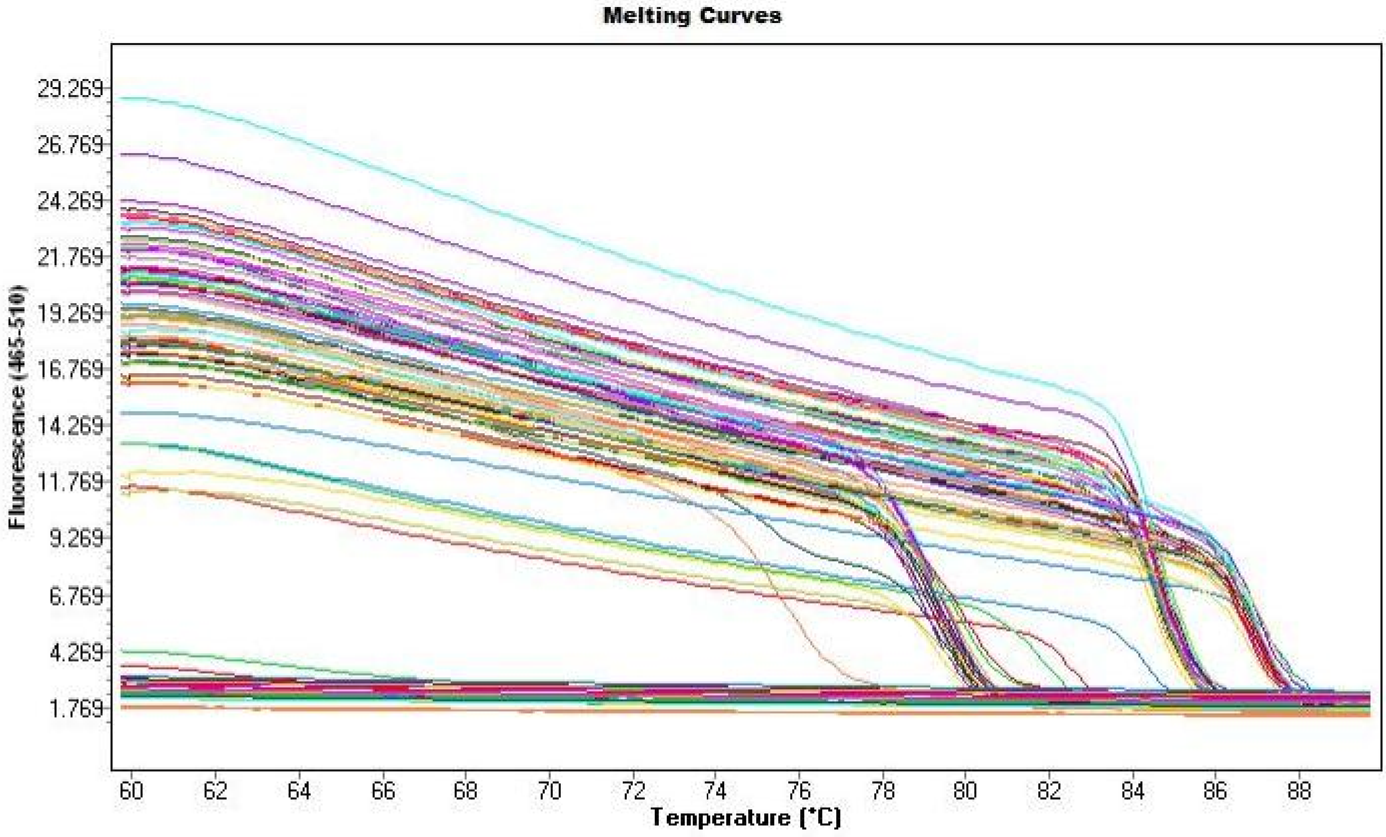

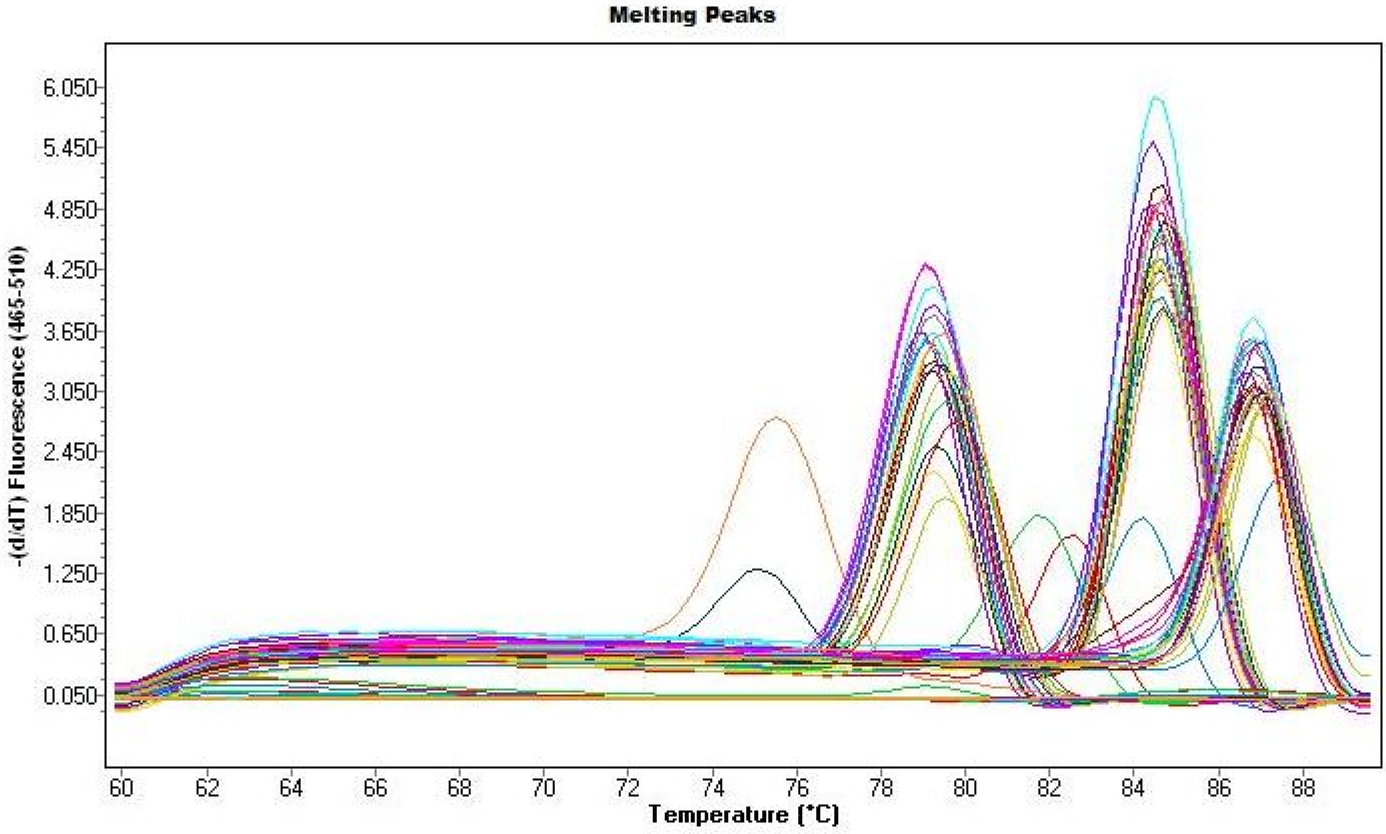

Analysis Notes	

Tm Calling for All Samples (Tm Calling)

Settings

Channel	465-510	Color Compensation	Off		
Program	Program				
Show Shoulders	False	Chemistry	HybProbe	Max Peaks	2

Subset Name	All Samples

Results

			Peak 1				Peak 2				
Inc	Pos	Sample Name	Tm	Area	Width	Height	Tm	Area	Width	Height	Status
☑	A1	GAPDH 102	84.31	4.40	2.52	1.75					
☑	A2	GAPDH 102	82.60	4.01	2.45	1.64					
☑	A3	GAPDH 102	81.80	5.11	2.71	1.89					
☑	A4	GAPDH 107	84.68	11.30	2.39	4.72					
☑	A5	GAPDH 107	84.76	9.61	2.22	4.33					
☑	A6	GAPDH 107	84.57	9.80	2.35	4.17					
☑	A7	GAPDH 110	84.67	10.47	2.38	4.40					
☑	A8	GAPDH 110	84.60	11.70	2.43	4.81					
☑	A9	GAPDH 110	84.75	9.62	2.39	4.03					
☑	A10	GAPDH 113	84.86	9.22	2.32	3.98					
☑	A11	GAPDH 113	84.47	10.94	2.14	5.11					
☑	A12	GAPDH 113	84.83	9.61	2.15	4.46					
☑	B1	FCER1A 102	71.97	0.17	8.89	0.02					
☑	B2	FCER1A 102	70.47	0.23	8.27	0.03					
☑	B3	FCER1A 102	69.22	0.26	6.65	0.04					
☑	B4	FCER1A 107	79.15	10.72	2.40	4.46					
☑	B5	FCER1A 107	79.14	8.97	2.43	3.70					
☑	B6	FCER1A 107	79.26	5.58	2.36	2.36					
☑	B7	FCER1A 110	79.25	7.75	2.32	3.34					
☑	B8	FCER1A 110	79.19	9.17	2.49	3.68					
☑	B9	FCER1A 110	75.05	2.21	2.25	0.98	79.40	6.02	2.35	2.56	
☑	B10	FCER1A 113	75.58	8.91	3.24	2.75					
☑	B11	FCER1A 113	78.92	9.65	2.56	3.77					
☑	B12	FCER1A 113	79.65	4.29	2.09	2.05					
☑	C1	RGS1 102	66.10	0.45	5.08	0.09					
☑	C2	RGS1 102	66.07	0.67	5.33	0.13					
☑	C3	RGS1 102									
☑	C4	RGS1 107									
☑	C5	RGS1 107									

Results

Inc	Pos	Sample Name	Peak 1 Tm	Area	Width	Height	Peak 2 Tm	Area	Width	Height	Status
☑	C6	RGS1 107									
☑	C7	RGS1 110									
☑	C8	RGS1 110									
☑	C9	RGS1 110									
☑	C10	RGS1 113									
☑	C11	RGS1 113									
☑	C12	RGS1 113									
☑	D1	GAPDH 115	84.73	9.47	2.32	4.08					
☑	D2	GAPDH 115	84.66	11.41	2.26	5.04					
☑	D3	GAPDH 115	84.78	10.95	2.29	4.78					
☑	D4	GAPDH 117	84.68	12.38	2.43	5.10					
☑	D5	GAPDH 117	84.67	10.94	2.41	4.53					
☑	D6	GAPDH 117	84.61	10.70	2.42	4.43					
☑	D7	GAPDH 119	84.71	11.97	2.24	5.36					
☑	D8	GAPDH 119	84.67	14.30	2.32	6.17					
☑	D9	GAPDH 119	84.87	11.37	2.32	4.91					
☑	D10	GAPDH 120	84.84	12.00	2.33	5.15					
☑	D11	GAPDH 120	84.55	13.28	2.36	5.64					
☑	D12	GAPDH 120	85.00	11.37	2.32	4.90					
☑	E1	FCER1A 115	79.16	9.24	2.54	3.63					
☑	E2	FCER1A 115	79.90	7.56	2.67	2.83					
☑	E3	FCER1A 115	79.69	8.30	2.72	3.05					
☑	E4	FCER1A 117	79.15	11.31	2.55	4.44					
☑	E5	FCER1A 117	79.29	10.31	2.62	3.94					
☑	E6	FCER1A 117	79.34	9.34	2.58	3.62					
☑	E7	FCER1A 119	79.32	9.00	2.59	3.47					
☑	E8	FCER1A 119	79.27	11.01	2.61	4.21					
☑	E9	FCER1A 119	79.47	9.15	2.66	3.44					
☑	E10	FCER1A 120	79.53	9.99	2.66	3.76					
☑	E11	FCER1A 120	79.31	10.71	2.63	4.07					
☑	E12	FCER1A 120	79.69	8.97	2.66	3.37					
☑	F1	RGS1 115									
☑	F2	RGS1 115									
☑	F3	RGS1 115									
☑	F4	RGS1 117									
☑	F5	RGS1 117									

Results

Inc	Pos	Sample Name	Peak 1				Peak 2				Status
			Tm	Area	Width	Height	Tm	Area	Width	Height	
☑	F6	RGS1 117									
☑	F7	RGS1 119									
☑	F8	RGS1 119									
☑	F9	RGS1 119									
☑	F10	RGS1 120									
☑	F11	RGS1 120									
☑	F12	RGS1 120									
☑	G1										
☑	G2										
☑	G3										
☑	G4										
☑	G5										
☑	G6										
☑	G7										
☑	G8										
☑	G9										
☑	G10										
☑	G11										
☑	G12										
☑	H1										
☑	H2										
☑	H3										
☑	H4										
☑	H5										
☑	H6										
☑	H7										
☑	H8										
☑	H9										
☑	H10										
☑	H11										
☑	H12										

Melting Curves

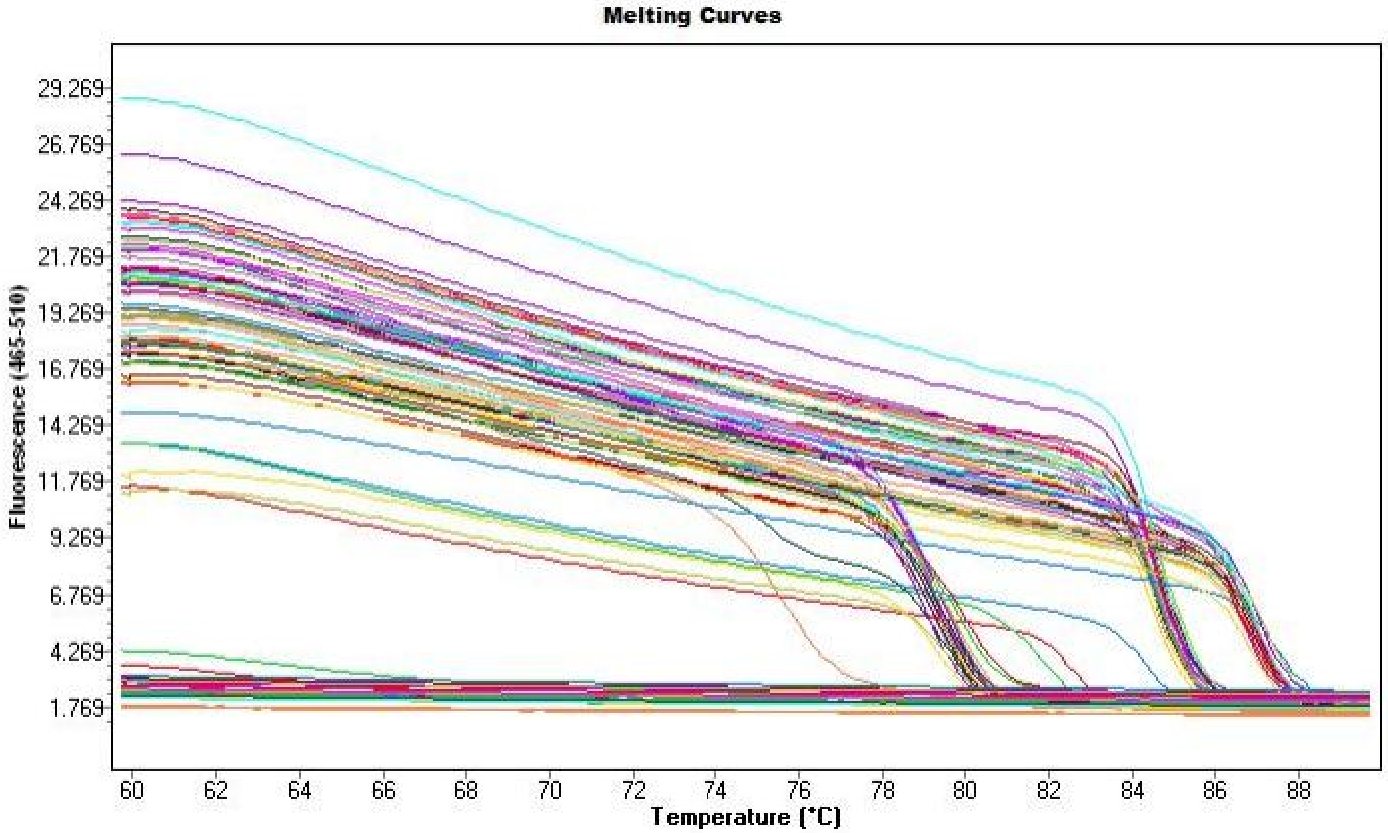

Melting Peaks

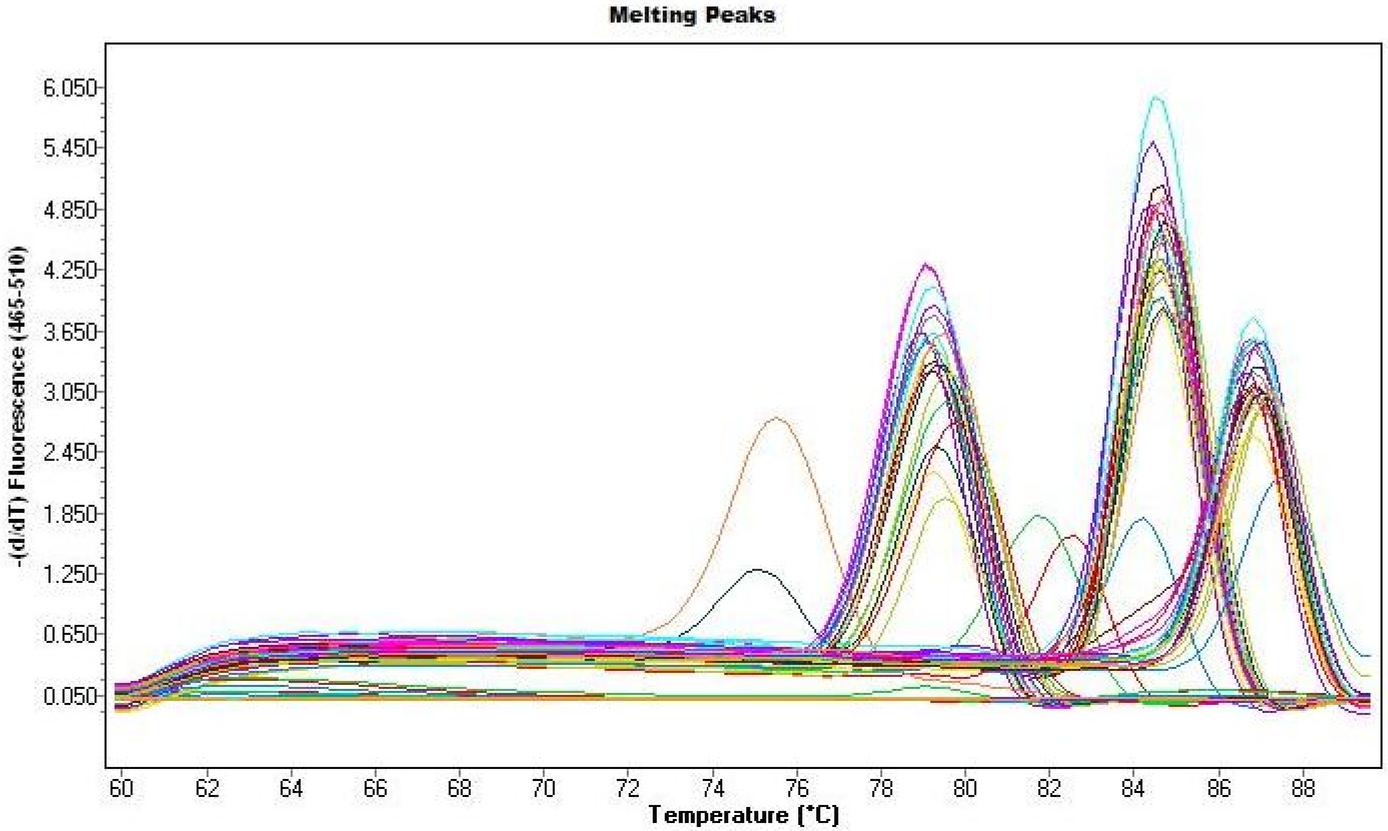

Analysis Notes	
