# Supplementary material for: Exploring differential gene expression and biomarker potential in systemic lupus erythematosus: a retrospective study
Source: PeerJ. 2025 Sep 8;13:e19891. doi: 10.7717/peerj.19891 (PMC12424612; doi:10.7717/peerj.19891)
Supplement: Supplemental Information 1 — The data include the Ct value for each sample, the Ct value for reference genes such as GAPDH, and the relative expression levels calculated using the ΔΔCt method. These data were used to validate the RNA sequencing results and further evaluate the potential of these genes as biomarkers for SLE. [file peerj-13-19891-s001.zip › qPCR/20241222 qPCR/1222plate1 SLE group.pdf]

cobas® 4800 User Defined Workflow (UDF)

20241222xj plate1

Experiment

Creation Date	12/22/2024 4:13:15 PM	Last Modified Date	12/22/2024 5:24:59 PM
Operator	System Admin	Owner	System Admin
Start Time	12/22/2024 4:21:50 PM	End Time	12/22/2024 5:20:24 PM
Run State	Completed	Software Version	LCS480 1.5.1.62 SP3 - UDF 2.1.0.26
Macro		Macro Owner	
Macro Status			
Templates	gongying	Plate ID	
Test ID		Lot ID	
Color Comp ID			
Run Notes			

Programs

Program Name	Program		
Cycles	1	Analysis Mode	None

Target (°C)	Acquisition Mode	Hold (hh:mm:ss)	Ramp Rate (°C/s)	Acquisitions (per °C)	Sec Target (°C)	Step size (°C)	Step Delay (cycles)
95	None	00:00:30	4.40		0	0	0

Program Name	Program		
Cycles	40	Analysis Mode	Quantification

Target (°C)	Acquisition Mode	Hold (hh:mm:ss)	Ramp Rate (°C/s)	Acquisitions (per °C)	Sec Target (°C)	Step size (°C)	Step Delay (cycles)
95	None	00:00:15	4.40		0	0	0
60	Single	00:00:30	2.20		0	0	0

Program Name	Program		
Cycles	1	Analysis Mode	Melting Curves

Target (°C)	Acquisition Mode	Hold (hh:mm:ss)	Ramp Rate (°C/s)	Acquisitions (per °C)	Sec Target (°C)	Step size (°C)	Step Delay (cycles)
95	None	00:01:00	4.40		0	0	0
60	None	00:00:30	2.20		0	0	0
90	Continuous		0.11	5	0	0	0

Program Name	Program		
Cycles	1	Analysis Mode	None

Target (°C)	Acquisition Mode	Hold (hh:mm:ss)	Ramp Rate (°C/s)	Acquisitions (per °C)	Sec Target (°C)	Step size (°C)	Step Delay (cycles)

Target (°C)	Acquisition Mode	Hold (hh:mm:ss)	Ramp Rate (°C/s)	Acquisitions (per °C)	Sec Target (°C)	Step size (°C)	Step Delay (cycles)
40	None	00:00:30	2.20		0	0	0

Samples

Sample Count	96
Subset	All Samples

Pos	Name	ID	Repl. Of	Sample Notes
A1	GAPDH 1			
A2	GAPDH 1			
A3	GAPDH 1			
A4	GAPDH 2			
A5	GAPDH 2			
A6	GAPDH 2			
A7	GAPDH 3			
A8	GAPDH 3			
A9	GAPDH 3			
A10	GAPDH 4			
A11	GAPDH 4			
A12	GAPDH 4			
B1	FCER1A 1			
B2	FCER1A 1			
B3	FCER1A 1			
B4	FCER1A 2			
B5	FCER1A 2			
B6	FCER1A 2			
B7	FCER1A 3			
B8	FCER1A 3			
B9	FCER1A 3			
B10	FCER1A 4			
B11	FCER1A 4			
B12	FCER1A 4			
C1	GAPDH 5			
C2	GAPDH 5			
C3	GAPDH 5			
C4	GAPDH 7			
C5	GAPDH 7			
C6	GAPDH 7			
C7	GAPDH 11			
C8	GAPDH 11			
C9	GAPDH 11			
C10	GAPDH 14			
C11	GAPDH 14			
C12	GAPDH 14			

Samples

Sample Count	96
Subset	All Samples

Pos	Name	ID	Repl. Of	Sample Notes
D1	FCER1A 5			
D2	FCER1A 5			
D3	FCER1A 5			
D4	FCER1A 7			
D5	FCER1A 7			
D6	FCER1A 7			
D7	FCER1A 11			
D8	FCER1A 11			
D9	FCER1A 11			
D10	FCER1A 14			
D11	FCER1A 14			
D12	FCER1A 14			
E1	GAPDH 15			
E2	GAPDH 15			
E3	GAPDH 15			
E4	GAPDH 18			
E5	GAPDH 18			
E6	GAPDH 18			
E7	GAPDH 19			
E8	GAPDH 19			
E9	GAPDH 19			
E10	GAPDH 20			
E11	GAPDH 20			
E12	GAPDH 20			
F1	FCER1A 15			
F2	FCER1A 15			
F3	FCER1A 15			
F4	FCER1A 18			
F5	FCER1A 18			
F6	FCER1A 18			
F7	FCER1A 19			
F8	FCER1A 19			
F9	FCER1A 19			
F10	FCER1A 20			
F11	FCER1A 20			
F12	FCER1A 20			
G1	GAPDH 24			
G2	GAPDH 24			
G3	GAPDH 24			

Samples

Sample Count	96
Subset	All Samples

Pos	Name	ID	Repl. Of	Sample Notes
G4	GAPDH 26			
G5	GAPDH 26			
G6	GAPDH 26			
G7				
G8				
G9				
G10				
G11				
G12				
H1	FCER1A 24			
H2	FCER1A 24			
H3	FCER1A 24			
H4	FCER1A 26			
H5	FCER1A 26			
H6	FCER1A 26			
H7				
H8				
H9				
H10				
H11				
H12				

Instrument

Name	Z480	Serial Number	56319

Revision History

Revision	Date	User	Reason

Abs Quant/2nd Derivative Max for All Samples (Abs Quant/2nd Derivative Max)

Settings

Channel	465-510		
Color Compensation	Off		
Program	Program	Units	
Mode	High Confidence		

Subset Name	All Samples

Results

> -Inte	CPosal	N(lasmtive cycles) has higher uncertainty	Type	CP	Concentration	Standard	Status

Results

Inc	Pos	Name	Type	CP	Concentration	Standard	Status
☑	A1	GAPDH 1	Unknown	26.99			
☑	A2	GAPDH 1	Unknown	27.00			
☑	A3	GAPDH 1	Unknown	26.92			
☑	A4	GAPDH 2	Unknown	30.88			
☑	A5	GAPDH 2	Unknown	28.54			
☑	A6	GAPDH 2	Unknown	31.58			
☑	A7	GAPDH 3	Unknown	21.53			
☑	A8	GAPDH 3	Unknown	21.33			
☑	A9	GAPDH 3	Unknown	21.59			
☑	A10	GAPDH 4	Unknown	31.85			
☑	A11	GAPDH 4	Unknown	32.06			
☑	A12	GAPDH 4	Unknown	24.61			
☑	B1	FCER1A 1	Unknown	33.73			
☑	B2	FCER1A 1	Unknown				
☑	B3	FCER1A 1	Unknown				
☑	B4	FCER1A 2	Unknown				
☑	B5	FCER1A 2	Unknown	31.89			
☑	B6	FCER1A 2	Unknown	33.00			
☑	B7	FCER1A 3	Unknown	28.30			
☑	B8	FCER1A 3	Unknown	28.24			
☑	B9	FCER1A 3	Unknown	28.11			
☑	B10	FCER1A 4	Unknown	32.36			
☑	B11	FCER1A 4	Unknown	31.51			
☑	B12	FCER1A 4	Unknown	31.62			
☑	C1	GAPDH 5	Unknown	26.27			
☑	C2	GAPDH 5	Unknown	25.29			
☑	C3	GAPDH 5	Unknown	26.26			
☑	C4	GAPDH 7	Unknown	23.09			
☑	C5	GAPDH 7	Unknown	23.88			
☑	C6	GAPDH 7	Unknown	23.99			
☑	C7	GAPDH 11	Unknown	31.24			
☑	C8	GAPDH 11	Unknown	31.46			
☑	C9	GAPDH 11	Unknown	31.24			
☑	C10	GAPDH 14	Unknown	23.89			
☑	C11	GAPDH 14	Unknown	23.51			
☑	C12	GAPDH 14	Unknown	24.00			
☑	D1	FCER1A 5	Unknown	32.99			

> - Late Cp call (last five cycles) has higher uncertainty

Results

Inc	Pos	Name	Type	CP	Concentration	Standard	Status
☑	D2	FCER1A 5	Unknown	31.88			
☑	D3	FCER1A 5	Unknown	32.41			
☑	D4	FCER1A 7	Unknown	31.12			
☑	D5	FCER1A 7	Unknown	31.92			
☑	D6	FCER1A 7	Unknown	31.28			
☑	D7	FCER1A 11	Unknown	31.55			
☑	D8	FCER1A 11	Unknown	35.00			>
☑	D9	FCER1A 11	Unknown	33.80			
☑	D10	FCER1A 14	Unknown	32.30			
☑	D11	FCER1A 14	Unknown	31.67			
☑	D12	FCER1A 14	Unknown	31.21			
☑	E1	GAPDH 15	Unknown	22.94			
☑	E2	GAPDH 15	Unknown	22.68			
☑	E3	GAPDH 15	Unknown	22.51			
☑	E4	GAPDH 18	Unknown	23.62			
☑	E5	GAPDH 18	Unknown	23.66			
☑	E6	GAPDH 18	Unknown	23.62			
☑	E7	GAPDH 19	Unknown	22.90			
☑	E8	GAPDH 19	Unknown	23.00			
☑	E9	GAPDH 19	Unknown	24.31			
☑	E10	GAPDH 20	Unknown	22.95			
☑	E11	GAPDH 20	Unknown	22.85			
☑	E12	GAPDH 20	Unknown	22.86			
☑	F1	FCER1A 15	Unknown	32.29			
☑	F2	FCER1A 15	Unknown	31.06			
☑	F3	FCER1A 15	Unknown	31.63			
☑	F4	FCER1A 18	Unknown	29.90			
☑	F5	FCER1A 18	Unknown	30.62			
☑	F6	FCER1A 18	Unknown	31.15			
☑	F7	FCER1A 19	Unknown	29.75			
☑	F8	FCER1A 19	Unknown	29.98			
☑	F9	FCER1A 19	Unknown	30.63			
☑	F10	FCER1A 20	Unknown	30.12			
☑	F11	FCER1A 20	Unknown	31.40			
☑	F12	FCER1A 20	Unknown	30.90			
☑	G1	GAPDH 24	Unknown	21.98			
☑	G2	GAPDH 24	Unknown	21.79			

> - Late Cp call (last five cycles) has higher uncertainty

Results

Inc	Pos	Name	Type	CP	Concentration	Standard	Status
☑	G3	GAPDH 24	Unknown	21.94			
☑	G4	GAPDH 26	Unknown	23.57			
☑	G5	GAPDH 26	Unknown	23.78			
☑	G6	GAPDH 26	Unknown	23.78			
☑	G7		Unknown				
☑	G8		Unknown				
☑	G9		Unknown				
☑	G10		Unknown				
☑	G11		Unknown				
☑	G12		Unknown				
☑	H1	FCER1A 24	Unknown	31.74			
☑	H2	FCER1A 24	Unknown	31.81			
☑	H3	FCER1A 24	Unknown	28.46			
☑	H4	FCER1A 26	Unknown	32.63			
☑	H5	FCER1A 26	Unknown	32.42			
☑	H6	FCER1A 26	Unknown	32.34			
☑	H7		Unknown				
☑	H8		Unknown				
☑	H9		Unknown				
☑	H10		Unknown				
☑	H11		Unknown				
☑	H12		Unknown				

> - Late Cp call (last five cycles) has higher uncertainty

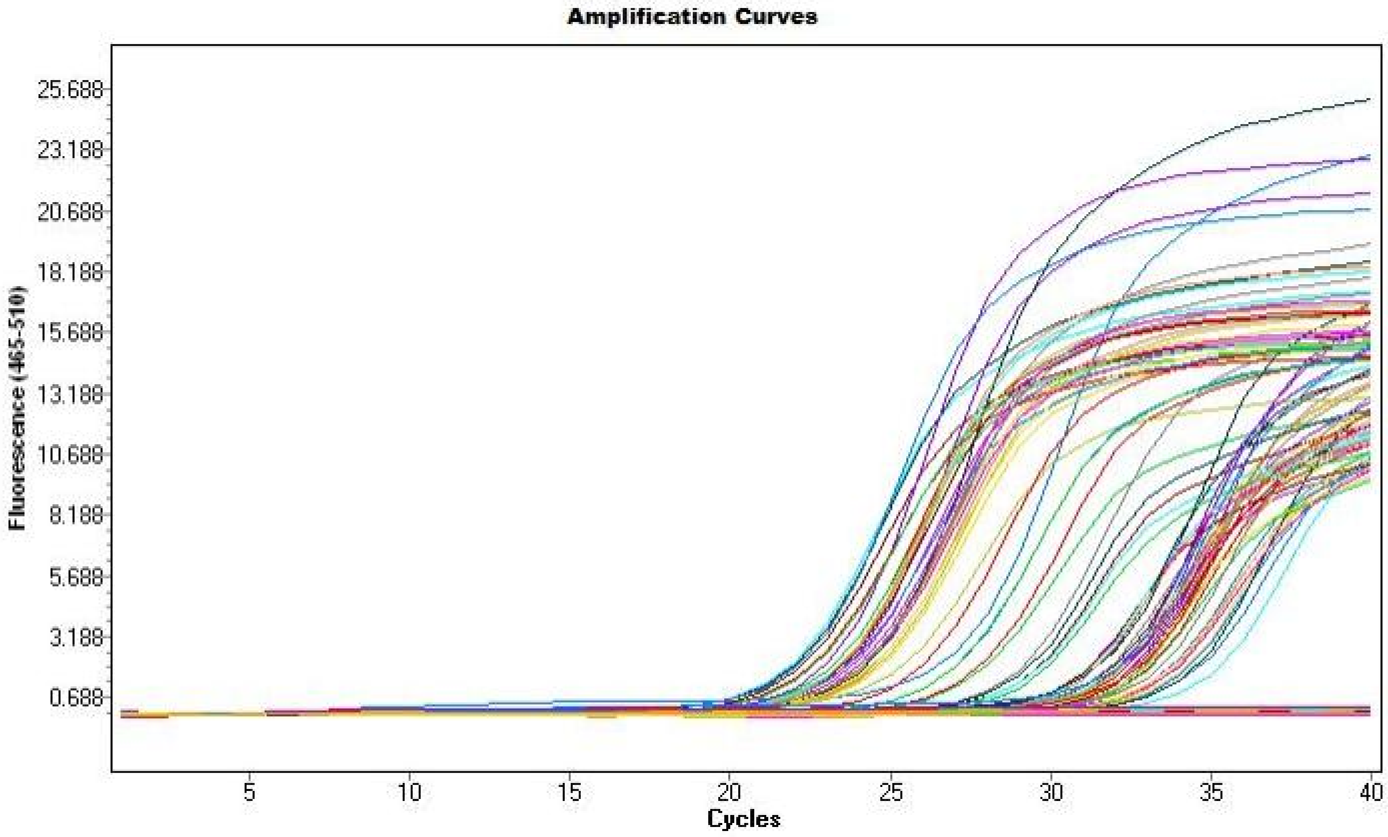

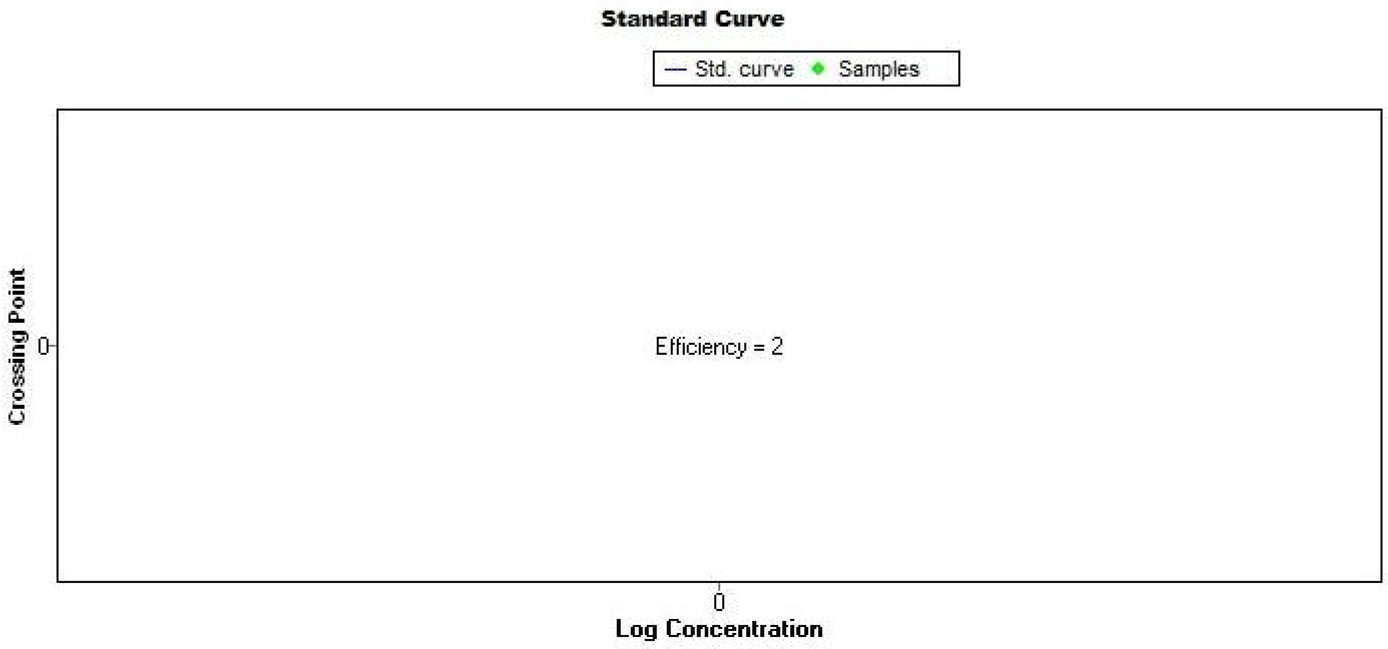

Analysis Notes	

Abs Quant/2nd Derivative Max for All Samples (1) (Abs Quant/2nd Derivative Max)

Settings

Channel	465-510		
Color Compensation	Off		
Program	Program	Units	
Mode	High Confidence		

Subset Name	All Samples

Results

Inc	Pos	Name	Type	CP	Concentration	Standard	Status
☑	A1	GAPDH 1	Unknown				
☑	A2	GAPDH 1	Unknown				
☑	A3	GAPDH 1	Unknown				
☑	A4	GAPDH 2	Unknown				
☑	A5	GAPDH 2	Unknown				
☑	A6	GAPDH 2	Unknown				
☑	A7	GAPDH 3	Unknown				
☑	A8	GAPDH 3	Unknown				
☑	A9	GAPDH 3	Unknown				
☑	A10	GAPDH 4	Unknown				
☑	A11	GAPDH 4	Unknown				
☑	A12	GAPDH 4	Unknown				
☑	B1	FCER1A 1	Unknown				
☑	B2	FCER1A 1	Unknown				
☑	B3	FCER1A 1	Unknown				
☑	B4	FCER1A 2	Unknown				
☑	B5	FCER1A 2	Unknown				
☑	B6	FCER1A 2	Unknown				
☑	B7	FCER1A 3	Unknown				
☑	B8	FCER1A 3	Unknown				
☑	B9	FCER1A 3	Unknown				
☑	B10	FCER1A 4	Unknown				
☑	B11	FCER1A 4	Unknown				
☑	B12	FCER1A 4	Unknown				
☑	C1	GAPDH 5	Unknown				
☑	C2	GAPDH 5	Unknown				
☑	C3	GAPDH 5	Unknown				
☑	C4	GAPDH 7	Unknown				
☑	C5	GAPDH 7	Unknown				
☑	C6	GAPDH 7	Unknown				

Results

Inc	Pos	Name	Type	CP	Concentration	Standard	Status
☑	C7	GAPDH 11	Unknown				
☑	C8	GAPDH 11	Unknown				
☑	C9	GAPDH 11	Unknown				
☑	C10	GAPDH 14	Unknown				
☑	C11	GAPDH 14	Unknown				
☑	C12	GAPDH 14	Unknown				
☑	D1	FCER1A 5	Unknown				
☑	D2	FCER1A 5	Unknown				
☑	D3	FCER1A 5	Unknown				
☑	D4	FCER1A 7	Unknown				
☑	D5	FCER1A 7	Unknown				
☑	D6	FCER1A 7	Unknown				
☑	D7	FCER1A 11	Unknown				
☑	D8	FCER1A 11	Unknown				
☑	D9	FCER1A 11	Unknown				
☑	D10	FCER1A 14	Unknown				
☑	D11	FCER1A 14	Unknown				
☑	D12	FCER1A 14	Unknown				
☑	E1	GAPDH 15	Unknown				
☑	E2	GAPDH 15	Unknown				
☑	E3	GAPDH 15	Unknown				
☑	E4	GAPDH 18	Unknown				
☑	E5	GAPDH 18	Unknown				
☑	E6	GAPDH 18	Unknown				
☑	E7	GAPDH 19	Unknown				
☑	E8	GAPDH 19	Unknown				
☑	E9	GAPDH 19	Unknown				
☑	E10	GAPDH 20	Unknown				
☑	E11	GAPDH 20	Unknown				
☑	E12	GAPDH 20	Unknown				
☑	F1	FCER1A 15	Unknown				
☑	F2	FCER1A 15	Unknown				
☑	F3	FCER1A 15	Unknown				
☑	F4	FCER1A 18	Unknown				
☑	F5	FCER1A 18	Unknown				
☑	F6	FCER1A 18	Unknown				
☑	F7	FCER1A 19	Unknown				

Results

Inc	Pos	Name	Type	CP	Concentration	Standard	Status
☑	F8	FCER1A 19	Unknown				
☑	F9	FCER1A 19	Unknown				
☑	F10	FCER1A 20	Unknown				
☑	F11	FCER1A 20	Unknown				
☑	F12	FCER1A 20	Unknown				
☑	G1	GAPDH 24	Unknown				
☑	G2	GAPDH 24	Unknown				
☑	G3	GAPDH 24	Unknown				
☑	G4	GAPDH 26	Unknown				
☑	G5	GAPDH 26	Unknown				
☑	G6	GAPDH 26	Unknown				
☑	G7		Unknown				
☑	G8		Unknown				
☑	G9		Unknown				
☑	G10		Unknown				
☑	G11		Unknown				
☑	G12		Unknown				
☑	H1	FCER1A 24	Unknown				
☑	H2	FCER1A 24	Unknown				
☑	H3	FCER1A 24	Unknown				
☑	H4	FCER1A 26	Unknown				
☑	H5	FCER1A 26	Unknown				
☑	H6	FCER1A 26	Unknown				
☑	H7		Unknown				
☑	H8		Unknown				
☑	H9		Unknown				
☑	H10		Unknown				
☑	H11		Unknown				
☑	H12		Unknown				

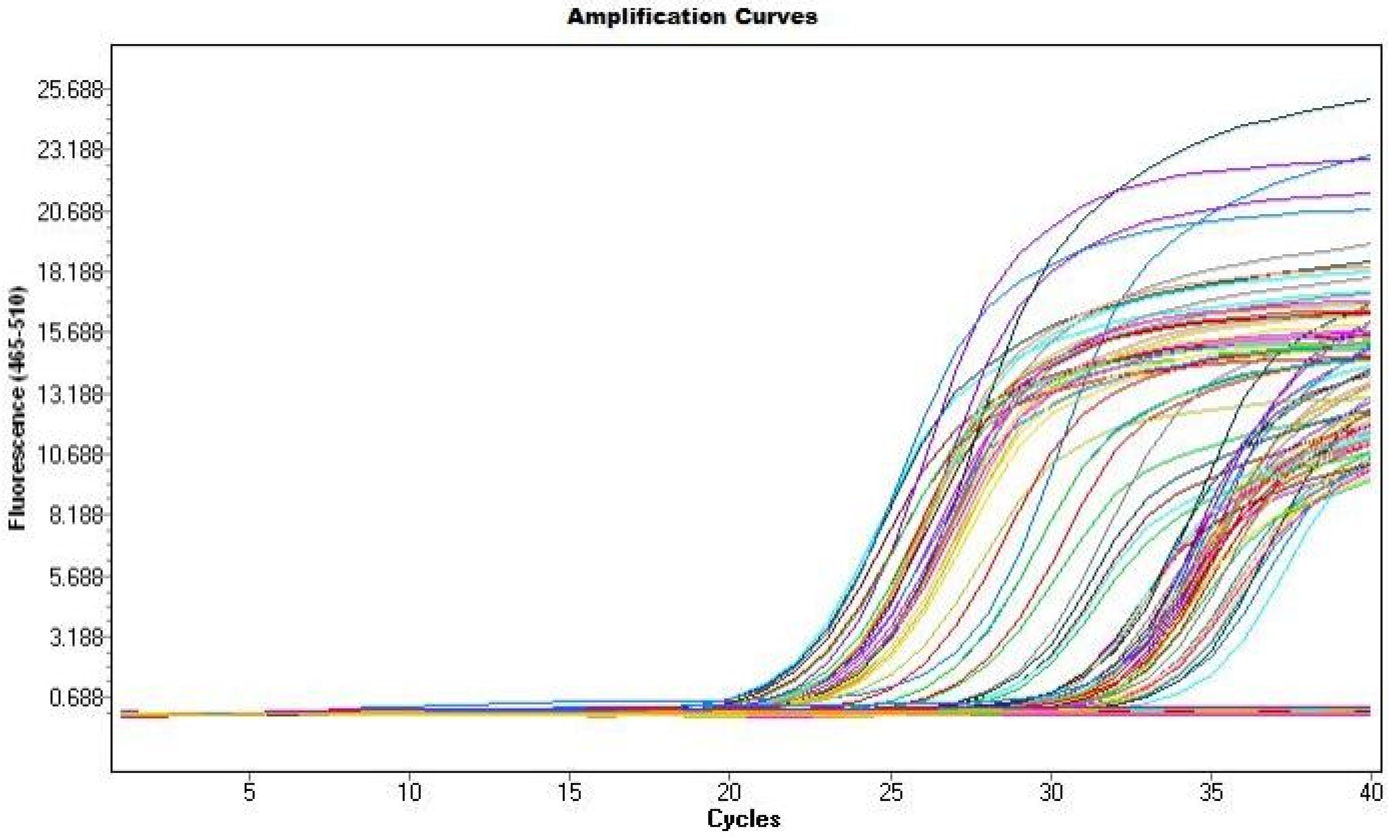

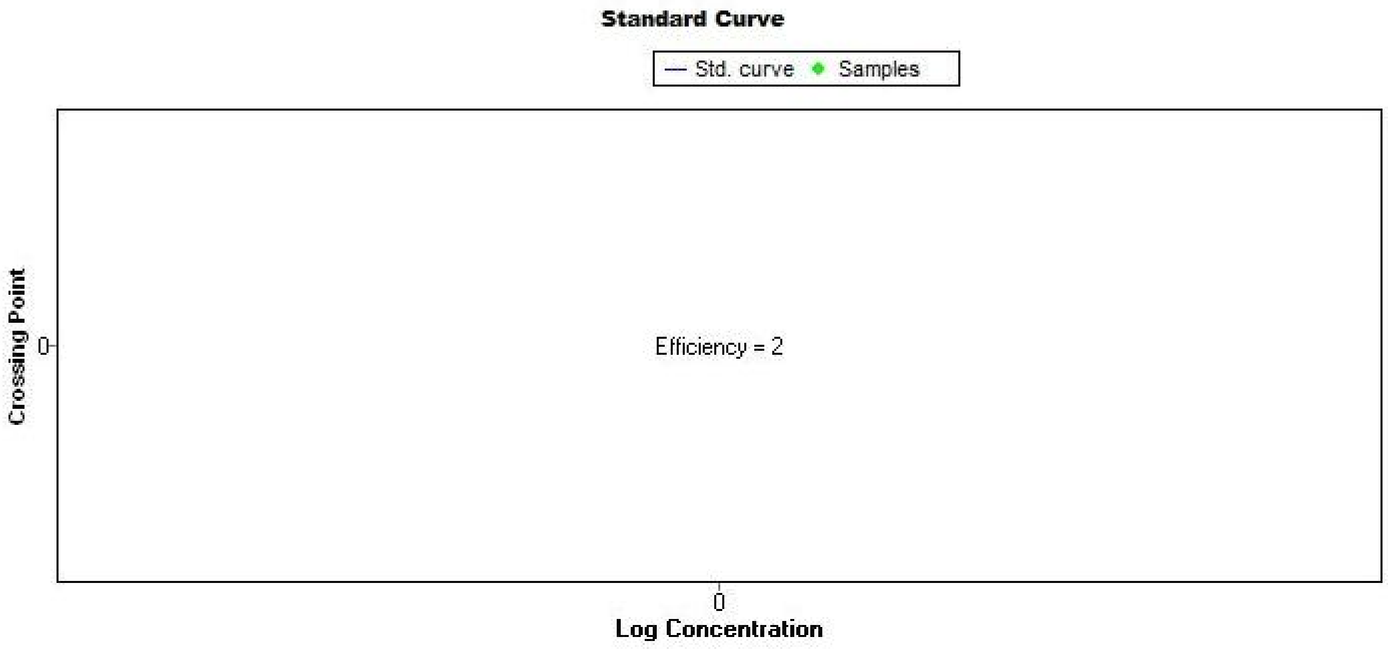

Analysis Notes	

Abs Quant/2nd Derivative Max for All Samples (2) (Abs Quant/2nd Derivative Max)

Settings

Channel	465-510		
Color Compensation	Off		
Program	Program	Units	
Mode	High Confidence		

Subset Name	All Samples

Results

Inc	Pos	Name	Type	CP	Concentration	Standard	Status
☑	A1	GAPDH 1	Unknown	26.99			
☑	A2	GAPDH 1	Unknown	27.00			
☑	A3	GAPDH 1	Unknown	26.92			
☑	A4	GAPDH 2	Unknown	30.88			
☑	A5	GAPDH 2	Unknown	28.54			
☑	A6	GAPDH 2	Unknown	31.58			
☑	A7	GAPDH 3	Unknown	21.53			
☑	A8	GAPDH 3	Unknown	21.33			
☑	A9	GAPDH 3	Unknown	21.59			
☑	A10	GAPDH 4	Unknown	31.85			
☑	A11	GAPDH 4	Unknown	32.06			
☑	A12	GAPDH 4	Unknown	24.61			
☑	B1	FCER1A 1	Unknown	33.73			
☑	B2	FCER1A 1	Unknown				
☑	B3	FCER1A 1	Unknown				
☑	B4	FCER1A 2	Unknown				
☑	B5	FCER1A 2	Unknown	31.89			
☑	B6	FCER1A 2	Unknown	33.00			
☑	B7	FCER1A 3	Unknown	28.30			
☑	B8	FCER1A 3	Unknown	28.24			
☑	B9	FCER1A 3	Unknown	28.11			
☑	B10	FCER1A 4	Unknown	32.36			
☑	B11	FCER1A 4	Unknown	31.51			
☑	B12	FCER1A 4	Unknown	31.62			
☑	C1	GAPDH 5	Unknown	26.27			
☑	C2	GAPDH 5	Unknown	25.29			
☑	C3	GAPDH 5	Unknown	26.26			
☑	C4	GAPDH 7	Unknown	23.09			
☑	C5	GAPDH 7	Unknown	23.88			
☑	C6	GAPDH 7	Unknown	23.99			

> - Late Cp call (last five cycles) has higher uncertainty

Results

Inc	Pos	Name	Type	CP	Concentration	Standard	Status
☑	C7	GAPDH 11	Unknown	31.24			
☑	C8	GAPDH 11	Unknown	31.46			
☑	C9	GAPDH 11	Unknown	31.24			
☑	C10	GAPDH 14	Unknown	23.89			
☑	C11	GAPDH 14	Unknown	23.51			
☑	C12	GAPDH 14	Unknown	24.00			
☑	D1	FCER1A 5	Unknown	32.99			
☑	D2	FCER1A 5	Unknown	31.88			
☑	D3	FCER1A 5	Unknown	32.41			
☑	D4	FCER1A 7	Unknown	31.12			
☑	D5	FCER1A 7	Unknown	31.92			
☑	D6	FCER1A 7	Unknown	31.28			
☑	D7	FCER1A 11	Unknown	31.55			
☑	D8	FCER1A 11	Unknown	35.00			>
☑	D9	FCER1A 11	Unknown	33.80			
☑	D10	FCER1A 14	Unknown	32.30			
☑	D11	FCER1A 14	Unknown	31.67			
☑	D12	FCER1A 14	Unknown	31.21			
☑	E1	GAPDH 15	Unknown	22.94			
☑	E2	GAPDH 15	Unknown	22.68			
☑	E3	GAPDH 15	Unknown	22.51			
☑	E4	GAPDH 18	Unknown	23.62			
☑	E5	GAPDH 18	Unknown	23.66			
☑	E6	GAPDH 18	Unknown	23.62			
☑	E7	GAPDH 19	Unknown	22.90			
☑	E8	GAPDH 19	Unknown	23.00			
☑	E9	GAPDH 19	Unknown	24.31			
☑	E10	GAPDH 20	Unknown	22.95			
☑	E11	GAPDH 20	Unknown	22.85			
☑	E12	GAPDH 20	Unknown	22.86			
☑	F1	FCER1A 15	Unknown	32.29			
☑	F2	FCER1A 15	Unknown	31.06			
☑	F3	FCER1A 15	Unknown	31.63			
☑	F4	FCER1A 18	Unknown	29.90			
☑	F5	FCER1A 18	Unknown	30.62			
☑	F6	FCER1A 18	Unknown	31.15			
☑	F7	FCER1A 19	Unknown	29.75			

> - Late Cp call (last five cycles) has higher uncertainty

Results

Inc	Pos	Name	Type	CP	Concentration	Standard	Status
☑	F8	FCER1A 19	Unknown	29.98			
☑	F9	FCER1A 19	Unknown	30.63			
☑	F10	FCER1A 20	Unknown	30.12			
☑	F11	FCER1A 20	Unknown	31.40			
☑	F12	FCER1A 20	Unknown	30.90			
☑	G1	GAPDH 24	Unknown	21.98			
☑	G2	GAPDH 24	Unknown	21.79			
☑	G3	GAPDH 24	Unknown	21.94			
☑	G4	GAPDH 26	Unknown	23.57			
☑	G5	GAPDH 26	Unknown	23.78			
☑	G6	GAPDH 26	Unknown	23.78			
☑	G7		Unknown				
☑	G8		Unknown				
☑	G9		Unknown				
☑	G10		Unknown				
☑	G11		Unknown				
☑	G12		Unknown				
☑	H1	FCER1A 24	Unknown	31.74			
☑	H2	FCER1A 24	Unknown	31.81			
☑	H3	FCER1A 24	Unknown	28.46			
☑	H4	FCER1A 26	Unknown	32.63			
☑	H5	FCER1A 26	Unknown	32.42			
☑	H6	FCER1A 26	Unknown	32.34			
☑	H7		Unknown				
☑	H8		Unknown				
☑	H9		Unknown				
☑	H10		Unknown				
☑	H11		Unknown				
☑	H12		Unknown				

> - Late Cp call (last five cycles) has higher uncertainty

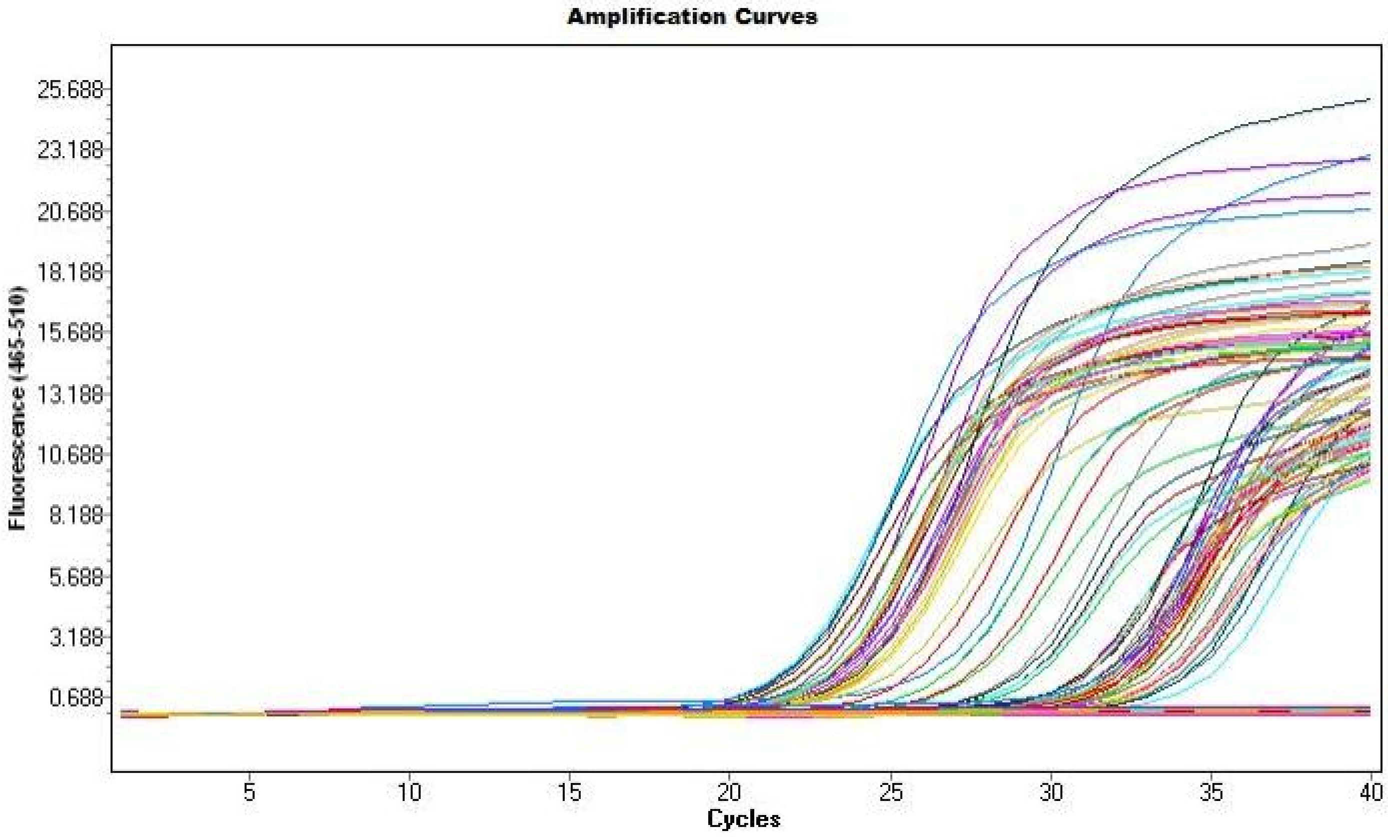

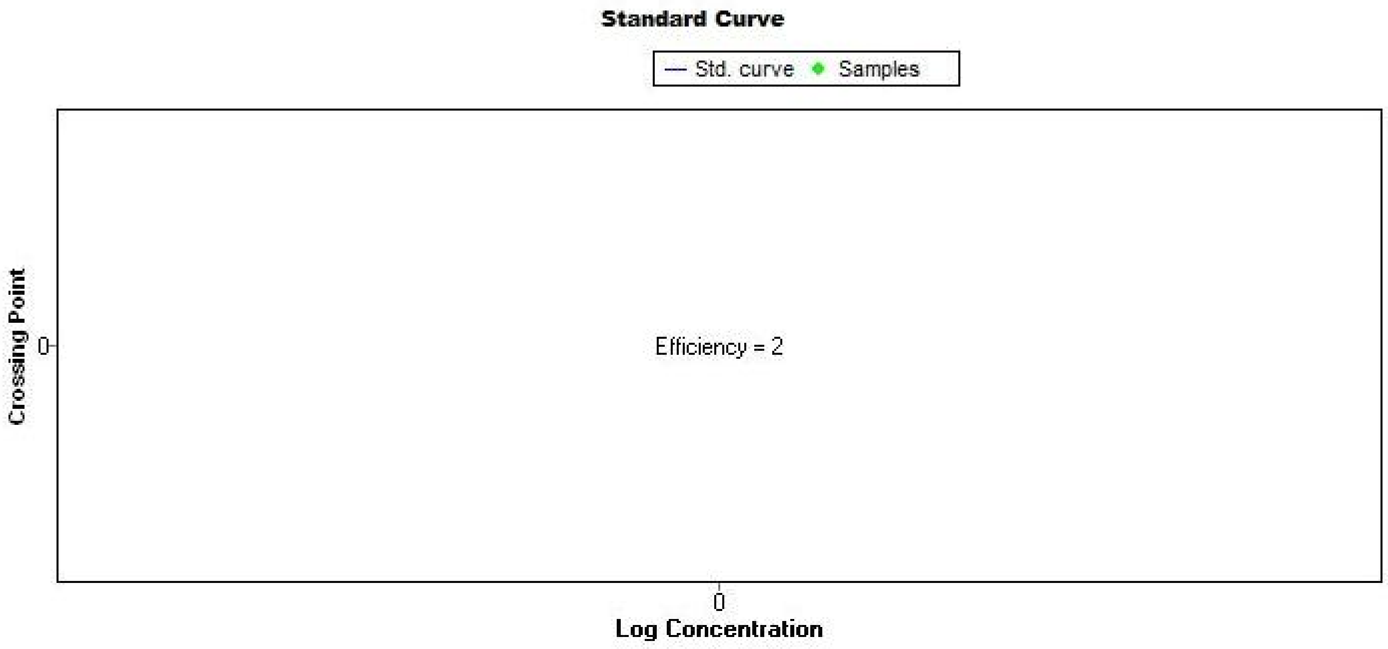

Analysis Notes	

Abs Quant/Fit Points for All Samples (Abs Quant/Fit Points)

Settings

Channel	465-510				
Color Compensation	Off				
Standard Curve	Set Efficiency (2.00)				
Program	Program			Units	
First Cycle	1	Last Cycle	40	Background	2-6
Noiseband Method	Auto	Noiseband	0.5193	STD Dev Multiplier	12.0000
# of Fit Points	2	Threshold Method	Auto	Threshold	0.5193

Subset Name	All Samples

Results

Inc	Pos	Name	Type	CP	Concentration	Standard	Status
☑	A1	GAPDH 1	Unknown	9.86			
☑	A2	GAPDH 1	Unknown	25.08			
☑	A3	GAPDH 1	Unknown	24.98			
☑	A4	GAPDH 2	Unknown	29.63			
☑	A5	GAPDH 2	Unknown	26.46			
☑	A6	GAPDH 2	Unknown	29.61			
☑	A7	GAPDH 3	Unknown	19.44			
☑	A8	GAPDH 3	Unknown	18.81			
☑	A9	GAPDH 3	Unknown	18.79			
☑	A10	GAPDH 4	Unknown	30.00			
☑	A11	GAPDH 4	Unknown	29.96			
☑	A12	GAPDH 4	Unknown	19.05			
☑	B1	FCER1A 1	Unknown	31.68			
☑	B2	FCER1A 1	Unknown				
☑	B3	FCER1A 1	Unknown				
☑	B4	FCER1A 2	Unknown				
☑	B5	FCER1A 2	Unknown	30.31			
☑	B6	FCER1A 2	Unknown	31.50			
☑	B7	FCER1A 3	Unknown	26.83			
☑	B8	FCER1A 3	Unknown	26.94			
☑	B9	FCER1A 3	Unknown	26.54			
☑	B10	FCER1A 4	Unknown	31.36			
☑	B11	FCER1A 4	Unknown	30.21			
☑	B12	FCER1A 4	Unknown	30.41			
☑	C1	GAPDH 5	Unknown	24.39			
☑	C2	GAPDH 5	Unknown	23.27			
☑	C3	GAPDH 5	Unknown	24.24			

Results

Inc	Pos	Name	Type	CP	Concentration	Standard	Status
☑	C4	GAPDH 7	Unknown	21.00			
☑	C5	GAPDH 7	Unknown	20.65			
☑	C6	GAPDH 7	Unknown	21.58			
☑	C7	GAPDH 11	Unknown	28.87			
☑	C8	GAPDH 11	Unknown	29.28			
☑	C9	GAPDH 11	Unknown	27.40			
☑	C10	GAPDH 14	Unknown	21.58			
☑	C11	GAPDH 14	Unknown	20.69			
☑	C12	GAPDH 14	Unknown	21.76			
☑	D1	FCER1A 5	Unknown	31.65			
☑	D2	FCER1A 5	Unknown	30.57			
☑	D3	FCER1A 5	Unknown	31.27			
☑	D4	FCER1A 7	Unknown	29.38			
☑	D5	FCER1A 7	Unknown	29.48			
☑	D6	FCER1A 7	Unknown	29.28			
☑	D7	FCER1A 11	Unknown	29.95			
☑	D8	FCER1A 11	Unknown	32.44			
☑	D9	FCER1A 11	Unknown	31.83			
☑	D10	FCER1A 14	Unknown	30.52			
☑	D11	FCER1A 14	Unknown	29.64			
☑	D12	FCER1A 14	Unknown	29.58			
☑	E1	GAPDH 15	Unknown	20.53			
☑	E2	GAPDH 15	Unknown	20.09			
☑	E3	GAPDH 15	Unknown	20.32			
☑	E4	GAPDH 18	Unknown	21.37			
☑	E5	GAPDH 18	Unknown	20.84			
☑	E6	GAPDH 18	Unknown	21.16			
☑	E7	GAPDH 19	Unknown	20.63			
☑	E8	GAPDH 19	Unknown	20.41			
☑	E9	GAPDH 19	Unknown	21.43			
☑	E10	GAPDH 20	Unknown	20.68			
☑	E11	GAPDH 20	Unknown	20.40			
☑	E12	GAPDH 20	Unknown	20.56			
☑	F1	FCER1A 15	Unknown	31.13			
☑	F2	FCER1A 15	Unknown	29.76			
☑	F3	FCER1A 15	Unknown	30.48			
☑	F4	FCER1A 18	Unknown	28.50			

Results

Inc	Pos	Name	Type	CP	Concentration	Standard	Status
☑	F5	FCER1A 18	Unknown	28.89			
☑	F6	FCER1A 18	Unknown	29.81			
☑	F7	FCER1A 19	Unknown	28.49			
☑	F8	FCER1A 19	Unknown	28.61			
☑	F9	FCER1A 19	Unknown	27.97			
☑	F10	FCER1A 20	Unknown	28.84			
☑	F11	FCER1A 20	Unknown	29.55			
☑	F12	FCER1A 20	Unknown	29.63			
☑	G1	GAPDH 24	Unknown	18.70			
☑	G2	GAPDH 24	Unknown	19.58			
☑	G3	GAPDH 24	Unknown	19.57			
☑	G4	GAPDH 26	Unknown	20.99			
☑	G5	GAPDH 26	Unknown	20.84			
☑	G6	GAPDH 26	Unknown	21.33			
☑	G7		Unknown				
☑	G8		Unknown				
☑	G9		Unknown				
☑	G10		Unknown				
☑	G11		Unknown				
☑	G12		Unknown				
☑	H1	FCER1A 24	Unknown	16.54			
☑	H2	FCER1A 24	Unknown	28.81			
☑	H3	FCER1A 24	Unknown	27.34			
☑	H4	FCER1A 26	Unknown	30.78			
☑	H5	FCER1A 26	Unknown	30.54			
☑	H6	FCER1A 26	Unknown	31.00			
☑	H7		Unknown				
☑	H8		Unknown				
☑	H9		Unknown				
☑	H10		Unknown				
☑	H11		Unknown	36.28			
☑	H12		Unknown				

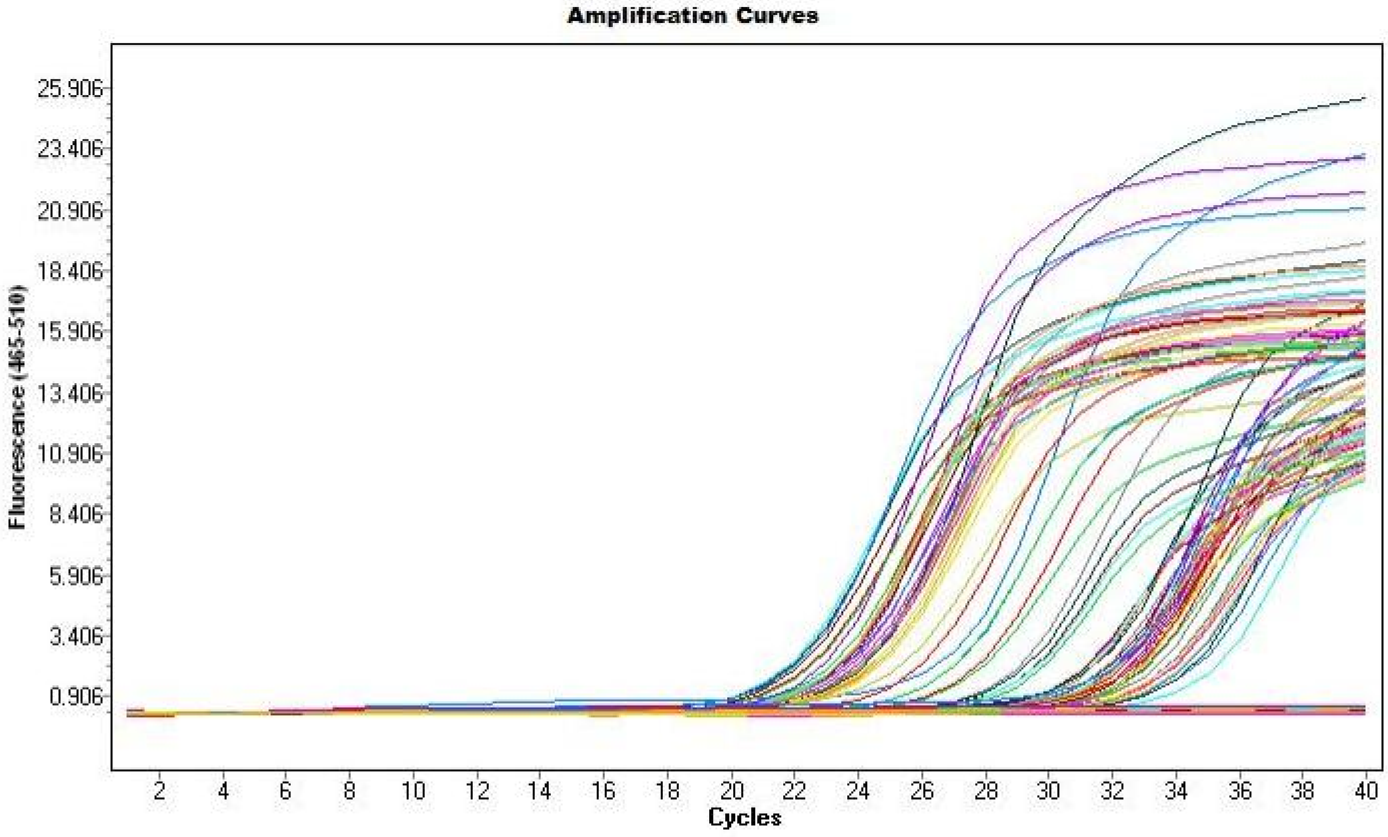
Amplification Curves
Fluorescence (465-510)
25.906
23.406
20.906
18.406
15.906
13.406
10.906
8.406
5.906
3.406
0.906
2
4
6
8
10
12
14
16
18
20
22
24
26
28
30
32
34
36
38
40
Cycles

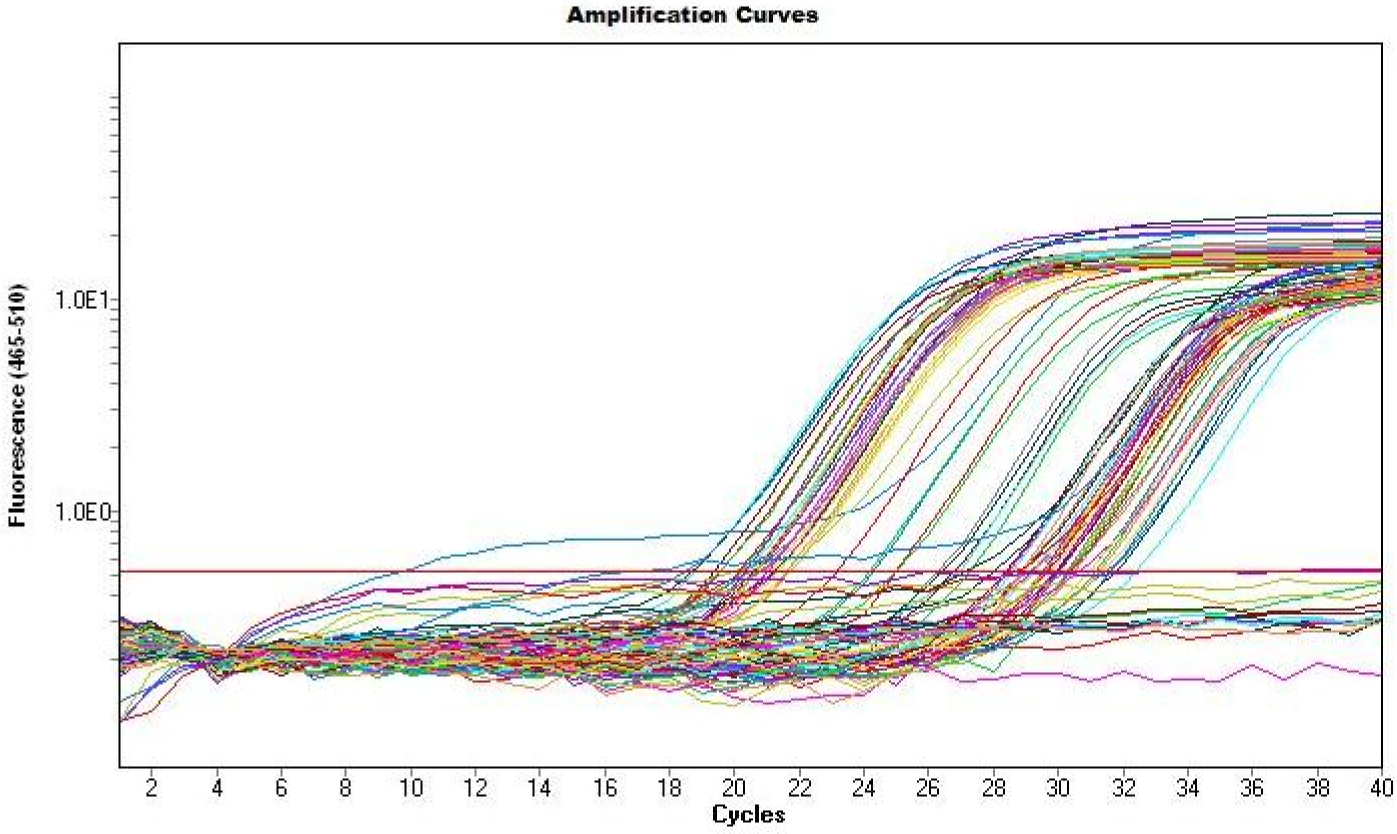
Amplification Curves
Fluorescence (465-510)
1.0E1
1.0E0
2
4
6
8
10
12
14
16
18
20
22
24
26
28
30
32
34
36
38
40
Cycles

Amplification Curves

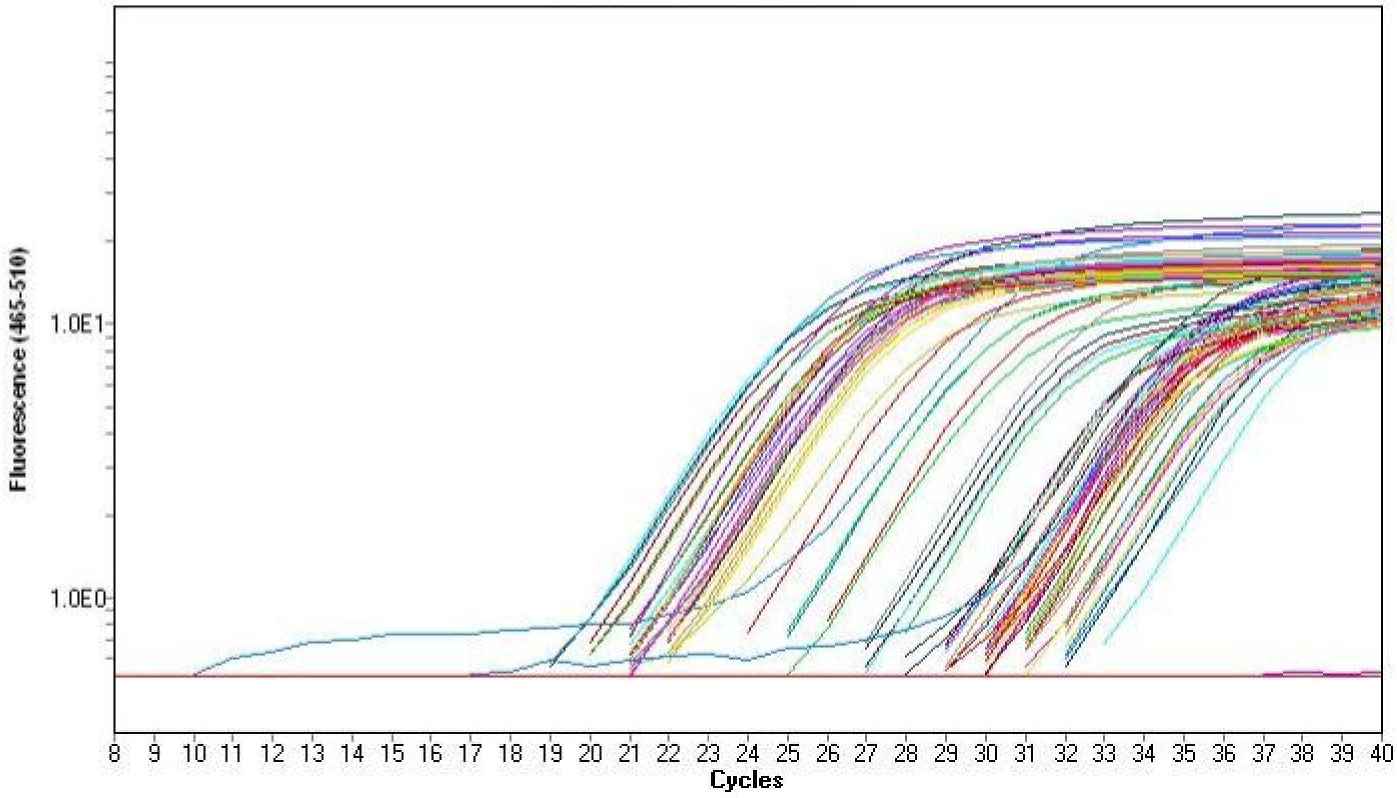

Standard Curve

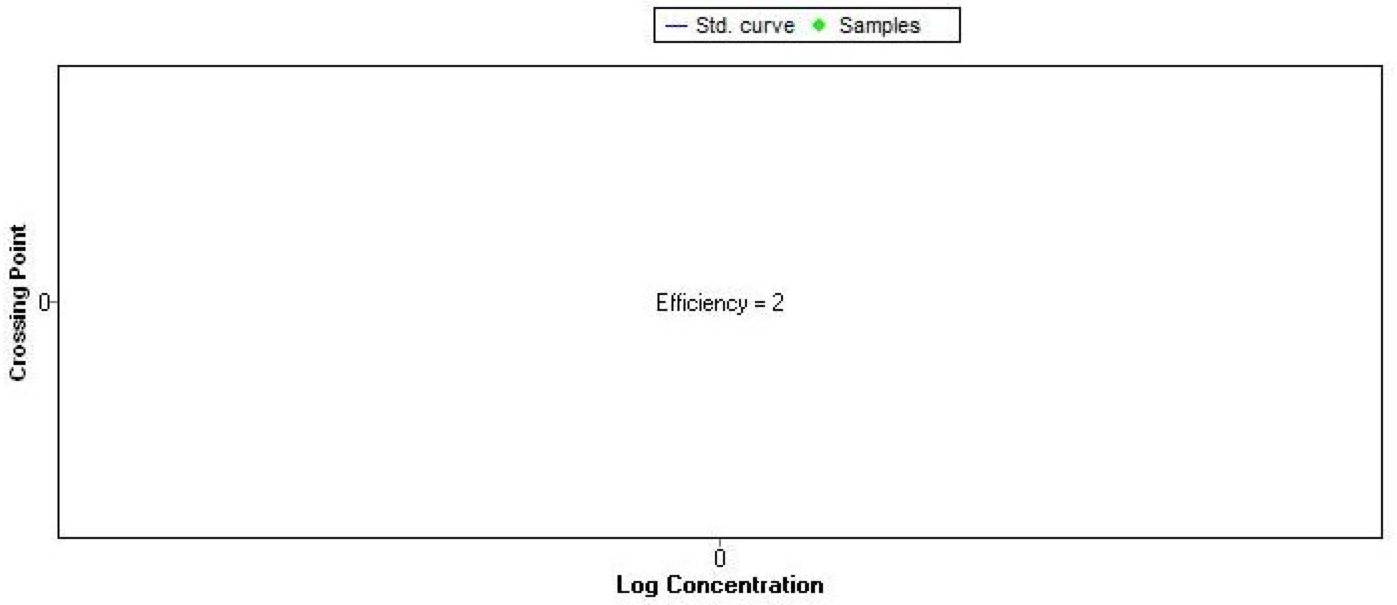

Analysis Notes	

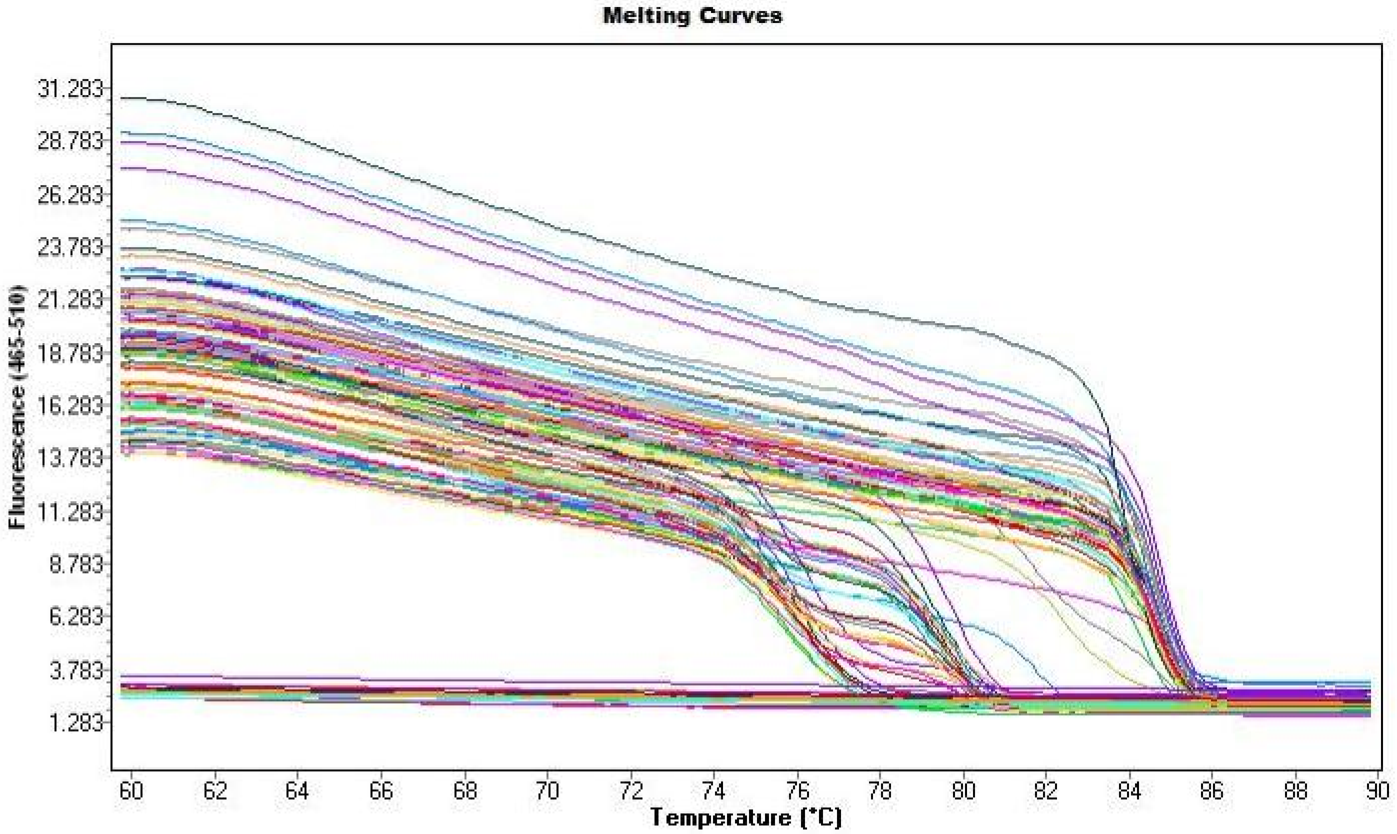

Melting Curves
Fluorescence (465-510)
31.283
28.783
26.283
23.783
21.283
18.783
16.283
13.783
11.283
8.783
6.283
3.783
1.283
60
62
64
66
68
70
72
74
76
78
80
82
84
86
88
90
Temperature (°C)

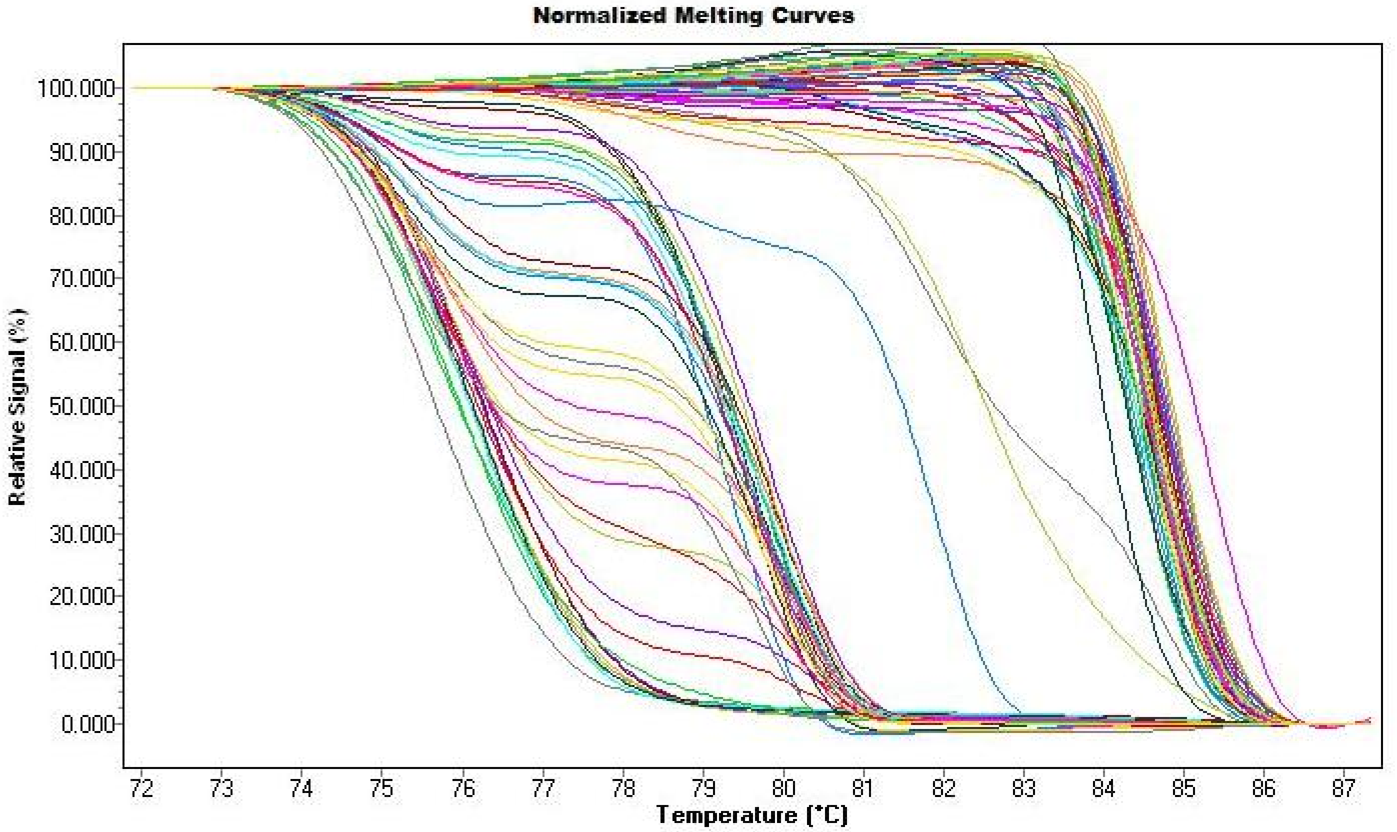

Normalized Melting Curves
Relative Signal (%)
100.000
90.000
80.000
70.000
60.000
50.000
40.000
30.000
20.000
10.000
0.000
72
73
74
75
76
77
78
79
80
81
82
83
84
85
86
87
Temperature (°C)

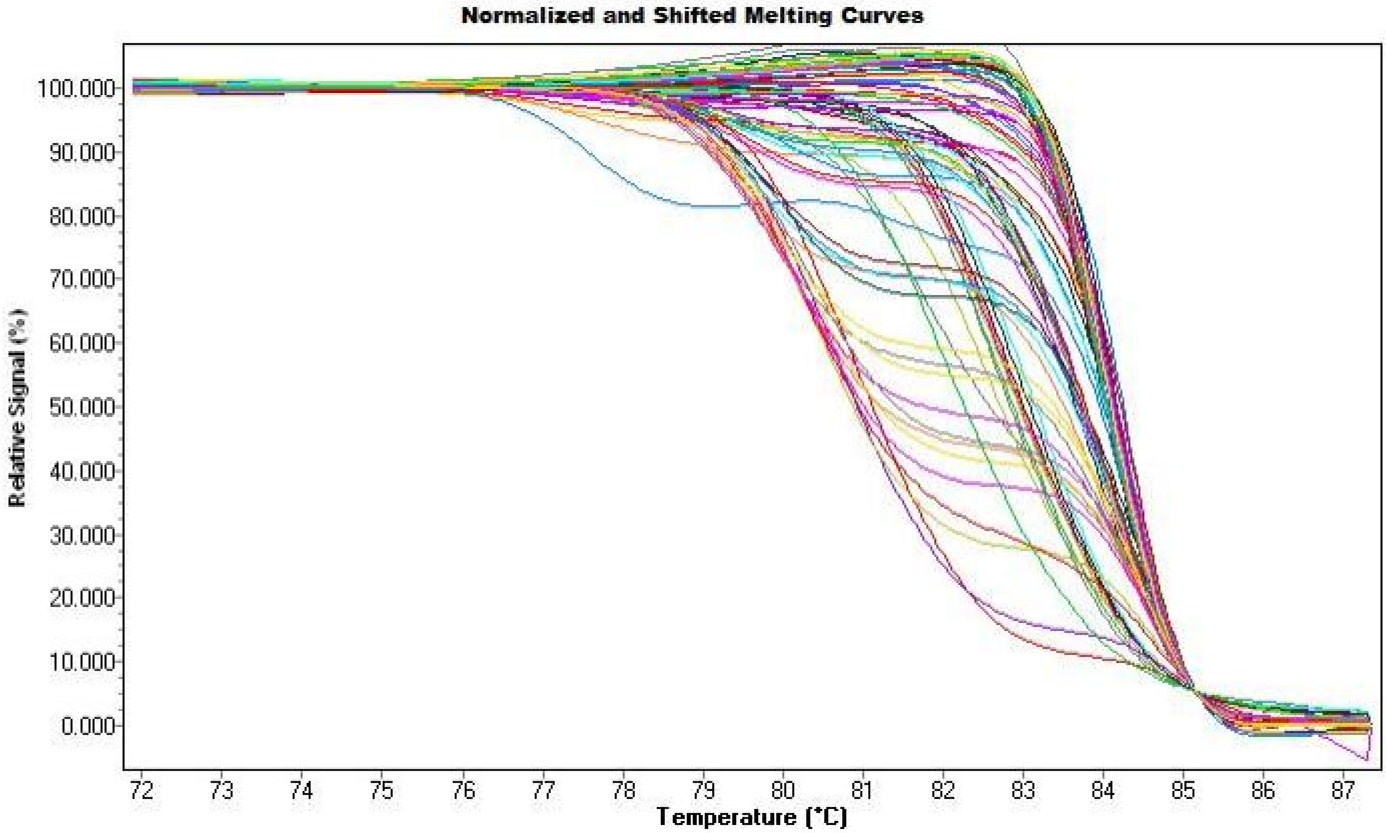

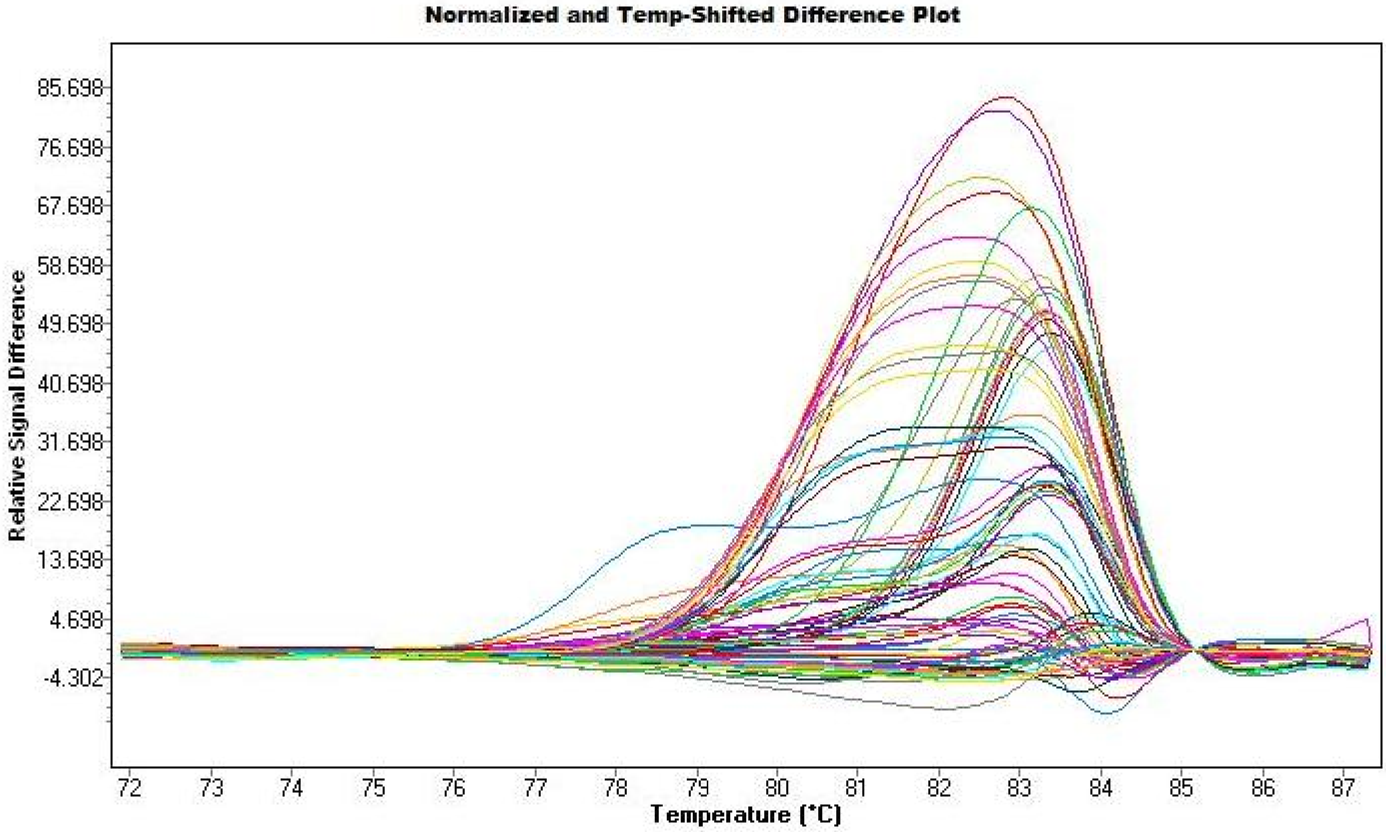

**Analysis Notes**	

Melting Curves

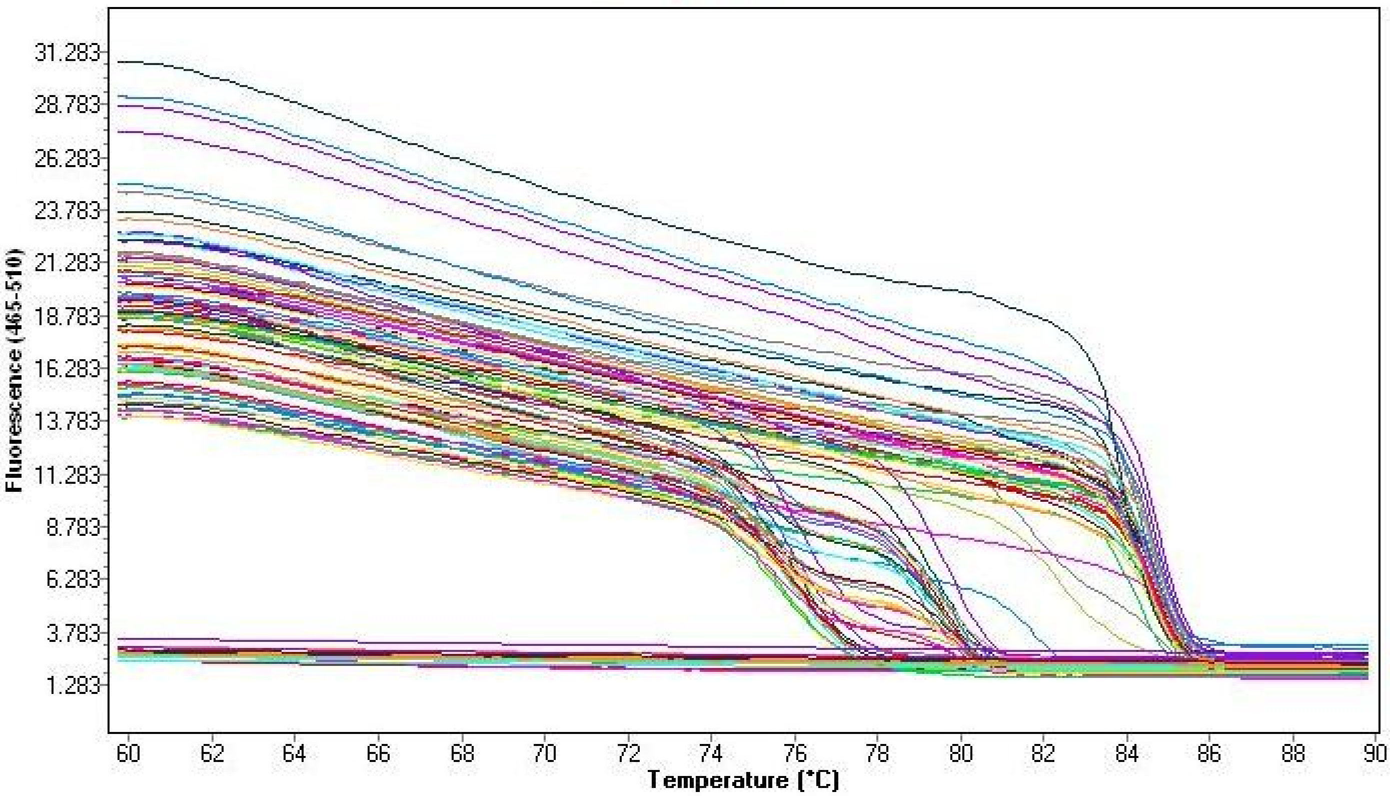

Melting Peaks

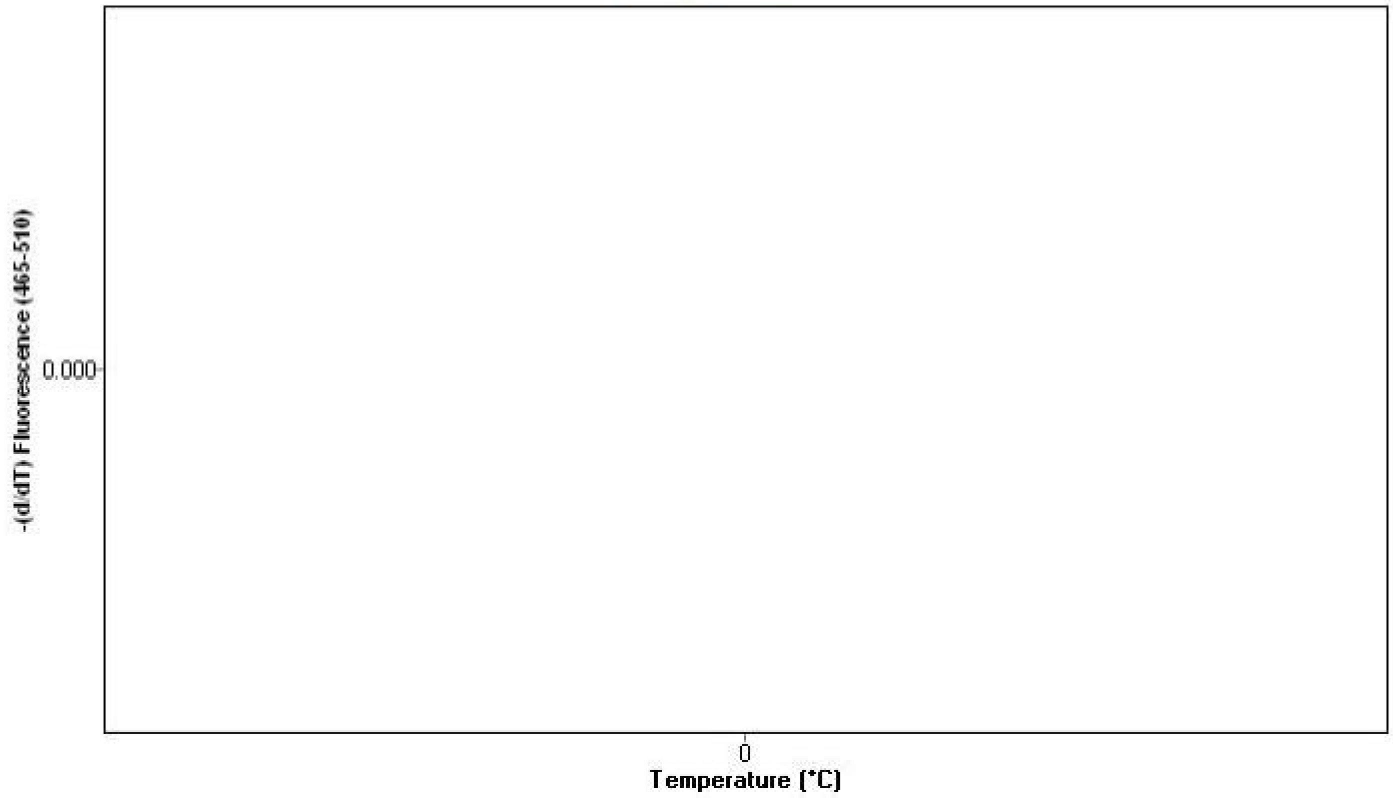

Analysis Notes	

Melt Curve Genotyping for All Samples (1) (Melt Curve Genotyping)

Channel	465-510	Color Compensation	Off				
Progam Name	Program						
Stds Settings	Auto-Group						
Sensitivity	Normal	Temp Range	62-85	Score	0.7	Res	0.1

Subset Name	All Samples

Results

Inc	Pos.	Sample Name	Sample Type	Group	Score	Resolution	Status
☑	A1	GAPDH 1	Unknown	1	0.99	0.58	
☑	A2	GAPDH 1	Unknown	1	0.98	0.48	
☑	A3	GAPDH 1	Unknown	1	0.97	0.64	
☑	A4	GAPDH 2	Unknown	1	0.94	0.34	
☑	A5	GAPDH 2	Unknown	1	0.71	0.15	
☑	A6	GAPDH 2	Unknown	1	0.93	0.31	
☑	A7	GAPDH 3	Unknown	1	0.98	0.64	
☑	A8	GAPDH 3	Unknown	1	0.99	0.65	
☑	A9	GAPDH 3	Unknown	1	0.92	0.70	
☑	A10	GAPDH 4	Unknown	1	0.88	0.15	

Results

Inc	Pos.	Sample Name	Sample Type	Group	Score	Resolution	Status
☑	A11	GAPDH 4	Unknown	1	0.99	0.52	
☑	A12	GAPDH 4	Unknown	1	0.85	0.31	
☑	B1	FCER1A 1	Unknown	4	0.96	0.41	
☑	B2	FCER1A 1	Unknown	Negative	1.00	1.00	
☑	B3	FCER1A 1	Unknown	5	1.00	0.99	
☑	B4	FCER1A 2	Unknown	5	0.74	0.74	
☑	B5	FCER1A 2	Unknown	2	0.99	0.36	
☑	B6	FCER1A 2	Unknown	2	0.99	0.36	
☑	B7	FCER1A 3	Unknown	4	0.95	0.55	
☑	B8	FCER1A 3	Unknown	4	0.98	0.43	
☑	B9	FCER1A 3	Unknown	4	0.93	0.53	
☑	B10	FCER1A 4	Unknown	2	0.98	0.36	
☑	B11	FCER1A 4	Unknown	2	0.99	0.36	
☑	B12	FCER1A 4	Unknown	2	0.99	0.49	
☑	C1	GAPDH 5	Unknown	1	0.99	0.60	
☑	C2	GAPDH 5	Unknown	1	0.95	0.31	
☑	C3	GAPDH 5	Unknown	1	0.98	0.60	
☑	C4	GAPDH 7	Unknown	1	0.96	0.36	
☑	C5	GAPDH 7	Unknown	1	0.94	0.67	
☑	C6	GAPDH 7	Unknown	1	0.99	0.56	
☑	C7	GAPDH 11	Unknown	1	0.93	0.35	
☑	C8	GAPDH 11	Unknown	1	0.93	0.40	
☑	C9	GAPDH 11	Unknown	1	0.95	0.44	
☑	C10	GAPDH 14	Unknown	1	0.99	0.64	
☑	C11	GAPDH 14	Unknown	1	0.98	0.44	
☑	C12	GAPDH 14	Unknown	1	0.98	0.62	
☑	D1	FCER1A 5	Unknown	6	1.00	0.31	
☑	D2	FCER1A 5	Unknown	2	0.98	0.39	
☑	D3	FCER1A 5	Unknown	2	0.99	0.50	
☑	D4	FCER1A 7	Unknown	4	0.94	0.32	
☑	D5	FCER1A 7	Unknown	2	1.00	0.48	
☑	D6	FCER1A 7	Unknown	3	0.91	0.23	
☑	D7	FCER1A 11	Unknown	2	0.99	0.46	
☑	D8	FCER1A 11	Unknown	2	0.98	0.49	
☑	D9	FCER1A 11	Unknown	2	0.99	0.50	
☑	D10	FCER1A 14	Unknown	Unknown	0.89	0.08	
☑	D11	FCER1A 14	Unknown	2	0.95	0.31	
☑	D12	FCER1A 14	Unknown	2	0.87	0.11	

Results

Inc	Pos.	Sample Name	Sample Type	Group	Score	Resolution	Status
☑	E1	GAPDH 15	Unknown	1	0.99	0.62	
☑	E2	GAPDH 15	Unknown	1	0.98	0.48	
☑	E3	GAPDH 15	Unknown	1	0.99	0.51	
☑	E4	GAPDH 18	Unknown	1	0.99	0.61	
☑	E5	GAPDH 18	Unknown	1	0.95	0.69	
☑	E6	GAPDH 18	Unknown	1	0.99	0.60	
☑	E7	GAPDH 19	Unknown	1	0.99	0.59	
☑	E8	GAPDH 19	Unknown	1	0.98	0.62	
☑	E9	GAPDH 19	Unknown	1	0.90	0.71	
☑	E10	GAPDH 20	Unknown	1	0.99	0.62	
☑	E11	GAPDH 20	Unknown	1	0.99	0.52	
☑	E12	GAPDH 20	Unknown	1	1.00	0.55	
☑	F1	FCER1A 15	Unknown	4	0.99	0.50	
☑	F2	FCER1A 15	Unknown	Unknown	0.84	0.04	
☑	F3	FCER1A 15	Unknown	4	1.00	0.51	
☑	F4	FCER1A 18	Unknown	3	0.97	0.38	
☑	F5	FCER1A 18	Unknown	3	0.99	0.30	
☑	F6	FCER1A 18	Unknown	3	0.98	0.27	
☑	F7	FCER1A 19	Unknown	Unknown	0.91	0.09	
☑	F8	FCER1A 19	Unknown	3	0.88	0.11	
☑	F9	FCER1A 19	Unknown	3	0.95	0.16	
☑	F10	FCER1A 20	Unknown	3	0.94	0.31	
☑	F11	FCER1A 20	Unknown	4	0.99	0.53	
☑	F12	FCER1A 20	Unknown	4	0.99	0.50	
☑	G1	GAPDH 24	Unknown	1	0.98	0.65	
☑	G2	GAPDH 24	Unknown	1	0.99	0.60	
☑	G3	GAPDH 24	Unknown	1	0.97	0.66	
☑	G4	GAPDH 26	Unknown	1	0.99	0.49	
☑	G5	GAPDH 26	Unknown	1	0.89	0.72	
☑	G6	GAPDH 26	Unknown	1	0.99	0.62	
☑	G7		Unknown	Negative	1.00	1.00	
☑	G8		Unknown	Negative	1.00	1.00	
☑	G9		Unknown	Negative	1.00	1.00	
☑	G10		Unknown	Negative	1.00	1.00	
☑	G11		Unknown	Negative	1.00	1.00	
☑	G12		Unknown	Negative	1.00	1.00	
☑	H1	FCER1A 24	Unknown	3	0.88	0.12	
☑	H2	FCER1A 24	Unknown	4	0.96	0.37	

Results

Inc	Pos.	Sample Name	Sample Type	Group	Score	Resolution	Status
☑	H3	FCER1A 24	Unknown	2	0.98	0.45	
☑	H4	FCER1A 26	Unknown	3	0.87	0.13	
☑	H5	FCER1A 26	Unknown	3	0.95	0.32	
☑	H6	FCER1A 26	Unknown	3	1.00	0.31	
☑	H7		Unknown	Negative	1.00	1.00	
☑	H8		Unknown	Negative	1.00	1.00	
☑	H9		Unknown	Negative	1.00	1.00	
☑	H10		Unknown	Negative	1.00	1.00	
☑	H11		Unknown	Negative	1.00	1.00	
☑	H12		Unknown	Negative	1.00	1.00	

**Group**	1

Inc	Pos	Sample Name	Sample Type	Score	Resolution	Status
☑	A1	GAPDH 1	Unknown	0.99	0.58	
☑	A2	GAPDH 1	Unknown	0.98	0.48	
☑	A3	GAPDH 1	Unknown	0.97	0.64	
☑	A4	GAPDH 2	Unknown	0.94	0.34	
☑	A5	GAPDH 2	Unknown	0.71	0.15	
☑	A6	GAPDH 2	Unknown	0.93	0.31	
☑	A7	GAPDH 3	Unknown	0.98	0.64	
☑	A8	GAPDH 3	Unknown	0.99	0.65	
☑	A9	GAPDH 3	Unknown	0.92	0.70	
☑	A10	GAPDH 4	Unknown	0.88	0.15	
☑	A11	GAPDH 4	Unknown	0.99	0.52	
☑	A12	GAPDH 4	Unknown	0.85	0.31	
☑	C1	GAPDH 5	Unknown	0.99	0.60	
☑	C2	GAPDH 5	Unknown	0.95	0.31	
☑	C3	GAPDH 5	Unknown	0.98	0.60	
☑	C4	GAPDH 7	Unknown	0.96	0.36	
☑	C5	GAPDH 7	Unknown	0.94	0.67	
☑	C6	GAPDH 7	Unknown	0.99	0.56	
☑	C7	GAPDH 11	Unknown	0.93	0.35	
☑	C8	GAPDH 11	Unknown	0.93	0.40	
☑	C9	GAPDH 11	Unknown	0.95	0.44	
☑	C10	GAPDH 14	Unknown	0.99	0.64	
☑	C11	GAPDH 14	Unknown	0.98	0.44	
☑	C12	GAPDH 14	Unknown	0.98	0.62	
☑	E1	GAPDH 15	Unknown	0.99	0.62	

Group	1

Inc	Pos	Sample Name	Sample Type	Score	Resolution	Status
☑	E2	GAPDH 15	Unknown	0.98	0.48	
☑	E3	GAPDH 15	Unknown	0.99	0.51	
☑	E4	GAPDH 18	Unknown	0.99	0.61	
☑	E5	GAPDH 18	Unknown	0.95	0.69	
☑	E6	GAPDH 18	Unknown	0.99	0.60	
☑	E7	GAPDH 19	Unknown	0.99	0.59	
☑	E8	GAPDH 19	Unknown	0.98	0.62	
☑	E9	GAPDH 19	Unknown	0.90	0.71	
☑	E10	GAPDH 20	Unknown	0.99	0.62	
☑	E11	GAPDH 20	Unknown	0.99	0.52	
☑	E12	GAPDH 20	Unknown	1.00	0.55	
☑	G1	GAPDH 24	Unknown	0.98	0.65	
☑	G2	GAPDH 24	Unknown	0.99	0.60	
☑	G3	GAPDH 24	Unknown	0.97	0.66	
☑	G4	GAPDH 26	Unknown	0.99	0.49	
☑	G5	GAPDH 26	Unknown	0.89	0.72	
☑	G6	GAPDH 26	Unknown	0.99	0.62	

Group	2

Inc	Pos	Sample Name	Sample Type	Score	Resolution	Status
☑	B5	FCER1A 2	Unknown	0.99	0.36	
☑	B6	FCER1A 2	Unknown	0.99	0.36	
☑	B10	FCER1A 4	Unknown	0.98	0.36	
☑	B11	FCER1A 4	Unknown	0.99	0.36	
☑	B12	FCER1A 4	Unknown	0.99	0.49	
☑	D2	FCER1A 5	Unknown	0.98	0.39	
☑	D3	FCER1A 5	Unknown	0.99	0.50	
☑	D5	FCER1A 7	Unknown	1.00	0.48	
☑	D7	FCER1A 11	Unknown	0.99	0.46	
☑	D8	FCER1A 11	Unknown	0.98	0.49	
☑	D9	FCER1A 11	Unknown	0.99	0.50	
☑	D11	FCER1A 14	Unknown	0.95	0.31	
☑	D12	FCER1A 14	Unknown	0.87	0.11	
☑	H3	FCER1A 24	Unknown	0.98	0.45	

Group	3

Inc	Pos	Sample Name	Sample Type	Score	Resolution	Status
☑	D6	FCER1A 7	Unknown	0.91	0.23	

Group	3

Inc	Pos	Sample Name	Sample Type	Score	Resolution	Status
☑	F4	FCER1A 18	Unknown	0.97	0.38	
☑	F5	FCER1A 18	Unknown	0.99	0.30	
☑	F6	FCER1A 18	Unknown	0.98	0.27	
☑	F8	FCER1A 19	Unknown	0.88	0.11	
☑	F9	FCER1A 19	Unknown	0.95	0.16	
☑	F10	FCER1A 20	Unknown	0.94	0.31	
☑	H1	FCER1A 24	Unknown	0.88	0.12	
☑	H4	FCER1A 26	Unknown	0.87	0.13	
☑	H5	FCER1A 26	Unknown	0.95	0.32	
☑	H6	FCER1A 26	Unknown	1.00	0.31	

Group	4

Inc	Pos	Sample Name	Sample Type	Score	Resolution	Status
☑	B1	FCER1A 1	Unknown	0.96	0.41	
☑	B7	FCER1A 3	Unknown	0.95	0.55	
☑	B8	FCER1A 3	Unknown	0.98	0.43	
☑	B9	FCER1A 3	Unknown	0.93	0.53	
☑	D4	FCER1A 7	Unknown	0.94	0.32	
☑	F1	FCER1A 15	Unknown	0.99	0.50	
☑	F3	FCER1A 15	Unknown	1.00	0.51	
☑	F11	FCER1A 20	Unknown	0.99	0.53	
☑	F12	FCER1A 20	Unknown	0.99	0.50	
☑	H2	FCER1A 24	Unknown	0.96	0.37	

Group	5

Inc	Pos	Sample Name	Sample Type	Score	Resolution	Status
☑	B3	FCER1A 1	Unknown	1.00	0.99	
☑	B4	FCER1A 2	Unknown	0.74	0.74	

Group	6

Inc	Pos	Sample Name	Sample Type	Score	Resolution	Status
☑	D1	FCER1A 5	Unknown	1.00	0.31	

Group	Negative

Inc	Pos	Sample Name	Sample Type	Score	Resolution	Status
☑	B2	FCER1A 1	Unknown	1.00	1.00	
☑	G7		Unknown	1.00	1.00	

Group	Negative

Inc	Pos	Sample Name	Sample Type	Score	Resolution	Status
☑	G8		Unknown	1.00	1.00	
☑	G9		Unknown	1.00	1.00	
☑	G10		Unknown	1.00	1.00	
☑	G11		Unknown	1.00	1.00	
☑	G12		Unknown	1.00	1.00	
☑	H7		Unknown	1.00	1.00	
☑	H8		Unknown	1.00	1.00	
☑	H9		Unknown	1.00	1.00	
☑	H10		Unknown	1.00	1.00	
☑	H11		Unknown	1.00	1.00	
☑	H12		Unknown	1.00	1.00	

Group	Unknown

Inc	Pos	Sample Name	Sample Type	Score	Resolution	Status
☑	D10	FCER1A 14	Unknown	0.89	0.08	
☑	F2	FCER1A 15	Unknown	0.84	0.04	
☑	F7	FCER1A 19	Unknown	0.91	0.09	

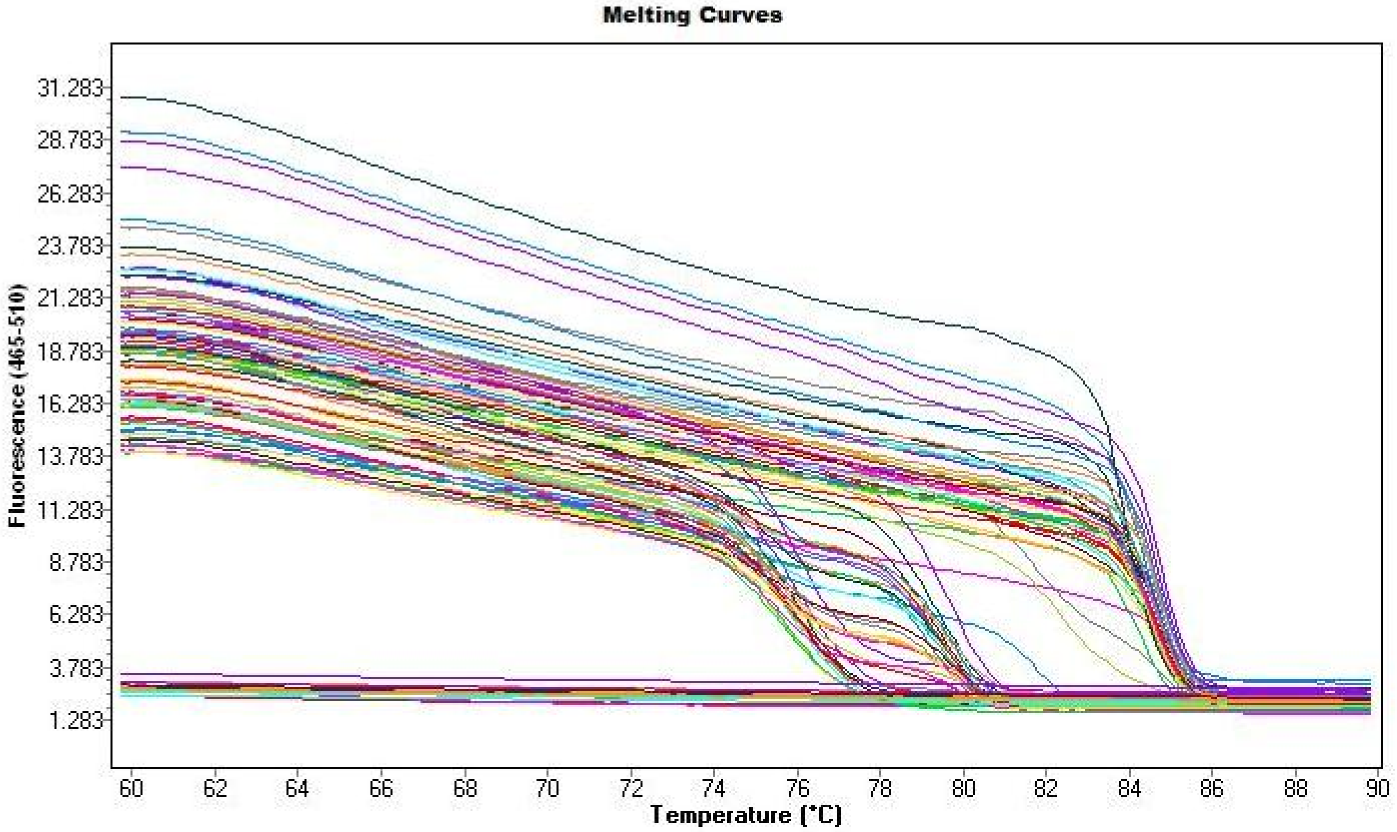

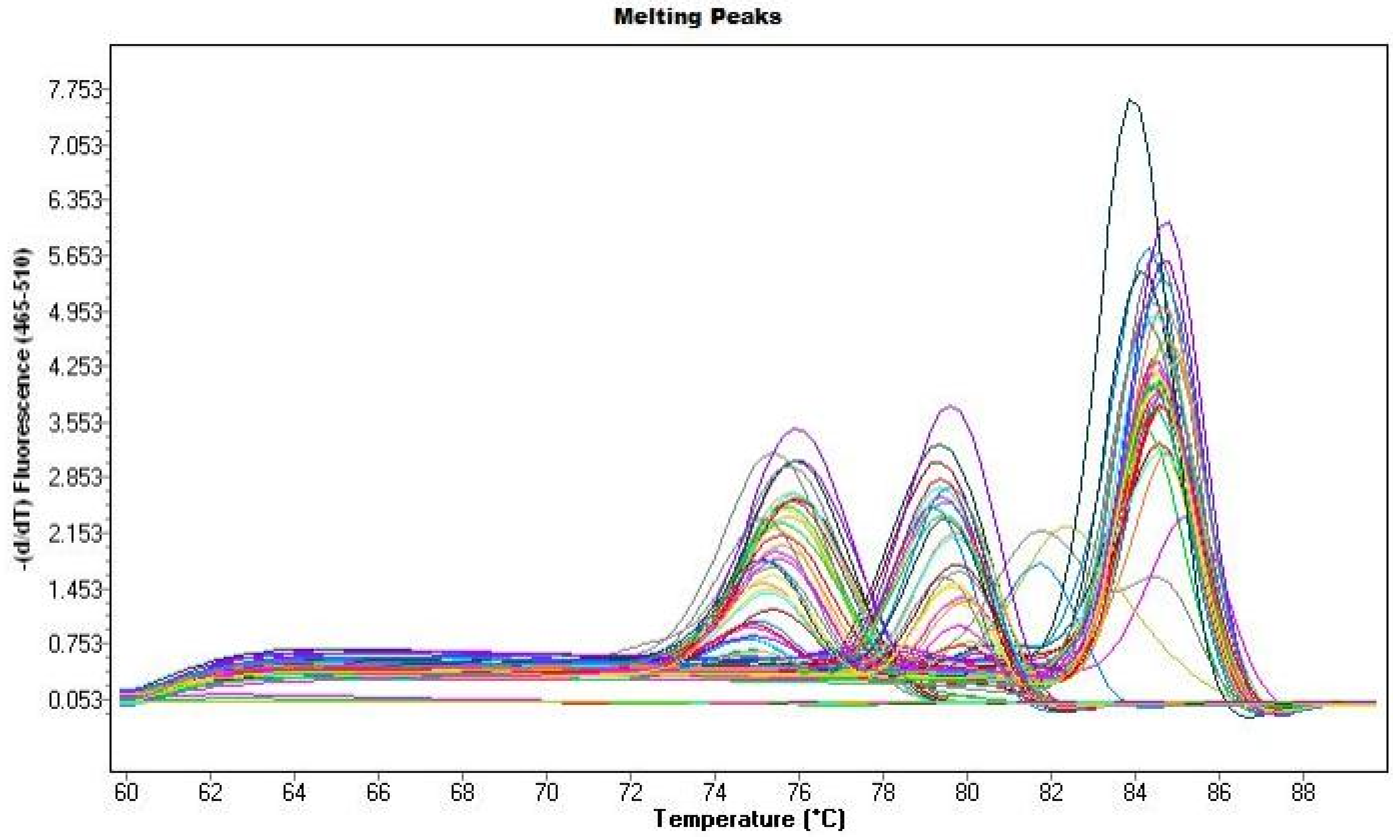

Analysis Notes	

Tm Calling for All Samples (Tm Calling)

Settings

Channel	465-510	Color Compensation	Off		
Program	Program				
Show Shoulders	False	Chemistry	HybProbe	Max Peaks	2

Subset Name	All Samples

Results

			Peak 1				Peak 2				
Inc	Pos	Sample Name	Tm	Area	Width	Height	Tm	Area	Width	Height	Status
☑	A1	GAPDH 1	84.36	13.00	2.21	5.89					
☑	A2	GAPDH 1	84.61	8.97	2.26	3.97					
☑	A3	GAPDH 1	84.26	8.48	2.34	3.62					
☑	A4	GAPDH 2	85.30	6.15	2.41	2.56					
☑	A5	GAPDH 2	83.06	11.43	5.44	2.10					
☑	A6	GAPDH 2	84.58	8.01	2.32	3.45					
☑	A7	GAPDH 3	84.57	10.14	2.23	4.54					
☑	A8	GAPDH 3	84.39	11.36	2.27	5.00					
☑	A9	GAPDH 3	84.27	12.98	2.30	5.65					

Results

Inc	Pos	Sample Name	Peak 1 Tm	Peak 1 Area	Peak 1 Width	Peak 1 Height	Peak 2 Tm	Peak 2 Area	Peak 2 Width	Peak 2 Height	Status
☑	A10	GAPDH 4	78.27	1.03	2.53	0.41	84.84	7.90	2.34	3.37	
☑	A11	GAPDH 4	84.68	10.23	2.19	4.66					
☑	A12	GAPDH 4	82.65	8.00	3.63	2.20					
☑	B1	FCER1A 1	74.61	1.26	2.25	0.56	79.20	6.23	2.39	2.61	
☑	B2	FCER1A 1									
☑	B3	FCER1A 1	66.28	0.41	8.02	0.05					
☑	B4	FCER1A 2									
☑	B5	FCER1A 2	75.89	9.71	3.19	3.04					
☑	B6	FCER1A 2	75.81	7.78	3.12	2.49					
☑	B7	FCER1A 3	79.35	8.56	2.69	3.18					
☑	B8	FCER1A 3	74.61	1.06	2.03	0.52	79.40	7.56	2.65	2.86	
☑	B9	FCER1A 3	79.39	9.45	2.77	3.41					
☑	B10	FCER1A 4	76.10	7.97	3.01	2.65					
☑	B11	FCER1A 4	76.17	10.02	3.20	3.13					
☑	B12	FCER1A 4	76.01	8.75	3.24	2.70					
☑	C1	GAPDH 5	84.81	9.17	2.19	4.18					
☑	C2	GAPDH 5	84.72	8.58	2.18	3.93					
☑	C3	GAPDH 5	84.62	9.53	2.26	4.22					
☑	C4	GAPDH 7	84.66	8.80	2.14	4.10					
☑	C5	GAPDH 7	84.54	13.61	2.29	5.95					
☑	C6	GAPDH 7	84.60	9.01	2.09	4.31					
☑	C7	GAPDH 11	84.58	8.79	2.49	3.52					
☑	C8	GAPDH 11	84.66	10.21	3.10	3.29					
☑	C9	GAPDH 11	84.57	11.36	2.67	4.25					
☑	C10	GAPDH 14	84.80	10.53	2.23	4.73					
☑	C11	GAPDH 14	84.78	11.80	2.02	5.83					
☑	C12	GAPDH 14	84.85	10.43	2.18	4.78					
☑	D1	FCER1A 5	75.06	1.71	2.21	0.77	81.70	5.12	2.83	1.81	
☑	D2	FCER1A 5	75.99	8.46	3.33	2.54					
☑	D3	FCER1A 5	75.85	8.16	3.20	2.55					
☑	D4	FCER1A 7	74.80	1.40	2.08	0.68	79.43	7.10	2.61	2.72	
☑	D5	FCER1A 7	75.54	10.20	3.17	3.21					
☑	D6	FCER1A 7	75.50	5.84	2.59	2.25	79.83	3.46	2.37	1.46	
☑	D7	FCER1A 11	76.14	8.45	3.22	2.62					
☑	D8	FCER1A 11	76.05	8.11	2.97	2.73					
☑	D9	FCER1A 11	76.06	9.50	3.03	3.14					

Results

Inc	Pos	Sample Name	Peak 1 Tm	Area	Width	Height	Peak 2 Tm	Area	Width	Height	Status
☑	D10	FCER1A 14	75.19	2.52	1.99	1.27	79.84	5.98	2.68	2.23	
☑	D11	FCER1A 14	75.99	11.84	3.49	3.39					
☑	D12	FCER1A 14	75.83	6.96	2.90	2.40	80.17	1.96	2.26	0.87	
☑	E1	GAPDH 15	84.45	8.67	2.08	4.16					
☑	E2	GAPDH 15	84.57	9.39	2.26	4.16					
☑	E3	GAPDH 15	84.50	8.81	2.26	3.89					
☑	E4	GAPDH 18	84.56	10.07	2.28	4.41					
☑	E5	GAPDH 18	84.30	11.72	2.35	4.99					
☑	E6	GAPDH 18	84.49	9.51	2.17	4.38					
☑	E7	GAPDH 19	84.66	10.08	2.18	4.62					
☑	E8	GAPDH 19	84.55	11.31	2.27	4.99					
☑	E9	GAPDH 19	83.98	17.44	2.21	7.90					
☑	E10	GAPDH 20	84.76	11.44	2.18	5.25					
☑	E11	GAPDH 20	84.81	12.68	2.01	6.32					
☑	E12	GAPDH 20	84.69	9.60	2.08	4.61					
☑	F1	FCER1A 15	74.66	0.89	2.26	0.39	79.49	7.13	2.69	2.65	
☑	F2	FCER1A 15	75.73	6.26	2.90	2.16	79.93	2.14	2.66	0.80	
☑	F3	FCER1A 15	74.53	0.71	2.01	0.35	79.36	6.58	2.65	2.48	
☑	F4	FCER1A 18	75.55	4.84	2.59	1.87	79.96	3.85	2.46	1.56	
☑	F5	FCER1A 18	75.41	3.77	2.35	1.61	79.86	4.50	2.52	1.79	
☑	F6	FCER1A 18	75.28	3.09	2.38	1.30	79.75	3.88	2.53	1.53	
☑	F7	FCER1A 19	75.34	2.32	2.44	0.95	79.82	4.64	2.53	1.83	
☑	F8	FCER1A 19	75.25	2.59	2.20	1.18	79.77	5.62	2.56	2.20	
☑	F9	FCER1A 19	75.10	3.57	2.30	1.55	79.55	5.95	2.45	2.43	
☑	F10	FCER1A 20	75.67	5.23	2.55	2.05	80.04	3.60	2.43	1.48	
☑	F11	FCER1A 20	79.64	10.37	2.65	3.91					
☑	F12	FCER1A 20	74.82	0.59	1.73	0.34	79.60	7.60	2.68	2.83	
☑	G1	GAPDH 24	84.74	12.37	2.22	5.57					
☑	G2	GAPDH 24	84.74	8.01	2.10	3.81					
☑	G3	GAPDH 24	84.58	9.63	2.27	4.24					
☑	G4	GAPDH 26	84.58	9.26	2.06	4.51					
☑	G5	GAPDH 26	84.45	12.08	2.27	5.33					
☑	G6	GAPDH 26	84.57	9.25	2.22	4.17					
☑	G7										
☑	G8										
☑	G9										

Results

Inc	Pos	Sample Name	Peak 1 Tm	Area	Width	Height	Peak 2 Tm	Area	Width	Height	Status
☑	G10										
☑	G11										
☑	G12										
☑	H1	FCER1A 24	75.18	3.30	2.18	1.51	79.64	6.98	2.44	2.86	
☑	H2	FCER1A 24	74.79	1.42	2.02	0.70	79.41	7.53	2.55	2.95	
☑	H3	FCER1A 24	75.84	7.85	3.42	2.29					
☑	H4	FCER1A 26	75.55	5.21	2.67	1.95	79.89	2.36	2.25	1.10	
☑	H5	FCER1A 26	75.23	6.06	2.52	2.40	79.51	4.36	2.40	1.82	
☑	H6	FCER1A 26	75.36	3.57	2.29	1.56	79.74	4.05	2.44	1.66	
☑	H7										
☑	H8										
☑	H9										
☑	H10										
☑	H11										
☑	H12										

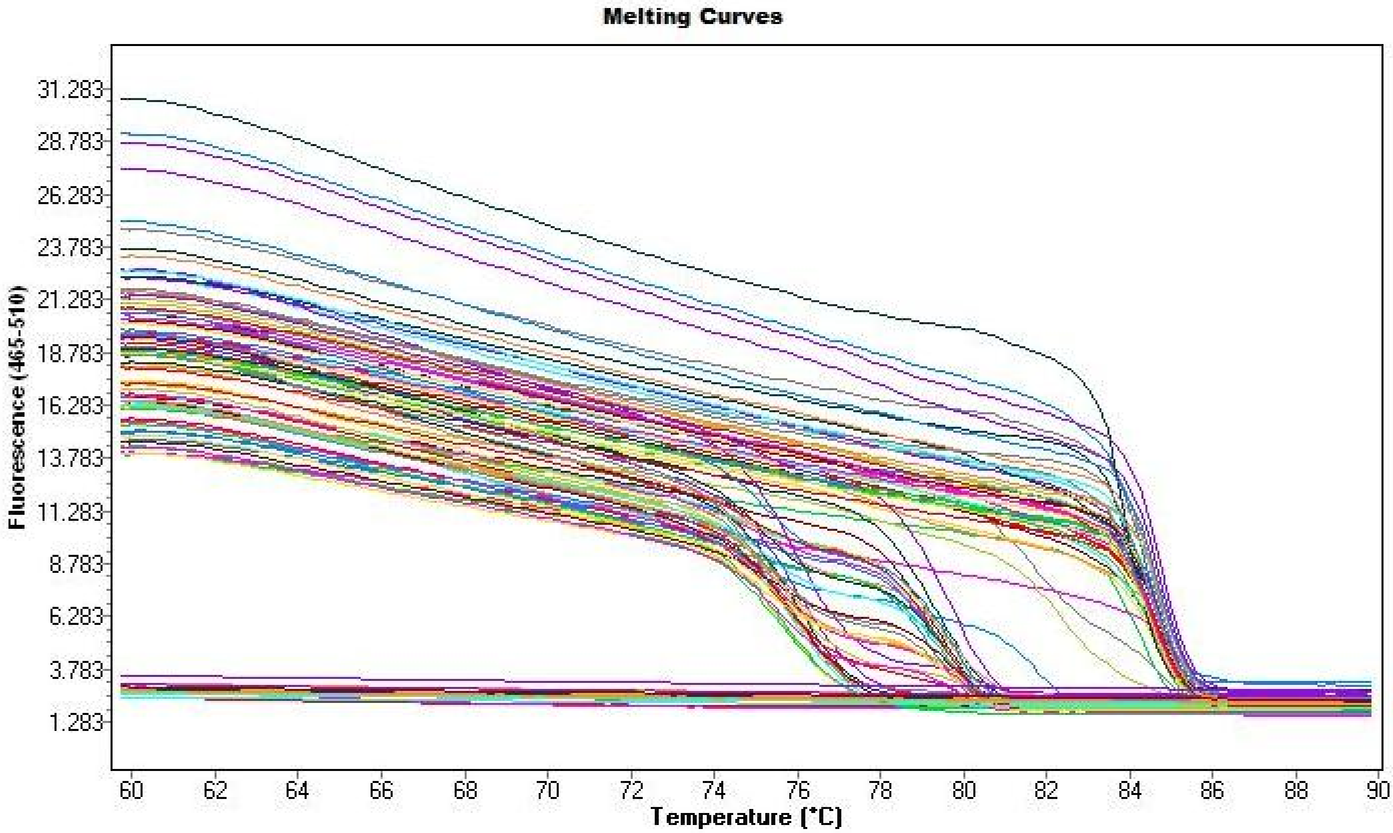

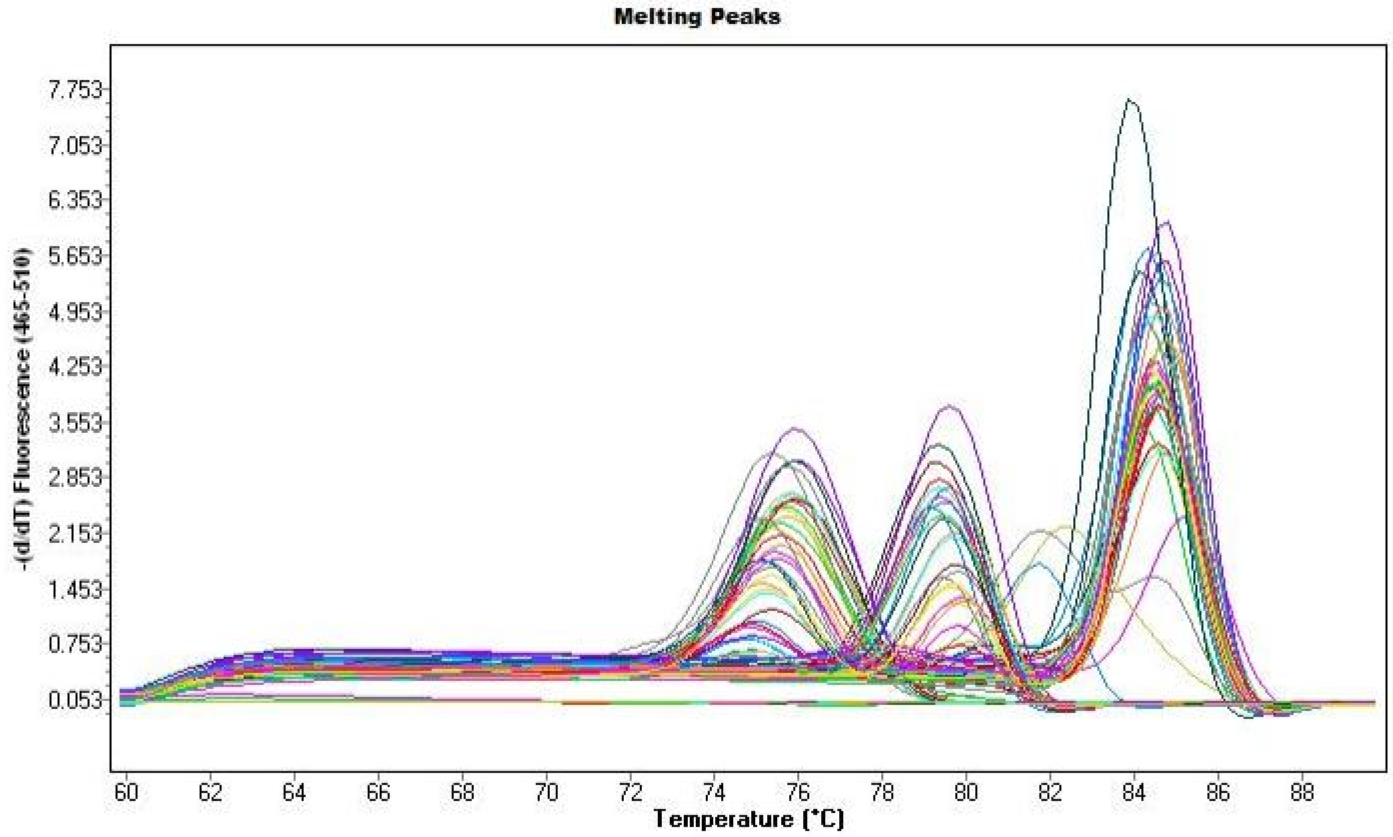

Analysis Notes	
