# Supplementary material for: Exploring differential gene expression and biomarker potential in systemic lupus erythematosus: a retrospective study
Source: PeerJ. 2025 Sep 8;13:e19891. doi: 10.7717/peerj.19891 (PMC12424612; doi:10.7717/peerj.19891)
Supplement: Supplemental Information 1 — The data include the Ct value for each sample, the Ct value for reference genes such as GAPDH, and the relative expression levels calculated using the ΔΔCt method. These data were used to validate the RNA sequencing results and further evaluate the potential of these genes as biomarkers for SLE. [file peerj-13-19891-s001.zip › qPCR/20241222 qPCR/1222plate2 HC group.pdf]

cobas® 4800 User Defined Workflow (UDF)

20241222xj plate2

Experiment

Creation Date	12/22/2024 5:28:20 PM	Last Modified Date	12/22/2024 6:42:58 PM
Operator	System Admin	Owner	System Admin
Start Time	12/22/2024 5:28:47 PM	End Time	12/22/2024 6:27:40 PM
Run State	Completed	Software Version	LCS480 1.5.1.62 SP3 - UDF 2.1.0.26
Macro		Macro Owner	
Macro Status			
Templates	gongying	Plate ID	
Test ID		Lot ID	
Color Comp ID			
Run Notes			

Programs

Program Name	Program		
Cycles	1	Analysis Mode	None

Target (°C)	Acquisition Mode	Hold (hh:mm:ss)	Ramp Rate (°C/s)	Acquisitions (per °C)	Sec Target (°C)	Step size (°C)	Step Delay (cycles)
95	None	00:00:30	4.40		0	0	0

Program Name	Program		
Cycles	40	Analysis Mode	Quantification

Target (°C)	Acquisition Mode	Hold (hh:mm:ss)	Ramp Rate (°C/s)	Acquisitions (per °C)	Sec Target (°C)	Step size (°C)	Step Delay (cycles)
95	None	00:00:15	4.40		0	0	0
60	Single	00:00:30	2.20		0	0	0

Program Name	Program		
Cycles	1	Analysis Mode	Melting Curves

Target (°C)	Acquisition Mode	Hold (hh:mm:ss)	Ramp Rate (°C/s)	Acquisitions (per °C)	Sec Target (°C)	Step size (°C)	Step Delay (cycles)
95	None	00:01:00	4.40		0	0	0
60	None	00:00:30	2.20		0	0	0
90	Continuous		0.11	5	0	0	0

Program Name	Program		
Cycles	1	Analysis Mode	None

Target (°C)	Acquisition Mode	Hold (hh:mm:ss)	Ramp Rate (°C/s)	Acquisitions (per °C)	Sec Target (°C)	Step size (°C)	Step Delay (cycles)

Target (°C)	Acquisition Mode	Hold (hh:mm:ss)	Ramp Rate (°C/s)	Acquisitions (per °C)	Sec Target (°C)	Step size (°C)	Step Delay (cycles)
40	None	00:00:30	2.20		0	0	0

Samples

Sample Count	96
Subset	All Samples

Pos	Name	ID	Repl. Of	Sample Notes
A1	GAPDH 102			
A2	GAPDH 102			
A3	GAPDH 102			
A4	GAPDH 107			
A5	GAPDH 107			
A6	GAPDH 107			
A7	GAPDH 110			
A8	GAPDH 110			
A9	GAPDH 110			
A10	GAPDH 113			
A11	GAPDH 113			
A12	GAPDH 113			
B1	FCER1A 102			
B2	FCER1A 102			
B3	FCER1A 102			
B4	FCER1A 107			
B5	FCER1A 107			
B6	FCER1A 107			
B7	FCER1A 110			
B8	FCER1A 110			
B9	FCER1A 110			
B10	FCER1A 113			
B11	FCER1A 113			
B12	FCER1A 113			
C1	GAPDH 115			
C2	GAPDH 115			
C3	GAPDH 115			
C4	GAPDH 117			
C5	GAPDH 117			
C6	GAPDH 117			
C7	GAPDH 119			
C8	GAPDH 119			
C9	GAPDH 119			
C10	GAPDH 120			
C11	GAPDH 120			
C12	GAPDH 120			

Samples

Sample Count	96
Subset	All Samples

Pos	Name	ID	Repl. Of	Sample Notes
D1	FCER1A 115			
D2	FCER1A 115			
D3	FCER1A 115			
D4	FCER1A 117			
D5	FCER1A 117			
D6	FCER1A 117			
D7	FCER1A 119			
D8	FCER1A 119			
D9	FCER1A 119			
D10	FCER1A 120			
D11	FCER1A 120			
D12	FCER1A 120			
E1				
E2				
E3				
E4				
E5				
E6				
E7				
E8				
E9				
E10				
E11				
E12				
F1				
F2				
F3				
F4				
F5				
F6				
F7				
F8				
F9				
F10				
F11				
F12				
G1				
G2				
G3				

Samples

Sample Count	96
Subset	All Samples

Pos	Name	ID	Repl. Of	Sample Notes
G4				
G5				
G6				
G7				
G8				
G9				
G10				
G11				
G12				
H1				
H2				
H3				
H4				
H5				
H6				
H7				
H8				
H9				
H10				
H11				
H12				

Instrument

Name	Z480	Serial Number	56319

Revision History

Revision	Date	User	Reason

Abs Quant/2nd Derivative Max for All Samples (Abs Quant/2nd Derivative Max)

Settings

Channel	465-510		
Color Compensation	Off		
Program	Program	Units	
Mode	High Confidence		

Subset Name	All Samples

Results

Inc	Pos	Name	Type	CP	Concentration	Standard	Status

Results

Inc	Pos	Name	Type	CP	Concentration	Standard	Status
☑	A1	GAPDH 102	Unknown	24.09			
☑	A2	GAPDH 102	Unknown	24.30			
☑	A3	GAPDH 102	Unknown	24.18			
☑	A4	GAPDH 107	Unknown	23.78			
☑	A5	GAPDH 107	Unknown	23.74			
☑	A6	GAPDH 107	Unknown	23.77			
☑	A7	GAPDH 110	Unknown	22.98			
☑	A8	GAPDH 110	Unknown	22.83			
☑	A9	GAPDH 110	Unknown	22.95			
☑	A10	GAPDH 113	Unknown	27.28			
☑	A11	GAPDH 113	Unknown	27.18			
☑	A12	GAPDH 113	Unknown	27.71			
☑	B1	FCER1A 102	Unknown	30.79			
☑	B2	FCER1A 102	Unknown	30.75			
☑	B3	FCER1A 102	Unknown	30.36			
☑	B4	FCER1A 107	Unknown	29.05			
☑	B5	FCER1A 107	Unknown	28.92			
☑	B6	FCER1A 107	Unknown	28.96			
☑	B7	FCER1A 110	Unknown	30.57			
☑	B8	FCER1A 110	Unknown	30.16			
☑	B9	FCER1A 110	Unknown	29.87			
☑	B10	FCER1A 113	Unknown	26.43			
☑	B11	FCER1A 113	Unknown	30.78			
☑	B12	FCER1A 113	Unknown	31.87			
☑	C1	GAPDH 115	Unknown	23.28			
☑	C2	GAPDH 115	Unknown	23.52			
☑	C3	GAPDH 115	Unknown	23.31			
☑	C4	GAPDH 117	Unknown	22.79			
☑	C5	GAPDH 117	Unknown	22.88			
☑	C6	GAPDH 117	Unknown	22.75			
☑	C7	GAPDH 119	Unknown	23.79			
☑	C8	GAPDH 119	Unknown	23.66			
☑	C9	GAPDH 119	Unknown	23.55			
☑	C10	GAPDH 120	Unknown	24.54			
☑	C11	GAPDH 120	Unknown	24.34			
☑	C12	GAPDH 120	Unknown	24.44			
☑	D1	FCER1A 115	Unknown	29.64			

Results

Inc	Pos	Name	Type	CP	Concentration	Standard	Status
☑	D2	FCER1A 115	Unknown	29.75			
☑	D3	FCER1A 115	Unknown	28.10			
☑	D4	FCER1A 117	Unknown	28.86			
☑	D5	FCER1A 117	Unknown	28.72			
☑	D6	FCER1A 117	Unknown	28.07			
☑	D7	FCER1A 119	Unknown	29.21			
☑	D8	FCER1A 119	Unknown	29.36			
☑	D9	FCER1A 119	Unknown	29.31			
☑	D10	FCER1A 120	Unknown	29.51			
☑	D11	FCER1A 120	Unknown	29.44			
☑	D12	FCER1A 120	Unknown	29.45			
☑	E1		Unknown				
☑	E2		Unknown				
☑	E3		Unknown				
☑	E4		Unknown				
☑	E5		Unknown				
☑	E6		Unknown				
☑	E7		Unknown				
☑	E8		Unknown				
☑	E9		Unknown				
☑	E10		Unknown				
☑	E11		Unknown				
☑	E12		Unknown				
☑	F1		Unknown				
☑	F2		Unknown				
☑	F3		Unknown				
☑	F4		Unknown				
☑	F5		Unknown				
☑	F6		Unknown				
☑	F7		Unknown				
☑	F8		Unknown				
☑	F9		Unknown				
☑	F10		Unknown				
☑	F11		Unknown				
☑	F12		Unknown				
☑	G1		Unknown				
☑	G2		Unknown				

Results

Inc	Pos	Name	Type	CP	Concentration	Standard	Status
☑	G3		Unknown				
☑	G4		Unknown				
☑	G5		Unknown				
☑	G6		Unknown				
☑	G7		Unknown				
☑	G8		Unknown				
☑	G9		Unknown				
☑	G10		Unknown				
☑	G11		Unknown				
☑	G12		Unknown				
☑	H1		Unknown				
☑	H2		Unknown				
☑	H3		Unknown				
☑	H4		Unknown				
☑	H5		Unknown				
☑	H6		Unknown				
☑	H7		Unknown				
☑	H8		Unknown				
☑	H9		Unknown				
☑	H10		Unknown				
☑	H11		Unknown				
☑	H12		Unknown				

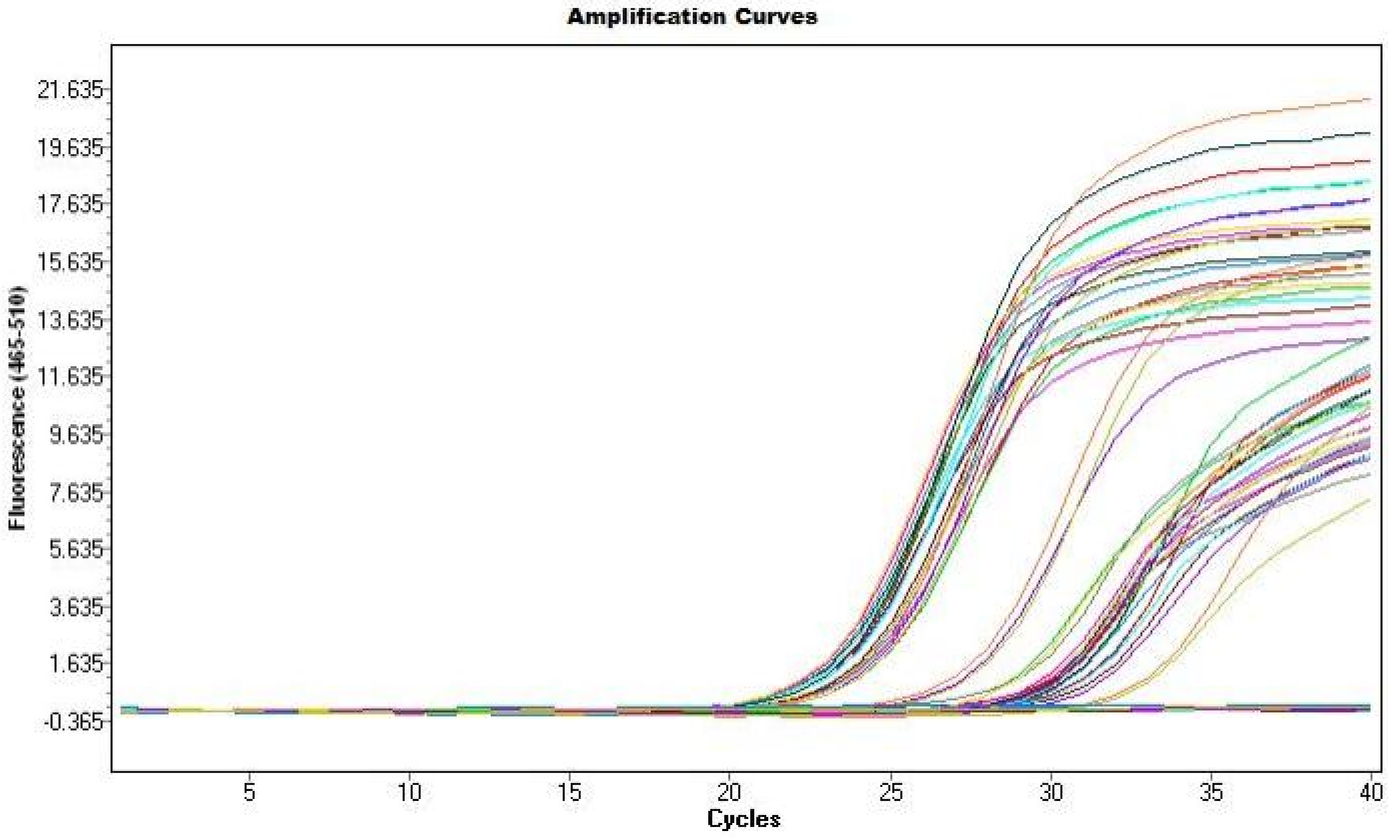

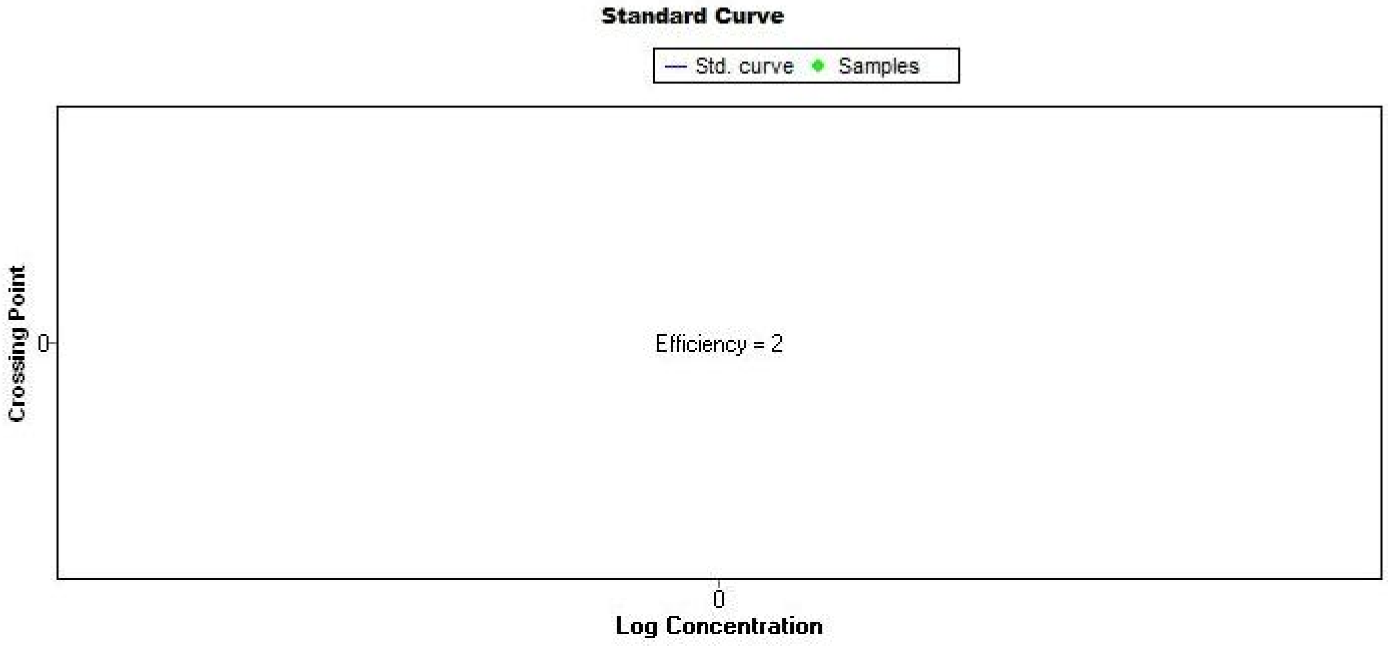

Analysis Notes	

Abs Quant/Fit Points for All Samples (Abs Quant/Fit Points)

Settings

Channel	465-510				
Color Compensation	Off				
Standard Curve	Set Efficiency (2.00)				
Program	Program			Units	
First Cycle	1	Last Cycle	40	Background	2-6
Noiseband Method	Auto	Noiseband	0.5250	STD Dev Multiplier	12.0000
# of Fit Points	2	Threshold Method	Auto	Threshold	0.5250

Subset Name	All Samples

Results

Inc	Pos	Name	Type	CP	Concentration	Standard	Status
☑	A1	GAPDH 102	Unknown	21.37			
☑	A2	GAPDH 102	Unknown	21.58			
☑	A3	GAPDH 102	Unknown	21.69			
☑	A4	GAPDH 107	Unknown	20.96			
☑	A5	GAPDH 107	Unknown	20.87			
☑	A6	GAPDH 107	Unknown	20.93			
☑	A7	GAPDH 110	Unknown	20.54			
☑	A8	GAPDH 110	Unknown	19.96			
☑	A9	GAPDH 110	Unknown	20.16			
☑	A10	GAPDH 113	Unknown	24.26			
☑	A11	GAPDH 113	Unknown	25.13			
☑	A12	GAPDH 113	Unknown	25.37			
☑	B1	FCER1A 102	Unknown	28.68			
☑	B2	FCER1A 102	Unknown	28.85			
☑	B3	FCER1A 102	Unknown	28.35			
☑	B4	FCER1A 107	Unknown	27.84			
☑	B5	FCER1A 107	Unknown	27.87			
☑	B6	FCER1A 107	Unknown	27.79			
☑	B7	FCER1A 110	Unknown	28.99			
☑	B8	FCER1A 110	Unknown	28.86			
☑	B9	FCER1A 110	Unknown	28.50			
☑	B10	FCER1A 113	Unknown	30.97			
☑	B11	FCER1A 113	Unknown	29.74			
☑	B12	FCER1A 113	Unknown	31.31			
☑	C1	GAPDH 115	Unknown	20.57			
☑	C2	GAPDH 115	Unknown	20.60			
☑	C3	GAPDH 115	Unknown	20.70			

Results

Inc	Pos	Name	Type	CP	Concentration	Standard	Status
☑	C4	GAPDH 117	Unknown	19.94			
☑	C5	GAPDH 117	Unknown	20.36			
☑	C6	GAPDH 117	Unknown	19.95			
☑	C7	GAPDH 119	Unknown	20.98			
☑	C8	GAPDH 119	Unknown	20.78			
☑	C9	GAPDH 119	Unknown	20.47			
☑	C10	GAPDH 120	Unknown	21.40			
☑	C11	GAPDH 120	Unknown	21.58			
☑	C12	GAPDH 120	Unknown	21.76			
☑	D1	FCER1A 115	Unknown	28.86			
☑	D2	FCER1A 115	Unknown	26.95			
☑	D3	FCER1A 115	Unknown	26.95			
☑	D4	FCER1A 117	Unknown	27.57			
☑	D5	FCER1A 117	Unknown	25.08			
☑	D6	FCER1A 117	Unknown	27.05			
☑	D7	FCER1A 119	Unknown	28.36			
☑	D8	FCER1A 119	Unknown	28.15			
☑	D9	FCER1A 119	Unknown	28.22			
☑	D10	FCER1A 120	Unknown	28.49			
☑	D11	FCER1A 120	Unknown	28.39			
☑	D12	FCER1A 120	Unknown	28.62			
☑	E1		Unknown				
☑	E2		Unknown				
☑	E3		Unknown				
☑	E4		Unknown				
☑	E5		Unknown				
☑	E6		Unknown				
☑	E7		Unknown				
☑	E8		Unknown				
☑	E9		Unknown				
☑	E10		Unknown				
☑	E11		Unknown				
☑	E12		Unknown				
☑	F1		Unknown				
☑	F2		Unknown				
☑	F3		Unknown				
☑	F4		Unknown				

Results

Inc	Pos	Name	Type	CP	Concentration	Standard	Status
☑	F5		Unknown				
☑	F6		Unknown				
☑	F7		Unknown				
☑	F8		Unknown				
☑	F9		Unknown				
☑	F10		Unknown				
☑	F11		Unknown				
☑	F12		Unknown				
☑	G1		Unknown				
☑	G2		Unknown				
☑	G3		Unknown				
☑	G4		Unknown				
☑	G5		Unknown				
☑	G6		Unknown				
☑	G7		Unknown				
☑	G8		Unknown				
☑	G9		Unknown				
☑	G10		Unknown				
☑	G11		Unknown				
☑	G12		Unknown				
☑	H1		Unknown				
☑	H2		Unknown				
☑	H3		Unknown				
☑	H4		Unknown				
☑	H5		Unknown				
☑	H6		Unknown				
☑	H7		Unknown				
☑	H8		Unknown				
☑	H9		Unknown				
☑	H10		Unknown				
☑	H11		Unknown				
☑	H12		Unknown				

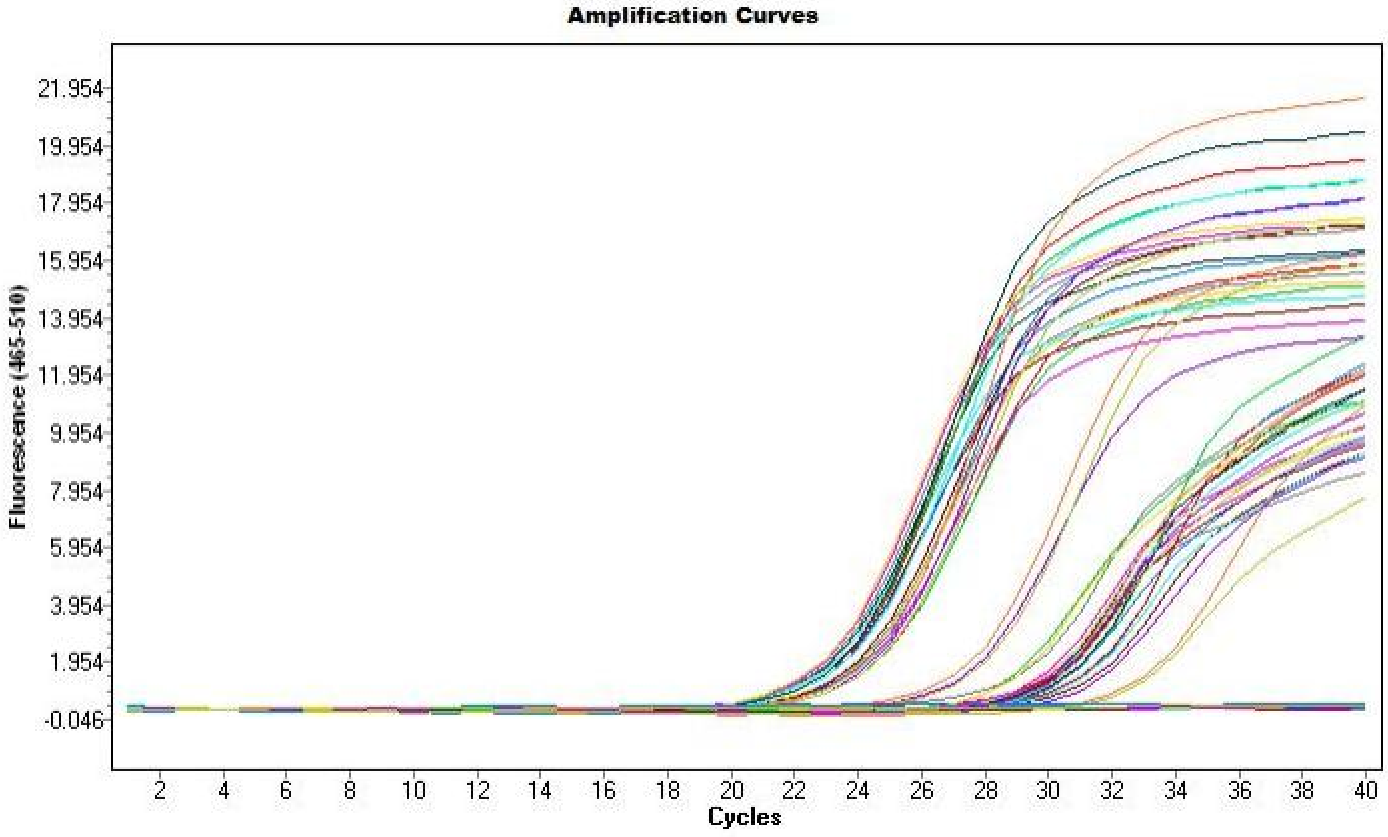
Amplification Curves
Fluorescence (465-510)
21.954
19.954
17.954
15.954
13.954
11.954
9.954
7.954
5.954
3.954
1.954
-0.046
2 4 6 8 10 12 14 16 18 20 22 24 26 28 30 32 34 36 38 40
Cycles

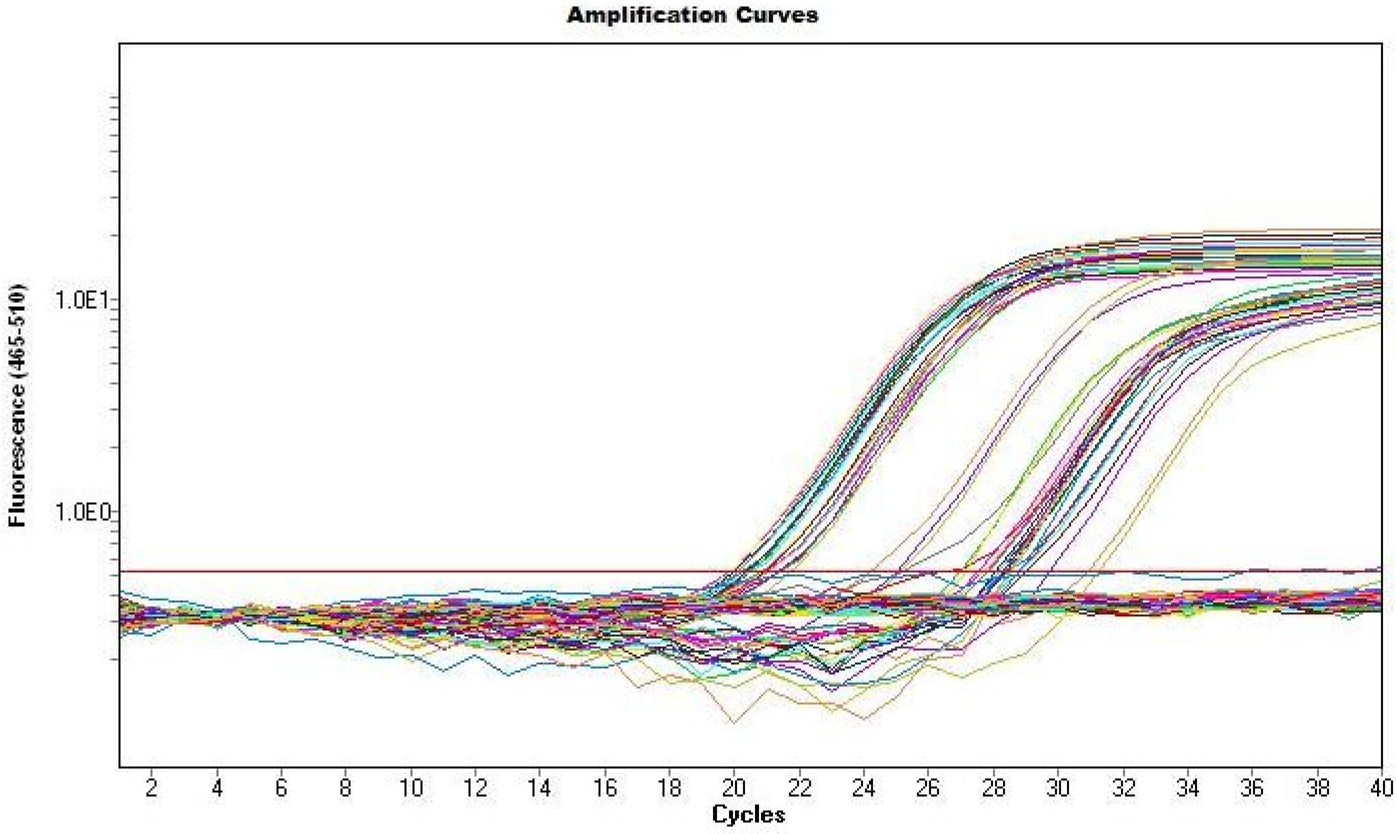
Amplification Curves
Fluorescence (465-510)
1.0E1
1.0E0
2 4 6 8 10 12 14 16 18 20 22 24 26 28 30 32 34 36 38 40
Cycles

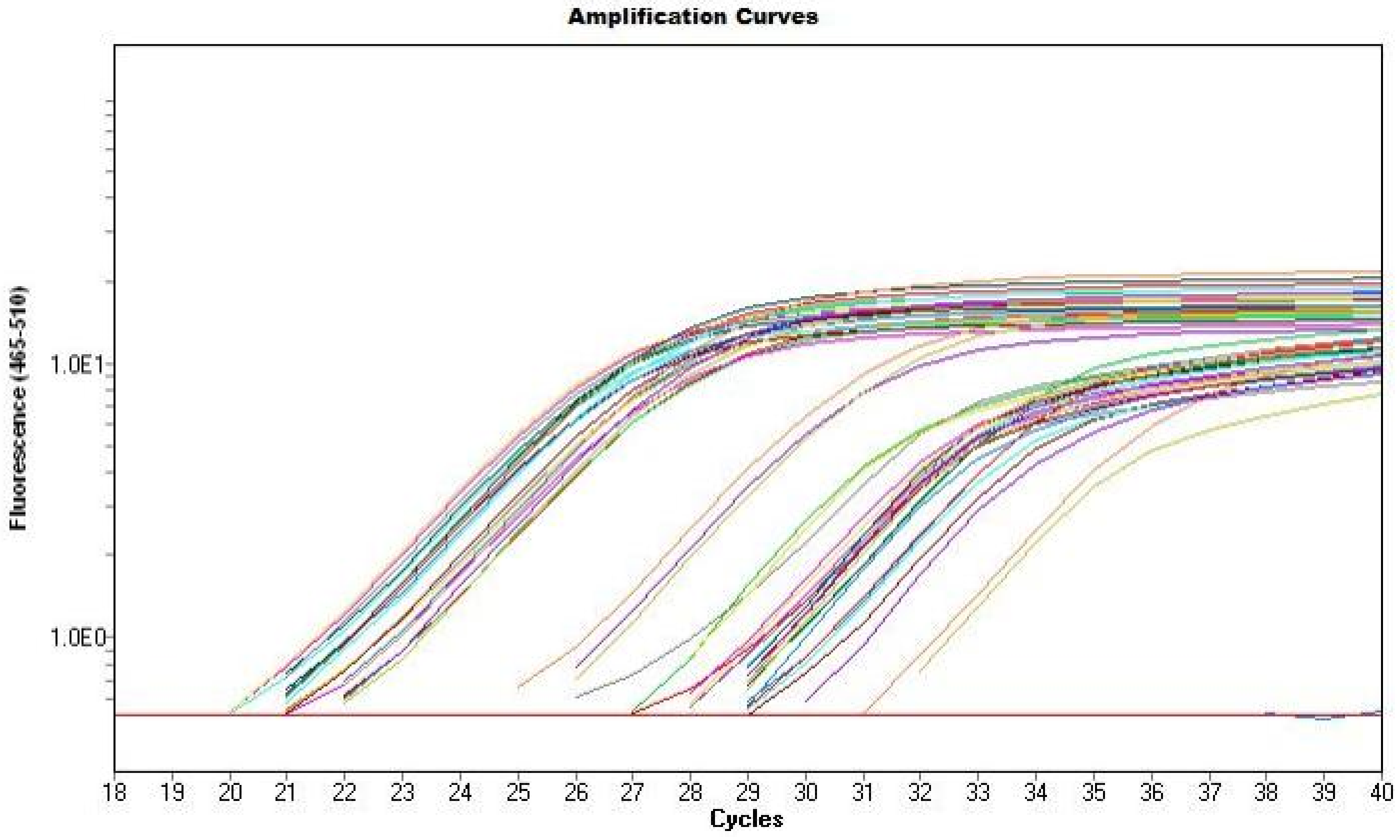

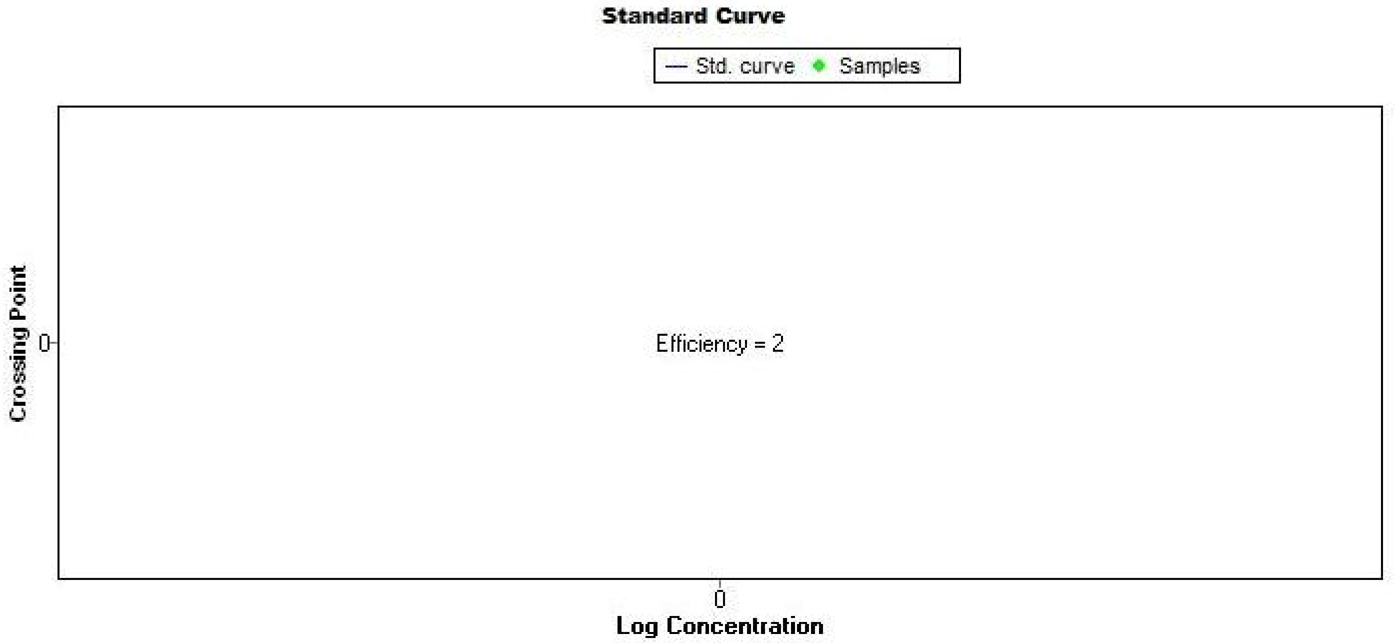

Analysis Notes	

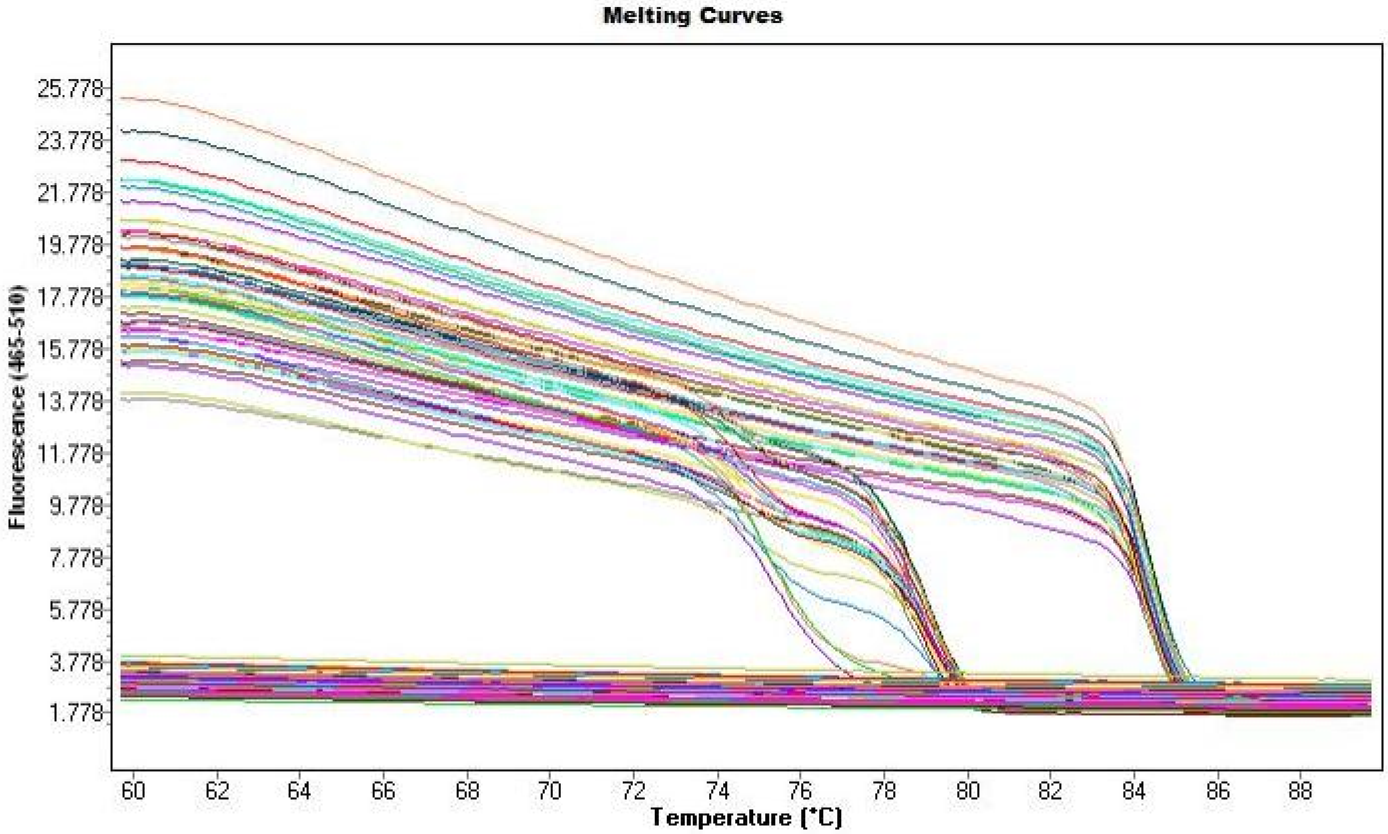

Melting Curves
Fluorescence (465-510)
25.778
23.778
21.778
19.778
17.778
15.778
13.778
11.778
9.778
7.778
5.778
3.778
1.778
60
62
64
66
68
70
72
74
76
78
80
82
84
86
88
Temperature (°C)

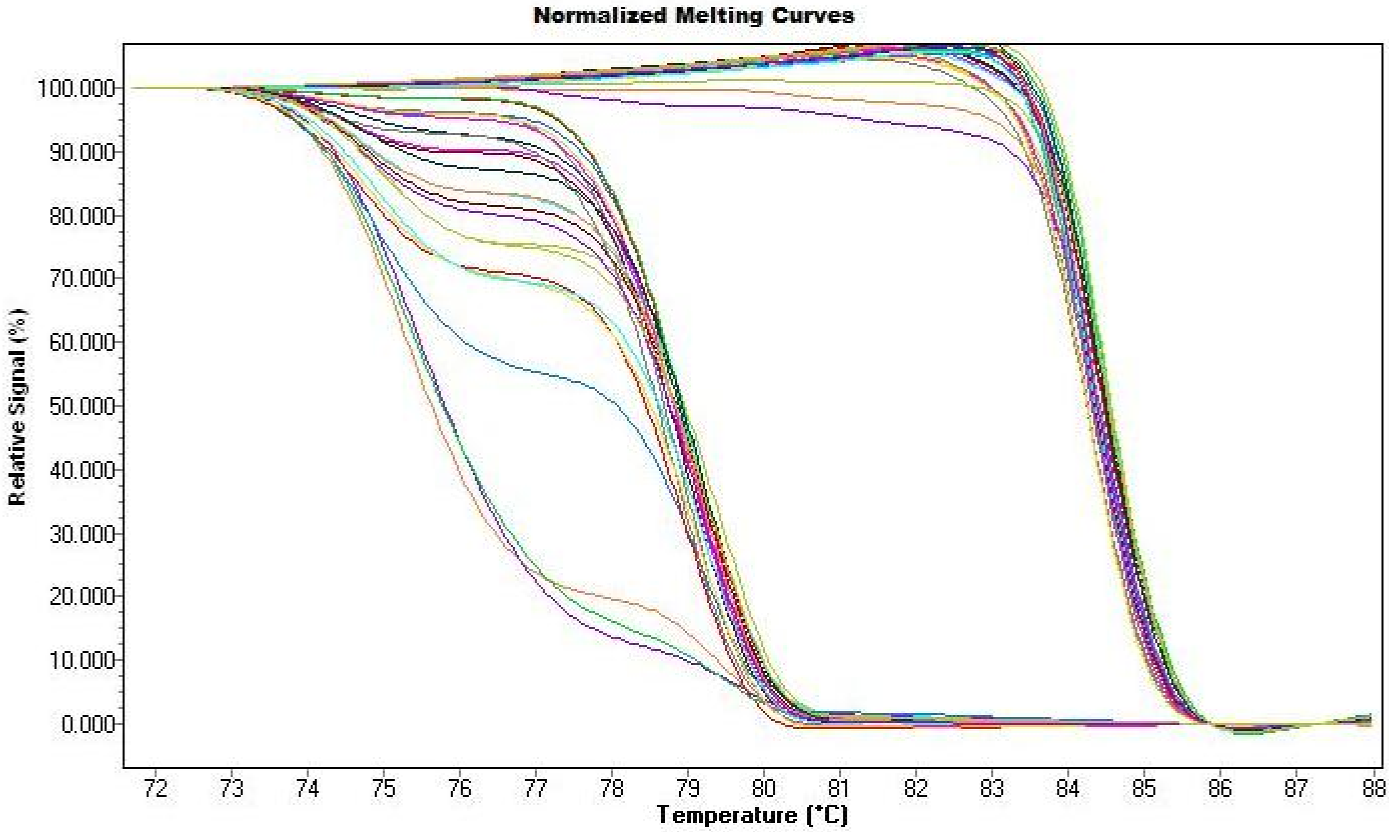

Normalized Melting Curves
Relative Signal (%)
100.000
90.000
80.000
70.000
60.000
50.000
40.000
30.000
20.000
10.000
0.000
72
73
74
75
76
77
78
79
80
81
82
83
84
85
86
87
88
Temperature (°C)

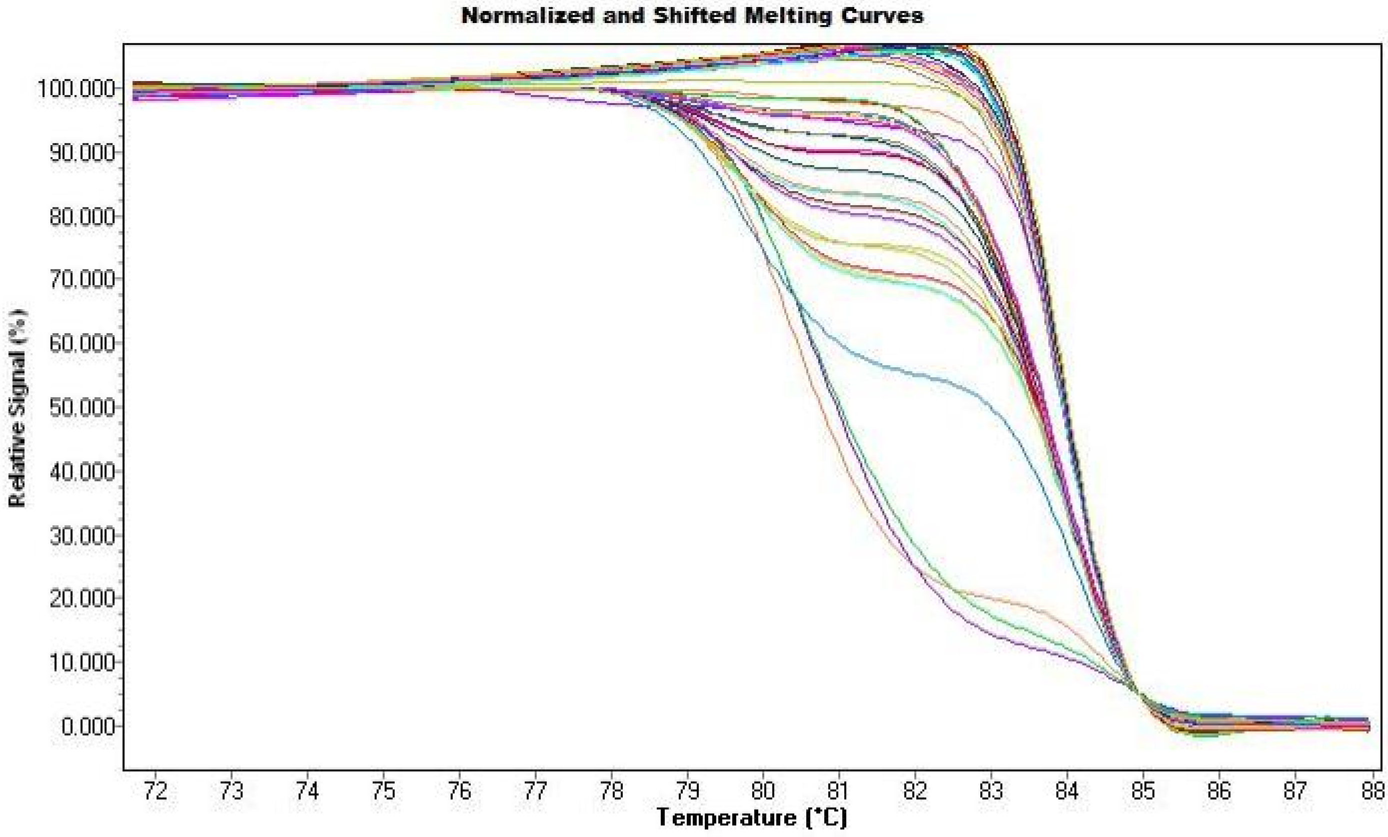

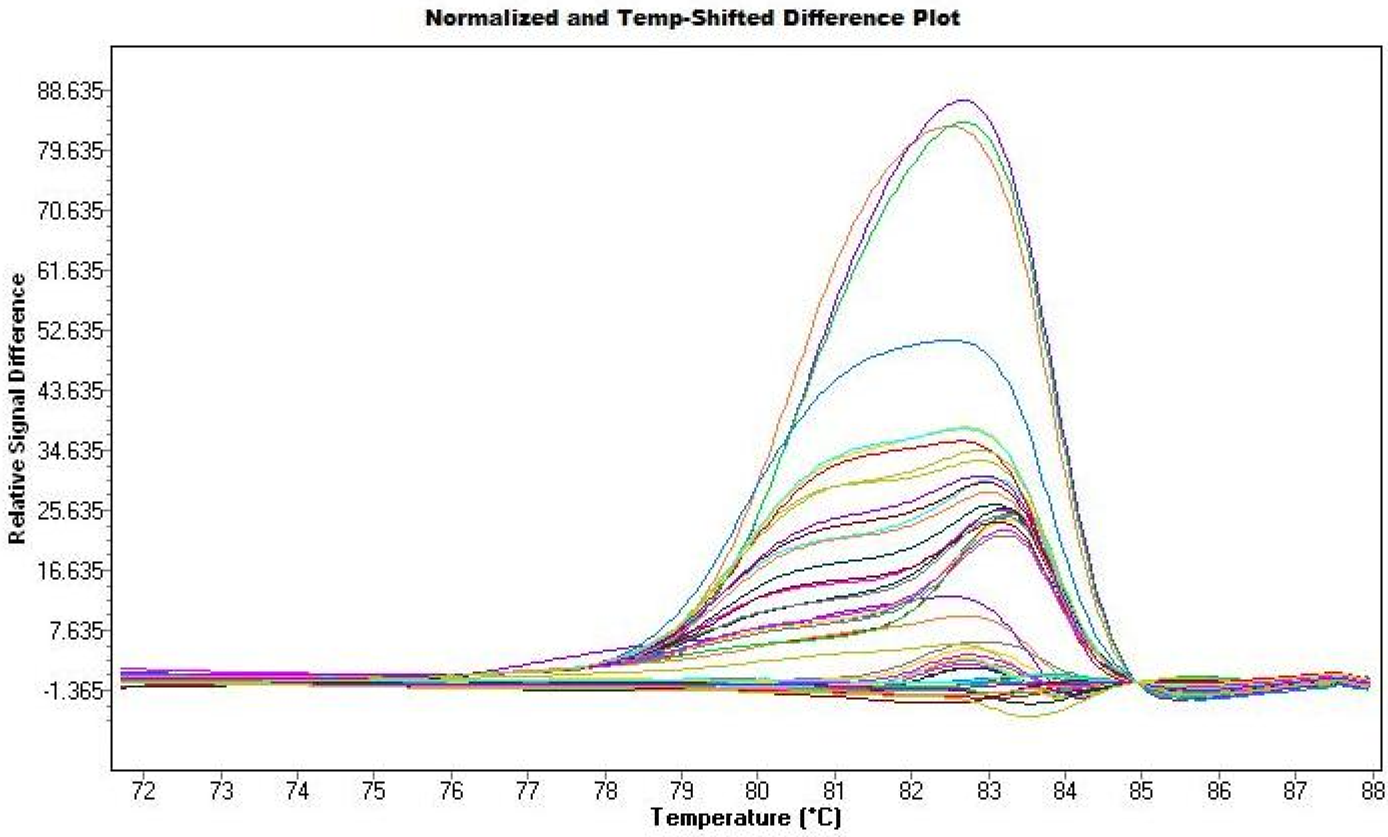

**Analysis Notes**	

Melt Curve Genotyping for All Samples (Melt Curve Genotyping)

Channel	465-510	Color Compensation	Off				
Progam Name	Program						
Stds Settings	Auto-Group						
Sensitivity	Normal	Temp Range	63-85	Score	0.7	Res	0.1

Subset Name	All Samples

Results

Inc	Pos.	Sample Name	Sample Type	Group	Score	Resolution	Status
☑	A1	GAPDH 102	Unknown	1	0.99	0.91	
☑	A2	GAPDH 102	Unknown	1	0.99	0.91	
☑	A3	GAPDH 102	Unknown	1	0.99	0.91	
☑	A4	GAPDH 107	Unknown	1	0.99	0.91	
☑	A5	GAPDH 107	Unknown	1	0.99	0.93	
☑	A6	GAPDH 107	Unknown	1	0.99	0.92	
☑	A7	GAPDH 110	Unknown	1	0.99	0.92	
☑	A8	GAPDH 110	Unknown	1	0.99	0.91	
☑	A9	GAPDH 110	Unknown	1	0.99	0.90	
☑	A10	GAPDH 113	Unknown	1	0.97	0.88	
☑	A11	GAPDH 113	Unknown	1	0.95	0.84	
☑	A12	GAPDH 113	Unknown	1	0.99	0.92	
☑	B1	FCER1A 102	Unknown	2	0.89	0.34	
☑	B2	FCER1A 102	Unknown	2	0.82	0.33	
☑	B3	FCER1A 102	Unknown	2	0.83	0.33	
☑	B4	FCER1A 107	Unknown	2	0.98	0.30	
☑	B5	FCER1A 107	Unknown	2	0.85	0.23	
☑	B6	FCER1A 107	Unknown	2	0.88	0.30	
☑	B7	FCER1A 110	Unknown	2	0.97	0.27	
☑	B8	FCER1A 110	Unknown	2	0.98	0.29	
☑	B9	FCER1A 110	Unknown	2	0.93	0.33	
☑	B10	FCER1A 113	Unknown	3	0.98	0.40	
☑	B11	FCER1A 113	Unknown	3	0.99	0.30	
☑	B12	FCER1A 113	Unknown	2	0.88	0.25	
☑	C1	GAPDH 115	Unknown	1	0.99	0.92	
☑	C2	GAPDH 115	Unknown	1	0.99	0.93	
☑	C3	GAPDH 115	Unknown	1	0.99	0.93	
☑	C4	GAPDH 117	Unknown	1	0.99	0.93	
☑	C5	GAPDH 117	Unknown	1	0.99	0.93	
☑	C6	GAPDH 117	Unknown	1	0.99	0.92	
☑	C7	GAPDH 119	Unknown	1	0.99	0.93	

Results

Inc	Pos.	Sample Name	Sample Type	Group	Score	Resolution	Status
☑	C8	GAPDH 119	Unknown	1	0.99	0.93	
☑	C9	GAPDH 119	Unknown	1	0.99	0.91	
☑	C10	GAPDH 120	Unknown	1	0.99	0.92	
☑	C11	GAPDH 120	Unknown	1	0.99	0.92	
☑	C12	GAPDH 120	Unknown	1	1.00	0.91	
☑	D1	FCER1A 115	Unknown	Unknown	0.67	0.10	
☑	D2	FCER1A 115	Unknown	2	0.81	0.18	
☑	D3	FCER1A 115	Unknown	3	1.00	0.33	
☑	D4	FCER1A 117	Unknown	2	0.88	0.30	
☑	D5	FCER1A 117	Unknown	2	0.93	0.20	
☑	D6	FCER1A 117	Unknown	2	0.79	0.16	
☑	D7	FCER1A 119	Unknown	2	0.96	0.27	
☑	D8	FCER1A 119	Unknown	2	0.80	0.17	
☑	D9	FCER1A 119	Unknown	2	1.00	0.33	
☑	D10	FCER1A 120	Unknown	2	0.98	0.27	
☑	D11	FCER1A 120	Unknown	2	0.94	0.22	
☑	D12	FCER1A 120	Unknown	2	0.89	0.22	
☑	E1		Unknown	Negative	1.00	1.00	
☑	E2		Unknown	Negative	1.00	1.00	
☑	E3		Unknown	Negative	1.00	1.00	
☑	E4		Unknown	Negative	1.00	1.00	
☑	E5		Unknown	Negative	1.00	1.00	
☑	E6		Unknown	Negative	1.00	1.00	
☑	E7		Unknown	Negative	1.00	1.00	
☑	E8		Unknown	Negative	1.00	1.00	
☑	E9		Unknown	Negative	1.00	1.00	
☑	E10		Unknown	Negative	1.00	1.00	
☑	E11		Unknown	Negative	1.00	1.00	
☑	E12		Unknown	Negative	1.00	1.00	
☑	F1		Unknown	Negative	1.00	1.00	
☑	F2		Unknown	Negative	1.00	1.00	
☑	F3		Unknown	Negative	1.00	1.00	
☑	F4		Unknown	Negative	1.00	1.00	
☑	F5		Unknown	Negative	1.00	1.00	
☑	F6		Unknown	Negative	1.00	1.00	
☑	F7		Unknown	Negative	1.00	1.00	
☑	F8		Unknown	Negative	1.00	1.00	
☑	F9		Unknown	Negative	1.00	1.00	

Results

Inc	Pos.	Sample Name	Sample Type	Group	Score	Resolution	Status
☑	F10		Unknown	Negative	1.00	1.00	
☑	F11		Unknown	Negative	1.00	1.00	
☑	F12		Unknown	Negative	1.00	1.00	
☑	G1		Unknown	Negative	1.00	1.00	
☑	G2		Unknown	Negative	1.00	1.00	
☑	G3		Unknown	Negative	1.00	1.00	
☑	G4		Unknown	Negative	1.00	1.00	
☑	G5		Unknown	Negative	1.00	1.00	
☑	G6		Unknown	Negative	1.00	1.00	
☑	G7		Unknown	Negative	1.00	1.00	
☑	G8		Unknown	Negative	1.00	1.00	
☑	G9		Unknown	Negative	1.00	1.00	
☑	G10		Unknown	Negative	1.00	1.00	
☑	G11		Unknown	Negative	1.00	1.00	
☑	G12		Unknown	Negative	1.00	1.00	
☑	H1		Unknown	Negative	1.00	1.00	
☑	H2		Unknown	Negative	1.00	1.00	
☑	H3		Unknown	Negative	1.00	1.00	
☑	H4		Unknown	Negative	1.00	1.00	
☑	H5		Unknown	Negative	1.00	1.00	
☑	H6		Unknown	Negative	1.00	1.00	
☑	H7		Unknown	Negative	1.00	1.00	
☑	H8		Unknown	Negative	1.00	1.00	
☑	H9		Unknown	Negative	1.00	1.00	
☑	H10		Unknown	Negative	1.00	1.00	
☑	H11		Unknown	Negative	1.00	1.00	
☑	H12		Unknown	Negative	1.00	1.00	

**Group**	1

Inc	Pos	Sample Name	Sample Type	Score	Resolution	Status
☑	A1	GAPDH 102	Unknown	0.99	0.91	
☑	A2	GAPDH 102	Unknown	0.99	0.91	
☑	A3	GAPDH 102	Unknown	0.99	0.91	
☑	A4	GAPDH 107	Unknown	0.99	0.91	
☑	A5	GAPDH 107	Unknown	0.99	0.93	
☑	A6	GAPDH 107	Unknown	0.99	0.92	
☑	A7	GAPDH 110	Unknown	0.99	0.92	
☑	A8	GAPDH 110	Unknown	0.99	0.91	

**Group**	1

Inc	Pos	Sample Name	Sample Type	Score	Resolution	Status
☑	A9	GAPDH 110	Unknown	0.99	0.90	
☑	A10	GAPDH 113	Unknown	0.97	0.88	
☑	A11	GAPDH 113	Unknown	0.95	0.84	
☑	A12	GAPDH 113	Unknown	0.99	0.92	
☑	C1	GAPDH 115	Unknown	0.99	0.92	
☑	C2	GAPDH 115	Unknown	0.99	0.93	
☑	C3	GAPDH 115	Unknown	0.99	0.93	
☑	C4	GAPDH 117	Unknown	0.99	0.93	
☑	C5	GAPDH 117	Unknown	0.99	0.93	
☑	C6	GAPDH 117	Unknown	0.99	0.92	
☑	C7	GAPDH 119	Unknown	0.99	0.93	
☑	C8	GAPDH 119	Unknown	0.99	0.93	
☑	C9	GAPDH 119	Unknown	0.99	0.91	
☑	C10	GAPDH 120	Unknown	0.99	0.92	
☑	C11	GAPDH 120	Unknown	0.99	0.92	
☑	C12	GAPDH 120	Unknown	1.00	0.91	

**Group**	2

Inc	Pos	Sample Name	Sample Type	Score	Resolution	Status
☑	B1	FCER1A 102	Unknown	0.89	0.34	
☑	B2	FCER1A 102	Unknown	0.82	0.33	
☑	B3	FCER1A 102	Unknown	0.83	0.33	
☑	B4	FCER1A 107	Unknown	0.98	0.30	
☑	B5	FCER1A 107	Unknown	0.85	0.23	
☑	B6	FCER1A 107	Unknown	0.88	0.30	
☑	B7	FCER1A 110	Unknown	0.97	0.27	
☑	B8	FCER1A 110	Unknown	0.98	0.29	
☑	B9	FCER1A 110	Unknown	0.93	0.33	
☑	B12	FCER1A 113	Unknown	0.88	0.25	
☑	D2	FCER1A 115	Unknown	0.81	0.18	
☑	D4	FCER1A 117	Unknown	0.88	0.30	
☑	D5	FCER1A 117	Unknown	0.93	0.20	
☑	D6	FCER1A 117	Unknown	0.79	0.16	
☑	D7	FCER1A 119	Unknown	0.96	0.27	
☑	D8	FCER1A 119	Unknown	0.80	0.17	
☑	D9	FCER1A 119	Unknown	1.00	0.33	
☑	D10	FCER1A 120	Unknown	0.98	0.27	
☑	D11	FCER1A 120	Unknown	0.94	0.22	

Group	2

Inc	Pos	Sample Name	Sample Type	Score	Resolution	Status
☑	D12	FCER1A 120	Unknown	0.89	0.22	

Group	3

Inc	Pos	Sample Name	Sample Type	Score	Resolution	Status
☑	B10	FCER1A 113	Unknown	0.98	0.40	
☑	B11	FCER1A 113	Unknown	0.99	0.30	
☑	D3	FCER1A 115	Unknown	1.00	0.33	

Group	Negative

Inc	Pos	Sample Name	Sample Type	Score	Resolution	Status
☑	E1		Unknown	1.00	1.00	
☑	E2		Unknown	1.00	1.00	
☑	E3		Unknown	1.00	1.00	
☑	E4		Unknown	1.00	1.00	
☑	E5		Unknown	1.00	1.00	
☑	E6		Unknown	1.00	1.00	
☑	E7		Unknown	1.00	1.00	
☑	E8		Unknown	1.00	1.00	
☑	E9		Unknown	1.00	1.00	
☑	E10		Unknown	1.00	1.00	
☑	E11		Unknown	1.00	1.00	
☑	E12		Unknown	1.00	1.00	
☑	F1		Unknown	1.00	1.00	
☑	F2		Unknown	1.00	1.00	
☑	F3		Unknown	1.00	1.00	
☑	F4		Unknown	1.00	1.00	
☑	F5		Unknown	1.00	1.00	
☑	F6		Unknown	1.00	1.00	
☑	F7		Unknown	1.00	1.00	
☑	F8		Unknown	1.00	1.00	
☑	F9		Unknown	1.00	1.00	
☑	F10		Unknown	1.00	1.00	
☑	F11		Unknown	1.00	1.00	
☑	F12		Unknown	1.00	1.00	
☑	G1		Unknown	1.00	1.00	
☑	G2		Unknown	1.00	1.00	
☑	G3		Unknown	1.00	1.00	
☑	G4		Unknown	1.00	1.00	

Group	Negative

Inc	Pos	Sample Name	Sample Type	Score	Resolution	Status
☑	G5		Unknown	1.00	1.00	
☑	G6		Unknown	1.00	1.00	
☑	G7		Unknown	1.00	1.00	
☑	G8		Unknown	1.00	1.00	
☑	G9		Unknown	1.00	1.00	
☑	G10		Unknown	1.00	1.00	
☑	G11		Unknown	1.00	1.00	
☑	G12		Unknown	1.00	1.00	
☑	H1		Unknown	1.00	1.00	
☑	H2		Unknown	1.00	1.00	
☑	H3		Unknown	1.00	1.00	
☑	H4		Unknown	1.00	1.00	
☑	H5		Unknown	1.00	1.00	
☑	H6		Unknown	1.00	1.00	
☑	H7		Unknown	1.00	1.00	
☑	H8		Unknown	1.00	1.00	
☑	H9		Unknown	1.00	1.00	
☑	H10		Unknown	1.00	1.00	
☑	H11		Unknown	1.00	1.00	
☑	H12		Unknown	1.00	1.00	

Group	Unknown

Inc	Pos	Sample Name	Sample Type	Score	Resolution	Status
☑	D1	FCER1A 115	Unknown	0.67	0.10	

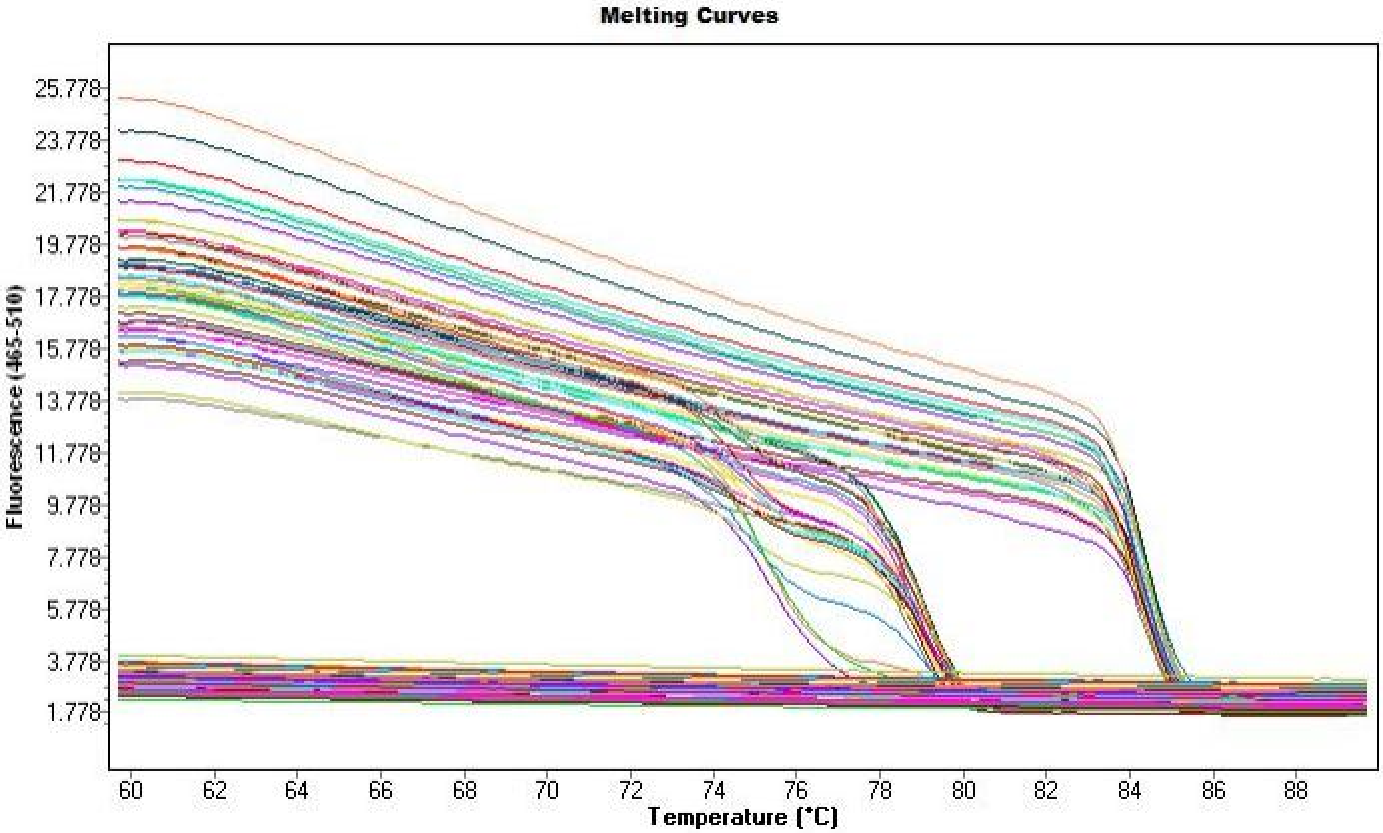

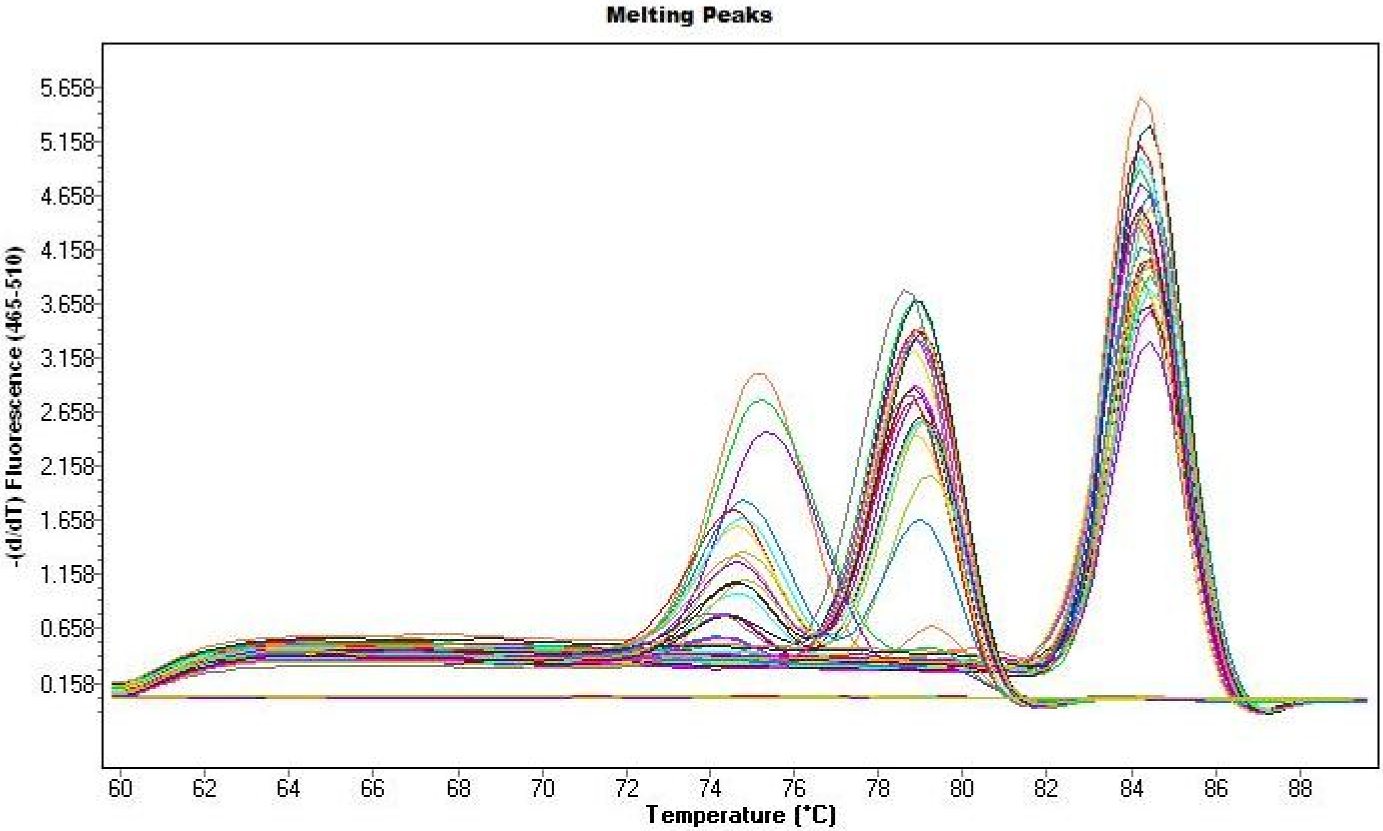

Analysis Notes	

Tm Calling for All Samples (Tm Calling)

Settings

Channel	465-510	Color Compensation	Off		
Program	Program				
Show Shoulders	False	Chemistry	HybProbe	Max Peaks	2

Subset Name	All Samples

Results

			Peak 1				Peak 2				
Inc	Pos	Sample Name	Tm	Area	Width	Height	Tm	Area	Width	Height	Status
☑	A1	GAPDH 102	84.55	11.02	2.27	4.86					
☑	A2	GAPDH 102	84.45	9.44	2.19	4.30					
☑	A3	GAPDH 102	84.53	8.80	2.18	4.04					
☑	A4	GAPDH 107	84.51	8.59	2.29	3.75					
☑	A5	GAPDH 107	84.25	8.70	2.13	4.08					
☑	A6	GAPDH 107	84.38	9.14	2.26	3.94					
☑	A7	GAPDH 110	84.46	8.76	2.29	3.82					
☑	A8	GAPDH 110	84.53	9.09	2.31	3.94					
☑	A9	GAPDH 110	84.53	8.94	2.13	4.20					
☑	A10	GAPDH 113	84.48	8.88	2.11	4.20					
☑	A11	GAPDH 113	84.51	7.29	2.09	3.49					
☑	A12	GAPDH 113	84.59	8.69	2.06	4.21					
☑	B1	FCER1A 102	78.97	8.70	2.50	3.48					
☑	B2	FCER1A 102	78.94	8.80	2.47	3.56					
☑	B3	FCER1A 102	78.94	9.61	2.50	3.85					
☑	B4	FCER1A 107	74.26	0.99	1.94	0.51	78.91	7.35	2.44	3.01	
☑	B5	FCER1A 107	78.74	7.22	2.46	2.93					
☑	B6	FCER1A 107	78.86	8.08	2.42	3.34					
☑	B7	FCER1A 110	74.26	0.94	1.91	0.49	78.86	7.16	2.40	2.98	
☑	B8	FCER1A 110	74.61	1.41	1.97	0.72	79.09	6.49	2.44	2.66	
☑	B9	FCER1A 110	78.96	9.49	2.49	3.82					
☑	B10	FCER1A 113	75.30	8.23	2.69	3.06	79.43	1.58	2.25	0.70	
☑	B11	FCER1A 113	75.58	7.50	3.00	2.50	79.63	0.72	2.28	0.26	
☑	B12	FCER1A 113	74.85	1.75	1.94	0.90	79.27	5.06	2.36	2.15	
☑	C1	GAPDH 115	84.40	9.47	2.17	4.36					
☑	C2	GAPDH 115	84.31	11.56	2.17	5.33					
☑	C3	GAPDH 115	84.30	11.10	2.16	5.13					
☑	C4	GAPDH 117	84.30	10.51	2.26	4.64					
☑	C5	GAPDH 117	84.23	10.31	2.25	4.59					

Results

Inc	Pos	Sample Name	Peak 1 Tm	Peak 1 Area	Peak 1 Width	Peak 1 Height	Peak 2 Tm	Peak 2 Area	Peak 2 Width	Peak 2 Height	Status
☑	C6	GAPDH 117	84.20	10.45	2.25	4.64					
☑	C7	GAPDH 119	84.30	10.29	2.18	4.72					
☑	C8	GAPDH 119	84.35	11.26	2.20	5.15					
☑	C9	GAPDH 119	84.46	11.74	2.15	5.47					
☑	C10	GAPDH 120	84.37	12.68	2.18	5.82					
☑	C11	GAPDH 120	84.35	10.94	2.21	4.95					
☑	C12	GAPDH 120	84.53	10.23	2.16	4.73					
☑	D1	FCER1A 115	74.88	4.55	2.51	1.82	79.05	4.50	2.40	1.87	
☑	D2	FCER1A 115	74.55	3.35	2.27	1.47	78.85	6.80	2.38	2.85	
☑	D3	FCER1A 115	75.46	8.63	3.09	2.80	79.30	1.22	2.57	0.47	
☑	D4	FCER1A 117	78.87	8.56	2.46	3.49					
☑	D5	FCER1A 117	73.93	0.83	1.81	0.45	78.67	9.61	2.46	3.91	
☑	D6	FCER1A 117	74.70	2.95	2.20	1.34	78.97	6.20	2.46	2.52	
☑	D7	FCER1A 119	74.62	1.66	2.04	0.81	79.07	6.55	2.42	2.71	
☑	D8	FCER1A 119	74.79	3.22	2.26	1.42	79.06	6.36	2.39	2.67	
☑	D9	FCER1A 119	74.55	1.56	2.12	0.74	79.06	8.54	2.43	3.51	
☑	D10	FCER1A 120	74.55	2.07	2.10	0.98	79.02	8.61	2.44	3.54	
☑	D11	FCER1A 120	74.64	2.09	2.14	0.98	79.01	7.05	2.46	2.87	
☑	D12	FCER1A 120	74.83	2.31	2.07	1.11	79.17	6.46	2.43	2.66	
☑	E1										
☑	E2										
☑	E3										
☑	E4										
☑	E5										
☑	E6										
☑	E7										
☑	E8										
☑	E9										
☑	E10										
☑	E11										
☑	E12										
☑	F1										
☑	F2										
☑	F3										
☑	F4										
☑	F5										

Results

Inc	Pos	Sample Name	Peak 1				Peak 2				Status
			Tm	Area	Width	Height	Tm	Area	Width	Height	
☑	F6										
☑	F7										
☑	F8										
☑	F9										
☑	F10										
☑	F11										
☑	F12										
☑	G1										
☑	G2										
☑	G3										
☑	G4										
☑	G5										
☑	G6										
☑	G7										
☑	G8										
☑	G9										
☑	G10										
☑	G11										
☑	G12										
☑	H1										
☑	H2										
☑	H3										
☑	H4										
☑	H5										
☑	H6										
☑	H7										
☑	H8										
☑	H9										
☑	H10										
☑	H11										
☑	H12										

Melting Curves

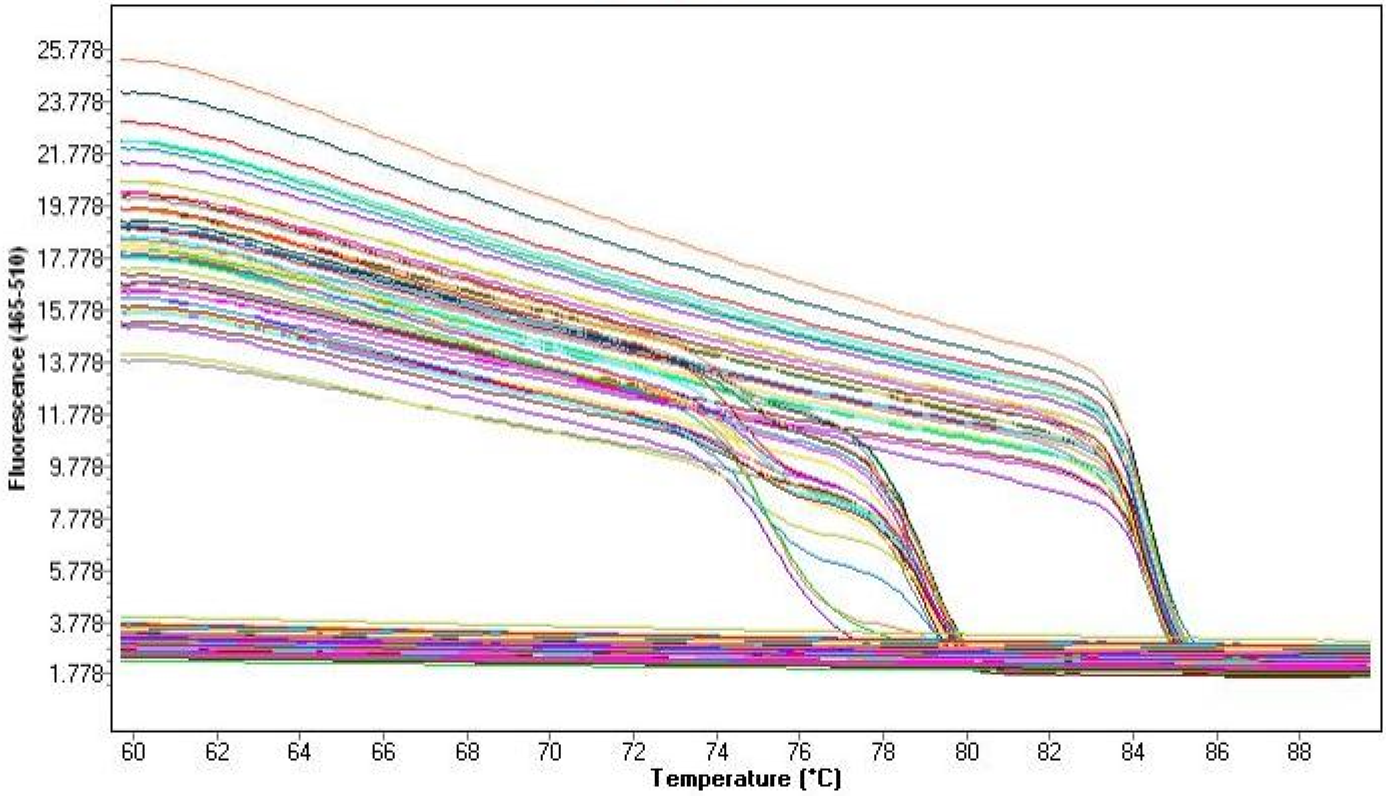

Melting Peaks

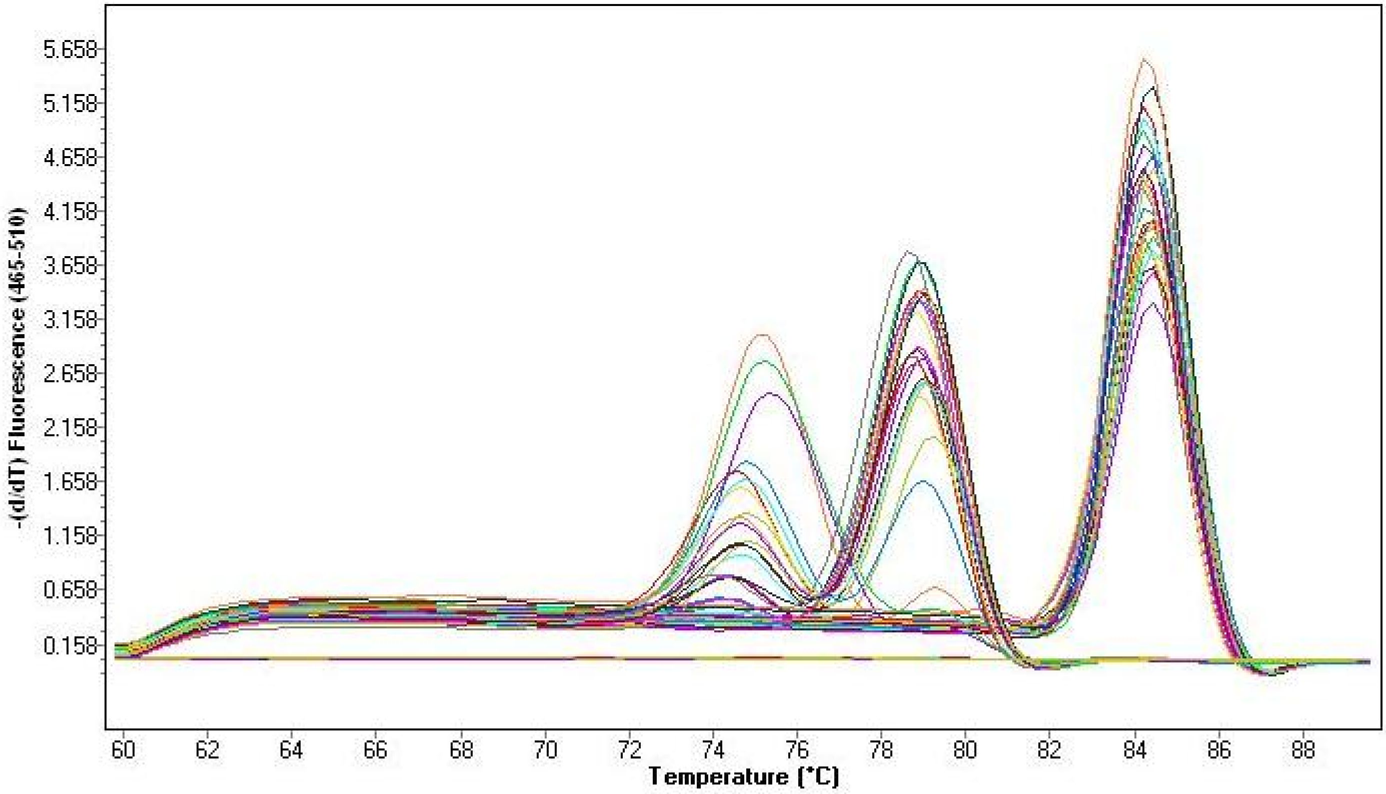

Analysis Notes	
