# Supplementary material for: Exploring differential gene expression and biomarker potential in systemic lupus erythematosus: a retrospective study
Source: PeerJ. 2025 Sep 8;13:e19891. doi: 10.7717/peerj.19891 (PMC12424612; doi:10.7717/peerj.19891)
Supplement: Supplemental Information 1 — The data include the Ct value for each sample, the Ct value for reference genes such as GAPDH, and the relative expression levels calculated using the ΔΔCt method. These data were used to validate the RNA sequencing results and further evaluate the potential of these genes as biomarkers for SLE. [file peerj-13-19891-s001.zip › qPCR/Method.docx]

Whole blood from HCs and SLE patients were collected and CD3^+^ T cells were sorted using anti-Human CD3 MicroBeads magnetic beads (Miltenyi, Germany). RNA was extracted using TRIzol reagent (Cat. #G3013 Servicebio, Wuhan, China), chloroform substitute (Cat. #G3014 Servicebio, Wuhan, China), etc. Swescript RT Enzyme Mix (Cat. # G331-1, Servicebio, Wuhan, China), Random Hexamer Primer (Cat. #G331-4, Servicebio, Wuhan, China) and 5xReaction Buffer (Cat. #G331-2, Servicebio, Wuhan, China) was used for cDNA synthesis. 2x Universal Blue SYBR Green qPCR Master Mix (Cat. #G3326-1, Servicebio, Wuhan, China) was used for qPCR. Relative gene expression was calculated using the 2^-ΔΔCt^ method, and the results were normalized using *GAPDH*.
