# Supplementary material for: Exploring differential gene expression and biomarker potential in systemic lupus erythematosus: a retrospective study
Source: PeerJ. 2025 Sep 8;13:e19891. doi: 10.7717/peerj.19891 (PMC12424612; doi:10.7717/peerj.19891)
Supplement: Supplemental Information 1 — The data include the Ct value for each sample, the Ct value for reference genes such as GAPDH, and the relative expression levels calculated using the ΔΔCt method. These data were used to validate the RNA sequencing results and further evaluate the potential of these genes as biomarkers for SLE. [file peerj-13-19891-s001.zip › qPCR/Primer.docx]

Primer Sequences.

| Primer Name | Sequence（5’-3’） |
| --- | --- |
| *GAPDH* -FOR | CTCATGACCACAGTCCATGC |
| *GAPDH* -REV | TTCAGCTCTGGGATGACCTT |
| *FCER1A*-FOR | GTTCTTCGCTCCAGATGGC |
| *FCER1A*-REV | TTGTGGAACCATTTGGTGGAA |
| *RGS1*-FOR | GGCGCAGTCTTTTGACAAGC |
| *RGS1*-REV | GCCTTCTCGTCTACCACATGC |
