# Supplementary figures and images for: Exploring differential gene expression and biomarker potential in systemic lupus erythematosus: a retrospective study
Source: PeerJ. 2025 Sep 8;13:e19891. doi: 10.7717/peerj.19891 (PMC12424612; doi:10.7717/peerj.19891)

## SLE1

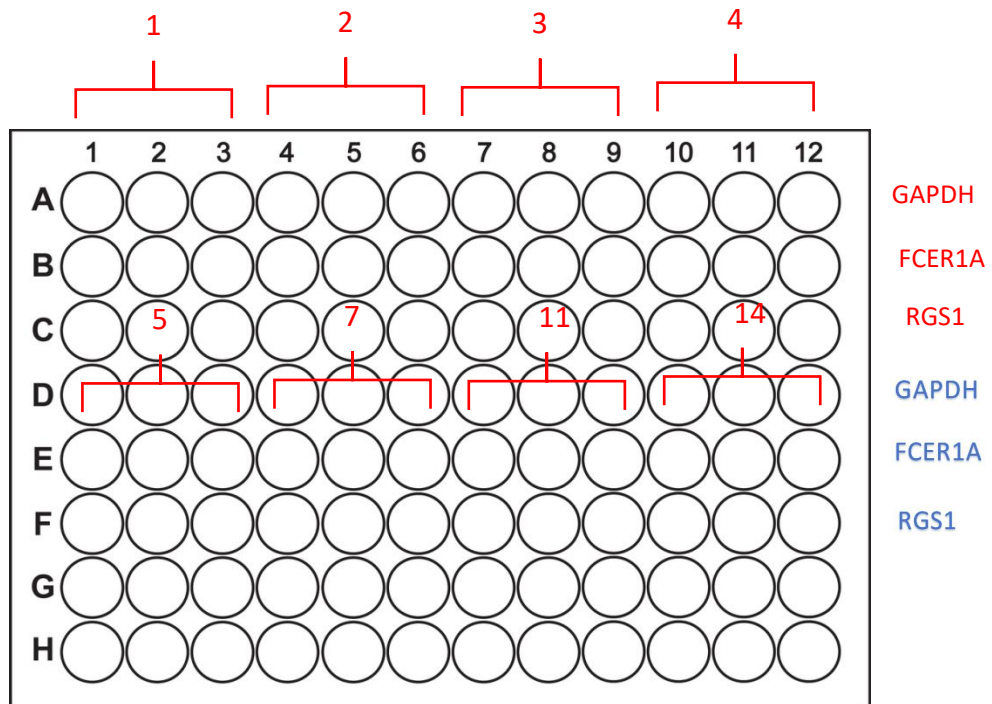

## SLE2

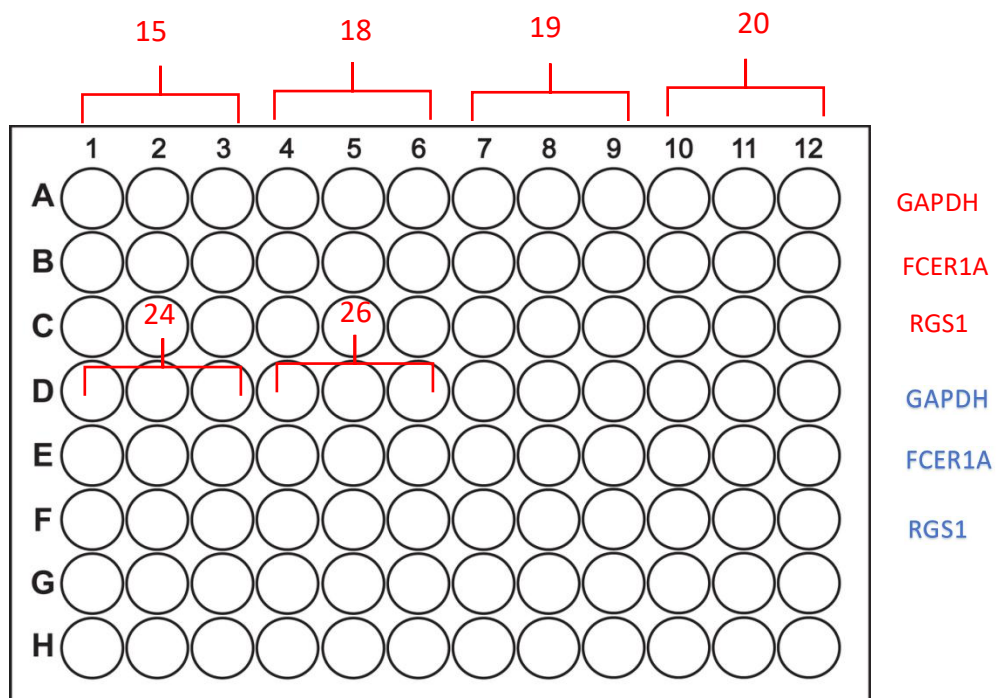

control3

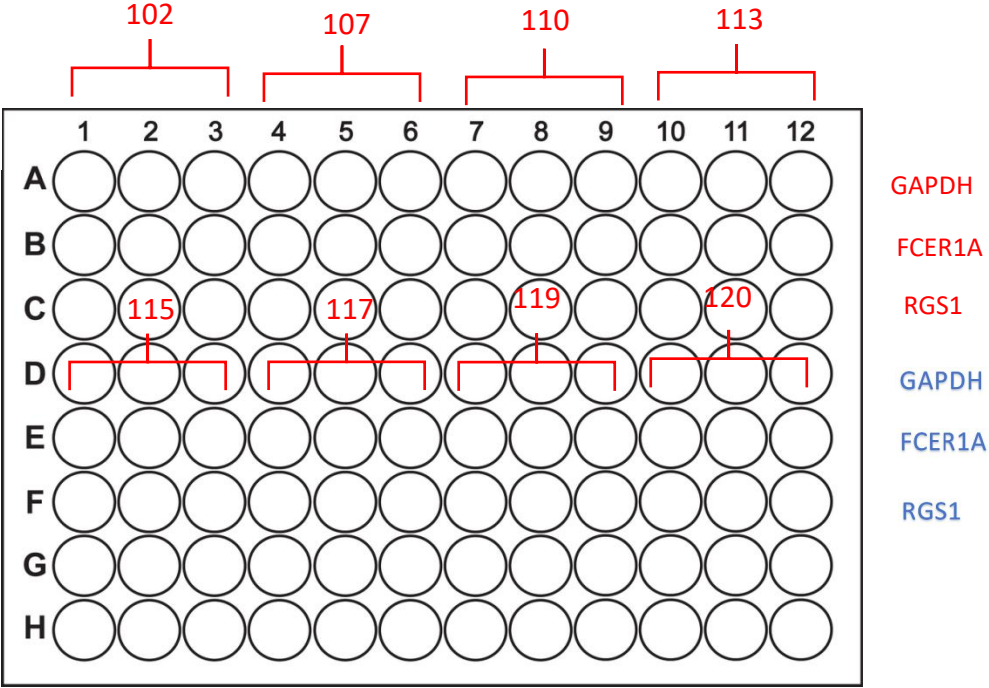

Supplement: Supplemental Information 1 — The data include the Ct value for each sample, the Ct value for reference genes such as GAPDH, and the relative expression levels calculated using the ΔΔCt method. These data were used to validate the RNA sequencing results and further evaluate the potential of these genes as biomarkers for SLE. [file peerj-13-19891-s001.zip › qPCR/20241212 qPCR/qPCR design.pdf]

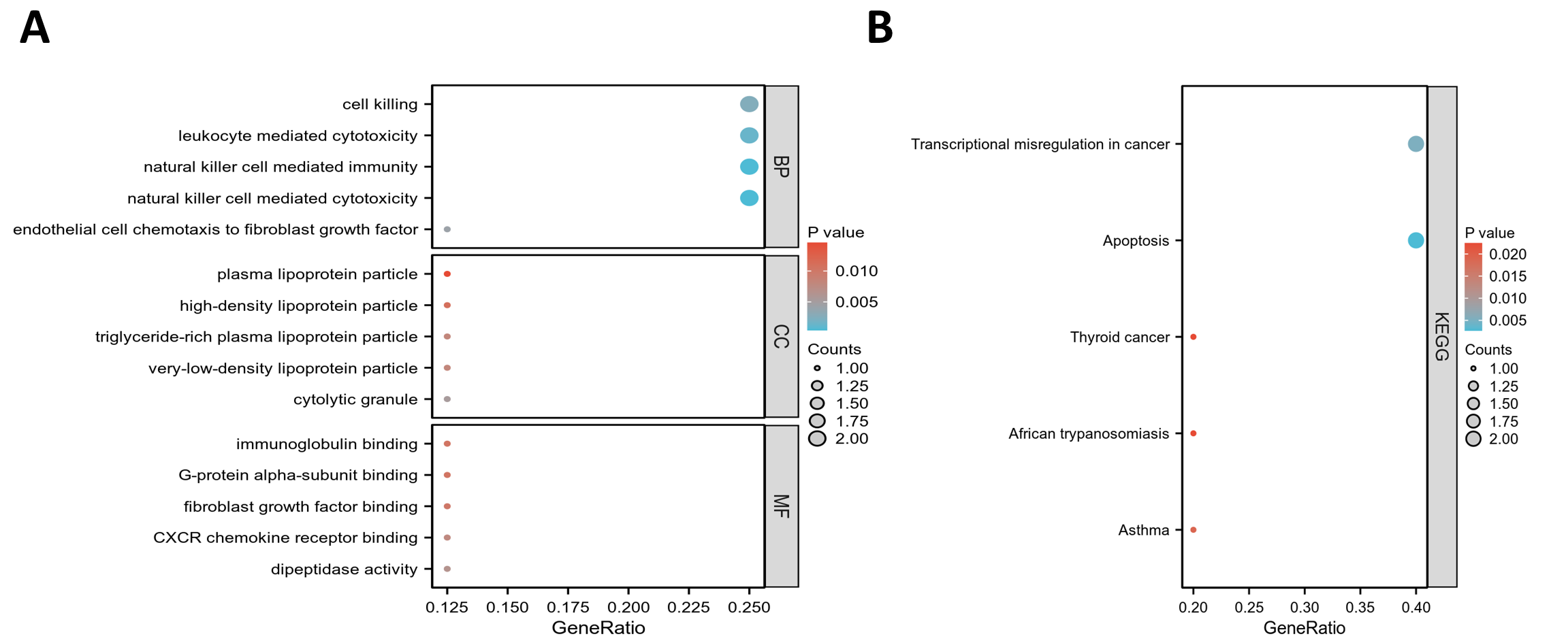

Supplement: Supplemental Information 7 — (A) The bubble plot shows the top 5 processes enriched in EP-DEGs in BPs, CCs, and MFs. (B) The bubble plot visualizes the top 5 significantly enriched KEGG pathways in DEGs (co-DEGs). [file peerj-13-19891-s007.png]

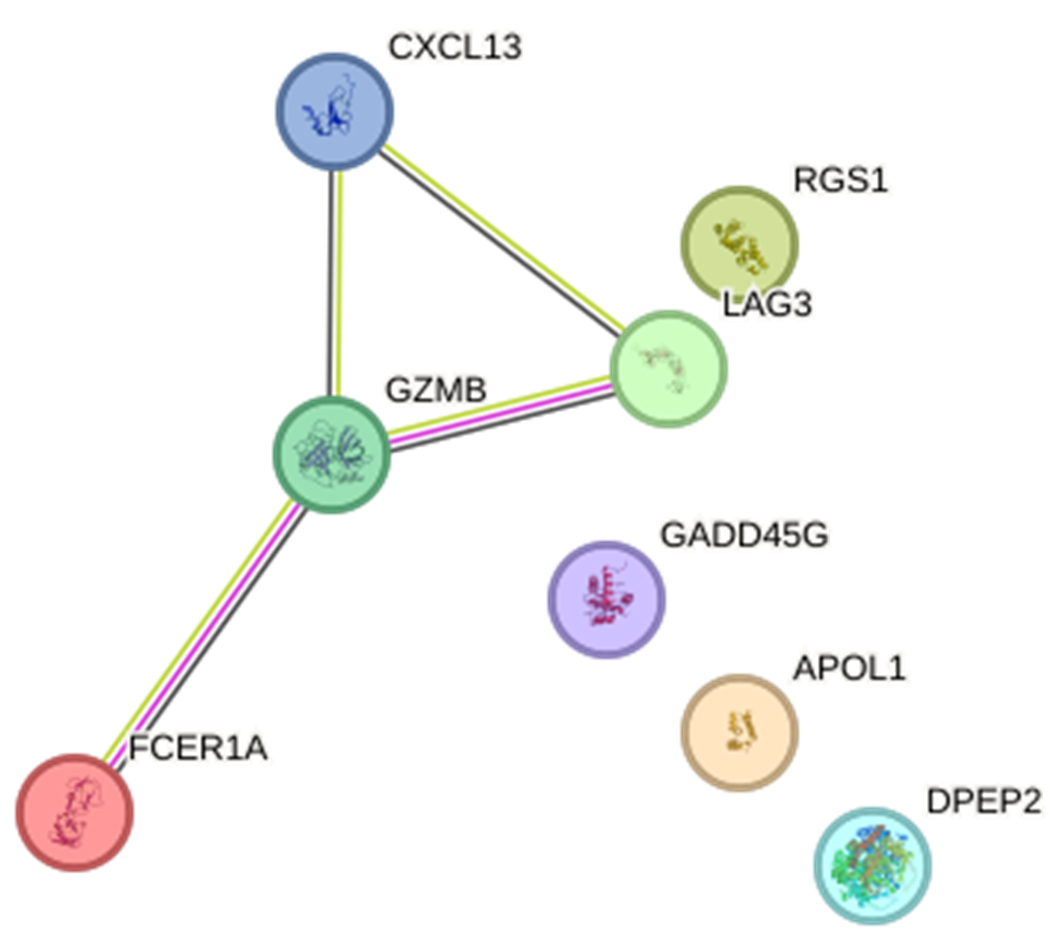

Supplement: Supplemental Information 8 — Fepicts a construction of a hub gene protein-protein interaction (PPI) network. [file peerj-13-19891-s008.png]

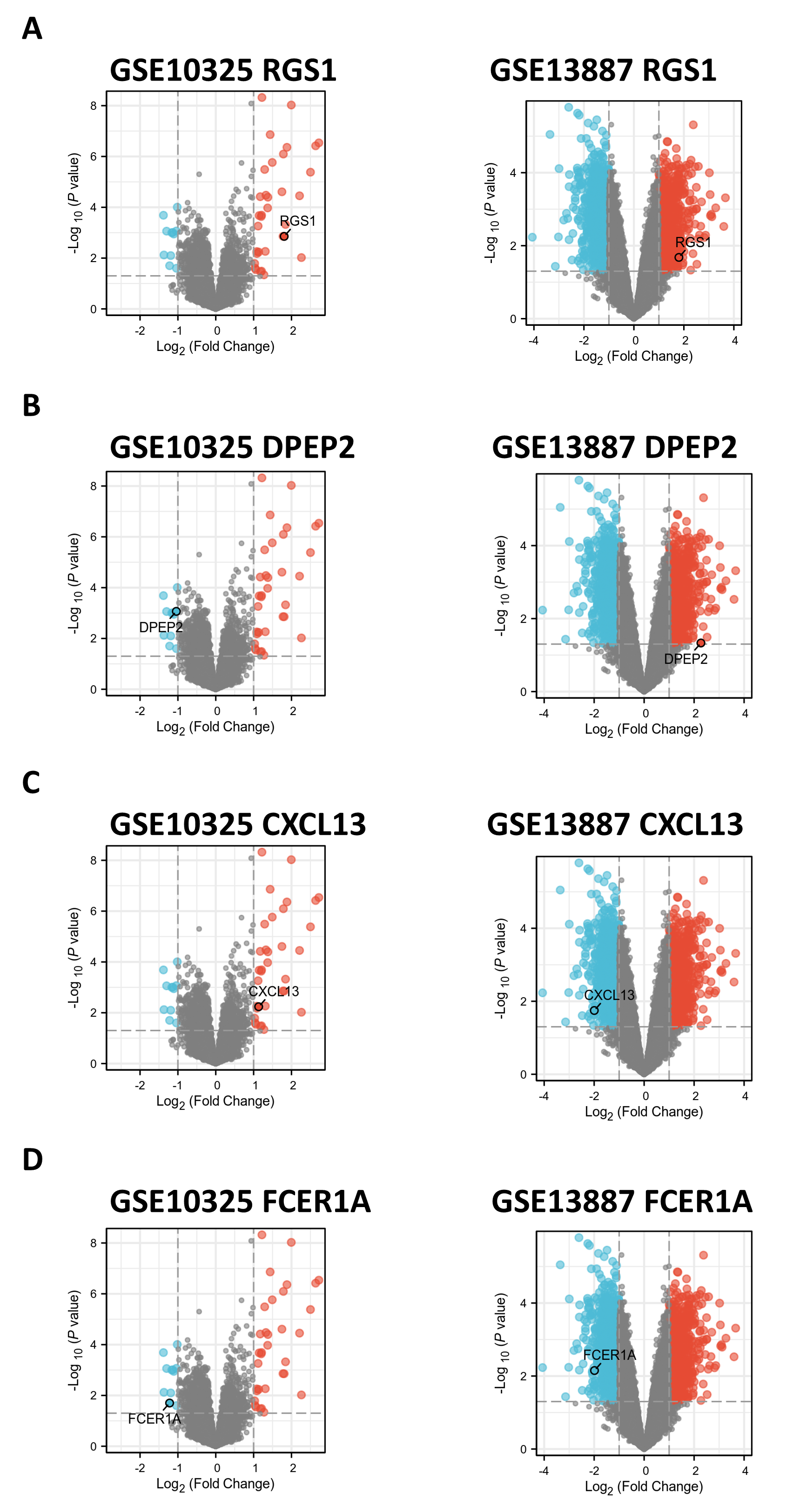

Supplement: Supplemental Information 9 — (A–D) Volcano plots of hub genes expressed in GSE10325 and GSE13887 datasets. [file peerj-13-19891-s009.png]

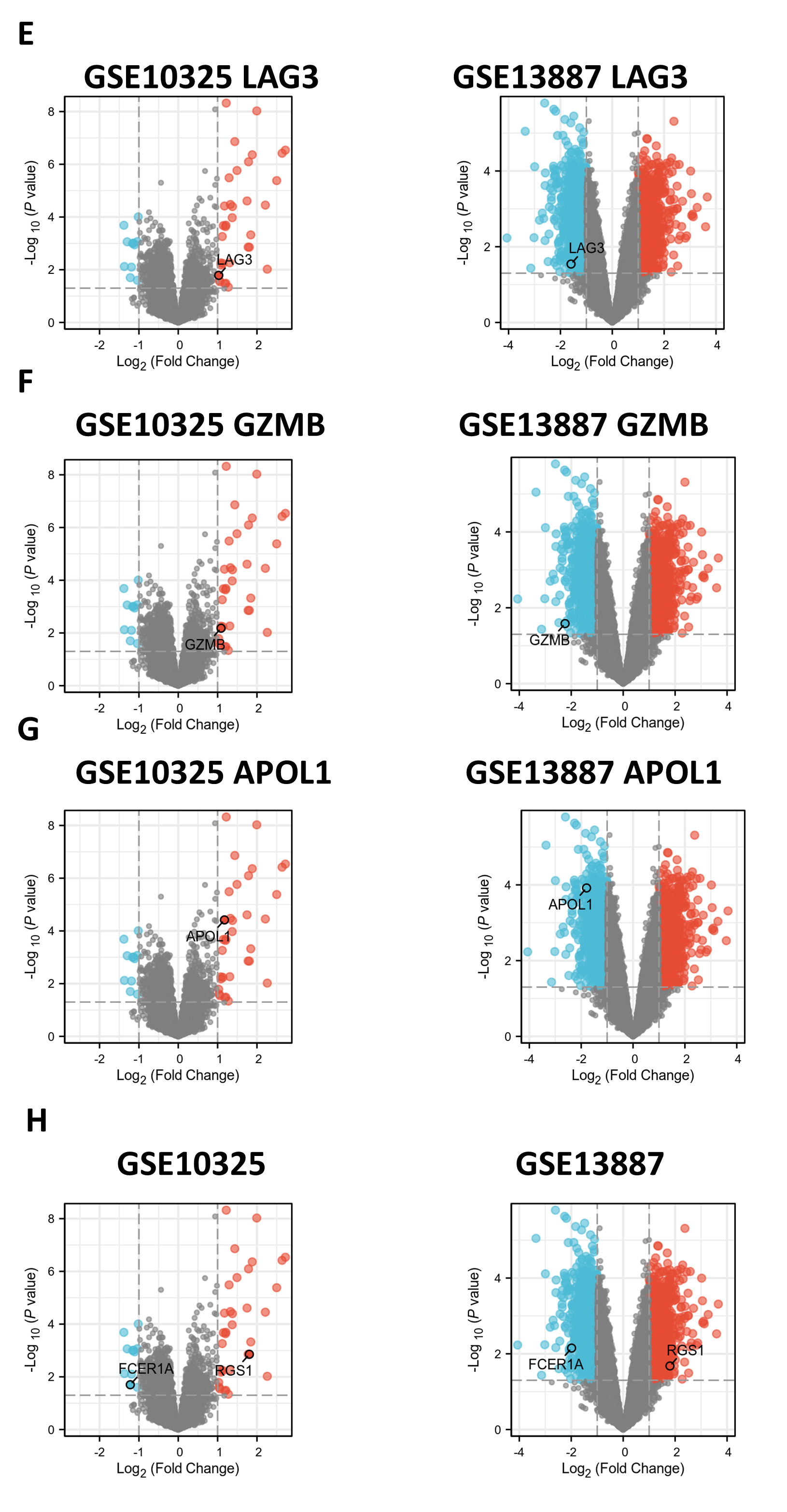

Supplement: Supplemental Information 10 — (E–G) Volcano plots of hub genes expressed in GSE10325 and GSE13887 datasets; (H) Volcano plot of FCER1A and RGS1, genes with consistent expression levels in GSE10325 and GSE13887. [file peerj-13-19891-s010.png]

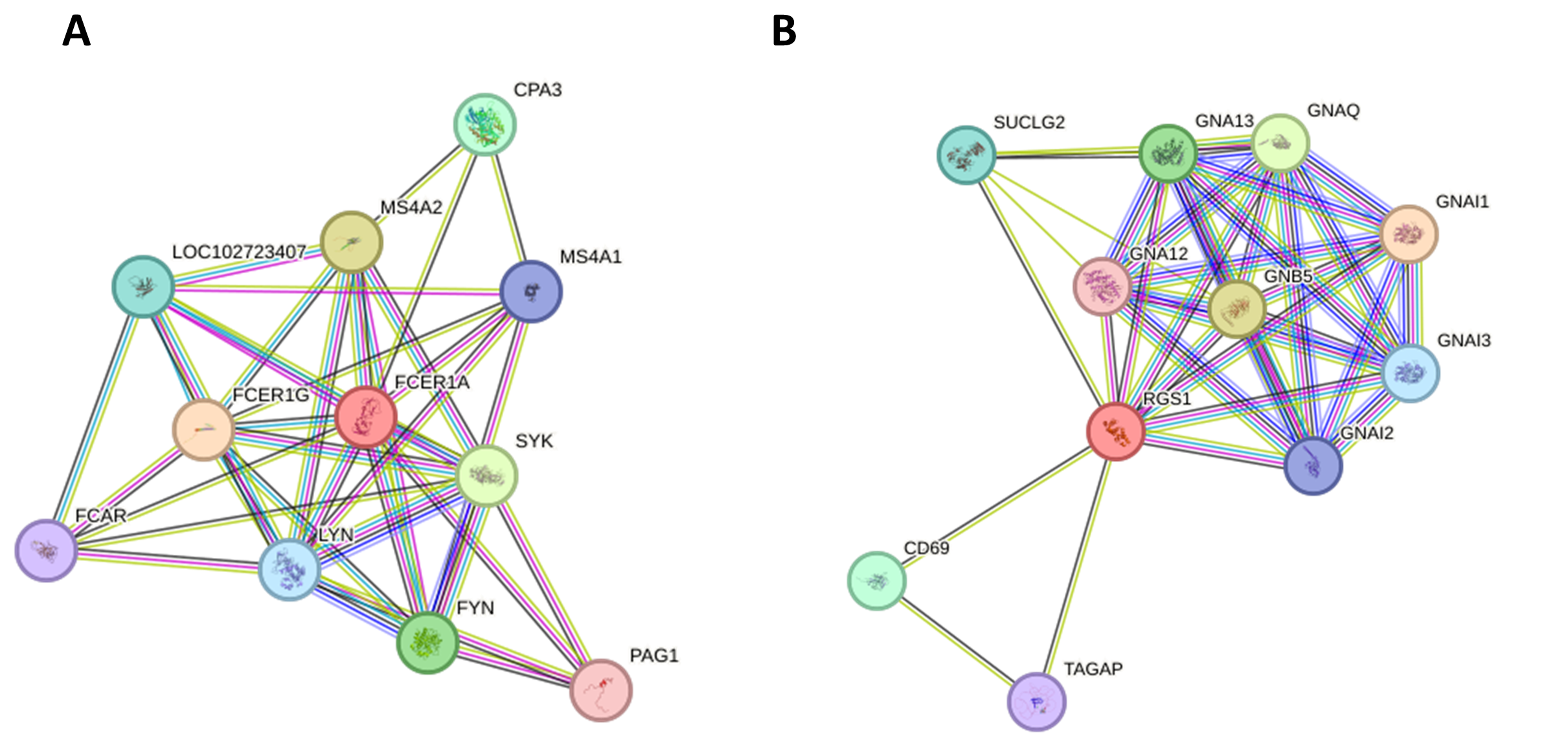

Supplement: Supplemental Information 11 — Displays a construction of the FCER1A and RGS1 protein-protein interaction (PPI) network. [file peerj-13-19891-s011.png]
