# Supplementary material for: Exploring differential gene expression and biomarker potential in systemic lupus erythematosus: a retrospective study
Source: PeerJ. 2025 Sep 8;13:e19891. doi: 10.7717/peerj.19891 (PMC12424612; doi:10.7717/peerj.19891)
Supplement: Supplemental Information 2 — These data are new and were recently supplemented with samples. The data include the Ct value for each sample, the Ct value for reference genes (e.g., GAPDH), and the relative expression levels calculated using the ΔΔCt method. These data were used to validate the RNA sequencing results and further evaluate the potential of these genes as biomarkers for SLE. [file peerj-13-19891-s002.zip › 20250528xj/20250528 HC2.PDF]

cobas® 4800 User Defined Workflow (UDF)

Roche

xj20250528 HC2

Experiment

Creation Date	5/28/2025 1:43:02 PM	Last Modified Date	6/2/2025 2:35:48 AM
Operator	System Admin	Owner	System Admin
Start Time	5/28/2025 1:44:03 PM	End Time	5/28/2025 2:43:34 PM
Run State	Completed	Software Version	LCS480 1.5.1.62 SP3 - UDF 2.1.0.26
Macro		Macro Owner	
Macro Status			
Templates	gongying	Plate ID	
Test ID		Lot ID	
Color Comp ID			
Run Notes			

Programs

Program Name	Program		
Cycles	1	Analysis Mode	None

Target (°C)	Acquisition Mode	Hold (hh:mm:ss)	Ramp Rate (°C/s)	Acquisitions (per °C)	Sec Target (°C)	Step size (°C)	Step Delay (cycles)
95	None	00:00:30	4.40		0	0	0

Program Name	Program		
Cycles	40	Analysis Mode	Quantification

Target (°C)	Acquisition Mode	Hold (hh:mm:ss)	Ramp Rate (°C/s)	Acquisitions (per °C)	Sec Target (°C)	Step size (°C)	Step Delay (cycles)
95	None	00:00:15	4.40		0	0	0
60	Single	00:00:30	2.20		0	0	0

Program Name	Program		
Cycles	1	Analysis Mode	Melting Curves

Target (°C)	Acquisition Mode	Hold (hh:mm:ss)	Ramp Rate (°C/s)	Acquisitions (per °C)	Sec Target (°C)	Step size (°C)	Step Delay (cycles)
95	None	00:01:00	4.40		0	0	0
60	None	00:00:30	2.20		0	0	0
90	Continuous		0.11	5	0	0	0

Program Name	Program		
Cycles	1	Analysis Mode	None

Target (°C)	Acquisition Mode	Hold (hh:mm:ss)	Ramp Rate (°C/s)	Acquisitions (per °C)	Sec Target (°C)	Step size (°C)	Step Delay (cycles)

Target (°C)	Acquisition Mode	Hold (hh:mm:ss)	Ramp Rate (°C/s)	Acquisitions (per °C)	Sec Target (°C)	Step size (°C)	Step Delay (cycles)
40	None	00:00:30	2.20		0	0	0

Samples

Sample Count	96
Subset	All Samples

Pos	Name	ID	Repl. Of	Sample Notes
A1	GAPDH 9			
A2	GAPDH 9			
A3	GAPDH 9			
A4	GAPDH 10			
A5	GAPDH 10			
A6	GAPDH 10			
A7	GAPDH 11			
A8	GAPDH 11			
A9	GAPDH 11			
A10	GAPDH 12			
A11	GAPDH 12			
A12	GAPDH 12			
B1	FCER1A 9			
B2	FCER1A 9			
B3	FCER1A 9			
B4	FCER1A 10			
B5	FCER1A 10			
B6	FCER1A 10			
B7	FCER1A 11			
B8	FCER1A 11			
B9	FCER1A 11			
B10	FCER1A 12			
B11	FCER1A 12			
B12	FCER1A 12			
C1	RGS1 9			
C2	RGS1 9			
C3	RGS1 9			
C4	RGS1 10			
C5	RGS1 10			
C6	RGS1 10			
C7	RGS1 1			
C8	RGS1 1			
C9	RGS1 1			
C10	RGS1 12			
C11	RGS1 12			
C12	RGS1 12			

Samples

Sample Count	96
Subset	All Samples

Pos	Name	ID	Repl. Of	Sample Notes
D1	GAPDH 13			
D2	GAPDH 13			
D3	GAPDH 13			
D4	GAPDH 14			
D5	GAPDH 14			
D6	GAPDH 14			
D7	GAPDH 15			
D8	GAPDH 15			
D9	GAPDH 15			
D10	GAPDH 16			
D11	GAPDH 16			
D12	GAPDH 16			
E1	FCER1A 13			
E2	FCER1A 13			
E3	FCER1A 13			
E4	FCER1A 14			
E5	FCER1A 14			
E6	FCER1A 14			
E7	FCER1A 15			
E8	FCER1A 15			
E9	FCER1A 15			
E10	FCER1A 16			
E11	FCER1A 16			
E12	FCER1A 16			
F1	RGS1 13			
F2	RGS1 13			
F3	RGS1 13			
F4	RGS1 14			
F5	RGS1 14			
F6	RGS1 14			
F7	RGS1 15			
F8	RGS1 15			
F9	RGS1 15			
F10	RGS1 16			
F11	RGS1 16			
F12	RGS1 16			
G1	blank			
G2	blank			
G3	blank			

Samples

Sample Count	96
Subset	All Samples

Pos	Name	ID	Repl. Of	Sample Notes
G4	blank			
G5	blank			
G6	blank			
G7	blank			
G8	blank			
G9	blank			
G10	blank			
G11	blank			
G12	blank			
H1	blank			
H2	blank			
H3	blank			
H4	blank			
H5	blank			
H6	blank			
H7	blank			
H8	blank			
H9	blank			
H10	blank			
H11	blank			
H12	blank			

Instrument

Name	Z480	Serial Number	56319

Revision History

Revision	Date	User	Reason

Abs Quant/2nd Derivative Max for All Samples (Abs Quant/2nd Derivative Max)

Settings

Channel	465-510		
Color Compensation	Off		
Program	Program	Units	
Mode	High Confidence		

Subset Name	All Samples

Results

> - late Cp call (last five cycles) has higher uncertainty

Inc	Pos	Name	Type	CP	Concentration	Standard	Status

Results

Inc	Pos	Name	Type	CP	Concentration	Standard	Status
☑	A1	GAPDH 9	Unknown	30.70			
☑	A2	GAPDH 9	Unknown	32.82			
☑	A3	GAPDH 9	Unknown	35.00			>
☑	A4	GAPDH 10	Unknown	32.35			
☑	A5	GAPDH 10	Unknown	29.30			
☑	A6	GAPDH 10	Unknown	35.00			>
☑	A7	GAPDH 11	Unknown	25.51			
☑	A8	GAPDH 11	Unknown	32.47			
☑	A9	GAPDH 11	Unknown	35.00			>
☑	A10	GAPDH 12	Unknown	35.00			>
☑	A11	GAPDH 12	Unknown	28.74			
☑	A12	GAPDH 12	Unknown	35.00			>
☑	B1	FCER1A 9	Unknown	30.00			
☑	B2	FCER1A 9	Unknown	29.91			
☑	B3	FCER1A 9	Unknown	31.11			
☑	B4	FCER1A 10	Unknown	30.02			
☑	B5	FCER1A 10	Unknown	35.00			>
☑	B6	FCER1A 10	Unknown	31.51			
☑	B7	FCER1A 11	Unknown	29.17			
☑	B8	FCER1A 11	Unknown	31.13			
☑	B9	FCER1A 11	Unknown	30.67			
☑	B10	FCER1A 12	Unknown	30.94			
☑	B11	FCER1A 12	Unknown	30.75			
☑	B12	FCER1A 12	Unknown	31.26			
☑	C1	RGS1 9	Unknown	35.00			>
☑	C2	RGS1 9	Unknown	33.00			
☑	C3	RGS1 9	Unknown	35.00			>
☑	C4	RGS1 10	Unknown	35.00			>
☑	C5	RGS1 10	Unknown	35.00			>
☑	C6	RGS1 10	Unknown	35.00			>
☑	C7	RGS1 1	Unknown	34.54			
☑	C8	RGS1 1	Unknown	35.00			>
☑	C9	RGS1 1	Unknown	35.00			>
☑	C10	RGS1 12	Unknown	32.67			
☑	C11	RGS1 12	Unknown	33.81			
☑	C12	RGS1 12	Unknown	33.44			
☑	D1	GAPDH 13	Unknown	32.05			

> - Late Cp call (last five cycles) has higher uncertainty

Results

Inc	Pos	Name	Type	CP	Concentration	Standard	Status
☑	D2	GAPDH 13	Unknown	31.62			
☑	D3	GAPDH 13	Unknown	35.00			>
☑	D4	GAPDH 14	Unknown	32.88			
☑	D5	GAPDH 14	Unknown	31.08			
☑	D6	GAPDH 14	Unknown	35.00			>
☑	D7	GAPDH 15	Unknown	30.57			
☑	D8	GAPDH 15	Unknown	35.00			>
☑	D9	GAPDH 15	Unknown	33.36			
☑	D10	GAPDH 16	Unknown	33.20			
☑	D11	GAPDH 16	Unknown	35.00			>
☑	D12	GAPDH 16	Unknown	33.35			
☑	E1	FCER1A 13	Unknown	29.81			
☑	E2	FCER1A 13	Unknown	29.49			
☑	E3	FCER1A 13	Unknown	31.04			
☑	E4	FCER1A 14	Unknown	29.49			
☑	E5	FCER1A 14	Unknown	28.67			
☑	E6	FCER1A 14	Unknown	28.74			
☑	E7	FCER1A 15	Unknown	29.11			
☑	E8	FCER1A 15	Unknown	32.33			
☑	E9	FCER1A 15	Unknown	30.88			
☑	E10	FCER1A 16	Unknown	29.97			
☑	E11	FCER1A 16	Unknown	30.96			
☑	E12	FCER1A 16	Unknown	30.64			
☑	F1	RGS1 13	Unknown	31.90			
☑	F2	RGS1 13	Unknown	33.90			
☑	F3	RGS1 13	Unknown	35.00			>
☑	F4	RGS1 14	Unknown	32.42			
☑	F5	RGS1 14	Unknown	32.70			
☑	F6	RGS1 14	Unknown	31.23			
☑	F7	RGS1 15	Unknown	35.00			>
☑	F8	RGS1 15	Unknown	35.00			>
☑	F9	RGS1 15	Unknown	33.13			
☑	F10	RGS1 16	Unknown	32.74			
☑	F11	RGS1 16	Unknown	33.21			
☑	F12	RGS1 16	Unknown	35.00			>
☑	G1	blank	Unknown				
☑	G2	blank	Unknown				

> - Late Cp call (last five cycles) has higher uncertainty

Results

Inc	Pos	Name	Type	CP	Concentration	Standard	Status
☑	G3	blank	Unknown				
☑	G4	blank	Unknown				
☑	G5	blank	Unknown				
☑	G6	blank	Unknown				
☑	G7	blank	Unknown				
☑	G8	blank	Unknown				
☑	G9	blank	Unknown				
☑	G10	blank	Unknown				
☑	G11	blank	Unknown				
☑	G12	blank	Unknown				
☑	H1	blank	Unknown				
☑	H2	blank	Unknown				
☑	H3	blank	Unknown				
☑	H4	blank	Unknown				
☑	H5	blank	Unknown				
☑	H6	blank	Unknown				
☑	H7	blank	Unknown				
☑	H8	blank	Unknown				
☑	H9	blank	Unknown				
☑	H10	blank	Unknown				
☑	H11	blank	Unknown				
☑	H12	blank	Unknown				

> - Late Cp call (last five cycles) has higher uncertainty

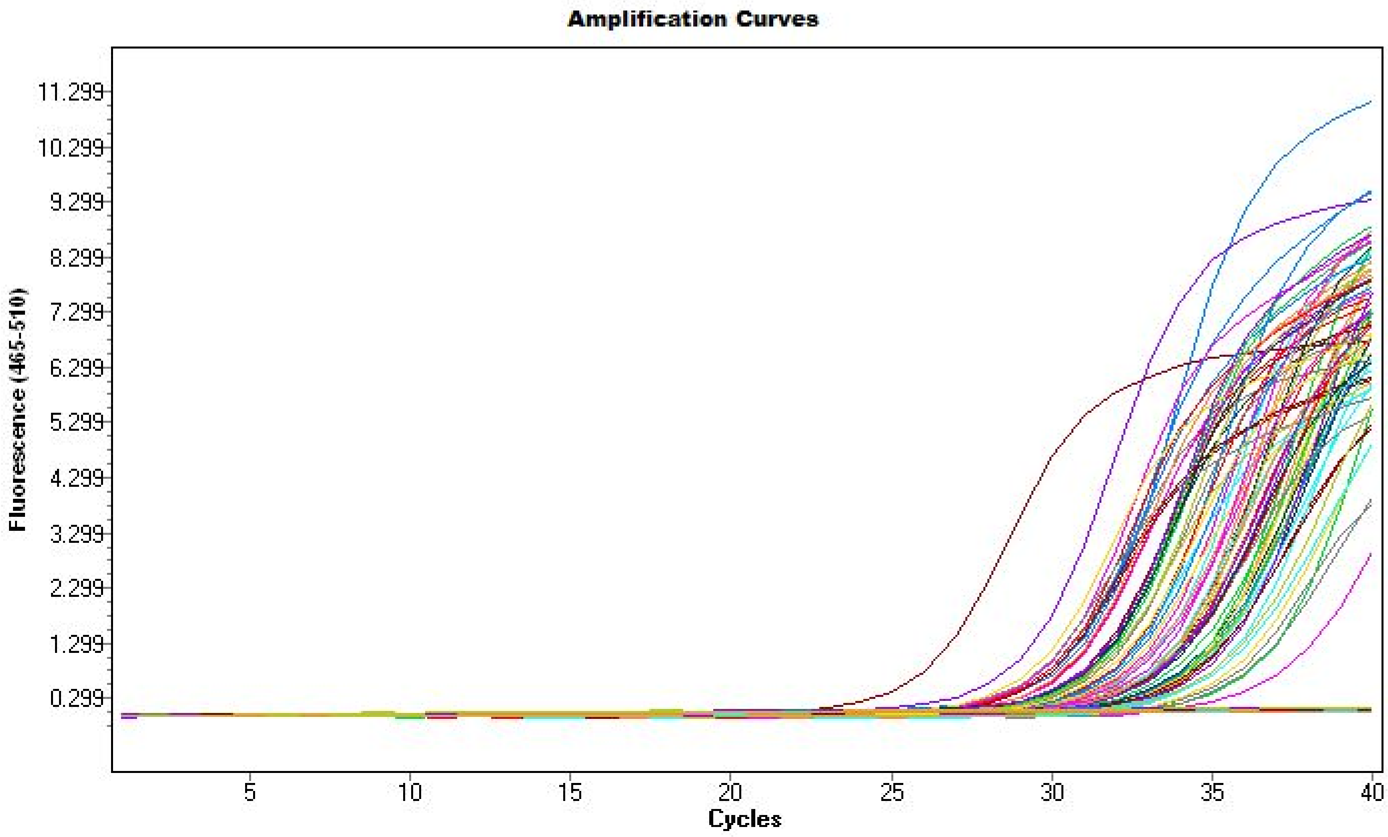

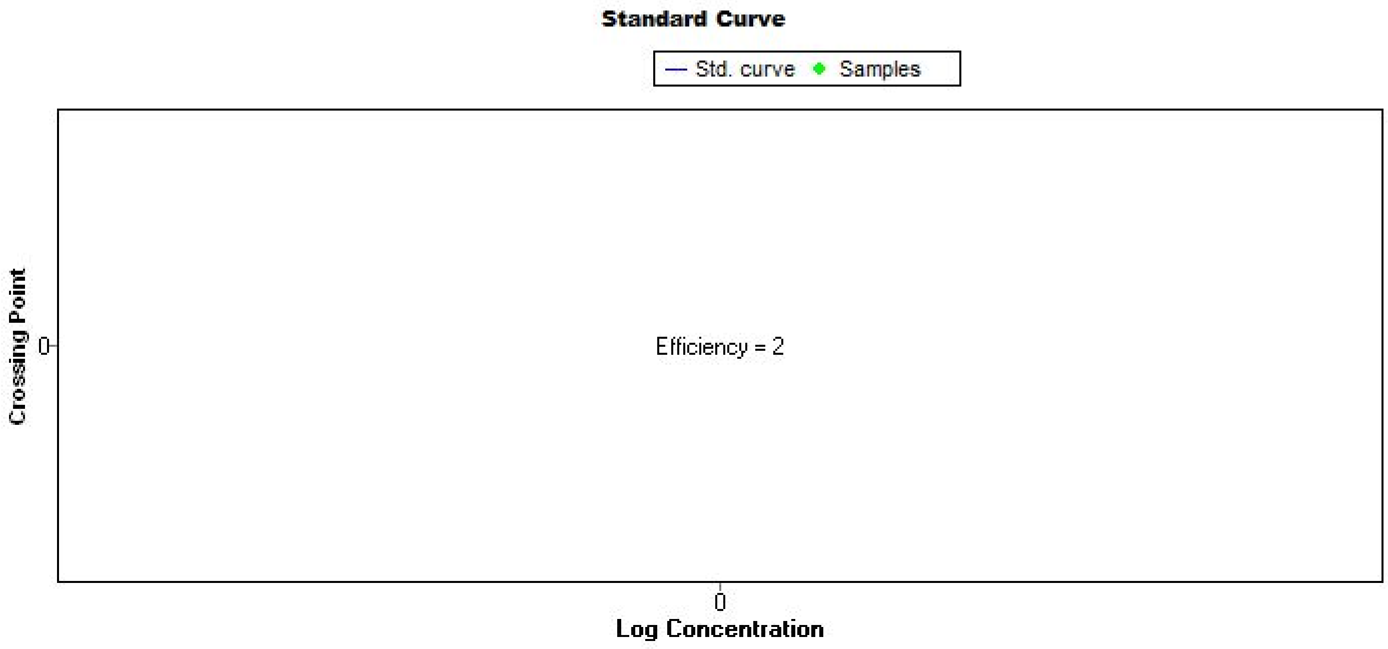

Analysis Notes	

Abs Quant/Fit Points for All Samples (Abs Quant/Fit Points)

Settings

Channel	465-510				
Color Compensation	Off				
Standard Curve	Set Efficiency (2.00)				
Program	Program			Units	
First Cycle	1	Last Cycle	40	Background	2-6
Noiseband Method	Auto	Noiseband	0.2576	STD Dev Multiplier	12.0000
# of Fit Points	2	Threshold Method	Auto	Threshold	0.2576

Subset Name	All Samples

Results

Inc	Pos	Name	Type	CP	Concentration	Standard	Status
☑	A1	GAPDH 9	Unknown	25.05			
☑	A2	GAPDH 9	Unknown	29.73			
☑	A3	GAPDH 9	Unknown	32.85			
☑	A4	GAPDH 10	Unknown	27.85			
☑	A5	GAPDH 10	Unknown	26.51			
☑	A6	GAPDH 10	Unknown	30.93			
☑	A7	GAPDH 11	Unknown	22.76			
☑	A8	GAPDH 11	Unknown	27.88			
☑	A9	GAPDH 11	Unknown	30.77			
☑	A10	GAPDH 12	Unknown	30.67			
☑	A11	GAPDH 12	Unknown	24.34			
☑	A12	GAPDH 12	Unknown	29.92			
☑	B1	FCER1A 9	Unknown	26.26			
☑	B2	FCER1A 9	Unknown	27.33			
☑	B3	FCER1A 9	Unknown	27.97			
☑	B4	FCER1A 10	Unknown	27.42			
☑	B5	FCER1A 10	Unknown	32.75			
☑	B6	FCER1A 10	Unknown	28.71			
☑	B7	FCER1A 11	Unknown	26.87			
☑	B8	FCER1A 11	Unknown	27.42			
☑	B9	FCER1A 11	Unknown	27.64			
☑	B10	FCER1A 12	Unknown	27.95			
☑	B11	FCER1A 12	Unknown	27.49			
☑	B12	FCER1A 12	Unknown	28.26			
☑	C1	RGS1 9	Unknown	30.38			
☑	C2	RGS1 9	Unknown	30.46			
☑	C3	RGS1 9	Unknown	31.22			

Results

Inc	Pos	Name	Type	CP	Concentration	Standard	Status
☑	C4	RGS1 10	Unknown	33.81			
☑	C5	RGS1 10	Unknown	33.17			
☑	C6	RGS1 10	Unknown	32.75			
☑	C7	RGS1 1	Unknown	31.75			
☑	C8	RGS1 1	Unknown	31.48			
☑	C9	RGS1 1	Unknown	30.52			
☑	C10	RGS1 12	Unknown	29.48			
☑	C11	RGS1 12	Unknown	29.81			
☑	C12	RGS1 12	Unknown	28.79			
☑	D1	GAPDH 13	Unknown	27.46			
☑	D2	GAPDH 13	Unknown	28.57			
☑	D3	GAPDH 13	Unknown	30.67			
☑	D4	GAPDH 14	Unknown	29.46			
☑	D5	GAPDH 14	Unknown	27.93			
☑	D6	GAPDH 14	Unknown	30.73			
☑	D7	GAPDH 15	Unknown	27.68			
☑	D8	GAPDH 15	Unknown	29.95			
☑	D9	GAPDH 15	Unknown	29.56			
☑	D10	GAPDH 16	Unknown	29.74			
☑	D11	GAPDH 16	Unknown	30.51			
☑	D12	GAPDH 16	Unknown	27.34			
☑	E1	FCER1A 13	Unknown	26.57			
☑	E2	FCER1A 13	Unknown	26.64			
☑	E3	FCER1A 13	Unknown	28.29			
☑	E4	FCER1A 14	Unknown	26.65			
☑	E5	FCER1A 14	Unknown	26.48			
☑	E6	FCER1A 14	Unknown	26.32			
☑	E7	FCER1A 15	Unknown	27.00			
☑	E8	FCER1A 15	Unknown	30.23			
☑	E9	FCER1A 15	Unknown	26.37			
☑	E10	FCER1A 16	Unknown	27.48			
☑	E11	FCER1A 16	Unknown	26.96			
☑	E12	FCER1A 16	Unknown	28.52			
☑	F1	RGS1 13	Unknown	28.89			
☑	F2	RGS1 13	Unknown	30.37			
☑	F3	RGS1 13	Unknown	30.52			
☑	F4	RGS1 14	Unknown	29.64			

Results

Inc	Pos	Name	Type	CP	Concentration	Standard	Status
☑	F5	RGS1 14	Unknown	29.91			
☑	F6	RGS1 14	Unknown	28.65			
☑	F7	RGS1 15	Unknown	31.25			
☑	F8	RGS1 15	Unknown	32.28			
☑	F9	RGS1 15	Unknown	30.52			
☑	F10	RGS1 16	Unknown	30.19			
☑	F11	RGS1 16	Unknown	30.43			
☑	F12	RGS1 16	Unknown	31.65			
☑	G1	blank	Unknown				
☑	G2	blank	Unknown				
☑	G3	blank	Unknown				
☑	G4	blank	Unknown				
☑	G5	blank	Unknown				
☑	G6	blank	Unknown				
☑	G7	blank	Unknown				
☑	G8	blank	Unknown				
☑	G9	blank	Unknown				
☑	G10	blank	Unknown				
☑	G11	blank	Unknown				
☑	G12	blank	Unknown				
☑	H1	blank	Unknown				
☑	H2	blank	Unknown				
☑	H3	blank	Unknown				
☑	H4	blank	Unknown				
☑	H5	blank	Unknown				
☑	H6	blank	Unknown				
☑	H7	blank	Unknown				
☑	H8	blank	Unknown				
☑	H9	blank	Unknown				
☑	H10	blank	Unknown				
☑	H11	blank	Unknown	32.17			
☑	H12	blank	Unknown				

Amplification Curves

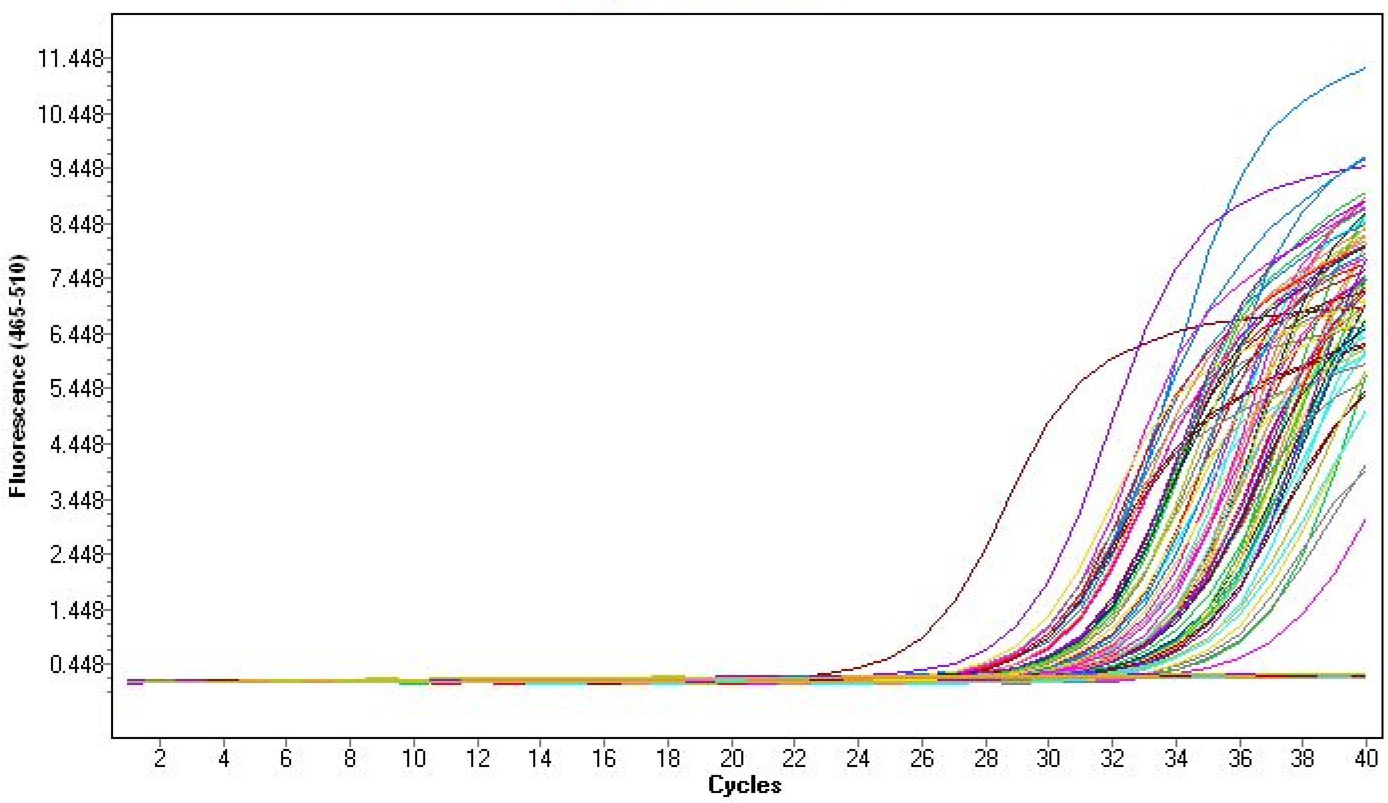

Amplification Curves

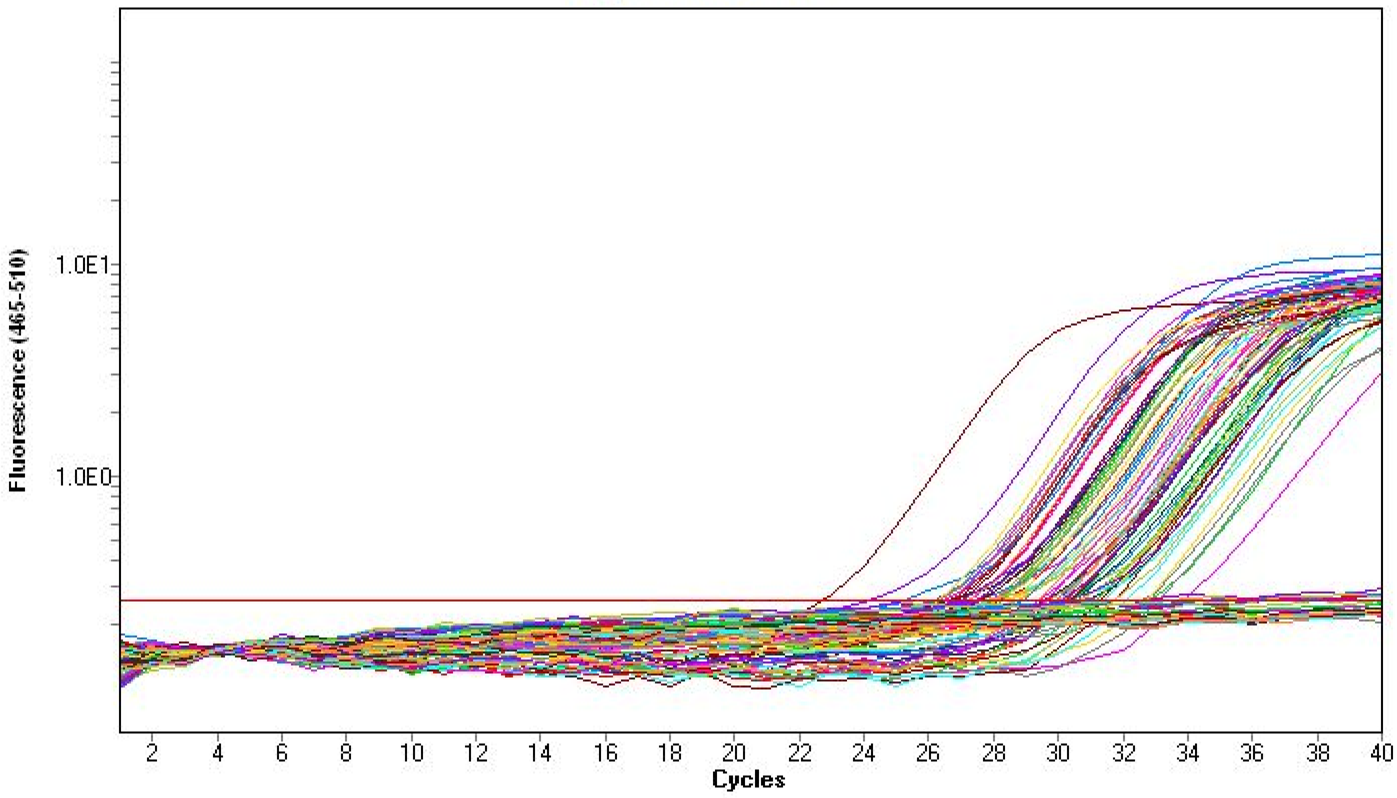

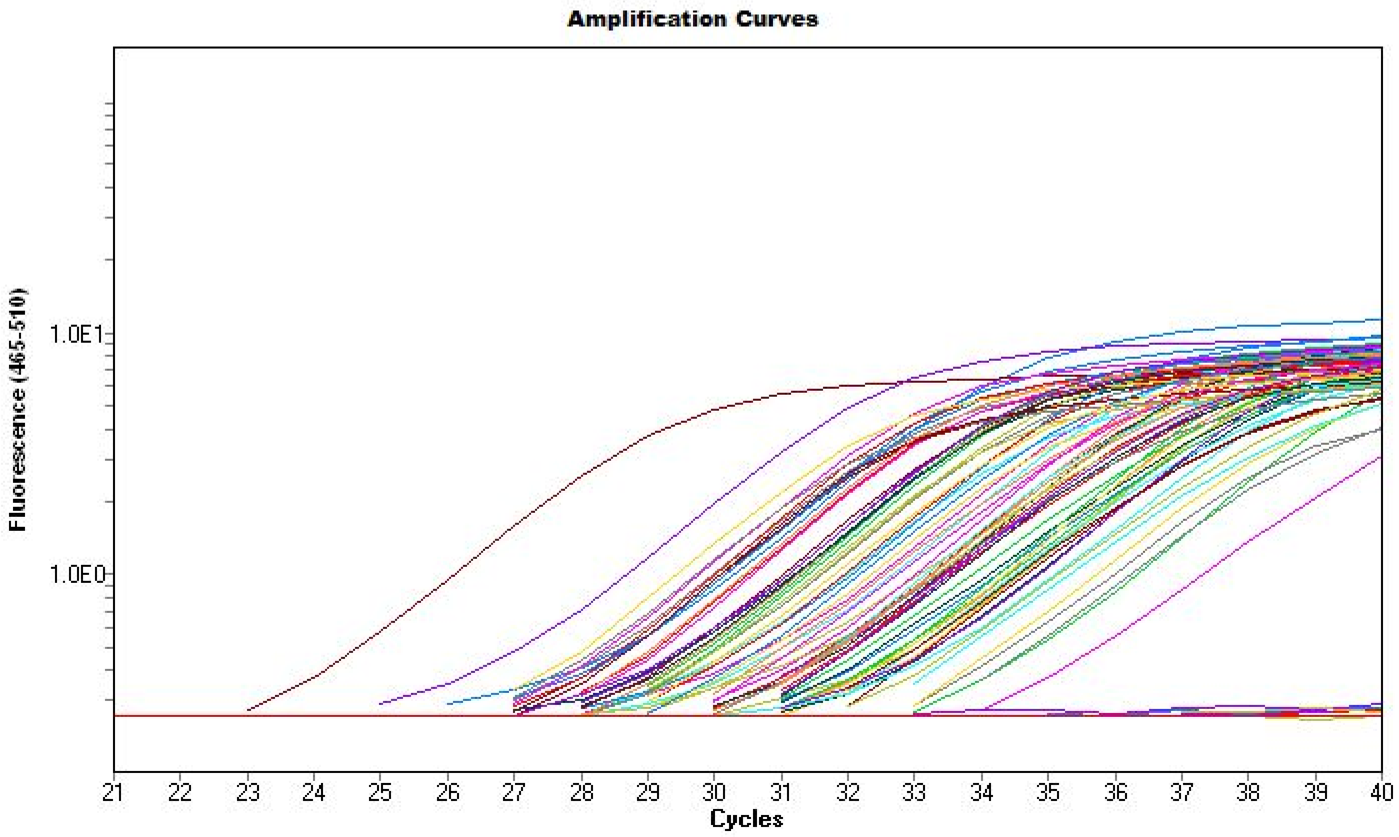

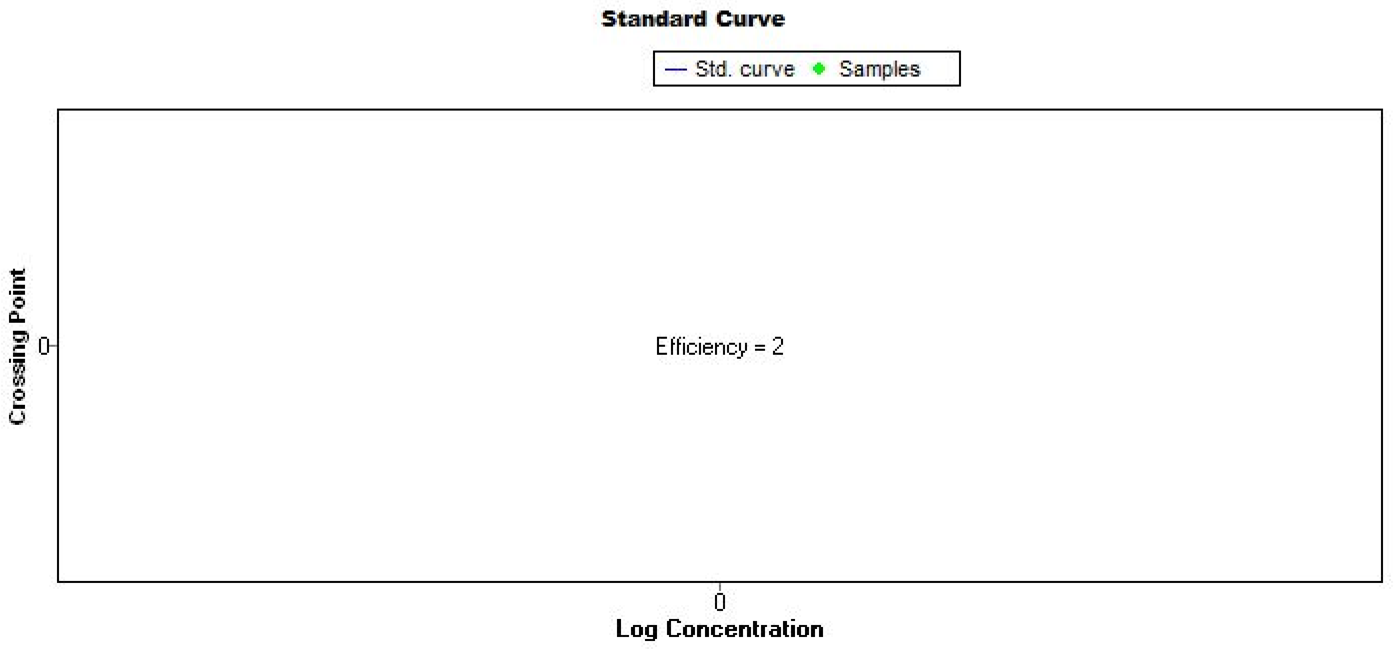

Analysis Notes	

Gene Scanning for All Samples (Gene Scanning)

Channel	465-510	Color Compensation	Off
Progam Name	Program		
Stds Settings	Auto-Group		
Sensitivity	0.30	Threshold (TmShift)	5.000
Pre-melt	69.99-70.99	Post-melt Temp	87.95-88.95

Subset Name	All Samples

Results

Inc	Pos.	Sample Name	Sample Type	Group	Status
☑	A1	GAPDH 9	Unknown	2	
☑	A2	GAPDH 9	Unknown	Unknown	
☑	A3	GAPDH 9	Unknown	Negative	
☑	A4	GAPDH 10	Unknown	2	
☑	A5	GAPDH 10	Unknown	5	
☑	A6	GAPDH 10	Unknown	2	
☑	A7	GAPDH 11	Unknown	5	
☑	A8	GAPDH 11	Unknown	Unknown	
☑	A9	GAPDH 11	Unknown	2	
☑	A10	GAPDH 12	Unknown	Unknown	
☑	A11	GAPDH 12	Unknown	5	
☑	A12	GAPDH 12	Unknown	2	
☑	B1	FCER1A 9	Unknown	4	
☑	B2	FCER1A 9	Unknown	4	
☑	B3	FCER1A 9	Unknown	3	
☑	B4	FCER1A 10	Unknown	5	
☑	B5	FCER1A 10	Unknown	Unknown	
☑	B6	FCER1A 10	Unknown	6	
☑	B7	FCER1A 11	Unknown	6	
☑	B8	FCER1A 11	Unknown	Unknown	
☑	B9	FCER1A 11	Unknown	4	
☑	B10	FCER1A 12	Unknown	3	
☑	B11	FCER1A 12	Unknown	3	
☑	B12	FCER1A 12	Unknown	3	
☑	C1	RGS1 9	Unknown	Unknown	
☑	C2	RGS1 9	Unknown	1	
☑	C3	RGS1 9	Unknown	1	
☑	C4	RGS1 10	Unknown	Negative	
☑	C5	RGS1 10	Unknown	1	
☑	C6	RGS1 10	Unknown	Unknown	
☑	C7	RGS1 1	Unknown	1	

Results

Inc	Pos.	Sample Name	Sample Type	Group	Status
☑	C8	RGS1 1	Unknown	1	
☑	C9	RGS1 1	Unknown	1	
☑	C10	RGS1 12	Unknown	1	
☑	C11	RGS1 12	Unknown	1	
☑	C12	RGS1 12	Unknown	Unknown	
☑	D1	GAPDH 13	Unknown	2	
☑	D2	GAPDH 13	Unknown	2	
☑	D3	GAPDH 13	Unknown	2	
☑	D4	GAPDH 14	Unknown	2	
☑	D5	GAPDH 14	Unknown	2	
☑	D6	GAPDH 14	Unknown	2	
☑	D7	GAPDH 15	Unknown	Unknown	
☑	D8	GAPDH 15	Unknown	2	
☑	D9	GAPDH 15	Unknown	2	
☑	D10	GAPDH 16	Unknown	2	
☑	D11	GAPDH 16	Unknown	2	
☑	D12	GAPDH 16	Unknown	2	
☑	E1	FCER1A 13	Unknown	6	
☑	E2	FCER1A 13	Unknown	4	
☑	E3	FCER1A 13	Unknown	Unknown	
☑	E4	FCER1A 14	Unknown	3	
☑	E5	FCER1A 14	Unknown	4	
☑	E6	FCER1A 14	Unknown	3	
☑	E7	FCER1A 15	Unknown	Unknown	
☑	E8	FCER1A 15	Unknown	3	
☑	E9	FCER1A 15	Unknown	3	
☑	E10	FCER1A 16	Unknown	Unknown	
☑	E11	FCER1A 16	Unknown	3	
☑	E12	FCER1A 16	Unknown	3	
☑	F1	RGS1 13	Unknown	1	
☑	F2	RGS1 13	Unknown	1	
☑	F3	RGS1 13	Unknown	1	
☑	F4	RGS1 14	Unknown	1	
☑	F5	RGS1 14	Unknown	1	
☑	F6	RGS1 14	Unknown	1	
☑	F7	RGS1 15	Unknown	1	
☑	F8	RGS1 15	Unknown	1	
☑	F9	RGS1 15	Unknown	1	

Results

Inc	Pos.	Sample Name	Sample Type	Group	Status
☑	F10	RGS1 16	Unknown	Unknown	
☑	F11	RGS1 16	Unknown	Unknown	
☑	F12	RGS1 16	Unknown	Unknown	
☑	G1	blank	Unknown	Negative	
☑	G2	blank	Unknown	Negative	
☑	G3	blank	Unknown	Negative	
☑	G4	blank	Unknown	Negative	
☑	G5	blank	Unknown	Negative	
☑	G6	blank	Unknown	Negative	
☑	G7	blank	Unknown	Negative	
☑	G8	blank	Unknown	Negative	
☑	G9	blank	Unknown	Negative	
☑	G10	blank	Unknown	Negative	
☑	G11	blank	Unknown	Negative	
☑	G12	blank	Unknown	Negative	
☑	H1	blank	Unknown	Negative	
☑	H2	blank	Unknown	Negative	
☑	H3	blank	Unknown	Negative	
☑	H4	blank	Unknown	Negative	
☑	H5	blank	Unknown	Negative	
☑	H6	blank	Unknown	Negative	
☑	H7	blank	Unknown	Negative	
☑	H8	blank	Unknown	Negative	
☑	H9	blank	Unknown	Negative	
☑	H10	blank	Unknown	Negative	
☑	H11	blank	Unknown	Negative	
☑	H12	blank	Unknown	Negative	

**Group**	1

Inc	Pos	Sample Name	Sample Type
☑	C2	RGS1 9	Unknown
☑	C3	RGS1 9	Unknown
☑	C5	RGS1 10	Unknown
☑	C7	RGS1 1	Unknown
☑	C8	RGS1 1	Unknown
☑	C9	RGS1 1	Unknown
☑	C10	RGS1 12	Unknown
☑	C11	RGS1 12	Unknown
☑	F1	RGS1 13	Unknown

Group	1

Inc	Pos	Sample Name	Sample Type
☑	F2	RGS1 13	Unknown
☑	F3	RGS1 13	Unknown
☑	F4	RGS1 14	Unknown
☑	F5	RGS1 14	Unknown
☑	F6	RGS1 14	Unknown
☑	F7	RGS1 15	Unknown
☑	F8	RGS1 15	Unknown
☑	F9	RGS1 15	Unknown

Group	2

Inc	Pos	Sample Name	Sample Type
☑	A1	GAPDH 9	Unknown
☑	A4	GAPDH 10	Unknown
☑	A6	GAPDH 10	Unknown
☑	A9	GAPDH 11	Unknown
☑	A12	GAPDH 12	Unknown
☑	D1	GAPDH 13	Unknown
☑	D2	GAPDH 13	Unknown
☑	D3	GAPDH 13	Unknown
☑	D4	GAPDH 14	Unknown
☑	D5	GAPDH 14	Unknown
☑	D6	GAPDH 14	Unknown
☑	D8	GAPDH 15	Unknown
☑	D9	GAPDH 15	Unknown
☑	D10	GAPDH 16	Unknown
☑	D11	GAPDH 16	Unknown
☑	D12	GAPDH 16	Unknown

Group	3

Inc	Pos	Sample Name	Sample Type
☑	B3	FCER1A 9	Unknown
☑	B10	FCER1A 12	Unknown
☑	B11	FCER1A 12	Unknown
☑	B12	FCER1A 12	Unknown
☑	E4	FCER1A 14	Unknown
☑	E6	FCER1A 14	Unknown
☑	E8	FCER1A 15	Unknown
☑	E9	FCER1A 15	Unknown
☑	E11	FCER1A 16	Unknown
☑	E12	FCER1A 16	Unknown

Group	4

Inc	Pos	Sample Name	Sample Type
☑	B1	FCER1A 9	Unknown
☑	B2	FCER1A 9	Unknown
☑	B9	FCER1A 11	Unknown
☑	E2	FCER1A 13	Unknown
☑	E5	FCER1A 14	Unknown

Group	5

Inc	Pos	Sample Name	Sample Type
☑	A5	GAPDH 10	Unknown
☑	A7	GAPDH 11	Unknown
☑	A11	GAPDH 12	Unknown
☑	B4	FCER1A 10	Unknown

Group	6

Inc	Pos	Sample Name	Sample Type
☑	B6	FCER1A 10	Unknown
☑	B7	FCER1A 11	Unknown
☑	E1	FCER1A 13	Unknown

Group	Negative

Inc	Pos	Sample Name	Sample Type
☑	A3	GAPDH 9	Unknown
☑	C4	RGS1 10	Unknown
☑	G1	blank	Unknown
☑	G2	blank	Unknown
☑	G3	blank	Unknown
☑	G4	blank	Unknown
☑	G5	blank	Unknown
☑	G6	blank	Unknown
☑	G7	blank	Unknown
☑	G8	blank	Unknown
☑	G9	blank	Unknown
☑	G10	blank	Unknown
☑	G11	blank	Unknown
☑	G12	blank	Unknown
☑	H1	blank	Unknown
☑	H2	blank	Unknown
☑	H3	blank	Unknown
☑	H4	blank	Unknown
☑	H5	blank	Unknown

**Group**	Negative

Inc	Pos	Sample Name	Sample Type
☑	H6	blank	Unknown
☑	H7	blank	Unknown
☑	H8	blank	Unknown
☑	H9	blank	Unknown
☑	H10	blank	Unknown
☑	H11	blank	Unknown
☑	H12	blank	Unknown

**Group**	Unknown

Inc	Pos	Sample Name	Sample Type
☑	A2	GAPDH 9	Unknown
☑	A8	GAPDH 11	Unknown
☑	A10	GAPDH 12	Unknown
☑	B5	FCER1A 10	Unknown
☑	B8	FCER1A 11	Unknown
☑	C1	RGS1 9	Unknown
☑	C6	RGS1 10	Unknown
☑	C12	RGS1 12	Unknown
☑	D7	GAPDH 15	Unknown
☑	E3	FCER1A 13	Unknown
☑	E7	FCER1A 15	Unknown
☑	E10	FCER1A 16	Unknown
☑	F10	RGS1 16	Unknown
☑	F11	RGS1 16	Unknown
☑	F12	RGS1 16	Unknown

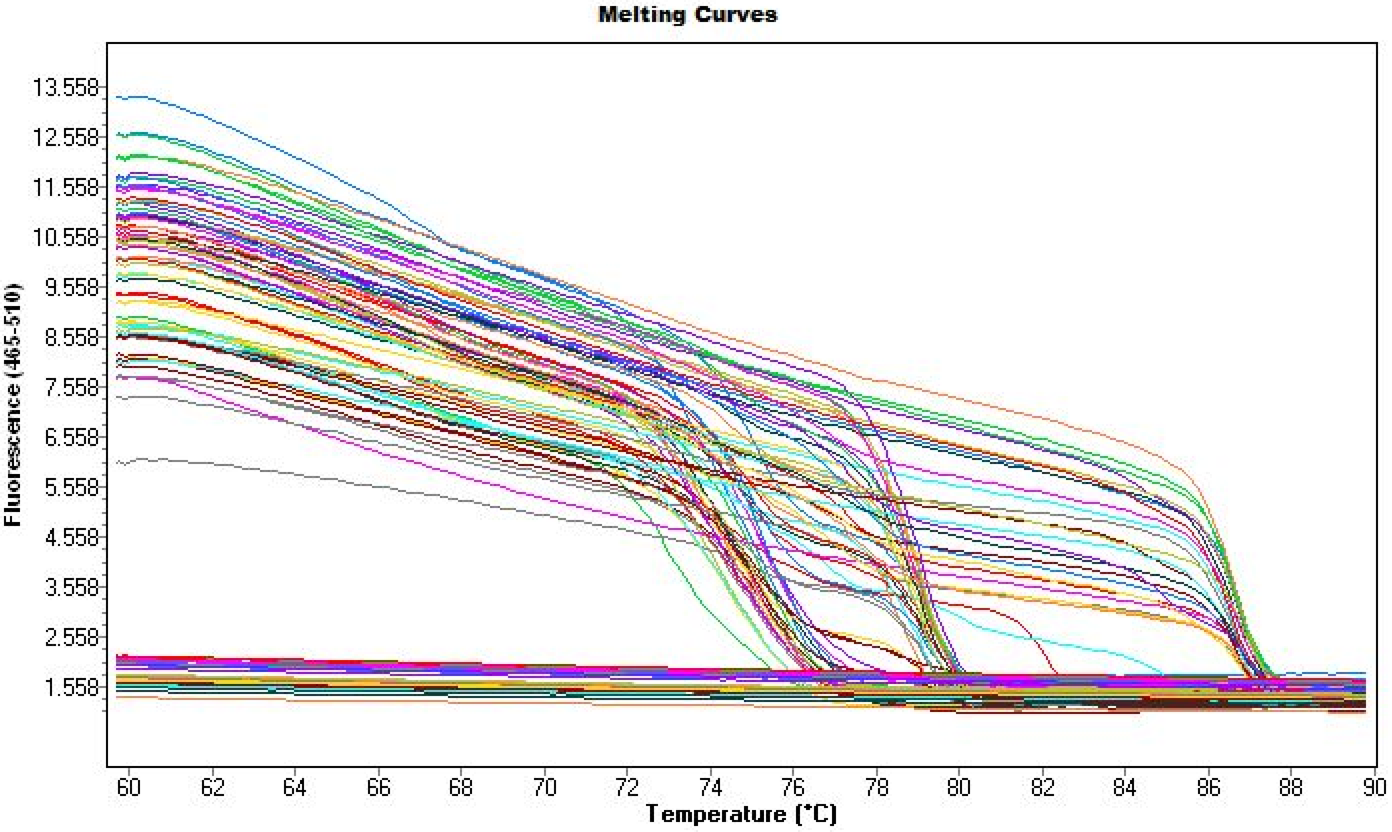
Melting Curves
Fluorescence (465-510)
13.558
12.558
11.558
10.558
9.558
8.558
7.558
6.558
5.558
4.558
3.558
2.558
1.558
60
62
64
66
68
70
72
74
76
78
80
82
84
86
88
90
Temperature (°C)

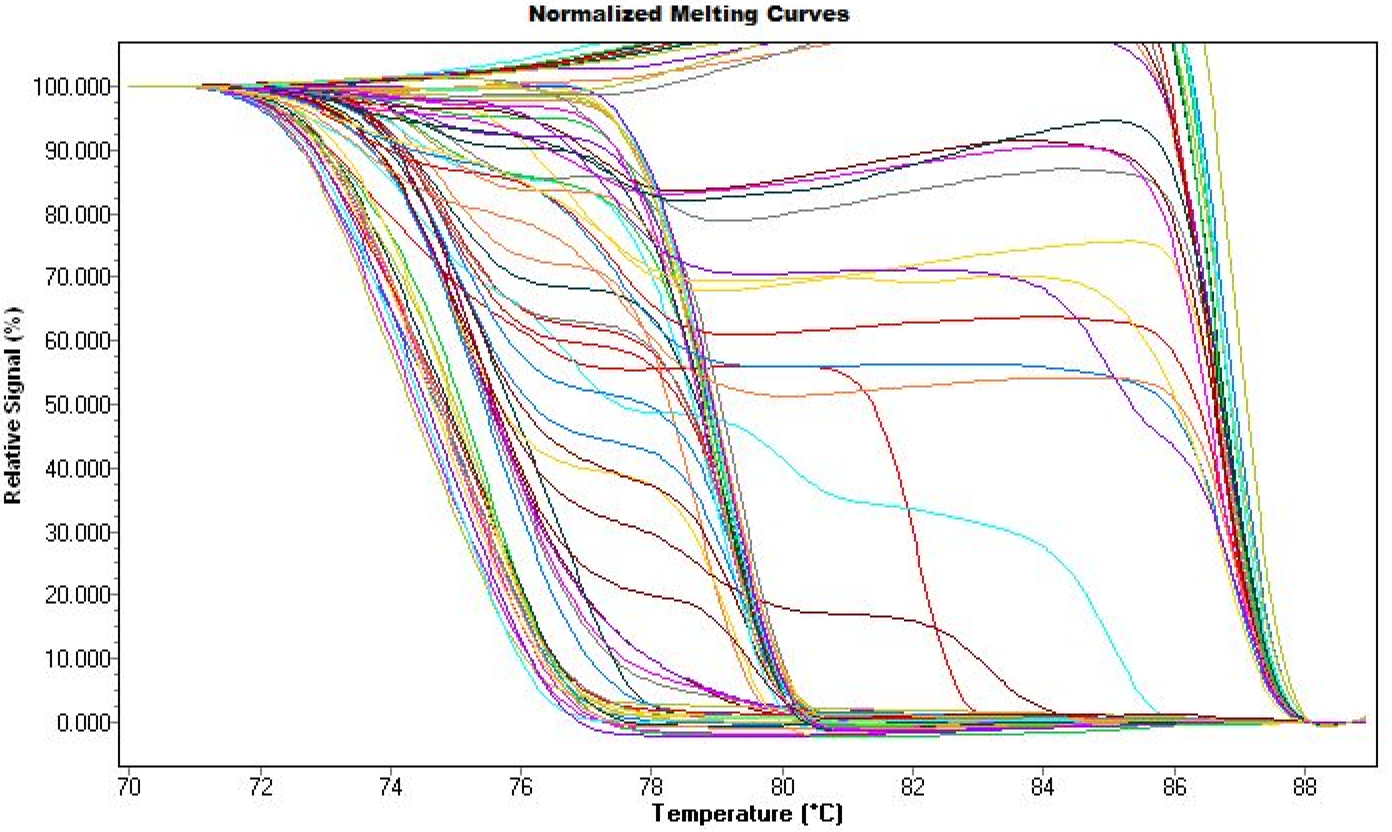
Normalized Melting Curves
Relative Signal (%)
100.000
90.000
80.000
70.000
60.000
50.000
40.000
30.000
20.000
10.000
0.000
70
72
74
76
78
80
82
84
86
88
Temperature (°C)

Normalized and Shifted Melting Curves

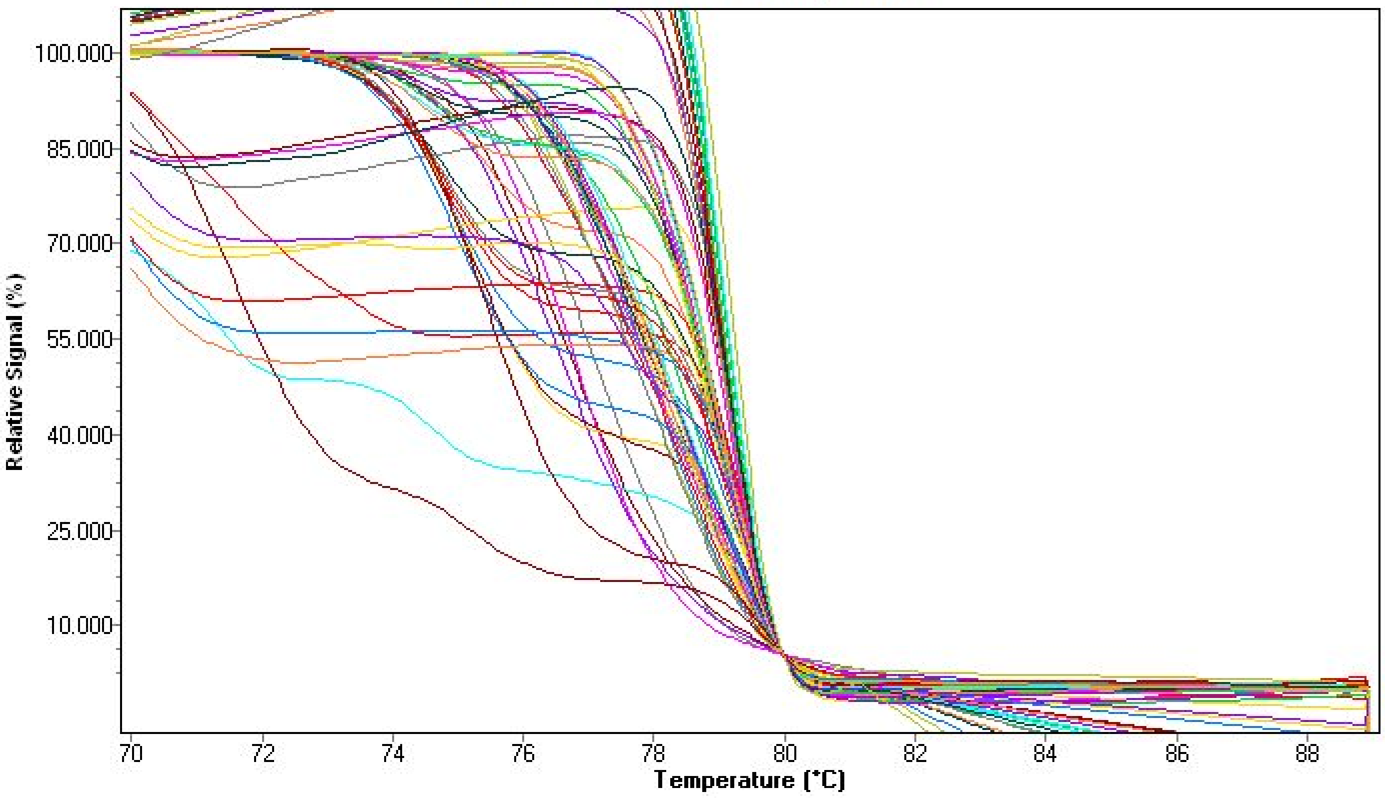

Normalized and Temp-Shifted Difference Plot

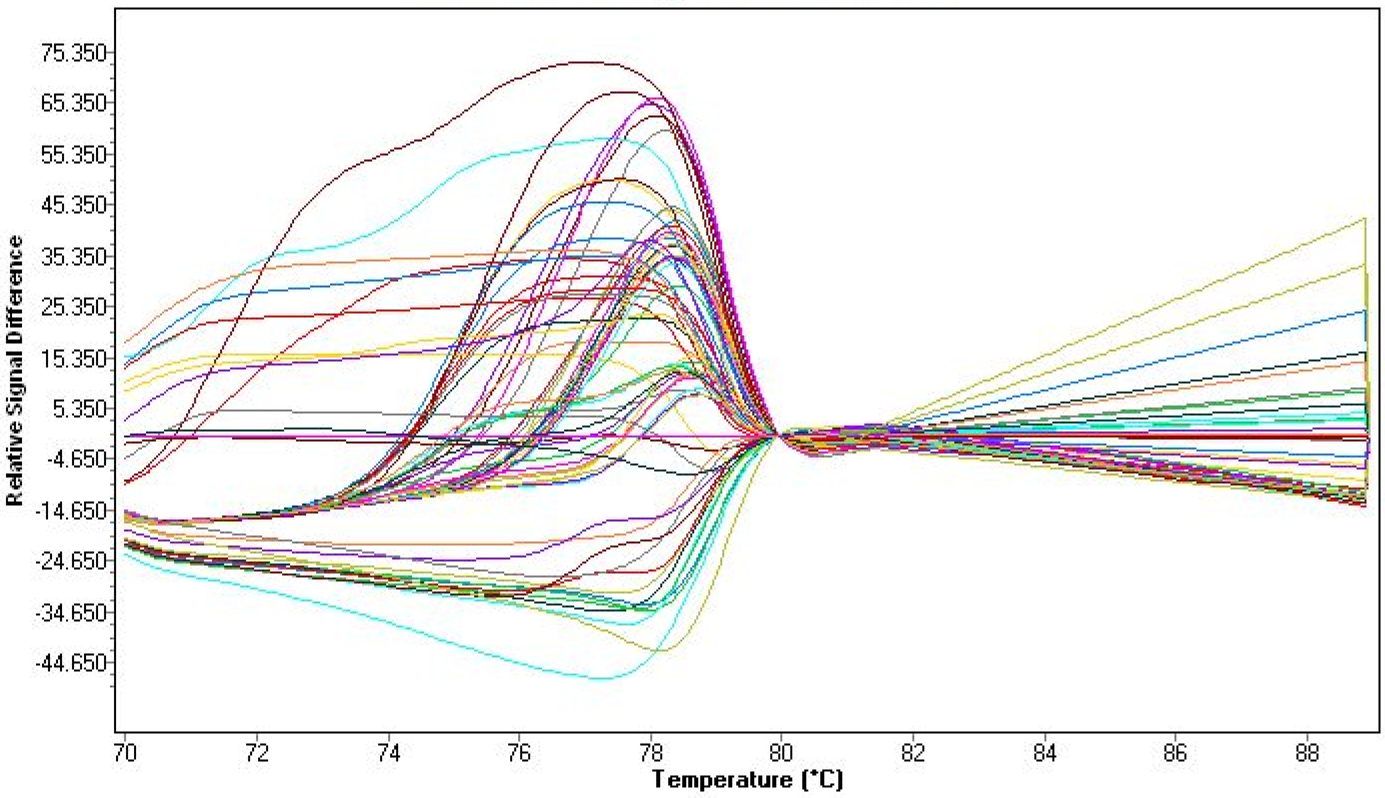

Analysis Notes	

Melt Curve Genotyping for All Samples (Melt Curve Genotyping)

Channel	465-510	Color Compensation	Off				
Progam Name	Program						
Stds Settings	Auto-Group						
Sensitivity	Normal	Temp Range	62-86	Score	0.7	Res	0.1

Subset Name	All Samples

Results

Inc	Pos.	Sample Name	Sample Type	Group	Score	Resolution	Status
☑	A1	GAPDH 9	Unknown	1	0.99	0.51	
☑	A2	GAPDH 9	Unknown	6	1.00	0.47	
☑	A3	GAPDH 9	Unknown	1	0.98	0.78	
☑	A4	GAPDH 10	Unknown	1	0.98	0.55	
☑	A5	GAPDH 10	Unknown	1	0.98	0.42	
☑	A6	GAPDH 10	Unknown	1	0.98	0.57	
☑	A7	GAPDH 11	Unknown	1	0.98	0.36	
☑	A8	GAPDH 11	Unknown	6	0.82	0.13	
☑	A9	GAPDH 11	Unknown	1	0.98	0.36	
☑	A10	GAPDH 12	Unknown	4	0.85	0.22	
☑	A11	GAPDH 12	Unknown	1	0.96	0.31	
☑	A12	GAPDH 12	Unknown	1	0.99	0.67	
☑	B1	FCER1A 9	Unknown	4	0.97	0.42	
☑	B2	FCER1A 9	Unknown	4	1.00	0.51	
☑	B3	FCER1A 9	Unknown	3	0.98	0.60	
☑	B4	FCER1A 10	Unknown	1	0.97	0.43	
☑	B5	FCER1A 10	Unknown	3	0.81	0.17	
☑	B6	FCER1A 10	Unknown	4	0.87	0.20	
☑	B7	FCER1A 11	Unknown	4	0.86	0.16	
☑	B8	FCER1A 11	Unknown	3	0.90	0.36	
☑	B9	FCER1A 11	Unknown	4	0.95	0.43	
☑	B10	FCER1A 12	Unknown	3	0.99	0.68	
☑	B11	FCER1A 12	Unknown	3	0.96	0.51	
☑	B12	FCER1A 12	Unknown	3	0.99	0.69	
☑	C1	RGS1 9	Unknown	2	0.99	0.36	
☑	C2	RGS1 9	Unknown	Unknown	0.77	0.05	
☑	C3	RGS1 9	Unknown	2	0.99	0.42	
☑	C4	RGS1 10	Unknown	2	0.90	0.47	
☑	C5	RGS1 10	Unknown	5	0.94	0.18	
☑	C6	RGS1 10	Unknown	5	0.84	0.14	
☑	C7	RGS1 1	Unknown	Unknown	0.90	0.03	

Results

Inc	Pos.	Sample Name	Sample Type	Group	Score	Resolution	Status
☑	C8	RGS1 1	Unknown	2	0.99	0.43	
☑	C9	RGS1 1	Unknown	2	1.00	0.37	
☑	C10	RGS1 12	Unknown	2	0.98	0.26	
☑	C11	RGS1 12	Unknown	2	0.96	0.20	
☑	C12	RGS1 12	Unknown	2	0.98	0.28	
☑	D1	GAPDH 13	Unknown	1	0.99	0.46	
☑	D2	GAPDH 13	Unknown	1	0.99	0.51	
☑	D3	GAPDH 13	Unknown	1	0.98	0.47	
☑	D4	GAPDH 14	Unknown	1	0.99	0.61	
☑	D5	GAPDH 14	Unknown	1	0.98	0.52	
☑	D6	GAPDH 14	Unknown	1	0.97	0.53	
☑	D7	GAPDH 15	Unknown	Unknown	0.72	0.03	
☑	D8	GAPDH 15	Unknown	1	0.99	0.63	
☑	D9	GAPDH 15	Unknown	1	1.00	0.51	
☑	D10	GAPDH 16	Unknown	1	0.99	0.55	
☑	D11	GAPDH 16	Unknown	1	0.99	0.58	
☑	D12	GAPDH 16	Unknown	1	0.98	0.50	
☑	E1	FCER1A 13	Unknown	4	0.90	0.25	
☑	E2	FCER1A 13	Unknown	4	0.99	0.49	
☑	E3	FCER1A 13	Unknown	Unknown	0.77	0.07	
☑	E4	FCER1A 14	Unknown	3	1.00	0.65	
☑	E5	FCER1A 14	Unknown	4	0.96	0.49	
☑	E6	FCER1A 14	Unknown	3	0.98	0.68	
☑	E7	FCER1A 15	Unknown	1	0.91	0.28	
☑	E8	FCER1A 15	Unknown	3	0.98	0.68	
☑	E9	FCER1A 15	Unknown	3	0.95	0.47	
☑	E10	FCER1A 16	Unknown	3	0.81	0.16	
☑	E11	FCER1A 16	Unknown	3	0.99	0.68	
☑	E12	FCER1A 16	Unknown	3	0.98	0.70	
☑	F1	RGS1 13	Unknown	5	0.83	0.12	
☑	F2	RGS1 13	Unknown	2	0.98	0.33	
☑	F3	RGS1 13	Unknown	2	0.99	0.43	
☑	F4	RGS1 14	Unknown	Unknown	0.88	0.01	
☑	F5	RGS1 14	Unknown	2	0.97	0.31	
☑	F6	RGS1 14	Unknown	5	0.88	0.10	
☑	F7	RGS1 15	Unknown	2	0.98	0.38	
☑	F8	RGS1 15	Unknown	2	0.97	0.49	
☑	F9	RGS1 15	Unknown	Unknown	0.85		

Results

Inc	Pos.	Sample Name	Sample Type	Group	Score	Resolution	Status
☑	F10	RGS1 16	Unknown	Unknown	0.79	0.09	
☑	F11	RGS1 16	Unknown	5	1.00	0.37	
☑	F12	RGS1 16	Unknown	2	0.98	0.34	
☑	G1	blank	Unknown	Negative	1.00	1.00	
☑	G2	blank	Unknown	Negative	1.00	1.00	
☑	G3	blank	Unknown	Negative	1.00	1.00	
☑	G4	blank	Unknown	Negative	1.00	1.00	
☑	G5	blank	Unknown	Negative	1.00	1.00	
☑	G6	blank	Unknown	Negative	1.00	1.00	
☑	G7	blank	Unknown	Negative	1.00	1.00	
☑	G8	blank	Unknown	Negative	1.00	1.00	
☑	G9	blank	Unknown	Negative	1.00	1.00	
☑	G10	blank	Unknown	Negative	1.00	1.00	
☑	G11	blank	Unknown	Negative	1.00	1.00	
☑	G12	blank	Unknown	Negative	1.00	1.00	
☑	H1	blank	Unknown	Negative	1.00	1.00	
☑	H2	blank	Unknown	Negative	1.00	1.00	
☑	H3	blank	Unknown	Negative	1.00	1.00	
☑	H4	blank	Unknown	Negative	1.00	1.00	
☑	H5	blank	Unknown	Negative	1.00	1.00	
☑	H6	blank	Unknown	Negative	1.00	1.00	
☑	H7	blank	Unknown	Negative	1.00	1.00	
☑	H8	blank	Unknown	Negative	1.00	1.00	
☑	H9	blank	Unknown	Negative	1.00	1.00	
☑	H10	blank	Unknown	Negative	1.00	1.00	
☑	H11	blank	Unknown	Negative	1.00	1.00	
☑	H12	blank	Unknown	Negative	1.00	1.00	

**Group**	1

Inc	Pos	Sample Name	Sample Type	Score	Resolution	Status
☑	A1	GAPDH 9	Unknown	0.99	0.51	
☑	A3	GAPDH 9	Unknown	0.98	0.78	
☑	A4	GAPDH 10	Unknown	0.98	0.55	
☑	A5	GAPDH 10	Unknown	0.98	0.42	
☑	A6	GAPDH 10	Unknown	0.98	0.57	
☑	A7	GAPDH 11	Unknown	0.98	0.36	
☑	A9	GAPDH 11	Unknown	0.98	0.36	
☑	A11	GAPDH 12	Unknown	0.96	0.31	

Group	1

Inc	Pos	Sample Name	Sample Type	Score	Resolution	Status
☑	A12	GAPDH 12	Unknown	0.99	0.67	
☑	B4	FCER1A 10	Unknown	0.97	0.43	
☑	D1	GAPDH 13	Unknown	0.99	0.46	
☑	D2	GAPDH 13	Unknown	0.99	0.51	
☑	D3	GAPDH 13	Unknown	0.98	0.47	
☑	D4	GAPDH 14	Unknown	0.99	0.61	
☑	D5	GAPDH 14	Unknown	0.98	0.52	
☑	D6	GAPDH 14	Unknown	0.97	0.53	
☑	D8	GAPDH 15	Unknown	0.99	0.63	
☑	D9	GAPDH 15	Unknown	1.00	0.51	
☑	D10	GAPDH 16	Unknown	0.99	0.55	
☑	D11	GAPDH 16	Unknown	0.99	0.58	
☑	D12	GAPDH 16	Unknown	0.98	0.50	
☑	E7	FCER1A 15	Unknown	0.91	0.28	

Group	2

Inc	Pos	Sample Name	Sample Type	Score	Resolution	Status
☑	C1	RGS1 9	Unknown	0.99	0.36	
☑	C3	RGS1 9	Unknown	0.99	0.42	
☑	C4	RGS1 10	Unknown	0.90	0.47	
☑	C8	RGS1 1	Unknown	0.99	0.43	
☑	C9	RGS1 1	Unknown	1.00	0.37	
☑	C10	RGS1 12	Unknown	0.98	0.26	
☑	C11	RGS1 12	Unknown	0.96	0.20	
☑	C12	RGS1 12	Unknown	0.98	0.28	
☑	F2	RGS1 13	Unknown	0.98	0.33	
☑	F3	RGS1 13	Unknown	0.99	0.43	
☑	F5	RGS1 14	Unknown	0.97	0.31	
☑	F7	RGS1 15	Unknown	0.98	0.38	
☑	F8	RGS1 15	Unknown	0.97	0.49	
☑	F12	RGS1 16	Unknown	0.98	0.34	

Group	3

Inc	Pos	Sample Name	Sample Type	Score	Resolution	Status
☑	B3	FCER1A 9	Unknown	0.98	0.60	
☑	B5	FCER1A 10	Unknown	0.81	0.17	
☑	B8	FCER1A 11	Unknown	0.90	0.36	
☑	B10	FCER1A 12	Unknown	0.99	0.68	

Group	3

Inc	Pos	Sample Name	Sample Type	Score	Resolution	Status
☑	B11	FCER1A 12	Unknown	0.96	0.51	
☑	B12	FCER1A 12	Unknown	0.99	0.69	
☑	E4	FCER1A 14	Unknown	1.00	0.65	
☑	E6	FCER1A 14	Unknown	0.98	0.68	
☑	E8	FCER1A 15	Unknown	0.98	0.68	
☑	E9	FCER1A 15	Unknown	0.95	0.47	
☑	E10	FCER1A 16	Unknown	0.81	0.16	
☑	E11	FCER1A 16	Unknown	0.99	0.68	
☑	E12	FCER1A 16	Unknown	0.98	0.70	

Group	4

Inc	Pos	Sample Name	Sample Type	Score	Resolution	Status
☑	A10	GAPDH 12	Unknown	0.85	0.22	
☑	B1	FCER1A 9	Unknown	0.97	0.42	
☑	B2	FCER1A 9	Unknown	1.00	0.51	
☑	B6	FCER1A 10	Unknown	0.87	0.20	
☑	B7	FCER1A 11	Unknown	0.86	0.16	
☑	B9	FCER1A 11	Unknown	0.95	0.43	
☑	E1	FCER1A 13	Unknown	0.90	0.25	
☑	E2	FCER1A 13	Unknown	0.99	0.49	
☑	E5	FCER1A 14	Unknown	0.96	0.49	

Group	5

Inc	Pos	Sample Name	Sample Type	Score	Resolution	Status
☑	C5	RGS1 10	Unknown	0.94	0.18	
☑	C6	RGS1 10	Unknown	0.84	0.14	
☑	F1	RGS1 13	Unknown	0.83	0.12	
☑	F6	RGS1 14	Unknown	0.88	0.10	
☑	F11	RGS1 16	Unknown	1.00	0.37	

Group	6

Inc	Pos	Sample Name	Sample Type	Score	Resolution	Status
☑	A2	GAPDH 9	Unknown	1.00	0.47	
☑	A8	GAPDH 11	Unknown	0.82	0.13	

Group	Negative

Inc	Pos	Sample Name	Sample Type	Score	Resolution	Status

Group	Negative

Inc	Pos	Sample Name	Sample Type	Score	Resolution	Status
☑	G1	blank	Unknown	1.00	1.00	
☑	G2	blank	Unknown	1.00	1.00	
☑	G3	blank	Unknown	1.00	1.00	
☑	G4	blank	Unknown	1.00	1.00	
☑	G5	blank	Unknown	1.00	1.00	
☑	G6	blank	Unknown	1.00	1.00	
☑	G7	blank	Unknown	1.00	1.00	
☑	G8	blank	Unknown	1.00	1.00	
☑	G9	blank	Unknown	1.00	1.00	
☑	G10	blank	Unknown	1.00	1.00	
☑	G11	blank	Unknown	1.00	1.00	
☑	G12	blank	Unknown	1.00	1.00	
☑	H1	blank	Unknown	1.00	1.00	
☑	H2	blank	Unknown	1.00	1.00	
☑	H3	blank	Unknown	1.00	1.00	
☑	H4	blank	Unknown	1.00	1.00	
☑	H5	blank	Unknown	1.00	1.00	
☑	H6	blank	Unknown	1.00	1.00	
☑	H7	blank	Unknown	1.00	1.00	
☑	H8	blank	Unknown	1.00	1.00	
☑	H9	blank	Unknown	1.00	1.00	
☑	H10	blank	Unknown	1.00	1.00	
☑	H11	blank	Unknown	1.00	1.00	
☑	H12	blank	Unknown	1.00	1.00	

Group	Unknown

Inc	Pos	Sample Name	Sample Type	Score	Resolution	Status
☑	C2	RGS1 9	Unknown	0.77	0.05	
☑	C7	RGS1 1	Unknown	0.90	0.03	
☑	D7	GAPDH 15	Unknown	0.72	0.03	
☑	E3	FCER1A 13	Unknown	0.77	0.07	
☑	F4	RGS1 14	Unknown	0.88	0.01	
☑	F9	RGS1 15	Unknown	0.85		
☑	F10	RGS1 16	Unknown	0.79	0.09	

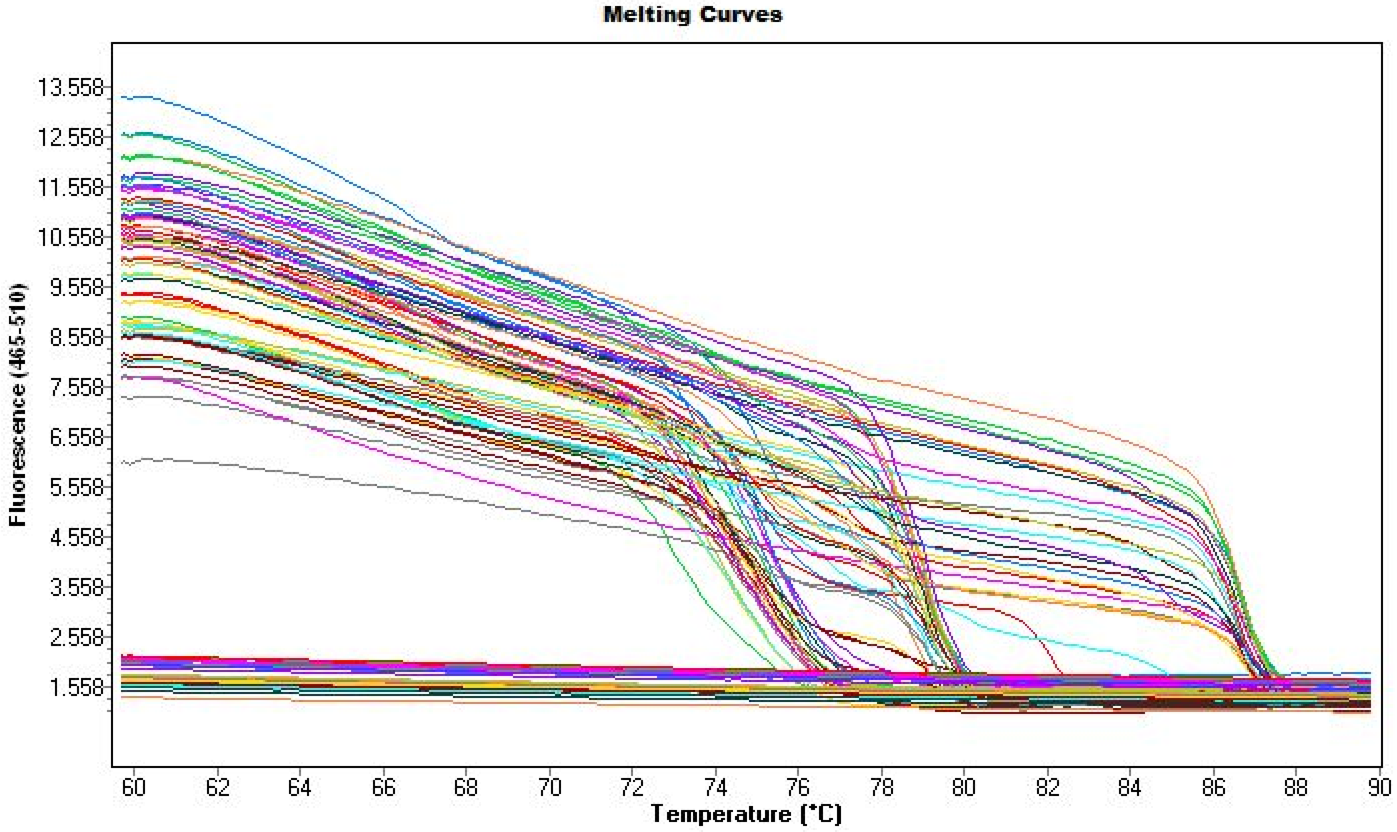

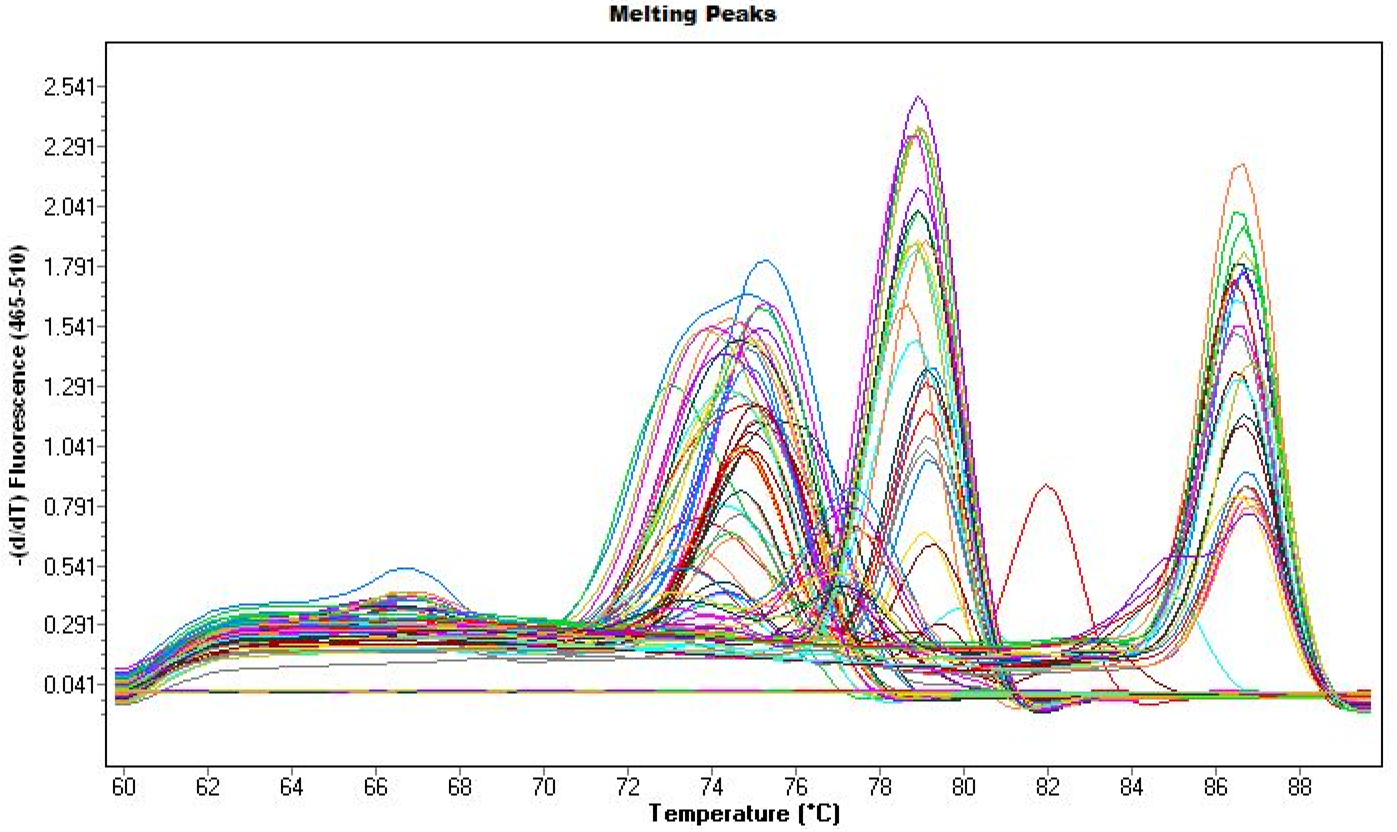

Analysis Notes	

Tm Calling for All Samples (Tm Calling)

Settings

Channel	465-510	Color Compensation	Off		
Program	Program				
Show Shoulders	False	Chemistry	HybProbe	Max Peaks	2

Subset Name	All Samples

Results

Inc	Pos	Sample Name	Peak 1 Tm	Peak 1 Area	Peak 1 Width	Peak 1 Height	Peak 2 Tm	Peak 2 Area	Peak 2 Width	Peak 2 Height	Status
☑	A1	GAPDH 9	74.51	8.16	4.45	1.84					
☑	A2	GAPDH 9	74.13	1.62	2.71	0.60	82.07	2.19	2.43	0.90	
☑	A3	GAPDH 9	65.16	2.14	8.23	0.26	73.33	4.89	3.82	1.28	
☑	A4	GAPDH 10	74.61	6.39	3.89	1.64					
☑	A5	GAPDH 10	75.42	3.77	3.26	1.16					
☑	A6	GAPDH 10	74.44	5.01	3.75	1.34					
☑	A7	GAPDH 11	75.17	3.84	3.58	1.07					
☑	A8	GAPDH 11	74.33	2.96	4.41	0.67					
☑	A9	GAPDH 11	75.90	4.38	3.57	1.23					
☑	A10	GAPDH 12	74.65	1.40	2.01	0.70	78.63	3.74	2.21	1.69	
☑	A11	GAPDH 12	75.17	5.34	3.65	1.47					
☑	A12	GAPDH 12	74.01	6.01	3.73	1.61					
☑	B1	FCER1A 9	74.95	3.22	2.32	1.39	79.30	3.41	2.29	1.49	
☑	B2	FCER1A 9	74.78	1.92	2.15	0.89	79.21	2.86	2.32	1.23	
☑	B3	FCER1A 9	78.90	5.87	2.39	2.46					
☑	B4	FCER1A 10	75.42	4.76	3.00	1.59					
☑	B5	FCER1A 10	74.57	0.55	2.28	0.24	79.20	2.54	2.26	1.13	
☑	B6	FCER1A 10	74.79	2.64	2.49	1.06	79.13	1.72	2.20	0.78	
☑	B7	FCER1A 11	75.06	2.71	2.58	1.05	79.32	1.68	2.35	0.72	
☑	B8	FCER1A 11	74.11	0.56	1.94	0.29	78.82	3.65	2.38	1.53	
☑	B9	FCER1A 11	74.66	1.58	2.21	0.72	79.17	3.29	2.31	1.42	
☑	B10	FCER1A 12	78.98	5.94	2.41	2.47					
☑	B11	FCER1A 12	74.10	0.38	1.63	0.23	78.96	5.19	2.35	2.21	
☑	B12	FCER1A 12	78.98	5.89	2.38	2.47					
☑	C1	RGS1 9									
☑	C2	RGS1 9	76.24	1.64	4.31	0.38					
☑	C3	RGS1 9									
☑	C4	RGS1 10	66.09	0.73	5.27	0.14					
☑	C5	RGS1 10	77.40	0.40	1.11	0.36					

Results

Inc	Pos	Sample Name	Peak 1 Tm	Peak 1 Area	Peak 1 Width	Peak 1 Height	Peak 2 Tm	Peak 2 Area	Peak 2 Width	Peak 2 Height	Status
☑	C6	RGS1 10	76.63	0.82	1.53	0.53					
☑	C7	RGS1 1									
☑	C8	RGS1 1	86.61	4.24	2.44	1.74					
☑	C9	RGS1 1	86.63	4.61	2.45	1.88					
☑	C10	RGS1 12	86.65	5.57	2.41	2.31					
☑	C11	RGS1 12									
☑	C12	RGS1 12									
☑	D1	GAPDH 13	66.18	3.24	8.45	0.38	75.17	6.60	3.66	1.80	
☑	D2	GAPDH 13	74.63	5.49	4.20	1.31					
☑	D3	GAPDH 13	75.22	5.47	3.32	1.65					
☑	D4	GAPDH 14	65.87	2.50	7.37	0.34	74.18	6.38	3.92	1.63	
☑	D5	GAPDH 14	74.61	5.09	3.88	1.31					
☑	D6	GAPDH 14	74.87	5.42	3.52	1.54					
☑	D7	GAPDH 15	65.50	2.10	9.22	0.23	74.91	3.29	3.36	0.98	
☑	D8	GAPDH 15	74.33	5.01	3.70	1.35					
☑	D9	GAPDH 15	74.71	6.32	3.98	1.59					
☑	D10	GAPDH 16	66.33	2.79	7.63	0.37	74.52	6.24	3.76	1.66	
☑	D11	GAPDH 16	74.39	5.83	3.85	1.52					
☑	D12	GAPDH 16	74.89	5.89	3.87	1.52					
☑	E1	FCER1A 13	74.97	3.48	2.47	1.41	79.28	2.57	2.30	1.12	
☑	E2	FCER1A 13	74.78	1.96	2.16	0.91	79.21	3.17	2.34	1.35	
☑	E3	FCER1A 13	74.42	0.92	2.07	0.45	78.97	5.11	2.43	2.10	
☑	E4	FCER1A 14	78.78	5.96	2.45	2.43					
☑	E5	FCER1A 14	74.69	1.46	2.20	0.66	79.14	2.39	2.26	1.06	
☑	E6	FCER1A 14	78.90	4.91	2.49	1.97					
☑	E7	FCER1A 15	75.17	4.10	3.49	1.17					
☑	E8	FCER1A 15	78.84	4.79	2.47	1.94					
☑	E9	FCER1A 15	74.06	0.54	1.94	0.28	78.91	5.12	2.42	2.11	
☑	E10	FCER1A 16	74.45	0.95	1.97	0.48	79.14	4.64	2.34	1.99	
☑	E11	FCER1A 16	78.96	6.16	2.37	2.60					
☑	E12	FCER1A 16	78.79	4.48	2.29	1.96					
☑	F1	RGS1 13	76.95	1.46	2.45	0.60					
☑	F2	RGS1 13	86.54	4.81	2.61	1.84					
☑	F3	RGS1 13	86.64	5.18	2.42	2.14					
☑	F4	RGS1 14	86.66	4.47	2.67	1.67					
☑	F5	RGS1 14	86.55	4.09	2.52	1.62					

Results

Inc	Pos	Sample Name	Peak 1				Peak 2				Status
			Tm	Area	Width	Height	Tm	Area	Width	Height	
☑	F6	RGS1 14	75.98	1.45	4.74	0.31	86.64	3.25	3.54	0.92	
☑	F7	RGS1 15	86.59	4.20	2.91	1.44					
☑	F8	RGS1 15	86.65	3.87	2.72	1.43					
☑	F9	RGS1 15	75.58	1.16	4.75	0.24					
☑	F10	RGS1 16	76.42	2.47	6.20	0.40					
☑	F11	RGS1 16	77.28	0.88	1.18	0.75					
☑	F12	RGS1 16									
☑	G1	blank									
☑	G2	blank									
☑	G3	blank									
☑	G4	blank									
☑	G5	blank									
☑	G6	blank									
☑	G7	blank									
☑	G8	blank									
☑	G9	blank									
☑	G10	blank									
☑	G11	blank									
☑	G12	blank									
☑	H1	blank									
☑	H2	blank									
☑	H3	blank									
☑	H4	blank									
☑	H5	blank									
☑	H6	blank									
☑	H7	blank									
☑	H8	blank									
☑	H9	blank									
☑	H10	blank									
☑	H11	blank									
☑	H12	blank									

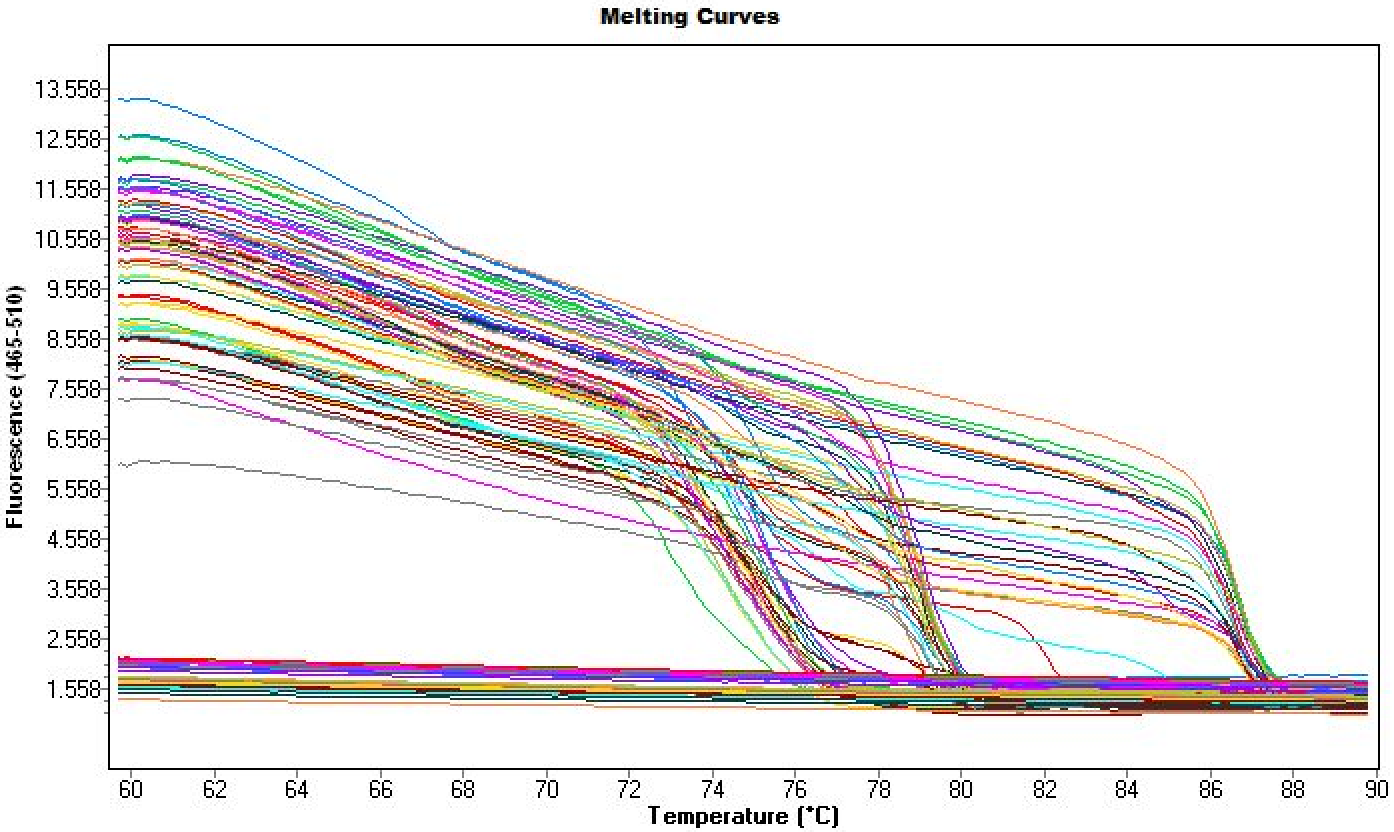

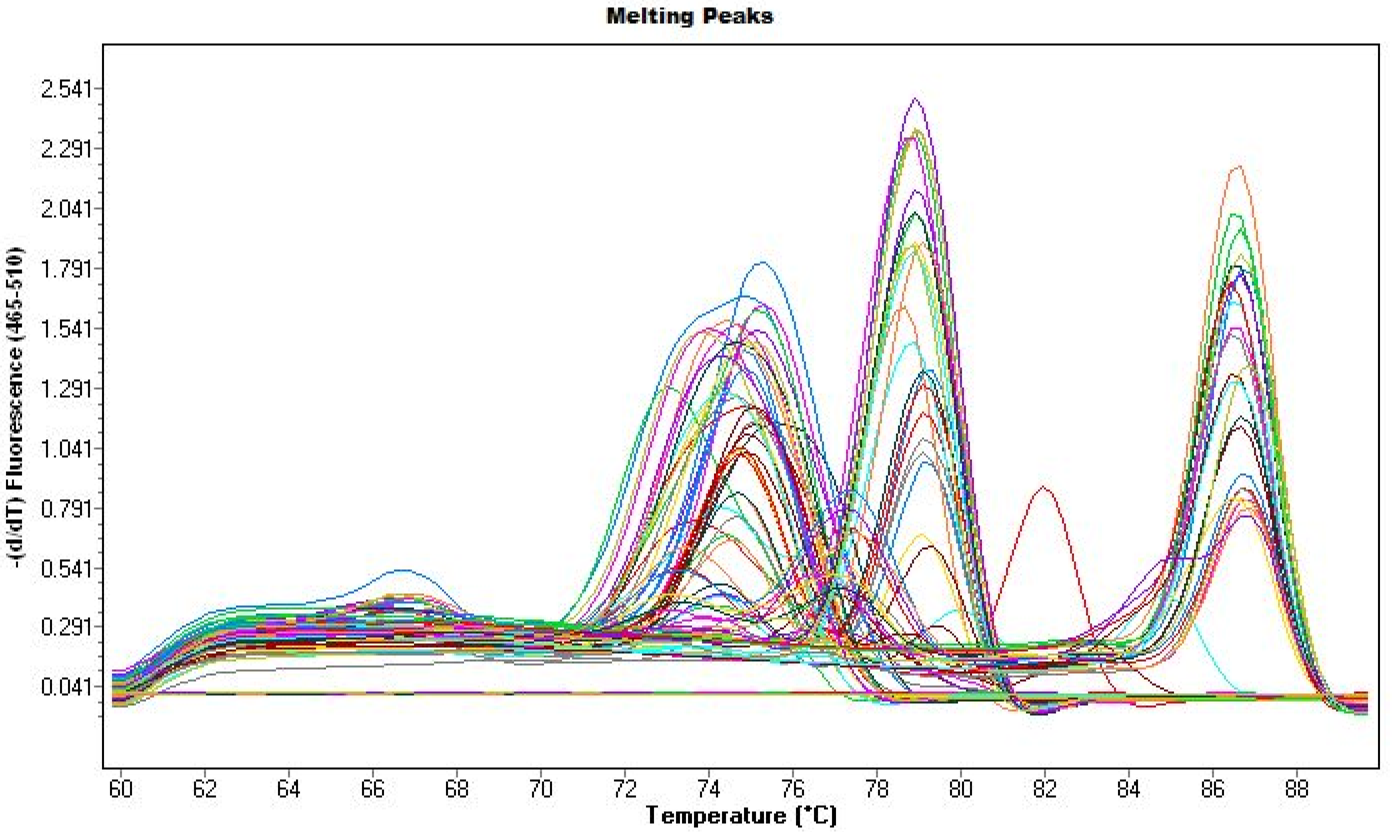

**Analysis Notes**	

Abs Quant/2nd Derivative Max for All Samples (1) (Abs Quant/2nd Derivative Max)

Settings

Channel	465-510		
Color Compensation	Off		
Program	Program	Units	
Mode	High Confidence		

Subset Name	All Samples

Results

Inc	Pos	Name	Type	CP	Concentration	Standard	Status
☑	A1	GAPDH 9	Unknown	30.70			
☑	A2	GAPDH 9	Unknown	32.82			
☑	A3	GAPDH 9	Unknown	35.00			>
☑	A4	GAPDH 10	Unknown	32.35			
☑	A5	GAPDH 10	Unknown	29.30			
☑	A6	GAPDH 10	Unknown	35.00			>
☑	A7	GAPDH 11	Unknown	25.51			
☑	A8	GAPDH 11	Unknown	32.47			
☑	A9	GAPDH 11	Unknown	35.00			>
☑	A10	GAPDH 12	Unknown	35.00			>
☑	A11	GAPDH 12	Unknown	28.74			
☑	A12	GAPDH 12	Unknown	35.00			>
☑	B1	FCER1A 9	Unknown	30.00			
☑	B2	FCER1A 9	Unknown	29.91			
☑	B3	FCER1A 9	Unknown	31.11			
☑	B4	FCER1A 10	Unknown	30.02			
☑	B5	FCER1A 10	Unknown	35.00			>
☑	B6	FCER1A 10	Unknown	31.51			
☑	B7	FCER1A 11	Unknown	29.17			
☑	B8	FCER1A 11	Unknown	31.13			
☑	B9	FCER1A 11	Unknown	30.67			
☑	B10	FCER1A 12	Unknown	30.94			
☑	B11	FCER1A 12	Unknown	30.75			
☑	B12	FCER1A 12	Unknown	31.26			
☑	C1	RGS1 9	Unknown	35.00			>
☑	C2	RGS1 9	Unknown	33.00			
☑	C3	RGS1 9	Unknown	35.00			>
☑	C4	RGS1 10	Unknown	35.00			>

> - Late Cp call (last five cycles) has higher uncertainty

Results

Inc	Pos	Name	Type	CP	Concentration	Standard	Status
☑	C5	RGS1 10	Unknown	35.00			>
☑	C6	RGS1 10	Unknown	35.00			>
☑	C7	RGS1 1	Unknown	34.54			
☑	C8	RGS1 1	Unknown	35.00			>
☑	C9	RGS1 1	Unknown	35.00			>
☑	C10	RGS1 12	Unknown	32.67			
☑	C11	RGS1 12	Unknown	33.81			
☑	C12	RGS1 12	Unknown	33.44			
☑	D1	GAPDH 13	Unknown	32.05			
☑	D2	GAPDH 13	Unknown	31.62			
☑	D3	GAPDH 13	Unknown	35.00			>
☑	D4	GAPDH 14	Unknown	32.88			
☑	D5	GAPDH 14	Unknown	31.08			
☑	D6	GAPDH 14	Unknown	35.00			>
☑	D7	GAPDH 15	Unknown	30.57			
☑	D8	GAPDH 15	Unknown	35.00			>
☑	D9	GAPDH 15	Unknown	33.36			
☑	D10	GAPDH 16	Unknown	33.20			
☑	D11	GAPDH 16	Unknown	35.00			>
☑	D12	GAPDH 16	Unknown	33.35			
☑	E1	FCER1A 13	Unknown	29.81			
☑	E2	FCER1A 13	Unknown	29.49			
☑	E3	FCER1A 13	Unknown	31.04			
☑	E4	FCER1A 14	Unknown	29.49			
☑	E5	FCER1A 14	Unknown	28.67			
☑	E6	FCER1A 14	Unknown	28.74			
☑	E7	FCER1A 15	Unknown	29.11			
☑	E8	FCER1A 15	Unknown	32.33			
☑	E9	FCER1A 15	Unknown	30.88			
☑	E10	FCER1A 16	Unknown	29.97			
☑	E11	FCER1A 16	Unknown	30.96			
☑	E12	FCER1A 16	Unknown	30.64			
☑	F1	RGS1 13	Unknown	31.90			
☑	F2	RGS1 13	Unknown	33.90			
☑	F3	RGS1 13	Unknown	35.00			>
☑	F4	RGS1 14	Unknown	32.42			
☑	F5	RGS1 14	Unknown	32.70			

> - Late Cp call (last five cycles) has higher uncertainty

Results

Inc	Pos	Name	Type	CP	Concentration	Standard	Status
☑	F6	RGS1 14	Unknown	31.23			
☑	F7	RGS1 15	Unknown	35.00			>
☑	F8	RGS1 15	Unknown	35.00			>
☑	F9	RGS1 15	Unknown	33.13			
☑	F10	RGS1 16	Unknown	32.74			
☑	F11	RGS1 16	Unknown	33.21			
☑	F12	RGS1 16	Unknown	35.00			>
☑	G1	blank	Unknown				
☑	G2	blank	Unknown				
☑	G3	blank	Unknown				
☑	G4	blank	Unknown				
☑	G5	blank	Unknown				
☑	G6	blank	Unknown				
☑	G7	blank	Unknown				
☑	G8	blank	Unknown				
☑	G9	blank	Unknown				
☑	G10	blank	Unknown				
☑	G11	blank	Unknown				
☑	G12	blank	Unknown				
☑	H1	blank	Unknown				
☑	H2	blank	Unknown				
☑	H3	blank	Unknown				
☑	H4	blank	Unknown				
☑	H5	blank	Unknown				
☑	H6	blank	Unknown				
☑	H7	blank	Unknown				
☑	H8	blank	Unknown				
☑	H9	blank	Unknown				
☑	H10	blank	Unknown				
☑	H11	blank	Unknown				
☑	H12	blank	Unknown				

> - Late Cp call (last five cycles) has higher uncertainty

Amplification Curves

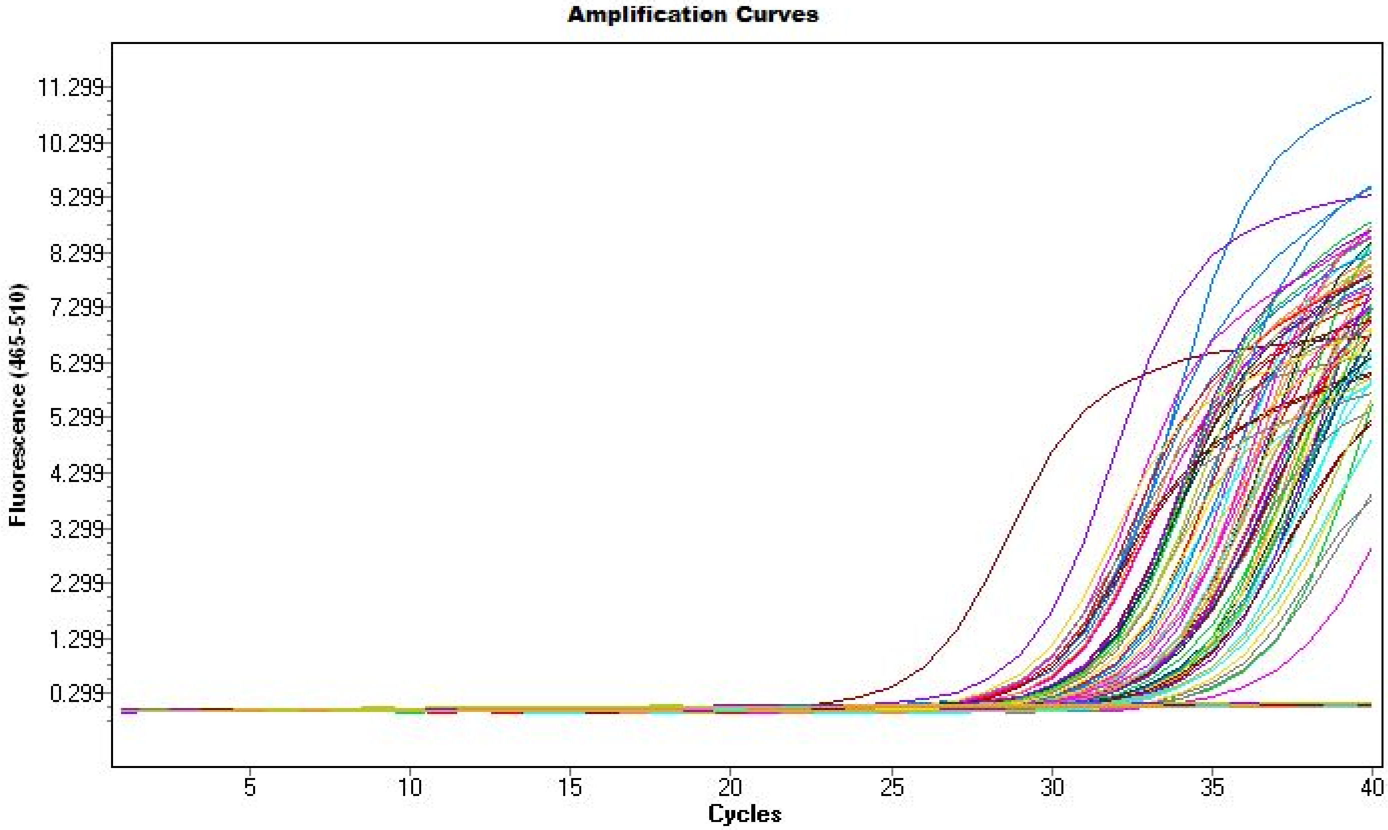

Standard Curve

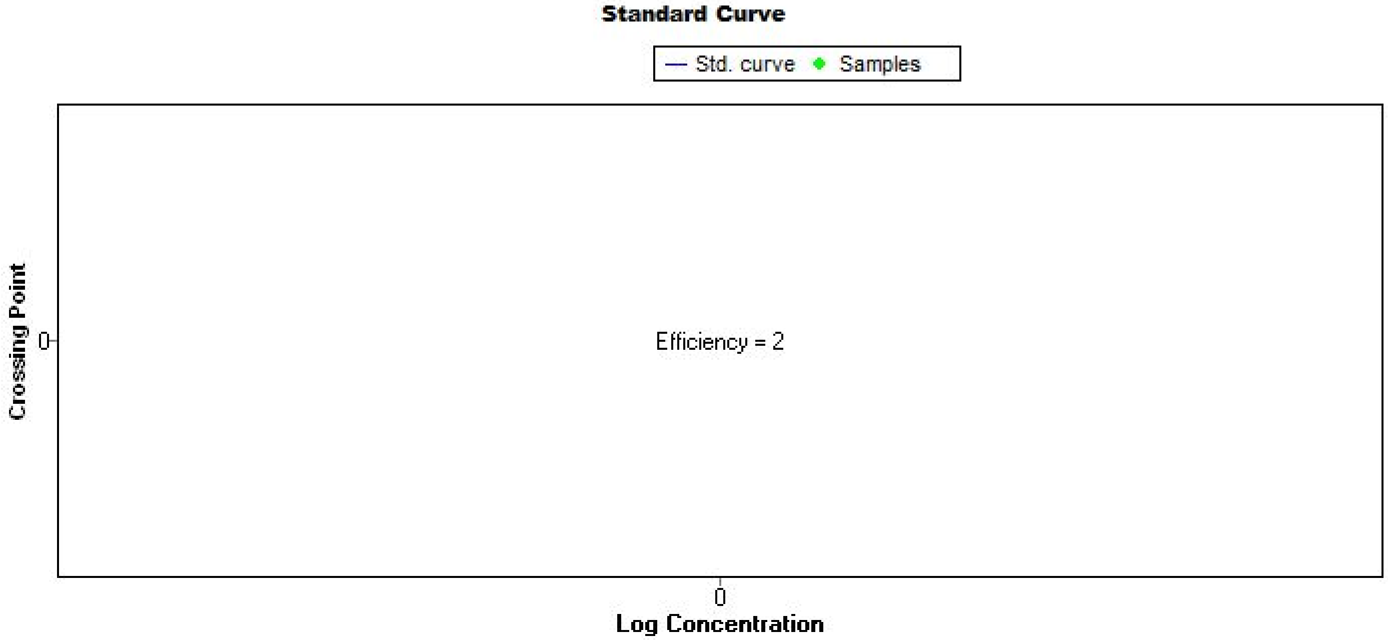

Analysis Notes	
