# Supplementary material for: Exploring differential gene expression and biomarker potential in systemic lupus erythematosus: a retrospective study
Source: PeerJ. 2025 Sep 8;13:e19891. doi: 10.7717/peerj.19891 (PMC12424612; doi:10.7717/peerj.19891)
Supplement: Supplemental Information 2 — These data are new and were recently supplemented with samples. The data include the Ct value for each sample, the Ct value for reference genes (e.g., GAPDH), and the relative expression levels calculated using the ΔΔCt method. These data were used to validate the RNA sequencing results and further evaluate the potential of these genes as biomarkers for SLE. [file peerj-13-19891-s002.zip › 20250528xj/20250528 SLE1.PDF]

cobas® 4800 User Defined Workflow (UDF)

xj20250528 SLE1

Experiment

Creation Date	5/28/2025 10:50:16 AM	Last Modified Date	6/2/2025 2:43:41 AM
Operator	System Admin	Owner	System Admin
Start Time	5/28/2025 10:50:43 AM	End Time	5/28/2025 11:50:16 AM
Run State	Completed	Software Version	LCS480 1.5.1.62 SP3 - UDF 2.1.0.26
Macro		Macro Owner	
Macro Status			
Templates	gongying	Plate ID	
Test ID		Lot ID	
Color Comp ID			
Run Notes			

Programs

Program Name	Program		
Cycles	1	Analysis Mode	None

Target (°C)	Acquisition Mode	Hold (hh:mm:ss)	Ramp Rate (°C/s)	Acquisitions (per °C)	Sec Target (°C)	Step size (°C)	Step Delay (cycles)
95	None	00:00:30	4.40		0	0	0

Program Name	Program		
Cycles	40	Analysis Mode	Quantification

Target (°C)	Acquisition Mode	Hold (hh:mm:ss)	Ramp Rate (°C/s)	Acquisitions (per °C)	Sec Target (°C)	Step size (°C)	Step Delay (cycles)
95	None	00:00:15	4.40		0	0	0
60	Single	00:00:30	2.20		0	0	0

Program Name	Program		
Cycles	1	Analysis Mode	Melting Curves

Target (°C)	Acquisition Mode	Hold (hh:mm:ss)	Ramp Rate (°C/s)	Acquisitions (per °C)	Sec Target (°C)	Step size (°C)	Step Delay (cycles)
95	None	00:01:00	4.40		0	0	0
60	None	00:00:30	2.20		0	0	0
90	Continuous		0.11	5	0	0	0

Program Name	Program		
Cycles	1	Analysis Mode	None

Target (°C)	Acquisition Mode	Hold (hh:mm:ss)	Ramp Rate (°C/s)	Acquisitions (per °C)	Sec Target (°C)	Step size (°C)	Step Delay (cycles)

Target (°C)	Acquisition Mode	Hold (hh:mm:ss)	Ramp Rate (°C/s)	Acquisitions (per °C)	Sec Target (°C)	Step size (°C)	Step Delay (cycles)
40	None	00:00:30	2.20		0	0	0

Samples

Sample Count	96
Subset	All Samples

Pos	Name	ID	Repl. Of	Sample Notes
A1	GAPDH 1			
A2	GAPDH 1			
A3	GAPDH 1			
A4	GAPDH 2			
A5	GAPDH 2			
A6	GAPDH 2			
A7	GAPDH 3			
A8	GAPDH 3			
A9	GAPDH 3			
A10	GAPDH 4			
A11	GAPDH 4			
A12	GAPDH 4			
B1	FCER1A 1			
B2	FCER1A 1			
B3	FCER1A 1			
B4	FCER1A 2			
B5	FCER1A 2			
B6	FCER1A 2			
B7	FCER1A 3			
B8	FCER1A 3			
B9	FCER1A 3			
B10	FCER1A 4			
B11	FCER1A 4			
B12	FCER1A 4			
C1	RGS1 1			
C2	RGS1 1			
C3	RGS1 1			
C4	RGS1 2			
C5	RGS1 2			
C6	RGS1 2			
C7	RGS1 3			
C8	RGS1 3			
C9	RGS1 3			
C10	RGS1 4			
C11	RGS1 4			
C12	RGS1 4			

Samples

Sample Count	96
Subset	All Samples

Pos	Name	ID	Repl. Of	Sample Notes
D1	GAPDH 5			
D2	GAPDH 5			
D3	GAPDH 5			
D4	GAPDH 6			
D5	GAPDH 6			
D6	GAPDH 6			
D7	GAPDH 7			
D8	GAPDH 7			
D9	GAPDH 7			
D10	GAPDH 8			
D11	GAPDH 8			
D12	GAPDH 8			
E1	FCER1A 5			
E2	FCER1A 5			
E3	FCER1A 5			
E4	FCER1A 6			
E5	FCER1A 6			
E6	FCER1A 6			
E7	FCER1A 7			
E8	FCER1A 7			
E9	FCER1A 7			
E10	FCER1A 8			
E11	FCER1A 8			
E12	FCER1A 8			
F1	RGS1 5			
F2	RGS1 5			
F3	RGS1 5			
F4	RGS1 6			
F5	RGS1 6			
F6	RGS1 6			
F7	RGS1 7			
F8	RGS1 7			
F9	RGS1 7			
F10	RGS1 8			
F11	RGS1 8			
F12	RGS1 8			
G1	blank			
G2	blank			
G3	blank			

Samples

Sample Count	96
Subset	All Samples

Pos	Name	ID	Repl. Of	Sample Notes
G4	blank			
G5	blank			
G6	blank			
G7	blank			
G8	blank			
G9	blank			
G10	blank			
G11	blank			
G12	blank			
H1	blank			
H2	blank			
H3	blank			
H4	blank			
H5	blank			
H6	blank			
H7	blank			
H8	blank			
H9	blank			
H10	blank			
H11	blank			
H12	blank			

Instrument

Name	Z480	Serial Number	56319

Revision History

Revision	Date	User	Reason

Abs Quant/2nd Derivative Max for All Samples (Abs Quant/2nd Derivative Max)

Settings

Channel	465-510		
Color Compensation	Off		
Program	Program	Units	
Mode	High Confidence		

Subset Name	All Samples

> - Late Cp call (last five cycles) has higher uncertainty

Results

Inc	Pos	Name	Type	CP	Concentration	Standard	Status
☑	A1	GAPDH 1	Unknown	30.93			
☑	A2	GAPDH 1	Unknown	30.22			
☑	A3	GAPDH 1	Unknown	30.27			
☑	A4	GAPDH 2	Unknown	29.75			
☑	A5	GAPDH 2	Unknown	32.17			
☑	A6	GAPDH 2	Unknown	28.85			
☑	A7	GAPDH 3	Unknown	29.11			
☑	A8	GAPDH 3	Unknown	28.48			
☑	A9	GAPDH 3	Unknown	28.88			
☑	A10	GAPDH 4	Unknown	23.82			
☑	A11	GAPDH 4	Unknown	25.92			
☑	A12	GAPDH 4	Unknown	27.54			
☑	B1	FCER1A 1	Unknown	28.52			
☑	B2	FCER1A 1	Unknown	29.22			
☑	B3	FCER1A 1	Unknown	28.43			
☑	B4	FCER1A 2	Unknown	28.34			
☑	B5	FCER1A 2	Unknown	30.22			
☑	B6	FCER1A 2	Unknown	27.88			
☑	B7	FCER1A 3	Unknown	30.55			
☑	B8	FCER1A 3	Unknown	28.68			
☑	B9	FCER1A 3	Unknown	27.77			
☑	B10	FCER1A 4	Unknown	28.78			
☑	B11	FCER1A 4	Unknown				
☑	B12	FCER1A 4	Unknown	26.75			
☑	C1	RGS1 1	Unknown	35.00			>
☑	C2	RGS1 1	Unknown	35.00			>
☑	C3	RGS1 1	Unknown	32.22			
☑	C4	RGS1 2	Unknown	32.96			
☑	C5	RGS1 2	Unknown	35.00			>
☑	C6	RGS1 2	Unknown	35.00			>
☑	C7	RGS1 3	Unknown	33.84			
☑	C8	RGS1 3	Unknown	35.00			>
☑	C9	RGS1 3	Unknown	35.00			>
☑	C10	RGS1 4	Unknown	31.03			
☑	C11	RGS1 4	Unknown	30.57			
☑	C12	RGS1 4	Unknown	31.84			
☑	D1	GAPDH 5	Unknown	29.33			

> - Late Cp call (last five cycles) has higher uncertainty

Results

Inc	Pos	Name	Type	CP	Concentration	Standard	Status
☑	D2	GAPDH 5	Unknown	30.34			
☑	D3	GAPDH 5	Unknown	30.70			
☑	D4	GAPDH 6	Unknown	29.67			
☑	D5	GAPDH 6	Unknown	31.15			
☑	D6	GAPDH 6	Unknown	28.85			
☑	D7	GAPDH 7	Unknown	31.24			
☑	D8	GAPDH 7	Unknown	31.16			
☑	D9	GAPDH 7	Unknown	29.90			
☑	D10	GAPDH 8	Unknown	29.51			
☑	D11	GAPDH 8	Unknown	29.45			
☑	D12	GAPDH 8	Unknown	29.08			
☑	E1	FCER1A 5	Unknown	27.13			
☑	E2	FCER1A 5	Unknown	27.10			
☑	E3	FCER1A 5	Unknown	27.28			
☑	E4	FCER1A 6	Unknown	28.97			
☑	E5	FCER1A 6	Unknown	28.85			
☑	E6	FCER1A 6	Unknown	27.06			
☑	E7	FCER1A 7	Unknown	29.15			
☑	E8	FCER1A 7	Unknown	28.85			
☑	E9	FCER1A 7	Unknown	28.54			
☑	E10	FCER1A 8	Unknown	29.21			
☑	E11	FCER1A 8	Unknown	28.98			
☑	E12	FCER1A 8	Unknown	28.80			
☑	F1	RGS1 5	Unknown	28.89			
☑	F2	RGS1 5	Unknown	29.48			
☑	F3	RGS1 5	Unknown	28.25			
☑	F4	RGS1 6	Unknown	35.00			>
☑	F5	RGS1 6	Unknown	35.00			>
☑	F6	RGS1 6	Unknown	35.00			>
☑	F7	RGS1 7	Unknown	33.10			
☑	F8	RGS1 7	Unknown	32.97			
☑	F9	RGS1 7	Unknown	32.93			
☑	F10	RGS1 8	Unknown	32.61			
☑	F11	RGS1 8	Unknown	32.84			
☑	F12	RGS1 8	Unknown	33.79			
☑	G1	blank	Unknown				
☑	G2	blank	Unknown				

> - Late Cp call (last five cycles) has higher uncertainty

Results

Inc	Pos	Name	Type	CP	Concentration	Standard	Status
☑	G3	blank	Unknown				
☑	G4	blank	Unknown				
☑	G5	blank	Unknown				
☑	G6	blank	Unknown				
☑	G7	blank	Unknown				
☑	G8	blank	Unknown				
☑	G9	blank	Unknown				
☑	G10	blank	Unknown				
☑	G11	blank	Unknown				
☑	G12	blank	Unknown				
☑	H1	blank	Unknown				
☑	H2	blank	Unknown				
☑	H3	blank	Unknown				
☑	H4	blank	Unknown				
☑	H5	blank	Unknown				
☑	H6	blank	Unknown				
☑	H7	blank	Unknown				
☑	H8	blank	Unknown				
☑	H9	blank	Unknown				
☑	H10	blank	Unknown				
☑	H11	blank	Unknown				
☑	H12	blank	Unknown				

> - Late Cp call (last five cycles) has higher uncertainty

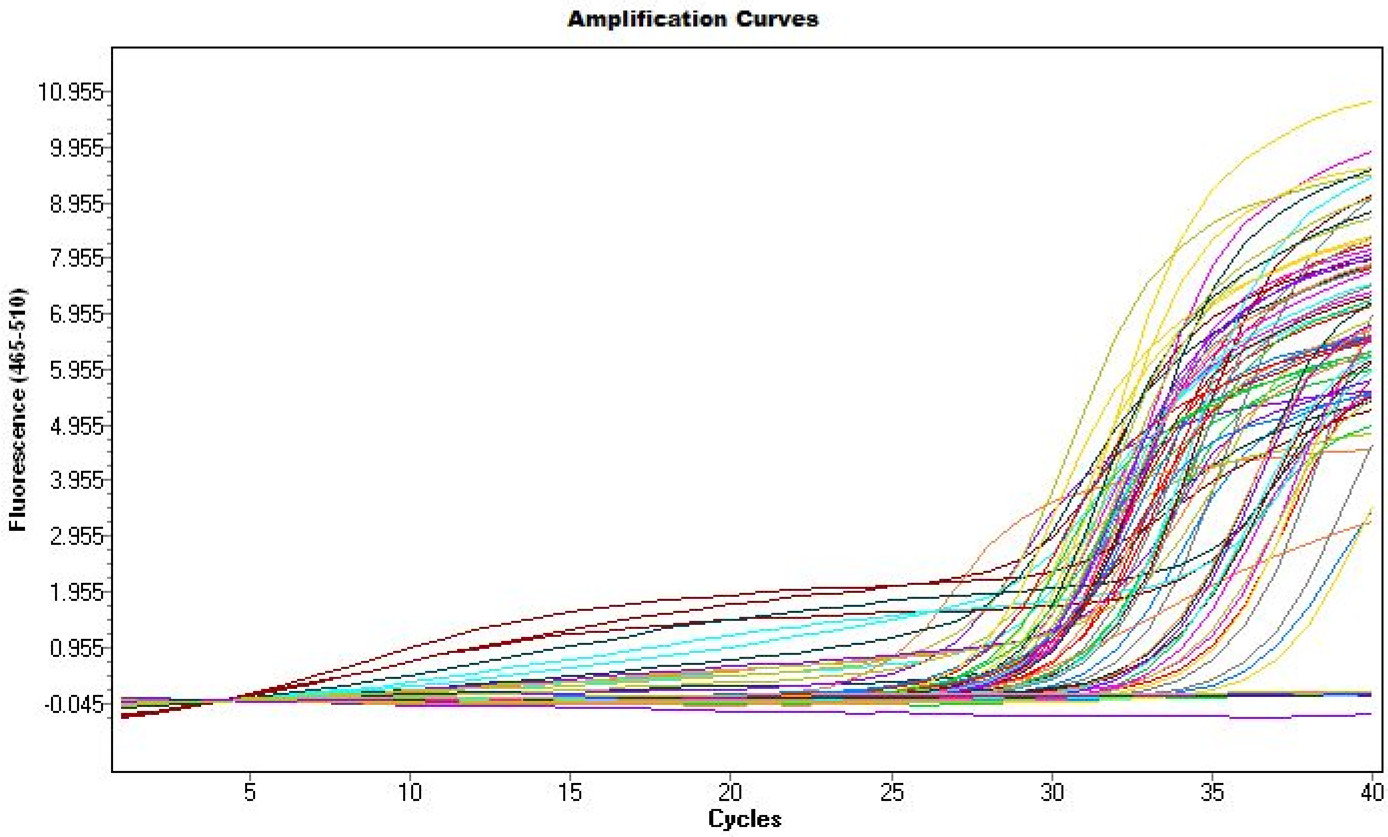

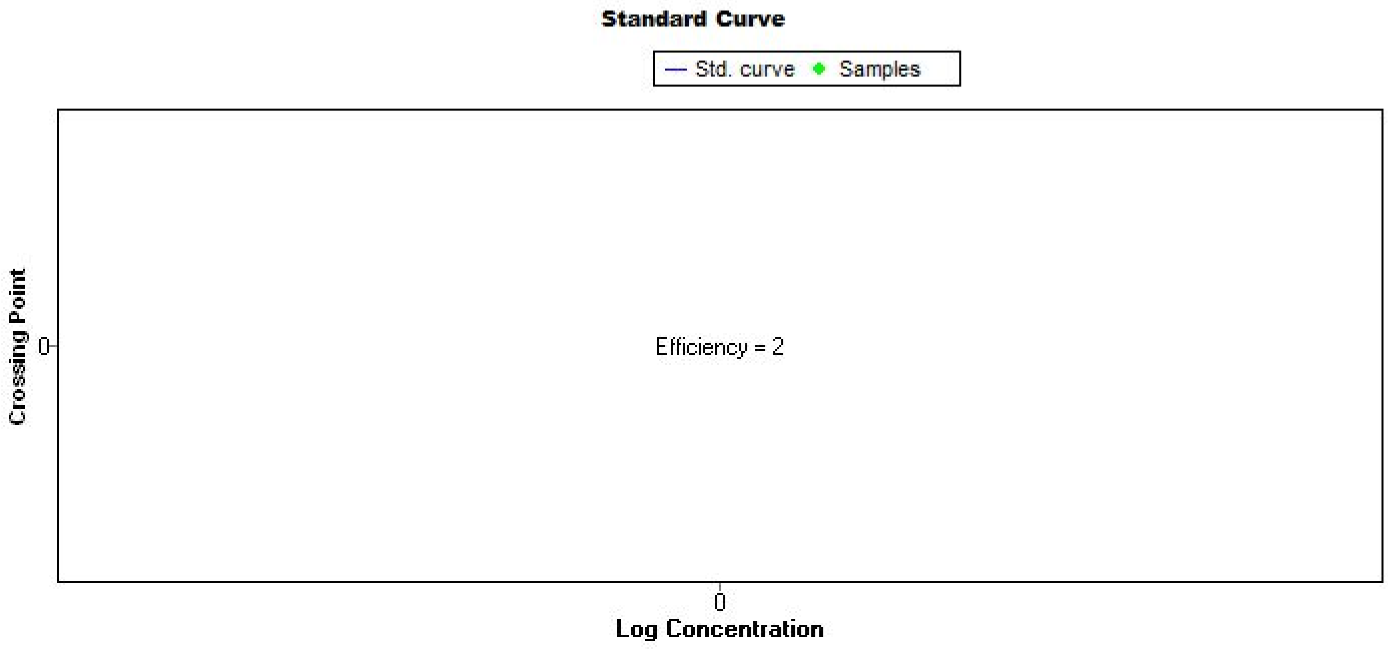

Analysis Notes	

Abs Quant/Fit Points for All Samples (Abs Quant/Fit Points)

Settings

Channel	465-510				
Color Compensation	Off				
Standard Curve	Set Efficiency (2.00)				
Program	Program			Units	
First Cycle	1	Last Cycle	40	Background	2-6
Noiseband Method	Auto	Noiseband	0.8071	STD Dev Multiplier	12.0000
# of Fit Points	2	Threshold Method	Auto	Threshold	0.8071

Subset Name	All Samples

Results

Inc	Pos	Name	Type	CP	Concentration	Standard	Status
☑	A1	GAPDH 1	Unknown	30.70			
☑	A2	GAPDH 1	Unknown	29.35			
☑	A3	GAPDH 1	Unknown	29.91			
☑	A4	GAPDH 2	Unknown	28.40			
☑	A5	GAPDH 2	Unknown	31.23			
☑	A6	GAPDH 2	Unknown	27.77			
☑	A7	GAPDH 3	Unknown	7.36			
☑	A8	GAPDH 3	Unknown	17.73			
☑	A9	GAPDH 3	Unknown	26.89			
☑	A10	GAPDH 4	Unknown	23.88			
☑	A11	GAPDH 4	Unknown	24.44			
☑	A12	GAPDH 4	Unknown	18.64			
☑	B1	FCER1A 1	Unknown	28.35			
☑	B2	FCER1A 1	Unknown	28.65			
☑	B3	FCER1A 1	Unknown	28.34			
☑	B4	FCER1A 2	Unknown	27.82			
☑	B5	FCER1A 2	Unknown	29.72			
☑	B6	FCER1A 2	Unknown	27.33			
☑	B7	FCER1A 3	Unknown	6.80			
☑	B8	FCER1A 3	Unknown	12.03			
☑	B9	FCER1A 3	Unknown	13.73			
☑	B10	FCER1A 4	Unknown	29.08			
☑	B11	FCER1A 4	Unknown				
☑	B12	FCER1A 4	Unknown	22.07			
☑	C1	RGS1 1	Unknown	35.66			
☑	C2	RGS1 1	Unknown	33.46			
☑	C3	RGS1 1	Unknown	32.48			

Results

Inc	Pos	Name	Type	CP	Concentration	Standard	Status
☑	C4	RGS1 2	Unknown	32.74			
☑	C5	RGS1 2	Unknown	34.22			
☑	C6	RGS1 2	Unknown	33.72			
☑	C7	RGS1 3	Unknown	7.55			
☑	C8	RGS1 3	Unknown	10.92			
☑	C9	RGS1 3	Unknown	9.56			
☑	C10	RGS1 4	Unknown	16.49			
☑	C11	RGS1 4	Unknown	14.71			
☑	C12	RGS1 4	Unknown	15.25			
☑	D1	GAPDH 5	Unknown	28.35			
☑	D2	GAPDH 5	Unknown	28.89			
☑	D3	GAPDH 5	Unknown	29.60			
☑	D4	GAPDH 6	Unknown	28.67			
☑	D5	GAPDH 6	Unknown	30.26			
☑	D6	GAPDH 6	Unknown	27.50			
☑	D7	GAPDH 7	Unknown	29.76			
☑	D8	GAPDH 7	Unknown	29.90			
☑	D9	GAPDH 7	Unknown	28.73			
☑	D10	GAPDH 8	Unknown	29.36			
☑	D11	GAPDH 8	Unknown	28.66			
☑	D12	GAPDH 8	Unknown	27.81			
☑	E1	FCER1A 5	Unknown	26.39			
☑	E2	FCER1A 5	Unknown	26.57			
☑	E3	FCER1A 5	Unknown	26.99			
☑	E4	FCER1A 6	Unknown	28.31			
☑	E5	FCER1A 6	Unknown	28.32			
☑	E6	FCER1A 6	Unknown	26.53			
☑	E7	FCER1A 7	Unknown	28.56			
☑	E8	FCER1A 7	Unknown	28.47			
☑	E9	FCER1A 7	Unknown	27.71			
☑	E10	FCER1A 8	Unknown	28.63			
☑	E11	FCER1A 8	Unknown	28.50			
☑	E12	FCER1A 8	Unknown	27.73			
☑	F1	RGS1 5	Unknown	27.68			
☑	F2	RGS1 5	Unknown	27.77			
☑	F3	RGS1 5	Unknown	27.15			
☑	F4	RGS1 6	Unknown	32.87			

Results

Inc	Pos	Name	Type	CP	Concentration	Standard	Status
☑	F5	RGS1 6	Unknown	35.21			
☑	F6	RGS1 6	Unknown	36.17			
☑	F7	RGS1 7	Unknown	31.79			
☑	F8	RGS1 7	Unknown	32.16			
☑	F9	RGS1 7	Unknown	31.59			
☑	F10	RGS1 8	Unknown	31.68			
☑	F11	RGS1 8	Unknown	32.18			
☑	F12	RGS1 8	Unknown	32.97			
☑	G1	blank	Unknown				
☑	G2	blank	Unknown				
☑	G3	blank	Unknown				
☑	G4	blank	Unknown				
☑	G5	blank	Unknown				
☑	G6	blank	Unknown				
☑	G7	blank	Unknown				
☑	G8	blank	Unknown				
☑	G9	blank	Unknown				
☑	G10	blank	Unknown				
☑	G11	blank	Unknown				
☑	G12	blank	Unknown				
☑	H1	blank	Unknown				
☑	H2	blank	Unknown				
☑	H3	blank	Unknown				
☑	H4	blank	Unknown				
☑	H5	blank	Unknown				
☑	H6	blank	Unknown				
☑	H7	blank	Unknown				
☑	H8	blank	Unknown				
☑	H9	blank	Unknown				
☑	H10	blank	Unknown				
☑	H11	blank	Unknown				
☑	H12	blank	Unknown				

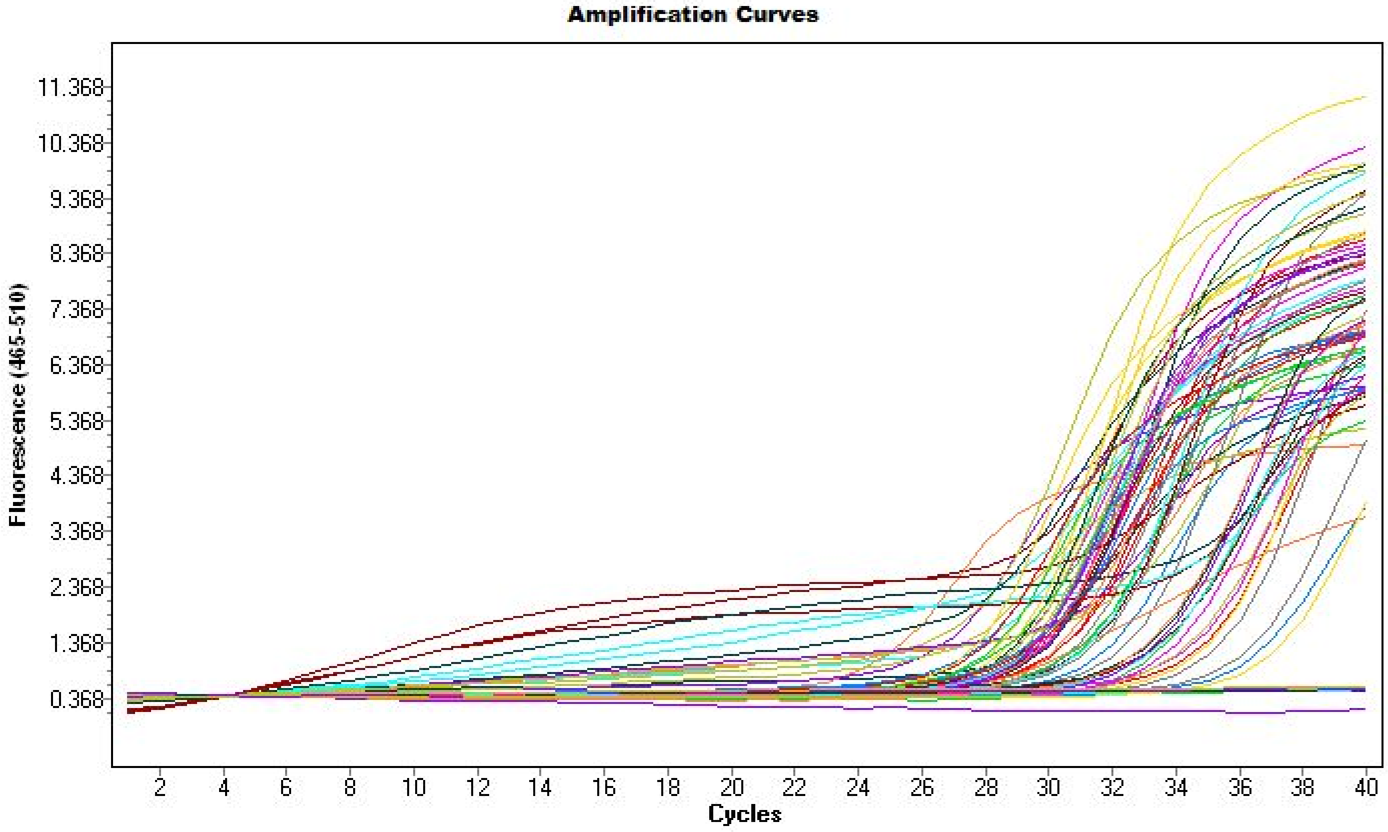
Amplification Curves
11.368
10.368
9.368
8.368
7.368
6.368
5.368
4.368
3.368
2.368
1.368
0.368
Fluorescence (465-510)
2 4 6 8 10 12 14 16 18 20 22 24 26 28 30 32 34 36 38 40
Cycles

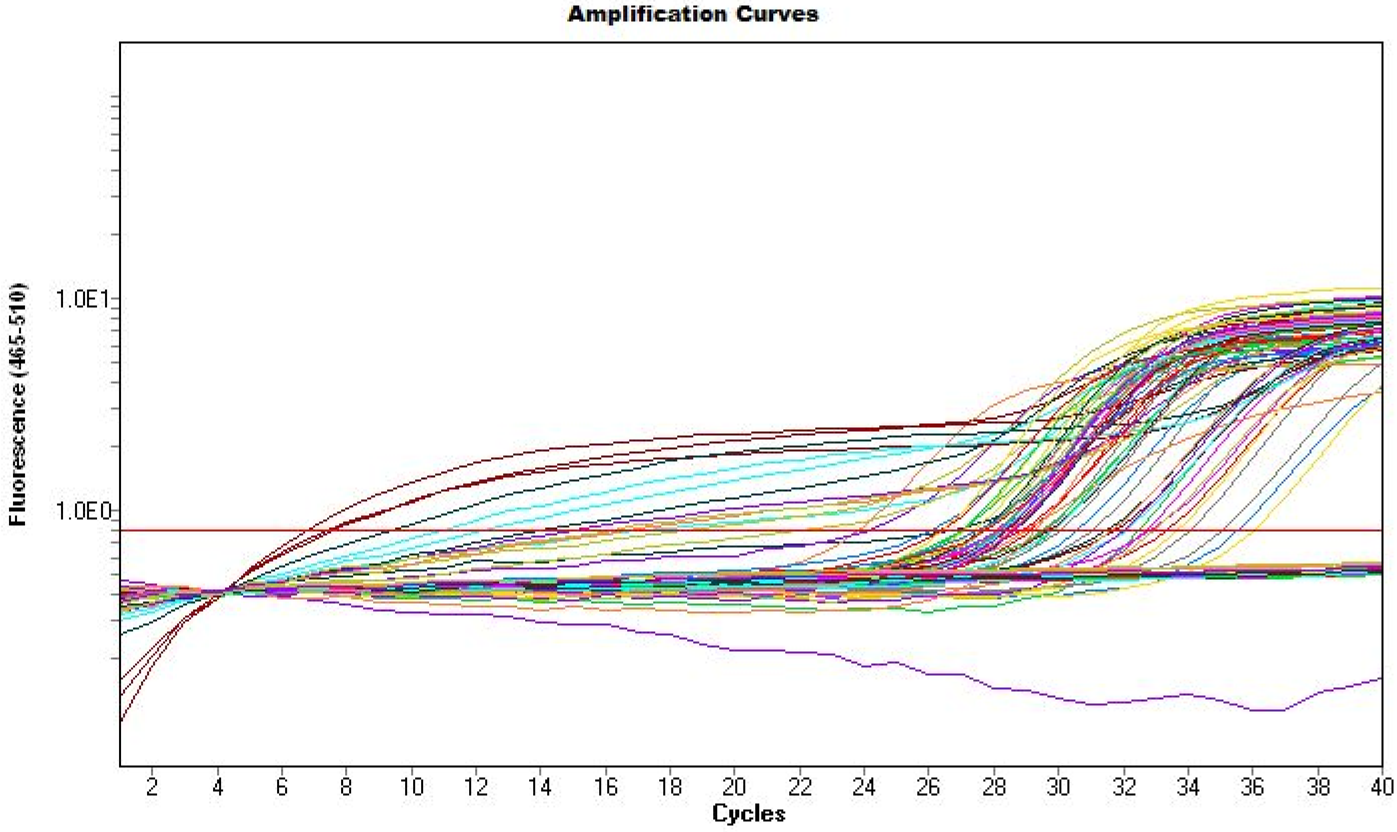
Amplification Curves
1.0E1
1.0E0
Fluorescence (465-510)
2 4 6 8 10 12 14 16 18 20 22 24 26 28 30 32 34 36 38 40
Cycles

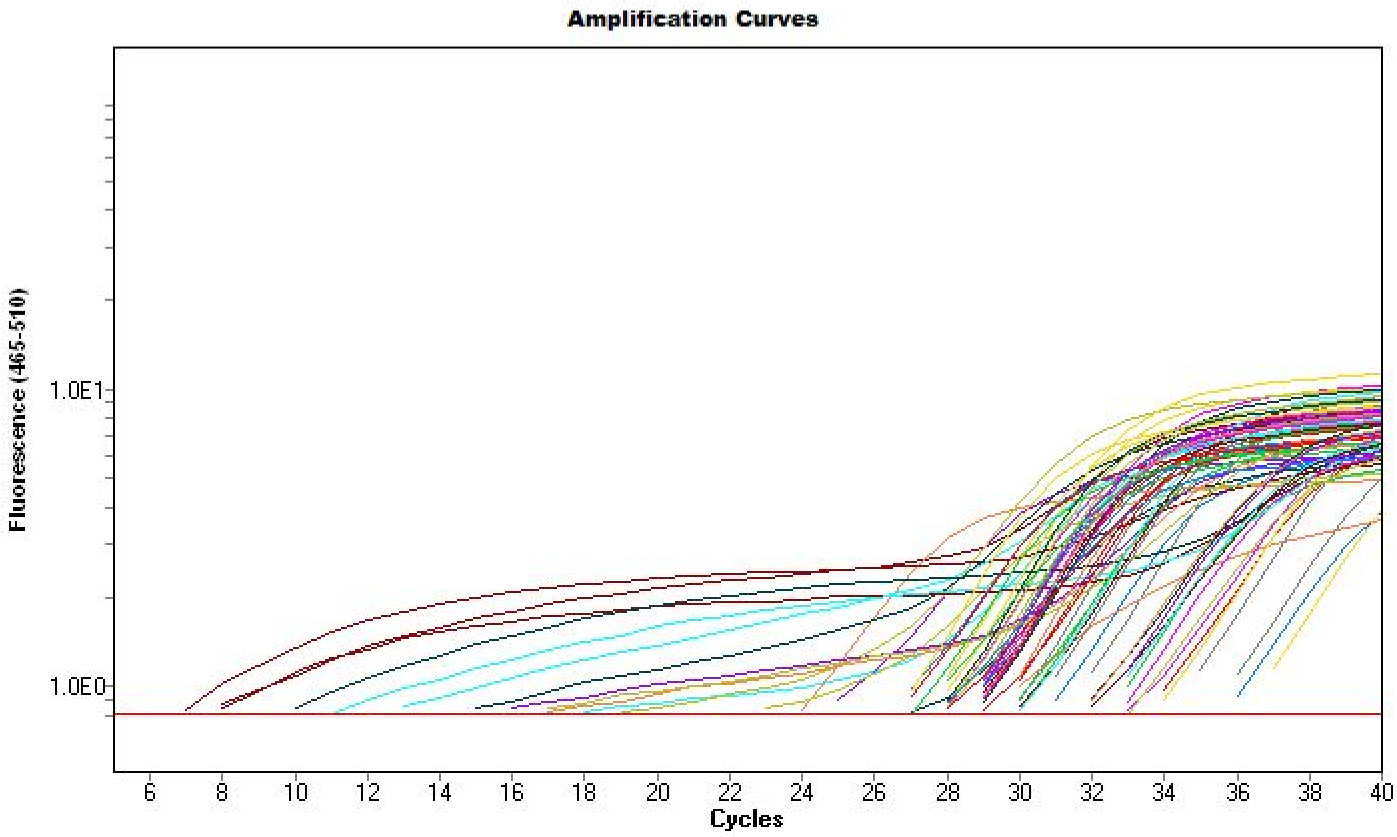

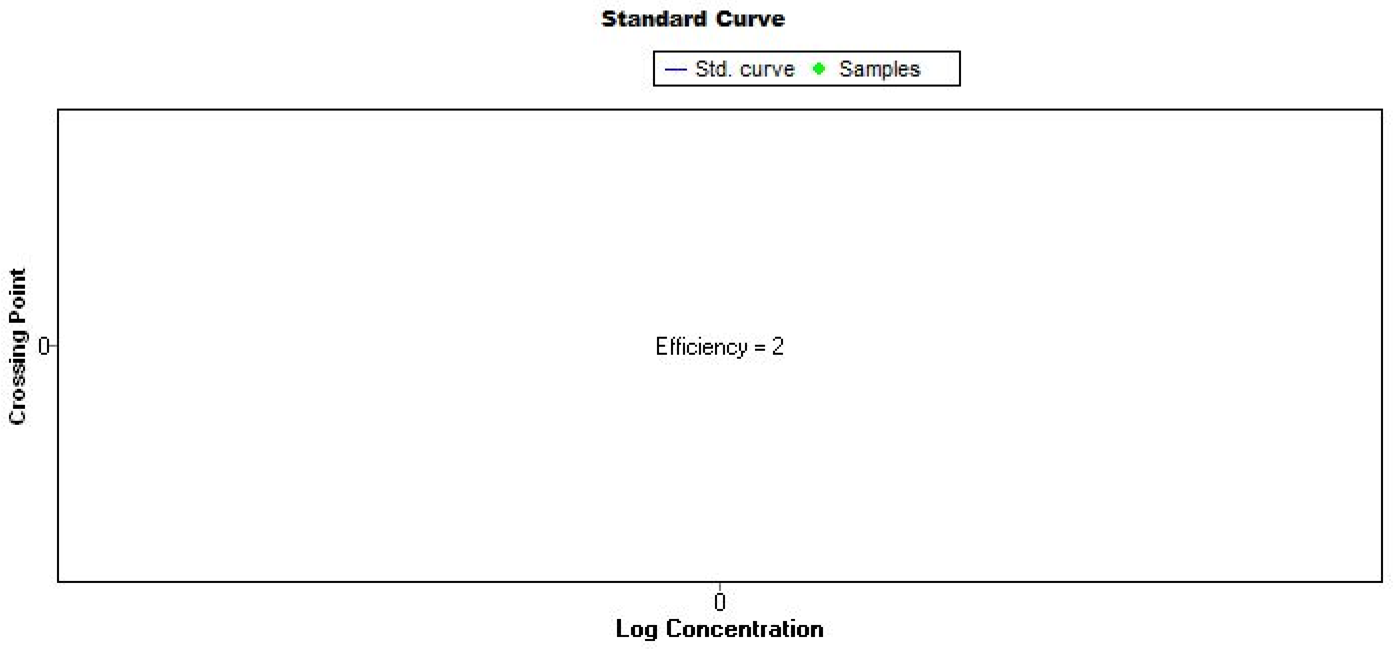

Analysis Notes	

Gene Scanning for All Samples (Gene Scanning)

Channel	465-510	Color Compensation	Off
Progam Name	Program		
Stds Settings	Auto-Group		
Sensitivity	0.30	Threshold (TmShift)	5.000
Pre-melt	70.72-71.71	Post-melt Temp	88.57-89.56

Subset Name	All Samples

Results

Inc	Pos.	Sample Name	Sample Type	Group	Status
☑	A1	GAPDH 1	Unknown	6	
☑	A2	GAPDH 1	Unknown	Negative	
☑	A3	GAPDH 1	Unknown	1	
☑	A4	GAPDH 2	Unknown	Unknown	
☑	A5	GAPDH 2	Unknown	Unknown	
☑	A6	GAPDH 2	Unknown	6	
☑	A7	GAPDH 3	Unknown	Unknown	
☑	A8	GAPDH 3	Unknown	Unknown	
☑	A9	GAPDH 3	Unknown	1	
☑	A10	GAPDH 4	Unknown	3	
☑	A11	GAPDH 4	Unknown	3	
☑	A12	GAPDH 4	Unknown	Unknown	
☑	B1	FCER1A 1	Unknown	1	
☑	B2	FCER1A 1	Unknown	1	
☑	B3	FCER1A 1	Unknown	Unknown	
☑	B4	FCER1A 2	Unknown	1	
☑	B5	FCER1A 2	Unknown	Unknown	
☑	B6	FCER1A 2	Unknown	1	
☑	B7	FCER1A 3	Unknown	1	
☑	B8	FCER1A 3	Unknown	1	
☑	B9	FCER1A 3	Unknown	1	
☑	B10	FCER1A 4	Unknown	Unknown	
☑	B11	FCER1A 4	Unknown	Negative	
☑	B12	FCER1A 4	Unknown	Unknown	
☑	C1	RGS1 1	Unknown	Negative	
☑	C2	RGS1 1	Unknown	Unknown	
☑	C3	RGS1 1	Unknown	Unknown	
☑	C4	RGS1 2	Unknown	Unknown	
☑	C5	RGS1 2	Unknown	Unknown	
☑	C6	RGS1 2	Unknown	Unknown	
☑	C7	RGS1 3	Unknown	2	

Results

Inc	Pos.	Sample Name	Sample Type	Group	Status
☑	C8	RGS1 3	Unknown	2	
☑	C9	RGS1 3	Unknown	4	
☑	C10	RGS1 4	Unknown	2	
☑	C11	RGS1 4	Unknown	Unknown	
☑	C12	RGS1 4	Unknown	2	
☑	D1	GAPDH 5	Unknown	3	
☑	D2	GAPDH 5	Unknown	Unknown	
☑	D3	GAPDH 5	Unknown	Unknown	
☑	D4	GAPDH 6	Unknown	Unknown	
☑	D5	GAPDH 6	Unknown	Unknown	
☑	D6	GAPDH 6	Unknown	Unknown	
☑	D7	GAPDH 7	Unknown	Unknown	
☑	D8	GAPDH 7	Unknown	Unknown	
☑	D9	GAPDH 7	Unknown	Unknown	
☑	D10	GAPDH 8	Unknown	Unknown	
☑	D11	GAPDH 8	Unknown	Unknown	
☑	D12	GAPDH 8	Unknown	3	
☑	E1	FCER1A 5	Unknown	Unknown	
☑	E2	FCER1A 5	Unknown	Unknown	
☑	E3	FCER1A 5	Unknown	Unknown	
☑	E4	FCER1A 6	Unknown	5	
☑	E5	FCER1A 6	Unknown	Unknown	
☑	E6	FCER1A 6	Unknown	1	
☑	E7	FCER1A 7	Unknown	5	
☑	E8	FCER1A 7	Unknown	Unknown	
☑	E9	FCER1A 7	Unknown	1	
☑	E10	FCER1A 8	Unknown	5	
☑	E11	FCER1A 8	Unknown	Unknown	
☑	E12	FCER1A 8	Unknown	6	
☑	F1	RGS1 5	Unknown	2	
☑	F2	RGS1 5	Unknown	2	
☑	F3	RGS1 5	Unknown	2	
☑	F4	RGS1 6	Unknown	Unknown	
☑	F5	RGS1 6	Unknown	Negative	
☑	F6	RGS1 6	Unknown	Unknown	
☑	F7	RGS1 7	Unknown	4	
☑	F8	RGS1 7	Unknown	Unknown	
☑	F9	RGS1 7	Unknown	Unknown	

Results

Inc	Pos.	Sample Name	Sample Type	Group	Status
☑	F10	RGS1 8	Unknown	2	
☑	F11	RGS1 8	Unknown	2	
☑	F12	RGS1 8	Unknown	4	
☑	G1	blank	Unknown	Negative	
☑	G2	blank	Unknown	Negative	
☑	G3	blank	Unknown	Negative	
☑	G4	blank	Unknown	Negative	
☑	G5	blank	Unknown	Negative	
☑	G6	blank	Unknown	Negative	
☑	G7	blank	Unknown	Negative	
☑	G8	blank	Unknown	Negative	
☑	G9	blank	Unknown	Negative	
☑	G10	blank	Unknown	Negative	
☑	G11	blank	Unknown	Negative	
☑	G12	blank	Unknown	Negative	
☑	H1	blank	Unknown	Negative	
☑	H2	blank	Unknown	Negative	
☑	H3	blank	Unknown	Negative	
☑	H4	blank	Unknown	Negative	
☑	H5	blank	Unknown	Negative	
☑	H6	blank	Unknown	Negative	
☑	H7	blank	Unknown	Negative	
☑	H8	blank	Unknown	Negative	
☑	H9	blank	Unknown	Negative	
☑	H10	blank	Unknown	Negative	
☑	H11	blank	Unknown	Negative	
☑	H12	blank	Unknown	Negative	

**Group**	1

Inc	Pos	Sample Name	Sample Type
☑	A3	GAPDH 1	Unknown
☑	A9	GAPDH 3	Unknown
☑	B1	FCER1A 1	Unknown
☑	B2	FCER1A 1	Unknown
☑	B4	FCER1A 2	Unknown
☑	B6	FCER1A 2	Unknown
☑	B7	FCER1A 3	Unknown
☑	B8	FCER1A 3	Unknown
☑	B9	FCER1A 3	Unknown

Group	1

Inc	Pos	Sample Name	Sample Type
☑	E6	FCER1A 6	Unknown
☑	E9	FCER1A 7	Unknown

Group	2

Inc	Pos	Sample Name	Sample Type
☑	C7	RGS1 3	Unknown
☑	C8	RGS1 3	Unknown
☑	C10	RGS1 4	Unknown
☑	C12	RGS1 4	Unknown
☑	F1	RGS1 5	Unknown
☑	F2	RGS1 5	Unknown
☑	F3	RGS1 5	Unknown
☑	F10	RGS1 8	Unknown
☑	F11	RGS1 8	Unknown

Group	3

Inc	Pos	Sample Name	Sample Type
☑	A10	GAPDH 4	Unknown
☑	A11	GAPDH 4	Unknown
☑	D1	GAPDH 5	Unknown
☑	D12	GAPDH 8	Unknown

Group	4

Inc	Pos	Sample Name	Sample Type
☑	C9	RGS1 3	Unknown
☑	F7	RGS1 7	Unknown
☑	F12	RGS1 8	Unknown

Group	5

Inc	Pos	Sample Name	Sample Type
☑	E4	FCER1A 6	Unknown
☑	E7	FCER1A 7	Unknown
☑	E10	FCER1A 8	Unknown

Group	6

Inc	Pos	Sample Name	Sample Type
☑	A1	GAPDH 1	Unknown
☑	A6	GAPDH 2	Unknown
☑	E12	FCER1A 8	Unknown

Group	Negative

Inc	Pos	Sample Name	Sample Type
☑	A2	GAPDH 1	Unknown
☑	B11	FCER1A 4	Unknown
☑	C1	RGS1 1	Unknown
☑	F5	RGS1 6	Unknown
☑	G1	blank	Unknown
☑	G2	blank	Unknown
☑	G3	blank	Unknown
☑	G4	blank	Unknown
☑	G5	blank	Unknown
☑	G6	blank	Unknown
☑	G7	blank	Unknown
☑	G8	blank	Unknown
☑	G9	blank	Unknown
☑	G10	blank	Unknown
☑	G11	blank	Unknown
☑	G12	blank	Unknown
☑	H1	blank	Unknown
☑	H2	blank	Unknown
☑	H3	blank	Unknown
☑	H4	blank	Unknown
☑	H5	blank	Unknown
☑	H6	blank	Unknown
☑	H7	blank	Unknown
☑	H8	blank	Unknown
☑	H9	blank	Unknown
☑	H10	blank	Unknown
☑	H11	blank	Unknown
☑	H12	blank	Unknown

Group	Unknown

Inc	Pos	Sample Name	Sample Type
☑	A4	GAPDH 2	Unknown
☑	A5	GAPDH 2	Unknown
☑	A7	GAPDH 3	Unknown
☑	A8	GAPDH 3	Unknown
☑	A12	GAPDH 4	Unknown
☑	B3	FCER1A 1	Unknown
☑	B5	FCER1A 2	Unknown
☑	B10	FCER1A 4	Unknown

**Group**	Unknown

Inc	Pos	Sample Name	Sample Type
☑	B12	FCER1A 4	Unknown
☑	C2	RGS1 1	Unknown
☑	C3	RGS1 1	Unknown
☑	C4	RGS1 2	Unknown
☑	C5	RGS1 2	Unknown
☑	C6	RGS1 2	Unknown
☑	C11	RGS1 4	Unknown
☑	D2	GAPDH 5	Unknown
☑	D3	GAPDH 5	Unknown
☑	D4	GAPDH 6	Unknown
☑	D5	GAPDH 6	Unknown
☑	D6	GAPDH 6	Unknown
☑	D7	GAPDH 7	Unknown
☑	D8	GAPDH 7	Unknown
☑	D9	GAPDH 7	Unknown
☑	D10	GAPDH 8	Unknown
☑	D11	GAPDH 8	Unknown
☑	E1	FCER1A 5	Unknown
☑	E2	FCER1A 5	Unknown
☑	E3	FCER1A 5	Unknown
☑	E5	FCER1A 6	Unknown
☑	E8	FCER1A 7	Unknown
☑	E11	FCER1A 8	Unknown
☑	F4	RGS1 6	Unknown
☑	F6	RGS1 6	Unknown
☑	F8	RGS1 7	Unknown
☑	F9	RGS1 7	Unknown

Melting Curves

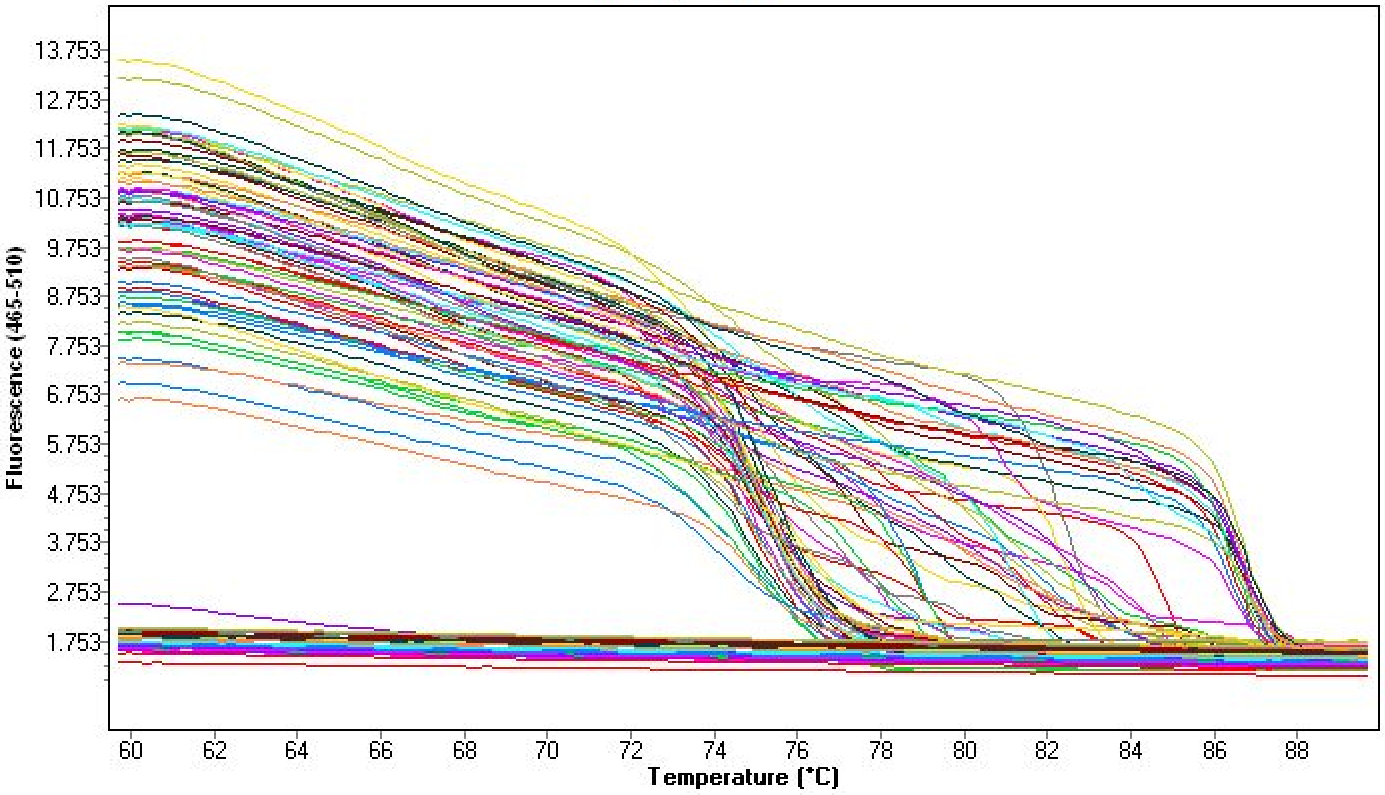

Normalized Melting Curves

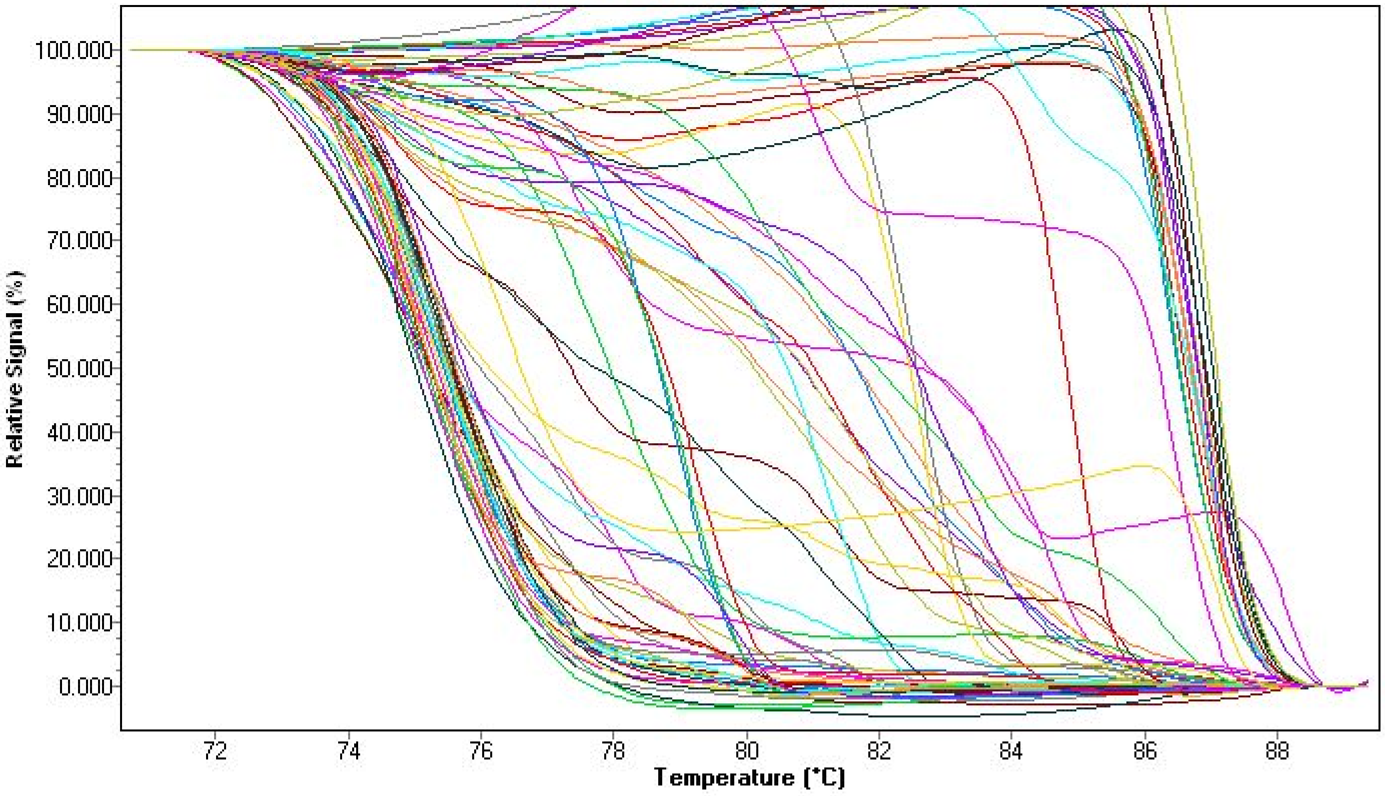

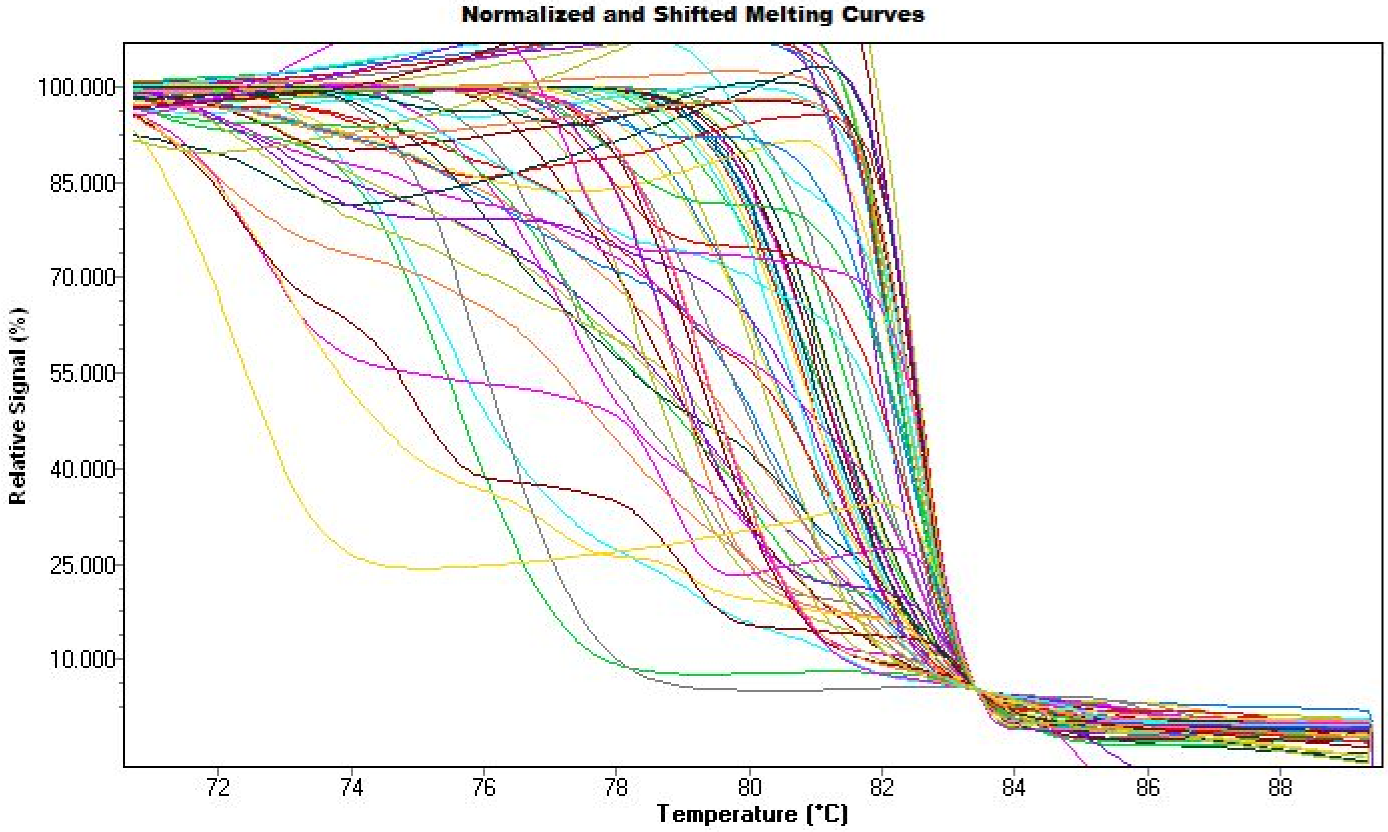

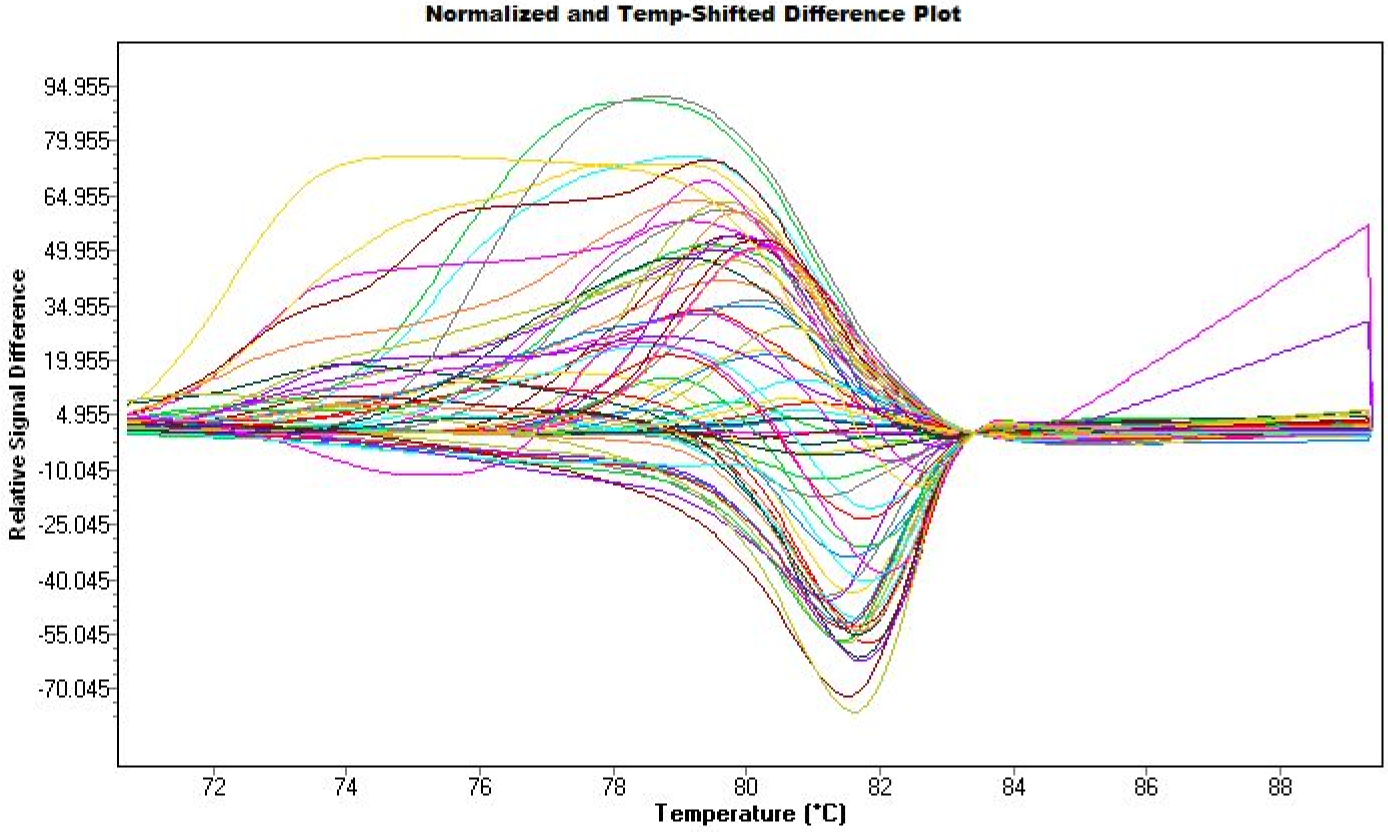

Analysis Notes	

Melt Curve Genotyping for All Samples (Melt Curve Genotyping)

Channel	465-510	Color Compensation	Off				
Progam Name	Program						
Stds Settings	Auto-Group						
Sensitivity	Normal	Temp Range	62-86	Score	0.7	Res	0.1

Subset Name	All Samples

Results

Inc	Pos.	Sample Name	Sample Type	Group	Score	Resolution	Status
☑	A1	GAPDH 1	Unknown	1	0.95	0.79	
☑	A2	GAPDH 1	Unknown	Unknown	0.65	0.18	
☑	A3	GAPDH 1	Unknown	1	0.95	0.80	
☑	A4	GAPDH 2	Unknown	1	0.86	0.53	
☑	A5	GAPDH 2	Unknown	1	0.89	0.40	
☑	A6	GAPDH 2	Unknown	1	0.96	0.65	
☑	A7	GAPDH 3	Unknown	1	0.93	0.72	
☑	A8	GAPDH 3	Unknown	1	0.90	0.59	
☑	A9	GAPDH 3	Unknown	1	0.97	0.83	
☑	A10	GAPDH 4	Unknown	3	0.97	0.28	
☑	A11	GAPDH 4	Unknown	3	1.00	0.40	
☑	A12	GAPDH 4	Unknown	3	0.98	0.50	
☑	B1	FCER1A 1	Unknown	1	0.96	0.71	
☑	B2	FCER1A 1	Unknown	1	0.97	0.78	
☑	B3	FCER1A 1	Unknown	1	0.95	0.77	
☑	B4	FCER1A 2	Unknown	1	0.91	0.73	
☑	B5	FCER1A 2	Unknown	1	0.94	0.71	
☑	B6	FCER1A 2	Unknown	1	0.96	0.73	
☑	B7	FCER1A 3	Unknown	1	0.97	0.66	
☑	B8	FCER1A 3	Unknown	1	0.97	0.69	
☑	B9	FCER1A 3	Unknown	1	0.97	0.63	
☑	B10	FCER1A 4	Unknown	1	0.95	0.72	
☑	B11	FCER1A 4	Unknown	Negative	1.00	1.00	
☑	B12	FCER1A 4	Unknown	1	1.00	0.71	
☑	C1	RGS1 1	Unknown	1	0.89	0.60	
☑	C2	RGS1 1	Unknown	2	0.78	0.19	
☑	C3	RGS1 1	Unknown	4	0.92	0.13	
☑	C4	RGS1 2	Unknown	3	0.84	0.22	
☑	C5	RGS1 2	Unknown	5	1.00	0.51	
☑	C6	RGS1 2	Unknown	Unknown	0.75	0.03	
☑	C7	RGS1 3	Unknown	2	0.95	0.37	

Results

Inc	Pos.	Sample Name	Sample Type	Group	Score	Resolution	Status
☑	C8	RGS1 3	Unknown	2	0.98	0.36	
☑	C9	RGS1 3	Unknown	2	0.91	0.41	
☑	C10	RGS1 4	Unknown	2	0.97	0.32	
☑	C11	RGS1 4	Unknown	2	0.99	0.39	
☑	C12	RGS1 4	Unknown	2	1.00	0.45	
☑	D1	GAPDH 5	Unknown	3	0.96	0.36	
☑	D2	GAPDH 5	Unknown	3	0.94	0.35	
☑	D3	GAPDH 5	Unknown	3	0.89	0.16	
☑	D4	GAPDH 6	Unknown	3	0.94	0.25	
☑	D5	GAPDH 6	Unknown	1	0.97	0.56	
☑	D6	GAPDH 6	Unknown	1	0.80	0.40	
☑	D7	GAPDH 7	Unknown	Unknown	0.62		
☑	D8	GAPDH 7	Unknown	3	0.89	0.32	
☑	D9	GAPDH 7	Unknown	3	0.75	0.16	
☑	D10	GAPDH 8	Unknown	3	0.93	0.37	
☑	D11	GAPDH 8	Unknown	3	0.85	0.11	
☑	D12	GAPDH 8	Unknown	3	0.93	0.36	
☑	E1	FCER1A 5	Unknown	4	1.00	0.53	
☑	E2	FCER1A 5	Unknown	Unknown	0.73	0.03	
☑	E3	FCER1A 5	Unknown	4	0.90	0.29	
☑	E4	FCER1A 6	Unknown	1	0.98	0.74	
☑	E5	FCER1A 6	Unknown	1	0.97	0.71	
☑	E6	FCER1A 6	Unknown	1	0.97	0.67	
☑	E7	FCER1A 7	Unknown	1	0.98	0.67	
☑	E8	FCER1A 7	Unknown	1	0.98	0.72	
☑	E9	FCER1A 7	Unknown	1	0.96	0.64	
☑	E10	FCER1A 8	Unknown	1	0.98	0.66	
☑	E11	FCER1A 8	Unknown	1	0.96	0.59	
☑	E12	FCER1A 8	Unknown	1	0.98	0.79	
☑	F1	RGS1 5	Unknown	2	0.97	0.44	
☑	F2	RGS1 5	Unknown	2	0.98	0.42	
☑	F3	RGS1 5	Unknown	2	0.96	0.49	
☑	F4	RGS1 6	Unknown	6	1.00	0.40	
☑	F5	RGS1 6	Unknown	Unknown	0.67	0.26	
☑	F6	RGS1 6	Unknown	1	0.87	0.42	
☑	F7	RGS1 7	Unknown	2	0.97	0.52	
☑	F8	RGS1 7	Unknown	2	0.89	0.23	
☑	F9	RGS1 7	Unknown	2	0.95	0.29	

Results

Inc	Pos.	Sample Name	Sample Type	Group	Score	Resolution	Status
☑	F10	RGS1 8	Unknown	2	0.98	0.43	
☑	F11	RGS1 8	Unknown	2	0.97	0.44	
☑	F12	RGS1 8	Unknown	2	0.93	0.53	
☑	G1	blank	Unknown	Negative	1.00	1.00	
☑	G2	blank	Unknown	Negative	1.00	1.00	
☑	G3	blank	Unknown	Negative	1.00	1.00	
☑	G4	blank	Unknown	Negative	1.00	1.00	
☑	G5	blank	Unknown	Negative	1.00	1.00	
☑	G6	blank	Unknown	Negative	1.00	1.00	
☑	G7	blank	Unknown	Negative	1.00	1.00	
☑	G8	blank	Unknown	Negative	1.00	1.00	
☑	G9	blank	Unknown	Negative	1.00	1.00	
☑	G10	blank	Unknown	Negative	1.00	1.00	
☑	G11	blank	Unknown	Negative	1.00	1.00	
☑	G12	blank	Unknown	Negative	1.00	1.00	
☑	H1	blank	Unknown	Negative	1.00	1.00	
☑	H2	blank	Unknown	Negative	1.00	1.00	
☑	H3	blank	Unknown	Negative	1.00	1.00	
☑	H4	blank	Unknown	Negative	1.00	1.00	
☑	H5	blank	Unknown	Negative	1.00	1.00	
☑	H6	blank	Unknown	Negative	1.00	1.00	
☑	H7	blank	Unknown	Negative	1.00	1.00	
☑	H8	blank	Unknown	Negative	1.00	1.00	
☑	H9	blank	Unknown	Negative	1.00	1.00	
☑	H10	blank	Unknown	Negative	1.00	1.00	
☑	H11	blank	Unknown	Negative	1.00	1.00	
☑	H12	blank	Unknown	Negative	1.00	1.00	

**Group**	1

Inc	Pos	Sample Name	Sample Type	Score	Resolution	Status
☑	A1	GAPDH 1	Unknown	0.95	0.79	
☑	A3	GAPDH 1	Unknown	0.95	0.80	
☑	A4	GAPDH 2	Unknown	0.86	0.53	
☑	A5	GAPDH 2	Unknown	0.89	0.40	
☑	A6	GAPDH 2	Unknown	0.96	0.65	
☑	A7	GAPDH 3	Unknown	0.93	0.72	
☑	A8	GAPDH 3	Unknown	0.90	0.59	
☑	A9	GAPDH 3	Unknown	0.97	0.83	

Group	1

Inc	Pos	Sample Name	Sample Type	Score	Resolution	Status
☑	B1	FCER1A 1	Unknown	0.96	0.71	
☑	B2	FCER1A 1	Unknown	0.97	0.78	
☑	B3	FCER1A 1	Unknown	0.95	0.77	
☑	B4	FCER1A 2	Unknown	0.91	0.73	
☑	B5	FCER1A 2	Unknown	0.94	0.71	
☑	B6	FCER1A 2	Unknown	0.96	0.73	
☑	B7	FCER1A 3	Unknown	0.97	0.66	
☑	B8	FCER1A 3	Unknown	0.97	0.69	
☑	B9	FCER1A 3	Unknown	0.97	0.63	
☑	B10	FCER1A 4	Unknown	0.95	0.72	
☑	B12	FCER1A 4	Unknown	1.00	0.71	
☑	C1	RGS1 1	Unknown	0.89	0.60	
☑	D5	GAPDH 6	Unknown	0.97	0.56	
☑	D6	GAPDH 6	Unknown	0.80	0.40	
☑	E4	FCER1A 6	Unknown	0.98	0.74	
☑	E5	FCER1A 6	Unknown	0.97	0.71	
☑	E6	FCER1A 6	Unknown	0.97	0.67	
☑	E7	FCER1A 7	Unknown	0.98	0.67	
☑	E8	FCER1A 7	Unknown	0.98	0.72	
☑	E9	FCER1A 7	Unknown	0.96	0.64	
☑	E10	FCER1A 8	Unknown	0.98	0.66	
☑	E11	FCER1A 8	Unknown	0.96	0.59	
☑	E12	FCER1A 8	Unknown	0.98	0.79	
☑	F6	RGS1 6	Unknown	0.87	0.42	

Group	2

Inc	Pos	Sample Name	Sample Type	Score	Resolution	Status
☑	C2	RGS1 1	Unknown	0.78	0.19	
☑	C7	RGS1 3	Unknown	0.95	0.37	
☑	C8	RGS1 3	Unknown	0.98	0.36	
☑	C9	RGS1 3	Unknown	0.91	0.41	
☑	C10	RGS1 4	Unknown	0.97	0.32	
☑	C11	RGS1 4	Unknown	0.99	0.39	
☑	C12	RGS1 4	Unknown	1.00	0.45	
☑	F1	RGS1 5	Unknown	0.97	0.44	
☑	F2	RGS1 5	Unknown	0.98	0.42	
☑	F3	RGS1 5	Unknown	0.96	0.49	
☑	F7	RGS1 7	Unknown	0.97	0.52	

Group	2

Inc	Pos	Sample Name	Sample Type	Score	Resolution	Status
☑	F8	RGS1 7	Unknown	0.89	0.23	
☑	F9	RGS1 7	Unknown	0.95	0.29	
☑	F10	RGS1 8	Unknown	0.98	0.43	
☑	F11	RGS1 8	Unknown	0.97	0.44	
☑	F12	RGS1 8	Unknown	0.93	0.53	

Group	3

Inc	Pos	Sample Name	Sample Type	Score	Resolution	Status
☑	A10	GAPDH 4	Unknown	0.97	0.28	
☑	A11	GAPDH 4	Unknown	1.00	0.40	
☑	A12	GAPDH 4	Unknown	0.98	0.50	
☑	C4	RGS1 2	Unknown	0.84	0.22	
☑	D1	GAPDH 5	Unknown	0.96	0.36	
☑	D2	GAPDH 5	Unknown	0.94	0.35	
☑	D3	GAPDH 5	Unknown	0.89	0.16	
☑	D4	GAPDH 6	Unknown	0.94	0.25	
☑	D8	GAPDH 7	Unknown	0.89	0.32	
☑	D9	GAPDH 7	Unknown	0.75	0.16	
☑	D10	GAPDH 8	Unknown	0.93	0.37	
☑	D11	GAPDH 8	Unknown	0.85	0.11	
☑	D12	GAPDH 8	Unknown	0.93	0.36	

Group	4

Inc	Pos	Sample Name	Sample Type	Score	Resolution	Status
☑	C3	RGS1 1	Unknown	0.92	0.13	
☑	E1	FCER1A 5	Unknown	1.00	0.53	
☑	E3	FCER1A 5	Unknown	0.90	0.29	

Group	5

Inc	Pos	Sample Name	Sample Type	Score	Resolution	Status
☑	C5	RGS1 2	Unknown	1.00	0.51	

Group	6

Inc	Pos	Sample Name	Sample Type	Score	Resolution	Status
☑	F4	RGS1 6	Unknown	1.00	0.40	

Group	Negative

Inc	Pos	Sample Name	Sample Type	Score	Resolution	Status
☑	B11	FCER1A 4	Unknown	1.00	1.00	
☑	G1	blank	Unknown	1.00	1.00	
☑	G2	blank	Unknown	1.00	1.00	
☑	G3	blank	Unknown	1.00	1.00	
☑	G4	blank	Unknown	1.00	1.00	
☑	G5	blank	Unknown	1.00	1.00	
☑	G6	blank	Unknown	1.00	1.00	
☑	G7	blank	Unknown	1.00	1.00	
☑	G8	blank	Unknown	1.00	1.00	
☑	G9	blank	Unknown	1.00	1.00	
☑	G10	blank	Unknown	1.00	1.00	
☑	G11	blank	Unknown	1.00	1.00	
☑	G12	blank	Unknown	1.00	1.00	
☑	H1	blank	Unknown	1.00	1.00	
☑	H2	blank	Unknown	1.00	1.00	
☑	H3	blank	Unknown	1.00	1.00	
☑	H4	blank	Unknown	1.00	1.00	
☑	H5	blank	Unknown	1.00	1.00	
☑	H6	blank	Unknown	1.00	1.00	
☑	H7	blank	Unknown	1.00	1.00	
☑	H8	blank	Unknown	1.00	1.00	
☑	H9	blank	Unknown	1.00	1.00	
☑	H10	blank	Unknown	1.00	1.00	
☑	H11	blank	Unknown	1.00	1.00	
☑	H12	blank	Unknown	1.00	1.00	

Group	Unknown

Inc	Pos	Sample Name	Sample Type	Score	Resolution	Status
☑	A2	GAPDH 1	Unknown	0.65	0.18	
☑	C6	RGS1 2	Unknown	0.75	0.03	
☑	D7	GAPDH 7	Unknown	0.62		
☑	E2	FCER1A 5	Unknown	0.73	0.03	
☑	F5	RGS1 6	Unknown	0.67	0.26	

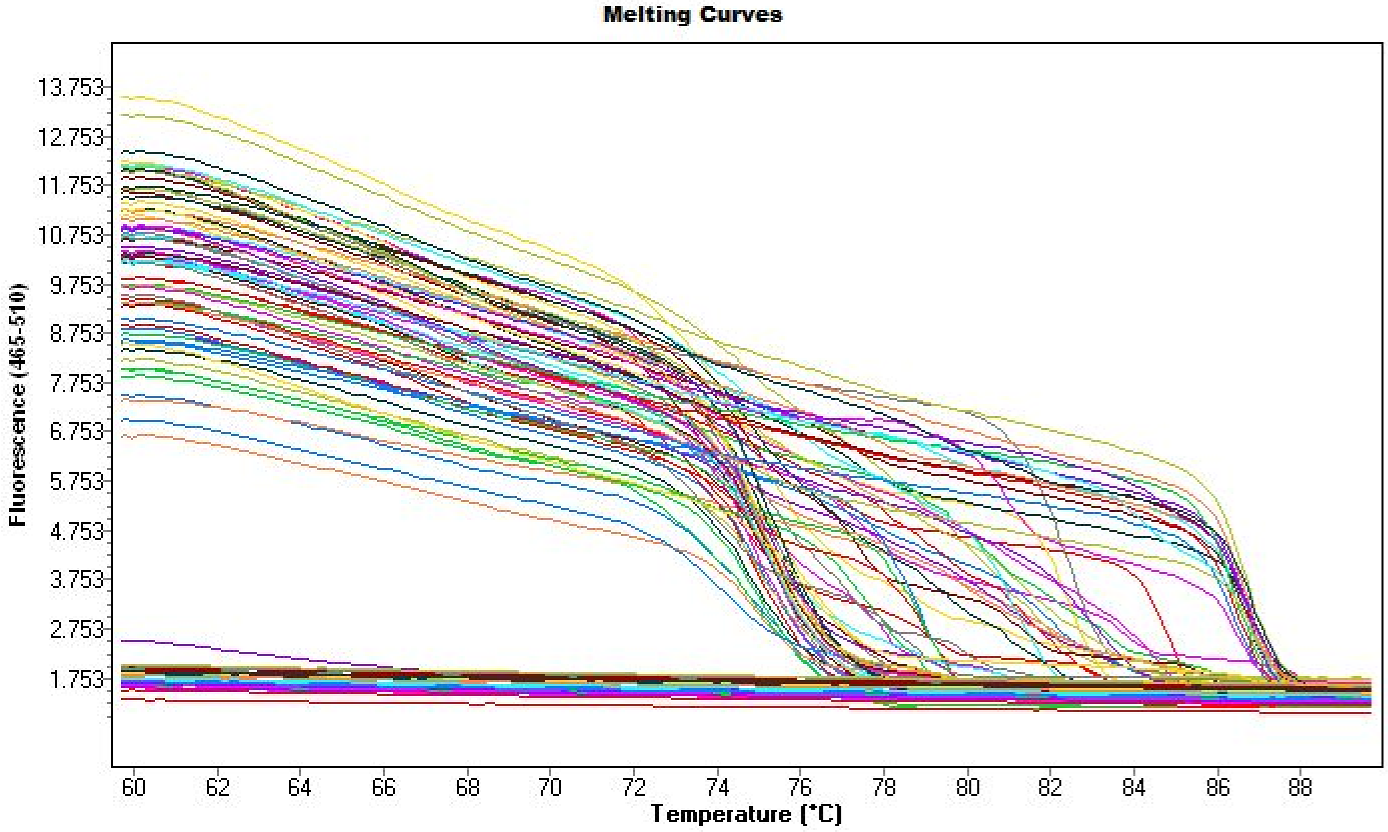

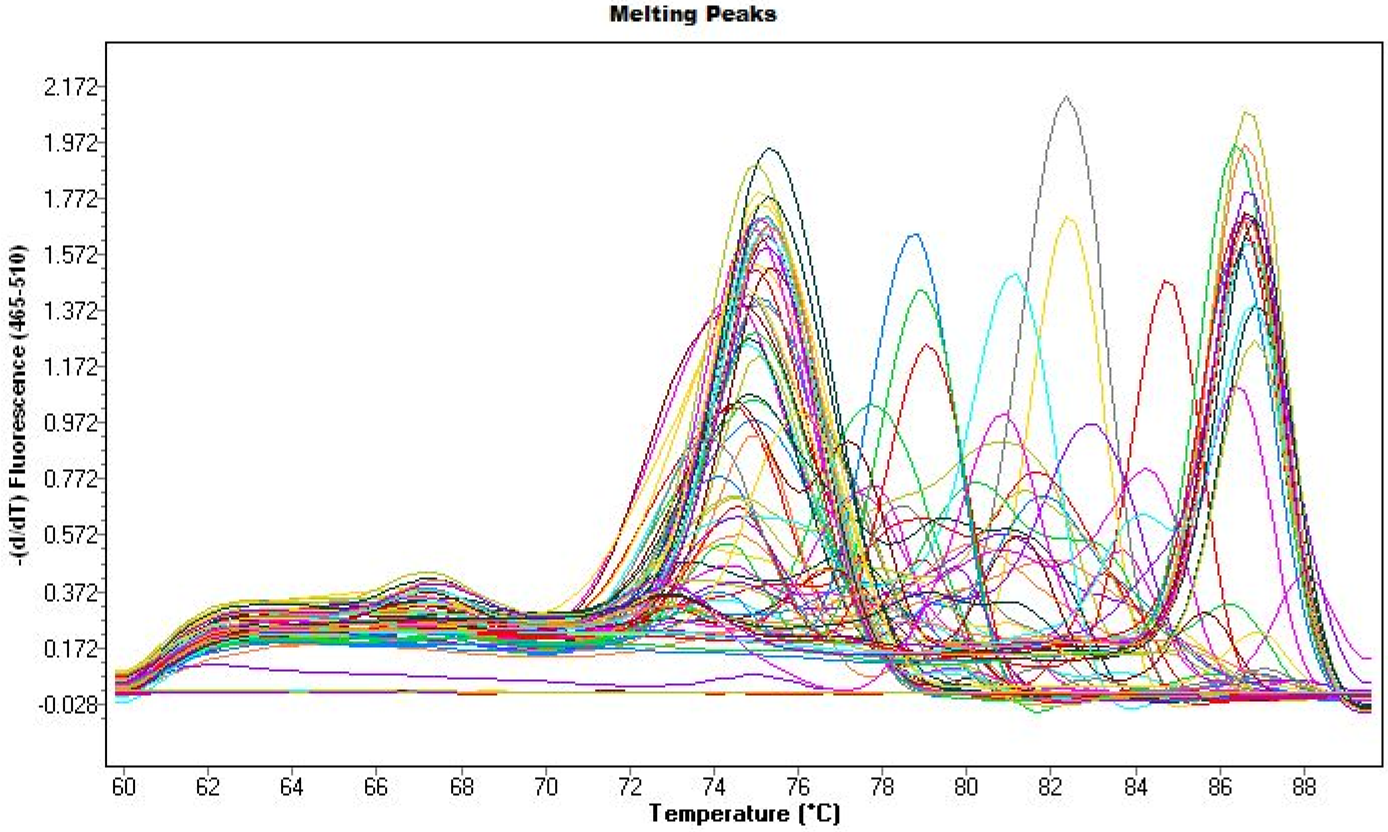

Analysis Notes	

Tm Calling for All Samples (Tm Calling)

Settings

Channel	465-510	Color Compensation	Off		
Program	Program				
Show Shoulders	False	Chemistry	HybProbe	Max Peaks	2

Subset Name	All Samples

Results

Inc	Pos	Sample Name	Peak 1 Tm	Area	Width	Height	Peak 2 Tm	Area	Width	Height	Status
☑	A1	GAPDH 1	74.90	4.35	4.24	1.03					
☑	A2	GAPDH 1	74.30	2.80	2.56	1.09	81.15	2.82	11.11	0.25	
☑	A3	GAPDH 1	74.82	4.98	4.59	1.09					
☑	A4	GAPDH 2	74.29	4.56	3.21	1.42					
☑	A5	GAPDH 2	75.13	4.08	2.97	1.37					
☑	A6	GAPDH 2	75.40	6.90	4.49	1.54					
☑	A7	GAPDH 3	74.84	6.59	4.82	1.37					
☑	A8	GAPDH 3	74.82	3.99	3.17	1.26					
☑	A9	GAPDH 3	74.81	4.42	3.53	1.25					
☑	A10	GAPDH 4	82.16	3.36	7.69	0.44					
☑	A11	GAPDH 4	74.42	0.60	2.32	0.26	81.10	2.94	6.50	0.45	
☑	A12	GAPDH 4	75.38	2.08	4.42	0.47	80.79	3.87	4.55	0.85	
☑	B1	FCER1A 1	75.41	4.56	3.18	1.44					
☑	B2	FCER1A 1	75.06	5.08	3.33	1.52					
☑	B3	FCER1A 1	75.08	4.55	3.44	1.32					
☑	B4	FCER1A 2	75.00	5.69	3.36	1.69					
☑	B5	FCER1A 2	75.38	5.43	3.17	1.71					
☑	B6	FCER1A 2	75.23	6.12	3.38	1.81					
☑	B7	FCER1A 3	75.44	4.95	3.22	1.54					
☑	B8	FCER1A 3	75.45	5.62	3.28	1.72					
☑	B9	FCER1A 3	75.62	5.93	3.32	1.79					
☑	B10	FCER1A 4	74.96	2.70	3.04	0.89					
☑	B11	FCER1A 4									
☑	B12	FCER1A 4	75.32	3.56	3.05	1.17					
☑	C1	RGS1 1	74.25	2.45	3.17	0.77	78.66	0.73	2.51	0.29	
☑	C2	RGS1 1	84.85	3.27	2.15	1.52					
☑	C3	RGS1 1	72.93	0.24	1.43	0.16	77.95	3.53	3.31	1.06	
☑	C4	RGS1 2	77.40	1.32	2.26	0.58	83.59	0.58	1.92	0.30	
☑	C5	RGS1 2	82.35	5.61	2.53	2.22					

Results

Inc	Pos	Sample Name	Peak 1				Peak 2				Status
			Tm	Area	Width	Height	Tm	Area	Width	Height	
☑	C6	RGS1 2	74.03	1.17	3.56	0.33	82.49	3.76	2.17	1.73	
☑	C7	RGS1 3									
☑	C8	RGS1 3									
☑	C9	RGS1 3	75.64	1.60	4.96	0.32					
☑	C10	RGS1 4									
☑	C11	RGS1 4									
☑	C12	RGS1 4									
☑	D1	GAPDH 5	77.02	1.25	5.33	0.24	82.24	2.39	3.91	0.61	
☑	D2	GAPDH 5	81.57	6.67	8.30	0.80					
☑	D3	GAPDH 5	73.21	0.25	1.97	0.13	81.34	3.22	5.68	0.57	
☑	D4	GAPDH 6	79.24	2.31	7.28	0.32	84.18	2.90	3.61	0.80	
☑	D5	GAPDH 6	75.55	5.66	4.12	1.37					
☑	D6	GAPDH 6	74.58	5.91	4.81	1.23					
☑	D7	GAPDH 7	75.57	4.75	5.36	0.89	81.34	0.93	1.84	0.51	
☑	D8	GAPDH 7	75.38	2.14	4.59	0.47	81.12	4.14	2.66	1.56	
☑	D9	GAPDH 7	75.16	3.80	3.46	1.10	80.61	4.14	5.49	0.75	
☑	D10	GAPDH 8	74.47	1.22	2.82	0.43	80.37	1.80	4.46	0.40	
☑	D11	GAPDH 8	74.57	1.09	2.26	0.48	82.96	3.60	3.77	0.95	
☑	D12	GAPDH 8	74.79	1.90	3.94	0.48	81.61	2.92	4.85	0.60	
☑	E1	FCER1A 5	73.92	0.31	1.54	0.20	78.78	4.18	2.46	1.70	
☑	E2	FCER1A 5	74.52	1.02	1.93	0.53	79.14	3.09	2.39	1.29	
☑	E3	FCER1A 5	74.25	0.71	1.76	0.40	78.96	3.57	2.37	1.51	
☑	E4	FCER1A 6	75.26	4.99	2.94	1.70					
☑	E5	FCER1A 6	75.33	5.62	3.23	1.74					
☑	E6	FCER1A 6	75.46	5.93	3.36	1.77					
☑	E7	FCER1A 7	75.33	5.29	3.26	1.62					
☑	E8	FCER1A 7	75.28	5.56	3.35	1.66					
☑	E9	FCER1A 7	75.46	6.83	3.45	1.98					
☑	E10	FCER1A 8	75.41	5.50	3.31	1.66					
☑	E11	FCER1A 8	75.33	5.24	3.45	1.52					
☑	E12	FCER1A 8	74.99	5.52	3.05	1.81					
☑	F1	RGS1 5	86.50	4.16	2.50	1.66					
☑	F2	RGS1 5	86.60	4.44	2.51	1.77					
☑	F3	RGS1 5	86.47	4.91	2.47	1.99					
☑	F4	RGS1 6	80.97	1.21	1.42	0.86	86.50	2.78	2.43	1.14	
☑	F5	RGS1 6	74.02	2.38	2.62	0.91	78.53	2.05	2.70	0.76	

Results

Inc	Pos	Sample Name	Peak 1				Peak 2				Status
			Tm	Area	Width	Height	Tm	Area	Width	Height	
☑	F6	RGS1 6	76.10	2.92	2.98	0.98					
☑	F7	RGS1 7									
☑	F8	RGS1 7									
☑	F9	RGS1 7									
☑	F10	RGS1 8									
☑	F11	RGS1 8	72.50	0.37	1.77	0.21					
☑	F12	RGS1 8	73.82	0.80	3.34	0.24					
☑	G1	blank									
☑	G2	blank									
☑	G3	blank									
☑	G4	blank									
☑	G5	blank									
☑	G6	blank									
☑	G7	blank									
☑	G8	blank									
☑	G9	blank									
☑	G10	blank									
☑	G11	blank									
☑	G12	blank									
☑	H1	blank									
☑	H2	blank									
☑	H3	blank									
☑	H4	blank									
☑	H5	blank									
☑	H6	blank									
☑	H7	blank									
☑	H8	blank									
☑	H9	blank									
☑	H10	blank									
☑	H11	blank									
☑	H12	blank									

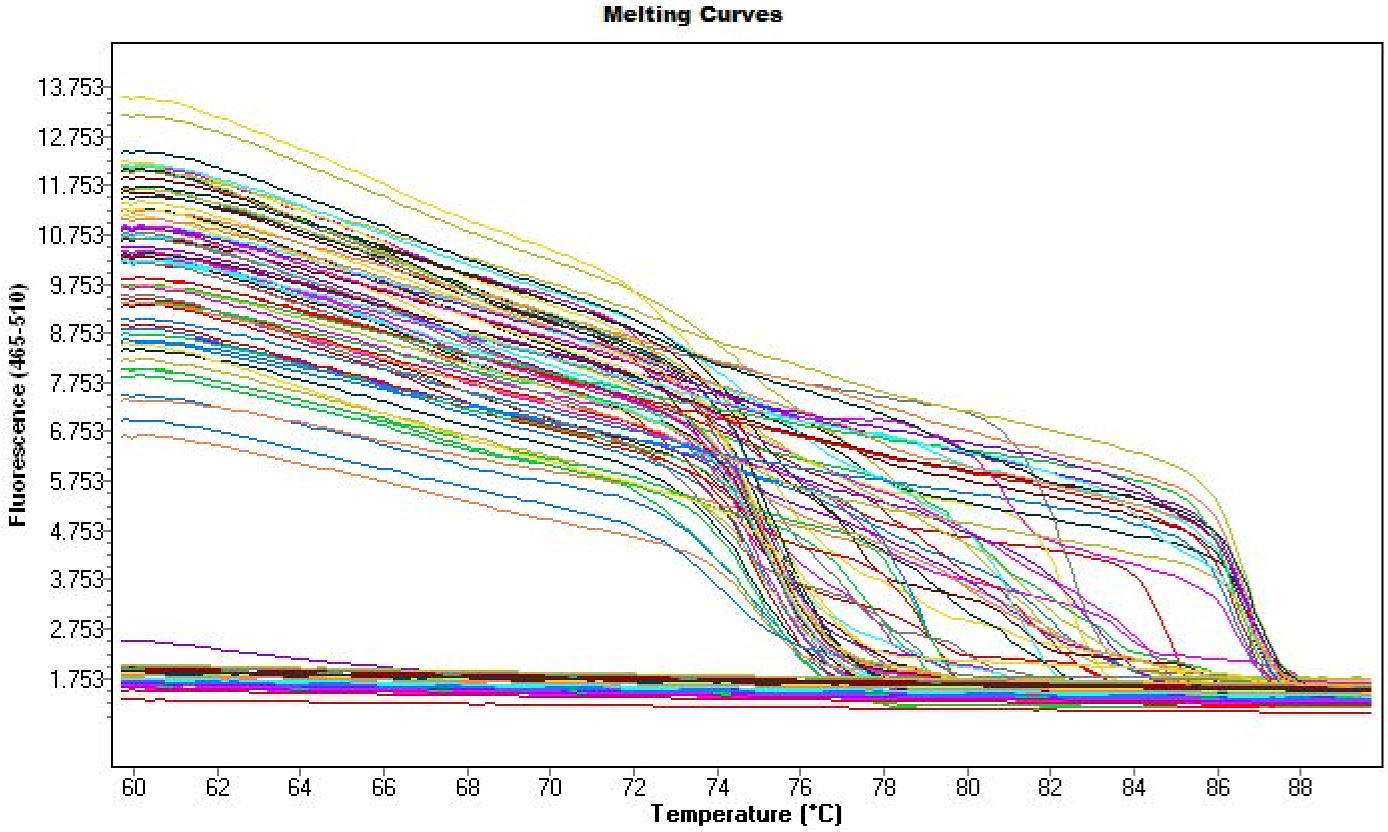

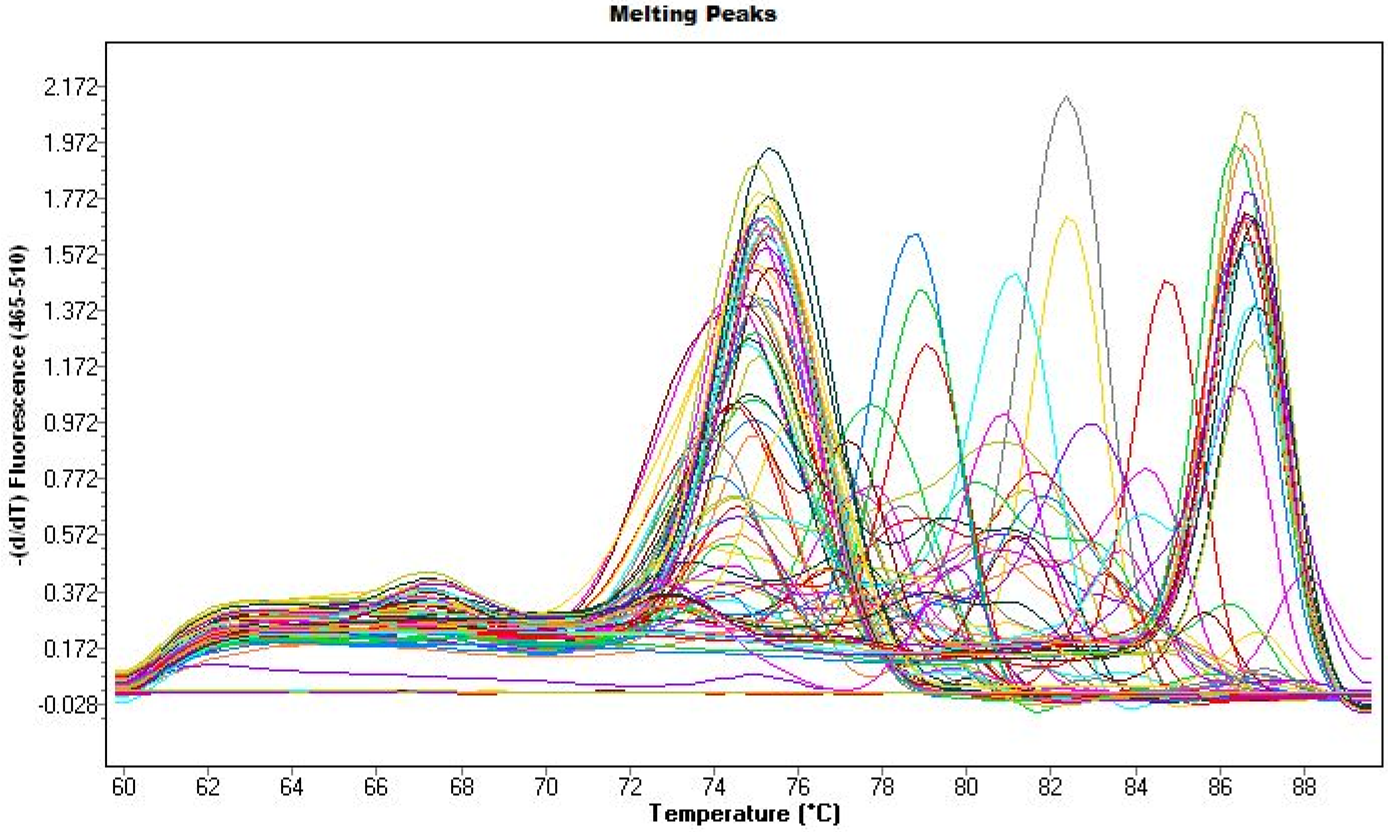

**Analysis Notes**	

Melt Curve Genotyping for All Samples (1) (Melt Curve Genotyping)

Channel	465-510	Color Compensation	Off
Progam Name	Program		
Stds Settings	Auto-Group		

Sensitivity	Normal	Temp Range	62-86	Score	0.7	Res	0.1

Subset Name	All Samples

Results

Inc	Pos.	Sample Name	Sample Type	Group	Score	Resolution	Status
☑	A1	GAPDH 1	Unknown	1	0.95	0.79	
☑	A2	GAPDH 1	Unknown	Unknown	0.65	0.18	
☑	A3	GAPDH 1	Unknown	1	0.95	0.80	
☑	A4	GAPDH 2	Unknown	1	0.86	0.53	
☑	A5	GAPDH 2	Unknown	1	0.89	0.40	
☑	A6	GAPDH 2	Unknown	1	0.96	0.65	
☑	A7	GAPDH 3	Unknown	1	0.93	0.72	
☑	A8	GAPDH 3	Unknown	1	0.90	0.59	
☑	A9	GAPDH 3	Unknown	1	0.97	0.83	
☑	A10	GAPDH 4	Unknown	3	0.97	0.28	
☑	A11	GAPDH 4	Unknown	3	1.00	0.40	
☑	A12	GAPDH 4	Unknown	3	0.98	0.50	
☑	B1	FCER1A 1	Unknown	1	0.96	0.71	
☑	B2	FCER1A 1	Unknown	1	0.97	0.78	
☑	B3	FCER1A 1	Unknown	1	0.95	0.77	
☑	B4	FCER1A 2	Unknown	1	0.91	0.73	
☑	B5	FCER1A 2	Unknown	1	0.94	0.71	
☑	B6	FCER1A 2	Unknown	1	0.96	0.73	
☑	B7	FCER1A 3	Unknown	1	0.97	0.66	
☑	B8	FCER1A 3	Unknown	1	0.97	0.69	
☑	B9	FCER1A 3	Unknown	1	0.97	0.63	
☑	B10	FCER1A 4	Unknown	1	0.95	0.72	
☑	B11	FCER1A 4	Unknown	Negative	1.00	1.00	
☑	B12	FCER1A 4	Unknown	1	1.00	0.71	
☑	C1	RGS1 1	Unknown	1	0.89	0.60	
☑	C2	RGS1 1	Unknown	2	0.78	0.19	
☑	C3	RGS1 1	Unknown	4	0.92	0.13	
☑	C4	RGS1 2	Unknown	3	0.84	0.22	
☑	C5	RGS1 2	Unknown	5	1.00	0.51	
☑	C6	RGS1 2	Unknown	Unknown	0.75	0.03	
☑	C7	RGS1 3	Unknown	2	0.95	0.37	

Results

Inc	Pos.	Sample Name	Sample Type	Group	Score	Resolution	Status
☑	C8	RGS1 3	Unknown	2	0.98	0.36	
☑	C9	RGS1 3	Unknown	2	0.91	0.41	
☑	C10	RGS1 4	Unknown	2	0.97	0.32	
☑	C11	RGS1 4	Unknown	2	0.99	0.39	
☑	C12	RGS1 4	Unknown	2	1.00	0.45	
☑	D1	GAPDH 5	Unknown	3	0.96	0.36	
☑	D2	GAPDH 5	Unknown	3	0.94	0.35	
☑	D3	GAPDH 5	Unknown	3	0.89	0.16	
☑	D4	GAPDH 6	Unknown	3	0.94	0.25	
☑	D5	GAPDH 6	Unknown	1	0.97	0.56	
☑	D6	GAPDH 6	Unknown	1	0.80	0.40	
☑	D7	GAPDH 7	Unknown	Unknown	0.62		
☑	D8	GAPDH 7	Unknown	3	0.89	0.32	
☑	D9	GAPDH 7	Unknown	3	0.75	0.16	
☑	D10	GAPDH 8	Unknown	3	0.93	0.37	
☑	D11	GAPDH 8	Unknown	3	0.85	0.11	
☑	D12	GAPDH 8	Unknown	3	0.93	0.36	
☑	E1	FCER1A 5	Unknown	4	1.00	0.53	
☑	E2	FCER1A 5	Unknown	Unknown	0.73	0.03	
☑	E3	FCER1A 5	Unknown	4	0.90	0.29	
☑	E4	FCER1A 6	Unknown	1	0.98	0.74	
☑	E5	FCER1A 6	Unknown	1	0.97	0.71	
☑	E6	FCER1A 6	Unknown	1	0.97	0.67	
☑	E7	FCER1A 7	Unknown	1	0.98	0.67	
☑	E8	FCER1A 7	Unknown	1	0.98	0.72	
☑	E9	FCER1A 7	Unknown	1	0.96	0.64	
☑	E10	FCER1A 8	Unknown	1	0.98	0.66	
☑	E11	FCER1A 8	Unknown	1	0.96	0.59	
☑	E12	FCER1A 8	Unknown	1	0.98	0.79	
☑	F1	RGS1 5	Unknown	2	0.97	0.44	
☑	F2	RGS1 5	Unknown	2	0.98	0.42	
☑	F3	RGS1 5	Unknown	2	0.96	0.49	
☑	F4	RGS1 6	Unknown	6	1.00	0.40	
☑	F5	RGS1 6	Unknown	Unknown	0.67	0.26	
☑	F6	RGS1 6	Unknown	1	0.87	0.42	
☑	F7	RGS1 7	Unknown	2	0.97	0.52	
☑	F8	RGS1 7	Unknown	2	0.89	0.23	
☑	F9	RGS1 7	Unknown	2	0.95	0.29	

Results

Inc	Pos.	Sample Name	Sample Type	Group	Score	Resolution	Status
☑	F10	RGS1 8	Unknown	2	0.98	0.43	
☑	F11	RGS1 8	Unknown	2	0.97	0.44	
☑	F12	RGS1 8	Unknown	2	0.93	0.53	
☑	G1	blank	Unknown	Negative	1.00	1.00	
☑	G2	blank	Unknown	Negative	1.00	1.00	
☑	G3	blank	Unknown	Negative	1.00	1.00	
☑	G4	blank	Unknown	Negative	1.00	1.00	
☑	G5	blank	Unknown	Negative	1.00	1.00	
☑	G6	blank	Unknown	Negative	1.00	1.00	
☑	G7	blank	Unknown	Negative	1.00	1.00	
☑	G8	blank	Unknown	Negative	1.00	1.00	
☑	G9	blank	Unknown	Negative	1.00	1.00	
☑	G10	blank	Unknown	Negative	1.00	1.00	
☑	G11	blank	Unknown	Negative	1.00	1.00	
☑	G12	blank	Unknown	Negative	1.00	1.00	
☑	H1	blank	Unknown	Negative	1.00	1.00	
☑	H2	blank	Unknown	Negative	1.00	1.00	
☑	H3	blank	Unknown	Negative	1.00	1.00	
☑	H4	blank	Unknown	Negative	1.00	1.00	
☑	H5	blank	Unknown	Negative	1.00	1.00	
☑	H6	blank	Unknown	Negative	1.00	1.00	
☑	H7	blank	Unknown	Negative	1.00	1.00	
☑	H8	blank	Unknown	Negative	1.00	1.00	
☑	H9	blank	Unknown	Negative	1.00	1.00	
☑	H10	blank	Unknown	Negative	1.00	1.00	
☑	H11	blank	Unknown	Negative	1.00	1.00	
☑	H12	blank	Unknown	Negative	1.00	1.00	

Group 1

Inc	Pos	Sample Name	Sample Type	Score	Resolution	Status
☑	A1	GAPDH 1	Unknown	0.95	0.79	
☑	A3	GAPDH 1	Unknown	0.95	0.80	
☑	A4	GAPDH 2	Unknown	0.86	0.53	
☑	A5	GAPDH 2	Unknown	0.89	0.40	
☑	A6	GAPDH 2	Unknown	0.96	0.65	
☑	A7	GAPDH 3	Unknown	0.93	0.72	
☑	A8	GAPDH 3	Unknown	0.90	0.59	
☑	A9	GAPDH 3	Unknown	0.97	0.83	

**Group**	1

Inc	Pos	Sample Name	Sample Type	Score	Resolution	Status
☑	B1	FCER1A 1	Unknown	0.96	0.71	
☑	B2	FCER1A 1	Unknown	0.97	0.78	
☑	B3	FCER1A 1	Unknown	0.95	0.77	
☑	B4	FCER1A 2	Unknown	0.91	0.73	
☑	B5	FCER1A 2	Unknown	0.94	0.71	
☑	B6	FCER1A 2	Unknown	0.96	0.73	
☑	B7	FCER1A 3	Unknown	0.97	0.66	
☑	B8	FCER1A 3	Unknown	0.97	0.69	
☑	B9	FCER1A 3	Unknown	0.97	0.63	
☑	B10	FCER1A 4	Unknown	0.95	0.72	
☑	B12	FCER1A 4	Unknown	1.00	0.71	
☑	C1	RGS1 1	Unknown	0.89	0.60	
☑	D5	GAPDH 6	Unknown	0.97	0.56	
☑	D6	GAPDH 6	Unknown	0.80	0.40	
☑	E4	FCER1A 6	Unknown	0.98	0.74	
☑	E5	FCER1A 6	Unknown	0.97	0.71	
☑	E6	FCER1A 6	Unknown	0.97	0.67	
☑	E7	FCER1A 7	Unknown	0.98	0.67	
☑	E8	FCER1A 7	Unknown	0.98	0.72	
☑	E9	FCER1A 7	Unknown	0.96	0.64	
☑	E10	FCER1A 8	Unknown	0.98	0.66	
☑	E11	FCER1A 8	Unknown	0.96	0.59	
☑	E12	FCER1A 8	Unknown	0.98	0.79	
☑	F6	RGS1 6	Unknown	0.87	0.42	

**Group**	2

Inc	Pos	Sample Name	Sample Type	Score	Resolution	Status
☑	C2	RGS1 1	Unknown	0.78	0.19	
☑	C7	RGS1 3	Unknown	0.95	0.37	
☑	C8	RGS1 3	Unknown	0.98	0.36	
☑	C9	RGS1 3	Unknown	0.91	0.41	
☑	C10	RGS1 4	Unknown	0.97	0.32	
☑	C11	RGS1 4	Unknown	0.99	0.39	
☑	C12	RGS1 4	Unknown	1.00	0.45	
☑	F1	RGS1 5	Unknown	0.97	0.44	
☑	F2	RGS1 5	Unknown	0.98	0.42	
☑	F3	RGS1 5	Unknown	0.96	0.49	
☑	F7	RGS1 7	Unknown	0.97	0.52	

Group	2

Inc	Pos	Sample Name	Sample Type	Score	Resolution	Status
☑	F8	RGS1 7	Unknown	0.89	0.23	
☑	F9	RGS1 7	Unknown	0.95	0.29	
☑	F10	RGS1 8	Unknown	0.98	0.43	
☑	F11	RGS1 8	Unknown	0.97	0.44	
☑	F12	RGS1 8	Unknown	0.93	0.53	

Group	3

Inc	Pos	Sample Name	Sample Type	Score	Resolution	Status
☑	A10	GAPDH 4	Unknown	0.97	0.28	
☑	A11	GAPDH 4	Unknown	1.00	0.40	
☑	A12	GAPDH 4	Unknown	0.98	0.50	
☑	C4	RGS1 2	Unknown	0.84	0.22	
☑	D1	GAPDH 5	Unknown	0.96	0.36	
☑	D2	GAPDH 5	Unknown	0.94	0.35	
☑	D3	GAPDH 5	Unknown	0.89	0.16	
☑	D4	GAPDH 6	Unknown	0.94	0.25	
☑	D8	GAPDH 7	Unknown	0.89	0.32	
☑	D9	GAPDH 7	Unknown	0.75	0.16	
☑	D10	GAPDH 8	Unknown	0.93	0.37	
☑	D11	GAPDH 8	Unknown	0.85	0.11	
☑	D12	GAPDH 8	Unknown	0.93	0.36	

Group	4

Inc	Pos	Sample Name	Sample Type	Score	Resolution	Status
☑	C3	RGS1 1	Unknown	0.92	0.13	
☑	E1	FCER1A 5	Unknown	1.00	0.53	
☑	E3	FCER1A 5	Unknown	0.90	0.29	

Group	5

Inc	Pos	Sample Name	Sample Type	Score	Resolution	Status
☑	C5	RGS1 2	Unknown	1.00	0.51	

Group	6

Inc	Pos	Sample Name	Sample Type	Score	Resolution	Status
☑	F4	RGS1 6	Unknown	1.00	0.40	

**Group**	Negative

Inc	Pos	Sample Name	Sample Type	Score	Resolution	Status
☑	B11	FCER1A 4	Unknown	1.00	1.00	
☑	G1	blank	Unknown	1.00	1.00	
☑	G2	blank	Unknown	1.00	1.00	
☑	G3	blank	Unknown	1.00	1.00	
☑	G4	blank	Unknown	1.00	1.00	
☑	G5	blank	Unknown	1.00	1.00	
☑	G6	blank	Unknown	1.00	1.00	
☑	G7	blank	Unknown	1.00	1.00	
☑	G8	blank	Unknown	1.00	1.00	
☑	G9	blank	Unknown	1.00	1.00	
☑	G10	blank	Unknown	1.00	1.00	
☑	G11	blank	Unknown	1.00	1.00	
☑	G12	blank	Unknown	1.00	1.00	
☑	H1	blank	Unknown	1.00	1.00	
☑	H2	blank	Unknown	1.00	1.00	
☑	H3	blank	Unknown	1.00	1.00	
☑	H4	blank	Unknown	1.00	1.00	
☑	H5	blank	Unknown	1.00	1.00	
☑	H6	blank	Unknown	1.00	1.00	
☑	H7	blank	Unknown	1.00	1.00	
☑	H8	blank	Unknown	1.00	1.00	
☑	H9	blank	Unknown	1.00	1.00	
☑	H10	blank	Unknown	1.00	1.00	
☑	H11	blank	Unknown	1.00	1.00	
☑	H12	blank	Unknown	1.00	1.00	

**Group**	Unknown

Inc	Pos	Sample Name	Sample Type	Score	Resolution	Status
☑	A2	GAPDH 1	Unknown	0.65	0.18	
☑	C6	RGS1 2	Unknown	0.75	0.03	
☑	D7	GAPDH 7	Unknown	0.62		
☑	E2	FCER1A 5	Unknown	0.73	0.03	
☑	F5	RGS1 6	Unknown	0.67	0.26	

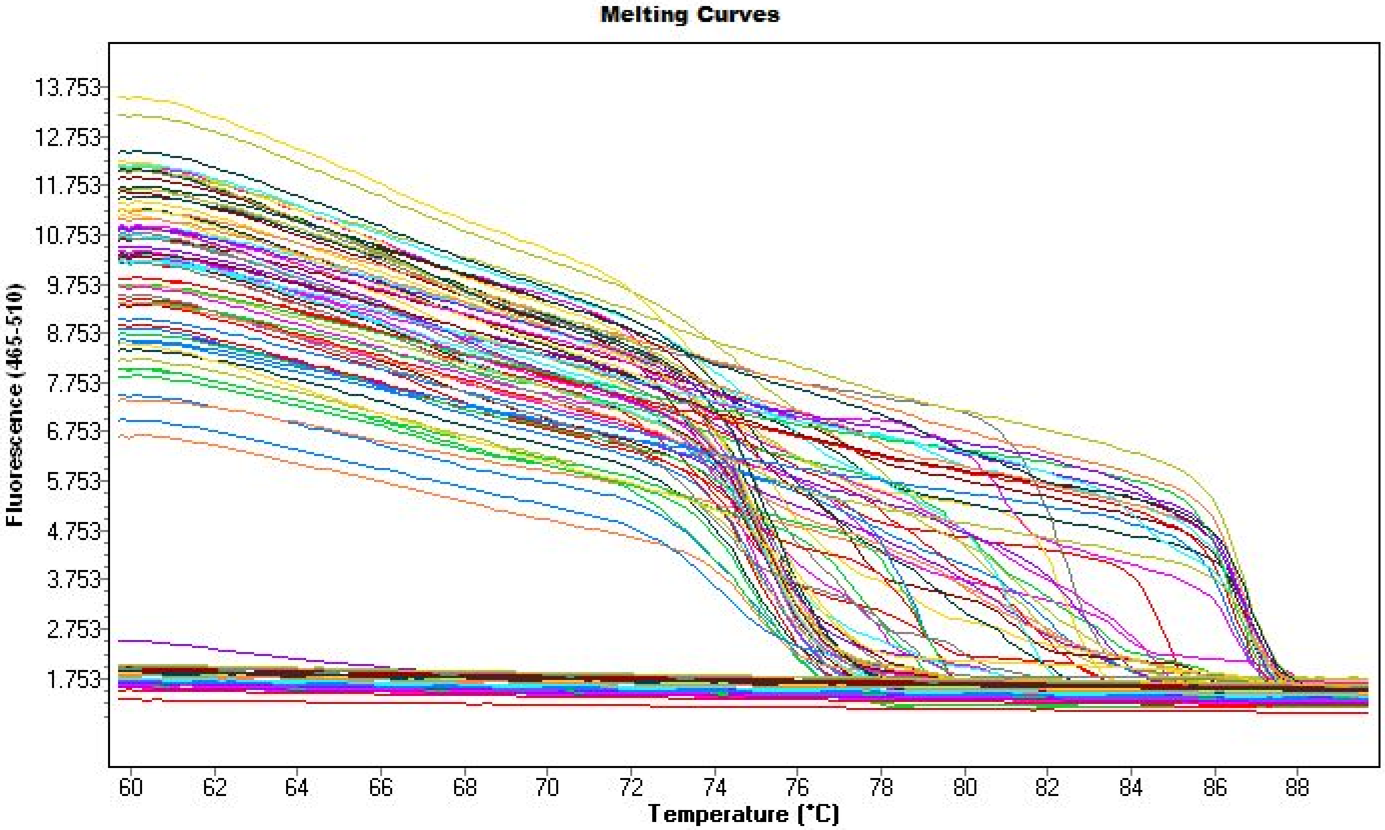

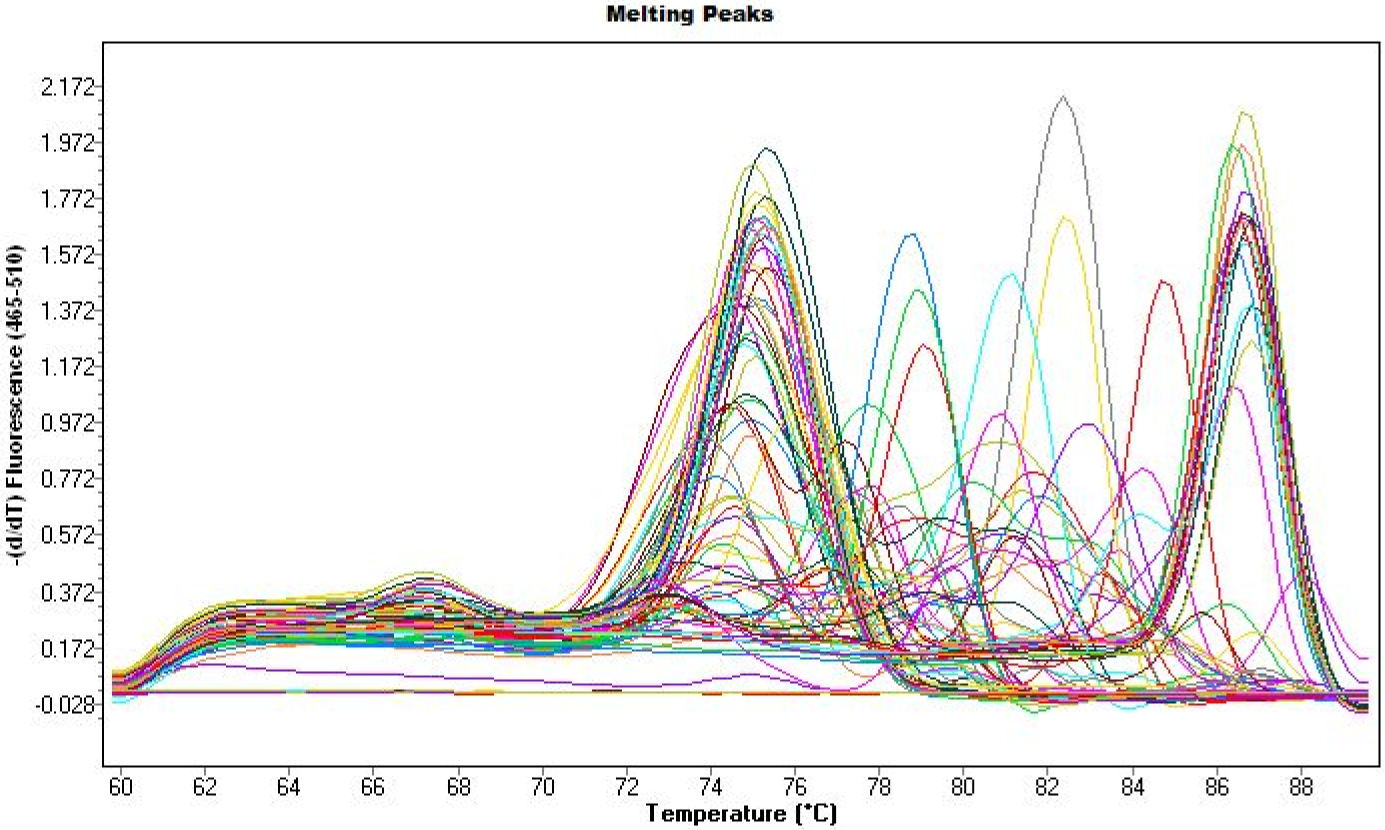

**Analysis Notes**	
