# Supplementary material for: Exploring differential gene expression and biomarker potential in systemic lupus erythematosus: a retrospective study
Source: PeerJ. 2025 Sep 8;13:e19891. doi: 10.7717/peerj.19891 (PMC12424612; doi:10.7717/peerj.19891)
Supplement: Supplemental Information 2 — These data are new and were recently supplemented with samples. The data include the Ct value for each sample, the Ct value for reference genes (e.g., GAPDH), and the relative expression levels calculated using the ΔΔCt method. These data were used to validate the RNA sequencing results and further evaluate the potential of these genes as biomarkers for SLE. [file peerj-13-19891-s002.zip › 20250528xj/20250528 SLE2.PDF]

# cobas® 4800 User Defined Workflow (UDF)

## xj20250528 SLE2

### Experiment

| | | | |
|---|---|---|---|
| Creation Date | 5/28/2025 9:33:36 AM | Last Modified Date | 6/2/2025 2:48:19 AM |
| Operator | System Admin | Owner | System Admin |
| Start Time | 5/28/2025 9:38:15 AM | End Time | 5/28/2025 10:37:47 AM |
| Run State | Completed | Software Version | LCS480 1.5.1.62 SP3 - UDF 2.1.0.26 |
| Macro | | Macro Owner | |
| Macro Status | | | |
| Templates | gongying | Plate ID | |
| Test ID | | Lot ID | |
| Color Comp ID | | | |
| Run Notes | | | |

### Programs

| | | | |
|---|---|---|---|
| Program Name | Program | | |
| Cycles | 1 | Analysis Mode | None |

| Target (°C) | Acquisition Mode | Hold (hh:mm:ss) | Ramp Rate (°C/s) | Acquisitions (per °C) | Sec Target (°C) | Step size (°C) | Step Delay (cycles) |
|---|---|---|---|---|---|---|---|
| 95 | None | 00:00:30 | 4.40 | | 0 | 0 | 0 |

| | | | |
|---|---|---|---|
| Program Name | Program | | |
| Cycles | 40 | Analysis Mode | Quantification |

| Target (°C) | Acquisition Mode | Hold (hh:mm:ss) | Ramp Rate (°C/s) | Acquisitions (per °C) | Sec Target (°C) | Step size (°C) | Step Delay (cycles) |
|---|---|---|---|---|---|---|---|
| 95 | None | 00:00:15 | 4.40 | | 0 | 0 | 0 |
| 60 | Single | 00:00:30 | 2.20 | | 0 | 0 | 0 |

| | | | |
|---|---|---|---|
| Program Name | Program | | |
| Cycles | 1 | Analysis Mode | Melting Curves |

| Target (°C) | Acquisition Mode | Hold (hh:mm:ss) | Ramp Rate (°C/s) | Acquisitions (per °C) | Sec Target (°C) | Step size (°C) | Step Delay (cycles) |
|---|---|---|---|---|---|---|---|
| 95 | None | 00:01:00 | 4.40 | | 0 | 0 | 0 |
| 60 | None | 00:00:30 | 2.20 | | 0 | 0 | 0 |
| 90 | Continuous | | 0.11 | 5 | 0 | 0 | 0 |

| | | | |
|---|---|---|---|
| Program Name | Program | | |
| Cycles | 1 | Analysis Mode | None |

| Target (°C) | Acquisition Mode | Hold (hh:mm:ss) | Ramp Rate (°C/s) | Acquisitions (per °C) | Sec Target (°C) | Step size (°C) | Step Delay (cycles) |
|---|---|---|---|---|---|---|---|

| Target (°C) | Acquisition Mode | Hold (hh:mm:ss) | Ramp Rate (°C/s) | Acquisitions (per °C) | Sec Target (°C) | Step size (°C) | Step Delay (cycles) |
|---|---|---|---|---|---|---|---|
| 40 | None | 00:00:30 | 2.20 | | 0 | 0 | 0 |

**Samples**

| | |
|---|---|
| Sample Count | 96 |
| Subset | All Samples |

| Pos | Name | ID | Repl. Of | Sample Notes |
|---|---|---|---|---|
| A1 | GAPDH 9 | | | |
| A2 | GAPDH 9 | | | |
| A3 | GAPDH 9 | | | |
| A4 | GAPDH 10 | | | |
| A5 | GAPDH 10 | | | |
| A6 | GAPDH 10 | | | |
| A7 | GAPDH 11 | | | |
| A8 | GAPDH 11 | | | |
| A9 | GAPDH 11 | | | |
| A10 | GAPDH 12 | | | |
| A11 | GAPDH 12 | | | |
| A12 | GAPDH 12 | | | |
| B1 | FCER1A 9 | | | |
| B2 | FCER1A 9 | | | |
| B3 | FCER1A 9 | | | |
| B4 | FCER1A 10 | | | |
| B5 | FCER1A 10 | | | |
| B6 | FCER1A 10 | | | |
| B7 | FCER1A 11 | | | |
| B8 | FCER1A 11 | | | |
| B9 | FCER1A 11 | | | |
| B10 | FCER1A 12 | | | |
| B11 | FCER1A 12 | | | |
| B12 | FCER1A 12 | | | |
| C1 | RGS1 9 | | | |
| C2 | RGS1 9 | | | |
| C3 | RGS1 9 | | | |
| C4 | RGS1 10 | | | |
| C5 | RGS1 10 | | | |
| C6 | RGS1 10 | | | |
| C7 | RGS1 11 | | | |
| C8 | RGS1 11 | | | |
| C9 | RGS1 11 | | | |
| C10 | RGS1 12 | | | |
| C11 | RGS1 12 | | | |
| C12 | RGS1 12 | | | |

**Samples**

| | |
|---|---|
| Sample Count | 96 |
| Subset | All Samples |

| Pos | Name | ID | Repl. Of | Sample Notes |
|---|---|---|---|---|
| D1 | GAPDH 13 | | | |
| D2 | GAPDH 13 | | | |
| D3 | GAPDH 13 | | | |
| D4 | blank | | | |
| D5 | blank | | | |
| D6 | blank | | | |
| D7 | blank | | | |
| D8 | blank | | | |
| D9 | blank | | | |
| D10 | blank | | | |
| D11 | blank | | | |
| D12 | blank | | | |
| E1 | FCER1A 13 | | | |
| E2 | FCER1A 13 | | | |
| E3 | FCER1A 13 | | | |
| E4 | blank | | | |
| E5 | blank | | | |
| E6 | blank | | | |
| E7 | blank | | | |
| E8 | blank | | | |
| E9 | blank | | | |
| E10 | blank | | | |
| E11 | blank | | | |
| E12 | blank | | | |
| F1 | RGS1 13 | | | |
| F2 | RGS1 13 | | | |
| F3 | RGS1 13 | | | |
| F4 | blank | | | |
| F5 | blank | | | |
| F6 | blank | | | |
| F7 | blank | | | |
| F8 | blank | | | |
| F9 | blank | | | |
| F10 | blank | | | |
| F11 | blank | | | |
| F12 | blank | | | |
| G1 | blank | | | |
| G2 | blank | | | |
| G3 | blank | | | |

### Samples

| | |
|---|---|
| Sample Count | 96 |
| Subset | All Samples |

| Pos | Name | ID | Repl. Of | Sample Notes |
|---|---|---|---|---|
| G4 | blank | | | |
| G5 | blank | | | |
| G6 | blank | | | |
| G7 | blank | | | |
| G8 | blank | | | |
| G9 | blank | | | |
| G10 | blank | | | |
| G11 | blank | | | |
| G12 | blank | | | |
| H1 | blank | | | |
| H2 | blank | | | |
| H3 | blank | | | |
| H4 | blank | | | |
| H5 | blank | | | |
| H6 | blank | | | |
| H7 | blank | | | |
| H8 | blank | | | |
| H9 | blank | | | |
| H10 | blank | | | |
| H11 | blank | | | |
| H12 | blank | | | |

### Instrument

| | | | |
|---|---|---|---|
| Name | Z480 | Serial Number | 56319 |

### Revision History

| Revision | Date | User | Reason |
|---|---|---|---|
| | | | |

## Abs Quant/2nd Derivative Max for All Samples (Abs Quant/2nd Derivative Max)

### Settings

| | | | |
|---|---|---|---|
| Channel | 465-510 | | |
| Color Compensation | Off | | |
| Program | Program | Units | |
| Mode | High Confidence | | |

| | |
|---|---|
| Subset Name | All Samples |

### Results

> - Late CP call (last five cycles) has higher uncertainty, ? - Detector Call uncertain

| Inc | Pos | Name | Type | CP | Concentration | Standard | Status |
|---|---|---|---|---|---|---|---|

## Results

| Inc | Pos | Name | Type | CP | Concentration | Standard | Status |
|---|---|---|---|---|---|---|---|
| ☑ | A1 | GAPDH 9 | Unknown | 27.98 | | | |
| ☑ | A2 | GAPDH 9 | Unknown | 28.43 | | | |
| ☑ | A3 | GAPDH 9 | Unknown | 28.30 | | | |
| ☑ | A4 | GAPDH 10 | Unknown | 28.26 | | | |
| ☑ | A5 | GAPDH 10 | Unknown | 28.14 | | | |
| ☑ | A6 | GAPDH 10 | Unknown | 28.30 | | | |
| ☑ | A7 | GAPDH 11 | Unknown | 29.46 | | | |
| ☑ | A8 | GAPDH 11 | Unknown | 29.26 | | | |
| ☑ | A9 | GAPDH 11 | Unknown | 29.15 | | | |
| ☑ | A10 | GAPDH 12 | Unknown | 27.92 | | | |
| ☑ | A11 | GAPDH 12 | Unknown | 27.75 | | | |
| ☑ | A12 | GAPDH 12 | Unknown | 27.71 | | | |
| ☑ | B1 | FCER1A 9 | Unknown | 31.00 | | | |
| ☑ | B2 | FCER1A 9 | Unknown | 31.22 | | | |
| ☑ | B3 | FCER1A 9 | Unknown | 31.03 | | | |
| ☑ | B4 | FCER1A 10 | Unknown | 30.00 | | | |
| ☑ | B5 | FCER1A 10 | Unknown | 29.91 | | | |
| ☑ | B6 | FCER1A 10 | Unknown | 29.91 | | | |
| ☑ | B7 | FCER1A 11 | Unknown | 30.93 | | | |
| ☑ | B8 | FCER1A 11 | Unknown | 30.29 | | | |
| ☑ | B9 | FCER1A 11 | Unknown | 30.13 | | | |
| ☑ | B10 | FCER1A 12 | Unknown | 31.54 | | | |
| ☑ | B11 | FCER1A 12 | Unknown | 30.92 | | | |
| ☑ | B12 | FCER1A 12 | Unknown | 31.49 | | | |
| ☑ | C1 | RGS1 9 | Unknown | 35.00 | | | > |
| ☑ | C2 | RGS1 9 | Unknown | 35.00 | | | > |
| ☑ | C3 | RGS1 9 | Unknown | 35.00 | | | > |
| ☑ | C4 | RGS1 10 | Unknown | 31.61 | | | |
| ☑ | C5 | RGS1 10 | Unknown | 31.94 | | | |
| ☑ | C6 | RGS1 10 | Unknown | 32.62 | | | |
| ☑ | C7 | RGS1 11 | Unknown | 29.14 | | | |
| ☑ | C8 | RGS1 11 | Unknown | 29.31 | | | |
| ☑ | C9 | RGS1 11 | Unknown | 27.91 | | | |
| ☑ | C10 | RGS1 12 | Unknown | 29.96 | | | |
| ☑ | C11 | RGS1 12 | Unknown | 31.09 | | | |
| ☑ | C12 | RGS1 12 | Unknown | 31.10 | | | |
| ☑ | D1 | GAPDH 13 | Unknown | 28.80 | | | |

> - Late Cp call (last five cycles) has higher uncertainty, ? - Detector Call uncertain

**Results**

| Inc | Pos | Name | Type | CP | Concentration | Standard | Status |
|---|---|---|---|---|---|---|---|
| ☑ | D2 | GAPDH 13 | Unknown | 28.80 | | | |
| ☑ | D3 | GAPDH 13 | Unknown | 30.54 | | | |
| ☑ | D4 | blank | Unknown | | | | |
| ☑ | D5 | blank | Unknown | | | | |
| ☑ | D6 | blank | Unknown | | | | |
| ☑ | D7 | blank | Unknown | | | | |
| ☑ | D8 | blank | Unknown | | | | |
| ☑ | D9 | blank | Unknown | | | | |
| ☑ | D10 | blank | Unknown | | | | |
| ☑ | D11 | blank | Unknown | | | | |
| ☑ | D12 | blank | Unknown | | | | |
| ☑ | E1 | FCER1A 13 | Unknown | 34.19 | | | |
| ☑ | E2 | FCER1A 13 | Unknown | 32.17 | | | |
| ☑ | E3 | FCER1A 13 | Unknown | 35.00 | | | > |
| ☑ | E4 | blank | Unknown | | | | |
| ☑ | E5 | blank | Unknown | | | | |
| ☑ | E6 | blank | Unknown | | | | |
| ☑ | E7 | blank | Unknown | | | | |
| ☑ | E8 | blank | Unknown | | | | |
| ☑ | E9 | blank | Unknown | | | | |
| ☑ | E10 | blank | Unknown | | | | |
| ☑ | E11 | blank | Unknown | | | | |
| ☑ | E12 | blank | Unknown | | | | |
| ☑ | F1 | RGS1 13 | Unknown | 31.79 | | | |
| ☑ | F2 | RGS1 13 | Unknown | 31.51 | | | |
| ☑ | F3 | RGS1 13 | Unknown | 31.56 | | | |
| ☑ | F4 | blank | Unknown | | | | |
| ☑ | F5 | blank | Unknown | | | | |
| ☑ | F6 | blank | Unknown | | | | |
| ☑ | F7 | blank | Unknown | | | | |
| ☑ | F8 | blank | Unknown | | | | |
| ☑ | F9 | blank | Unknown | | | | |
| ☑ | F10 | blank | Unknown | | | | |
| ☑ | F11 | blank | Unknown | | | | |
| ☑ | F12 | blank | Unknown | | | | |
| ☑ | G1 | blank | Unknown | | | | |
| ☑ | G2 | blank | Unknown | | | | |

> - Late Cp call (last five cycles) has higher uncertainty, ? - Detector Call uncertain

**Results**

| Inc | Pos | Name | Type | CP | Concentration | Standard | Status |
|---|---|---|---|---|---|---|---|
| ☑ | G3 | blank | Unknown | | | | |
| ☑ | G4 | blank | Unknown | | | | |
| ☑ | G5 | blank | Unknown | | | | |
| ☑ | G6 | blank | Unknown | | | | |
| ☑ | G7 | blank | Unknown | | | | |
| ☑ | G8 | blank | Unknown | | | | |
| ☑ | G9 | blank | Unknown | | | | |
| ☑ | G10 | blank | Unknown | | | | |
| ☑ | G11 | blank | Unknown | | | | |
| ☑ | G12 | blank | Unknown | | | | |
| ☑ | H1 | blank | Unknown | | | | |
| ☑ | H2 | blank | Unknown | 18.86 | | | ? |
| ☑ | H3 | blank | Unknown | | | | |
| ☑ | H4 | blank | Unknown | | | | |
| ☑ | H5 | blank | Unknown | | | | |
| ☑ | H6 | blank | Unknown | | | | |
| ☑ | H7 | blank | Unknown | | | | |
| ☑ | H8 | blank | Unknown | | | | |
| ☑ | H9 | blank | Unknown | | | | |
| ☑ | H10 | blank | Unknown | | | | |
| ☑ | H11 | blank | Unknown | 17.57 | | | ? |
| ☑ | H12 | blank | Unknown | | | | |

> - Late Cp call (last five cycles) has higher uncertainty, ? - Detector Call uncertain

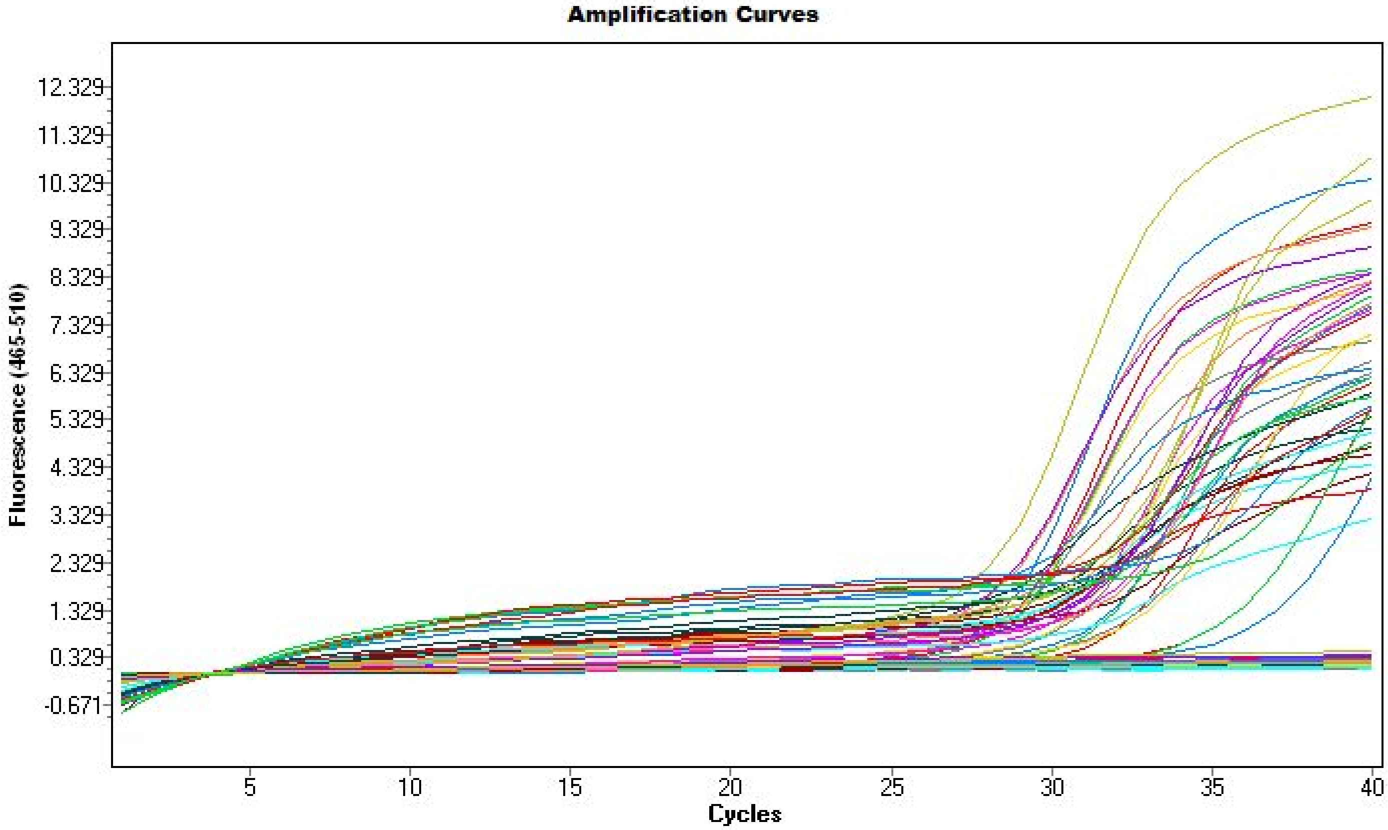


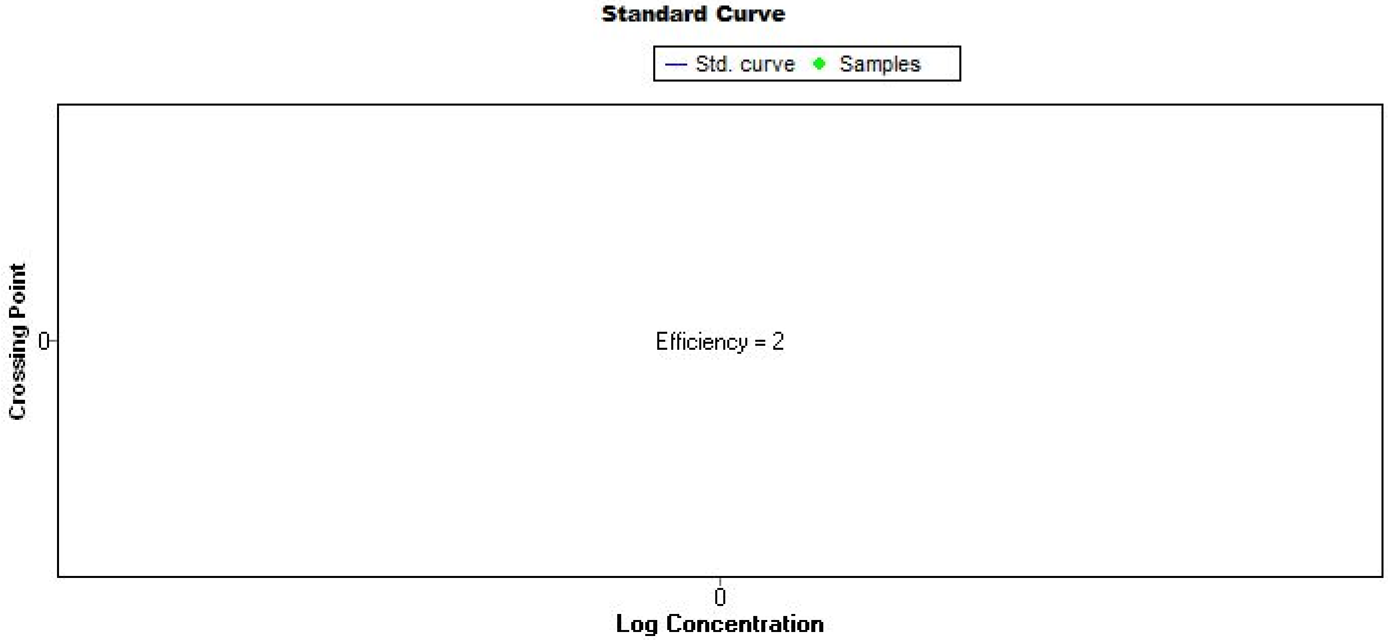


| Analysis Notes | |
|---|---|

## Abs Quant/Fit Points for All Samples (Abs Quant/Fit Points)

**Settings**

| | | | | | |
|---|---|---|---|---|---|
| Channel | 465-510 | | | | |
| Color Compensation | Off | | | | |
| Standard Curve | Set Efficiency (2.00) | | | | |
| Program | Program | | | Units | |
| First Cycle | 1 | Last Cycle | 40 | Background | 2-6 |
| Noiseband Method | Auto | Noiseband | 2.0580 | STD Dev Multiplier | 12.0000 |
| # of Fit Points | 2 | Threshold Method | Auto | Threshold | 2.0580 |

| | |
|---|---|
| Subset Name | All Samples |

**Results**

| Inc | Pos | Name | Type | CP | Concentration | Standard | Status |
|---|---|---|---|---|---|---|---|
| ☑ | A1 | GAPDH 9 | Unknown | 28.33 | | | |
| ☑ | A2 | GAPDH 9 | Unknown | 28.66 | | | |
| ☑ | A3 | GAPDH 9 | Unknown | 28.84 | | | |
| ☑ | A4 | GAPDH 10 | Unknown | 28.59 | | | |
| ☑ | A5 | GAPDH 10 | Unknown | 28.75 | | | |
| ☑ | A6 | GAPDH 10 | Unknown | 28.63 | | | |
| ☑ | A7 | GAPDH 11 | Unknown | 27.34 | | | |
| ☑ | A8 | GAPDH 11 | Unknown | 28.42 | | | |
| ☑ | A9 | GAPDH 11 | Unknown | 22.31 | | | |
| ☑ | A10 | GAPDH 12 | Unknown | 26.79 | | | |
| ☑ | A11 | GAPDH 12 | Unknown | 26.62 | | | |
| ☑ | A12 | GAPDH 12 | Unknown | 24.68 | | | |
| ☑ | B1 | FCER1A 9 | Unknown | 31.62 | | | |
| ☑ | B2 | FCER1A 9 | Unknown | 32.50 | | | |
| ☑ | B3 | FCER1A 9 | Unknown | 31.73 | | | |
| ☑ | B4 | FCER1A 10 | Unknown | 30.34 | | | |
| ☑ | B5 | FCER1A 10 | Unknown | 30.75 | | | |
| ☑ | B6 | FCER1A 10 | Unknown | 30.59 | | | |
| ☑ | B7 | FCER1A 11 | Unknown | 29.40 | | | |
| ☑ | B8 | FCER1A 11 | Unknown | 31.91 | | | |
| ☑ | B9 | FCER1A 11 | Unknown | 17.11 | | | |
| ☑ | B10 | FCER1A 12 | Unknown | 30.58 | | | |
| ☑ | B11 | FCER1A 12 | Unknown | 29.62 | | | |
| ☑ | B12 | FCER1A 12 | Unknown | 25.55 | | | |
| ☑ | C1 | RGS1 9 | Unknown | 36.64 | | | |
| ☑ | C2 | RGS1 9 | Unknown | 35.55 | | | |
| ☑ | C3 | RGS1 9 | Unknown | 35.58 | | | |

**Results**

| Inc | Pos | Name | Type | CP | Concentration | Standard | Status |
|---|---|---|---|---|---|---|---|
| ☑ | C4 | RGS1 10 | Unknown | 30.32 | | | |
| ☑ | C5 | RGS1 10 | Unknown | 32.44 | | | |
| ☑ | C6 | RGS1 10 | Unknown | 32.64 | | | |
| ☑ | C7 | RGS1 11 | Unknown | 29.30 | | | |
| ☑ | C8 | RGS1 11 | Unknown | 28.90 | | | |
| ☑ | C9 | RGS1 11 | Unknown | 24.66 | | | |
| ☑ | C10 | RGS1 12 | Unknown | 25.89 | | | |
| ☑ | C11 | RGS1 12 | Unknown | 29.73 | | | |
| ☑ | C12 | RGS1 12 | Unknown | 26.91 | | | |
| ☑ | D1 | GAPDH 13 | Unknown | 14.66 | | | |
| ☑ | D2 | GAPDH 13 | Unknown | 29.41 | | | |
| ☑ | D3 | GAPDH 13 | Unknown | 10.92 | | | |
| ☑ | D4 | blank | Unknown | | | | |
| ☑ | D5 | blank | Unknown | | | | |
| ☑ | D6 | blank | Unknown | | | | |
| ☑ | D7 | blank | Unknown | | | | |
| ☑ | D8 | blank | Unknown | | | | |
| ☑ | D9 | blank | Unknown | | | | |
| ☑ | D10 | blank | Unknown | | | | |
| ☑ | D11 | blank | Unknown | | | | |
| ☑ | D12 | blank | Unknown | | | | |
| ☑ | E1 | FCER1A 13 | Unknown | 11.79 | | | |
| ☑ | E2 | FCER1A 13 | Unknown | 11.52 | | | |
| ☑ | E3 | FCER1A 13 | Unknown | 11.59 | | | |
| ☑ | E4 | blank | Unknown | | | | |
| ☑ | E5 | blank | Unknown | | | | |
| ☑ | E6 | blank | Unknown | | | | |
| ☑ | E7 | blank | Unknown | | | | |
| ☑ | E8 | blank | Unknown | | | | |
| ☑ | E9 | blank | Unknown | | | | |
| ☑ | E10 | blank | Unknown | | | | |
| ☑ | E11 | blank | Unknown | | | | |
| ☑ | E12 | blank | Unknown | | | | |
| ☑ | F1 | RGS1 13 | Unknown | 17.10 | | | |
| ☑ | F2 | RGS1 13 | Unknown | 12.90 | | | |
| ☑ | F3 | RGS1 13 | Unknown | 15.05 | | | |
| ☑ | F4 | blank | Unknown | | | | |

**Results**

| Inc | Pos | Name | Type | CP | Concentration | Standard | Status |
|---|---|---|---|---|---|---|---|
| ☑ | F5 | blank | Unknown | | | | |
| ☑ | F6 | blank | Unknown | | | | |
| ☑ | F7 | blank | Unknown | | | | |
| ☑ | F8 | blank | Unknown | | | | |
| ☑ | F9 | blank | Unknown | | | | |
| ☑ | F10 | blank | Unknown | | | | |
| ☑ | F11 | blank | Unknown | | | | |
| ☑ | F12 | blank | Unknown | | | | |
| ☑ | G1 | blank | Unknown | | | | |
| ☑ | G2 | blank | Unknown | | | | |
| ☑ | G3 | blank | Unknown | | | | |
| ☑ | G4 | blank | Unknown | | | | |
| ☑ | G5 | blank | Unknown | | | | |
| ☑ | G6 | blank | Unknown | | | | |
| ☑ | G7 | blank | Unknown | | | | |
| ☑ | G8 | blank | Unknown | | | | |
| ☑ | G9 | blank | Unknown | | | | |
| ☑ | G10 | blank | Unknown | | | | |
| ☑ | G11 | blank | Unknown | | | | |
| ☑ | G12 | blank | Unknown | | | | |
| ☑ | H1 | blank | Unknown | | | | |
| ☑ | H2 | blank | Unknown | | | | |
| ☑ | H3 | blank | Unknown | | | | |
| ☑ | H4 | blank | Unknown | | | | |
| ☑ | H5 | blank | Unknown | | | | |
| ☑ | H6 | blank | Unknown | | | | |
| ☑ | H7 | blank | Unknown | | | | |
| ☑ | H8 | blank | Unknown | | | | |
| ☑ | H9 | blank | Unknown | | | | |
| ☑ | H10 | blank | Unknown | | | | |
| ☑ | H11 | blank | Unknown | | | | |
| ☑ | H12 | blank | Unknown | | | | |

## Amplification Curves

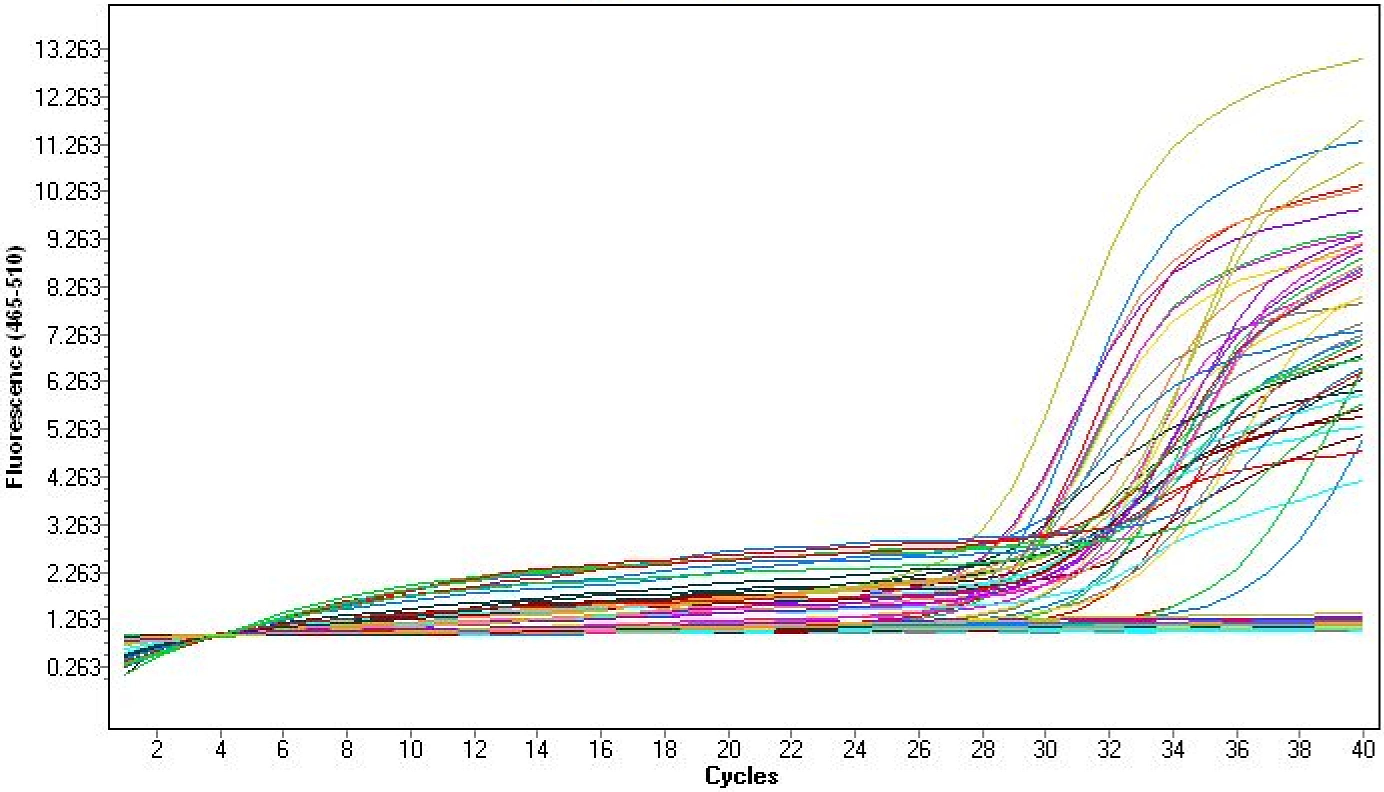


## Amplification Curves

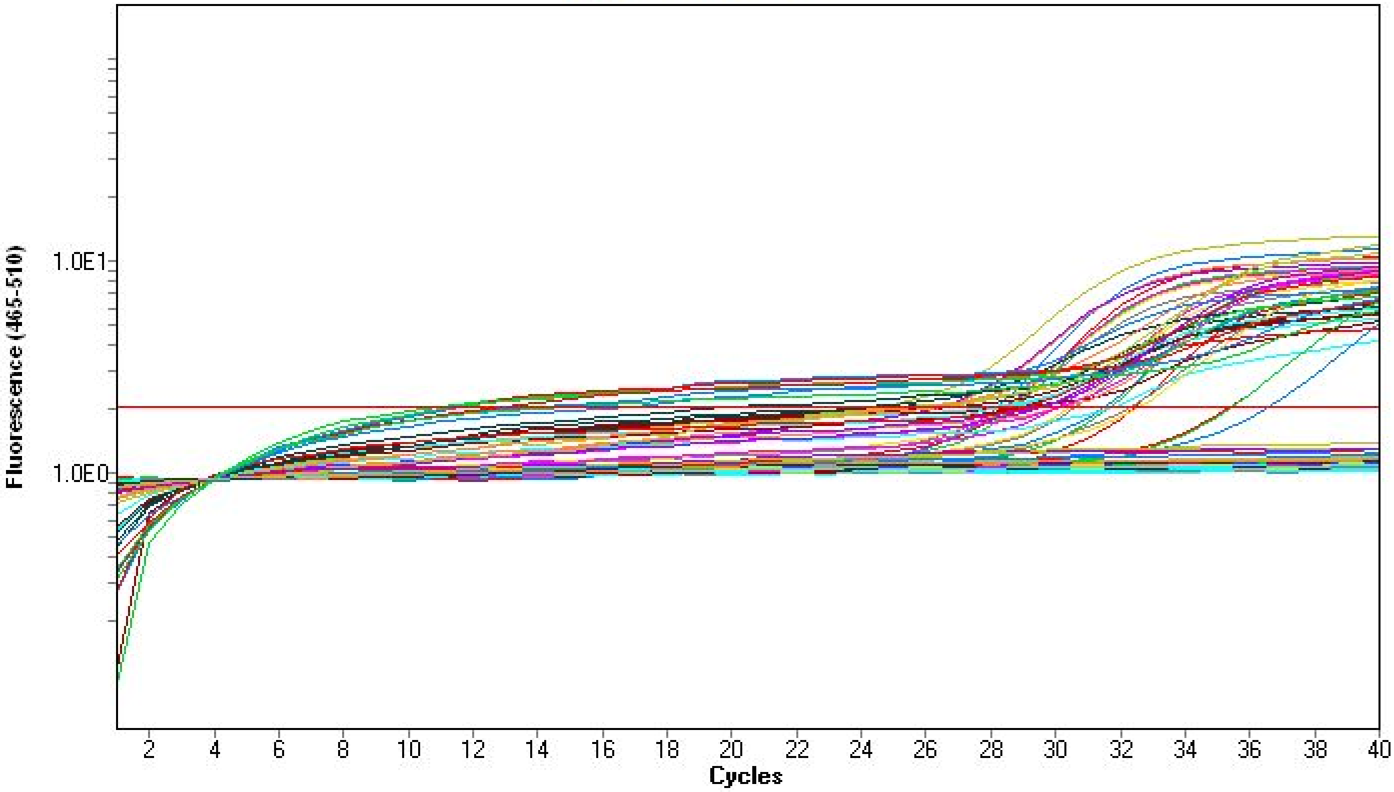

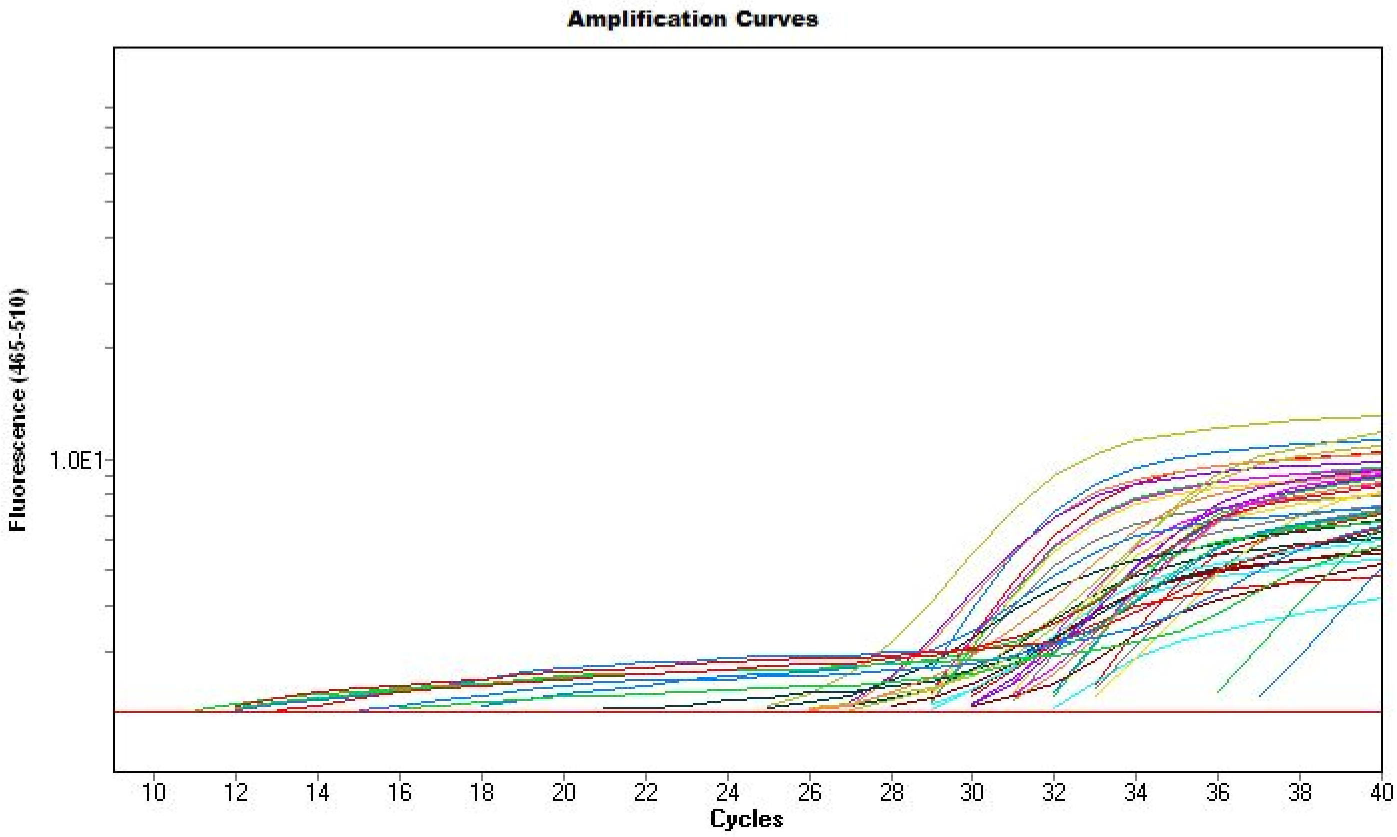


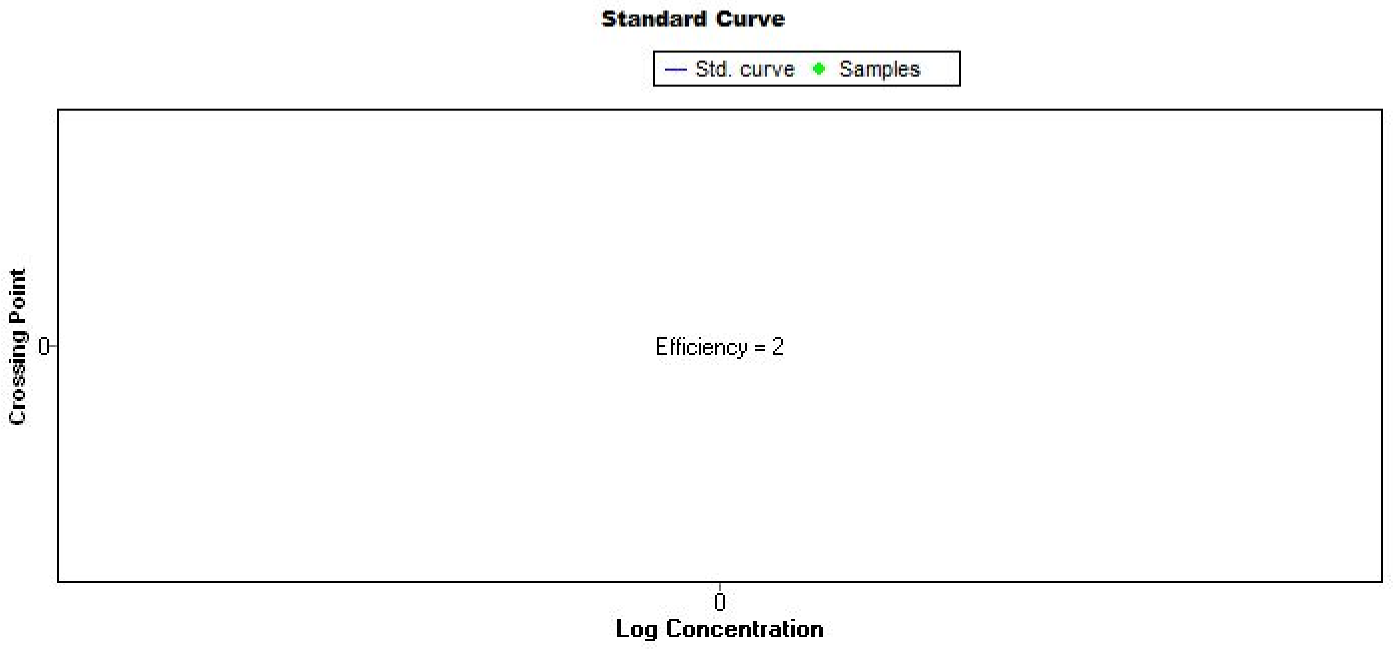


| Analysis Notes | |
|---|---|

## Gene Scanning for All Samples (Gene Scanning)

| | | | |
|---|---|---|---|
| Channel | 465-510 | Color Compensation | Off |
| Progam Name | Program | | |
| Stds Settings | Auto-Group | | |
| Sensitivity | 0.30 | Threshold (TmShift) | 5.000 |
| Pre-melt | 67.26-69.72 | Post-melt Temp | 87.78-88.77 |

| | |
|---|---|
| Subset Name | All Samples |

**Results**

| Inc | Pos. | Sample Name | Sample Type | Group | Status |
|---|---|---|---|---|---|
| ☑ | A1 | GAPDH 9 | Unknown | 3 | |
| ☑ | A2 | GAPDH 9 | Unknown | 4 | |
| ☑ | A3 | GAPDH 9 | Unknown | Unknown | |
| ☑ | A4 | GAPDH 10 | Unknown | 6 | |
| ☑ | A5 | GAPDH 10 | Unknown | 3 | |
| ☑ | A6 | GAPDH 10 | Unknown | 3 | |
| ☑ | A7 | GAPDH 11 | Unknown | Unknown | |
| ☑ | A8 | GAPDH 11 | Unknown | 4 | |
| ☑ | A9 | GAPDH 11 | Unknown | 4 | |
| ☑ | A10 | GAPDH 12 | Unknown | 4 | |
| ☑ | A11 | GAPDH 12 | Unknown | 3 | |
| ☑ | A12 | GAPDH 12 | Unknown | 3 | |
| ☑ | B1 | FCER1A 9 | Unknown | 1 | |
| ☑ | B2 | FCER1A 9 | Unknown | 1 | |
| ☑ | B3 | FCER1A 9 | Unknown | 1 | |
| ☑ | B4 | FCER1A 10 | Unknown | 1 | |
| ☑ | B5 | FCER1A 10 | Unknown | 1 | |
| ☑ | B6 | FCER1A 10 | Unknown | 1 | |
| ☑ | B7 | FCER1A 11 | Unknown | 1 | |
| ☑ | B8 | FCER1A 11 | Unknown | 1 | |
| ☑ | B9 | FCER1A 11 | Unknown | 1 | |
| ☑ | B10 | FCER1A 12 | Unknown | 1 | |
| ☑ | B11 | FCER1A 12 | Unknown | 1 | |
| ☑ | B12 | FCER1A 12 | Unknown | 1 | |
| ☑ | C1 | RGS1 9 | Unknown | Unknown | |
| ☑ | C2 | RGS1 9 | Unknown | 5 | |
| ☑ | C3 | RGS1 9 | Unknown | 5 | |
| ☑ | C4 | RGS1 10 | Unknown | 2 | |
| ☑ | C5 | RGS1 10 | Unknown | 2 | |
| ☑ | C6 | RGS1 10 | Unknown | 2 | |
| ☑ | C7 | RGS1 11 | Unknown | 2 | |

**Results**

| Inc | Pos. | Sample Name | Sample Type | Group | Status |
|---|---|---|---|---|---|
| ☑ | C8 | RGS1 11 | Unknown | 2 | |
| ☑ | C9 | RGS1 11 | Unknown | 2 | |
| ☑ | C10 | RGS1 12 | Unknown | Unknown | |
| ☑ | C11 | RGS1 12 | Unknown | Unknown | |
| ☑ | C12 | RGS1 12 | Unknown | 2 | |
| ☑ | D1 | GAPDH 13 | Unknown | Unknown | |
| ☑ | D2 | GAPDH 13 | Unknown | 4 | |
| ☑ | D3 | GAPDH 13 | Unknown | 3 | |
| ☑ | D4 | blank | Unknown | Negative | |
| ☑ | D5 | blank | Unknown | Negative | |
| ☑ | D6 | blank | Unknown | Negative | |
| ☑ | D7 | blank | Unknown | Negative | |
| ☑ | D8 | blank | Unknown | Negative | |
| ☑ | D9 | blank | Unknown | Negative | |
| ☑ | D10 | blank | Unknown | Negative | |
| ☑ | D11 | blank | Unknown | Negative | |
| ☑ | D12 | blank | Unknown | Negative | |
| ☑ | E1 | FCER1A 13 | Unknown | 1 | |
| ☑ | E2 | FCER1A 13 | Unknown | Unknown | |
| ☑ | E3 | FCER1A 13 | Unknown | 1 | |
| ☑ | E4 | blank | Unknown | Negative | |
| ☑ | E5 | blank | Unknown | Negative | |
| ☑ | E6 | blank | Unknown | Negative | |
| ☑ | E7 | blank | Unknown | Negative | |
| ☑ | E8 | blank | Unknown | Negative | |
| ☑ | E9 | blank | Unknown | Negative | |
| ☑ | E10 | blank | Unknown | Negative | |
| ☑ | E11 | blank | Unknown | Negative | |
| ☑ | E12 | blank | Unknown | Negative | |
| ☑ | F1 | RGS1 13 | Unknown | Unknown | |
| ☑ | F2 | RGS1 13 | Unknown | 2 | |
| ☑ | F3 | RGS1 13 | Unknown | 2 | |
| ☑ | F4 | blank | Unknown | Negative | |
| ☑ | F5 | blank | Unknown | Negative | |
| ☑ | F6 | blank | Unknown | Negative | |
| ☑ | F7 | blank | Unknown | Negative | |
| ☑ | F8 | blank | Unknown | Negative | |
| ☑ | F9 | blank | Unknown | Negative | |

**Results**

| Inc | Pos. | Sample Name | Sample Type | Group | Status |
|---|---|---|---|---|---|
| ☑ | F10 | blank | Unknown | Negative | |
| ☑ | F11 | blank | Unknown | Negative | |
| ☑ | F12 | blank | Unknown | Negative | |
| ☑ | G1 | blank | Unknown | Negative | |
| ☑ | G2 | blank | Unknown | Negative | |
| ☑ | G3 | blank | Unknown | Negative | |
| ☑ | G4 | blank | Unknown | Negative | |
| ☑ | G5 | blank | Unknown | Negative | |
| ☑ | G6 | blank | Unknown | Negative | |
| ☑ | G7 | blank | Unknown | Negative | |
| ☑ | G8 | blank | Unknown | Negative | |
| ☑ | G9 | blank | Unknown | Negative | |
| ☑ | G10 | blank | Unknown | Negative | |
| ☑ | G11 | blank | Unknown | Negative | |
| ☑ | G12 | blank | Unknown | Negative | |
| ☑ | H1 | blank | Unknown | Negative | |
| ☑ | H2 | blank | Unknown | Negative | |
| ☑ | H3 | blank | Unknown | Negative | |
| ☑ | H4 | blank | Unknown | Negative | |
| ☑ | H5 | blank | Unknown | Negative | |
| ☑ | H6 | blank | Unknown | Negative | |
| ☑ | H7 | blank | Unknown | Negative | |
| ☑ | H8 | blank | Unknown | Negative | |
| ☑ | H9 | blank | Unknown | Negative | |
| ☑ | H10 | blank | Unknown | Negative | |
| ☑ | H11 | blank | Unknown | Negative | |
| ☑ | H12 | blank | Unknown | Negative | |

| **Group** | 1 |
|---|---|

| Inc | Pos | Sample Name | Sample Type |
|---|---|---|---|
| ☑ | B1 | FCER1A 9 | Unknown |
| ☑ | B2 | FCER1A 9 | Unknown |
| ☑ | B3 | FCER1A 9 | Unknown |
| ☑ | B4 | FCER1A 10 | Unknown |
| ☑ | B5 | FCER1A 10 | Unknown |
| ☑ | B6 | FCER1A 10 | Unknown |
| ☑ | B7 | FCER1A 11 | Unknown |
| ☑ | B8 | FCER1A 11 | Unknown |
| ☑ | B9 | FCER1A 11 | Unknown |

| Group | 1 |
|---|---|

| Inc | Pos | Sample Name | Sample Type |
|---|---|---|---|
| ☑ | B10 | FCER1A 12 | Unknown |
| ☑ | B11 | FCER1A 12 | Unknown |
| ☑ | B12 | FCER1A 12 | Unknown |
| ☑ | E1 | FCER1A 13 | Unknown |
| ☑ | E3 | FCER1A 13 | Unknown |

| Group | 2 |
|---|---|

| Inc | Pos | Sample Name | Sample Type |
|---|---|---|---|
| ☑ | C4 | RGS1 10 | Unknown |
| ☑ | C5 | RGS1 10 | Unknown |
| ☑ | C6 | RGS1 10 | Unknown |
| ☑ | C7 | RGS1 11 | Unknown |
| ☑ | C8 | RGS1 11 | Unknown |
| ☑ | C9 | RGS1 11 | Unknown |
| ☑ | C12 | RGS1 12 | Unknown |
| ☑ | F2 | RGS1 13 | Unknown |
| ☑ | F3 | RGS1 13 | Unknown |

| Group | 3 |
|---|---|

| Inc | Pos | Sample Name | Sample Type |
|---|---|---|---|
| ☑ | A1 | GAPDH 9 | Unknown |
| ☑ | A5 | GAPDH 10 | Unknown |
| ☑ | A6 | GAPDH 10 | Unknown |
| ☑ | A11 | GAPDH 12 | Unknown |
| ☑ | A12 | GAPDH 12 | Unknown |
| ☑ | D3 | GAPDH 13 | Unknown |

| Group | 4 |
|---|---|

| Inc | Pos | Sample Name | Sample Type |
|---|---|---|---|
| ☑ | A2 | GAPDH 9 | Unknown |
| ☑ | A8 | GAPDH 11 | Unknown |
| ☑ | A9 | GAPDH 11 | Unknown |
| ☑ | A10 | GAPDH 12 | Unknown |
| ☑ | D2 | GAPDH 13 | Unknown |

| Group | 5 |
|---|---|

| Inc | Pos | Sample Name | Sample Type |
|---|---|---|---|
| ☑ | C2 | RGS1 9 | Unknown |
| ☑ | C3 | RGS1 9 | Unknown |

| Group | 6 |
|---|---|

| Inc | Pos | Sample Name | Sample Type |
|---|---|---|---|
| ☑ | A4 | GAPDH 10 | Unknown |

| Group | Negative |
|---|---|

| Inc | Pos | Sample Name | Sample Type |
|---|---|---|---|
| ☑ | D4 | blank | Unknown |
| ☑ | D5 | blank | Unknown |
| ☑ | D6 | blank | Unknown |
| ☑ | D7 | blank | Unknown |
| ☑ | D8 | blank | Unknown |
| ☑ | D9 | blank | Unknown |
| ☑ | D10 | blank | Unknown |
| ☑ | D11 | blank | Unknown |
| ☑ | D12 | blank | Unknown |
| ☑ | E4 | blank | Unknown |
| ☑ | E5 | blank | Unknown |
| ☑ | E6 | blank | Unknown |
| ☑ | E7 | blank | Unknown |
| ☑ | E8 | blank | Unknown |
| ☑ | E9 | blank | Unknown |
| ☑ | E10 | blank | Unknown |
| ☑ | E11 | blank | Unknown |
| ☑ | E12 | blank | Unknown |
| ☑ | F4 | blank | Unknown |
| ☑ | F5 | blank | Unknown |
| ☑ | F6 | blank | Unknown |
| ☑ | F7 | blank | Unknown |
| ☑ | F8 | blank | Unknown |
| ☑ | F9 | blank | Unknown |
| ☑ | F10 | blank | Unknown |
| ☑ | F11 | blank | Unknown |
| ☑ | F12 | blank | Unknown |
| ☑ | G1 | blank | Unknown |
| ☑ | G2 | blank | Unknown |
| ☑ | G3 | blank | Unknown |
| ☑ | G4 | blank | Unknown |
| ☑ | G5 | blank | Unknown |
| ☑ | G6 | blank | Unknown |
| ☑ | G7 | blank | Unknown |
| ☑ | G8 | blank | Unknown |

| Group | Negative |
|---|---|

| Inc | Pos | Sample Name | Sample Type |
|---|---|---|---|
| ☑ | G9 | blank | Unknown |
| ☑ | G10 | blank | Unknown |
| ☑ | G11 | blank | Unknown |
| ☑ | G12 | blank | Unknown |
| ☑ | H1 | blank | Unknown |
| ☑ | H2 | blank | Unknown |
| ☑ | H3 | blank | Unknown |
| ☑ | H4 | blank | Unknown |
| ☑ | H5 | blank | Unknown |
| ☑ | H6 | blank | Unknown |
| ☑ | H7 | blank | Unknown |
| ☑ | H8 | blank | Unknown |
| ☑ | H9 | blank | Unknown |
| ☑ | H10 | blank | Unknown |
| ☑ | H11 | blank | Unknown |
| ☑ | H12 | blank | Unknown |

| Group | Unknown |
|---|---|

| Inc | Pos | Sample Name | Sample Type |
|---|---|---|---|
| ☑ | A3 | GAPDH 9 | Unknown |
| ☑ | A7 | GAPDH 11 | Unknown |
| ☑ | C1 | RGS1 9 | Unknown |
| ☑ | C10 | RGS1 12 | Unknown |
| ☑ | C11 | RGS1 12 | Unknown |
| ☑ | D1 | GAPDH 13 | Unknown |
| ☑ | E2 | FCER1A 13 | Unknown |
| ☑ | F1 | RGS1 13 | Unknown |

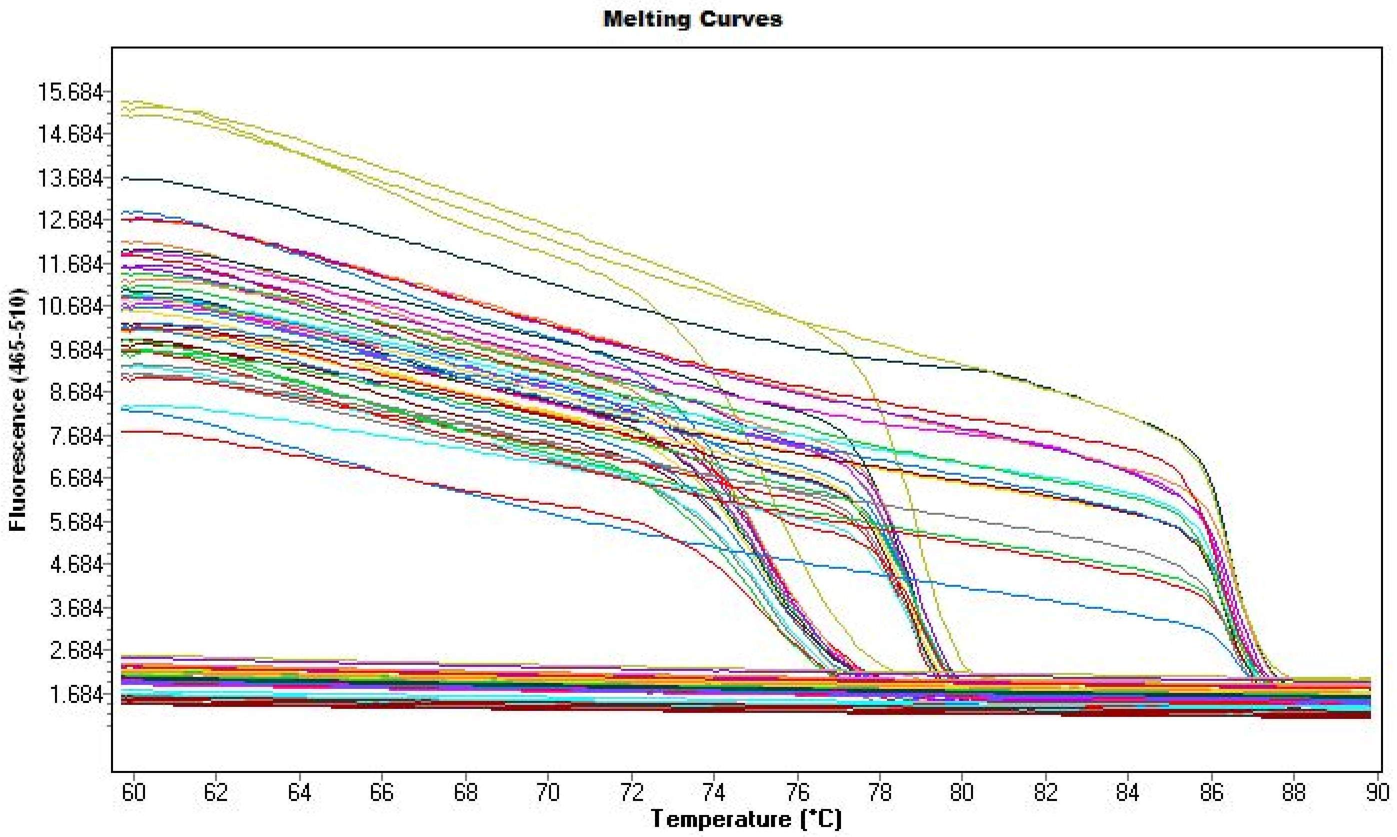

Melting Curves
15.684
14.684
13.684
12.684
11.684
10.684
9.684
8.684
7.684
6.684
5.684
4.684
3.684
2.684
1.684
Fluorescence (465-510)
60
62
64
66
68
70
72
74
76
78
80
82
84
86
88
90
Temperature (°C)


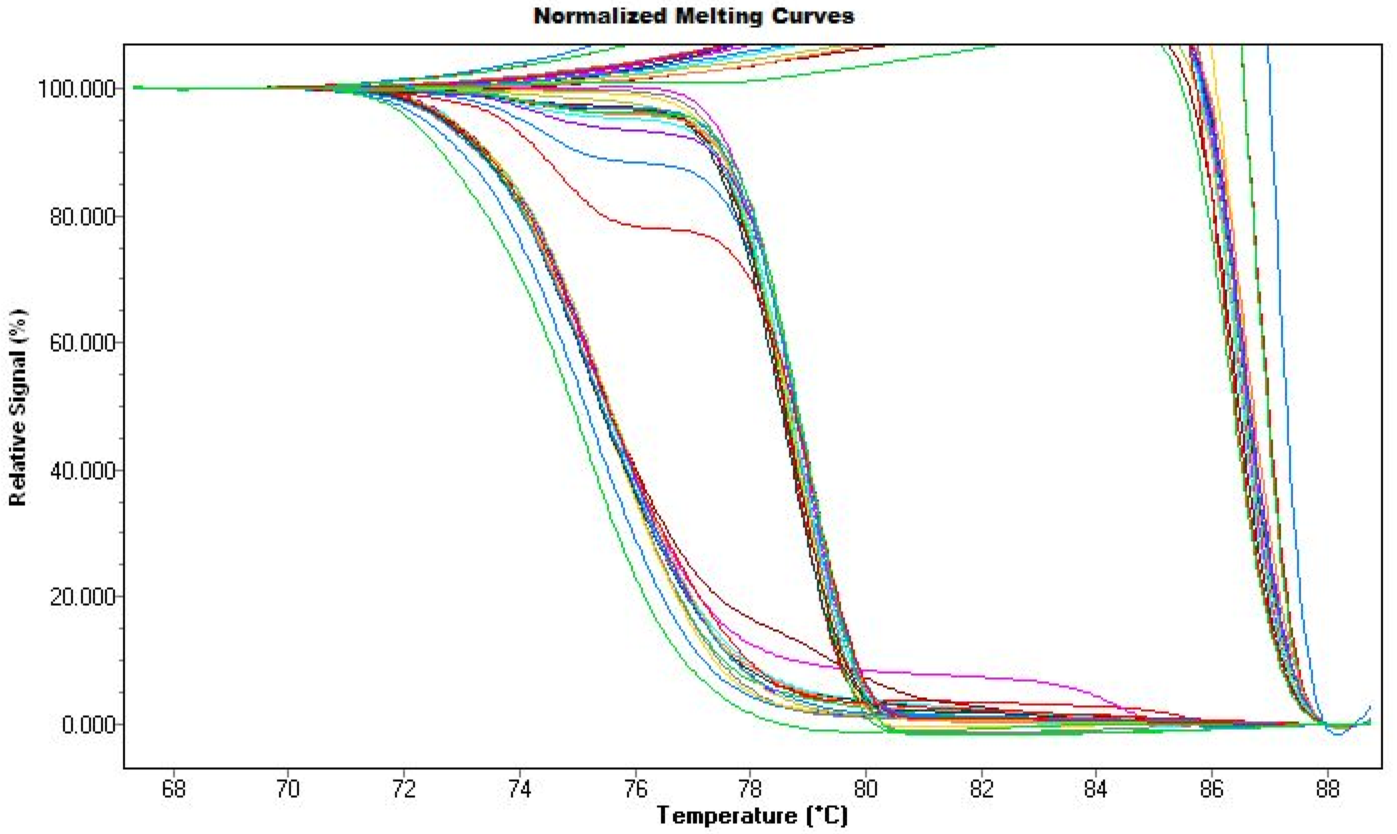

Normalized Melting Curves
100.000
80.000
60.000
40.000
20.000
0.000
Relative Signal (%)
68
70
72
74
76
78
80
82
84
86
88
Temperature (°C)

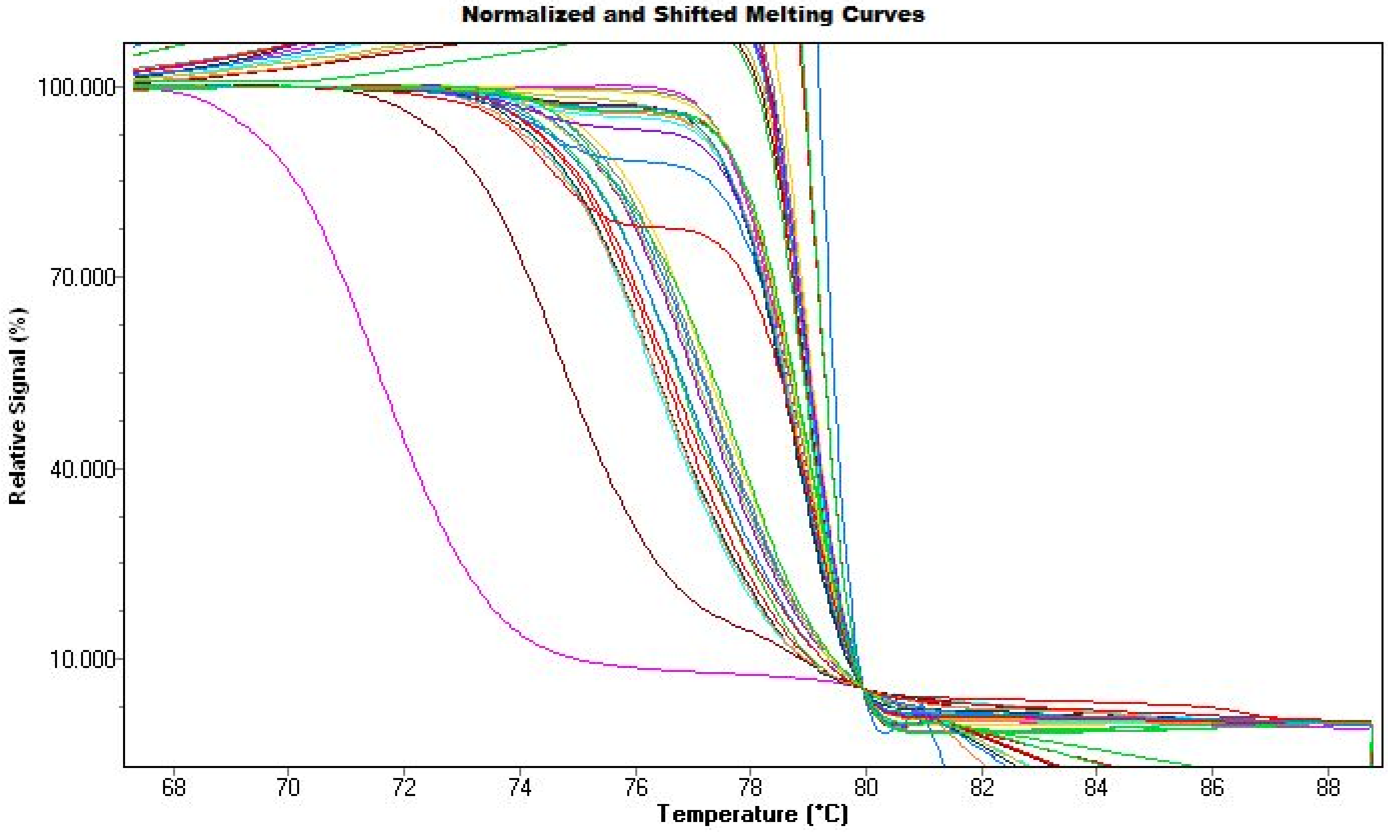


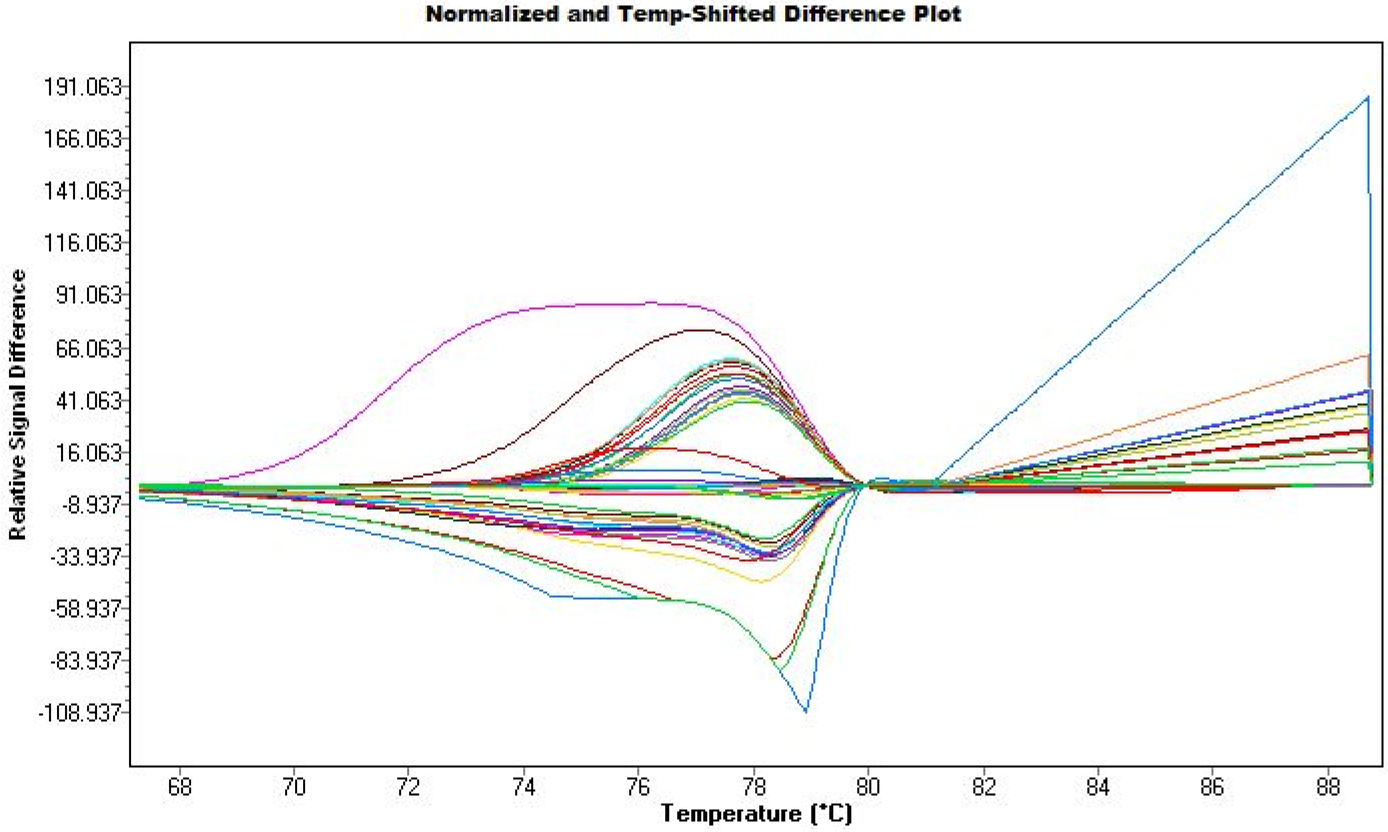


| Analysis Notes | |
|---|---|

## Melt Curve Genotyping for All Samples (Melt Curve Genotyping)

| Channel | 465-510 | Color Compensation | Off | | | | |
|---|---|---|---|---|---|---|---|
| Progam Name | Program | | | | | | |
| Stds Settings | Auto-Group | | | | | | |
| Sensitivity | Normal | Temp Range | 62-86 | Score | 0.7 | Res | 0.1 |

| Subset Name | All Samples |
|---|---|

**Results**

| Inc | Pos. | Sample Name | Sample Type | Group | Score | Resolution | Status |
|---|---|---|---|---|---|---|---|
| ☑ | A1 | GAPDH 9 | Unknown | 2 | 0.99 | 0.89 | |
| ☑ | A2 | GAPDH 9 | Unknown | 2 | 0.99 | 0.83 | |
| ☑ | A3 | GAPDH 9 | Unknown | 2 | 0.99 | 0.83 | |
| ☑ | A4 | GAPDH 10 | Unknown | 2 | 0.98 | 0.80 | |
| ☑ | A5 | GAPDH 10 | Unknown | 2 | 0.98 | 0.82 | |
| ☑ | A6 | GAPDH 10 | Unknown | 2 | 0.98 | 0.84 | |
| ☑ | A7 | GAPDH 11 | Unknown | 2 | 0.97 | 0.75 | |
| ☑ | A8 | GAPDH 11 | Unknown | 2 | 0.99 | 0.80 | |
| ☑ | A9 | GAPDH 11 | Unknown | 2 | 0.99 | 0.83 | |
| ☑ | A10 | GAPDH 12 | Unknown | 2 | 1.00 | 0.82 | |
| ☑ | A11 | GAPDH 12 | Unknown | 2 | 0.99 | 0.80 | |
| ☑ | A12 | GAPDH 12 | Unknown | 2 | 0.99 | 0.78 | |
| ☑ | B1 | FCER1A 9 | Unknown | 3 | 0.93 | 0.64 | |
| ☑ | B2 | FCER1A 9 | Unknown | 3 | 0.99 | 0.77 | |
| ☑ | B3 | FCER1A 9 | Unknown | 3 | 0.99 | 0.76 | |
| ☑ | B4 | FCER1A 10 | Unknown | 3 | 0.98 | 0.83 | |
| ☑ | B5 | FCER1A 10 | Unknown | 3 | 0.98 | 0.82 | |
| ☑ | B6 | FCER1A 10 | Unknown | 3 | 0.99 | 0.77 | |
| ☑ | B7 | FCER1A 11 | Unknown | 3 | 0.98 | 0.78 | |
| ☑ | B8 | FCER1A 11 | Unknown | 3 | 0.99 | 0.82 | |
| ☑ | B9 | FCER1A 11 | Unknown | 3 | 0.99 | 0.76 | |
| ☑ | B10 | FCER1A 12 | Unknown | 3 | 1.00 | 0.82 | |
| ☑ | B11 | FCER1A 12 | Unknown | 3 | 0.97 | 0.76 | |
| ☑ | B12 | FCER1A 12 | Unknown | 3 | 0.99 | 0.81 | |
| ☑ | C1 | RGS1 9 | Unknown | 1 | 0.90 | 0.84 | |
| ☑ | C2 | RGS1 9 | Unknown | 1 | 0.95 | 0.90 | |
| ☑ | C3 | RGS1 9 | Unknown | 1 | 0.95 | 0.89 | |
| ☑ | C4 | RGS1 10 | Unknown | 1 | 0.98 | 0.91 | |
| ☑ | C5 | RGS1 10 | Unknown | 1 | 0.99 | 0.92 | |
| ☑ | C6 | RGS1 10 | Unknown | 1 | 0.98 | 0.88 | |
| ☑ | C7 | RGS1 11 | Unknown | 1 | 0.99 | 0.91 | |

**Results**

| Inc | Pos. | Sample Name | Sample Type | Group | Score | Resolution | Status |
|---|---|---|---|---|---|---|---|
| ☑ | C8 | RGS1 11 | Unknown | 1 | 0.98 | 0.89 | |
| ☑ | C9 | RGS1 11 | Unknown | 1 | 0.96 | 0.90 | |
| ☑ | C10 | RGS1 12 | Unknown | 1 | 0.99 | 0.90 | |
| ☑ | C11 | RGS1 12 | Unknown | 1 | 0.99 | 0.93 | |
| ☑ | C12 | RGS1 12 | Unknown | 1 | 0.99 | 0.92 | |
| ☑ | D1 | GAPDH 13 | Unknown | 2 | 0.99 | 0.82 | |
| ☑ | D2 | GAPDH 13 | Unknown | 2 | 0.99 | 0.81 | |
| ☑ | D3 | GAPDH 13 | Unknown | 2 | 0.98 | 0.91 | |
| ☑ | D4 | blank | Unknown | Negative | 1.00 | 1.00 | |
| ☑ | D5 | blank | Unknown | Negative | 1.00 | 1.00 | |
| ☑ | D6 | blank | Unknown | Negative | 1.00 | 1.00 | |
| ☑ | D7 | blank | Unknown | Negative | 1.00 | 1.00 | |
| ☑ | D8 | blank | Unknown | Negative | 1.00 | 1.00 | |
| ☑ | D9 | blank | Unknown | Negative | 1.00 | 1.00 | |
| ☑ | D10 | blank | Unknown | Negative | 1.00 | 1.00 | |
| ☑ | D11 | blank | Unknown | Negative | 1.00 | 1.00 | |
| ☑ | D12 | blank | Unknown | Negative | 1.00 | 1.00 | |
| ☑ | E1 | FCER1A 13 | Unknown | 3 | 0.99 | 0.80 | |
| ☑ | E2 | FCER1A 13 | Unknown | Unknown | 0.66 | 0.20 | |
| ☑ | E3 | FCER1A 13 | Unknown | 3 | 0.99 | 0.79 | |
| ☑ | E4 | blank | Unknown | Negative | 1.00 | 1.00 | |
| ☑ | E5 | blank | Unknown | Negative | 1.00 | 1.00 | |
| ☑ | E6 | blank | Unknown | Negative | 1.00 | 1.00 | |
| ☑ | E7 | blank | Unknown | Negative | 1.00 | 1.00 | |
| ☑ | E8 | blank | Unknown | Negative | 1.00 | 1.00 | |
| ☑ | E9 | blank | Unknown | Negative | 1.00 | 1.00 | |
| ☑ | E10 | blank | Unknown | Negative | 1.00 | 1.00 | |
| ☑ | E11 | blank | Unknown | Negative | 1.00 | 1.00 | |
| ☑ | E12 | blank | Unknown | Negative | 1.00 | 1.00 | |
| ☑ | F1 | RGS1 13 | Unknown | 1 | 1.00 | 0.92 | |
| ☑ | F2 | RGS1 13 | Unknown | 1 | 0.97 | 0.90 | |
| ☑ | F3 | RGS1 13 | Unknown | 1 | 0.98 | 0.90 | |
| ☑ | F4 | blank | Unknown | Negative | 1.00 | 1.00 | |
| ☑ | F5 | blank | Unknown | Negative | 1.00 | 1.00 | |
| ☑ | F6 | blank | Unknown | Negative | 1.00 | 1.00 | |
| ☑ | F7 | blank | Unknown | Negative | 1.00 | 1.00 | |
| ☑ | F8 | blank | Unknown | Negative | 1.00 | 1.00 | |
| ☑ | F9 | blank | Unknown | Negative | 1.00 | 1.00 | |

**Results**

| Inc | Pos. | Sample Name | Sample Type | Group | Score | Resolution | Status |
|---|---|---|---|---|---|---|---|
| ☑ | F10 | blank | Unknown | Negative | 1.00 | 1.00 | |
| ☑ | F11 | blank | Unknown | Negative | 1.00 | 1.00 | |
| ☑ | F12 | blank | Unknown | Negative | 1.00 | 1.00 | |
| ☑ | G1 | blank | Unknown | Negative | 1.00 | 1.00 | |
| ☑ | G2 | blank | Unknown | Negative | 1.00 | 1.00 | |
| ☑ | G3 | blank | Unknown | Negative | 1.00 | 1.00 | |
| ☑ | G4 | blank | Unknown | Negative | 1.00 | 1.00 | |
| ☑ | G5 | blank | Unknown | Negative | 1.00 | 1.00 | |
| ☑ | G6 | blank | Unknown | Negative | 1.00 | 1.00 | |
| ☑ | G7 | blank | Unknown | Negative | 1.00 | 1.00 | |
| ☑ | G8 | blank | Unknown | Negative | 1.00 | 1.00 | |
| ☑ | G9 | blank | Unknown | Negative | 1.00 | 1.00 | |
| ☑ | G10 | blank | Unknown | Negative | 1.00 | 1.00 | |
| ☑ | G11 | blank | Unknown | Negative | 1.00 | 1.00 | |
| ☑ | G12 | blank | Unknown | Negative | 1.00 | 1.00 | |
| ☑ | H1 | blank | Unknown | Negative | 1.00 | 1.00 | |
| ☑ | H2 | blank | Unknown | Negative | 1.00 | 1.00 | |
| ☑ | H3 | blank | Unknown | Negative | 1.00 | 1.00 | |
| ☑ | H4 | blank | Unknown | Negative | 1.00 | 1.00 | |
| ☑ | H5 | blank | Unknown | Negative | 1.00 | 1.00 | |
| ☑ | H6 | blank | Unknown | Negative | 1.00 | 1.00 | |
| ☑ | H7 | blank | Unknown | Negative | 1.00 | 1.00 | |
| ☑ | H8 | blank | Unknown | Negative | 1.00 | 1.00 | |
| ☑ | H9 | blank | Unknown | Negative | 1.00 | 1.00 | |
| ☑ | H10 | blank | Unknown | Negative | 1.00 | 1.00 | |
| ☑ | H11 | blank | Unknown | Negative | 1.00 | 1.00 | |
| ☑ | H12 | blank | Unknown | Negative | 1.00 | 1.00 | |

| Group | 1 |
|---|---|

| Inc | Pos | Sample Name | Sample Type | Score | Resolution | Status |
|---|---|---|---|---|---|---|
| ☑ | C1 | RGS1 9 | Unknown | 0.90 | 0.84 | |
| ☑ | C2 | RGS1 9 | Unknown | 0.95 | 0.90 | |
| ☑ | C3 | RGS1 9 | Unknown | 0.95 | 0.89 | |
| ☑ | C4 | RGS1 10 | Unknown | 0.98 | 0.91 | |
| ☑ | C5 | RGS1 10 | Unknown | 0.99 | 0.92 | |
| ☑ | C6 | RGS1 10 | Unknown | 0.98 | 0.88 | |
| ☑ | C7 | RGS1 11 | Unknown | 0.99 | 0.91 | |
| ☑ | C8 | RGS1 11 | Unknown | 0.98 | 0.89 | |

| Group | 1 |
|---|---|

| Inc | Pos | Sample Name | Sample Type | Score | Resolution | Status |
|---|---|---|---|---|---|---|
| ☑ | C9 | RGS1 11 | Unknown | 0.96 | 0.90 | |
| ☑ | C10 | RGS1 12 | Unknown | 0.99 | 0.90 | |
| ☑ | C11 | RGS1 12 | Unknown | 0.99 | 0.93 | |
| ☑ | C12 | RGS1 12 | Unknown | 0.99 | 0.92 | |
| ☑ | F1 | RGS1 13 | Unknown | 1.00 | 0.92 | |
| ☑ | F2 | RGS1 13 | Unknown | 0.97 | 0.90 | |
| ☑ | F3 | RGS1 13 | Unknown | 0.98 | 0.90 | |

| Group | 2 |
|---|---|

| Inc | Pos | Sample Name | Sample Type | Score | Resolution | Status |
|---|---|---|---|---|---|---|
| ☑ | A1 | GAPDH 9 | Unknown | 0.99 | 0.89 | |
| ☑ | A2 | GAPDH 9 | Unknown | 0.99 | 0.83 | |
| ☑ | A3 | GAPDH 9 | Unknown | 0.99 | 0.83 | |
| ☑ | A4 | GAPDH 10 | Unknown | 0.98 | 0.80 | |
| ☑ | A5 | GAPDH 10 | Unknown | 0.98 | 0.82 | |
| ☑ | A6 | GAPDH 10 | Unknown | 0.98 | 0.84 | |
| ☑ | A7 | GAPDH 11 | Unknown | 0.97 | 0.75 | |
| ☑ | A8 | GAPDH 11 | Unknown | 0.99 | 0.80 | |
| ☑ | A9 | GAPDH 11 | Unknown | 0.99 | 0.83 | |
| ☑ | A10 | GAPDH 12 | Unknown | 1.00 | 0.82 | |
| ☑ | A11 | GAPDH 12 | Unknown | 0.99 | 0.80 | |
| ☑ | A12 | GAPDH 12 | Unknown | 0.99 | 0.78 | |
| ☑ | D1 | GAPDH 13 | Unknown | 0.99 | 0.82 | |
| ☑ | D2 | GAPDH 13 | Unknown | 0.99 | 0.81 | |
| ☑ | D3 | GAPDH 13 | Unknown | 0.98 | 0.91 | |

| Group | 3 |
|---|---|

| Inc | Pos | Sample Name | Sample Type | Score | Resolution | Status |
|---|---|---|---|---|---|---|
| ☑ | B1 | FCER1A 9 | Unknown | 0.93 | 0.64 | |
| ☑ | B2 | FCER1A 9 | Unknown | 0.99 | 0.77 | |
| ☑ | B3 | FCER1A 9 | Unknown | 0.99 | 0.76 | |
| ☑ | B4 | FCER1A 10 | Unknown | 0.98 | 0.83 | |
| ☑ | B5 | FCER1A 10 | Unknown | 0.98 | 0.82 | |
| ☑ | B6 | FCER1A 10 | Unknown | 0.99 | 0.77 | |
| ☑ | B7 | FCER1A 11 | Unknown | 0.98 | 0.78 | |
| ☑ | B8 | FCER1A 11 | Unknown | 0.99 | 0.82 | |
| ☑ | B9 | FCER1A 11 | Unknown | 0.99 | 0.76 | |
| ☑ | B10 | FCER1A 12 | Unknown | 1.00 | 0.82 | |

| Group | 3 |
|---|---|

| Inc | Pos | Sample Name | Sample Type | Score | Resolution | Status |
|---|---|---|---|---|---|---|
| ☑ | B11 | FCER1A 12 | Unknown | 0.97 | 0.76 | |
| ☑ | B12 | FCER1A 12 | Unknown | 0.99 | 0.81 | |
| ☑ | E1 | FCER1A 13 | Unknown | 0.99 | 0.80 | |
| ☑ | E3 | FCER1A 13 | Unknown | 0.99 | 0.79 | |

| Group | Negative |
|---|---|

| Inc | Pos | Sample Name | Sample Type | Score | Resolution | Status |
|---|---|---|---|---|---|---|
| ☑ | D4 | blank | Unknown | 1.00 | 1.00 | |
| ☑ | D5 | blank | Unknown | 1.00 | 1.00 | |
| ☑ | D6 | blank | Unknown | 1.00 | 1.00 | |
| ☑ | D7 | blank | Unknown | 1.00 | 1.00 | |
| ☑ | D8 | blank | Unknown | 1.00 | 1.00 | |
| ☑ | D9 | blank | Unknown | 1.00 | 1.00 | |
| ☑ | D10 | blank | Unknown | 1.00 | 1.00 | |
| ☑ | D11 | blank | Unknown | 1.00 | 1.00 | |
| ☑ | D12 | blank | Unknown | 1.00 | 1.00 | |
| ☑ | E4 | blank | Unknown | 1.00 | 1.00 | |
| ☑ | E5 | blank | Unknown | 1.00 | 1.00 | |
| ☑ | E6 | blank | Unknown | 1.00 | 1.00 | |
| ☑ | E7 | blank | Unknown | 1.00 | 1.00 | |
| ☑ | E8 | blank | Unknown | 1.00 | 1.00 | |
| ☑ | E9 | blank | Unknown | 1.00 | 1.00 | |
| ☑ | E10 | blank | Unknown | 1.00 | 1.00 | |
| ☑ | E11 | blank | Unknown | 1.00 | 1.00 | |
| ☑ | E12 | blank | Unknown | 1.00 | 1.00 | |
| ☑ | F4 | blank | Unknown | 1.00 | 1.00 | |
| ☑ | F5 | blank | Unknown | 1.00 | 1.00 | |
| ☑ | F6 | blank | Unknown | 1.00 | 1.00 | |
| ☑ | F7 | blank | Unknown | 1.00 | 1.00 | |
| ☑ | F8 | blank | Unknown | 1.00 | 1.00 | |
| ☑ | F9 | blank | Unknown | 1.00 | 1.00 | |
| ☑ | F10 | blank | Unknown | 1.00 | 1.00 | |
| ☑ | F11 | blank | Unknown | 1.00 | 1.00 | |
| ☑ | F12 | blank | Unknown | 1.00 | 1.00 | |
| ☑ | G1 | blank | Unknown | 1.00 | 1.00 | |
| ☑ | G2 | blank | Unknown | 1.00 | 1.00 | |
| ☑ | G3 | blank | Unknown | 1.00 | 1.00 | |
| ☑ | G4 | blank | Unknown | 1.00 | 1.00 | |

| Group | Negative |
|---|---|

| Inc | Pos | Sample Name | Sample Type | Score | Resolution | Status |
|---|---|---|---|---|---|---|
| ☑ | G5 | blank | Unknown | 1.00 | 1.00 | |
| ☑ | G6 | blank | Unknown | 1.00 | 1.00 | |
| ☑ | G7 | blank | Unknown | 1.00 | 1.00 | |
| ☑ | G8 | blank | Unknown | 1.00 | 1.00 | |
| ☑ | G9 | blank | Unknown | 1.00 | 1.00 | |
| ☑ | G10 | blank | Unknown | 1.00 | 1.00 | |
| ☑ | G11 | blank | Unknown | 1.00 | 1.00 | |
| ☑ | G12 | blank | Unknown | 1.00 | 1.00 | |
| ☑ | H1 | blank | Unknown | 1.00 | 1.00 | |
| ☑ | H2 | blank | Unknown | 1.00 | 1.00 | |
| ☑ | H3 | blank | Unknown | 1.00 | 1.00 | |
| ☑ | H4 | blank | Unknown | 1.00 | 1.00 | |
| ☑ | H5 | blank | Unknown | 1.00 | 1.00 | |
| ☑ | H6 | blank | Unknown | 1.00 | 1.00 | |
| ☑ | H7 | blank | Unknown | 1.00 | 1.00 | |
| ☑ | H8 | blank | Unknown | 1.00 | 1.00 | |
| ☑ | H9 | blank | Unknown | 1.00 | 1.00 | |
| ☑ | H10 | blank | Unknown | 1.00 | 1.00 | |
| ☑ | H11 | blank | Unknown | 1.00 | 1.00 | |
| ☑ | H12 | blank | Unknown | 1.00 | 1.00 | |

| Group | Unknown |
|---|---|

| Inc | Pos | Sample Name | Sample Type | Score | Resolution | Status |
|---|---|---|---|---|---|---|
| ☑ | E2 | FCER1A 13 | Unknown | 0.66 | 0.20 | |

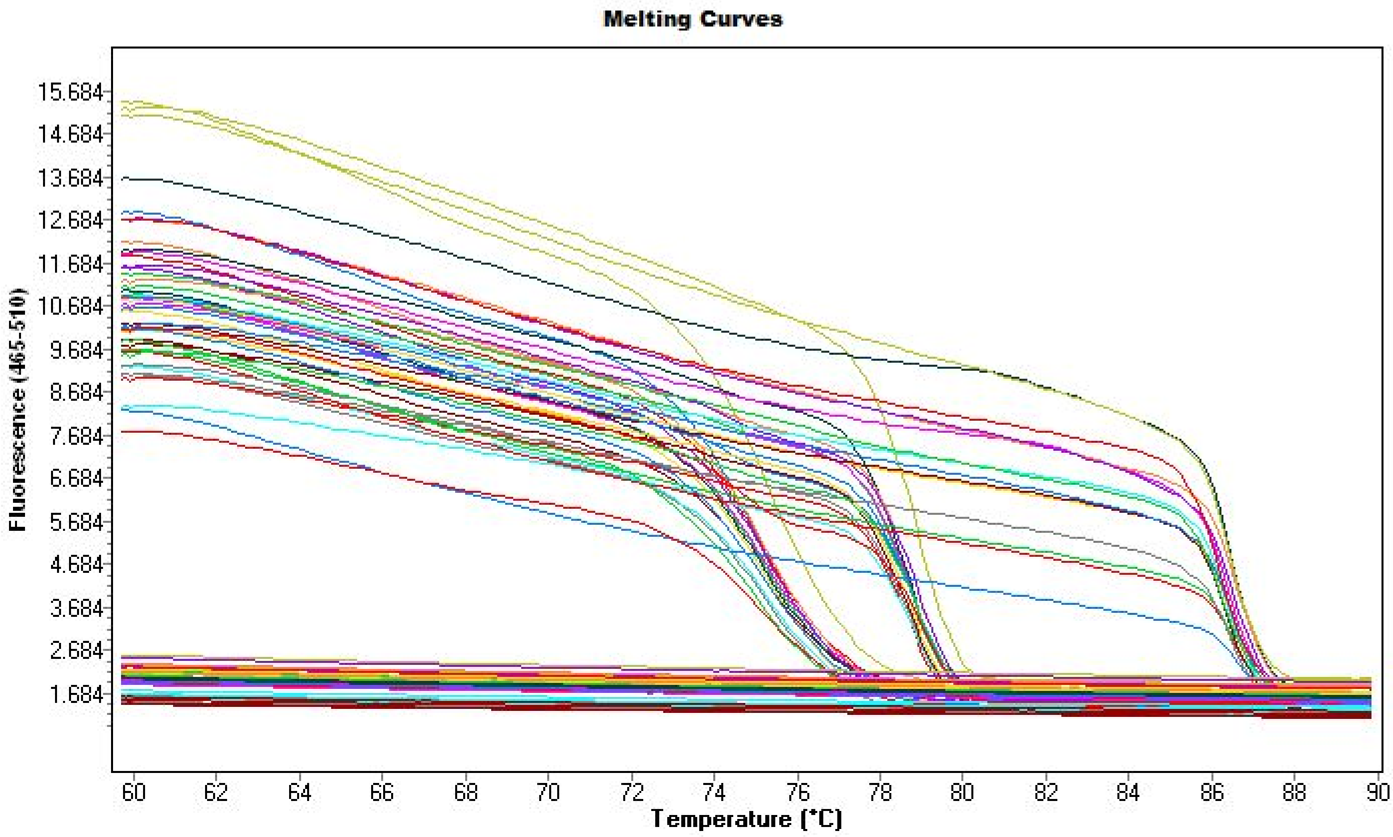


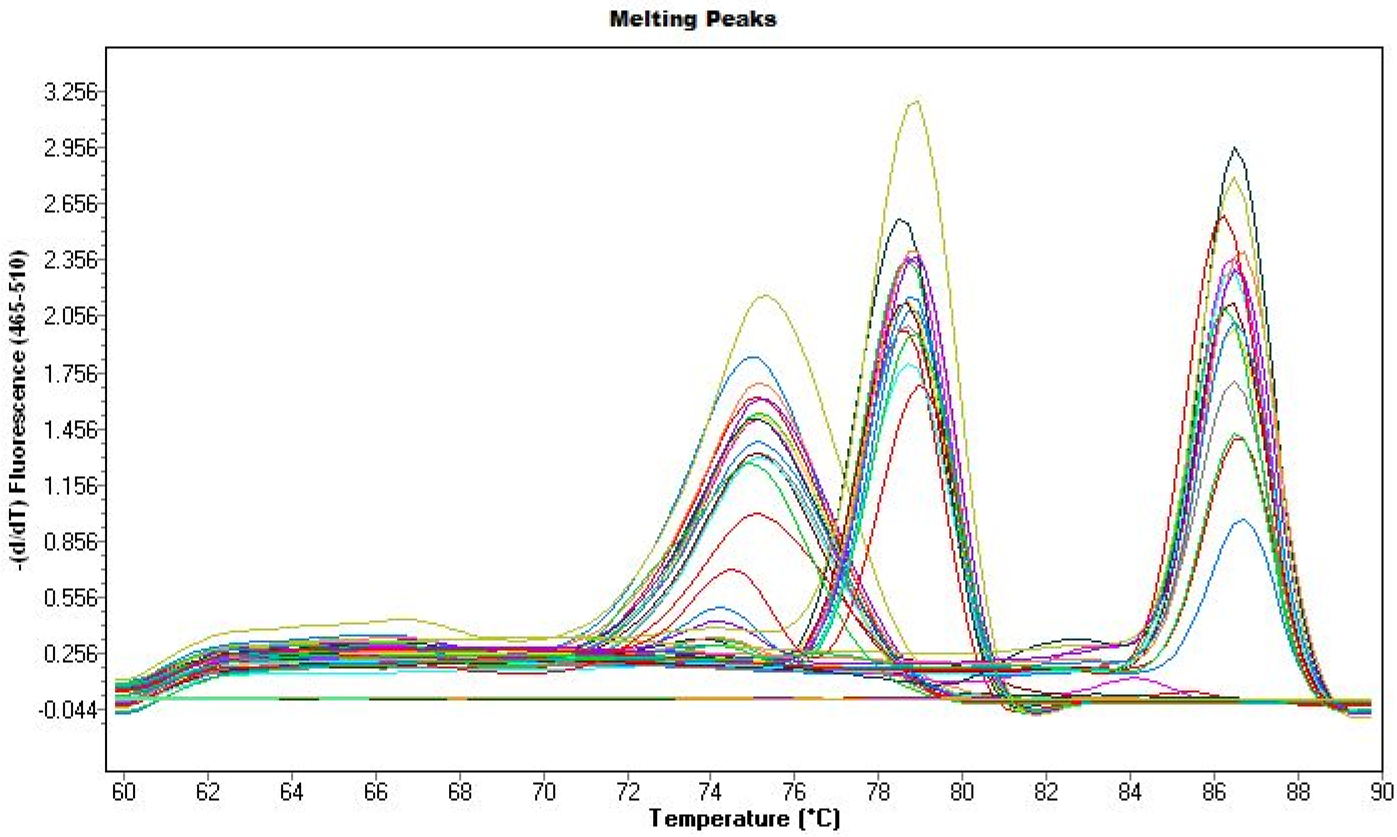


| **Analysis Notes** | |
|---|---|

## Tm Calling for All Samples (Tm Calling)

### Settings

| | | | | | |
|---|---|---|---|---|---|
| Channel | 465-510 | Color Compensation | Off | | |
| Program | Program | | | | |
| Show Shoulders | False | Chemistry | HybProbe | Max Peaks | 2 |

| | |
|---|---|
| Subset Name | All Samples |

### Results

| | | | Peak 1 | | | | Peak 2 | | | | |
|---|---|---|---|---|---|---|---|---|---|---|---|
| Inc | Pos | Sample Name | Tm | Area | Width | Height | Tm | Area | Width | Height | Status |
| ☑ | A1 | GAPDH 9 | 75.17 | 7.40 | 3.94 | 1.88 | | | | | |
| ☑ | A2 | GAPDH 9 | 75.39 | 6.52 | 3.97 | 1.65 | | | | | |
| ☑ | A3 | GAPDH 9 | 75.37 | 5.87 | 3.80 | 1.54 | | | | | |
| ☑ | A4 | GAPDH 10 | 75.36 | 5.69 | 3.81 | 1.49 | | | | | |
| ☑ | A5 | GAPDH 10 | 75.43 | 5.27 | 3.90 | 1.35 | | | | | |
| ☑ | A6 | GAPDH 10 | 75.41 | 5.96 | 3.82 | 1.56 | | | | | |
| ☑ | A7 | GAPDH 11 | 75.35 | 5.08 | 4.03 | 1.26 | | | | | |
| ☑ | A8 | GAPDH 11 | 75.41 | 5.03 | 3.91 | 1.29 | | | | | |
| ☑ | A9 | GAPDH 11 | 75.32 | 5.97 | 3.99 | 1.50 | | | | | |
| ☑ | A10 | GAPDH 12 | 75.39 | 6.83 | 4.04 | 1.69 | | | | | |
| ☑ | A11 | GAPDH 12 | 75.52 | 6.60 | 4.06 | 1.63 | | | | | |
| ☑ | A12 | GAPDH 12 | 75.58 | 8.89 | 4.05 | 2.19 | | | | | |
| ☑ | B1 | FCER1A 9 | 74.05 | 0.65 | 2.03 | 0.32 | 78.83 | 5.19 | 2.38 | 2.18 | |
| ☑ | B2 | FCER1A 9 | 78.60 | 4.73 | 2.30 | 2.05 | | | | | |
| ☑ | B3 | FCER1A 9 | 78.69 | 5.81 | 2.37 | 2.45 | | | | | |
| ☑ | B4 | FCER1A 10 | 78.76 | 5.90 | 2.41 | 2.45 | | | | | |
| ☑ | B5 | FCER1A 10 | 78.70 | 5.12 | 2.45 | 2.09 | | | | | |
| ☑ | B6 | FCER1A 10 | 78.67 | 5.32 | 2.40 | 2.21 | | | | | |
| ☑ | B7 | FCER1A 11 | 78.62 | 5.38 | 2.45 | 2.19 | | | | | |
| ☑ | B8 | FCER1A 11 | 78.73 | 4.52 | 2.42 | 1.87 | | | | | |
| ☑ | B9 | FCER1A 11 | 78.55 | 6.43 | 2.41 | 2.67 | | | | | |
| ☑ | B10 | FCER1A 12 | 78.82 | 6.19 | 2.47 | 2.51 | | | | | |
| ☑ | B11 | FCER1A 12 | 78.88 | 5.92 | 2.39 | 2.47 | | | | | |
| ☑ | B12 | FCER1A 12 | 78.84 | 8.40 | 2.53 | 3.33 | | | | | |
| ☑ | C1 | RGS1 9 | 68.32 | 0.63 | 5.00 | 0.13 | 86.64 | 2.43 | 3.22 | 0.76 | |
| ☑ | C2 | RGS1 9 | 86.72 | 3.69 | 2.50 | 1.48 | | | | | |
| ☑ | C3 | RGS1 9 | 86.72 | 3.72 | 2.52 | 1.48 | | | | | |
| ☑ | C4 | RGS1 10 | 86.44 | 5.75 | 2.25 | 2.56 | | | | | |
| ☑ | C5 | RGS1 10 | 86.55 | 4.37 | 2.42 | 1.80 | | | | | |

**Results**

| Inc | Pos | Sample Name | Peak 1 Tm | Peak 1 Area | Peak 1 Width | Peak 1 Height | Peak 2 Tm | Peak 2 Area | Peak 2 Width | Peak 2 Height | Status |
|---|---|---|---|---|---|---|---|---|---|---|---|
| ☑ | C6 | RGS1 10 | 86.53 | 4.97 | 2.38 | 2.09 | | | | | |
| ☑ | C7 | RGS1 11 | 86.28 | 4.29 | 2.16 | 1.99 | | | | | |
| ☑ | C8 | RGS1 11 | 86.46 | 5.11 | 2.20 | 2.32 | | | | | |
| ☑ | C9 | RGS1 11 | 86.58 | 7.53 | 2.42 | 3.11 | | | | | |
| ☑ | C10 | RGS1 12 | 86.72 | 5.67 | 2.26 | 2.51 | | | | | |
| ☑ | C11 | RGS1 12 | 86.63 | 6.05 | 2.48 | 2.44 | | | | | |
| ☑ | C12 | RGS1 12 | 86.57 | 6.92 | 2.36 | 2.93 | | | | | |
| ☑ | D1 | GAPDH 13 | 75.44 | 5.77 | 4.14 | 1.39 | | | | | |
| ☑ | D2 | GAPDH 13 | 75.49 | 4.22 | 4.19 | 1.01 | | | | | |
| ☑ | D3 | GAPDH 13 | 74.86 | 5.71 | 4.44 | 1.29 | | | | | |
| ☑ | D4 | blank | | | | | | | | | |
| ☑ | D5 | blank | | | | | | | | | |
| ☑ | D6 | blank | | | | | | | | | |
| ☑ | D7 | blank | | | | | | | | | |
| ☑ | D8 | blank | | | | | | | | | |
| ☑ | D9 | blank | | | | | | | | | |
| ☑ | D10 | blank | | | | | | | | | |
| ☑ | D11 | blank | | | | | | | | | |
| ☑ | D12 | blank | | | | | | | | | |
| ☑ | E1 | FCER1A 13 | 78.77 | 5.58 | 2.47 | 2.26 | | | | | |
| ☑ | E2 | FCER1A 13 | 74.39 | 1.20 | 2.20 | 0.55 | 79.03 | 4.14 | 2.35 | 1.76 | |
| ☑ | E3 | FCER1A 13 | 78.85 | 5.00 | 2.45 | 2.04 | | | | | |
| ☑ | E4 | blank | | | | | | | | | |
| ☑ | E5 | blank | | | | | | | | | |
| ☑ | E6 | blank | | | | | | | | | |
| ☑ | E7 | blank | | | | | | | | | |
| ☑ | E8 | blank | | | | | | | | | |
| ☑ | E9 | blank | | | | | | | | | |
| ☑ | E10 | blank | | | | | | | | | |
| ☑ | E11 | blank | | | | | | | | | |
| ☑ | E12 | blank | | | | | | | | | |
| ☑ | F1 | RGS1 13 | 86.59 | 5.06 | 2.39 | 2.12 | | | | | |
| ☑ | F2 | RGS1 13 | 86.28 | 6.18 | 2.25 | 2.75 | | | | | |
| ☑ | F3 | RGS1 13 | 86.36 | 5.02 | 2.32 | 2.17 | | | | | |
| ☑ | F4 | blank | | | | | | | | | |
| ☑ | F5 | blank | | | | | | | | | |

## Results

| Inc | Pos | Sample Name | Peak 1 | | | | Peak 2 | | | | Status |
|---|---|---|---|---|---|---|---|---|---|---|---|
| | | | Tm | Area | Width | Height | Tm | Area | Width | Height | |
| ☑ | F6 | blank | | | | | | | | | |
| ☑ | F7 | blank | | | | | | | | | |
| ☑ | F8 | blank | | | | | | | | | |
| ☑ | F9 | blank | | | | | | | | | |
| ☑ | F10 | blank | | | | | | | | | |
| ☑ | F11 | blank | | | | | | | | | |
| ☑ | F12 | blank | | | | | | | | | |
| ☑ | G1 | blank | | | | | | | | | |
| ☑ | G2 | blank | | | | | | | | | |
| ☑ | G3 | blank | | | | | | | | | |
| ☑ | G4 | blank | | | | | | | | | |
| ☑ | G5 | blank | | | | | | | | | |
| ☑ | G6 | blank | | | | | | | | | |
| ☑ | G7 | blank | | | | | | | | | |
| ☑ | G8 | blank | | | | | | | | | |
| ☑ | G9 | blank | | | | | | | | | |
| ☑ | G10 | blank | | | | | | | | | |
| ☑ | G11 | blank | | | | | | | | | |
| ☑ | G12 | blank | | | | | | | | | |
| ☑ | H1 | blank | | | | | | | | | |
| ☑ | H2 | blank | | | | | | | | | |
| ☑ | H3 | blank | | | | | | | | | |
| ☑ | H4 | blank | | | | | | | | | |
| ☑ | H5 | blank | | | | | | | | | |
| ☑ | H6 | blank | | | | | | | | | |
| ☑ | H7 | blank | | | | | | | | | |
| ☑ | H8 | blank | | | | | | | | | |
| ☑ | H9 | blank | | | | | | | | | |
| ☑ | H10 | blank | | | | | | | | | |
| ☑ | H11 | blank | | | | | | | | | |
| ☑ | H12 | blank | | | | | | | | | |

## Melting Curves

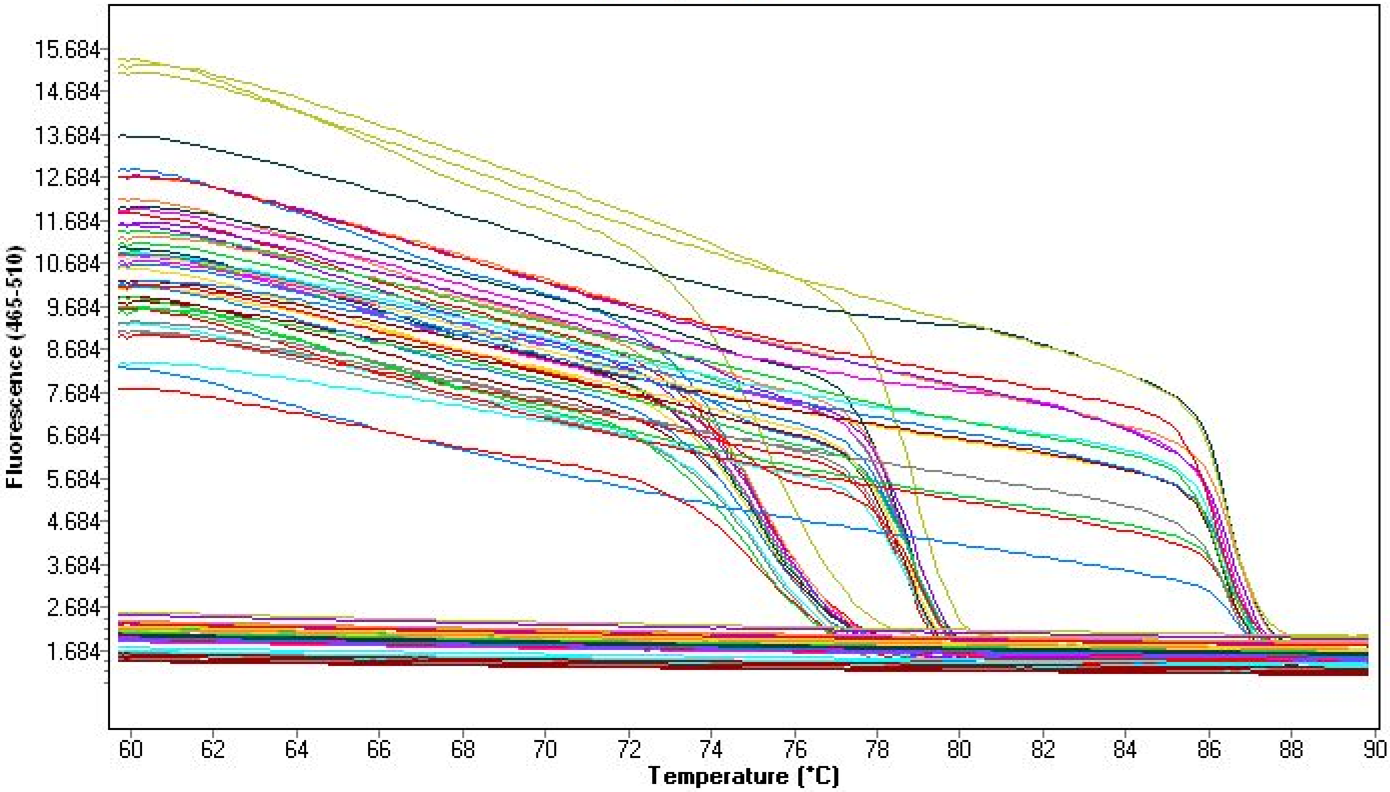


## Melting Peaks

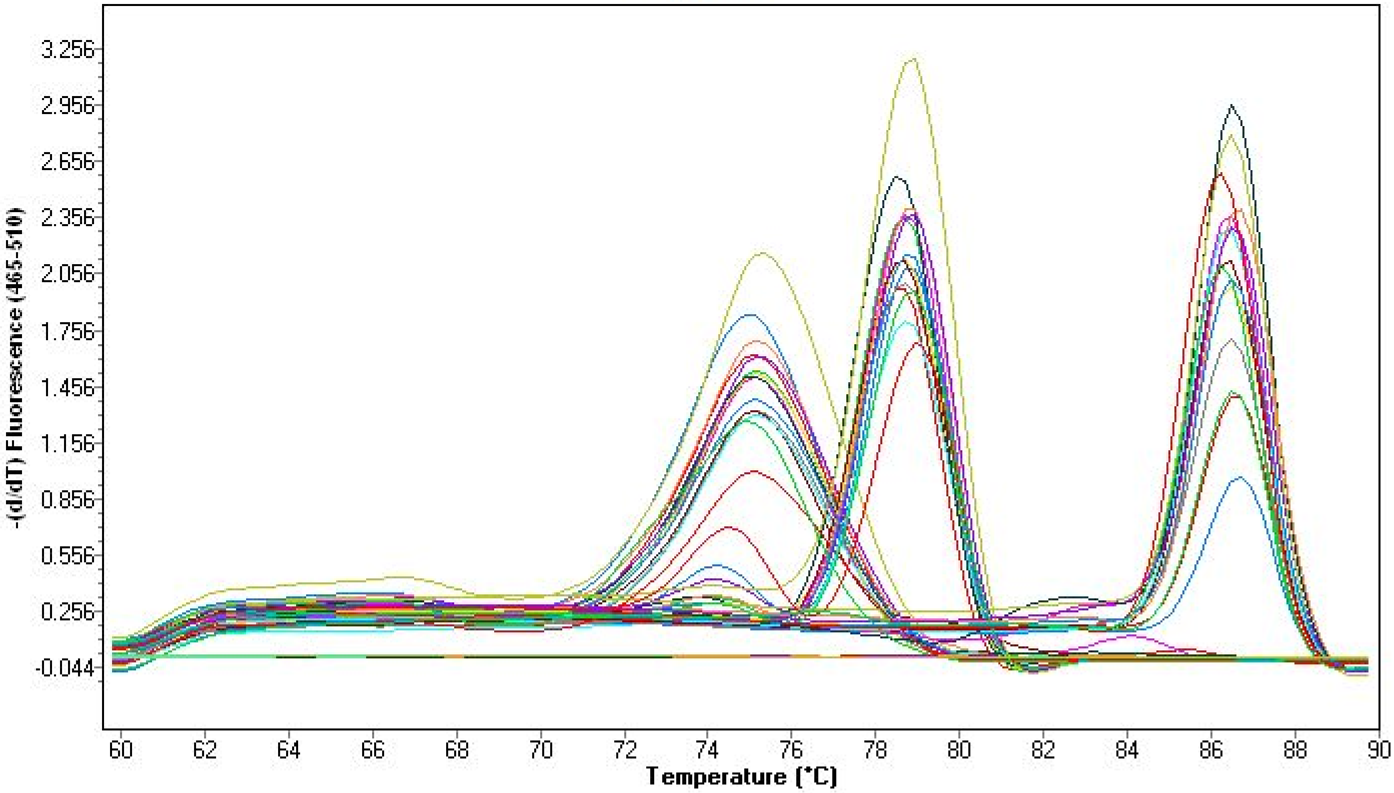


| Analysis Notes | |
|---|---|

## Tm Calling for All Samples (1) (Tm Calling)

### Settings

| | | | | | |
|---|---|---|---|---|---|
| Channel | 465-510 | Color Compensation | Off | | |
| Program | Program | | | | |
| Show Shoulders | False | Chemistry | HybProbe | Max Peaks | 2 |

| | |
|---|---|
| Subset Name | All Samples |

### Results

| | | | Peak 1 | | | | Peak 2 | | | | |
|---|---|---|---|---|---|---|---|---|---|---|---|
| Inc | Pos | Sample Name | Tm | Area | Width | Height | Tm | Area | Width | Height | Status |
| ☑ | A1 | GAPDH 9 | | | | | | | | | |
| ☑ | A2 | GAPDH 9 | | | | | | | | | |
| ☑ | A3 | GAPDH 9 | | | | | | | | | |
| ☑ | A4 | GAPDH 10 | | | | | | | | | |
| ☑ | A5 | GAPDH 10 | | | | | | | | | |
| ☑ | A6 | GAPDH 10 | | | | | | | | | |
| ☑ | A7 | GAPDH 11 | | | | | | | | | |
| ☑ | A8 | GAPDH 11 | | | | | | | | | |
| ☑ | A9 | GAPDH 11 | | | | | | | | | |
| ☑ | A10 | GAPDH 12 | | | | | | | | | |
| ☑ | A11 | GAPDH 12 | | | | | | | | | |
| ☑ | A12 | GAPDH 12 | | | | | | | | | |
| ☑ | B1 | FCER1A 9 | | | | | | | | | |
| ☑ | B2 | FCER1A 9 | | | | | | | | | |
| ☑ | B3 | FCER1A 9 | | | | | | | | | |
| ☑ | B4 | FCER1A 10 | | | | | | | | | |
| ☑ | B5 | FCER1A 10 | | | | | | | | | |
| ☑ | B6 | FCER1A 10 | | | | | | | | | |
| ☑ | B7 | FCER1A 11 | | | | | | | | | |
| ☑ | B8 | FCER1A 11 | | | | | | | | | |
| ☑ | B9 | FCER1A 11 | | | | | | | | | |
| ☑ | B10 | FCER1A 12 | | | | | | | | | |
| ☑ | B11 | FCER1A 12 | | | | | | | | | |
| ☑ | B12 | FCER1A 12 | | | | | | | | | |
| ☑ | C1 | RGS1 9 | | | | | | | | | |
| ☑ | C2 | RGS1 9 | | | | | | | | | |
| ☑ | C3 | RGS1 9 | | | | | | | | | |
| ☑ | C4 | RGS1 10 | | | | | | | | | |
| ☑ | C5 | RGS1 10 | | | | | | | | | |

**Results**

| Inc | Pos | Sample Name | Peak 1 Tm | Peak 1 Area | Peak 1 Width | Peak 1 Height | Peak 2 Tm | Peak 2 Area | Peak 2 Width | Peak 2 Height | Status |
|---|---|---|---|---|---|---|---|---|---|---|---|
| ☑ | C6 | RGS1 10 | | | | | | | | | |
| ☑ | C7 | RGS1 11 | | | | | | | | | |
| ☑ | C8 | RGS1 11 | | | | | | | | | |
| ☑ | C9 | RGS1 11 | | | | | | | | | |
| ☑ | C10 | RGS1 12 | | | | | | | | | |
| ☑ | C11 | RGS1 12 | | | | | | | | | |
| ☑ | C12 | RGS1 12 | | | | | | | | | |
| ☑ | D1 | GAPDH 13 | | | | | | | | | |
| ☑ | D2 | GAPDH 13 | | | | | | | | | |
| ☑ | D3 | GAPDH 13 | | | | | | | | | |
| ☑ | D4 | blank | | | | | | | | | |
| ☑ | D5 | blank | | | | | | | | | |
| ☑ | D6 | blank | | | | | | | | | |
| ☑ | D7 | blank | | | | | | | | | |
| ☑ | D8 | blank | | | | | | | | | |
| ☑ | D9 | blank | | | | | | | | | |
| ☑ | D10 | blank | | | | | | | | | |
| ☑ | D11 | blank | | | | | | | | | |
| ☑ | D12 | blank | | | | | | | | | |
| ☑ | E1 | FCER1A 13 | | | | | | | | | |
| ☑ | E2 | FCER1A 13 | | | | | | | | | |
| ☑ | E3 | FCER1A 13 | | | | | | | | | |
| ☑ | E4 | blank | | | | | | | | | |
| ☑ | E5 | blank | | | | | | | | | |
| ☑ | E6 | blank | | | | | | | | | |
| ☑ | E7 | blank | | | | | | | | | |
| ☑ | E8 | blank | | | | | | | | | |
| ☑ | E9 | blank | | | | | | | | | |
| ☑ | E10 | blank | | | | | | | | | |
| ☑ | E11 | blank | | | | | | | | | |
| ☑ | E12 | blank | | | | | | | | | |
| ☑ | F1 | RGS1 13 | | | | | | | | | |
| ☑ | F2 | RGS1 13 | | | | | | | | | |
| ☑ | F3 | RGS1 13 | | | | | | | | | |
| ☑ | F4 | blank | | | | | | | | | |
| ☑ | F5 | blank | | | | | | | | | |

**Results**

| Inc | Pos | Sample Name | Peak 1 | | | | Peak 2 | | | | Status |
|---|---|---|---|---|---|---|---|---|---|---|---|
| | | | Tm | Area | Width | Height | Tm | Area | Width | Height | |
| ☑ | F6 | blank | | | | | | | | | |
| ☑ | F7 | blank | | | | | | | | | |
| ☑ | F8 | blank | | | | | | | | | |
| ☑ | F9 | blank | | | | | | | | | |
| ☑ | F10 | blank | | | | | | | | | |
| ☑ | F11 | blank | | | | | | | | | |
| ☑ | F12 | blank | | | | | | | | | |
| ☑ | G1 | blank | | | | | | | | | |
| ☑ | G2 | blank | | | | | | | | | |
| ☑ | G3 | blank | | | | | | | | | |
| ☑ | G4 | blank | | | | | | | | | |
| ☑ | G5 | blank | | | | | | | | | |
| ☑ | G6 | blank | | | | | | | | | |
| ☑ | G7 | blank | | | | | | | | | |
| ☑ | G8 | blank | | | | | | | | | |
| ☑ | G9 | blank | | | | | | | | | |
| ☑ | G10 | blank | | | | | | | | | |
| ☑ | G11 | blank | | | | | | | | | |
| ☑ | G12 | blank | | | | | | | | | |
| ☑ | H1 | blank | | | | | | | | | |
| ☑ | H2 | blank | | | | | | | | | |
| ☑ | H3 | blank | | | | | | | | | |
| ☑ | H4 | blank | | | | | | | | | |
| ☑ | H5 | blank | | | | | | | | | |
| ☑ | H6 | blank | | | | | | | | | |
| ☑ | H7 | blank | | | | | | | | | |
| ☑ | H8 | blank | | | | | | | | | |
| ☑ | H9 | blank | | | | | | | | | |
| ☑ | H10 | blank | | | | | | | | | |
| ☑ | H11 | blank | | | | | | | | | |
| ☑ | H12 | blank | | | | | | | | | |

Melting Curves

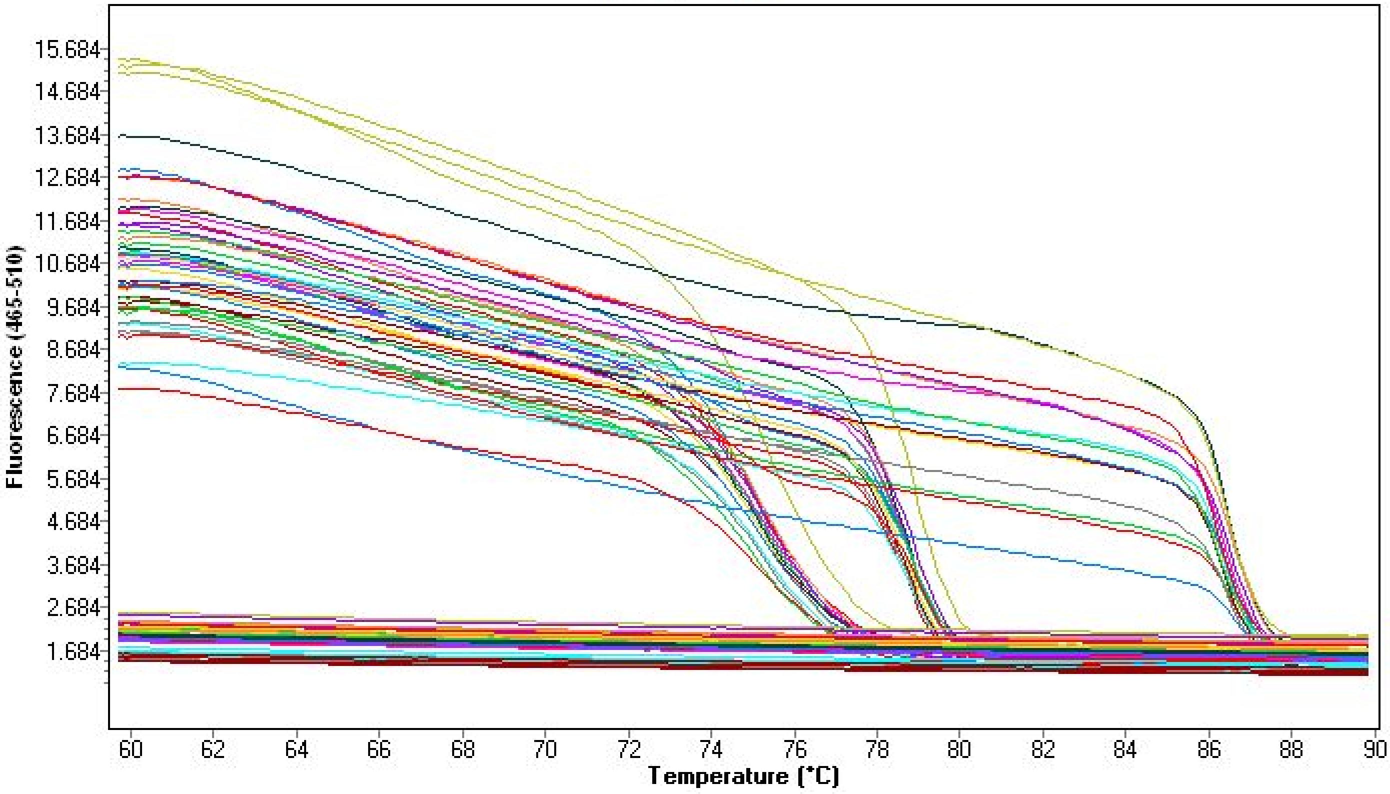


Melting Peaks

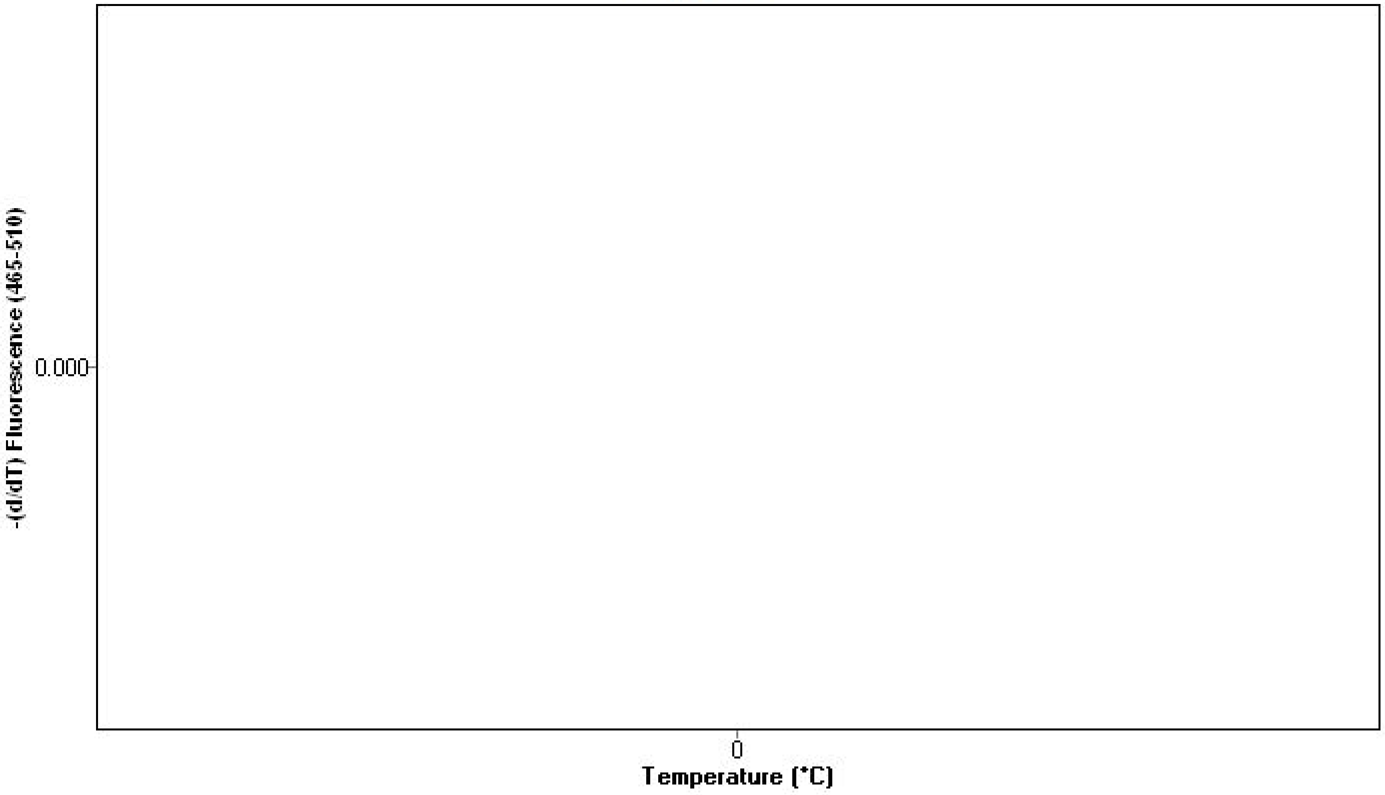


| Analysis Notes | |
|---|---|

## Melt Curve Genotyping for All Samples (1) (Melt Curve Genotyping)

| Channel | 465-510 | Color Compensation | Off | | | | |
|---|---|---|---|---|---|---|---|
| Progam Name | Program | | | | | | |
| Stds Settings | Auto-Group | | | | | | |
| Sensitivity | Normal | Temp Range | 62-86 | Score | 0.7 | Res | 0.1 |

| Subset Name | All Samples |
|---|---|

**Results**

| Inc | Pos. | Sample Name | Sample Type | Group | Score | Resolution | Status |
|---|---|---|---|---|---|---|---|
| ☑ | A1 | GAPDH 9 | Unknown | 2 | 0.99 | 0.89 | |
| ☑ | A2 | GAPDH 9 | Unknown | 2 | 0.99 | 0.83 | |
| ☑ | A3 | GAPDH 9 | Unknown | 2 | 0.99 | 0.83 | |
| ☑ | A4 | GAPDH 10 | Unknown | 2 | 0.98 | 0.80 | |
| ☑ | A5 | GAPDH 10 | Unknown | 2 | 0.98 | 0.82 | |
| ☑ | A6 | GAPDH 10 | Unknown | 2 | 0.98 | 0.84 | |
| ☑ | A7 | GAPDH 11 | Unknown | 2 | 0.97 | 0.75 | |
| ☑ | A8 | GAPDH 11 | Unknown | 2 | 0.99 | 0.80 | |
| ☑ | A9 | GAPDH 11 | Unknown | 2 | 0.99 | 0.83 | |
| ☑ | A10 | GAPDH 12 | Unknown | 2 | 1.00 | 0.82 | |
| ☑ | A11 | GAPDH 12 | Unknown | 2 | 0.99 | 0.80 | |
| ☑ | A12 | GAPDH 12 | Unknown | 2 | 0.99 | 0.78 | |
| ☑ | B1 | FCER1A 9 | Unknown | 3 | 0.93 | 0.64 | |
| ☑ | B2 | FCER1A 9 | Unknown | 3 | 0.99 | 0.77 | |
| ☑ | B3 | FCER1A 9 | Unknown | 3 | 0.99 | 0.76 | |
| ☑ | B4 | FCER1A 10 | Unknown | 3 | 0.98 | 0.83 | |
| ☑ | B5 | FCER1A 10 | Unknown | 3 | 0.98 | 0.82 | |
| ☑ | B6 | FCER1A 10 | Unknown | 3 | 0.99 | 0.77 | |
| ☑ | B7 | FCER1A 11 | Unknown | 3 | 0.98 | 0.78 | |
| ☑ | B8 | FCER1A 11 | Unknown | 3 | 0.99 | 0.82 | |
| ☑ | B9 | FCER1A 11 | Unknown | 3 | 0.99 | 0.76 | |
| ☑ | B10 | FCER1A 12 | Unknown | 3 | 1.00 | 0.82 | |
| ☑ | B11 | FCER1A 12 | Unknown | 3 | 0.97 | 0.76 | |
| ☑ | B12 | FCER1A 12 | Unknown | 3 | 0.99 | 0.81 | |
| ☑ | C1 | RGS1 9 | Unknown | 1 | 0.90 | 0.84 | |
| ☑ | C2 | RGS1 9 | Unknown | 1 | 0.95 | 0.90 | |
| ☑ | C3 | RGS1 9 | Unknown | 1 | 0.95 | 0.89 | |
| ☑ | C4 | RGS1 10 | Unknown | 1 | 0.98 | 0.91 | |
| ☑ | C5 | RGS1 10 | Unknown | 1 | 0.99 | 0.92 | |
| ☑ | C6 | RGS1 10 | Unknown | 1 | 0.98 | 0.88 | |
| ☑ | C7 | RGS1 11 | Unknown | 1 | 0.99 | 0.91 | |

**Results**

| Inc | Pos. | Sample Name | Sample Type | Group | Score | Resolution | Status |
|---|---|---|---|---|---|---|---|
| ☑ | C8 | RGS1 11 | Unknown | 1 | 0.98 | 0.89 | |
| ☑ | C9 | RGS1 11 | Unknown | 1 | 0.96 | 0.90 | |
| ☑ | C10 | RGS1 12 | Unknown | 1 | 0.99 | 0.90 | |
| ☑ | C11 | RGS1 12 | Unknown | 1 | 0.99 | 0.93 | |
| ☑ | C12 | RGS1 12 | Unknown | 1 | 0.99 | 0.92 | |
| ☑ | D1 | GAPDH 13 | Unknown | 2 | 0.99 | 0.82 | |
| ☑ | D2 | GAPDH 13 | Unknown | 2 | 0.99 | 0.81 | |
| ☑ | D3 | GAPDH 13 | Unknown | 2 | 0.98 | 0.91 | |
| ☑ | D4 | blank | Unknown | Negative | 1.00 | 1.00 | |
| ☑ | D5 | blank | Unknown | Negative | 1.00 | 1.00 | |
| ☑ | D6 | blank | Unknown | Negative | 1.00 | 1.00 | |
| ☑ | D7 | blank | Unknown | Negative | 1.00 | 1.00 | |
| ☑ | D8 | blank | Unknown | Negative | 1.00 | 1.00 | |
| ☑ | D9 | blank | Unknown | Negative | 1.00 | 1.00 | |
| ☑ | D10 | blank | Unknown | Negative | 1.00 | 1.00 | |
| ☑ | D11 | blank | Unknown | Negative | 1.00 | 1.00 | |
| ☑ | D12 | blank | Unknown | Negative | 1.00 | 1.00 | |
| ☑ | E1 | FCER1A 13 | Unknown | 3 | 0.99 | 0.80 | |
| ☑ | E2 | FCER1A 13 | Unknown | Unknown | 0.66 | 0.20 | |
| ☑ | E3 | FCER1A 13 | Unknown | 3 | 0.99 | 0.79 | |
| ☑ | E4 | blank | Unknown | Negative | 1.00 | 1.00 | |
| ☑ | E5 | blank | Unknown | Negative | 1.00 | 1.00 | |
| ☑ | E6 | blank | Unknown | Negative | 1.00 | 1.00 | |
| ☑ | E7 | blank | Unknown | Negative | 1.00 | 1.00 | |
| ☑ | E8 | blank | Unknown | Negative | 1.00 | 1.00 | |
| ☑ | E9 | blank | Unknown | Negative | 1.00 | 1.00 | |
| ☑ | E10 | blank | Unknown | Negative | 1.00 | 1.00 | |
| ☑ | E11 | blank | Unknown | Negative | 1.00 | 1.00 | |
| ☑ | E12 | blank | Unknown | Negative | 1.00 | 1.00 | |
| ☑ | F1 | RGS1 13 | Unknown | 1 | 1.00 | 0.92 | |
| ☑ | F2 | RGS1 13 | Unknown | 1 | 0.97 | 0.90 | |
| ☑ | F3 | RGS1 13 | Unknown | 1 | 0.98 | 0.90 | |
| ☑ | F4 | blank | Unknown | Negative | 1.00 | 1.00 | |
| ☑ | F5 | blank | Unknown | Negative | 1.00 | 1.00 | |
| ☑ | F6 | blank | Unknown | Negative | 1.00 | 1.00 | |
| ☑ | F7 | blank | Unknown | Negative | 1.00 | 1.00 | |
| ☑ | F8 | blank | Unknown | Negative | 1.00 | 1.00 | |
| ☑ | F9 | blank | Unknown | Negative | 1.00 | 1.00 | |

**Results**

| Inc | Pos. | Sample Name | Sample Type | Group | Score | Resolution | Status |
|---|---|---|---|---|---|---|---|
| ☑ | F10 | blank | Unknown | Negative | 1.00 | 1.00 | |
| ☑ | F11 | blank | Unknown | Negative | 1.00 | 1.00 | |
| ☑ | F12 | blank | Unknown | Negative | 1.00 | 1.00 | |
| ☑ | G1 | blank | Unknown | Negative | 1.00 | 1.00 | |
| ☑ | G2 | blank | Unknown | Negative | 1.00 | 1.00 | |
| ☑ | G3 | blank | Unknown | Negative | 1.00 | 1.00 | |
| ☑ | G4 | blank | Unknown | Negative | 1.00 | 1.00 | |
| ☑ | G5 | blank | Unknown | Negative | 1.00 | 1.00 | |
| ☑ | G6 | blank | Unknown | Negative | 1.00 | 1.00 | |
| ☑ | G7 | blank | Unknown | Negative | 1.00 | 1.00 | |
| ☑ | G8 | blank | Unknown | Negative | 1.00 | 1.00 | |
| ☑ | G9 | blank | Unknown | Negative | 1.00 | 1.00 | |
| ☑ | G10 | blank | Unknown | Negative | 1.00 | 1.00 | |
| ☑ | G11 | blank | Unknown | Negative | 1.00 | 1.00 | |
| ☑ | G12 | blank | Unknown | Negative | 1.00 | 1.00 | |
| ☑ | H1 | blank | Unknown | Negative | 1.00 | 1.00 | |
| ☑ | H2 | blank | Unknown | Negative | 1.00 | 1.00 | |
| ☑ | H3 | blank | Unknown | Negative | 1.00 | 1.00 | |
| ☑ | H4 | blank | Unknown | Negative | 1.00 | 1.00 | |
| ☑ | H5 | blank | Unknown | Negative | 1.00 | 1.00 | |
| ☑ | H6 | blank | Unknown | Negative | 1.00 | 1.00 | |
| ☑ | H7 | blank | Unknown | Negative | 1.00 | 1.00 | |
| ☑ | H8 | blank | Unknown | Negative | 1.00 | 1.00 | |
| ☑ | H9 | blank | Unknown | Negative | 1.00 | 1.00 | |
| ☑ | H10 | blank | Unknown | Negative | 1.00 | 1.00 | |
| ☑ | H11 | blank | Unknown | Negative | 1.00 | 1.00 | |
| ☑ | H12 | blank | Unknown | Negative | 1.00 | 1.00 | |

| **Group** | 1 |
|---|---|

| Inc | Pos | Sample Name | Sample Type | Score | Resolution | Status |
|---|---|---|---|---|---|---|
| ☑ | C1 | RGS1 9 | Unknown | 0.90 | 0.84 | |
| ☑ | C2 | RGS1 9 | Unknown | 0.95 | 0.90 | |
| ☑ | C3 | RGS1 9 | Unknown | 0.95 | 0.89 | |
| ☑ | C4 | RGS1 10 | Unknown | 0.98 | 0.91 | |
| ☑ | C5 | RGS1 10 | Unknown | 0.99 | 0.92 | |
| ☑ | C6 | RGS1 10 | Unknown | 0.98 | 0.88 | |
| ☑ | C7 | RGS1 11 | Unknown | 0.99 | 0.91 | |
| ☑ | C8 | RGS1 11 | Unknown | 0.98 | 0.89 | |

| Group | 1 |
|---|---|

| Inc | Pos | Sample Name | Sample Type | Score | Resolution | Status |
|---|---|---|---|---|---|---|
| ☑ | C9 | RGS1 11 | Unknown | 0.96 | 0.90 | |
| ☑ | C10 | RGS1 12 | Unknown | 0.99 | 0.90 | |
| ☑ | C11 | RGS1 12 | Unknown | 0.99 | 0.93 | |
| ☑ | C12 | RGS1 12 | Unknown | 0.99 | 0.92 | |
| ☑ | F1 | RGS1 13 | Unknown | 1.00 | 0.92 | |
| ☑ | F2 | RGS1 13 | Unknown | 0.97 | 0.90 | |
| ☑ | F3 | RGS1 13 | Unknown | 0.98 | 0.90 | |

| Group | 2 |
|---|---|

| Inc | Pos | Sample Name | Sample Type | Score | Resolution | Status |
|---|---|---|---|---|---|---|
| ☑ | A1 | GAPDH 9 | Unknown | 0.99 | 0.89 | |
| ☑ | A2 | GAPDH 9 | Unknown | 0.99 | 0.83 | |
| ☑ | A3 | GAPDH 9 | Unknown | 0.99 | 0.83 | |
| ☑ | A4 | GAPDH 10 | Unknown | 0.98 | 0.80 | |
| ☑ | A5 | GAPDH 10 | Unknown | 0.98 | 0.82 | |
| ☑ | A6 | GAPDH 10 | Unknown | 0.98 | 0.84 | |
| ☑ | A7 | GAPDH 11 | Unknown | 0.97 | 0.75 | |
| ☑ | A8 | GAPDH 11 | Unknown | 0.99 | 0.80 | |
| ☑ | A9 | GAPDH 11 | Unknown | 0.99 | 0.83 | |
| ☑ | A10 | GAPDH 12 | Unknown | 1.00 | 0.82 | |
| ☑ | A11 | GAPDH 12 | Unknown | 0.99 | 0.80 | |
| ☑ | A12 | GAPDH 12 | Unknown | 0.99 | 0.78 | |
| ☑ | D1 | GAPDH 13 | Unknown | 0.99 | 0.82 | |
| ☑ | D2 | GAPDH 13 | Unknown | 0.99 | 0.81 | |
| ☑ | D3 | GAPDH 13 | Unknown | 0.98 | 0.91 | |

| Group | 3 |
|---|---|

| Inc | Pos | Sample Name | Sample Type | Score | Resolution | Status |
|---|---|---|---|---|---|---|
| ☑ | B1 | FCER1A 9 | Unknown | 0.93 | 0.64 | |
| ☑ | B2 | FCER1A 9 | Unknown | 0.99 | 0.77 | |
| ☑ | B3 | FCER1A 9 | Unknown | 0.99 | 0.76 | |
| ☑ | B4 | FCER1A 10 | Unknown | 0.98 | 0.83 | |
| ☑ | B5 | FCER1A 10 | Unknown | 0.98 | 0.82 | |
| ☑ | B6 | FCER1A 10 | Unknown | 0.99 | 0.77 | |
| ☑ | B7 | FCER1A 11 | Unknown | 0.98 | 0.78 | |
| ☑ | B8 | FCER1A 11 | Unknown | 0.99 | 0.82 | |
| ☑ | B9 | FCER1A 11 | Unknown | 0.99 | 0.76 | |
| ☑ | B10 | FCER1A 12 | Unknown | 1.00 | 0.82 | |

| Group | 3 |
|---|---|

| Inc | Pos | Sample Name | Sample Type | Score | Resolution | Status |
|---|---|---|---|---|---|---|
| ☑ | B11 | FCER1A 12 | Unknown | 0.97 | 0.76 | |
| ☑ | B12 | FCER1A 12 | Unknown | 0.99 | 0.81 | |
| ☑ | E1 | FCER1A 13 | Unknown | 0.99 | 0.80 | |
| ☑ | E3 | FCER1A 13 | Unknown | 0.99 | 0.79 | |

| Group | Negative |
|---|---|

| Inc | Pos | Sample Name | Sample Type | Score | Resolution | Status |
|---|---|---|---|---|---|---|
| ☑ | D4 | blank | Unknown | 1.00 | 1.00 | |
| ☑ | D5 | blank | Unknown | 1.00 | 1.00 | |
| ☑ | D6 | blank | Unknown | 1.00 | 1.00 | |
| ☑ | D7 | blank | Unknown | 1.00 | 1.00 | |
| ☑ | D8 | blank | Unknown | 1.00 | 1.00 | |
| ☑ | D9 | blank | Unknown | 1.00 | 1.00 | |
| ☑ | D10 | blank | Unknown | 1.00 | 1.00 | |
| ☑ | D11 | blank | Unknown | 1.00 | 1.00 | |
| ☑ | D12 | blank | Unknown | 1.00 | 1.00 | |
| ☑ | E4 | blank | Unknown | 1.00 | 1.00 | |
| ☑ | E5 | blank | Unknown | 1.00 | 1.00 | |
| ☑ | E6 | blank | Unknown | 1.00 | 1.00 | |
| ☑ | E7 | blank | Unknown | 1.00 | 1.00 | |
| ☑ | E8 | blank | Unknown | 1.00 | 1.00 | |
| ☑ | E9 | blank | Unknown | 1.00 | 1.00 | |
| ☑ | E10 | blank | Unknown | 1.00 | 1.00 | |
| ☑ | E11 | blank | Unknown | 1.00 | 1.00 | |
| ☑ | E12 | blank | Unknown | 1.00 | 1.00 | |
| ☑ | F4 | blank | Unknown | 1.00 | 1.00 | |
| ☑ | F5 | blank | Unknown | 1.00 | 1.00 | |
| ☑ | F6 | blank | Unknown | 1.00 | 1.00 | |
| ☑ | F7 | blank | Unknown | 1.00 | 1.00 | |
| ☑ | F8 | blank | Unknown | 1.00 | 1.00 | |
| ☑ | F9 | blank | Unknown | 1.00 | 1.00 | |
| ☑ | F10 | blank | Unknown | 1.00 | 1.00 | |
| ☑ | F11 | blank | Unknown | 1.00 | 1.00 | |
| ☑ | F12 | blank | Unknown | 1.00 | 1.00 | |
| ☑ | G1 | blank | Unknown | 1.00 | 1.00 | |
| ☑ | G2 | blank | Unknown | 1.00 | 1.00 | |
| ☑ | G3 | blank | Unknown | 1.00 | 1.00 | |
| ☑ | G4 | blank | Unknown | 1.00 | 1.00 | |

| Group | Negative |
|---|---|

| Inc | Pos | Sample Name | Sample Type | Score | Resolution | Status |
|---|---|---|---|---|---|---|
| ☑ | G5 | blank | Unknown | 1.00 | 1.00 | |
| ☑ | G6 | blank | Unknown | 1.00 | 1.00 | |
| ☑ | G7 | blank | Unknown | 1.00 | 1.00 | |
| ☑ | G8 | blank | Unknown | 1.00 | 1.00 | |
| ☑ | G9 | blank | Unknown | 1.00 | 1.00 | |
| ☑ | G10 | blank | Unknown | 1.00 | 1.00 | |
| ☑ | G11 | blank | Unknown | 1.00 | 1.00 | |
| ☑ | G12 | blank | Unknown | 1.00 | 1.00 | |
| ☑ | H1 | blank | Unknown | 1.00 | 1.00 | |
| ☑ | H2 | blank | Unknown | 1.00 | 1.00 | |
| ☑ | H3 | blank | Unknown | 1.00 | 1.00 | |
| ☑ | H4 | blank | Unknown | 1.00 | 1.00 | |
| ☑ | H5 | blank | Unknown | 1.00 | 1.00 | |
| ☑ | H6 | blank | Unknown | 1.00 | 1.00 | |
| ☑ | H7 | blank | Unknown | 1.00 | 1.00 | |
| ☑ | H8 | blank | Unknown | 1.00 | 1.00 | |
| ☑ | H9 | blank | Unknown | 1.00 | 1.00 | |
| ☑ | H10 | blank | Unknown | 1.00 | 1.00 | |
| ☑ | H11 | blank | Unknown | 1.00 | 1.00 | |
| ☑ | H12 | blank | Unknown | 1.00 | 1.00 | |

| Group | Unknown |
|---|---|

| Inc | Pos | Sample Name | Sample Type | Score | Resolution | Status |
|---|---|---|---|---|---|---|
| ☑ | E2 | FCER1A 13 | Unknown | 0.66 | 0.20 | |

## Melting Curves

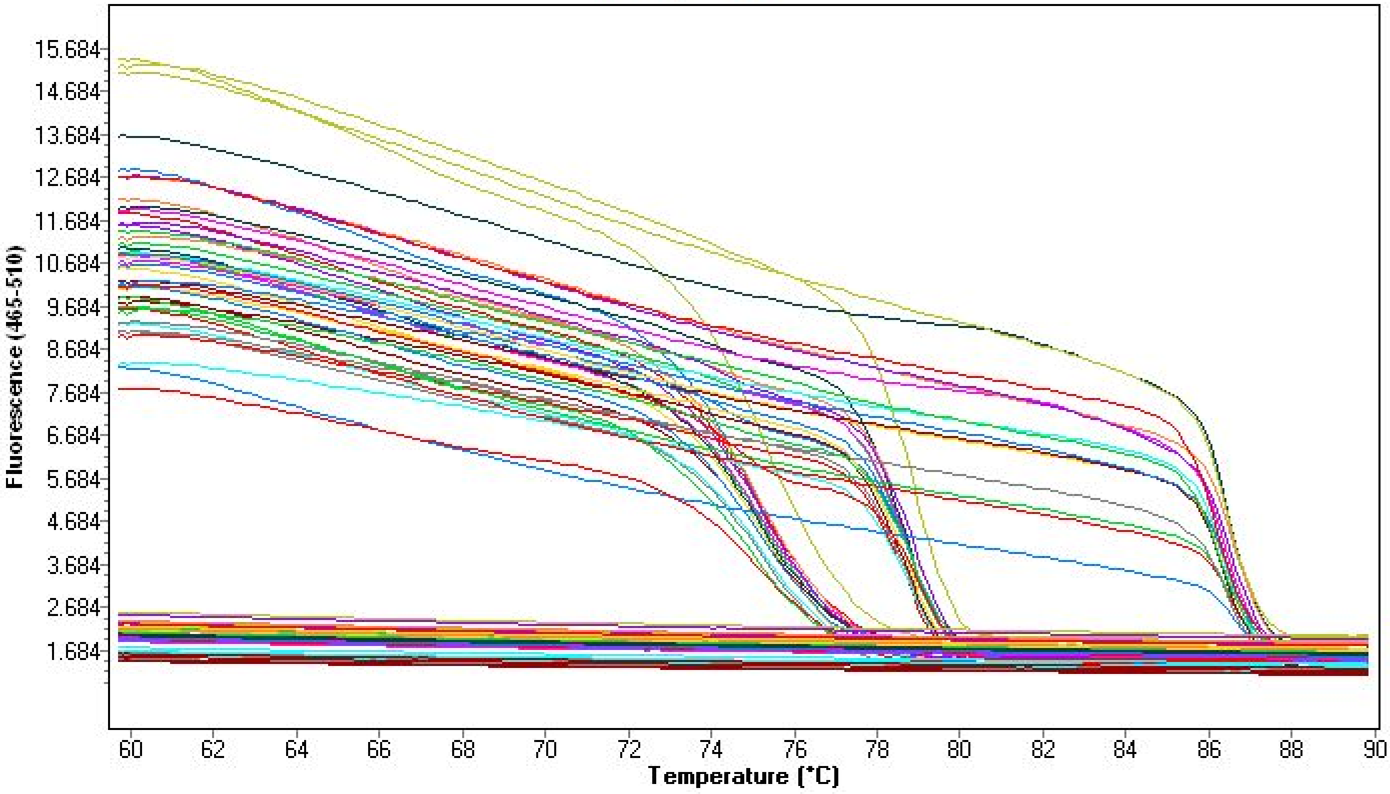


## Melting Peaks

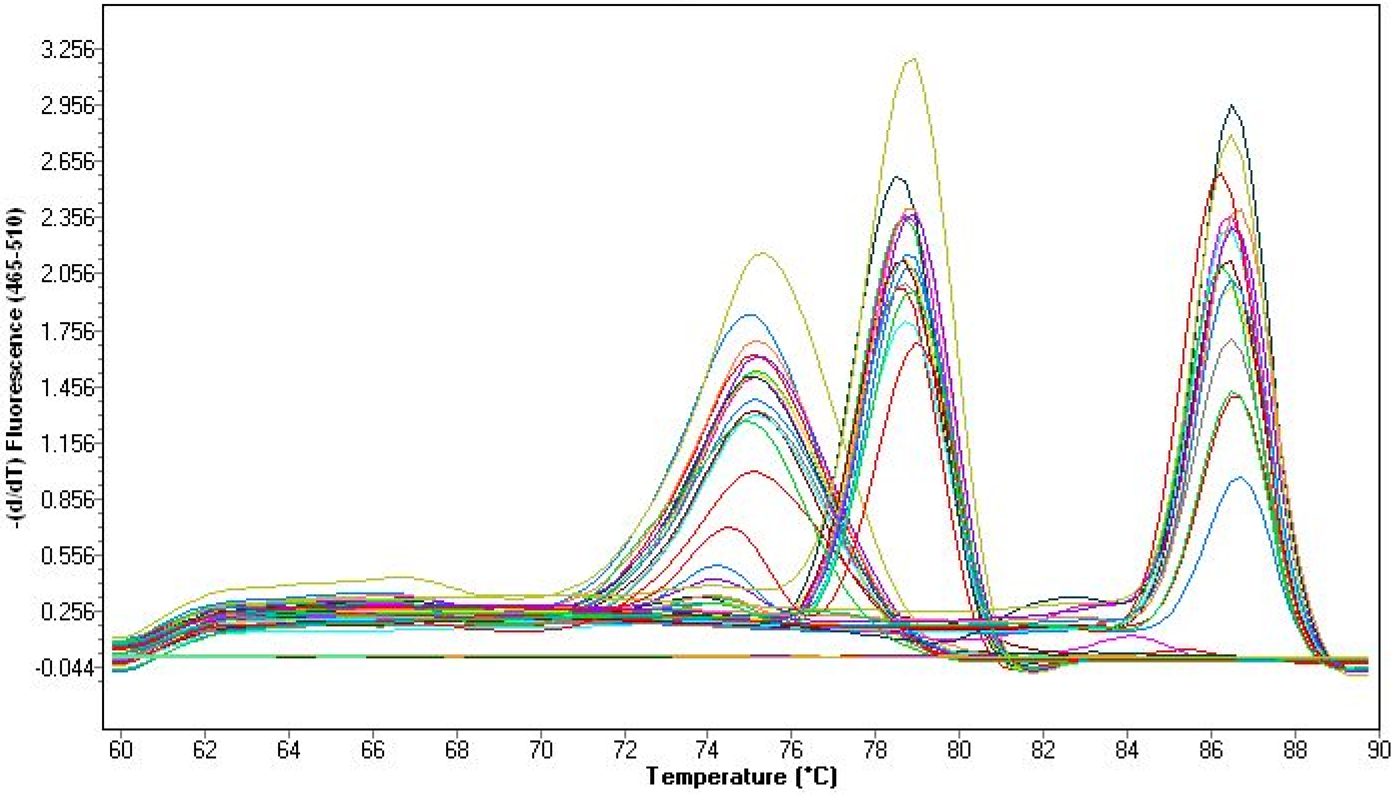


| **Analysis Notes** | |
|---|---|
