# Supplementary material for: Exploring differential gene expression and biomarker potential in systemic lupus erythematosus: a retrospective study
Source: PeerJ. 2025 Sep 8;13:e19891. doi: 10.7717/peerj.19891 (PMC12424612; doi:10.7717/peerj.19891)
Supplement: Supplemental Information 5 — Supplemental Table 1: Primer Sequences. Lists the primer sequences used for gene expression analysis, including GAPDH, FCER1A, and RGS1. Supplemental Table 2: Top 20 up-regulated and down-regulated genes in GSE13887 dataset. Presents the top 20 genes with the most significant up-regulation and down-regulation in the GSE13887 dataset, including their log2 fold change and p-values. Supplemental Table 3: Top 20 up-regulated and down-regulated genes in GSE10325 dataset. Lists the top 20 genes with the most significant up-regulation and down-regulation in the GSE10325 dataset, including their log2 fold change and p-values. Supplemental Table 4: Top 5 GO terms of each category for DEGs between Control and SLE. Summarizes the top 5 Gene Ontology (GO) terms for Biological Processes (BP), Cellular Components (CC), and Molecular Functions (MF) enriched in differentially expressed genes (DEGs) between control and SLE samples. Supplemental Table 5: Top 5 KEGG pathways for DEGs between Control and SLE. Highlights the top 5 KEGG pathways significantly enriched in DEGs between control and SLE samples, including the associated genes and p-values. [file peerj-13-19891-s005.docx]

**Exploring differential gene expression and biomarker potential in systemic lupus erythematosus**

**Supplementary Table**

Jie Xiao^#1^, Yuhong Luo^#1^, Lina Duan^#1^, Xinru Mao^1^, Lingyue Jin^1^, Haifang Wang^1^, Hongxia Wang^1^, Jie Pan^1^, Ying Gong*^1，2^ and Haixia Li*^1^

^#^Jie Xiao, Yuhong Luo and Lina Duan contributed equally to this work.

^1^Department of Laboratory Medicine, Guangdong Provincial Key Laboratory of Precision Medical Diagnostics, Guangdong Engineering and Technology Research Center for Rapid Diagnostic Biosensors, Guangdong Provincial Key Laboratory of Single-cell and Extracellular Vesicles, Nanfang Hospital, Southern Medical University, Guangzhou, 510515, P. R. China.

^2^ Department of Internal Medicine, Division of Hematology, Maastricht University Medical Center+, Maastricht, the Netherlands

Supplementary Table 1

Primer Sequences.

| Primer Name | Sequence（5’-3’） |
| --- | --- |
| *GAPDH* -FOR | CTCATGACCACAGTCCATGC |
| *GAPDH* -REV | TTCAGCTCTGGGATGACCTT |
| *FCER1A*-FOR | GTTCTTCGCTCCAGATGGC |
| *FCER1A*-REV | TTGTGGAACCATTTGGTGGAA |
| *RGS1*-FOR | GGCGCAGTCTTTTGACAAGC |
| *RGS1*-REV | GCCTTCTCGTCTACCACATGC |

Supplementary Table 2

Primer information.

| Primer Name | NCBI GeneID | GenBank Accession | Amplicon Size | Coding DNA Length | PrimerBank ID |
| --- | --- | --- | --- | --- | --- |
| *GAPDH* | 26330 | NM_014364 | 116 | 1227 | 126273608c1 |
| *FCER1A* | 2205 | NM_002001 | 153 | 774 | 345110626c1 |
| *RGS1* | 10287 | NM_001039467 | 156 | 654 | 86990434c1 |

Supplementary Table 3

Top 20 up-regulated and down-regulated genes in GSE13887 dataset.

| Gene Name | log_2_FoldChange | *p* value | up/down |
| --- | --- | --- | --- |
| *IL2* | -4.063767902 | 0.005857468 | down |
| *BYSL* | -3.357875717 | 8.96229E-06 | down |
| *ZBED2* | -3.144103067 | 0.036797758 | down |
| *LIF* | -3.018968341 | 0.005764205 | down |
| *PPP1R14B* | -2.99425037 | 7.71937E-05 | down |
| *UBE2M* | -2.807668685 | 0.001988916 | down |
| *SAC3D1* | -2.69791599 | 0.001299184 | down |
| *CXCL9* | -2.644775912 | 0.001798584 | down |
| *TSLP* | -2.608611634 | 1.61409E-06 | down |
| *IDO1* | -2.594791865 | 0.006870362 | down |
| *CCT5* | -2.592931433 | 0.000242426 | down |
| *WDR46* | -2.581595316 | 0.00011254 | down |
| *CCL22* | -2.476532666 | 0.024689265 | down |
| *UBE2T* | -2.440496957 | 0.004631799 | down |
| *INSM1* | -2.429210316 | 0.000889907 | down |
| *PARS2* | -2.38526852 | 0.000287159 | down |
| *PSAT1* | -2.350408767 | 0.000489685 | down |
| *PRG2* | -2.347699664 | 0.000595307 | down |
| *E2F5* | -2.341956189 | 0.000209071 | down |
| *NME1* | -2.314321723 | 0.001034307 | down |
| *NR4A2* | 3.66213329 | 0.000487272 | up |
| *FOSB* | 3.597704012 | 0.002972483 | up |
| *AF090939* | 3.273778096 | 0.000918876 | up |
| *PDCD4* | 3.120056464 | 0.001615251 | up |
| *F13A1* | 3.094931236 | 0.001381321 | up |
| *RAP1GAP2* | 3.06263928 | 0.00150205 | up |
| *RP11-489E7.4* | 3.050983773 | 0.000398261 | up |
| *ZNF331* | 3.021306058 | 0.000100692 | up |
| *TUBB1* | 2.867362624 | 0.005167409 | up |
| *LTF* | 2.816928304 | 0.005932442 | up |
| *LYPD3* | 2.707228419 | 0.000629832 | up |
| *ELOVL7* | 2.675263489 | 0.002927889 | up |
| *SDPR* | 2.623463755 | 0.005129112 | up |
| *YPEL5* | 2.580862388 | 6.72331E-05 | up |
| *FOS* | 2.566928502 | 0.006500405 | up |
| *PPBP* | 2.520522454 | 0.032194012 | up |
| *TRBV27* | 2.504911481 | 0.000966496 | up |
| *SIK1* | 2.502213638 | 0.000997844 | up |
| *HIST1H2AE* | 2.498407726 | 0.000251669 | up |
| *SPARC* | 2.447629371 | 0.002574334 | up |

Supplementary Table 4

Top 20 up-regulated and down-regulated genes in GSE10325 dataset.

| Name | log2FoldChange | *p* value | up/down |
| --- | --- | --- | --- |
| *PDGFRL* | -1.381863126 | 0.000205857 | down |
| *SYT17* | -1.3686922 | 0.00753647 | down |
| *PDZRN3* | -1.299061412 | 0.000869868 | down |
| *FCER1A* | -1.219140076 | 0.019927457 | down |
| *CRIP2* | -1.189430385 | 0.00796272 | down |
| *MIA* | -1.163218058 | 0.001000502 | down |
| *FOLR2* | -1.13492973 | 0.000979543 | down |
| *AGBL3* | -1.109523138 | 0.00113102 | down |
| *DPEP2* | -1.041799711 | 0.000858317 | down |
| *ZNF135* | -1.039503688 | 0.025100911 | down |
| *ZNF259P1* | -1.017159146 | 9.88879E-05 | down |
| *LAG3* | 1.028060622 | 0.016326122 | down |
| *RRM2* | 1.038622147 | 0.024927787 | down |
| *BIK* | 1.05984963 | 0.028529767 | down |
| *GADD45G* | 1.082690906 | 0.005532708 | down |
| *GZMB* | 1.08718234 | 0.00659748 | down |
| *PLSCR1* | 1.118171391 | 0.00054867 | down |
| *CXCL13* | 1.133791213 | 0.0057886 | down |
| *HERC6* | 1.145573801 | 0.000211816 | down |
| *APOL1* | 1.174226414 | 3.8269E-05 | down |
| *IFI44L* | 2.722909089 | 2.90651E-07 | up |
| *USP18* | 2.638155669 | 3.81672E-07 | up |
| *IFI27* | 2.50165831 | 4.15494E-06 | up |
| *HLA-DQA1* | 2.262146974 | 0.009544552 | up |
| *LAMP3* | 2.21281218 | 3.53006E-05 | up |
| *ISG15* | 1.994958347 | 9.44216E-09 | up |
| *RSAD2* | 1.879960529 | 4.35968E-07 | up |
| *HBB* | 1.842780238 | 0.000475755 | up |
| *RGS1* | 1.80690388 | 0.001397664 | up |
| *IFI44* | 1.785570684 | 8.05093E-07 | up |
| *IFIT1* | 1.775749133 | 0.001388335 | up |
| *SPATS2L* | 1.747368674 | 2.46045E-05 | up |
| *MX1* | 1.49254697 | 1.72007E-06 | up |
| *HERC5* | 1.43660638 | 1.37902E-07 | up |
| *IFI6* | 1.376758362 | 4.01309E-05 | up |
| *IFIT3* | 1.370831786 | 0.000106046 | up |
| *PIR* | 1.324702351 | 3.34406E-05 | up |
| *MCM10* | 1.308767459 | 0.005448905 | up |
| *DDX60* | 1.291554948 | 3.25203E-06 | up |
| *GZMH* | 1.273411732 | 0.045793582 | up |

Supplementary Table 5

Top 5 GO terms of each category for DEGs between Control and SLE.

| Category | Term | Genes | Count | *P* Value |
| --- | --- | --- | --- | --- |
| BP | GO:0042267~natural killer cell mediated cytotoxicity | *GZMB/LAG3* | 2 | 0.00 |
| BP | GO:0002228~natural killer cell mediated immunity | *GZMB/LAG3* | 2 | 0.00 |
| BP | GO:0001909~leukocyte mediated cytotoxicity | *GZMB/LAG3* | 2 | 0.00 |
| BP | GO:0001906~cell killing | *GZMB/LAG3* | 2 | 0.00 |
| BP | GO:0035768~endothelial cell chemotaxis to fibroblast growth factor | *CXCL13* | 1 | 0.00 |
| CC | GO:0044194~cytolytic granule | *GZMB* | 1 | 0.01 |
| CC | GO:0034361~very-low-density lipoprotein particle | *APOL1* | 1 | 0.01 |
| CC | GO:0034385~triglyceride-rich plasma lipoprotein particle | *APOL1* | 1 | 0.01 |
| CC | GO:0034364~high-density lipoprotein particle | *APOL1* | 1 | 0.01 |
| CC | GO:0034358~plasma lipoprotein particle | *APOL1* | 1 | 0.01 |
| MF | GO:0016805~dipeptidase activity | *DPEP2* | 1 | 0.01 |
| MF | GO:0045236~CXCR chemokine receptor binding | *CXCL13* | 1 | 0.01 |
| MF | GO:0017134~fibroblast growth factor binding | *CXCL13* | 1 | 0.01 |
| MF | GO:0001965~G-protein alpha-subunit binding | *RGS1* | 1 | 0.01 |
| MF | GO:0019865~immunoglobulin binding | *FCER1A* | 1 | 0.01 |

Supplementary Table 6

Top 5 KEGG pathways for DEGs between Control and SLE.

| Category | Term | Genes | Count | *P* Value |
| --- | --- | --- | --- | --- |
| KEGG_PATHWAY | hsa04210:Apoptosis | *GZMB/GADD45G* | 2 | 0.00 |
| KEGG_PATHWAY | hsa05202:Transcriptional misregulation in cancer | *GZMB/GADD45G* | 2 | 0.01 |
| KEGG_PATHWAY | hsa05310:Asthma | *FCER1A* | 1 | 0.02 |
| KEGG_PATHWAY | hsa05143:African trypanosomiasis | *APOL1* | 1 | 0.02 |
| KEGG_PATHWAY | hsa05216:Thyroid cancer | *GADD45G* | 1 | 0.02 |
