# Supplementary material for: Exploring differential gene expression and biomarker potential in systemic lupus erythematosus: a retrospective study
Source: PeerJ. 2025 Sep 8;13:e19891. doi: 10.7717/peerj.19891 (PMC12424612; doi:10.7717/peerj.19891)

Exploring differential gene expression and biomarker potential in systemic lupus erythematosus

Supplementary Figure

Jie Xiao[#1], Yuhong Luo[#1], Lina Duan[#1], Xinru Mao[1], Lingyue Jin[1], Haifang Wang[1], Hongxia Wang[1], Jie Pan[1], Ying Gong*[1, 2] and Haixia Li*[1]

[#]Jie Xiao, Yuhong Luo and Lina Duan contributed equally to this work.

[1]Department of Laboratory Medicine, Guangdong Provincial Key Laboratory of Precision Medical Diagnostics, Guangdong Engineering and Technology Research Center for Rapid Diagnostic Biosensors, Guangdong Provincial Key Laboratory of Single-cell and Extracellular Vesicles, Nanfang Hospital, Southern Medical University, Guangzhou, 510515, P. R. China.

[2] Department of Internal Medicine, Division of Hematology, Maastricht University Medical Center+, Maastricht, the Netherlands

Supplement Figure 1

Bubble plots of GO and KEGG pathway enrichment analysis results.

(A) The bubble plot shows the top 5 processes enriched in EP-DEGs in BPs, CCs, and MFs. (B) The bubble plot visualizes the top 5 significantly enriched KEGG pathways in DEGs (co-DEGs). The size of the ball represents the number of genes, and the color change of the ball corresponds to different *p*-values.

A

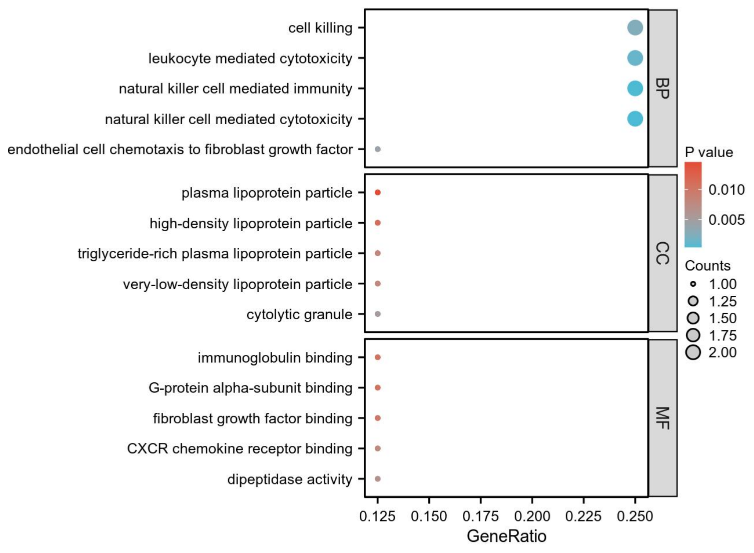

B

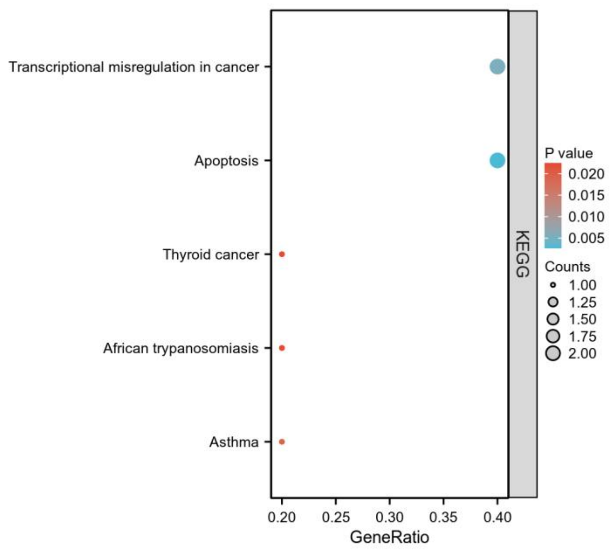

Supplement Figure 2

Construction of a Protein-protein interaction (PPI) network.

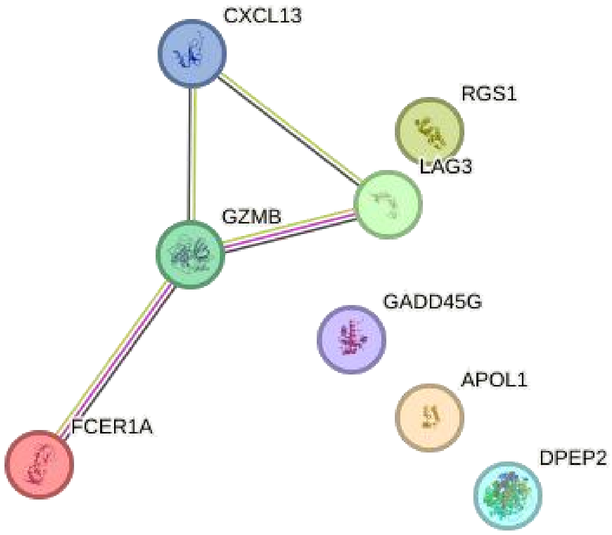

Supplement Figure 3

Gene validation in GSE10325 and GSE13887 datasets.

(A-G)Volcano plots of hub genes expressed in GSE10325 and GSE13887 datasets.

(H)Volcano plot of *FCER1A* and *RGS1*, genes with consistent expression levels in GSE10325 and GSE13887.

A

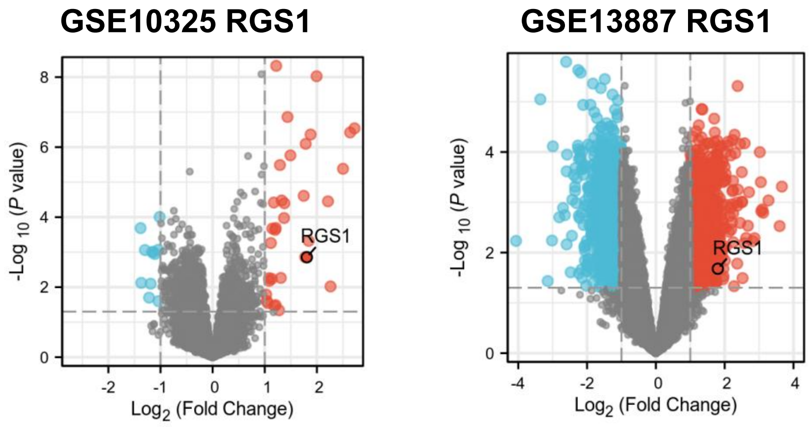

B

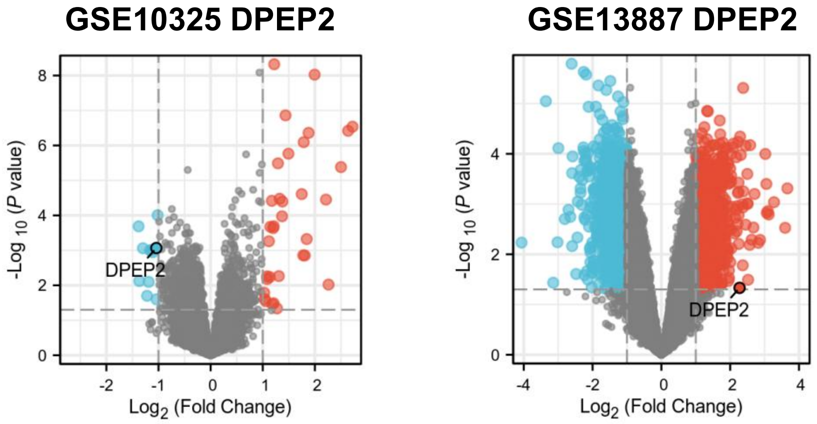

C

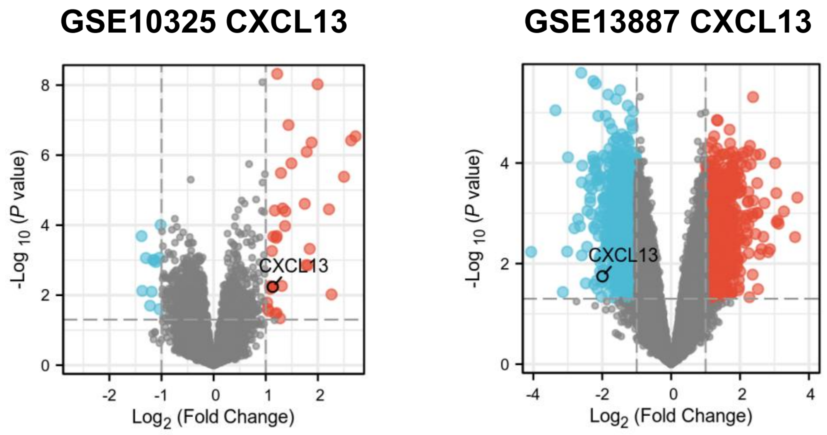

D

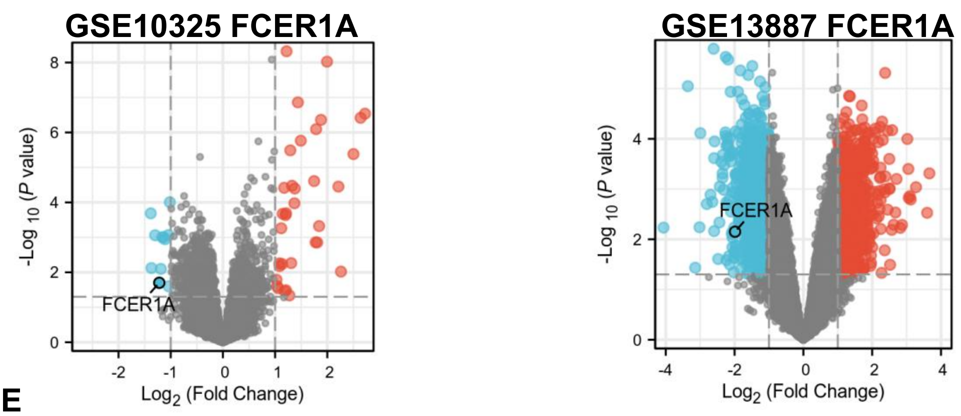
GSE10325 FCER1A
GSE13887 FCER1A
FCER1A
FCER1A
-Log 10 (P value)
Log2 (Fold Change)

E

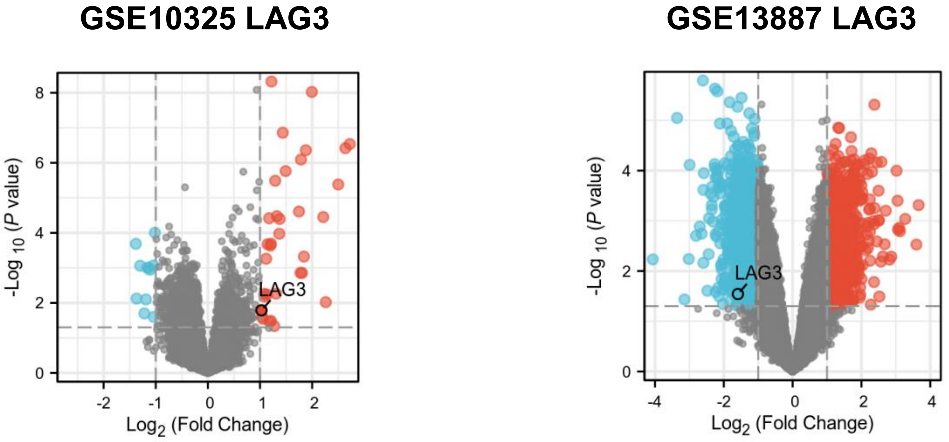
GSE10325 LAG3
GSE13887 LAG3
LAG3
LAG3
-Log 10 (P value)
Log2 (Fold Change)

F

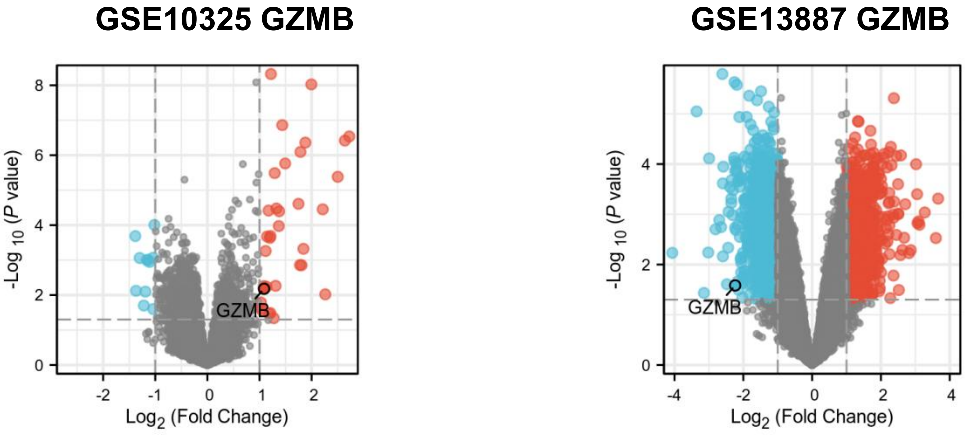
GSE10325 GZMB
GSE13887 GZMB
GZMB
GZMB
-Log 10 (P value)
Log2 (Fold Change)

G

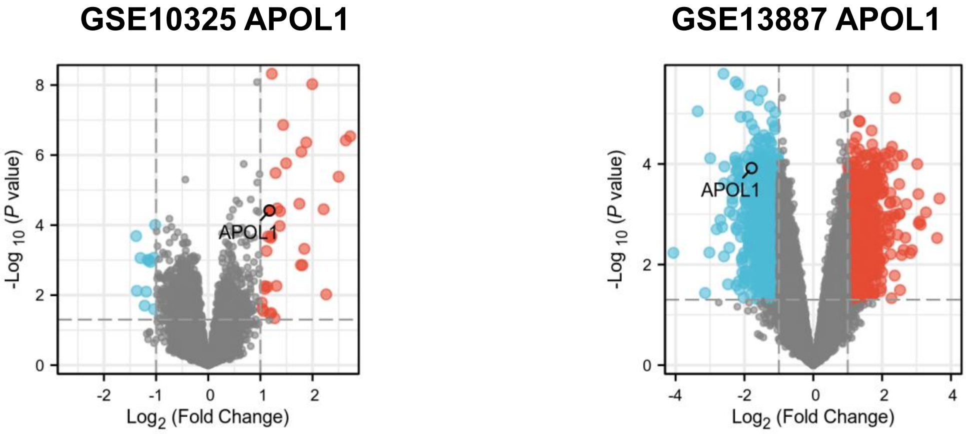
GSE10325 APOL1
GSE13887 APOL1
APOL1
APOL1
-Log 10 (P value)
Log2 (Fold Change)

H

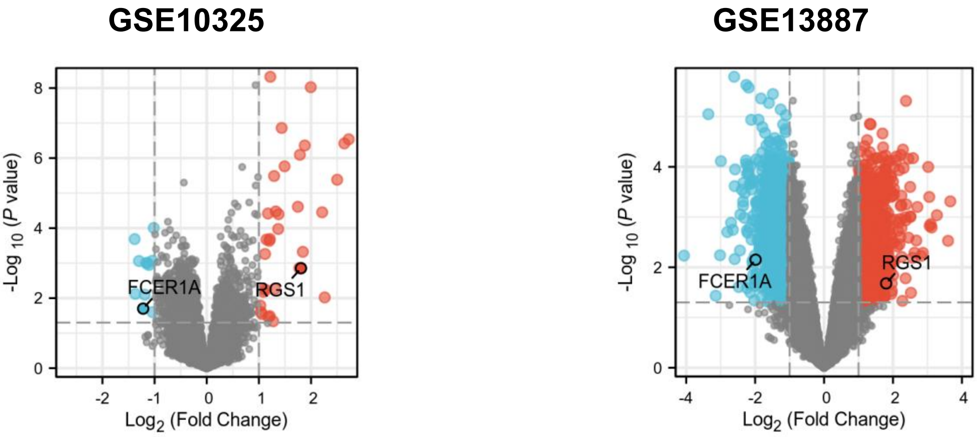
GSE10325
GSE13887
-Log 10 (P value)
Log2 (Fold Change)
FCER1A
RGS1

Supplement Figure 4

Construction of the FCER1A and RGS1 PPI network.

A B

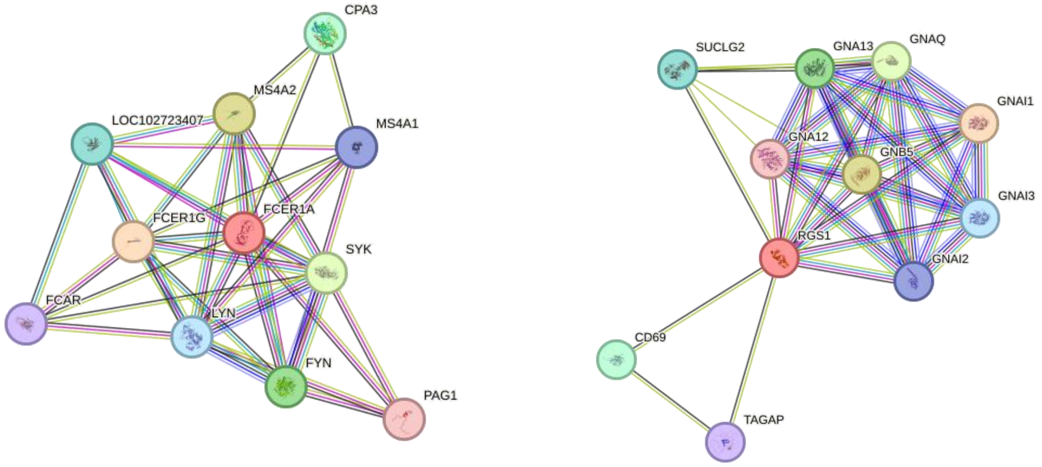

Supplement: Supplemental Information 6 — Supplemental Figure 1: Bubble plots of GO and KEGG pathway enrichment analysis results. (A) The bubble plot shows the top 5 processes enriched in EP-DEGs in Biological Processes (BPs), Cellular Components (CCs), and Molecular Functions (MFs). The size of the bubbles represents the number of genes, and the color corresponds to different p-values. (B) The bubble plot visualizes the top 5 significantly enriched KEGG pathways in DEGs (co-DEGs). The size of the bubbles represents the number of genes, and the color corresponds to different p-values. Supplement Figure 2: Construction of a Protein-protein Interaction (PPI) network. The PPI network highlights key genes such as CXCL13, RGS1, LAG3, GZMB, GADD45G, APOL1, DPEP2, and FCER1A, showing their interactions in the context of SLE. Supplemental Figure 3: Gene validation in GSE10325 and GSE13887 datasets. (A-G) Volcano plots of hub genes expressed in GSE10325 and GSE13887 datasets, showing differential expression patterns. (H) Volcano plot of FCER1A and RGS1, genes with consistent expression levels in GSE10325 and GSE13887 datasets. Supplemental Figure 4: Construction of the FCER1A and RGS1 PPI network. (A-B) The PPI network focuses on FCER1A and RGS1, illustrating their interactions and potential roles in SLE. [file peerj-13-19891-s006.pdf]
